# Molar Cervical Root Cross‐Sectional Morphology and Diet in Extant Catarrhines

**DOI:** 10.1002/ajpa.70164

**Published:** 2025-11-18

**Authors:** Zana R. Sims

**Affiliations:** ^1^ Department of Ecology, Evolution & Behavior University of Minnesota Saint Paul Minnesota USA

**Keywords:** dental, diet, feeding biomechanics, function, morphology

## Abstract

**Objectives:**

External tooth root morphology has demonstrated utility in understanding the mechanical function of teeth including patterns of loading during mastication and the overall mechanical challenge of foods consumed. Yet, there is still a paucity of information regarding how signals of diet, apart from these patterns, might be reflected in root form. This study examines whether a cross‐section taken from the molar root cervix contains signals related to diet and masticatory function in extant catarrhine primates.

**Methods:**

Micro‐CT scans of 11 genera of catarrhines were used to obtain 188 mandibular molars (M_1_, M_2_, and M_3_) from the right side. A single slice taken from the cervical margin was used to generate cross‐sectional properties for analysis.

**Results:**

OLS regression of dentin area on mandibular length provided evidence of a strong relationship (*r* ^2^ = 0.90–0.93, *p* < 0.001) and analysis of the residuals indicated significant differences for dietary category. Post hoc tests revealed that soft and hard object frugivores differ in relative dentin area across the molar row (*p* < 0.05). Additionally, a ratio calculated from the second moments of area *I*_x_ and *I*_y_ describing the cross‐sectional dentin distribution showed that dentin allocation was also significantly associated with dietary category serving to distinguish hard object frugivores from all other groups.

**Conclusions:**

The cervical root cross‐section contains information regarding both the mechanical function of a tooth as well as conveying some aspects of dietary specialization, particularly for frugivorous catarrhines. This region provides a more nuanced understanding of dental adaptation along the folivore–frugivore continuum.

## 1 Introduction

Analyses of dental crown morphology form the primary basis for much of what we understand about dietary adaptation in extant and fossil primates (Kay 1975; Rosenberger and Kinzey 1976; Molnar and Gantt 1977; Kay 1981; Martin 1985; Wood and Uytterschaut 1987; Anapol and Lee 1994; Wu et al. 2002; M'Kirera and Ungar 2003; Alba et al. 2010; Galbany et al. 2016; Schwartz et al. 2020). In contrast, studies that examine the dental root are far fewer in number, though the available literature has demonstrated links between root surface area and function (Kovacs 1979; Ward and Molnar 1980; Spencer 2003; Kupczik 2003; Kupczik and Dean 2008; Deutsch et al. 2022), root and crown volumes (Kupczik et al. 2009), and root shape and taxonomy (Wood et al. 1988; Kupczik and Hublin 2010; Le Cabec et al. 2013). Despite these promising findings, there are a few studies that examine the relationship between root shape, function, and specific dietary regimes in non‐human primates. The present study seeks to address some of the gaps in our knowledge through testing for correlations between diet and dentin shape variation about the root cervix in the mandibular molars of extant catarrhines.

Studies have shown that root surface anatomy can be used to infer masticatory function as predicted by Kovacs (1979). Experimental research indicated that loads incurred during mastication are not equally distributed across the root system in molars (Ward 1979; Ward and Molnar 1980). While work using extant taxa has demonstrated that measures of root size, including surface areas and linear dimensions, can be used to predict which teeth undergo larger masticatory loads during the processing of mechanically challenging foods (e.g., Spencer 2003; Kupczik 2003; Kupczik et al. 2018). Recently, Deutsch et al. (2022) investigated the relationship between root surface area, diet, and scaling variables including body mass confirming the relationship between root size and diets with high frequency loading.

The anatomy of roots has also been used to reconstruct the dietary ecology and function in extant and fossil taxa (e.g., Wood et al. 1988; Kupczik and Dean 2008; Le Cabec et al. 2013; Kupczik et al. 2018; Deane and Agosto 2025). Fossil species that fall outside of the morphological range of extant primates may be compared to additional mammalian taxa to make dietary inferences as Kupczik and Dean (2008) did for the extinct ape *Gigantopithecus blacki*. The analysis of tooth root form has also served to provide incidental support for hypotheses regarding oral processing behaviors (e.g., Le Cabec et al. 2013).

Likewise, research has indicated that the morphology of roots bears some phylogenetic significance. Variation in root dimensions and shape has been used to assess taxonomic affinity and classify unknown specimens (e.g., Abbott 1984; Wood et al. 1988; Le Cabec et al. 2013). For example, Le Cabec et al. (2013) examined root morphology in a variety of hominin species to associate unknown teeth with Neanderthals and early modern humans. This research demonstrated that root form can be beneficial for exploring hypotheses regarding ancestral character states and systematics.

Previous research provides consistent evidence that the tooth root dimensions, including surface area and diameter, can be a useful indicator of loading patterns across primate taxa. These data, however, are limited to basic functional inferences of loading magnitude and frequency. In addition, each of these methods relies on an assessment of the whole external root surface, requiring that roots be complete and relatively free from damage to enable reliable comparisons. While informative, these approaches greatly limit the analysis and interpretation of extant and fossil material by curtailing which specimens can be included in a sample. As evidenced by some of the extant data from studies presented above, species may engage in similar behaviors with disparate food items resulting in similar morphological adaptations (e.g., Spencer 2003; Kupczik 2003). This overlap may lead to incorrect interpretations of diet‐driven adaptations or ecological niche reconstructions.

The current study examines a two‐dimensional cross‐section of dentin taken at the root cervix to explicate the biomechanical function of the tooth in a region that has the ability to respond to individual in‐vivo use and hence is likely to reflect diet. The rationale for this location is three‐fold; first, this position allows for quantification of dentin distribution across the cervix, which must be sufficient to resist applied forces for effective load dissipation without failure (Biewener 1989).

Second, this position has potential developmental significance: the enamel organ of the developing tooth germ forms a distinctive ring of cells, where the inner and outer enamel epithelium meet, called the cervical loop (Hillson 2014). At the cervical loop, a portion of the enamel organ continues to lengthen toward the future root apex forming a temporary covering that surrounds what will become the root system, a structure known as Hertwig's Epithelial Root Sheath, or HERS (Butler 1956; Luan et al. 2006; Luder 2015). It is at this position that the initial deposition of dentin outside of the crown structure occurs, forming the future root cervix and first acellular layer of cementum (Thomas and Kollar 1989; Luder 2015; Li et al. 2017). As the earliest formation of dentin outside of the crown structure; it is under the strong influence of several genes, as evidenced by its persistent formation during knockout studies targeting the root system demonstrating that successful development of the root cervix and root system is critical to the functionality of the future tooth (Kovacs 1967; Zilberman and Smith 1992; Zhang et al. 2014; Ihn et al. 2021).

Finally, the pulp chamber, though not included in the measurements used in these analyses, houses the residual odontoblasts post‐development that produce tertiary dentin in response to insult from wear or infection altering the total volume of dentin, and this response has been demonstrated to vary in relation to diet (Yu and Abbott 2007; Towle 2019; Selig et al. 2021). All told, this location has the potential to be a useful alternative to crown‐based measures as it can be directly applied to broken teeth that are not well suited for other methods like shearing quotients or dental topographic analysis (e.g., Kay and Ungar 1997; Ungar and M'Kirera 2003).

## 2 Materials and Methods

### 2.1 Sample

The sample consists of 188 right‐side mandibular molars (M_1_ *n* = 63, M_2_ *n* = 65, M_3_ *n* = 60) from 11 genera of non‐human catarrhines across two families, Hominoidea and Cercopithecoidea. The timing of the split between these groups is estimated to have occurred ca. 30 million years ago (Steiper and Young 2006; Chatterjee et al. 2009). Within these two families, niche ecology as well as dietary adaptations of the sampled taxa are wide‐ranging (Table 1).

**TABLE 1:** List of primate genera included in the study.

Genus	*n*	Sex	Dietary Category	Reference(s)
*Cercocebus*	5	3 M 2 F	Hard object frugivore	Gautier‐Hion 1983; Hohmann 2009; Wieczkowski 2009
*Colobus*	5	3 M 2 F	Folivore	Harris and Chapman 2007; Thiery et al. 2017
*Erythrocebus*	4	4 M	Omnivore	Isbell 1998; Enstam and Isbell 2007
*Gorilla*	9	6 M 3 F	Mixed folivore	Remis 1997; Remis et al. 2001; Lodwick and Salmi 2019
*Hylobates*	9	4 M 5 F	Soft object frugivore	Palombit 1997; Bartlett et al. 2016
*Miopithecus*	5	3 M 2 F	Omnivore	Jones 1970; Gautier‐Hion 1983; Enstam and Isbell 2007
*Pan*	9	5 M 4 F	Soft object frugivore	Conklin‐Brittain et al. 2001; Moscovice et al. 2007
*Papio*	6	5 M 1 F	Omnivore	Hill and Dunbar 2002; Johnson et al. 2012
*Pongo*	9	4 M 5 F	Hard object frugivore	Fox et al. 2004; Hohmann 2009; Vogel et al. 2014
*Symphalangus*	3	2 M 1 F	Mixed folivore	Palombit 1997; Elder 2009; Hohmann 2009
*Theropithecus*	4	3 M 1 F	Folivore	Woldegeorgis and Bekele 2015; Jarvey et al. 2018

*Note:* Dietary category assignment and references used for this determination are provided. Individual specimen metadata is provided in the Supporting Information.

Data were collected from micro‐CT scans, many of which were obtained from MorphoSource (https://www.morphosource.org) from the Delson Primate scan and Copes et al. (2016) collections, augmented with specimens scanned by the author from the Harvard Museum of Comparative Zoology. This latter set was scanned at the Harvard Center for Nanoscale Systems using a Nikon HMX ST 225 Micro‐CT x‐ray imaging system with parameters ranging from 135 to 160 kV and 180 to 430 μA. All specimens were imaged with a 2.5 mm copper filter and a tungsten target to reduce beam hardening artifacts (Ketcham and Hanna 2014). Three individuals lacked available information on sex and were assigned to a category based on species‐matched comparisons of canine and facial sizes. Specimen information including taxonomy down to the level of subspecies (when available) and scan resolution are provided (Table S1).

Permanent teeth were identified based on two hierarchical criteria: first, molars had to be fully erupted, whereby all parts of the enamel crown sat above the alveolus (Schultz 1935); second, only those with the completed formation of the root apices and canals, verified through volume rendering of micro‐CT scans, were retained (Dean et al. 2022). Finally, the sample included molars with both damaged (e.g., broken or cracked) and heavily worn enamel crowns to assess the viability of applying these protocols to damaged fossil material.

### 2.2 Image Processing

Image stacks were imported into Dragonfly ORS version 2022.1 (Dragonfly ORS 2022) and adjusted manually using threshold values to reduce noise due to air or scanning artifacts (i.e., acrylic or foam scanning supports), and to enhance tissue contrasts on the 3D‐volume rendering of the mandibles.

**FIGURE 1:**
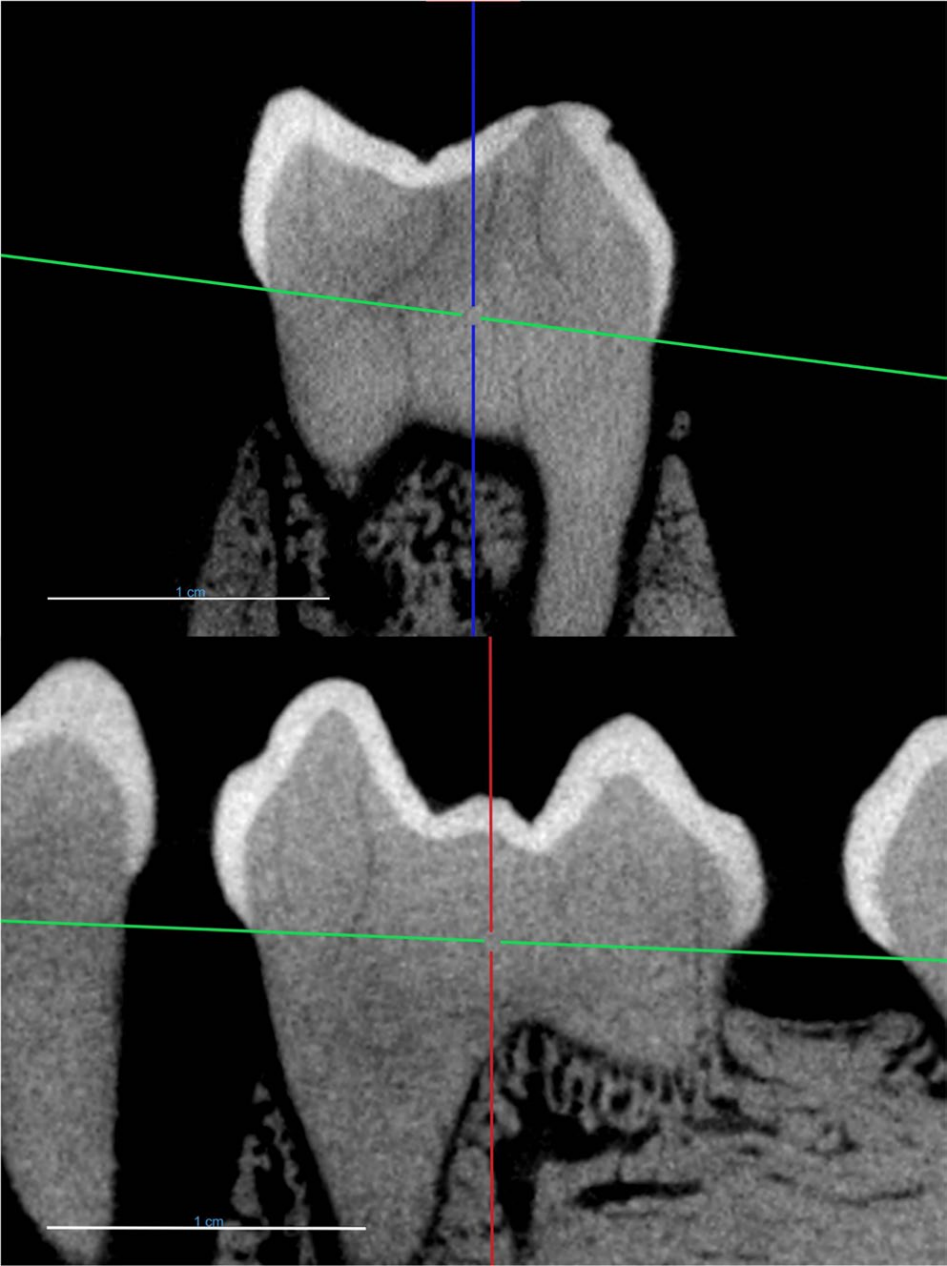
Identification of mesial margin (top) and lingual margin (bottom) of the M_2_ from specimen MCZ 20038. The red line indicates the coronal plane, the blue line indicates the sagittal plane, and the green line indicates the transverse plane. Scale bar = 1 cm.

**FIGURE 2:**
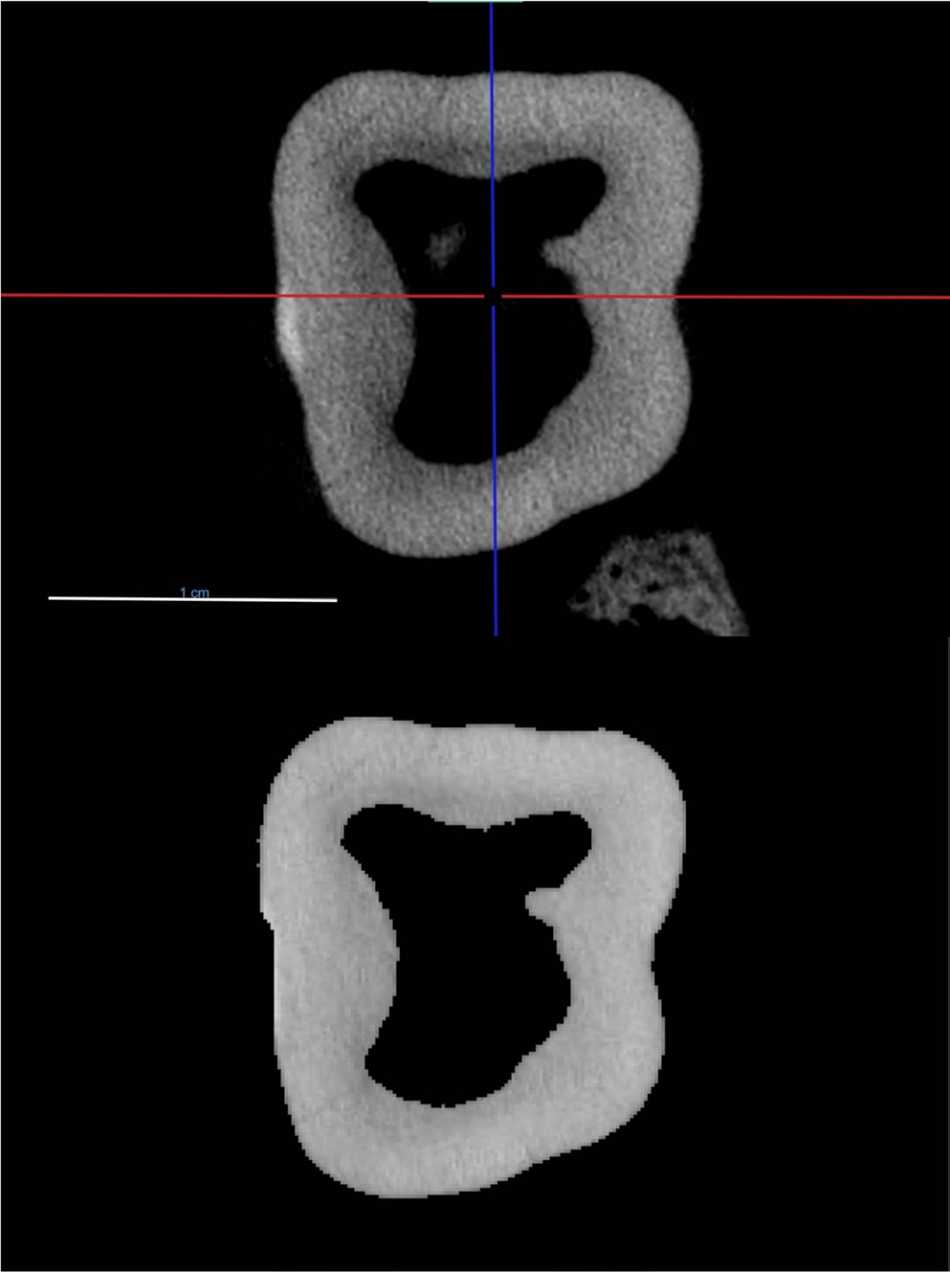
The aligned and resampled M_2_ from specimen MCZ 20038 after the process of apical navigation to minimize enamel (top). Reference planes as in Figure 1. Scale bar = 1 cm. The same cross‐section after post‐processing steps (bottom). Any enamel remnants and alveolar bone have been removed. Image is not scaled.

Individual molars were isolated and then manipulated across three independently controlled planar views (coronal, sagittal, and transverse). The most mesial and lingual margins of each molar's cementoenamel junction (CEJ) demarcate a unique plane that can be used to identify the dental cervix (Figure 1). Because the cervical plane does not lie in accordance with a true anatomical plane, the location of the dental cervix is approximated by this initial alignment performed along the coronal and sagittal views (Benazzi et al. 2012; Emonet et al. 2012). The image stack was resampled using the built‐in resample function with isotropic spacing to reorient the scan to the new alignment so that an individual slice of interest could be identified and exported for analysis. The stack slices were navigated along the transverse planar view moving apically to reach a position on the dental cervix that best minimized any visible apical enamel extensions (Olejniczak et al. 2007). The resulting cross‐section represents the portion of the root along the cervix whereby enamel is minimized, and the pulp chamber remains visible, hereafter referred to as the cervical root cross‐section (Figure 2, top).

The final step performed in Dragonfly was a measurement of the total mandibular length for each specimen. This was completed using the built‐in measurement tool and landmark points placed at the posterior‐most edge of the mandibular condyle and a second at infradentale, following Taylor et al. (2015). After landmark points were selected, a line was computed between them and recorded in millimeters. Dental variables scaled using features of the facial skeleton can reveal important information regarding dietary signals across clades, and with differing levels of prognathism, than demonstrated by the same variables when scaled using body mass (e.g., Scott 2011, 2012, 2021). For this study, mandibular length was selected as it represents one such biomechanically relevant variable that has previously been demonstrated to provide insight into diet‐driven size variation when used to scale dental variables like incisor width (Scott 2021).

### 2.3 Cross‐Section Measurement

The cross‐section slices for each molar were imported into Fiji by ImageJ (Schindelin et al. 2012) and the data were scaled prior to measurement collection using the voxel sizes obtained in the previous steps. Additional processing steps were undertaken for any slices that retained features that were not of interest (e.g., alveolar bone, enamel, adjacent dentition) before analysis (Figure 2, bottom).

Image J plugin Moment Macro (https://fae.johnshopkins.edu/chris‐ruff/) was used to obtain five variables of interest for each tooth position: dentin area (DA) a measure of cross‐sectional dentin to the exclusion of the pulp chamber, and the second moments of area (*I*_x_, *I*_y_, *I*_max_, and *I*_min_), which were used to compute ratios as measures of shape (Ruff and Hayes 1983; Ruff 1987; Ruff et al. 1999; Carlson 2005; Patel et al. 2013; Wilson and Humphrey 2015). In biomechanical applications, *I*_x_ and *I*_y_ are used to describe the distribution of material around a cross‐section (Wilson and Humphrey 2015). A ratio *I*_x_/*I*_y_ where the value is < 1.0 represents an increase of material in the BL direction, whereas a ratio > 1.0 represents an increase in the MD direction; a ratio of 1.0 indicates an equal distribution of material about the axes (Ruff and Hayes 1983; Ruff 1987; Ruff et al. 1999). The *I*_max_/*I*_min_ ratio provides a measure of the circularity of a cross‐section: where a circle is 1 and deviations represent a more elliptical form (Ruff 1987; Wilson and Humphrey 2015). As an indicator of cross‐sectional shape, this ratio has demonstrated functional significance for loading patterns in postcranial analyses (e.g., Carlson 2005; Patel et al. 2013). For this study, second moments of area were used to quantify both dentin distribution and cross‐sectional ‘shape,’ though it should be noted that molars are not well suited to beam modeling (see, Carlson 2005) and so no assessment of strength, rigidity, or bending was undertaken.

In this sample, the second moments of area *I*_x_ and *I*_y_ were measured about the anatomical axes, mesial‐distal (MD) and buccal‐lingual (BL), respectively. *I*_max_ and *I*_min_ were calculated using the greatest and least dimensions (principal axes) of the cross‐section as the reference plane.

### 2.4 Dietary Category Assignment

Most primates feed along a spectrum and, with few exceptions (e.g., Lambert 1998; Milton 1998), there is little that precludes a folivore from consuming fruits (e.g., *Gorilla*, Remis 1997), or a frugivore from consuming insects (e.g., *Pan*, Bogart and Pruetz 2011). It has been demonstrated that most primates will consume a range of the available food resources in their habitat such as fruits, leaves, bark, seeds, and flowers, which may be supplemented with insects or vertebrate prey (e.g., Conklin‐Brittain et al. 2001; Hohmann 2009). Primate diets are often augmented by other lesser preferred or lower quality food resources (i.e., fallback foods) and the proportion of their consumption can reflect seasonality and/or competition (e.g., Marshall and Wrangham 2007; Hohmann 2009; Bersacola et al. 2015).

For this study, a survey of primate ecology literature provided data that were used to assign each primate to a broad dietary category using the range of published consumption percentages (Table 1). Assignments were determined using an arbitrary cutoff of 60% or more for a single food item. The additional designation of ‘mixed’ was used for primates whose diets were split between two primary food items, each approaching 50% (e.g., fruit and leaves). The ‘omnivore’ category applied to primates whose diets include components that are not easily classified within traditional categories (e.g., flowers, gums, or tubers) and where no single food item exceeded the 60% threshold. While this may obscure some of the nuances of consumptive patterns, it simplifies comparisons to elicit general patterns in morphology.

Diets were partitioned as follows: folivores (*Colobus* and *Theropithecus*) had reported folivorous components > 75% (Harris and Chapman 2007; Woldegeorgis and Bekele 2015; Jarvey et al. 2018). Mixed folivores (*Gorilla* and *Symphalangus*) had significant fluctuations in reported fruit‐to‐foliage ranges, 43%–51% and 31%–56%, respectively (Remis 1997; Lodwick and Salmi 2019; Palombit 1997; Hohmann 2009). Soft object frugivores (*Hylobates* and *Pan*) both have high fruit components in their reported diets (50%–84% and 64%–93.5%, respectively), with the additional specification of preferring ripe, fleshy fruits (Hohmann 2009; Bartlett et al. 2016; Conklin‐Brittain et al. 2001; Moscovice et al. 2007). Hard object frugivores also have high fruit proportions (*Cercocebus*: 49%–78%; *Pongo*: 60%–68%) but are distinct in the consumption of fibrous and pithy fruits (Gautier‐Hion 1983; Wieczkowski 2009; Hohmann 2009; Fox et al. 2004; Vogel et al. 2014). Finally, omnivores were classified based on reported consumption of a variety of items with relatively high percentages: *Erythrocebus* reportedly consumes as little as 2%–6% of leaves but anywhere from 7%–65% of flowers, 5%–35% of insects, and 12%–37% of gums (Isbell 1998; Enstam and Isbell 2007); *Miopithecus* has been reported to consume less than 2% of leaves, around 43% of fruit, and 35% of insects, though much of what is known about this primate comes from just a few studies (Jones 1970; Gautier‐Hion 1983; Enstam and Isbell 2007). Reports for the diet of *Papio* range from 10%–59% fruit, 7%–53% leaves, 1%–21% flowers, up to 33% stems, 1%–27% tubers, and 1%–13% animal matter (Hill and Dunbar 2002; Johnson et al. 2012).

### 2.5 Analysis

All analyses were conducted in RStudio version 4.1.2 (R Core Team 2021), with statistical significance arbitrated at an alpha level of 0.05. To examine how dentin area scales across dietary categories, an ordinary least squares regression (OLS) was performed for dentin area on mandibular length (pooled sample and individual tooth positions) and another using genus average dentin area on genus average mandibular length. All data were natural log transformed. To understand differences in the cervical root cross section by diet, beyond scaling, residuals for dentin area regressed on mandibular length were further analyzed. These residuals plus the ratios for second moments of area (pooled sample and individual tooth positions) were subjected to tests for normality, homogeneity of variance, and multicollinearity (dentin area only) using the packages ‘olsrr’ and ‘car’ (Mason and Perreault 1991; Hebbali 2020; Fox and Weisberg 2019).

Because of the shared evolutionary history of clades represented in this sample, it is important to account for the potential effects of phylogeny on any patterns observed in the data. A phylogenetic generalized least squares regression (PGLS) was performed using the packages ‘caper,’ ‘phytools,’ and ‘ape’ (Orme et al. 2013; Revell 2012; Paradis and Schliep 2019). A maximum likelihood test was used to estimate phylogenetic signal measured as Pagel's λ, where *λ* = 1 represents strong phylogenetic signal for a trait and *λ* = 0 represents no phylogenetic signal under Brownian motion (Symonds and Blomberg 2014). If the phylogenetic signal in the residuals is absent (*λ* = 0), results for the PGLS will be the same as and OLS (Symonds and Blomberg 2014). To construct the model, a time‐calibrated consensus tree downloaded from the 10 k Trees website (Arnold et al. 2010).

A factorial analysis of variance (ANOVA) was performed to compare the effect of sex, diet, tooth position (pooled sample), and their interactions on the regression residuals. For ANOVA results, Eta squared (*η* ^2^) was calculated using the ‘lsr’ package (Navarro 2015) to test for the estimated effect size of each independent variable (Cohen 1973; Levine and Hullett 2002). A Tukey's Honestly Significant Difference (HSD) test for multiple comparisons was used to explore the pattern of differences for any ANOVA models that returned significant results.

Data for cross‐sectional ratios were pooled for all teeth to reduce the impact of unequal sample sizes for all dietary categories and to increase statistical power. Cross‐sectional ratios between dietary categories were analyzed using the non‐parametric Kruskal‐Wallis rank sum test. For tests that returned significant results, a non‐parametric pairwise Wilcoxon rank sum test with Benjamini‐Hochberg correction was applied to identify which groups were significantly different.

## 3 Results

**FIGURE 3:**
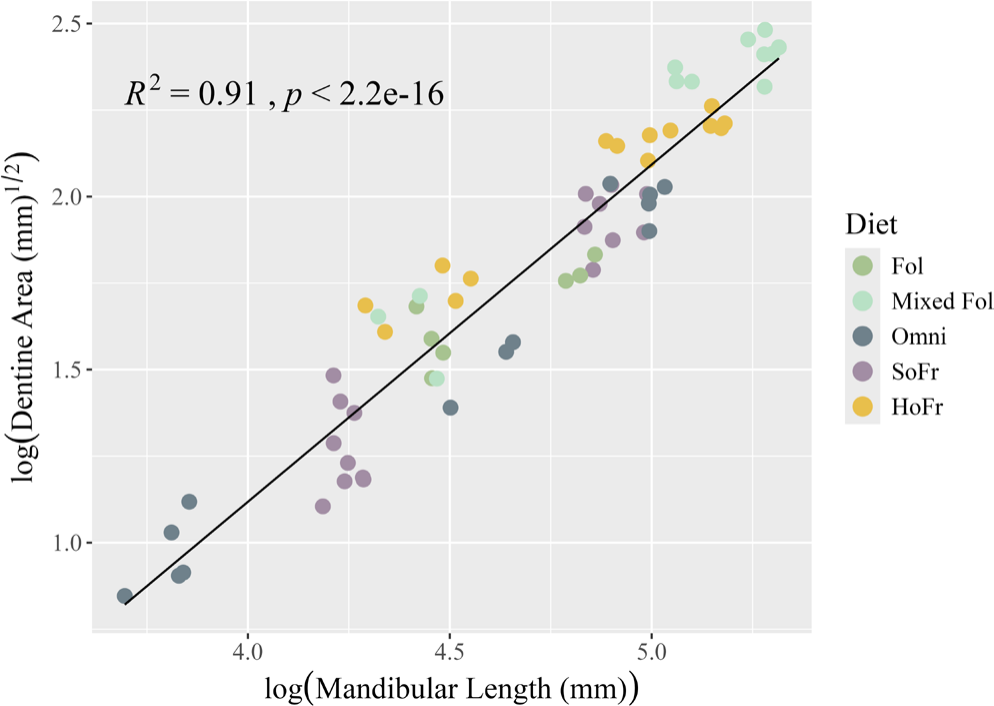
OLS linear regression of dentin area on mandibular length for the M_1_. All variables were natural log transformed (see text). Observations are colored by dietary category.

**FIGURE 4:**
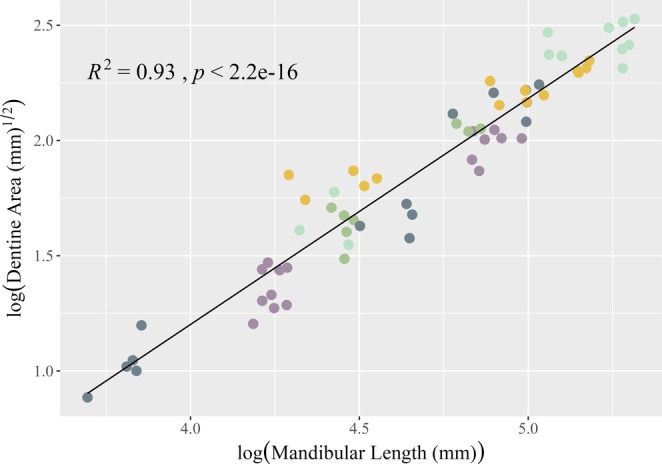
OLS linear regression of dentin area on mandibular length for the M_2_. All variables were natural log transformed. Observations are colored by dietary category, legend as in Figure 3.

**FIGURE 5:**
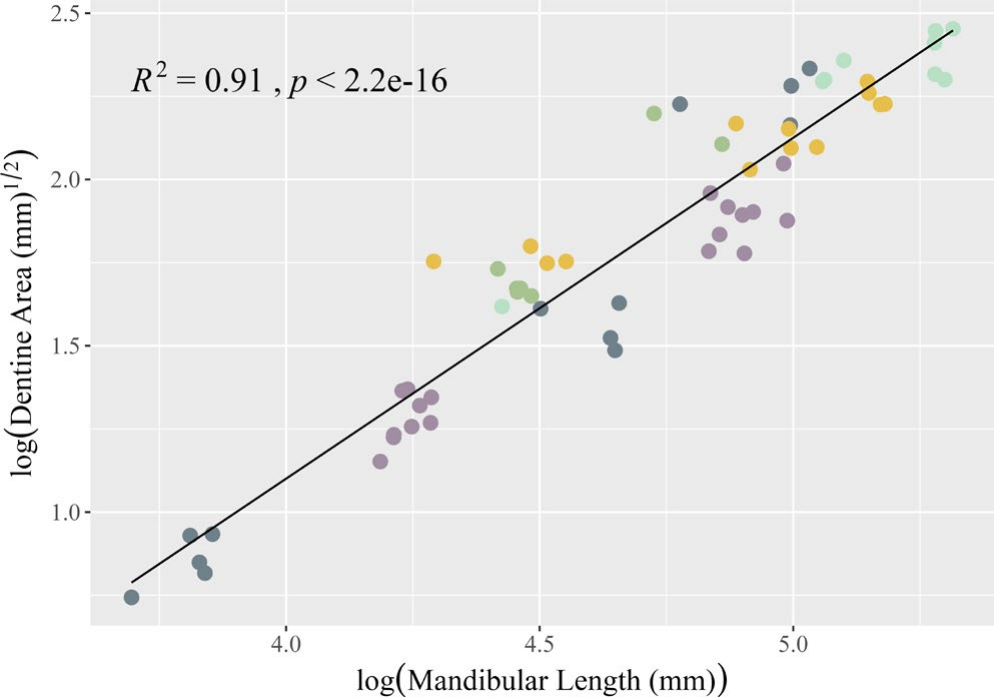
OLS linear regression of dentin area on mandibular length for the M_3_. All variables were natural log transformed. Observations are colored by dietary category, legend as in Figure 3.

**FIGURE 6:**
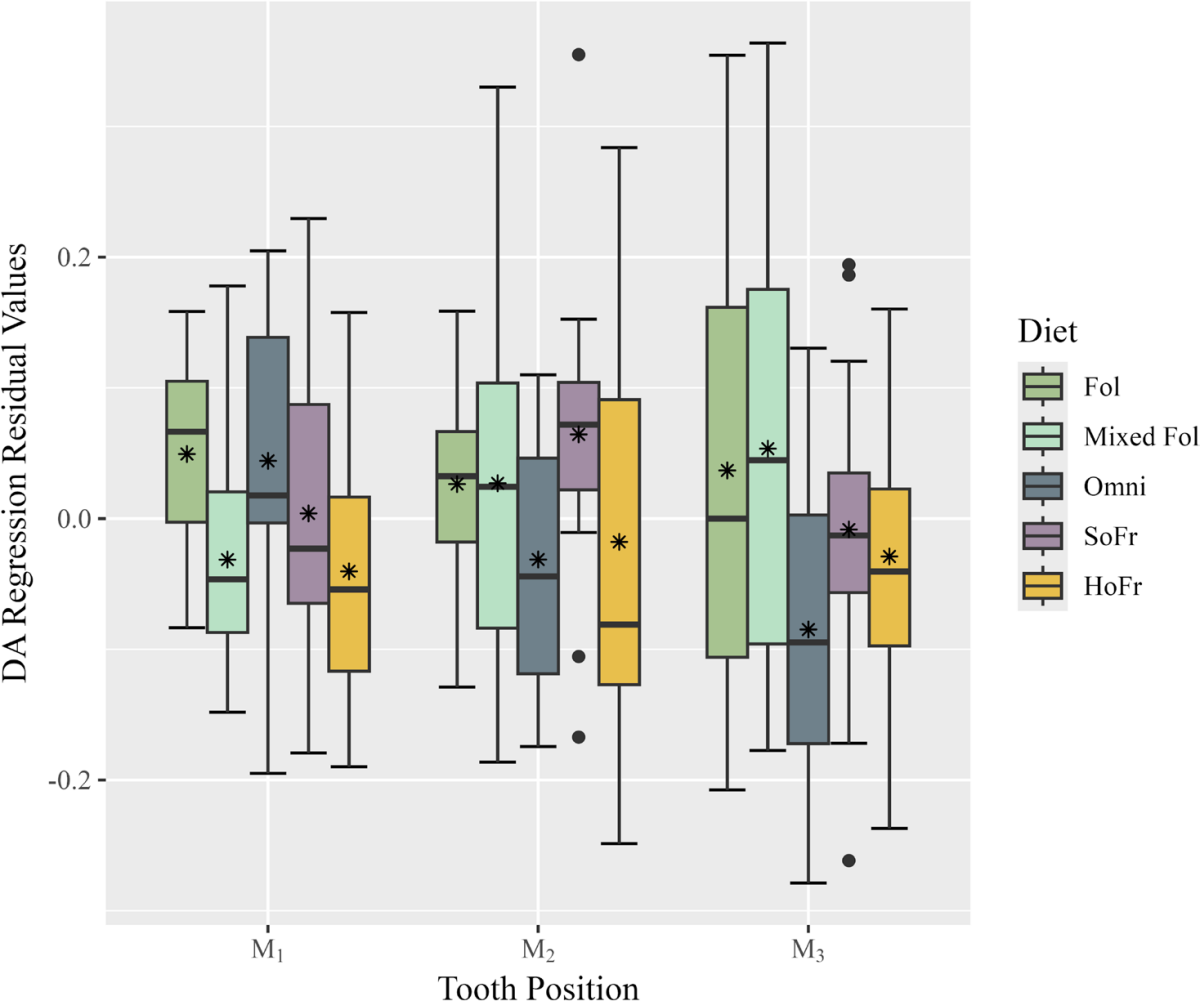
Boxplot of regression residuals for dentin area against mandibular length by dietary category and tooth position. Thick horizontal bars within each box represent the median and asterisk symbols represent mean values for each diet. Single black dots represent outliers.

**FIGURE 7:**
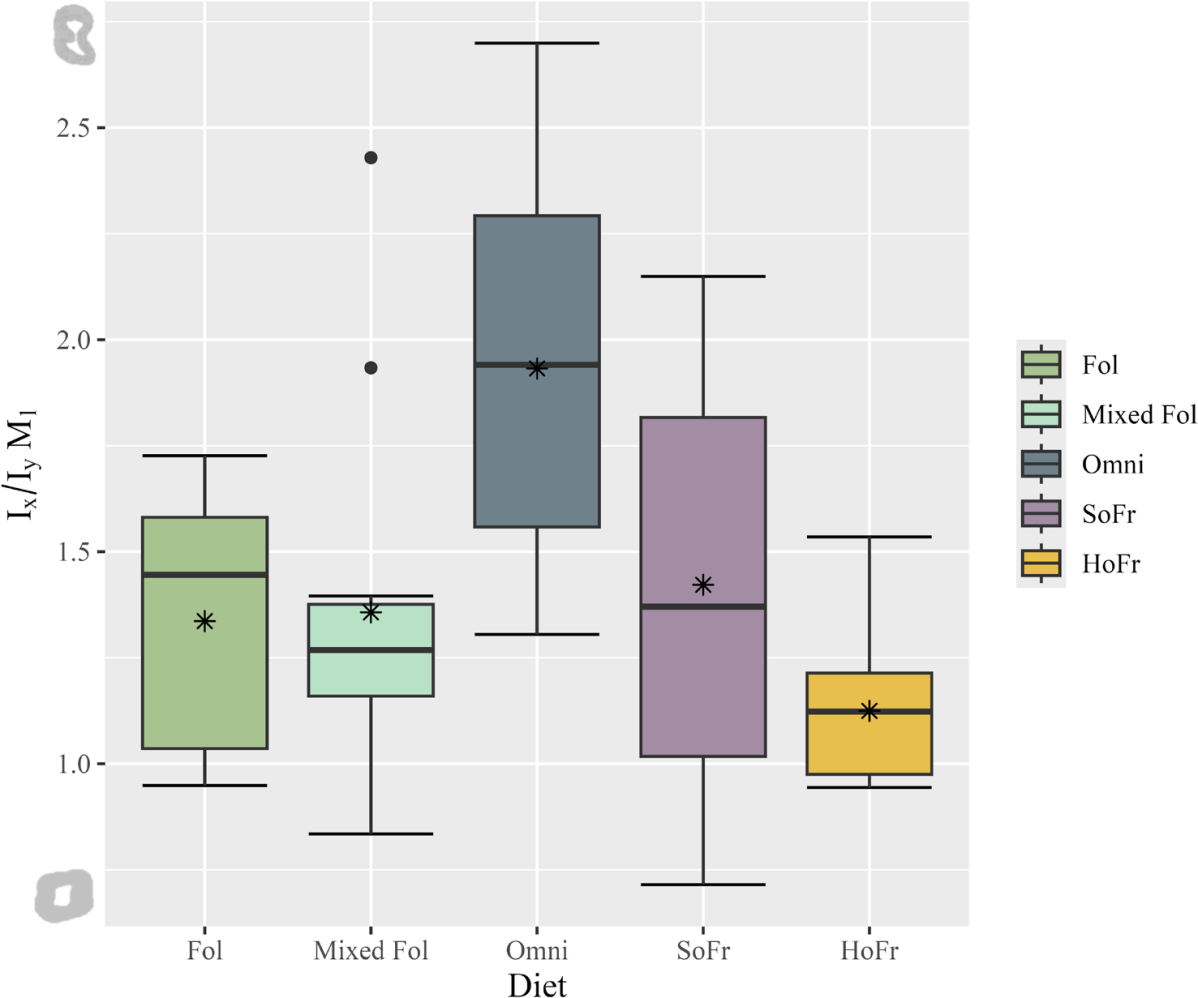
Boxplot of *I*_x_/*I*_y_ ratios for the M_1_ tooth position by dietary category. Thick horizontal bars within each box represent the median and asterisk symbols represent mean values for each diet. Single black dots represent outliers. Illustrations to the left of the Y‐axis represent the maximum and minimum *I*_x_/*I*_y_ ratio morphologies for M_1_.

**FIGURE 8:**
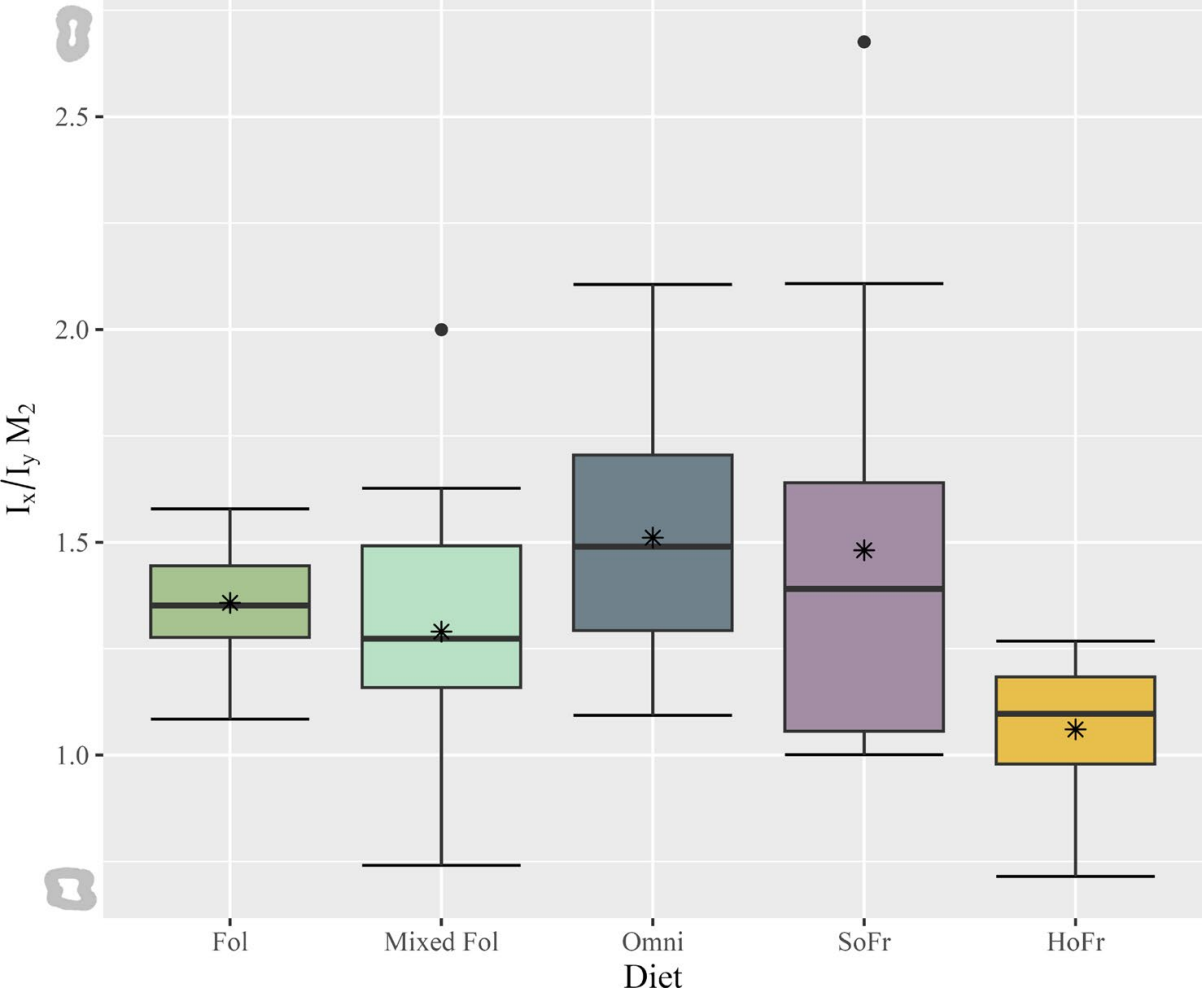
Boxplot of *I*_x_/*I*_y_ ratios for the M_2_ tooth position by dietary category. Thick horizontal bars within each box represent the median and asterisk symbols represent mean values for each diet. Single black dots represent outliers. Illustrations to the left of the Y‐axis represent the maximum and minimum *I*_x_/*I*_y_ ratio morphologies for M_2_.

**FIGURE 9:**
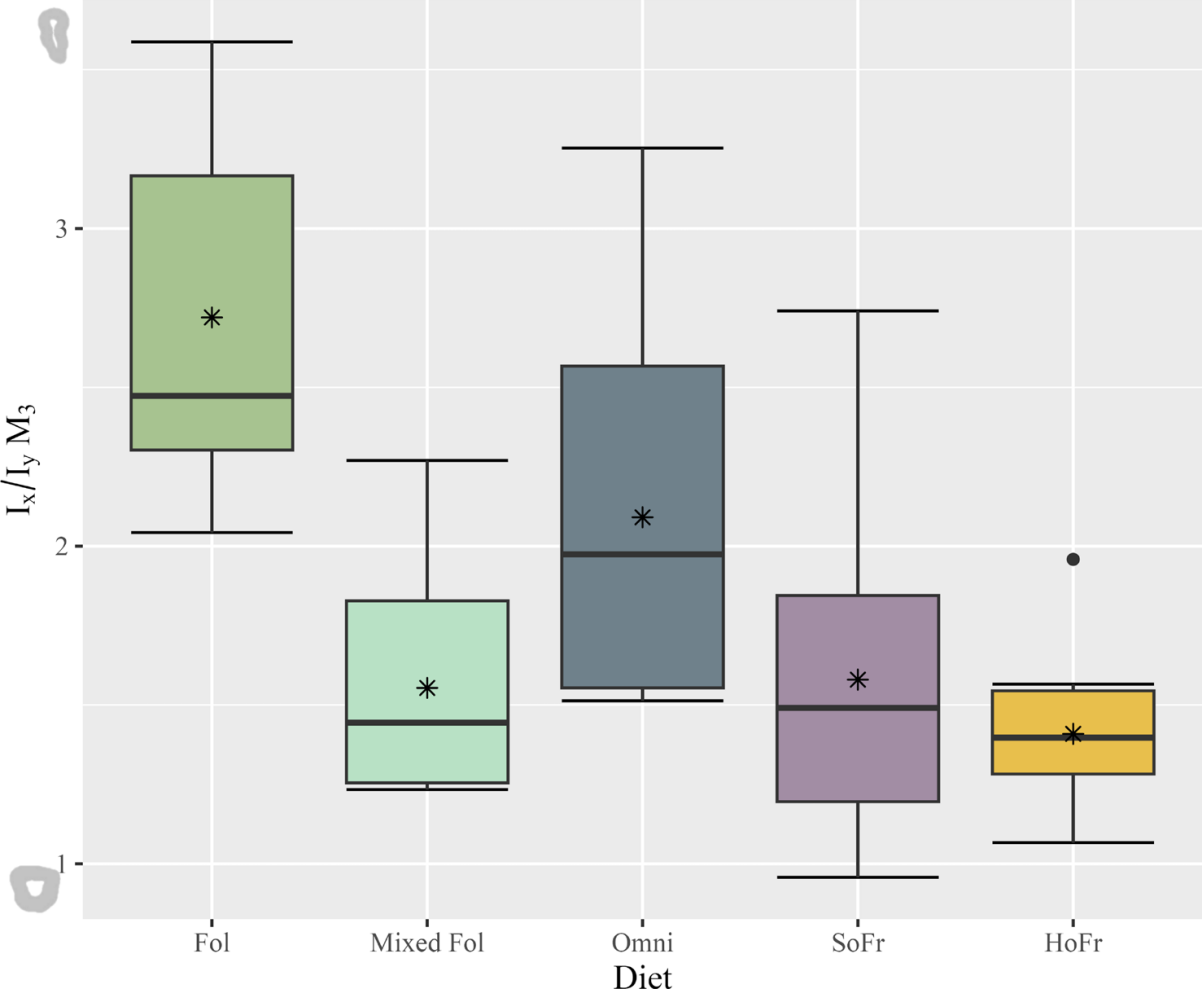
Boxplot of *I*_x_/*I*_y_ ratios for the M_3_ tooth position by dietary category. Thick horizontal bars within each box represent the median and asterisk symbols represent mean values for each diet. Single black dots represent outliers. Illustrations to the left of the Y‐axis represent the maximum and minimum *I*_x_/*I*_y_ ratio morphologies for M_3_.

The results for the analyses are presented in Tables 2, 3, 4 with additional details, including summary statistics and raw data used to calculate the ratios, contained in the Tables S1–S8, Figures S1–S3. Graphical output of results can be found in Figures 3, 4, 5, 6, 7, 8, 9. Ratios for *I*_max_/*I*_min_ did not differ substantially from the other results and hence those results are provided in the Supplement rather than the main text.

**TABLE 2:** Regression coefficients for dentin area on mandibular length.

Linear regression coefficients dentin area on mandibular length												
Y‐variable	Slope (95% CI)	SE	Slope *t* value (df)	*p*	Intercept (95% CI)	SE	Intercept *t* value (df)	*p*	*r* ^2^	Adjusted *r* ^2^	*F* value (df)	*p*
Pooled sample	0.99214 (0.9460617 to 1.038216)	0.02336	42.48 (186)	**< 0.001**	−2.82689 (−3.0434505 to −2.610339)	0.10977	−25.75 (186)	**< 0.001**	0.9066	0.906	1804 (1, 186)	**< 0.001**
Genus Average	1.00878 (0.9801798 to 1.037386)	0.01450	69.58 (186)	**< 0.001**	−2.89906 (−3.0335573 to −2.764568)	0.06817	−42.52 (186)	**< 0.001**	0.963	0.9628	4841 (1, 186)	**< 0.001**
M_1_	0.9745 (0.8960672 to 1.052840)	0.0392	2486 (61)	**< 0.001**	−2.7799 (−3.1485896 to −2.411128)	0.1844	−15.07 (61)	**< 0.001**	0.9102	0.9087	617.9 (1, 61)	**< 0.001**
M_2_	0.98067 (0.9107054 to 1.050636)	0.03501	28.01 (63)	**< 0.001**	−2.72095 (−3.0495403 to −2.392365)	0.16443	−16.55 (63)	**< 0.001**	0.9257	0.9245	784.5 (1, 63)	**< 0.001**
M_3_	1.02477 (0.9385598 to 1.110988)	0.04307	23.79 (58)	**< 0.001**	−2.99867 (−3.4038052 to −2.593545)	0.20239	−14.82 (58)	**< 0.001**	0.9071	0.9055	566.1 (1, 58)	**< 0.001**

*Note:* Pooled and individual tooth positions are provided. Significant values in bold (*p* < 0.05). CI = 95% confidence interval.

**TABLE 3:** Table of Tukey's HSD test results for pairwise comparisons of dentin area regression residuals for all dietary categories.

Pooled sample	Folivore	Mixed folivore	Omnivore	Soft object frugivore	Hard object frugivore
Folivore	1.000	—	—	—	—
Mixed folivore	0.94	1.000	—	—	—
Omnivore	0.22	**0.01**	1.000	—	—
Soft object frugivore	**< 0.001**	**< 0.001**	**0.03**	1.000	—
Hard object frugivore	0.82	0.99	**0.002**	**< 0.001**	1.000
M_1_					
Folivore	1.000	—	—	—	—
Mixed folivore	0.05	1.000	—	—	—
Omnivore	0.997	**0.004**	1.000	—	—
Soft object frugivore	0.994	**0.002**	0.999	—	—
Hard object frugivore	0.07	0.999	**0.006**	**0.002**	1.000
M_2_					
Folivore	1.000	—	—	—	—
Mixed folivore	0.93	1.000	—	—	—
Omnivore	0.99	0.59	1.000	—	—
Soft object frugivore	0.30	**0.02**	0.39	—	—
Hard object frugivore	0.48	0.89	0.11	**< 0.001**	1.000
M_3_					
Folivore	1.000	—	—	—	—
Mixed folivore	0.24	1.000	—	—	—
Omnivore	**0.03**	0.92	1.000	—	—
Soft object frugivore	**< 0.001**	0.07	0.27	—	—
Hard object frugivore	0.41	0.98	0.57	**0.005**	1.000

*Note:* Pooled and individual tooth positions are provided. Significant values in bold (*p* < 0.05).

**TABLE 4:** Wilcoxon rank sum results for pairwise comparisons of *I*_x_/*I*_y_ ratios for all dietary categories.

Wilcoxon rank sum pairwise comparisons *I*_x_/*I*_y_					
	Folivore	Mixed folivore	Omnivore	Soft object frugivore	Hard object frugivore
Folivore	1.000	—	—	—	—
Mixed folivore	0.05	1.000	—	—	—
Omnivore	0.35	**< 0.001**	1.000	—	—
Soft object Frugivore	0.23	0.35	**0.005**	1.000	—
Hard object Frugivore	**< 0.001**	**0.02**	**< 0.001**	**0.004**	1.000

*Note:* Significant values in bold (*p* < 0.05).

### 3.1 Dentin Area

The results for the PGLS demonstrate no phylogenetic signal in the residuals for dentin area regressed on mandibular length (*λ* = 0, *p* = 1 and *λ* = 1, *p* = 0.03). As no phylogenetic correction was warranted, the OLS models for each tooth position were used in subsequent analyses.

OLS linear regression results for dentin area on mandibular length are summarized in Table 2. There is a strong relationship between mandibular length and dentin area for all dietary categories (Figures 3, 4, 5) at each tooth position. Shapiro–Wilk tests of normality for dentin area regression residuals (pooled sample and individual tooth positions) were not significant (*W* = 0.9692–0.99247, *p* = 0.12–0.69) suggesting that the data follow a normal distribution. Levene's test for homogeneity of variance suggests equal variances across the sample (*F*(4) = 0.2301–1.7605, *p* = 0.14–0.92). Estimates for the Variance Inflation Factor (VIF) for the pooled sample return low values (< 2) for sex, tooth position, and diet indicating no collinearity between the independent variables (1.0–1.16).

Post hoc pairwise comparisons by diet are reported for all ANOVA models in Table 3. Factorial ANOVA results for the pooled sample residuals showed significant differences in group means sex (*F*(1) = 8.326, *p* = 0.004). Significant differences were also found for both tooth position (*F*(2) = 11.926, *p* < 0.001) and diet (*F*(4) = 16.333, *p* < 0.001). The interaction between variables, however, were not significant (*F*(12) = 0.0350, *p* > 0.05). The estimated effect sizes for significant variables were very small to moderate for sex *η* ^2^ = 0.03, tooth position *η* ^2^ = 0.08, and diet *η* ^2^ = 0.23. Post hoc tests for sex indicated significant mean differences (*p* = 0.004; estimated difference = −0.05). There were also group mean differences for tooth position between M_1_ and M_2_ (*p* < 0.001) and between M_2_ and M_3_ (*p* = 0.001). Soft object frugivores are significantly different from all other dietary groups (*p* = 0.02—*p* < 0.001). Hard object frugivores are significantly different from omnivores (*p* = 0.008) and soft object frugivores (*p* < 0.001). Omnivores additionally are significantly different from mixed folivores (*p* = 0.02).

For the M_1_ residuals, the factorial ANOVA results showed significant differences in group means diet (*F*(4) = 7.707, *p* < 0.001), but not sex (*F*(1) = 2.250, *p* > 0.05). The estimated effect size for diet was large *η* ^2^ = 0.33. Post hoc pairwise comparisons by diet show that soft object frugivores are significantly different from mixed folivores and hard object frugivores (*p* = 0.002). Omnivores are significantly different from mixed folivores (*p* = 0.004) and hard object frugivores (*p* = 0.006). All other groups are indistinguishable.

M_2_ factorial ANOVA results for the regression residuals also showed significant differences in group means diet (*F*(4) = 5.218, *p* < 0.001), but not sex (*F*(1) = 2.755, *p* > 0.05). The estimated effect size for diet at the M_2_ was large *η* ^2^ = 0.26. Pairwise comparisons again indicate that soft object frugivores are significantly different from mixed folivores and hard object frugivores (*p* = 0.02 and *p* < 0.001, respectively). No other significant differences were returned for the M_2_.

Results for the factorial ANOVA on the regression residuals of the M_3_ showed significant differences in group means for both diet (*F*(4) = 7.112, *p* < 0.001) and sex (*F*(1) = 4.346, *p* = 0.04). The estimated effect size for diet, as with the other two teeth, was large *η* ^2^ = 0.34, but small for sex *η* ^2^ = 0.05. The post hoc test for sex indicates differences between male and female group means but statistical significance is lost (*p* = 0.05, estimated difference = −0.06). In the pairwise comparisons, soft object frugivores are significantly different from folivores (*p* < 0.001) and hard object frugivores (*p* = 0.005). Omnivores are significantly different from only folivores (*p* = 0.005). No other differences were recovered.

### 3.2 *I*_x_/*I*_y_ Ratios

*I*_x_/*I*_y_ ratios are plotted for individual tooth positions and grouped by diet (Figures 7, 8, 9). Data demonstrate unequal variances and do not follow a normal distribution (*W* = 0.89986, *p* < 0.001; *F*(4) = 5.001, *p* < 0.001) and therefore non‐parametric test results are reported. The Kruskal‐Wallis rank sum test returned no significant differences by sex (*X* ^2^(1) = 0.78233, *p* > 0.05). Results for tooth position are significant (*X* ^2^(2) = 22.191, *p* < 0.001); however, and pairwise Wilcoxon rank sum tests show differences between M_3_ and the two other tooth positions (*p* < 0.001) but not between M_1_ and M_2_ (*p* > 0.05). Comparisons among diet groups indicate significant differences (*X* ^2^ (4) = 42.814, *p* < 0.001). Hard object frugivores differ significantly from all dietary categories having a lower mean value of *I*_x_/*I*_y_ (*p* = 0.02—*p* < 0.001), whereas omnivores additionally differ from soft object frugivores (*p* = 0.005) and mixed folivores having a higher mean value of *I*_x_/*I*_y_ (*p* < 0.001) (Table 4).

For M_1_, *Erythrocebus* has the only *I*_x_/*I*_y_ mean value that falls above 2.0, with the greatest dimensions in the MD plane. All other genus ratios are between 1.0 and 2.0. At M_2_, *I*_x_/*I*_y_ ratios are markedly different: the mean for *Cercocebus* is below 1.0 (0.89), with increased BL distribution; mean values for the rest of the genera fall between 1.0 and 2.0, with *Erythrocebus* maintaining the highest mean value overall and a MD distribution of cross‐sectional dentin (1.84). The M_3_ ratios demonstrate no mean values below 1.0, though *Pan* returns the lowest mean and, across its dentition, a consistent BL dentin distribution (1.02–1.26). *Theropithecus* (2.42), *Colobus* (2.84), and *Papio* (2.88) have the highest mean values and MD‐oriented dentin cross‐sections at M_3_ (Table S3).

## 4 Discussion

The decision to assign the sample to broad dietary categories inherently complicates the interpretation of the influence of diet on the root morphology and biomechanics across taxa as these categories are necessarily simplistic. An alternative strategy to categorizing diet is to use food material properties, however, this presents several issues including which measure to use (for details see, Berthaume 2016). Hardness, toughness, and Young's modulus are often reported, though these measures alone can create a dizzying array of possible combinations for a given species' diet (e.g., Thiery et al. 2017). Methodological differences, such as the choice of the material tester, can further complicate analyses with different testers leading to incongruent results across studies (Lucas et al. 2012; Berthaume 2016). Lastly, as discussed above, most primates eat a range of food items and a thorough understanding of the relationship between food material properties and observed consumption patterns has not been fully realized (Coiner‐Collier et al. 2016).

### 4.1 Dentin Area

A lack of phylogenetic signal in the residuals for dentin area regressed on mandibular length is not unusual in data where the traits themselves might have high phylogenetic signal (*λ*) if measured individually (Revell 2010; Symonds and Blomberg 2014; Pearse et al. 2025). However, the value of λ in individual traits should not be used as justification for phylogenetic correction as they may lead to the selection of improper model and poor performance (Revell 2010). It is possible that scaling dentin area by mandibular length for each tooth position accounted for the detectable proportion of phylogenetic signal in the data. It may also be the case that the limited sample size for each species is influencing the ability of the PGLS to capture the true value of λ as has been demonstrated under simulation (e.g., Münkemüller et al. 2012), or that a more complex model of evolution underlie these data (Mazel et al. 2016). While the results presented for the current sample can be interpreted without phylogenetic correction, future work on dentin area should include a more robust sample to understand the effects, if any, of taxonomy on the morphological patterns.

The results for dentin area regressions are related to both mechanical challenge and diet. Groups that consume more resistant items, either through repetitive loading (e.g., leaves, stems, and bark) or forceful biting (e.g., seeds), had larger relative dentin area for a given mandibular length than did generalists and soft‐fruit specialists, who had smaller dentin area relative to mandibular length. This result accords with previous studies that found differences between root size and mechanical effort (e.g., Spencer 2003). A similar pattern is observed in these data for *Pongo* and *Cercocebus*, two taxa that have relatively larger dentin area values than any of the taxa that process pliant fruits. Residual values varied significantly between some dietary groups but not all. In particular, the soft object frugivore results (Table 3) may reflect differences in the pulp cavity size/shape relative to high‐wear genera (i.e., reduced overall pulp cavity size). This interpretation is consistent with the results from Selig et al. (2021), who found that primates engaged in processing high amounts of high wear items had an enlarged pulp cavity volume relative to overall tooth volume. However, as the relative pulp cavity size in dedicated folivores has not yet been examined, it is difficult to assess the existence of such a relationship. Furthermore, if the pulp cavity is driving the differences in this sample, it would be expected that genera with higher rates of folivory would be significantly different from the generalist omnivores as well as the soft object frugivores, yet this was only observed in these data when the M_3_ was analyzed individually. That dentin area was also useful in distinguishing diets with more complex strategies, like omnivory and, to a lesser extent, mixed folivory, is particularly interesting as it suggests that dentin cross‐sectional area is not simply a reflection the need for load dissipation, as per Kovacs (1979), but may be indicative of more subtle ecological adaptations.

Dentin area was the only variable in this sample to return a statistically significant result when sex was considered, with females having marginally higher dentin areas than expected given mandibular length. This pattern was not consistent across molars and, in fact, only present in the M_3_ when analyzed using individual tooth position. The literature is inconsistent when signals of sexual dimorphism are sought from the roots. Some researchers, like Abbott (1984), found degrees of sexual dimorphism for the mesiodistal neck diameters in the molars of *Gorilla*, *Pongo*, and to a lesser extent *Pan* (M_3_) and *Homo sapiens* (M_1_ and M_2_); alternatively, Spencer (2003) found surface area dimorphism in the *Sapajus* sample, but not for *Pithecia* or *Chiropotes*. Plavcan and Van Schaik (1992) found strong sexual dimorphism in the canine crown, measured as linear dimensions and a general canine dimorphism index (GCDI) derived from a principal component analysis of those linear dimensions, has been reported for anthropoid primates and associated with male–male competition and body mass. Canine dimorphism (i.e., GCDI and linear dimensions) was strongly correlated with higher body mass dimorphism in the sample of anthropoids excepting the cercopithecines (Plavcan and Van Schaik 1992). Other studies indicate limited dimorphism; for example, Schwartz and Dean (2005) found tooth mass was significantly different between male and female modern human molars and that males had significantly more dentin. Male molars were also shown to have more dentin than their female counterparts in a recent analysis by Sorenti et al. (2019), who showed that males had larger mean values for crown dentin measured from 2D cross‐sections obtained from CT scans. Taking these studies into account along with the result from this study that found a negligible but statistically significant difference between male and female relative dentin areas, there is inconsistency along the toothrow in degrees of dental dimorphism. Because this study had a limited number of sex‐matched samples, as well as small sample sizes, the amount of male to female sexual dimorphism in size of dentin area for molar crowns is not reliably assessed here from cervical root cross‐section. In addition, because this study did not find any statistically significant signal in the ratio‐based data, it may be that potential differences in sex‐based size signals are obscured by the ratio itself.

Finally, this study did not explore the relationship between the dentin area measured here, which excludes the pulp chamber, and the dimensions of the tooth crown itself. A functional relationship between crown and root size was first hypothesized by Kovacs (1979) and subsequent work, as summarized in the introduction, has demonstrated that the link between the crown and external root dimensions varies in relation to loading (e.g., Ward and Molnar 1980; Spencer 2003; Kupczik 2003; Deutsch et al. 2022). Given the close developmental and physical association between the cervix and crown, their dimensions are likely highly interrelated. Future studies that examine dentin area would benefit from directly testing this relationship to better understand how the crown and root covary under different loading conditions.

### 4.2 *I*_x_/*I*_y_ Ratios

Results for the *I*_x_/*I*_y_ ratios are informative of dietary differences in dentin distribution. Omnivore taxa have the most dentin allocated to the mesial‐distal aspect of the molars, followed by soft frugivores, with the exception of the M_3_, where folivores have an increased proportion of dentin across the mesial‐distal aspect than do the sample of omnivores. Variation for the M_3_ *I*_x_/*I*_y_ ratios were significantly higher than at the other tooth positions. However, the only group that demonstrated interspecific variation that was statistically different from the overall M_3_ sample were the hard object frugivores, which were significantly less variable than the rest of the dietary categories (Table S5).

The distribution of dentin in omnivores and soft object frugivores may reflect a generalized cervical root pattern capable of processing a variety of low‐specialization food items. One alternative explanation for the variation in the dentin distribution for these two groups may be related to an increased reliance on alternative food processing strategies such as the use of anterior teeth for pre‐oral processing (e.g., Hylander 1975; Ungar 1996; Kupczik 2003; Wieczkowski 2009). *Papio* and *Pan*, for example, have relative larger incisors and engage in a large amount of incisal preparation of fruit items before processing with the postcanine teeth (Hylander 1975). This may reduce the loads incurred by molar teeth leading to less need for additional dentin allocation to either the mesial‐distal or buccal‐lingual extremes. *Hylobates*, in contrast, tends to consume foods that are soft and small, which do not require incisor engagement (Ungar 1996). This behavior could impose an increased reliance on the molars relative to *Pan*. As the omnivore category includes taxa that consume a large quantity of insects, gums, and a variety of plant matter, it is unlikely that this morphology is linked to a particular food item common to all three taxa (Gautier‐Hion 1983; Enstam and Isbell 2007; Johnson et al. 2012); instead, it is interpreted here as a generalized form capable supporting of a variety of food items with varying mechanical challenge (Kay and Hiiemae 1974; Wall et al. 2006). The soft object frugivores span a large range driven largely by *Hylobates*, which demonstrates increased mesial‐distal dentin allocation relative to *Pan*. Interestingly, *Hylobates*, like *Pan*, has been observed to include a proportion of insects in its diet (Bogart and Pruetz 2011; Bartlett et al. 2016). The differences in the morphology of these two taxa may indicate differences in a variety of factors including, the proportion of fruits or insects consumed, the degree of mastication performed, the material properties of the food items themselves, and/or consumed objects that are yet unidentified. Although the PGLS did not return significant phylogenetic signal for these data, there remains the possibility that some of the morphological differences are the result of taxonomic differences between the larger‐bodied hominid and the smaller‐bodied hylobatid that are too subtle for detection using this method.

In contrast, more buccal‐lingual orientation of the dentin is found in folivores (dedicated and mixed) and hard object frugivores; this may be correlated with the mechanically resistant foods consumed by *Colobus*, *Gorilla*, and *Pongo* (Thiery et al. 2017). If this is the case, then reallocation of dentin to the buccal‐lingual aspect may serve to buttress the tooth against heavy loading, repetitive chewing, or multi‐directional movements. These interpretations are supported by previous studies that found root size differs along the tooth row for species that engaged in incisal, canine, or premolar use relative to those that rely on more dedicated molar processing (e.g., Kupczik 2003; Le Cabec et al. 2013; Spencer 2003). The present results suggest that the cross‐sectional dentin distribution, measured as the ratio of *I*_x_/*I*_y_, may be used to parse out feeding strategies and specifically to separate generalist omnivores and more specialized hard object frugivores in samples that demonstrate mixed or unclear dietary signals at the dental crown, or in the case of fossil primates with unknown feeding strategies.

## 5 Conclusion

This work has shown that the cervical root cross‐section is a region that contains information regarding both the biomechanical function of a tooth as well as conveying some aspects of dietary specialization in extant catarrhines. Whereas this study included a large variety of catarrhine primates, sample sizes for genera were small, and as such, any application of these methods to taxa or fossils outside of the two families included herein should be undertaken with some caution. This cross‐section provided limited information regarding molar sexual dimorphism, highlighting inconsistent patterns along the toothrow, and more work is needed to determine if this region could be useful as an additional line of evidence regarding in the assessment of sexually dimorphic taxa. Future research should focus on the expansion of sample sizes within each taxon, as well as the incorporation of strepsirrhines and platyrrhines to look at the range of variation across the order Primates and to understand the influence of ontogeny on the observed patterns. Increased sampled would provide a more robust estimate of the influence of phylogeny on dentin distribution across the cervical root cross‐section and facilitate a broader understanding of the impact of a variety of dietary types on this region. Furthermore, better sampling of different dietary ecologies would permit better resolution for reconstructing the paleoecology of fossil primates.

## Author Contributions

**Zana R. Sims:** conceptualization, methodology, investigation, formal analysis, funding acquisition, writing – original draft.

## Conflicts of Interest

The author declares no conflicts of interest.

## Supporting information


**Data S1:** Supporting Information.

**Data S2:** Supporting Information.

## Data Availability

The data that support the findings of this study are available in the Supporting Information for this article. Cross‐sectional slices are provided as an additional data file. All original scans were collected using specimens on loan from the Harvard Museum of Comparative Zoology and micro‐CT scanned at the Harvard Center for Nanoscale Systems with support from a grant from the Leakey Foundation.
