# Supplementary material for: Molar Cervical Root Cross‐Sectional Morphology and Diet in Extant Catarrhines
Source: Am J Biol Anthropol. 2025 Nov 18;188(3):e70164. doi: 10.1002/ajpa.70164 (PMC12625805; doi:10.1002/ajpa.70164)
Supplement: Supplementary file 1 — Data S1: Supporting Information. [file AJPA-188-e70164-s002.pdf]

**Supplemental Information**

Table S1. µ-CT Sample Listed by Institution then by Taxa

| Institution | No. | Species | MorphoSource DOI/ARK | Sex | Resolution (mm) |
|---|---|---|---|---|---|
| AMNH | 52640 | *Cercocebus agilis* | https://n2t.net/ark:/87602/m4/M13610 | F | 0.06 |
| AMNH | 52645 | *C. agilis* | https://n2t.net/ark:/87602/m4/M13620 | M | 0.08 |
| AMNH | 52635 | *C. agilis* | https://n2t.net/ark:/87602/m4/M13608 | F | 0.06 |
| AMNH | 52641 | *C. agilis* | https://n2t.net/ark:/87602/m4/M13615 | M | 0.07 |
| AMNH | 52634 | *C. agilis* | https://n2t.net/ark:/87602/m4/M13575 | M | 0.06 |
| AMNH | 52215 | *Colobus guereza occidentalis* | https://n2t.net/ark:/87602/m4/M12514 | F | 0.06 |
| AMNH | 52238 | *C. guereza occidentalis* | https://n2t.net/ark:/87602/m4/M13437 | F | 0.07 |
| AMNH | 52209 | *C. guereza occidentalis* | https://n2t.net/ark:/87602/m4/M10642 | M | 0.07 |
| AMNH | 52210 | *C. guereza occidentalis* | https://n2t.net/ark:/87602/m4/M12498 | M | 0.07 |
| AMNH | 52237 | *C. guereza occidentalis* | https://n2t.net/ark:/87602/m4/M13435 | M | 0.07 |
| AMNH | 34714 | *Erythrocebus patas* | https://n2t.net/ark:/87602/m4/M16054 | M | 0.06 |
| AMNH | A99-9687 | *Gorilla gorilla* | https://n2t.net/ark:/87602/m4/M22240 | F | 0.08 |
| AMNH | L.223 | *G. gorilla* | https://n2t.net/ark:/87602/m4/M22245 | M | 0.10 |
| AMNH | 167338 | *G. gorilla gorilla* | https://n2t.net/ark:/87602/m4/M21966 | M | 0.13 |
| AMNH | 167342 | *Pan troglodytes* | https://n2t.net/ark:/87602/m4/M21967 | M | 0.09 |
| AMNH | 102724 | *Symphalangus syndactylus* | https://n2t.net/ark:/87602/m4/M26157 | M | 0.06 |
| AMNH | 19549 | *Theropithecus gelada* | https://n2t.net/ark:/87602/m4/M17896 | M | 0.08 |
| AMNH | 90309 | *T. gelada* | https://n2t.net/ark:/87602/m4/M18337 | M† | 0.09 |
| AMNH | 238034 | *T. gelada* | https://n2t.net/ark:/87602/m4/M20353 | F | 0.12 |
| MCZ | 47016 | *Erythrocebus patas pyrrhonotus* | https://n2t.net/ark:/c7602/m2/m2924 https://doi.org/10.17602/M2/M2924 | M | 0.08 |
| MCZ | 47015 | *E. patas pyrrhonotus* | https://n2t.net/ark:/c7602/m2/m2923 https://doi.org/10.17602/M2/M2923 | M | 0.08 |
| MCZ | 37280 | *E. patas* | https://n2t.net/ark:/c7602/m2/m2922 https://doi.org/10.17602/M2/M2922 | M† | 0.08 |
| MCZ | 14750 | *Gorilla gorilla gorilla* | https://n2t.net/ark:/c7602/m2/m2940 https://doi.org/10.17602/M2/M2940 | F | 0.09 |
| MCZ | 37264 | *G. gorilla gorilla* | https://n2t.net/ark:/c7602/m2/m2949 https://doi.org/10.17602/M2/M2949 | F | 0.13 |
| MCZ | 20038 | *G. gorilla gorilla* | This project | M | 0.12 |
| MCZ | 20039 | *G. gorilla gorilla* | This project | M | 0.12 |

| | | | | | |
|---|---|---|---|---|---|
| MCZ | 23160 | *G. gorilla gorilla* | This project | M | 0.12 |
| MCZ | 57482 | *G. gorilla gorilla* | This project | M | 0.12 |
| MCZ | 41414 | *Hylobates lar lar* | https://n2t.net/ark:/c7602/m2/m2963 https://doi.org/10.17602/M2/M2963 | F | 0.07 |
| MCZ | 41421 | *H. lar lar* | https://n2t.net/ark:/c7602/m2/m2969 https://doi.org/10.17602/M2/M2969 | F | 0.07 |
| MCZ | 41460 | *H. lar lar* | https://n2t.net/ark:/c7602/m2/m3019 https://doi.org/10.17602/M2/M3019 | F | 0.07 |
| MCZ | 41463 | *H. lar lar* | https://n2t.net/ark:/c7602/m2/m3021 https://doi.org/10.17602/M2/M3021 | F | 0.07 |
| MCZ | 41493 | *H. lar lar* | https://n2t.net/ark:/c7602/m2/m3025 https://doi.org/10.17602/M2/M3025 | F | 0.07 |
| MCZ | 12742 | *H. lar moloch* | This project | M | 0.06 |
| MCZ | 37382 | *H. lar mulleri* | This project | M | 0.05 |
| MCZ | 37385 | *H. lar mulleri* | This project | M | 0.05 |
| MCZ | 41428 | *H. lar lar* | This project | M | 0.06 |
| MCZ | 34264 | *Miopithecus talapoin* | https://n2t.net/ark:/c7602/m2/m5092 https://doi.org/10.17602/M2/M5092 | F | 0.05 |
| MCZ | 23197 | *M. talapoin* | https://n2t.net/ark:/c7602/m2/m5093 https://doi.org/10.17602/M2/M5093 | M | 0.05 |
| MCZ | 23196 | *M. talapoin* | https://n2t.net/ark:/c7602/m2/m5094 https://doi.org/10.17602/M2/M5094 | M | 0.05 |
| MCZ | 19976 | *M. talapoin* | https://n2t.net/ark:/c7602/m2/m5095 https://doi.org/10.17602/M2/M5095 | M | 0.05 |
| MCZ | 37278 | *M. talapoin* | https://n2t.net/ark:/c7602/m2/m5086 https://doi.org/10.17602/M2/M5086 | F† | 0.05 |
| MCZ | BOM-9493 | *Pan troglodytes* | https://n2t.net/ark:/c7602/m2/m4392 https://doi.org/10.17602/M2/M4392 | F | 0.11 |
| MCZ | 17702 | *P. troglodytes* | https://n2t.net/ark:/c7602/m2/m4396 https://doi.org/10.17602/M2/M4396 | F | 0.10 |
| MCZ | 23167 | *P. troglodytes* | https://n2t.net/ark:/c7602/m2/m4390 https://doi.org/10.17602/M2/M4390 | F | 0.11 |
| MCZ | 15312 | *P. troglodytes troglodytes* | https://n2t.net/ark:/c7602/m2/m4391 https://doi.org/10.17602/M2/M4391 | F | 0.11 |
| MCZ | 19187 | *P. troglodytes* | This project | M | 0.09 |
| MCZ | 20041 | *P. troglodytes* | This project | M | 0.09 |
| MCZ | 23163 | *P. troglodytes* | This project | M | 0.09 |
| MCZ | 23164 | *P. troglodytes* | This project | M | 0.09 |
| MCZ | 17342 | *Papio doguera neumanni* | https://n2t.net/ark:/c7602/m2/m4886 https://doi.org/10.17602/M2/M4886 | M | 0.08 |
| MCZ | 21161 | *P. doguera furax* | https://n2t.net/ark:/c7602/m2/m4880 https://doi.org/10.17602/M2/M4880 | M | 0.08 |
| MCZ | 29786 | *P. doguera neumanni* | https://n2t.net/ark:/c7602/m2/m4878 https://doi.org/10.17602/M2/M4878 | M | 0.08 |
| MCZ | 8304 | *P. doguera neumanni* | https://n2t.net/ark:/c7602/m2/m4888 https://doi.org/10.17602/M2/M4888 | M | 0.09 |
| MCZ | 21160 | *P. doguera furax* | https://n2t.net/ark:/c7602/m2/m4882 https://doi.org/10.17602/M2/M4882 | M | 0.08 |
| MCZ | 23084 | *P. doguera* | This project | F | 0.07 |
| MCZ | 37518 | *Pongo pygmaeus* | https://n2t.net/ark:/c7602/m2/m4614 https://doi.org/10.17602/M2/M4614 | F | 0.08 |

| | | | | | |
|---|---|---|---|---|---|
| MCZ | 37519 | *P. pygmaeus* | https://n2t.net/ark:/c7602/m2/m4616<br>https://doi.org/10.17602/M2/M4616 | F | 0.08 |
| MCZ | 50958 | *P. pygmaeus* | https://n2t.net/ark:/c7602/m2/m4612<br>https://doi.org/10.17602/M2/M4612 | F | 0.11 |
| MCZ | 37362 | *P. pygmaeus* | This project | M | 0.11 |
| MCZ | 37363 | *P. pygmaeus* | This project | F | 0.11 |
| MCZ | 37365 | *P. pygmaeus* | This project | F | 0.10 |
| MCZ | 37516 | *P. pygmaeus* | This project | M | 0.10 |
| MCZ | 37517 | *P. pygmaeus* | This project | M | 0.10 |
| MCZ | 50960 | *P. pygmaeus* | This project | M | 0.11 |
| MCZ | 36032 | *Symphalangus syndactylus* | https://n2t.net/ark:/c7602/m2/m4443<br>https://doi.org/10.17602/M2/M4443 | F | 0.07 |
| MCZ | 36031 | *S. syndactylus* | https://n2t.net/ark:/c7602/m2/m4444<br>https://doi.org/10.17602/M2/M4444 | M | 0.08 |
| MCZ | 23986 | *Theropithecus gelada* | https://n2t.net/ark:/c7602/m2/m4440<br>https://doi.org/10.17602/M2/M4440 | M | 0.08 |

† Sex estimated through species-matched comparisons of canine and facial size.

## Summary Statistics

### Table S2. Statistics by Diet

| | n | mean | sd | median | min | max | range | se |
|---|---|---|---|---|---|---|---|---|
| **Folivore** | | | | | | | | |
| DA | 22 | 36.68 | 17.49 | 28.71 | 19.11 | 81.16 | 62.05 | 3.73 |
| $I_{max}$ | 22 | 254.82 | 298.82 | 139.61 | 43.40 | 1249.24 | 1205.84 | 63.71 |
| $I_{min}$ | 22 | 139.08 | 140.22 | 67.32 | 37.14 | 444.00 | 406.86 | 29.90 |
| JL(mm) | 22 | 99.66 | 18.84 | 87.62 | 82.89 | 128.98 | 46.09 | 4.02 |
| Ix | 22 | 248.78 | 292.61 | 135.59 | 42.06 | 1225.55 | 1183.49 | 62.38 |
| Iy | 22 | 145.13 | 146.71 | 67.60 | 38.78 | 456.99 | 418.21 | 31.28 |
| $I_x/I_y$ | 22 | 1.78 | 0.76 | 1.53 | 0.95 | 3.59 | 2.64 | 0.16 |
| $I_{max}/I_{min}$ | 22 | 1.87 | 0.77 | 1.58 | 1.16 | 3.80 | 2.64 | 0.16 |
| $M_1$ $I_x/I_y$ | 7 | 1.34 | 0.33 | 1.45 | 0.95 | 1.73 | 0.78 | 0.12 |
| $M_2$ $I_x/I_y$ | 8 | 1.36 | 0.16 | 1.35 | 1.08 | 1.58 | 0.49 | 0.06 |
| $M_3$ $I_x/I_y$ | 7 | 2.72 | 0.62 | 2.47 | 2.04 | 3.59 | 1.54 | 0.23 |
| $M_1$ $I_{max}/I_{min}$ | 7 | 1.45 | 0.24 | 1.47 | 1.16 | 1.74 | 0.58 | 0.09 |
| $M_2$ $I_{max}/I_{min}$ | 8 | 1.40 | 0.14 | 1.38 | 1.18 | 1.58 | 0.41 | 0.05 |
| $M_3$ $I_{max}/I_{min}$ | 7 | 2.83 | 0.66 | 2.48 | 2.20 | 3.80 | 1.60 | 0.25 |
| **Mixed Folivore** | | | | | | | | |
| | n | mean | sd | median | min | max | range | se |
| DA | 33 | 101.46 | 42.47 | 113.79 | 19.07 | 156.81 | 137.74 | 7.39 |
| $I_{max}$ | 33 | 1788.39 | 1043.92 | 1927.70 | 48.00 | 3864.09 | 3816.09 | 181.72 |
| $I_{min}$ | 33 | 1325.31 | 818.54 | 1455.64 | 36.48 | 2997.21 | 2960.72 | 142.49 |

| | | | | | | | | |
|---|---|---|---|---|---|---|---|---|
| JL(mm) | 33 | 162.65 | 45.50 | 188.45 | 75.40 | 203.38 | 127.98 | 7.92 |
| Ix | 33 | 1726.83 | 1032.01 | 1881.77 | 47.88 | 3813.11 | 3765.23 | 179.65 |
| Iy | 33 | 1386.87 | 844.13 | 1493.36 | 36.60 | 3048.19 | 3011.59 | 146.95 |
| $I_x/I_y$ | 33 | 1.39 | 0.39 | 1.30 | 0.74 | 2.43 | 1.69 | 0.07 |
| $I_{max}/I_{min}$ | 33 | 1.47 | 0.35 | 1.32 | 1.06 | 2.44 | 1.38 | 0.06 |
| $M_1$ $I_x/I_y$ | 12 | 1.36 | 0.43 | 1.27 | 0.83 | 2.43 | 1.59 | 0.12 |
| $M_2$ $I_x/I_y$ | 12 | 1.29 | 0.35 | 1.27 | 0.74 | 2.00 | 1.26 | 0.10 |
| $M_3$ $I_x/I_y$ | 9 | 1.55 | 0.37 | 1.44 | 1.23 | 2.27 | 1.04 | 0.12 |
| $M_1$ $I_{max}/I_{min}$ | 12 | 1.41 | 0.39 | 1.28 | 1.06 | 2.44 | 1.38 | 0.11 |
| $M_2$ $I_{max}/I_{min}$ | 12 | 1.42 | 0.26 | 1.33 | 1.16 | 2.09 | 0.93 | 0.08 |
| $M_3$ $I_{max}/I_{min}$ | 9 | 1.63 | 0.38 | 1.63 | 1.26 | 2.29 | 1.03 | 0.13 |
| Omnivore | | | | | | | | |
| | n | mean | sd | median | min | max | range | se |
| DA | 41 | 35.71 | 31.49 | 23.54 | 4.42 | 106.42 | 102.00 | 4.92 |
| $I_{max}$ | 41 | 371.86 | 559.35 | 92.88 | 2.82 | 2174.37 | 2171.55 | 87.36 |
| $I_{min}$ | 41 | 195.34 | 269.88 | 42.94 | 1.70 | 948.46 | 946.76 | 42.15 |
| JL(mm) | 41 | 95.79 | 43.24 | 103.51 | 40.25 | 153.29 | 113.04 | 6.75 |
| Ix | 41 | 367.91 | 553.54 | 91.28 | 2.77 | 2141.61 | 2138.84 | 86.45 |
| Iy | 41 | 199.29 | 276.68 | 42.95 | 1.75 | 998.12 | 996.38 | 43.21 |
| $I_x/I_y$ | 41 | 1.83 | 0.52 | 1.68 | 1.09 | 3.25 | 2.16 | 0.08 |
| $I_{max}/I_{min}$ | 41 | 1.88 | 0.53 | 1.71 | 1.19 | 3.34 | 2.16 | 0.08 |
| $M_1$ $I_x/I_y$ | 13 | 1.93 | 0.46 | 1.94 | 1.30 | 2.70 | 1.39 | 0.13 |
| $M_2$ $I_x/I_y$ | 15 | 1.51 | 0.31 | 1.49 | 1.09 | 2.11 | 1.01 | 0.08 |
| $M_3$ $I_x/I_y$ | 13 | 2.09 | 0.60 | 1.97 | 1.51 | 3.25 | 1.74 | 0.17 |
| $M_1$ $I_{max}/I_{min}$ | 13 | 1.99 | 0.47 | 2.01 | 1.33 | 2.70 | 1.37 | 0.13 |
| $M_2$ $I_{max}/I_{min}$ | 15 | 1.56 | 0.29 | 1.54 | 1.19 | 2.11 | 0.92 | 0.07 |
| $M_3$ $I_{max}/I_{min}$ | 13 | 2.15 | 0.62 | 2.00 | 1.58 | 3.34 | 1.76 | 0.17 |
| Soft Object Frugivore | | | | | | | | |
| | n | mean | sd | median | min | max | range | se |
| DA | 51 | 30.09 | 18.40 | 18.93 | 9.11 | 60.06 | 50.95 | 2.58 |
| $I_{max}$ | 51 | 165.82 | 157.97 | 50.82 | 15.04 | 504.75 | 489.71 | 22.12 |
| $I_{min}$ | 51 | 130.85 | 136.78 | 28.46 | 8.72 | 441.08 | 432.35 | 19.15 |
| JL(mm) | 51 | 99.68 | 32.81 | 72.70 | 65.74 | 146.68 | 80.94 | 4.59 |
| Ix | 51 | 159.07 | 152.53 | 50.66 | 13.77 | 493.88 | 480.12 | 21.36 |
| Iy | 51 | 137.60 | 143.14 | 29.14 | 8.91 | 446.75 | 437.84 | 20.04 |
| $I_x/I_y$ | 51 | 1.50 | 0.47 | 1.39 | 0.71 | 2.74 | 2.03 | 0.07 |
| $I_{max}/I_{min}$ | 51 | 1.59 | 0.43 | 1.47 | 1.06 | 2.81 | 1.75 | 0.06 |
| $M_1$ $I_x/I_y$ | 17 | 1.42 | 0.45 | 1.37 | 0.71 | 2.15 | 1.43 | 0.11 |
| $M_2$ $I_x/I_y$ | 16 | 1.48 | 0.47 | 1.39 | 1.00 | 2.68 | 1.68 | 0.12 |
| $M_3$ $I_x/I_y$ | 18 | 1.58 | 0.48 | 1.49 | 0.96 | 2.74 | 1.78 | 0.11 |

| | | | | | | | | |
|---|---|---|---|---|---|---|---|---|
| $M_1$ $I_{max}/I_{min}$ | 17 | 1.58 | 0.36 | 1.55 | 1.12 | 2.23 | 1.12 | 0.09 |
| $M_2$ $I_{max}/I_{min}$ | 16 | 1.52 | 0.46 | 1.42 | 1.06 | 2.71 | 1.65 | 0.11 |
| $M_3$ $I_{max}/I_{min}$ | 18 | 1.66 | 0.49 | 1.53 | 1.09 | 2.81 | 1.72 | 0.11 |

| Hard Object Frugivore | | | | | | | | |
|---|---|---|---|---|---|---|---|---|
| | n | mean | sd | median | min | max | range | se |
| DA | 41 | 66.19 | 25.33 | 74.23 | 24.98 | 109.18 | 84.19 | 3.96 |
| $I_{max}$ | 41 | 688.91 | 465.01 | 709.26 | 77.46 | 1725.80 | 1648.35 | 72.62 |
| $I_{min}$ | 41 | 548.62 | 398.45 | 536.28 | 67.77 | 1511.32 | 1443.55 | 62.23 |
| JL(mm) | 41 | 132.89 | 37.50 | 147.08 | 73.04 | 177.85 | 104.81 | 5.86 |
| Ix | 41 | 667.59 | 460.51 | 696.27 | 70.84 | 1697.57 | 1626.73 | 71.92 |
| Iy | 41 | 569.94 | 404.86 | 562.39 | 74.39 | 1539.55 | 1465.16 | 63.23 |
| $I_x/I_y$ | 41 | 1.19 | 0.24 | 1.16 | 0.72 | 1.96 | 1.24 | 0.04 |
| $I_{max}/I_{min}$ | 41 | 1.32 | 0.19 | 1.27 | 1.10 | 2.07 | 0.96 | 0.03 |
| $M_1$ $I_x/I_y$ | 14 | 1.12 | 0.17 | 1.12 | 0.94 | 1.53 | 0.59 | 0.04 |
| $M_2$ $I_x/I_y$ | 14 | 1.06 | 0.18 | 1.10 | 0.72 | 1.27 | 0.55 | 0.05 |
| $M_3$ $I_x/I_y$ | 13 | 1.41 | 0.23 | 1.40 | 1.07 | 1.96 | 0.89 | 0.06 |
| $M_1$ $I_{max}/I_{min}$ | 14 | 1.27 | 0.12 | 1.26 | 1.12 | 1.60 | 0.48 | 0.03 |
| $M_2$ $I_{max}/I_{min}$ | 14 | 1.23 | 0.11 | 1.22 | 1.10 | 1.51 | 0.41 | 0.03 |
| $M_3$ $I_{max}/I_{min}$ | 13 | 1.48 | 0.24 | 1.54 | 1.14 | 2.07 | 0.93 | 0.07 |

Table S3. Statistics by Genus

| *Cercocebus* | | | | | | | | |
|---|---|---|---|---|---|---|---|---|
| | n | mean | sd | median | min | max | range | se |
| DA | 14 | 34.43 | 4.63 | 33.67 | 24.98 | 41.99 | 17.01 | 1.24 |
| $I_{max}$ | 14 | 160.84 | 37.24 | 157.82 | 77.46 | 208.82 | 131.37 | 9.95 |
| $I_{min}$ | 14 | 116.76 | 33.50 | 109.00 | 67.77 | 172.55 | 104.78 | 8.95 |
| JL(mm) | 14 | 85.45 | 8.81 | 88.42 | 73.04 | 94.82 | 21.78 | 2.36 |
| Ix | 14 | 145.37 | 35.95 | 143.89 | 70.84 | 203.60 | 132.77 | 9.61 |
| Iy | 14 | 132.23 | 41.65 | 123.78 | 74.39 | 206.23 | 131.84 | 11.13 |
| $I_x/I_y$ | 14 | 1.16 | 0.35 | 1.07 | 0.72 | 1.96 | 1.24 | 0.09 |
| $I_{max}/I_{min}$ | 14 | 1.41 | 0.26 | 1.39 | 1.12 | 2.07 | 0.94 | 0.07 |
| $M_1$ $I_x/I_y$ | 5 | 1.13 | 0.26 | 0.97 | 0.94 | 1.53 | 0.59 | 0.12 |
| $M_2$ $I_x/I_y$ | 5 | 0.89 | 0.19 | 0.85 | 0.72 | 1.18 | 0.46 | 0.08 |
| $M_3$ $I_x/I_y$ | 4 | 1.53 | 0.29 | 1.40 | 1.36 | 1.96 | 0.60 | 0.14 |
| $M_1$ $I_{max}/I_{min}$ | 5 | 1.29 | 0.20 | 1.20 | 1.12 | 1.60 | 0.48 | 0.09 |
| $M_2$ $I_{max}/I_{min}$ | 5 | 1.30 | 0.14 | 1.22 | 1.20 | 1.51 | 0.31 | 0.06 |
| $M_3$ $I_{max}/I_{min}$ | 4 | 1.68 | 0.26 | 1.56 | 1.54 | 2.07 | 0.52 | 0.13 |

| *Colobus* | | | | | | | | |
|---|---|---|---|---|---|---|---|---|
| | n | mean | sd | median | min | max | range | se |

| | | | | | | | | |
|---|---|---|---|---|---|---|---|---|
| DA | 14 | 26.31 | 3.88 | 27.56 | 19.11 | 31.90 | 12.79 | 1.04 |
| $I_{max}$ | 14 | 102.78 | 39.23 | 100.11 | 43.40 | 166.81 | 123.41 | 10.48 |
| $I_{min}$ | 14 | 53.58 | 15.20 | 50.05 | 37.14 | 87.59 | 50.45 | 4.06 |
| JL(mm) | 14 | 86.02 | 1.95 | 86.15 | 82.89 | 88.56 | 5.67 | 0.52 |
| Ix | 14 | 102.13 | 39.10 | 99.75 | 42.06 | 165.04 | 122.98 | 10.45 |
| Iy | 14 | 54.23 | 14.98 | 50.19 | 38.78 | 87.64 | 48.86 | 4.00 |
| $I_x/I_y$ | 14 | 1.93 | 0.81 | 1.62 | 1.08 | 3.59 | 2.50 | 0.22 |
| $I_{max}/I_{min}$ | 14 | 1.98 | 0.85 | 1.64 | 1.16 | 3.80 | 2.64 | 0.23 |
| $M_1$ $I_x/I_y$ | 4 | 1.48 | 0.29 | 1.56 | 1.08 | 1.73 | 0.64 | 0.15 |
| $M_2$ $I_x/I_y$ | 5 | 1.39 | 0.20 | 1.38 | 1.08 | 1.58 | 0.49 | 0.09 |
| $M_3$ $I_x/I_y$ | 5 | 2.84 | 0.66 | 2.47 | 2.30 | 3.59 | 1.29 | 0.30 |
| $M_1$ $I_{max}/I_{min}$ | 4 | 1.51 | 0.26 | 1.58 | 1.16 | 1.74 | 0.58 | 0.13 |
| $M_2$ $I_{max}/I_{min}$ | 5 | 1.41 | 0.17 | 1.38 | 1.18 | 1.58 | 0.41 | 0.08 |
| $M_3$ $I_{max}/I_{min}$ | 5 | 2.92 | 0.73 | 2.48 | 2.32 | 3.80 | 1.48 | 0.33 |
| *Erythrocebus* | | | | | | | | |
| | n | mean | sd | median | min | max | range | se |
| DA | 11 | 23.93 | 4.26 | 23.54 | 16.12 | 31.50 | 15.38 | 1.29 |
| $I_{max}$ | 11 | 91.66 | 33.51 | 92.88 | 47.30 | 159.21 | 111.91 | 10.10 |
| $I_{min}$ | 11 | 46.18 | 17.49 | 42.94 | 20.60 | 77.64 | 57.04 | 5.27 |
| JL(mm) | 11 | 100.53 | 6.68 | 103.51 | 90.19 | 105.28 | 15.09 | 2.01 |
| Ix | 11 | 91.42 | 33.51 | 91.28 | 47.30 | 159.18 | 111.89 | 10.10 |
| Iy | 11 | 46.42 | 17.64 | 42.95 | 20.61 | 77.67 | 57.06 | 5.32 |
| $I_x/I_y$ | 11 | 2.02 | 0.37 | 2.05 | 1.49 | 2.70 | 1.21 | 0.11 |
| $I_{max}/I_{min}$ | 11 | 2.03 | 0.36 | 2.05 | 1.49 | 2.70 | 1.21 | 0.11 |
| $M_1$ $I_x/I_y$ | 3 | 2.47 | 0.21 | 2.42 | 2.29 | 2.70 | 0.40 | 0.12 |
| $M_2$ $I_x/I_y$ | 4 | 1.84 | 0.29 | 1.89 | 1.49 | 2.11 | 0.62 | 0.14 |
| $M_3$ $I_x/I_y$ | 4 | 1.87 | 0.26 | 1.94 | 1.51 | 2.08 | 0.56 | 0.13 |
| $M_1$ $I_{max}/I_{min}$ | 3 | 2.47 | 0.21 | 2.42 | 2.30 | 2.70 | 0.40 | 0.12 |
| $M_2$ $I_{max}/I_{min}$ | 4 | 1.85 | 0.28 | 1.91 | 1.49 | 2.11 | 0.62 | 0.14 |
| $M_3$ $I_{max}/I_{min}$ | 4 | 1.89 | 0.23 | 1.94 | 1.58 | 2.09 | 0.50 | 0.11 |
| *Gorilla* | | | | | | | | |
| | n | mean | sd | median | min | max | range | se |
| DA | 26 | 121.67 | 17.29 | 122.51 | 98.43 | 156.81 | 58.38 | 3.39 |
| $I_{max}$ | 26 | 2236.73 | 639.37 | 2081.89 | 1245.80 | 3864.09 | 2618.29 | 125.39 |
| $I_{min}$ | 26 | 1664.26 | 542.41 | 1526.10 | 678.34 | 2997.21 | 2318.86 | 106.37 |
| JL(mm) | 26 | 184.29 | 18.66 | 196.09 | 157.29 | 203.38 | 46.09 | 3.66 |
| Ix | 26 | 2158.73 | 669.39 | 2019.28 | 1084.67 | 3813.11 | 2728.44 | 131.28 |
| Iy | 26 | 1742.26 | 541.02 | 1687.87 | 680.39 | 3048.19 | 2367.80 | 106.10 |
| $I_x/I_y$ | 26 | 1.27 | 0.27 | 1.25 | 0.74 | 1.91 | 1.17 | 0.05 |
| $I_{max}/I_{min}$ | 26 | 1.38 | 0.23 | 1.31 | 1.06 | 2.12 | 1.05 | 0.04 |

| | | | | | | | | |
|---|---|---|---|---|---|---|---|---|
| $M_1$ $I_x/I_y$ | 9 | 1.18 | 0.18 | 1.22 | 0.83 | 1.40 | 0.56 | 0.06 |
| $M_2$ $I_x/I_y$ | 9 | 1.19 | 0.27 | 1.25 | 0.74 | 1.50 | 0.76 | 0.09 |
| $M_3$ $I_x/I_y$ | 8 | 1.46 | 0.27 | 1.38 | 1.23 | 1.91 | 0.68 | 0.09 |
| $M_1$ $I_{max}/I_{min}$ | 9 | 1.25 | 0.11 | 1.24 | 1.06 | 1.44 | 0.38 | 0.04 |
| $M_2$ $I_{max}/I_{min}$ | 9 | 1.35 | 0.13 | 1.32 | 1.17 | 1.57 | 0.40 | 0.04 |
| $M_3$ $I_{max}/I_{min}$ | 8 | 1.55 | 0.31 | 1.48 | 1.26 | 2.12 | 0.86 | 0.11 |
| *Hylobates* | | | | | | | | |
| | n | mean | sd | median | min | max | range | se |
| DA | 27 | 13.81 | 2.90 | 13.13 | 9.11 | 19.42 | 10.31 | 0.56 |
| $I_{max}$ | 27 | 31.22 | 11.14 | 27.93 | 15.04 | 58.39 | 43.35 | 2.14 |
| $I_{min}$ | 27 | 16.90 | 6.05 | 15.80 | 8.72 | 32.32 | 23.60 | 1.16 |
| JL(mm) | 27 | 69.45 | 2.28 | 69.38 | 65.74 | 72.70 | 6.96 | 0.44 |
| Ix | 27 | 30.81 | 11.15 | 26.76 | 13.77 | 57.68 | 43.92 | 2.15 |
| Iy | 27 | 17.32 | 6.11 | 16.00 | 8.91 | 32.51 | 23.60 | 1.18 |
| $I_x/I_y$ | 27 | 1.81 | 0.39 | 1.80 | 1.21 | 2.74 | 1.53 | 0.08 |
| $I_{max}/I_{min}$ | 27 | 1.88 | 0.38 | 1.82 | 1.42 | 2.81 | 1.39 | 0.07 |
| $M_1$ $I_x/I_y$ | 9 | 1.78 | 0.28 | 1.82 | 1.21 | 2.15 | 0.94 | 0.09 |
| $M_2$ $I_x/I_y$ | 9 | 1.76 | 0.44 | 1.59 | 1.33 | 2.68 | 1.34 | 0.15 |
| $M_3$ $I_x/I_y$ | 9 | 1.90 | 0.46 | 1.91 | 1.33 | 2.74 | 1.41 | 0.15 |
| $M_1$ $I_{max}/I_{min}$ | 9 | 1.86 | 0.24 | 1.91 | 1.49 | 2.23 | 0.75 | 0.08 |
| $M_2$ $I_{max}/I_{min}$ | 9 | 1.80 | 0.43 | 1.65 | 1.42 | 2.71 | 1.29 | 0.14 |
| $M_3$ $I_{max}/I_{min}$ | 9 | 2.00 | 0.46 | 2.06 | 1.47 | 2.81 | 1.34 | 0.15 |
| *Miopithecus* | | | | | | | | |
| | n | mean | sd | median | min | max | range | se |
| DA | 15 | 6.86 | 1.73 | 6.41 | 4.42 | 10.98 | 6.56 | 0.45 |
| $I_{max}$ | 15 | 6.97 | 3.00 | 5.65 | 2.82 | 13.06 | 10.24 | 0.77 |
| $I_{min}$ | 15 | 4.20 | 2.27 | 3.50 | 1.70 | 10.36 | 8.66 | 0.59 |
| JL(mm) | 15 | 45.04 | 2.57 | 46.02 | 40.25 | 47.22 | 6.97 | 0.66 |
| Ix | 15 | 6.82 | 2.89 | 5.55 | 2.77 | 12.46 | 9.70 | 0.75 |
| Iy | 15 | 4.35 | 2.39 | 3.57 | 1.75 | 10.96 | 9.21 | 0.62 |
| $I_x/I_y$ | 15 | 1.66 | 0.36 | 1.56 | 1.14 | 2.29 | 1.15 | 0.09 |
| $I_{max}/I_{min}$ | 15 | 1.75 | 0.39 | 1.65 | 1.26 | 2.49 | 1.23 | 0.10 |
| $M_1$ $I_x/I_y$ | 5 | 1.93 | 0.46 | 2.24 | 1.30 | 2.29 | 0.99 | 0.21 |
| $M_2$ $I_x/I_y$ | 5 | 1.41 | 0.22 | 1.41 | 1.14 | 1.68 | 0.54 | 0.10 |
| $M_3$ $I_x/I_y$ | 5 | 1.64 | 0.19 | 1.55 | 1.53 | 1.97 | 0.44 | 0.08 |
| $M_1$ $I_{max}/I_{min}$ | 5 | 2.05 | 0.51 | 2.35 | 1.33 | 2.49 | 1.16 | 0.23 |
| $M_2$ $I_{max}/I_{min}$ | 5 | 1.49 | 0.20 | 1.54 | 1.26 | 1.73 | 0.47 | 0.09 |
| $M_3$ $I_{max}/I_{min}$ | 5 | 1.70 | 0.17 | 1.65 | 1.58 | 2.00 | 0.42 | 0.08 |
| *Pan* | | | | | | | | |
| | n | mean | sd | median | min | max | range | se |

| | n | mean | sd | median | min | max | range | se |
|---|---|---|---|---|---|---|---|---|
| DA | 24 | 48.40 | 8.12 | 46.24 | 35.01 | 60.06 | 25.05 | 1.66 |
| $I_{max}$ | 24 | 317.24 | 94.44 | 292.62 | 168.71 | 504.75 | 336.04 | 19.28 |
| $I_{min}$ | 24 | 259.04 | 90.77 | 226.28 | 125.68 | 441.08 | 315.40 | 18.53 |
| JL(mm) | 24 | 133.69 | 7.45 | 132.39 | 125.67 | 146.68 | 21.01 | 1.52 |
| Ix | 24 | 303.37 | 96.94 | 288.52 | 157.62 | 493.88 | 336.26 | 19.79 |
| Iy | 24 | 272.91 | 91.71 | 257.51 | 133.84 | 446.75 | 312.91 | 18.72 |
| $I_x/I_y$ | 24 | 1.14 | 0.22 | 1.07 | 0.71 | 1.62 | 0.91 | 0.04 |
| $I_{max}/I_{min}$ | 24 | 1.26 | 0.17 | 1.21 | 1.06 | 1.62 | 0.56 | 0.03 |
| $M_1$ $I_x/I_y$ | 8 | 1.02 | 0.21 | 1.00 | 0.71 | 1.37 | 0.66 | 0.07 |
| $M_2$ $I_x/I_y$ | 7 | 1.12 | 0.17 | 1.03 | 1.00 | 1.39 | 0.39 | 0.07 |
| $M_3$ $I_x/I_y$ | 9 | 1.26 | 0.22 | 1.18 | 0.96 | 1.62 | 0.66 | 0.07 |
| $M_1$ $I_{max}/I_{min}$ | 8 | 1.26 | 0.15 | 1.19 | 1.12 | 1.55 | 0.44 | 0.05 |
| $M_2$ $I_{max}/I_{min}$ | 7 | 1.17 | 0.14 | 1.11 | 1.06 | 1.39 | 0.33 | 0.05 |
| $M_3$ $I_{max}/I_{min}$ | 9 | 1.32 | 0.19 | 1.26 | 1.09 | 1.62 | 0.54 | 0.06 |
| | *Papio* | | | | | | | |
| | n | mean | sd | median | min | max | range | se |
| DA | 15 | 73.19 | 17.64 | 75.70 | 44.74 | 106.42 | 61.68 | 4.56 |
| $I_{max}$ | 15 | 942.22 | 583.27 | 891.01 | 309.79 | 2174.37 | 1864.58 | 150.60 |
| $I_{min}$ | 15 | 495.86 | 233.45 | 402.99 | 154.31 | 948.46 | 794.15 | 60.28 |
| JL(mm) | 15 | 143.07 | 11.43 | 147.55 | 118.72 | 153.29 | 34.57 | 2.95 |
| Ix | 15 | 931.75 | 578.14 | 885.99 | 306.28 | 2141.61 | 1835.33 | 149.28 |
| Iy | 15 | 506.33 | 241.67 | 411.47 | 157.82 | 998.12 | 840.30 | 62.40 |
| $I_x/I_y$ | 15 | 1.85 | 0.69 | 1.56 | 1.09 | 3.25 | 2.16 | 0.18 |
| $I_{max}/I_{min}$ | 15 | 1.91 | 0.72 | 1.57 | 1.19 | 3.34 | 2.16 | 0.18 |
| $M_1$ $I_x/I_y$ | 5 | 1.61 | 0.23 | 1.56 | 1.35 | 1.94 | 0.59 | 0.10 |
| $M_2$ $I_x/I_y$ | 6 | 1.37 | 0.24 | 1.36 | 1.09 | 1.75 | 0.65 | 0.10 |
| $M_3$ $I_x/I_y$ | 4 | 2.88 | 0.30 | 2.86 | 2.57 | 3.25 | 0.69 | 0.15 |
| $M_1$ $I_{max}/I_{min}$ | 5 | 1.64 | 0.23 | 1.57 | 1.40 | 2.01 | 0.60 | 0.10 |
| $M_2$ $I_{max}/I_{min}$ | 6 | 1.42 | 0.23 | 1.40 | 1.19 | 1.79 | 0.61 | 0.10 |
| $M_3$ $I_{max}/I_{min}$ | 4 | 2.98 | 0.33 | 2.96 | 2.65 | 3.34 | 0.70 | 0.16 |
| | *Pongo* | | | | | | | |
| | n | mean | sd | median | min | max | range | se |
| DA | 27 | 82.65 | 12.34 | 81.09 | 57.94 | 109.18 | 51.24 | 2.38 |
| $I_{max}$ | 27 | 962.72 | 322.43 | 892.64 | 479.52 | 1725.80 | 1246.29 | 62.05 |
| $I_{min}$ | 27 | 772.55 | 301.97 | 709.53 | 359.13 | 1511.32 | 1152.19 | 58.11 |
| JL(mm) | 27 | 157.48 | 16.88 | 155.48 | 132.58 | 177.85 | 45.27 | 3.25 |
| Ix | 27 | 938.37 | 320.35 | 823.08 | 478.45 | 1697.57 | 1219.11 | 61.65 |
| Iy | 27 | 796.90 | 307.65 | 791.91 | 360.47 | 1539.55 | 1179.08 | 59.21 |
| $I_x/I_y$ | 27 | 1.21 | 0.17 | 1.16 | 0.95 | 1.57 | 0.62 | 0.03 |
| $I_{max}/I_{min}$ | 27 | 1.28 | 0.14 | 1.26 | 1.10 | 1.57 | 0.47 | 0.03 |

| | | | | | | | | |
|---|---|---|---|---|---|---|---|---|
| $M_1$ $I_x/I_y$ | 9 | 1.12 | 0.10 | 1.13 | 0.95 | 1.27 | 0.32 | 0.03 |
| $M_2$ $I_x/I_y$ | 9 | 1.15 | 0.09 | 1.14 | 1.02 | 1.27 | 0.24 | 0.03 |
| $M_3$ $I_x/I_y$ | 9 | 1.36 | 0.19 | 1.36 | 1.07 | 1.57 | 0.50 | 0.06 |
| $M_1$ $I_{max}/I_{min}$ | 9 | 1.26 | 0.05 | 1.27 | 1.16 | 1.33 | 0.17 | 0.02 |
| $M_2$ $I_{max}/I_{min}$ | 9 | 1.19 | 0.08 | 1.18 | 1.10 | 1.32 | 0.22 | 0.03 |
| $M_3$ $I_{max}/I_{min}$ | 9 | 1.39 | 0.17 | 1.37 | 1.14 | 1.57 | 0.43 | 0.06 |
| *Symphalangus* | | | | | | | | |
| | n | mean | sd | median | min | max | range | se |
| DA | 7 | 26.38 | 5.27 | 25.43 | 19.07 | 34.90 | 15.83 | 1.99 |
| $I_{max}$ | 7 | 123.14 | 50.50 | 135.24 | 48.00 | 179.62 | 131.62 | 19.09 |
| $I_{min}$ | 7 | 66.36 | 23.96 | 58.92 | 36.48 | 105.42 | 68.94 | 9.05 |
| JL(mm) | 7 | 82.27 | 4.96 | 83.58 | 75.40 | 87.17 | 11.77 | 1.87 |
| Ix | 7 | 122.64 | 50.42 | 134.84 | 47.88 | 179.53 | 131.65 | 19.06 |
| Iy | 7 | 66.85 | 24.00 | 59.41 | 36.60 | 106.11 | 69.52 | 9.07 |
| $I_x/I_y$ | 7 | 1.82 | 0.48 | 1.93 | 1.15 | 2.43 | 1.28 | 0.18 |
| $I_{max}/I_{min}$ | 7 | 1.84 | 0.49 | 1.94 | 1.16 | 2.44 | 1.28 | 0.18 |
| $M_1$ $I_x/I_y$ | 3 | 1.89 | 0.56 | 1.93 | 1.31 | 2.43 | 1.12 | 0.32 |
| $M_2$ $I_x/I_y$ | 3 | 1.59 | 0.43 | 1.63 | 1.15 | 2.00 | 0.85 | 0.25 |
| $M_3$ $I_x/I_y$ | 1 | 2.27 | NA | 2.27 | 2.27 | 2.27 | NA | NA |
| $M_1$ $I_{max}/I_{min}$ | 3 | 1.90 | 0.56 | 1.94 | 1.32 | 2.44 | 1.12 | 0.32 |
| $M_2$ $I_{max}/I_{min}$ | 3 | 1.63 | 0.47 | 1.64 | 1.16 | 2.09 | 0.93 | 0.27 |
| $M_3$ $I_{max}/I_{min}$ | 1 | 2.29 | NA | 2.29 | 2.29 | 2.29 | NA | NA |
| *Theropithecus* | | | | | | | | |
| | n | mean | sd | median | min | max | range | se |
| DA | 8 | 54.83 | 17.26 | 59.79 | 33.55 | 81.16 | 47.61 | 6.10 |
| $I_{max}$ | 8 | 520.90 | 371.32 | 480.95 | 155.23 | 1249.24 | 1094.01 | 131.28 |
| $I_{min}$ | 8 | 288.71 | 135.47 | 347.67 | 120.82 | 444.00 | 323.19 | 47.90 |
| JL(mm) | 8 | 123.55 | 5.74 | 124.33 | 112.78 | 128.98 | 16.20 | 2.03 |
| Ix | 8 | 505.41 | 368.42 | 467.42 | 137.16 | 1225.55 | 1088.39 | 130.26 |
| Iy | 8 | 304.20 | 136.80 | 363.61 | 138.89 | 456.99 | 318.11 | 48.37 |
| $I_x/I_y$ | 8 | 1.52 | 0.62 | 1.35 | 0.95 | 2.79 | 1.85 | 0.22 |
| $I_{max}/I_{min}$ | 8 | 1.69 | 0.62 | 1.43 | 1.21 | 3.01 | 1.80 | 0.22 |
| $M_1$ $I_x/I_y$ | 3 | 1.14 | 0.30 | 0.99 | 0.95 | 1.49 | 0.54 | 0.18 |
| $M_2$ $I_x/I_y$ | 3 | 1.31 | 0.09 | 1.29 | 1.24 | 1.41 | 0.17 | 0.05 |
| $M_3$ $I_x/I_y$ | 2 | 2.42 | 0.53 | 2.42 | 2.04 | 2.79 | 0.75 | 0.38 |
| $M_1$ $I_{max}/I_{min}$ | 3 | 1.37 | 0.21 | 1.28 | 1.21 | 1.61 | 0.40 | 0.12 |
| $M_2$ $I_{max}/I_{min}$ | 3 | 1.39 | 0.08 | 1.38 | 1.32 | 1.48 | 0.16 | 0.05 |
| $M_3$ $I_{max}/I_{min}$ | 2 | 2.61 | 0.57 | 2.61 | 2.20 | 3.01 | 0.81 | 0.40 |

## Cross-Sectional Variable $\sigma^2$ by Tooth Position

### Table S4. Dentine Area $\sigma^2$ by Diet

| DA | | | |
|---|---|---|---|
| | $M_1$ | $M_2$ | $M_3$ |
| Sample | 1400.72 | 1666.83 | 1361.18 |
| Folivore | 53.76 | 335.71 | 513.76 |
| Mixed Folivore | 2001.58 | 2436.08 | 1067.20 |
| Omnivore | 488.75 | 1048.74 | 1494.56 |
| Soft Object Frugivore | 370.12 | 403.37 | 286.26 |
| Hard Object Frugivore | 611.21 | 781.73 | 566.46 |

### Table S5. $I_x/I_y$ σ2 by Diet

| $I_x/I_y$ | | | |
|---|---|---|---|
| | $M_1$ | $M_2$ | $M_3$ |
| Sample | 0.22 | 0.13 | 0.39 |
| Folivore | 0.11 | 0.03 | 0.38 |
| Mixed Folivore | 0.19 | 0.12 | 0.13 |
| Omnivore | 0.21 | 0.10 | 0.36 |
| Soft Object Frugivore | 0.21 | 0.22 | 0.23 |
| Hard Object Frugivore | 0.03 | 0.03 | 0.05 |

### Table S6. Wilcoxon Rank Sum Pairwise Comparisons $I_{max}/I_{min}$

| $I_{max}/I_{min}$ | | | | | |
|---|---|---|---|---|---|
| | Folivore | Mixed Folivore | Omnivore | Soft Object Frugivore | Hard Object Frugivore |
| Folivore | 1.000 | - | - | - | - |
| Mixed Folivore | 0.06 | 1.000 | - | - | - |
| Omnivore | 0.25 | **<0.001** | 1.000 | - | - |
| Soft Object Frugivore | 0.30 | 0.30 | **0.008** | 1.000 | - |
| Hard Object Frugivore | **0.001** | 0.05 | **<0.001** | **0.009** | 1.000 |
| Significant values bolded ($p < 0.05$) | | | | | |

### Table S7. $I_{max}/I_{min}$ σ2 by Diet

| $I_{max}/I_{min}$ | | | |
|---|---|---|---|
| | $M_1$ | $M_2$ | $M_3$ |
| Sample | 0.18 | 0.10 | 0.41 |
| Folivore | 0.06 | 0.02 | 0.43 |
| Mixed Folivore | 0.15 | 0.07 | 0.15 |
| Omnivore | 0.22 | 0.08 | 0.38 |
| Soft Object Frugivore | 0.13 | 0.21 | 0.24 |
| Hard Object Frugivore | 0.02 | 0.01 | 0.06 |

Figure S1. Boxplot of $I_{max}/I_{min}$ ratios for the $M_1$ tooth position by dietary category. Thick horizontal bars within each box represent the median and asterisk symbols represent mean values for each diet. Single black dots represent outliers.

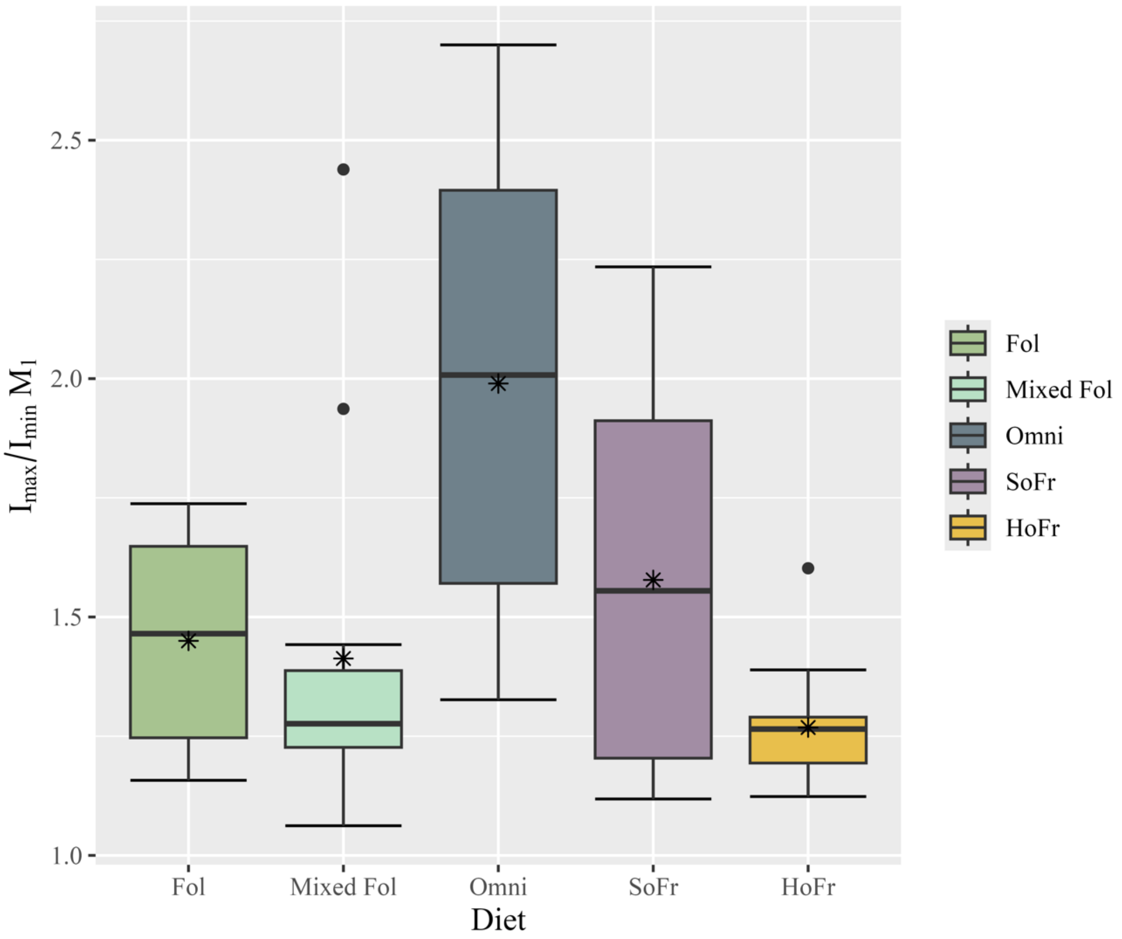

Figure S2. Boxplot of $I_{max}/I_{min}$ ratios for the $M_2$ tooth position by dietary category. Thick horizontal bars within each box represent the median and asterisk symbols represent mean values for each diet. Single black dots represent outliers.

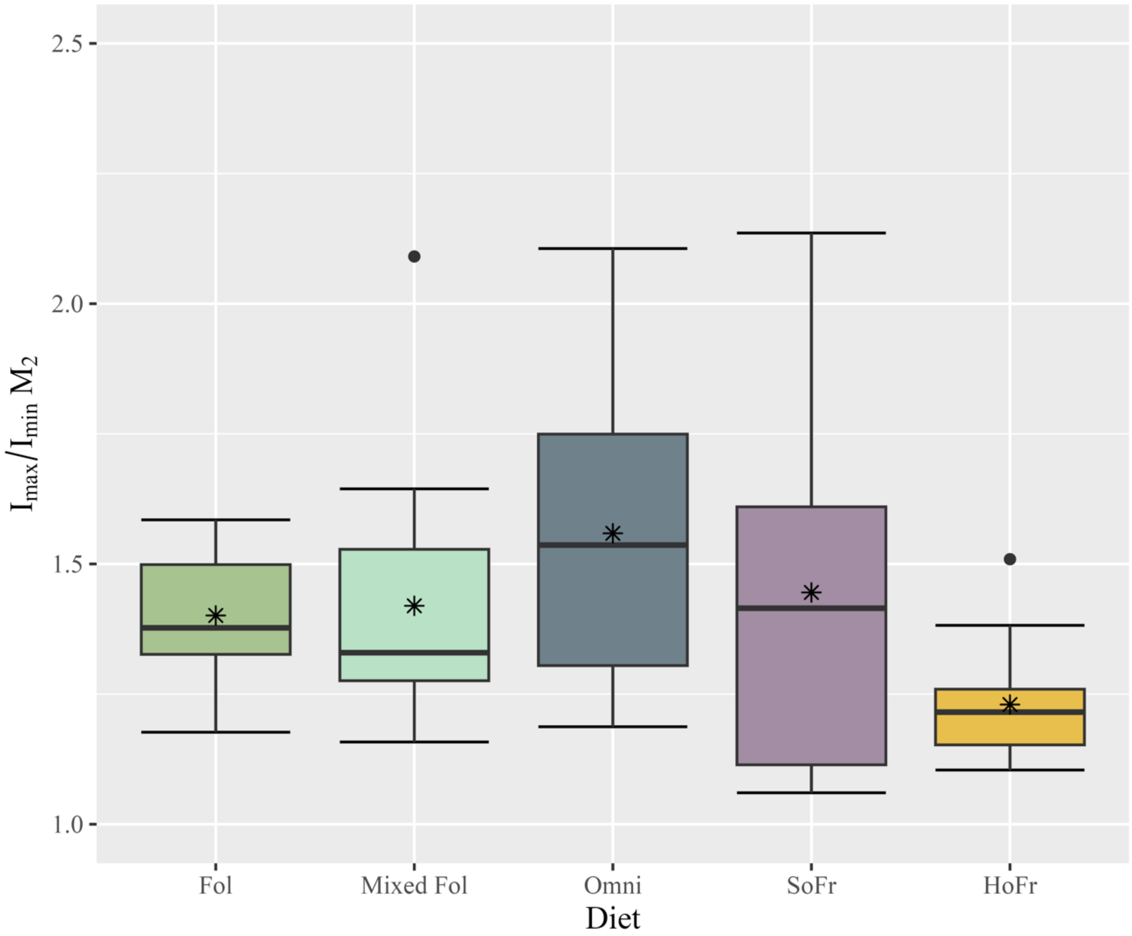

Figure S3. Boxplot of $I_{max}/I_{min}$ ratios for the $M_3$ tooth position by dietary category. Thick horizontal bars within each box represent the median and asterisk symbols represent mean values for each diet. Single black dots represent outliers.

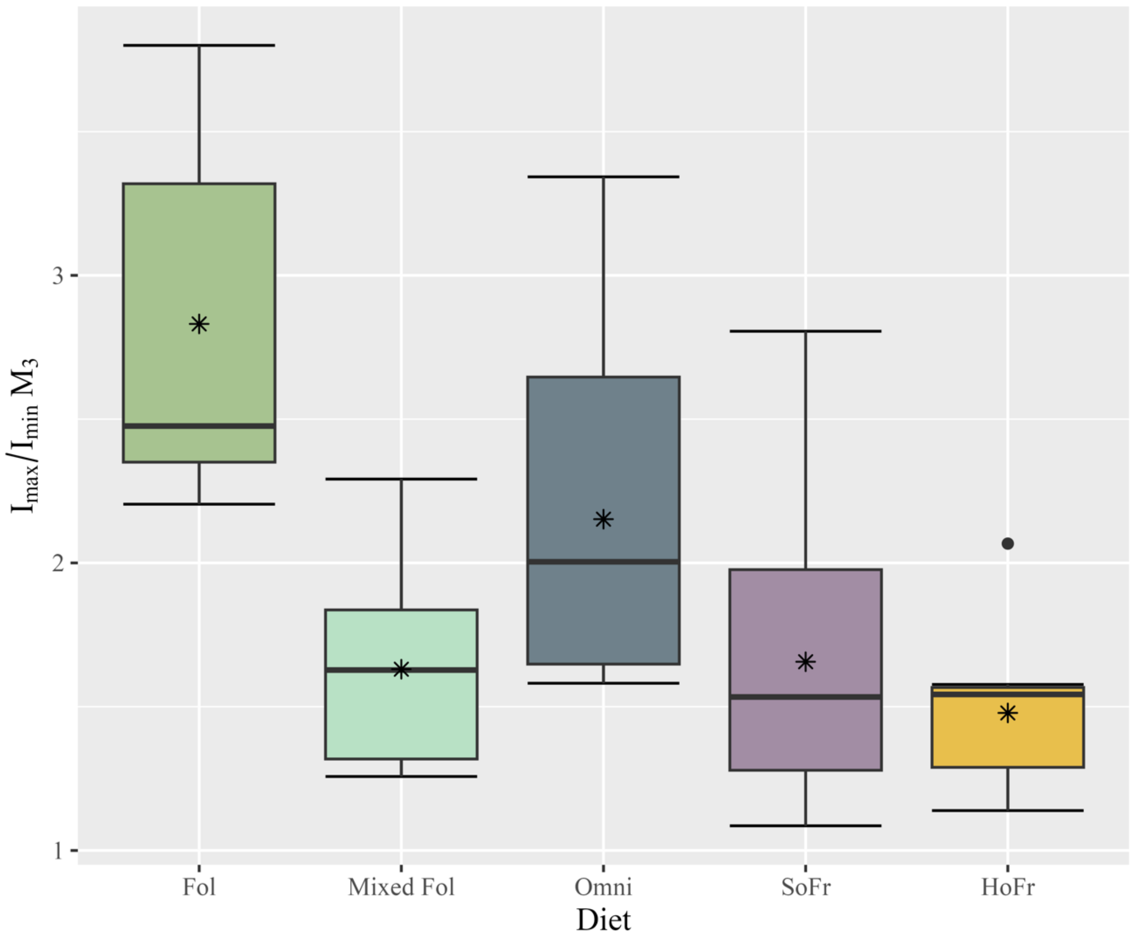

Table S8. Raw MomentMacro Results, Mandibular Length, and Image Scale Data

| ID | Genus | Tooth Position | Diet | Sex | TA | DA | Ix | Iy | Imax | Imin | JL | Image Scale |
|---|---|---|---|---|---|---|---|---|---|---|---|---|
| 52634 | *Cercocebus* | Rm1 | HoFr | M | 40.3797 | 33.9909 | 147.9681 | 117.6868 | 154.4603 | 111.1946 | 94.82 | 13.2993pixels/mm |
| 52634 | *Cercocebus* | Rm2 | HoFr | M | 47.3722 | 39.2898 | 178.5282 | 185.1593 | 199.9046 | 163.7829 | 94.82 | 13.3849pixels/mm |
| 52634 | *Cercocebus* | Rm3 | HoFr | M | 38.7749 | 33.339 | 144.886 | 106.607 | 152.6732 | 98.8197 | 94.82 | 14.2963pixels/mm |
| 52635 | *Cercocebus* | Rm1 | HoFr | F | 35.9673 | 29.0989 | 125.7113 | 81.9135 | 127.8375 | 79.7873 | 73.04 | 12.4522pixels/mm |
| 52635 | *Cercocebus* | Rm2 | HoFr | F | 48.6095 | 40.5197 | 203.6018 | 172.8444 | 205.4669 | 170.9792 | 73.04 | 12.4652pixels/mm |
| 52635 | *Cercocebus* | Rm3 | HoFr | F | 38.9181 | 33.3519 | 176.9111 | 90.3306 | 180.1124 | 87.1294 | 73.04 | 13.3567pixels/mm |
| 52640 | *Cercocebus* | Rm1 | HoFr | F | 30.3207 | 24.9849 | 70.8351 | 74.3915 | 77.4562 | 67.7704 | 76.68 | 16.0411pixels/mm |
| 52640 | *Cercocebus* | Rm2 | HoFr | F | 41.0889 | 32.6324 | 111.7204 | 156.2492 | 161.1728 | 106.7968 | 76.68 | 16.1914pixels/mm |
| 52641 | *Cercocebus* | Rm1 | HoFr | M | 36.7866 | 29.8783 | 103.361 | 109.5185 | 116.1968 | 96.6827 | 91.35 | 13.3813pixels/mm |
| 52641 | *Cercocebus* | Rm2 | HoFr | M | 44.1094 | 36.7813 | 134.8035 | 177.6175 | 181.2653 | 131.1557 | 91.35 | 13.8466pixels/mm |
| 52641 | *Cercocebus* | Rm3 | HoFr | M | 38.1675 | 33.0017 | 142.8862 | 102.2635 | 148.7366 | 96.4131 | 91.35 | 14.6718pixels/mm |
| 52645 | *Cercocebus* | Rm1 | HoFr | M | 41.1588 | 36.6546 | 136.2431 | 140.5487 | 146.4398 | 130.352 | 88.42 | 12.5019pixels/mm |
| 52645 | *Cercocebus* | Rm2 | HoFr | M | 48.5384 | 41.992 | 175.1415 | 206.2328 | 208.8235 | 172.5508 | 88.42 | 12.1726pixels/mm |
| 52645 | *Cercocebus* | Rm3 | HoFr | M | 42.8474 | 36.5504 | 182.5538 | 129.8636 | 191.2034 | 121.214 | 88.42 | 10.3535pixels/mm |
| 52209 | *Colobus* | Rm1 | Fol | M | 26.1078 | 22.1454 | 70.074 | 40.5899 | 70.2403 | 40.4236 | 88.56 | 12.3871pixels/mm |
| 52209 | *Colobus* | Rm2 | Fol | M | 30.7014 | 27.3532 | 88.9465 | 64.656 | 88.9553 | 64.6472 | 88.56 | 13.2069pixels/mm |
| 52209 | *Colobus* | Rm3 | Fol | M | 30.6874 | 27.0968 | 116.3666 | 50.4412 | 116.5501 | 50.2577 | 88.56 | 13.711pixels/mm |
| 52210 | *Colobus* | Rm1 | Fol | M | 3.5677 | 28.932 | 101.9687 | 70.5469 | 102.532 | 69.9837 | 82.89 | 13.3935pixels/mm |
| 52210 | *Colobus* | Rm2 | Fol | M | 35.6621 | 30.4755 | 116.3273 | 87.6382 | 116.3787 | 87.5868 | 82.89 | 14.4768pixels/mm |
| 52210 | *Colobus* | Rm3 | Fol | M | 36.4044 | 31.9038 | 165.0361 | 71.8053 | 166.8084 | 70.033 | 82.89 | 14.8315pixels/mm |
| 52215 | *Colobus* | Rm2 | Fol | F | 28.1802 | 24.7101 | 78.1222 | 49.938 | 78.2116 | 49.8486 | 86.67 | 15.6693pixels/mm |
| 52215 | *Colobus* | Rm3 | Fol | F | 31.7503 | 28.3669 | 157.0261 | 43.7709 | 158.962 | 41.835 | 86.67 | 13.6998pixels/mm |
| 52237 | *Colobus* | Rm1 | Fol | M | 22.6347 | 19.1092 | 42.0698 | 38.825 | 43.4 | 37.4948 | 86.15 | 12.6481pixels/mm |
| 52237 | *Colobus* | Rm2 | Fol | M | 22.5035 | 19.5593 | 42.0602 | 38.783 | 43.7043 | 37.139 | 86.15 | 12.6481pixels/mm |
| 52237 | *Colobus* | Rm3 | Fol | M | 32.6275 | 27.7742 | 134.0292 | 54.1983 | 134.0771 | 54.1504 | 86.15 | 13.1872pixels/mm |

| | | | | | | | | | | | | |
|---|---|---|---|---|---|---|---|---|---|---|---|---|
| 52238 | *Colobus* | Rm1 | Fol | F | 27.3388 | 23.9983 | 75.9185 | 45.469 | 76.2609 | 45.1266 | 86.03 | 14.0347pixels/mm |
| 52238 | *Colobus* | Rm2 | Fol | F | 31.0435 | 28.492 | 97.541 | 61.7857 | 97.6856 | 61.641 | 86.03 | 14.6152pixels/mm |
| 52238 | *Colobus* | Rm3 | Fol | F | 30.2118 | 28.3595 | 144.351 | 40.8032 | 145.1367 | 40.0175 | 86.03 | 14.398pixels/mm |
| 34714 | *Erythrocebus* | Rm2 | Omni | M | 25.3659 | 23.3911 | 63.9939 | 42.9511 | 64.0001 | 42.9449 | 104.44 | 16.9446pixels/mm |
| 34714 | *Erythrocebus* | Rm3 | Omni | M | 21.3509 | 19.5428 | 54.4595 | 26.2191 | 54.5407 | 26.1379 | 104.44 | 17.4883pixels/mm |
| 37280 | *Erythrocebus* | Rm1 | Omni | M | 19.9033 | 16.1183 | 47.2967 | 20.6105 | 47.3031 | 20.6041 | 90.19 | 11.5279pixels/mm |
| 37280 | *Erythrocebus* | Rm2 | Omni | M | 29.821 | 26.0059 | 95.1203 | 55.0278 | 95.9472 | 54.2009 | 90.19 | 11.5279pixels/mm |
| 37280 | *Erythrocebus* | Rm3 | Omni | M | 29.968 | 25.1017 | 91.2845 | 60.3201 | 92.883 | 58.7216 | 90.19 | 11.9507pixels/mm |
| 47015 | *Erythrocebus* | Rm1 | Omni | M | 26.6939 | 23.5372 | 89.3802 | 36.9294 | 89.4173 | 36.8922 | 105.28 | 10.7677pixels/mm |
| 47015 | *Erythrocebus* | Rm2 | Omni | M | 34.2598 | 28.702 | 136.9511 | 65.0328 | 136.9527 | 65.0312 | 105.28 | 11.1261pixels/mm |
| 47015 | *Erythrocebus* | Rm3 | Omni | M | 30.4992 | 25.9649 | 102.4208 | 55.7326 | 102.4236 | 55.7298 | 105.28 | 11.7027pixels/mm |
| 47016 | *Erythrocebus* | Rm1 | Omni | M | 26.8614 | 22.262 | 93.002 | 34.4541 | 93.0076 | 34.4485 | 103.51 | 10.6942pixels/mm |
| 47016 | *Erythrocebus* | Rm2 | Omni | M | 37.6379 | 31.5012 | 159.1826 | 77.6705 | 159.2132 | 77.6399 | 103.51 | 11.0034pixels/mm |
| 47016 | *Erythrocebus* | Rm3 | Omni | M | 25.1543 | 21.0538 | 72.5442 | 35.6905 | 72.6029 | 35.6318 | 103.51 | 11.8932pixels/mm |
| 14750 | *Gorilla* | Rm1 | Mixed Fol | F | 138.1137 | 115.183 | 1727.1706 | 1261.1902 | 1727.1733 | 1261.1875 | 157.29 | 10.4665pixels/mm |
| 14750 | *Gorilla* | Rm2 | Mixed Fol | F | 171.4101 | 139.4763 | 2772.7783 | 1842.696 | 2780.6692 | 1834.8051 | 157.29 | 10.6911pixels/mm |
| 14750 | *Gorilla* | Rm3 | Mixed Fol | F | 106.7586 | 98.4307 | 1243.7542 | 680.389 | 1245.8024 | 678.3408 | 157.29 | 10.1206pixels/mm |
| 20038 | *Gorilla* | Rm1 | Mixed Fol | M | 177.6154 | 143.1656 | 2532.6183 | 2285.8557 | 2582.9445 | 2235.5294 | 196.52 | 8.1103pixels/mm |
| 20038 | *Gorilla* | Rm2 | Mixed Fol | M | 215.0753 | 152.8043 | 3813.1091 | 3048.1858 | 3864.0894 | 2997.2055 | 196.52 | 8.1103pixels/mm |
| 20038 | *Gorilla* | Rm3 | Mixed Fol | M | 186.6155 | 133.375 | 2984.9641 | 2269.9403 | 2988.0488 | 2266.8557 | 196.52 | 8.1103pixels/mm |
| 20039 | *Gorilla* | Rm1 | Mixed Fol | M | 147.9372 | 129.4149 | 1904.9381 | 1561.2171 | 1918.4303 | 1547.7249 | 203.38 | 7.8864pixels/mm |
| 20039 | *Gorilla* | Rm2 | Mixed Fol | M | 191.6543 | 156.8125 | 3059.3556 | 2633.1635 | 3065.1629 | 2627.3562 | 203.38 | 7.8864pixels/mm |
| 20039 | *Gorilla* | Rm3 | Mixed Fol | M | 168.9838 | 135.0745 | 2694.2651 | 1865.3558 | 2831.3031 | 1728.3178 | 203.38 | 7.8864pixels/mm |
| 23160 | *Gorilla* | Rm1 | Mixed Fol | M | 183.9823 | 124.5449 | 2707.4025 | 2205.5761 | 2710.886 | 2202.0927 | 200.11 | 8.0451pixels/mm |
| 23160 | *Gorilla* | Rm2 | Mixed Fol | M | 202.569 | 125.0702 | 3101.8734 | 2611.1079 | 3156.9272 | 2556.054 | 200.11 | 8.0451pixels/mm |
| 23160 | *Gorilla* | Rm3 | Mixed Fol | M | 152.8808 | 99.5309 | 1881.7658 | 1501.5662 | 1927.6966 | 1455.6354 | 200.11 | 8.0451pixels/mm |
| 37264 | *Gorilla* | Rm1 | Mixed Fol | F | 126.6494 | 106.1 | 1451.1918 | 1108.3684 | 1451.8991 | 1107.6611 | 163.98 | 7.6258pixels/mm |
| 37264 | *Gorilla* | Rm2 | Mixed Fol | F | 146.1906 | 113.7856 | 1891.423 | 1459.0906 | 1918.5688 | 1431.9448 | 163.98 | 7.8187pixels/mm |
| 37264 | *Gorilla* | Rm3 | Mixed Fol | F | 144.7105 | 111.6699 | 1993.1068 | 1354.4508 | 2073.5204 | 1274.0371 | 163.98 | 7.9094pixels/mm |

| | | | | | | | | | | | | |
|---|---|---|---|---|---|---|---|---|---|---|---|---|
| 57482 | *Gorilla* | Rm1 | Mixed Fol | M | 147.9931 | 135.4123 | 2045.4546 | 1465.7317 | 2073.4 | 1437.7863 | 188.45 | 8.1566pixels/mm |
| 57482 | *Gorilla* | Rm2 | Mixed Fol | M | 173.5605 | 145.3778 | 2831.7608 | 1903.6354 | 2889.9528 | 1845.4435 | 188.45 | 8.1566pixels/mm |
| 167338 | *Gorilla* | Rm1 | Mixed Fol | M | 126.1725 | 103.0475 | 1084.6731 | 1300.2357 | 1307.2961 | 1077.6127 | 196.31 | 6.8781pixels/mm |
| 167338 | *Gorilla* | Rm2 | Mixed Fol | M | 153.0946 | 102.187 | 1483.1134 | 1913.87 | 1932.2901 | 1464.6933 | 196.31 | 7.3337pixels/mm |
| 167338 | *Gorilla* | Rm3 | Mixed Fol | M | 152.7528 | 102.8266 | 1874.205 | 1493.3586 | 1880.4603 | 1487.1032 | 196.31 | 7.6489pixels/mm |
| A999687 | *Gorilla* | Rm1 | Mixed Fol | F | 136.2289 | 106.3244 | 1518.9152 | 1291.2361 | 1556.5368 | 1253.6145 | 157.85 | 11.5292pixels/mm |
| A999687 | *Gorilla* | Rm2 | Mixed Fol | F | 162.1839 | 115.1369 | 2203.6748 | 1695.8444 | 2203.9709 | 1695.5484 | 157.85 | 11.8523pixels/mm |
| A999687 | *Gorilla* | Rm3 | Mixed Fol | F | 139.6631 | 99.4733 | 2046.8562 | 1070.227 | 2116.5804 | 1000.5029 | 157.85 | 11.931pixels/mm |
| L223 | *Gorilla* | Rm1 | Mixed Fol | M | 146.9767 | 124.2314 | 1661.9877 | 1713.23 | 1738.5272 | 1636.6905 | 196.09 | 9.9834pixels/mm |
| L223 | *Gorilla* | Rm2 | Mixed Fol | M | 151.2894 | 120.8319 | 1543.8036 | 2083.3643 | 2122.683 | 1504.4849 | 196.09 | 9.908pixels/mm |
| L223 | *Gorilla* | Rm3 | Mixed Fol | M | 154.4118 | 124.1883 | 2072.8568 | 1679.8866 | 2090.2541 | 1662.4893 | 196.09 | 10.4397pixels/mm |
| 12742 | *Hylobates* | Rm1 | SoFr | M | 17.9413 | 15.6379 | 35.9255 | 18.8183 | 35.9424 | 18.8014 | 71.07 | 16.2866pixels/mm |
| 12742 | *Hylobates* | Rm2 | SoFr | M | 20.3579 | 17.7189 | 46.7217 | 23.4309 | 46.7553 | 23.3973 | 71.07 | 16.2866pixels/mm |
| 12742 | *Hylobates* | Rm3 | SoFr | M | 16.2637 | 14.0168 | 30.0329 | 14.5656 | 30.0332 | 14.5653 | 71.07 | 16.2866pixels/mm |
| 37382 | *Hylobates* | Rm1 | SoFr | M | 19.1649 | 16.7005 | 40.2816 | 21.2606 | 40.6986 | 20.8436 | 68.66 | 18.8324pixels/mm |
| 37382 | *Hylobates* | Rm2 | SoFr | M | 21.5192 | 18.9308 | 46.22 | 29.142 | 46.8976 | 28.4644 | 68.66 | 18.8324pixels/mm |
| 37382 | *Hylobates* | Rm3 | SoFr | M | 17.1348 | 15.3189 | 39.5728 | 14.4406 | 39.8206 | 14.1928 | 68.66 | 18.8324pixels/mm |
| 37385 | *Hylobates* | Rm1 | SoFr | M | 22.0803 | 19.4215 | 57.6824 | 26.8414 | 58.391 | 26.1329 | 67.49 | 18.8324pixels/mm |
| 37385 | *Hylobates* | Rm2 | SoFr | M | 19.5765 | 17.8368 | 50.6582 | 18.9308 | 50.8212 | 18.7679 | 67.49 | 18.8324pixels/mm |
| 37385 | *Hylobates* | Rm3 | SoFr | M | 13.6864 | 11.5801 | 22.2831 | 9.858 | 22.5904 | 9.5507 | 67.49 | 18.8324pixels/mm |
| 41414 | *Hylobates* | Rm1 | SoFr | F | 12.9994 | 9.1122 | 17.9316 | 8.91 | 18.117 | 8.7245 | 65.74 | 14.6212pixels/mm |
| 41414 | *Hylobates* | Rm2 | SoFr | F | 13.7887 | 11.1234 | 21.638 | 10.2648 | 21.7288 | 10.1739 | 65.74 | 14.2602pixels/mm |
| 41414 | *Hylobates* | Rm3 | SoFr | F | 13.8424 | 10.0145 | 21.6024 | 9.7696 | 21.6143 | 9.7577 | 65.74 | 14.5016pixels/mm |
| 41421 | *Hylobates* | Rm1 | SoFr | F | 15.3283 | 13.1255 | 23.4488 | 14.6585 | 23.5077 | 14.5995 | 67.52 | 14.0038pixels/mm |
| 41421 | *Hylobates* | Rm2 | SoFr | F | 16.9897 | 13.5862 | 26.7609 | 19.2908 | 27.06 | 18.9917 | 67.52 | 14.7154pixels/mm |
| 41421 | *Hylobates* | Rm3 | SoFr | F | 13.9453 | 11.7618 | 18.7241 | 12.9269 | 18.8365 | 12.8145 | 67.52 | 14.1471pixels/mm |
| 41428 | *Hylobates* | Rm1 | SoFr | M | 14.9216 | 11.7133 | 23.7344 | 13.0676 | 24.3722 | 12.4297 | 69.94 | 16.2866pixels/mm |
| 41428 | *Hylobates* | Rm2 | SoFr | M | 16.9687 | 12.7463 | 29.7051 | 16.4832 | 29.7405 | 16.4478 | 69.94 | 16.2866pixels/mm |
| 41428 | *Hylobates* | Rm3 | SoFr | M | 15.4607 | 12.3505 | 26.5702 | 13.8788 | 27.9258 | 12.5232 | 69.94 | 16.2866pixels/mm |

| | | | | | | | | | | | | |
|---|---|---|---|---|---|---|---|---|---|---|---|---|
| 41460 | *Hylobates* | Rm1 | SoFr | F | 16.3119 | 10.7599 | 24.586 | 15.3044 | 24.9592 | 14.9312 | 72.59 | 13.0947pixels/mm |
| 41460 | *Hylobates* | Rm2 | SoFr | F | 19.6617 | 13.0959 | 32.6138 | 24.444 | 33.8805 | 23.1773 | 72.59 | 12.9435pixels/mm |
| 41460 | *Hylobates* | Rm3 | SoFr | F | 16.1193 | 12.6381 | 23.0973 | 17.3662 | 24.0842 | 16.3792 | 72.59 | 13.3991pixels/mm |
| 41463 | *Hylobates* | Rm1 | SoFr | F | 12.5508 | 10.5286 | 13.7658 | 11.3986 | 15.0435 | 10.1209 | 69.38 | 13.0426pixels/mm |
| 41463 | *Hylobates* | Rm2 | SoFr | F | 15.6494 | 14.3082 | 24.6331 | 16.0023 | 24.8362 | 15.7992 | 69.38 | 13.3211pixels/mm |
| 41463 | *Hylobates* | Rm3 | SoFr | F | 18.3134 | 15.4635 | 33.7498 | 20.6298 | 34.3923 | 19.9873 | 69.38 | 13.0587pixels/mm |
| 41493 | *Hylobates* | Rm1 | SoFr | F | 14.5606 | 10.6438 | 21.8692 | 12.2387 | 22.0082 | 12.0997 | 72.7 | 12.7527pixels/mm |
| 41493 | *Hylobates* | Rm2 | SoFr | F | 22.4213 | 18.1067 | 45.5528 | 32.5061 | 45.7368 | 32.3221 | 72.7 | 13.3417pixels/mm |
| 41493 | *Hylobates* | Rm3 | SoFr | F | 18.169 | 14.7327 | 32.4466 | 21.1578 | 33.251 | 20.3534 | 72.7 | 13.0811pixels/mm |
| 19976 | *Miopithecus* | Rm1 | Omni | M | 10.4175 | 9.3596 | 11.2752 | 7.1985 | 11.5985 | 6.8752 | 47.22 | 17.7955pixels/mm |
| 19976 | *Miopithecus* | Rm2 | Omni | M | 11.9 | 10.9796 | 12.4639 | 10.957 | 13.0593 | 10.3615 | 47.22 | 19.2201pixels/mm |
| 19976 | *Miopithecus* | Rm3 | Omni | M | 7.3421 | 6.4713 | 5.4591 | 3.5682 | 5.5305 | 3.4968 | 47.22 | 19.8468pixels/mm |
| 23196 | *Miopithecus* | Rm1 | Omni | M | 7.2676 | 6.2159 | 6.6168 | 2.9381 | 6.817 | 2.738 | 46.52 | 16.6055pixels/mm |
| 23196 | *Miopithecus* | Rm2 | Omni | M | 8.8285 | 7.3963 | 7.8609 | 5.0467 | 7.9358 | 4.9719 | 46.52 | 18.7959pixels/mm |
| 23196 | *Miopithecus* | Rm3 | Omni | M | 6.0286 | 5.1195 | 4.1471 | 2.1008 | 4.168 | 2.0799 | 46.52 | 19.5366pixels/mm |
| 23197 | *Miopithecus* | Rm1 | Omni | M | 9.4125 | 7.8339 | 10.657 | 4.6491 | 10.7978 | 4.5084 | 45.19 | 14.8476pixels/mm |
| 23197 | *Miopithecus* | Rm2 | Omni | M | 9.0371 | 7.6752 | 8.5981 | 5.1125 | 8.6859 | 5.0247 | 45.19 | 17.7274pixels/mm |
| 23197 | *Miopithecus* | Rm3 | Omni | M | 7.426 | 6.414 | 5.5503 | 3.5714 | 5.6548 | 3.467 | 45.19 | 19.7309pixels/mm |
| 34264 | *Miopithecus* | Rm1 | Omni | F | 6.5758 | 5.4275 | 5.2762 | 2.3534 | 5.3548 | 2.2748 | 40.25 | 16.9265pixels/mm |
| 34264 | *Miopithecus* | Rm2 | Omni | F | 7.2584 | 5.8722 | 5.0281 | 3.5565 | 5.211 | 3.3735 | 40.25 | 19.1435pixels/mm |
| 34264 | *Miopithecus* | Rm3 | Omni | F | 5.2213 | 4.4239 | 2.7672 | 1.7488 | 2.8173 | 1.6988 | 40.25 | 18.4689pixels/mm |
| 37278 | *Miopithecus* | Rm1 | Omni | F | 7.1396 | 6.103 | 4.7455 | 3.6367 | 4.7792 | 3.6029 | 46.02 | 18.7649pixels/mm |
| 37278 | *Miopithecus* | Rm2 | Omni | F | 9.36 | 8.1065 | 7.9538 | 6.1976 | 8.0135 | 6.1379 | 46.02 | 18.9466pixels/mm |
| 37278 | *Miopithecus* | Rm3 | Omni | F | 6.228 | 5.4598 | 3.956 | 2.5677 | 4.0602 | 2.4635 | 46.02 | 19.6959pixels/mm |
| 15312 | *Pan* | Rm1 | SoFr | F | 44.3699 | 35.7529 | 157.6244 | 154.9569 | 170.7498 | 141.8316 | 128.35 | 9.4591pixels/mm |
| 15312 | *Pan* | Rm2 | SoFr | F | 54.1597 | 41.9126 | 265.1383 | 194.6442 | 265.5754 | 194.207 | 128.35 | 9.503pixels/mm |
| 15312 | *Pan* | Rm3 | SoFr | F | 50.7462 | 39.2154 | 248.255 | 158.3497 | 250.0568 | 156.5479 | 128.35 | 8.6512pixels/mm |
| 17702 | *Pan* | Rm1 | SoFr | F | 61.7691 | 52.3602 | 291.2835 | 322.2995 | 323.9267 | 289.6563 | 130.48 | 12.2677pixels/mm |
| 17702 | *Pan* | Rm2 | SoFr | F | 64.7219 | 55.0433 | 333.6239 | 333.3505 | 350.5627 | 316.4118 | 130.48 | 12.7201pixels/mm |

| | | | | | | | | | | | | |
|---|---|---|---|---|---|---|---|---|---|---|---|---|
| 17702 | *Pan* | Rm3 | SoFr | F | 53.2139 | 46.2442 | 221.179 | 230.9638 | 238.3069 | 213.8359 | 130.48 | 12.6653pixels/mm |
| 19187 | *Pan* | Rm1 | SoFr | M | 72.1048 | 58.4152 | 427.5702 | 397.8186 | 444.3161 | 381.0727 | 134.29 | 10.4712pixels/mm |
| 19187 | *Pan* | Rm2 | SoFr | M | 77.1574 | 59.8836 | 475.1533 | 446.7502 | 480.8245 | 441.0789 | 134.29 | 10.4712pixels/mm |
| 19187 | *Pan* | Rm3 | SoFr | M | 57.3208 | 44.0964 | 285.7532 | 228.5935 | 286.5434 | 227.8033 | 134.29 | 10.4712pixels/mm |
| 20041 | *Pan* | Rm1 | SoFr | M | 67.0982 | 55.4096 | 416.9286 | 304.2435 | 426.4363 | 294.7357 | 146.68 | 10.4932pixels/mm |
| 20041 | *Pan* | Rm3 | SoFr | M | 56.2815 | 42.6221 | 256.4734 | 226.9731 | 265.6342 | 217.8123 | 146.68 | 10.4932pixels/mm |
| 23163 | *Pan* | Rm2 | SoFr | M | 67.3378 | 55.727 | 419.1243 | 300.635 | 419.1606 | 300.5986 | 137.14 | 10.8108pixels/mm |
| 23163 | *Pan* | Rm3 | SoFr | M | 56.8478 | 44.9033 | 294.0646 | 216.1577 | 294.4103 | 215.8119 | 137.14 | 10.8108pixels/mm |
| 23164 | *Pan* | Rm1 | SoFr | M | 54.9344 | 42.4294 | 258.5207 | 207.5106 | 258.5647 | 207.4665 | 134.79 | 10.4822pixels/mm |
| 23164 | *Pan* | Rm3 | SoFr | M | 47.4824 | 35.0134 | 216.8785 | 133.8427 | 217.1057 | 133.6156 | 134.79 | 10.482pixels/mm |
| 23167 | *Pan* | Rm1 | SoFr | F | 64.5235 | 55.4771 | 330.6298 | 336.32 | 361.6999 | 305.2499 | 126.05 | 9.1778pixels/mm |
| 23167 | *Pan* | Rm2 | SoFr | F | 72.0129 | 59.1359 | 405.0184 | 404.2516 | 419.1832 | 390.0868 | 126.05 | 9.1454pixels/mm |
| 23167 | *Pan* | Rm3 | SoFr | F | 60.8292 | 50.2891 | 299.0444 | 278.8117 | 300.8205 | 277.0356 | 126.05 | 9.1529pixels/mm |
| 167342 | *Pan* | Rm1 | SoFr | M | 52.2326 | 44.3802 | 204.5817 | 232.6594 | 236.4855 | 200.7556 | 145.61 | 10.9528pixels/mm |
| 167342 | *Pan* | Rm2 | SoFr | M | 69.3553 | 55.6185 | 382.3993 | 370.1554 | 387.3175 | 365.2372 | 145.61 | 11.1212pixels/mm |
| 167342 | *Pan* | Rm3 | SoFr | M | 76.8563 | 60.0638 | 493.8831 | 419.4626 | 504.7524 | 408.5933 | 145.61 | 11.0839pixels/mm |
| BOM9493 | *Pan* | Rm1 | SoFr | F | 54.5416 | 45.851 | 199.2295 | 278.6432 | 290.8351 | 187.0376 | 125.67 | 8.994pixels/mm |
| BOM9493 | *Pan* | Rm2 | SoFr | F | 55.2492 | 46.2271 | 240.1843 | 236.3802 | 251.8089 | 224.7556 | 125.67 | 8.9767pixels/mm |
| BOM9493 | *Pan* | Rm3 | SoFr | F | 43.3789 | 35.4611 | 158.3599 | 136.0264 | 168.7107 | 125.6757 | 125.67 | 8.7629pixels/mm |
| 8304 | *Papio* | Rm1 | Omni | M | 66.5355 | 52.4495 | 400.2301 | 296.7193 | 406.8886 | 290.0607 | 147.35 | 11.5773pixels/mm |
| 8304 | *Papio* | Rm2 | Omni | M | 118.8592 | 84.4772 | 1091.3353 | 998.1239 | 1141.0002 | 948.4591 | 147.35 | 11.31pixels/mm |
| 17342 | *Papio* | Rm1 | Omni | M | 69.2664 | 58.8223 | 496.0434 | 290.4113 | 496.1083 | 290.3465 | 133.96 | 11.0835pixels/mm |
| 17342 | *Papio* | Rm2 | Omni | M | 103.7978 | 82.613 | 943.899 | 724.9006 | 944.485 | 724.3147 | 133.96 | 11.1632pixels/mm |
| 21160 | *Papio* | Rm1 | Omni | M | 70.3825 | 55.2212 | 493.3215 | 316.6453 | 494.8714 | 315.0954 | 147.83 | 11.6649pixels/mm |
| 21160 | *Papio* | Rm2 | Omni | M | 95.6947 | 76.0991 | 890.2314 | 592.5691 | 891.0081 | 591.7924 | 147.83 | 11.8572pixels/mm |
| 21160 | *Papio* | Rm3 | Omni | M | 119.3769 | 95.8644 | 2141.1559 | 721.2017 | 2174.3707 | 687.987 | 147.83 | 11.5966pixels/mm |
| 21161 | *Papio* | Rm1 | Omni | M | 51.7342 | 44.7391 | 306.2799 | 157.8206 | 309.7901 | 154.3104 | 147.55 | 11.3555pixels/mm |
| 21161 | *Papio* | Rm2 | Omni | M | 77.4082 | 64.2643 | 636.0778 | 363.99 | 642.1782 | 357.8895 | 147.55 | 11.3358pixels/mm |
| 21161 | *Papio* | Rm3 | Omni | M | 93.0325 | 75.7007 | 1338.553 | 411.4662 | 1347.0308 | 402.9884 | 147.55 | 11.4871pixels/mm |

| | | | | | | | | | | | | |
|---|---|---|---|---|---|---|---|---|---|---|---|---|
| 23084 | *Papio* | Rm2 | Omni | F | 76.6908 | 68.8596 | 575.0526 | 404.2361 | 593.1807 | 386.1081 | 118.72 | 12.9534pixels/mm |
| 23084 | *Papio* | Rm3 | Omni | F | 93.128 | 85.8928 | 1180.1521 | 459.7751 | 1190.1841 | 449.7431 | 118.72 | 12.9534pixels/mm |
| 29786 | *Papio* | Rm1 | Omni | M | 67.8544 | 57.7409 | 456.3723 | 306.5403 | 458.493 | 304.4196 | 153.29 | 11.8951pixels/mm |
| 29786 | *Papio* | Rm2 | Omni | M | 103.0718 | 88.7272 | 885.9902 | 771.9908 | 899.9684 | 758.0126 | 153.29 | 12.0156pixels/mm |
| 29786 | *Papio* | Rm3 | Omni | M | 126.2149 | 106.4236 | 2141.6115 | 778.5499 | 2143.8103 | 776.3511 | 153.29 | 12.0317pixels/mm |
| 37362 | *Pongo* | Rm1 | HoFr | M | 100.7365 | 82.1533 | 780.2859 | 821.8816 | 892.6377 | 709.5299 | 171.78 | 8.8028pixels/mm |
| 37362 | *Pongo* | Rm2 | HoFr | M | 127.7467 | 99.717 | 1273.9357 | 1245.0814 | 1324.9608 | 1194.0562 | 171.78 | 8.8028pixels/mm |
| 37362 | *Pongo* | Rm3 | HoFr | M | 128.0822 | 98.362 | 1391.5361 | 1142.3201 | 1391.7551 | 1142.101 | 171.78 | 8.8028pixels/mm |
| 37363 | *Pongo* | Rm1 | HoFr | F | 87.668 | 77.8435 | 644.4103 | 577.0731 | 697.9965 | 523.4869 | 147.74 | 9.0744pixels/mm |
| 37363 | *Pongo* | Rm2 | HoFr | F | 88.2874 | 76.3012 | 696.2663 | 549.2708 | 709.2595 | 536.2776 | 147.74 | 9.0744pixels/mm |
| 37363 | *Pongo* | Rm3 | HoFr | F | 75.2204 | 65.9544 | 557.7138 | 360.4688 | 557.7235 | 360.4591 | 147.74 | 9.0744pixels/mm |
| 37365 | *Pongo* | Rm1 | HoFr | F | 95.5531 | 79.9879 | 771.7852 | 666.0916 | 791.0295 | 646.8473 | 155.48 | 9.2507pixels/mm |
| 37365 | *Pongo* | Rm2 | HoFr | F | 95.6583 | 80.876 | 823.0776 | 649.1774 | 823.1744 | 649.0807 | 155.48 | 9.2507pixels/mm |
| 37365 | *Pongo* | Rm3 | HoFr | F | 74.9981 | 66.3041 | 561.0263 | 363.1925 | 565.0901 | 359.1287 | 155.48 | 9.2507pixels/mm |
| 37516 | *Pongo* | Rm1 | HoFr | M | 108.9074 | 81.092 | 951.2308 | 860.1013 | 1018.3296 | 793.0025 | 176.29 | 9.5785pixels/mm |
| 37516 | *Pongo* | Rm2 | HoFr | M | 136.4612 | 102.4985 | 1515.395 | 1325.5228 | 1540.2048 | 1300.7131 | 176.29 | 9.5785pixels/mm |
| 37516 | *Pongo* | Rm3 | HoFr | M | 110.7494 | 85.6371 | 1106.251 | 811.1359 | 1108.4868 | 808.9001 | 176.29 | 9.5785pixels/mm |
| 37517 | *Pongo* | Rm1 | HoFr | M | 108.5081 | 83.3363 | 904.5785 | 912.5476 | 1021.3247 | 795.8015 | 177.85 | 9.4607pixels/mm |
| 37517 | *Pongo* | Rm2 | HoFr | M | 145.3329 | 109.1784 | 1697.5678 | 1539.5513 | 1725.8028 | 1511.3162 | 177.85 | 9.4607pixels/mm |
| 37517 | *Pongo* | Rm3 | HoFr | M | 106.1842 | 85.9953 | 944.4826 | 829.5135 | 988.7382 | 785.258 | 177.85 | 9.4607pixels/mm |
| 37518 | *Pongo* | Rm1 | HoFr | F | 86.4901 | 67.2017 | 651.9695 | 514.2791 | 652.908 | 513.3406 | 147.08 | 11.8226pixels/mm |
| 37518 | *Pongo* | Rm2 | HoFr | F | 116.4559 | 84.2221 | 1060.5206 | 971.4889 | 1067.4861 | 964.5234 | 147.08 | 11.9161pixels/mm |
| 37518 | *Pongo* | Rm3 | HoFr | F | 97.9878 | 74.0137 | 768.1699 | 720.1523 | 792.4445 | 695.8777 | 147.08 | 11.9769pixels/mm |
| 37519 | *Pongo* | Rm1 | HoFr | F | 87.0016 | 75.2531 | 634.9641 | 562.3855 | 650.8614 | 546.4882 | 132.58 | 11.9581pixels/mm |
| 37519 | *Pongo* | Rm2 | HoFr | F | 104.7395 | 91.4795 | 938.9782 | 791.9066 | 955.7452 | 775.1396 | 132.58 | 11.9986pixels/mm |
| 37519 | *Pongo* | Rm3 | HoFr | F | 89.2989 | 76.4012 | 775.3825 | 526.706 | 785.9298 | 516.1586 | 132.58 | 11.7938pixels/mm |
| 50958 | *Pongo* | Rm1 | HoFr | F | 92.3918 | 73.1942 | 731.697 | 593.922 | 747.2243 | 578.3947 | 136.27 | 11.359pixels/mm |
| 50958 | *Pongo* | Rm2 | HoFr | F | 97.898 | 74.2295 | 801.8654 | 657.4531 | 803.3596 | 655.9589 | 136.27 | 11.8668pixels/mm |
| 50958 | *Pongo* | Rm3 | HoFr | F | 73.9357 | 57.9394 | 478.4541 | 373.0279 | 479.5178 | 371.9642 | 136.27 | 11.9335pixels/mm |

| | | | | | | | | | | | | |
|---|---|---|---|---|---|---|---|---|---|---|---|---|
| 50960 | *Pongo* | Rm1 | HoFr | M | 120.0194 | 92.101 | 1173.1047 | 1033.8878 | 1185.069 | 1021.9235 | 172.28 | 8.8183pixels/mm |
| 50960 | *Pongo* | Rm2 | HoFr | M | 132.1204 | 98.4409 | 1366.6537 | 1265.4218 | 1381.179 | 1250.8965 | 172.28 | 8.8183pixels/mm |
| 50960 | *Pongo* | Rm3 | HoFr | M | 117.4861 | 91.831 | 1334.7095 | 852.6201 | 1335.1865 | 852.1431 | 172.28 | 8.8183pixels/mm |
| 36031 | *Symphalangus* | Rm1 | Mixed Fol | M | 41.1161 | 30.7512 | 179.5277 | 92.8456 | 179.62 | 92.7533 | 83.58 | 11.1863pixels/mm |
| 36031 | *Symphalangus* | Rm2 | Mixed Fol | M | 41.6764 | 34.9023 | 172.6529 | 106.1138 | 173.3439 | 105.4228 | 83.58 | 12.4143pixels/mm |
| 36031 | *Symphalangus* | Rm3 | Mixed Fol | M | 34.5996 | 25.4261 | 134.8416 | 59.4103 | 135.2364 | 59.0155 | 83.58 | 11.8078pixels/mm |
| 36032 | *Symphalangus* | Rm1 | Mixed Fol | F | 33.7658 | 27.2827 | 138.8278 | 57.1537 | 138.9883 | 56.9932 | 75.4 | 11.7364pixels/mm |
| 36032 | *Symphalangus* | Rm2 | Mixed Fol | F | 33.4914 | 25.0928 | 121.3912 | 60.7029 | 123.1776 | 58.9165 | 75.4 | 12.4701pixels/mm |
| 102724 | *Symphalangus* | Rm1 | Mixed Fol | M | 22.9623 | 19.0685 | 47.8825 | 36.5966 | 47.9966 | 36.4824 | 87.17 | 15.4878pixels/mm |
| 102724 | *Symphalangus* | Rm2 | Mixed Fol | M | 27.5139 | 22.1063 | 63.3664 | 55.1437 | 63.5936 | 54.9165 | 87.17 | 15.5407pixels/mm |
| 19549 | *Theropithecus* | Rm1 | Fol | M | 43.2761 | 33.5546 | 139.7865 | 147.3444 | 157.1169 | 130.014 | 120 | 11.3348pixels/mm |
| 19549 | *Theropithecus* | Rm2 | Fol | M | 83.3187 | 63.1724 | 642.0984 | 456.9913 | 655.0861 | 444.0036 | 120 | 10.853pixels/mm |
| 23986 | *Theropithecus* | Rm1 | Fol | M | 47.2527 | 39.0569 | 217.7303 | 145.9112 | 224.1317 | 139.5098 | 128.98 | 12.7005pixels/mm |
| 23986 | *Theropithecus* | Rm2 | Fol | M | 70.7276 | 60.4944 | 466.9526 | 361.988 | 480.4352 | 348.5054 | 128.98 | 12.5704pixels/mm |
| 23986 | *Theropithecus* | Rm3 | Fol | M | 78.8518 | 67.5141 | 746.1145 | 365.2247 | 764.5111 | 346.828 | 128.98 | 12.5791pixels/mm |
| 90309 | *Theropithecus* | Rm1 | Fol | M | 41.3489 | 34.5866 | 137.1595 | 138.8856 | 155.2288 | 120.8163 | 124.33 | 10.5943pixels/mm |
| 90309 | *Theropithecus* | Rm2 | Fol | M | 72.1833 | 59.0756 | 467.8966 | 378.5615 | 481.4638 | 364.9944 | 124.33 | 10.1973pixels/mm |
| 238034 | *Theropithecus* | Rm3 | Fol | F | 92.931 | 81.1629 | 1225.5475 | 438.67 | 1249.2396 | 414.9779 | 112.78 | 7.3746pixels/mm |
