# Supplementary figures and images for: Molar Cervical Root Cross‐Sectional Morphology and Diet in Extant Catarrhines (part 2 of 2)
Source: Am J Biol Anthropol. 2025 Nov 18;188(3):e70164. doi: 10.1002/ajpa.70164 (PMC12625805; doi:10.1002/ajpa.70164)

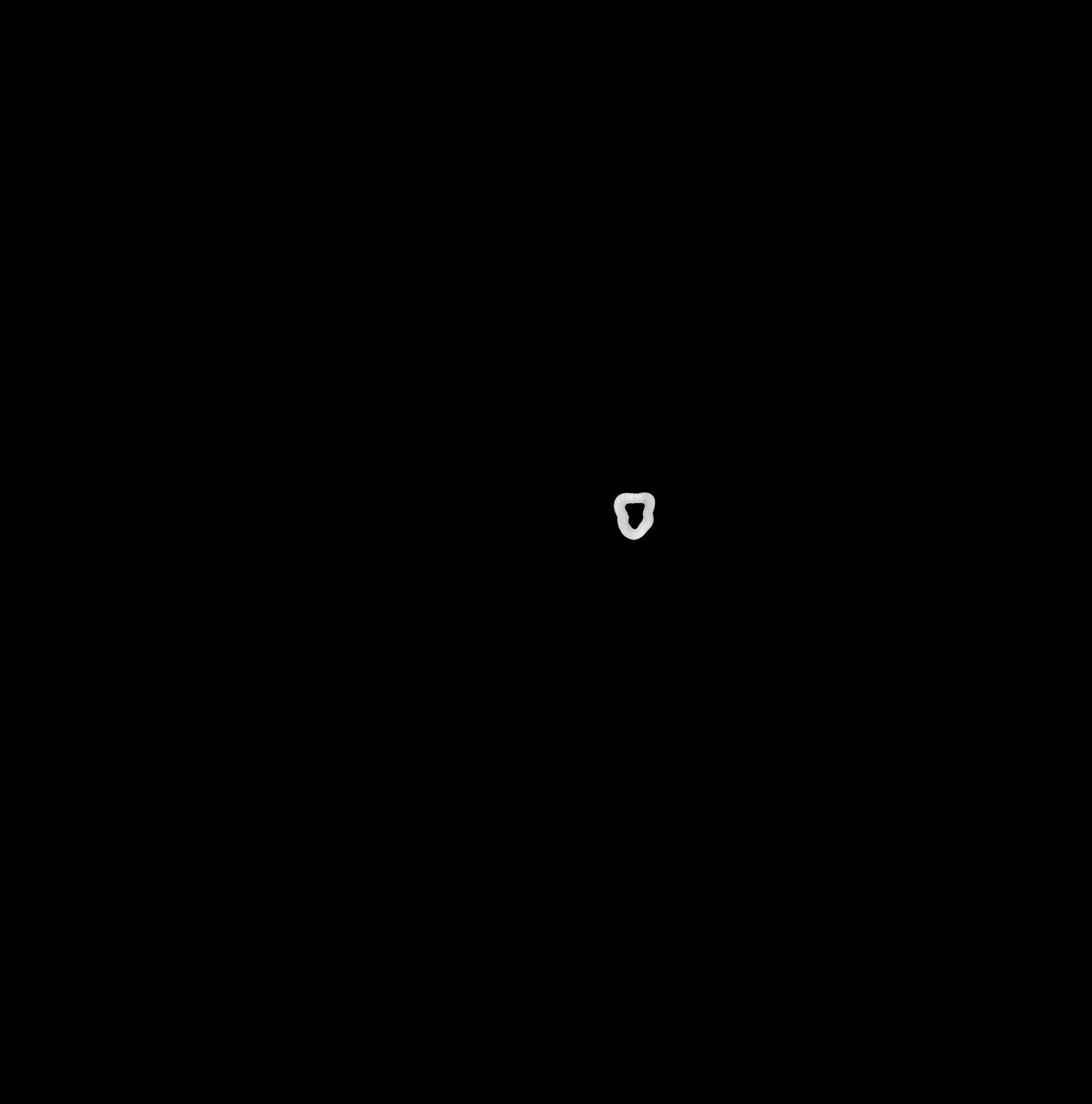

Supplement: Supplementary file 2 — Data S2: Supporting Information. [file AJPA-188-e70164-s001.zip › Cross-Section Tiff Files/mcz_37516_Rm3.tif]

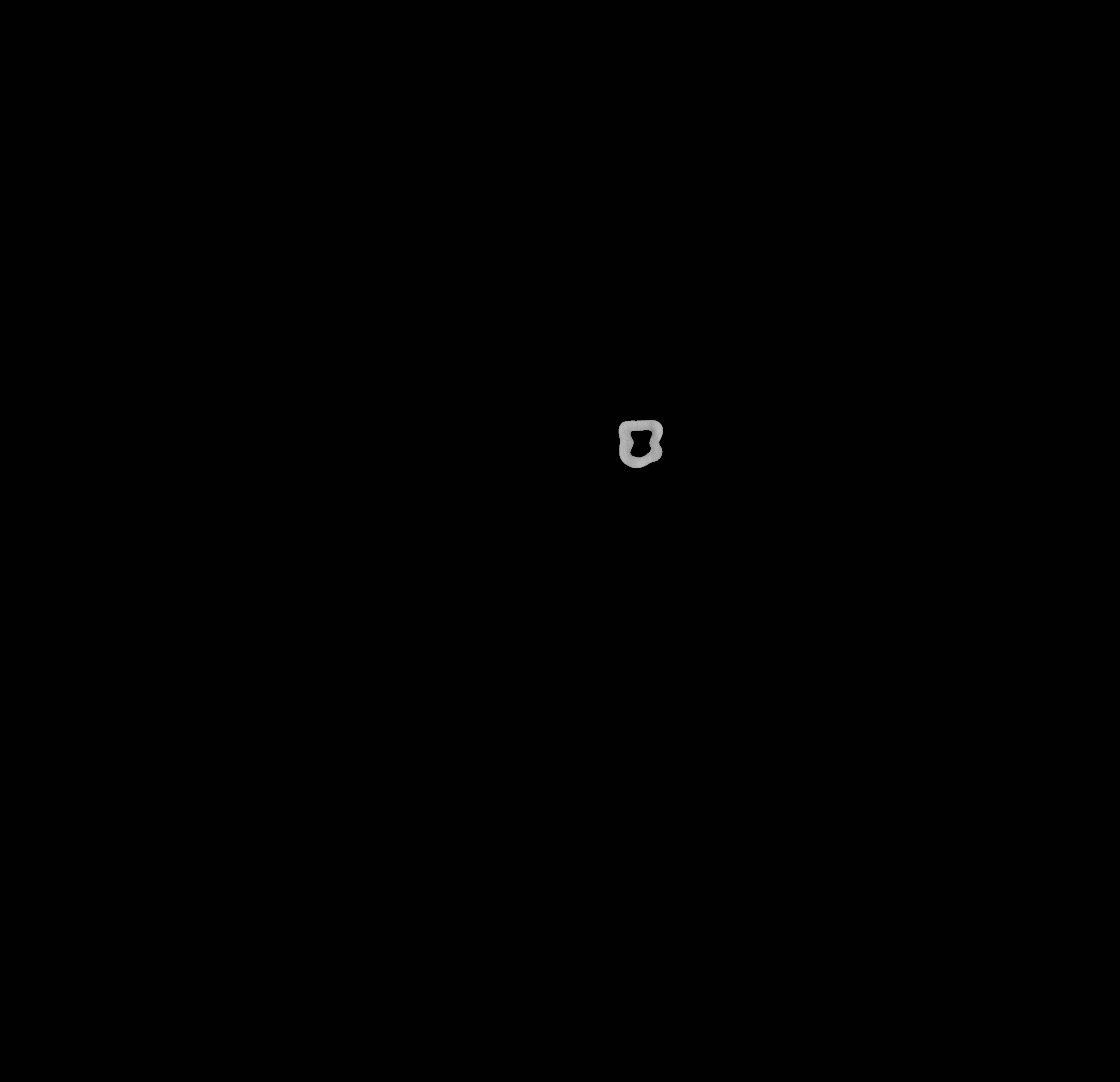

Supplement: Supplementary file 2 — Data S2: Supporting Information. [file AJPA-188-e70164-s001.zip › Cross-Section Tiff Files/mcz_37516_Rm2.tif]

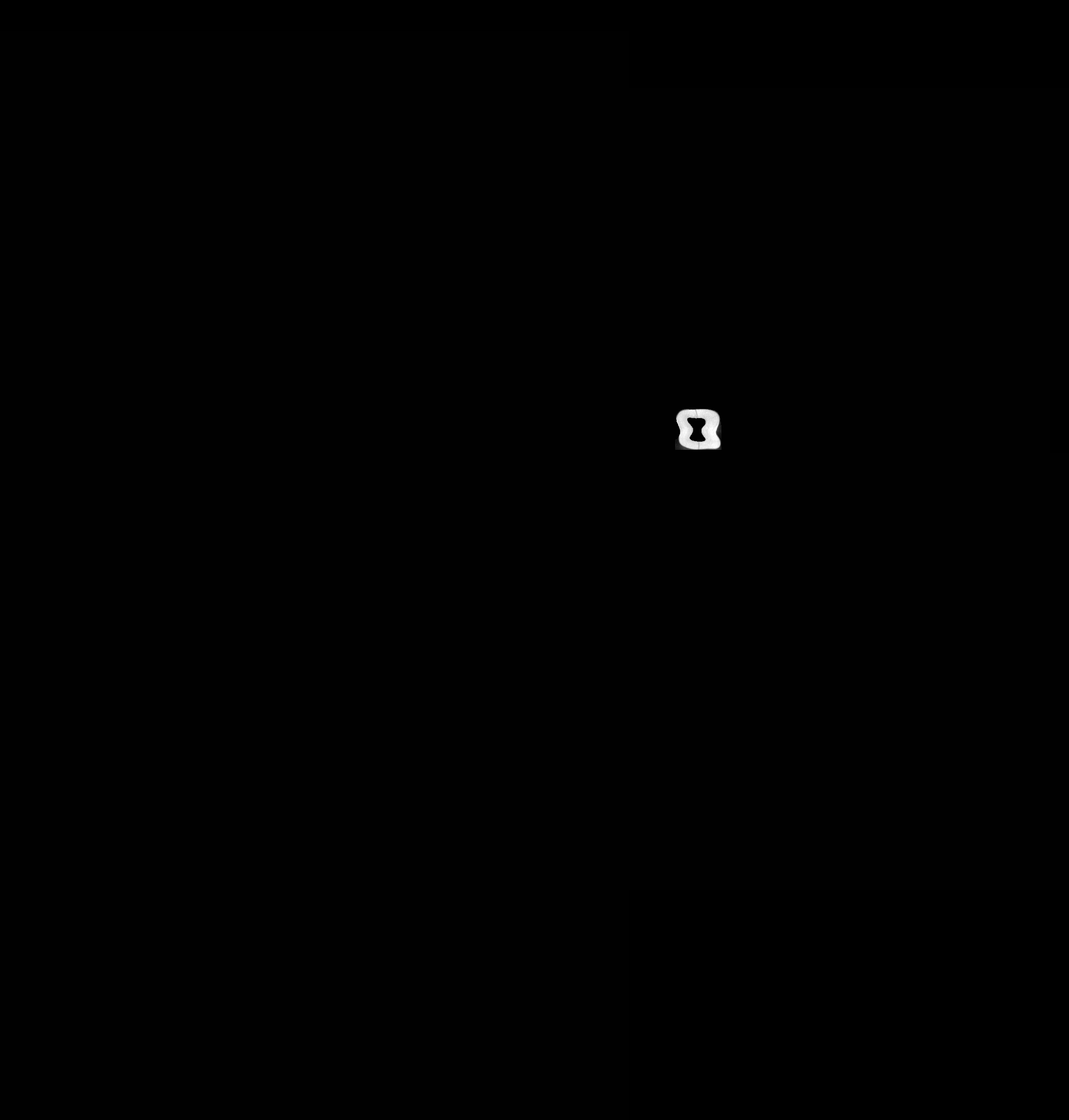

Supplement: Supplementary file 2 — Data S2: Supporting Information. [file AJPA-188-e70164-s001.zip › Cross-Section Tiff Files/amnh_52641_Rm1.tif]

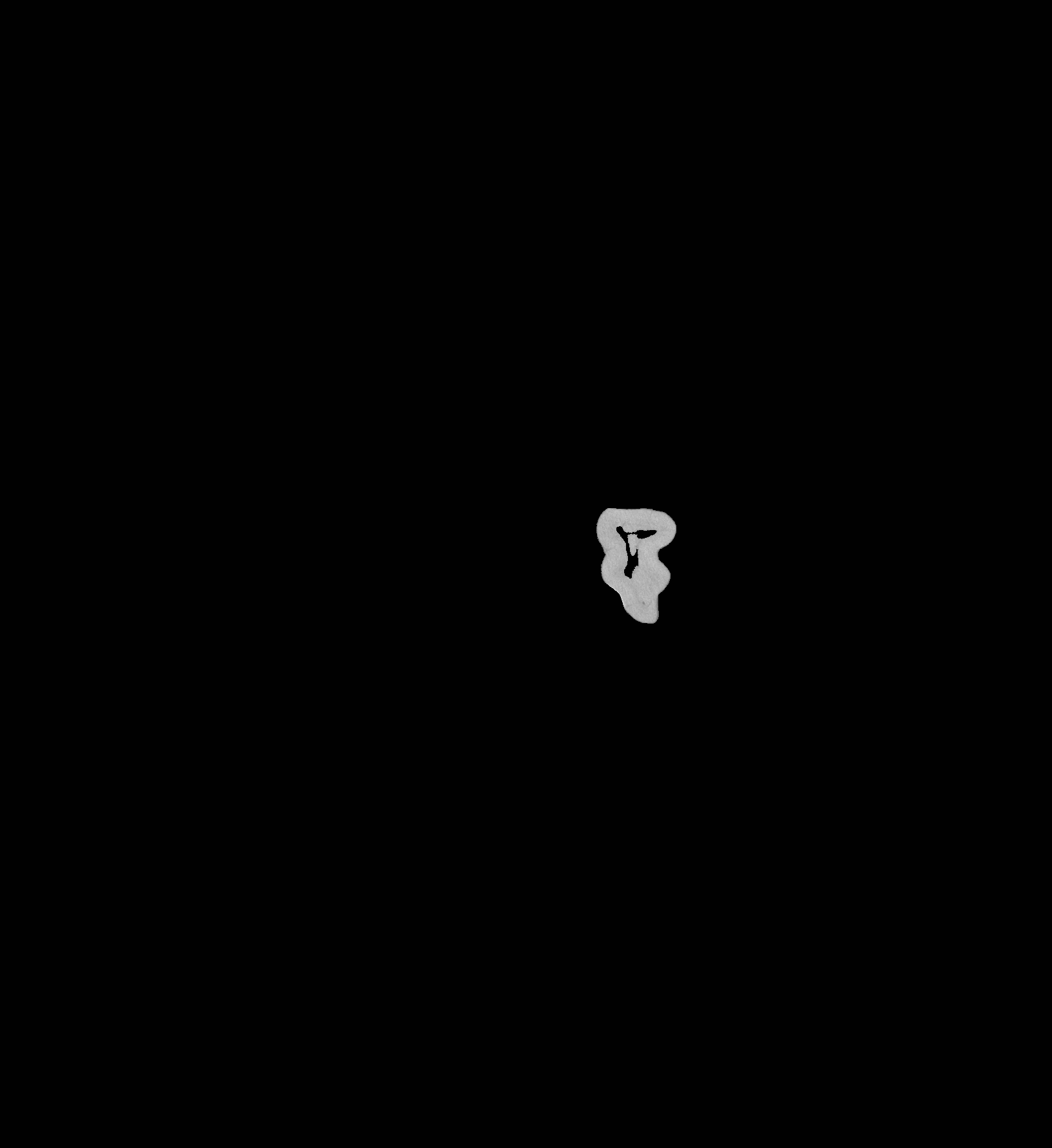

Supplement: Supplementary file 2 — Data S2: Supporting Information. [file AJPA-188-e70164-s001.zip › Cross-Section Tiff Files/mcz_23084_Rm3.tif]

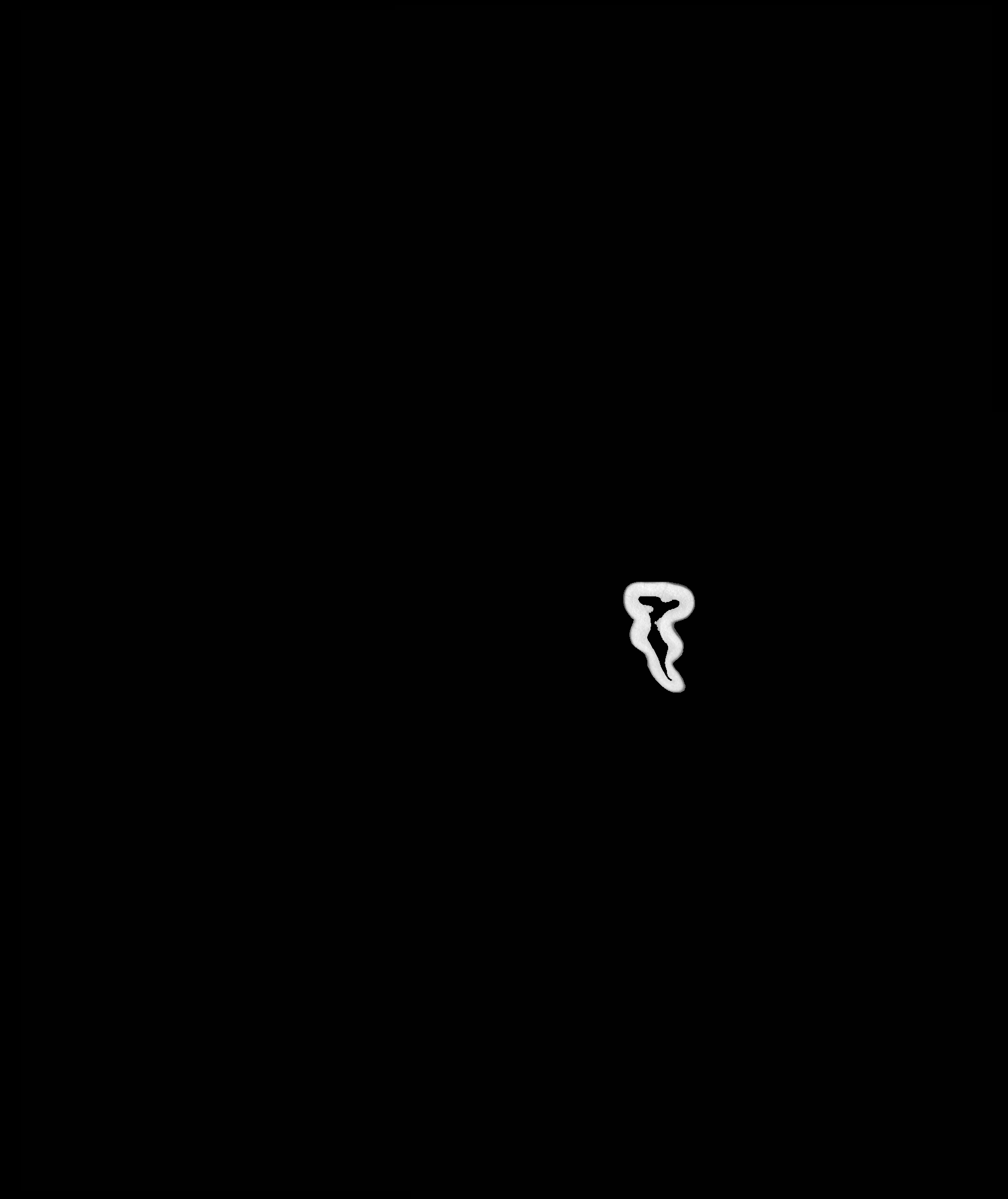

Supplement: Supplementary file 2 — Data S2: Supporting Information. [file AJPA-188-e70164-s001.zip › Cross-Section Tiff Files/mcz_21160_Rm3.tif]

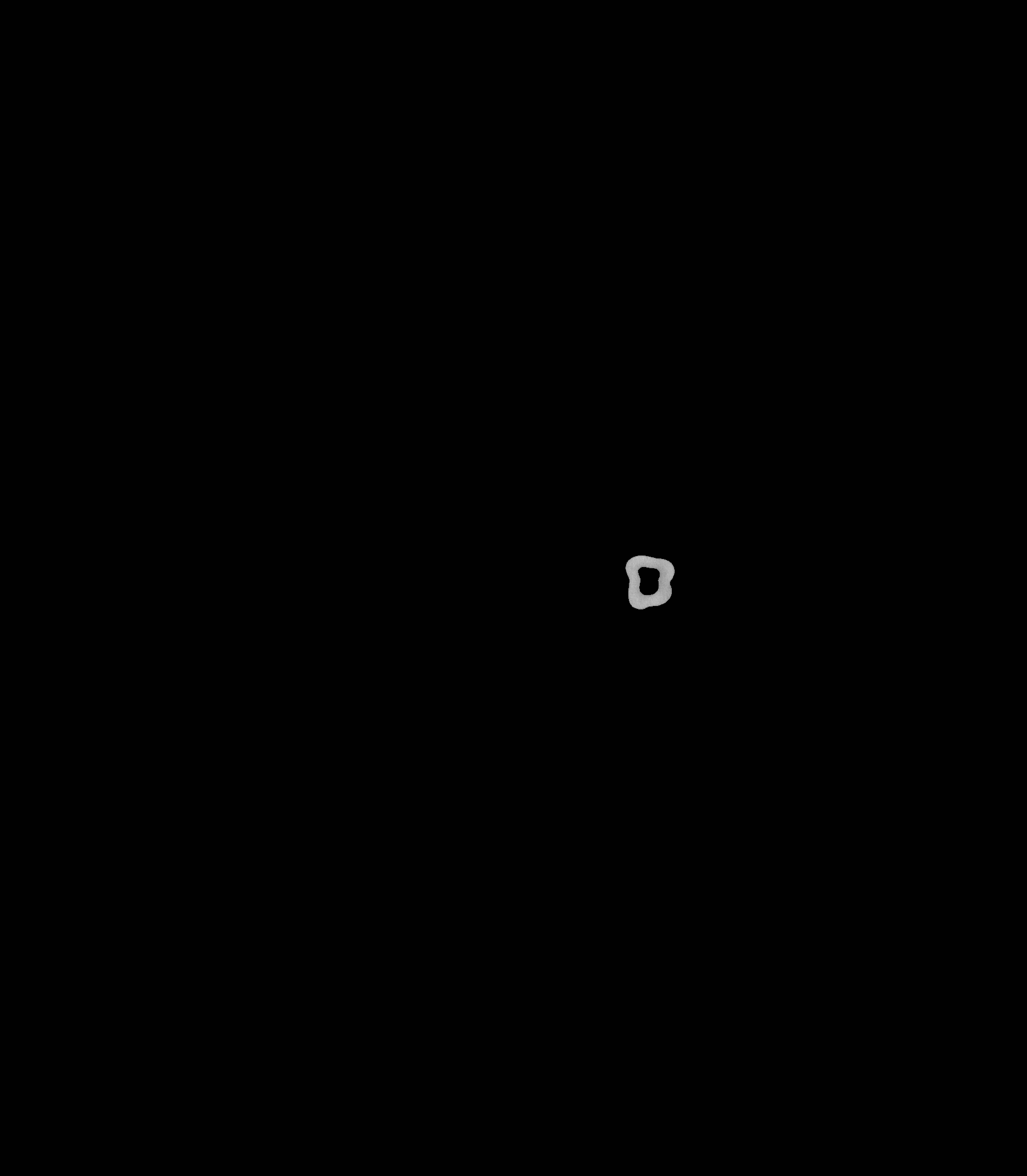

Supplement: Supplementary file 2 — Data S2: Supporting Information. [file AJPA-188-e70164-s001.zip › Cross-Section Tiff Files/mcz_19187_Rm3.tif]

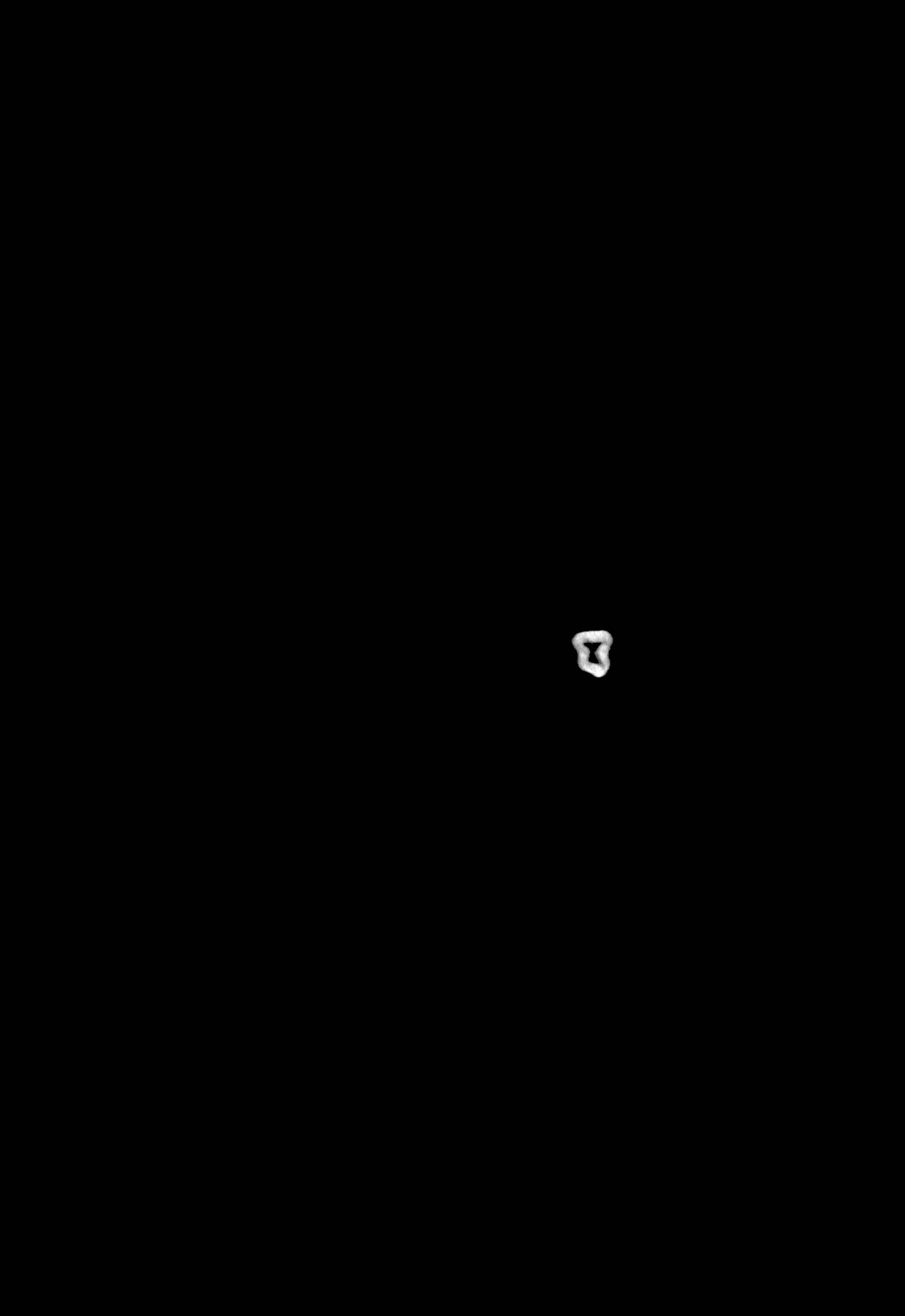

Supplement: Supplementary file 2 — Data S2: Supporting Information. [file AJPA-188-e70164-s001.zip › Cross-Section Tiff Files/mcz_19976_Rm3.tif]

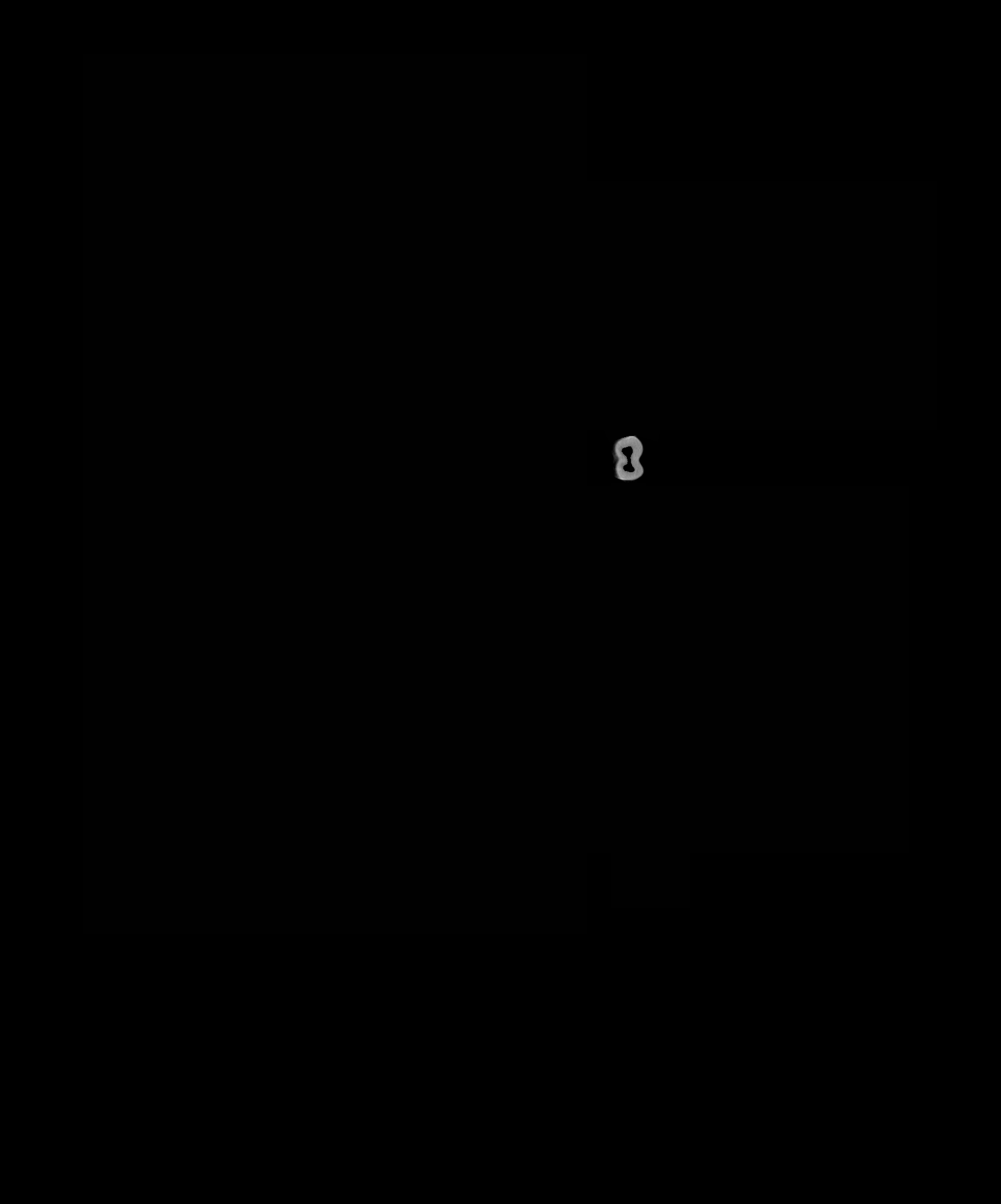

Supplement: Supplementary file 2 — Data S2: Supporting Information. [file AJPA-188-e70164-s001.zip › Cross-Section Tiff Files/mcz_47016_Rm1.tif]

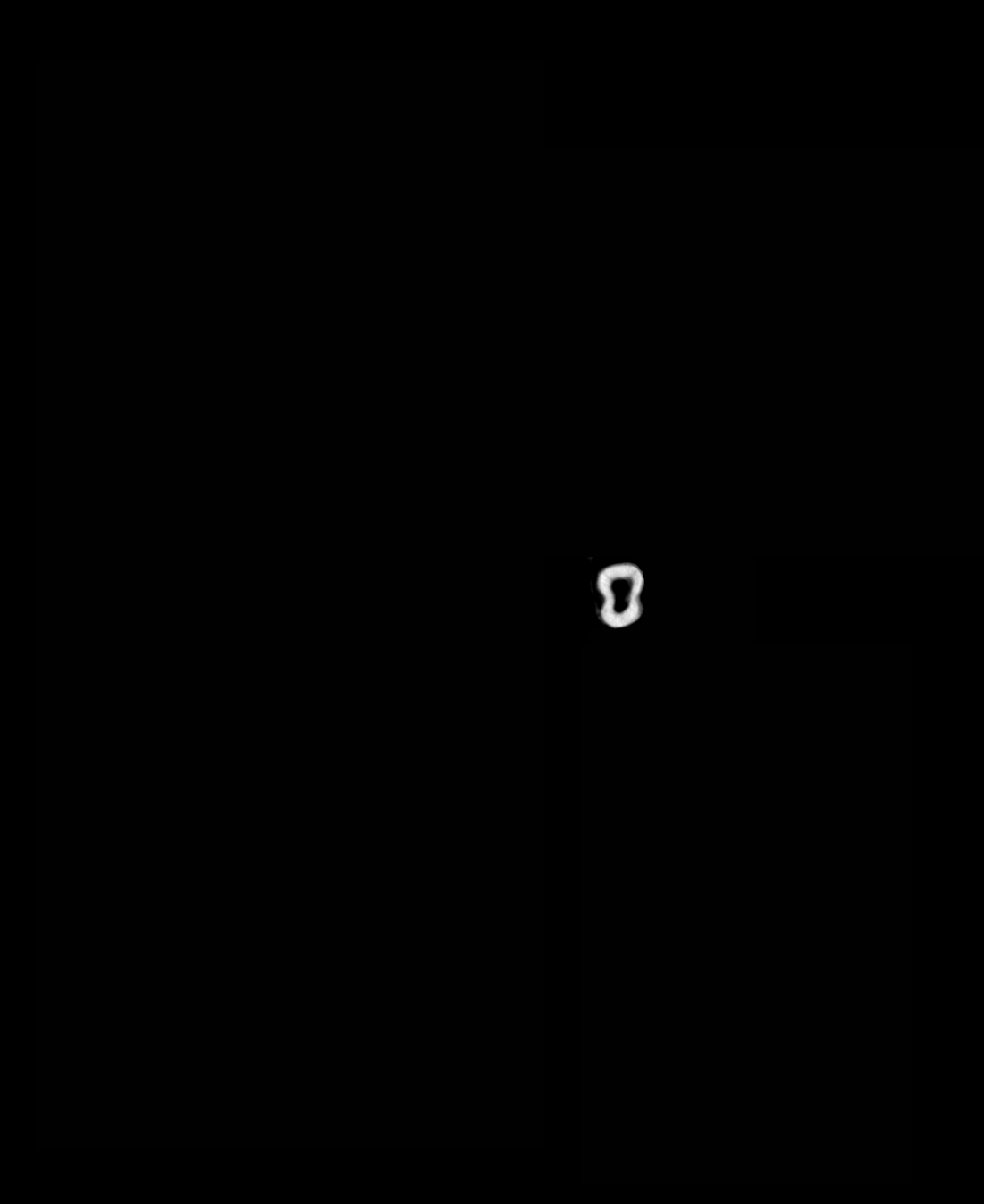

Supplement: Supplementary file 2 — Data S2: Supporting Information. [file AJPA-188-e70164-s001.zip › Cross-Section Tiff Files/mcz_41414_Rm3.tif]

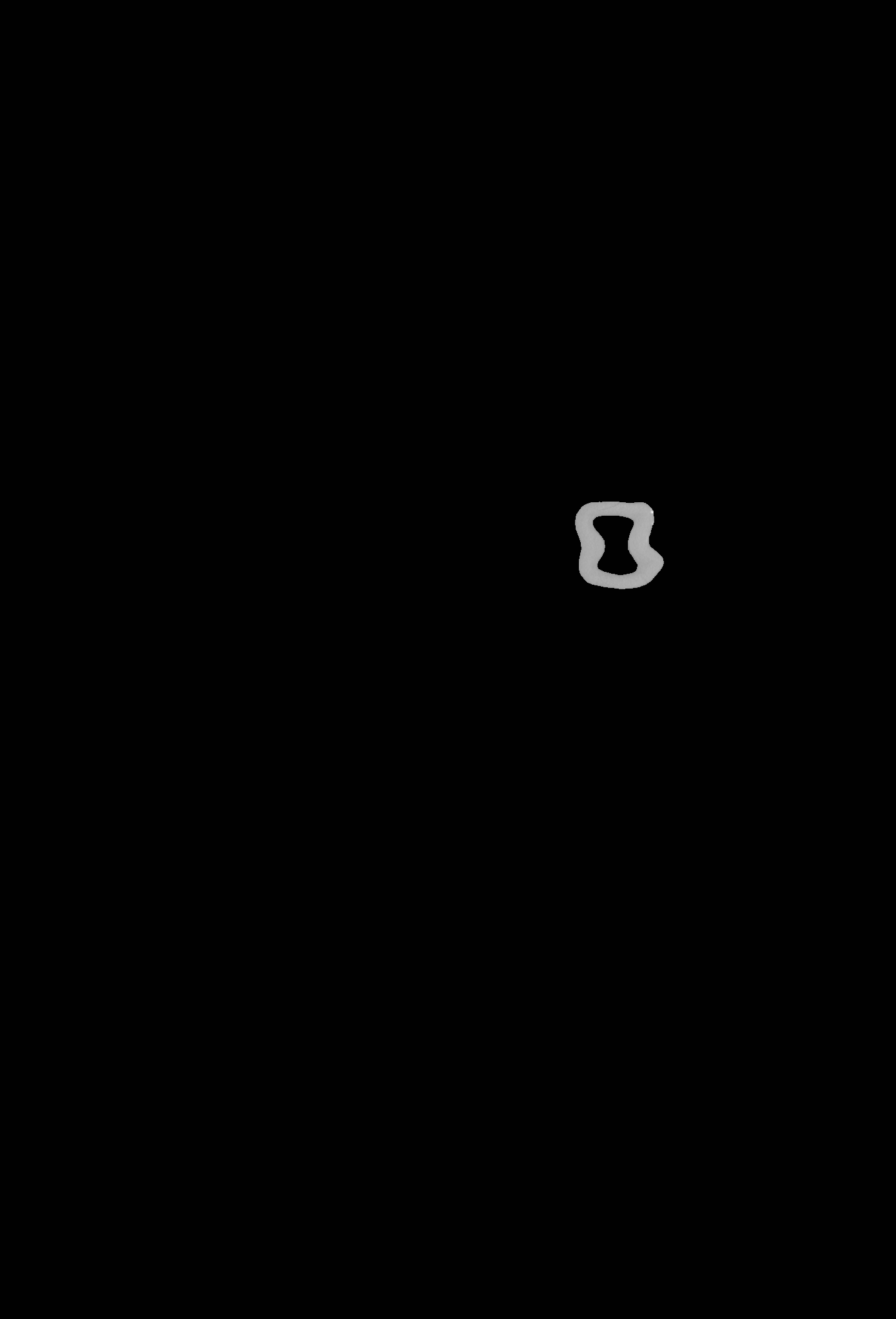

Supplement: Supplementary file 2 — Data S2: Supporting Information. [file AJPA-188-e70164-s001.zip › Cross-Section Tiff Files/mcz_8304_Rm2.tif]

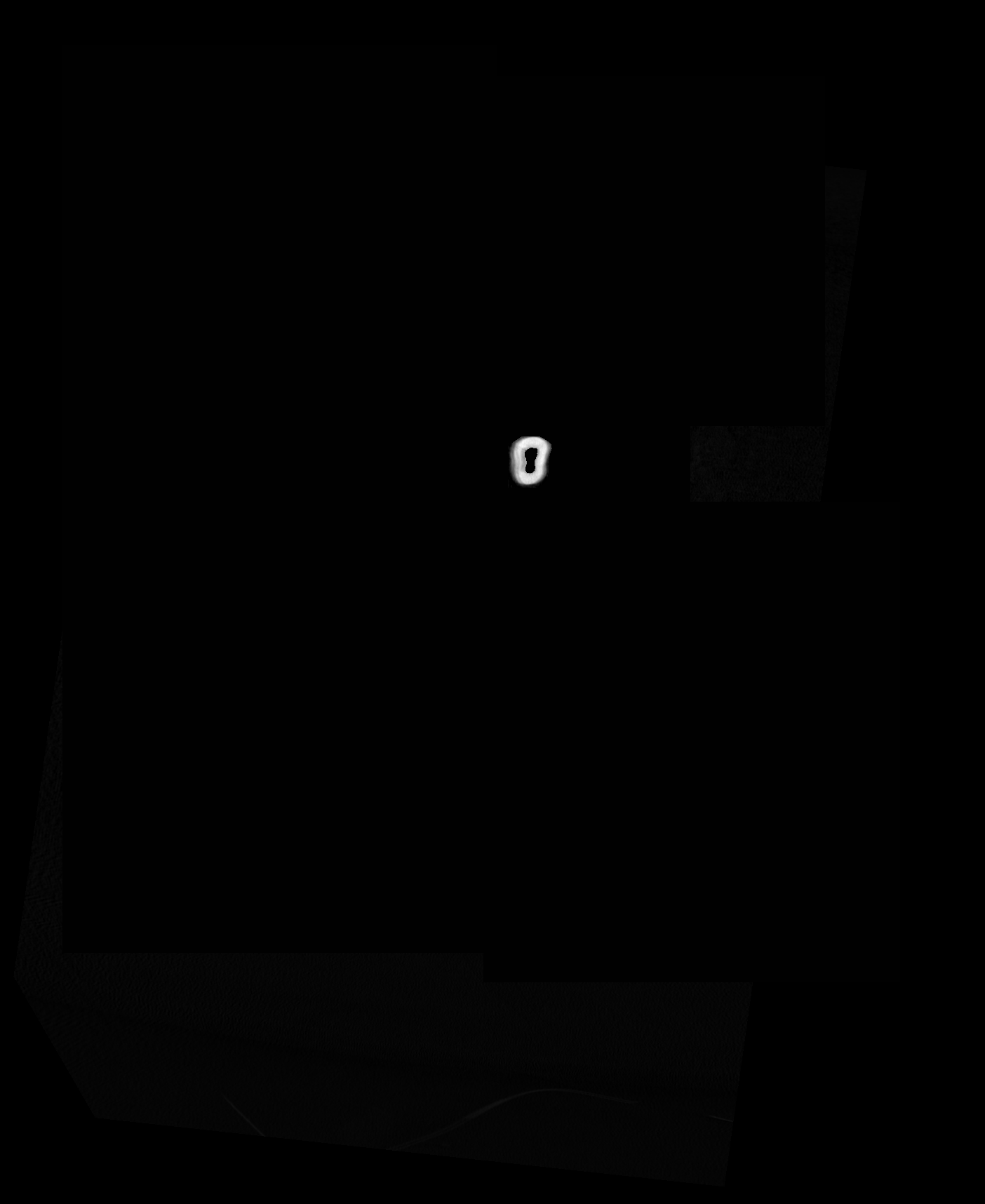

Supplement: Supplementary file 2 — Data S2: Supporting Information. [file AJPA-188-e70164-s001.zip › Cross-Section Tiff Files/mcz_41463_Rm3.tif]

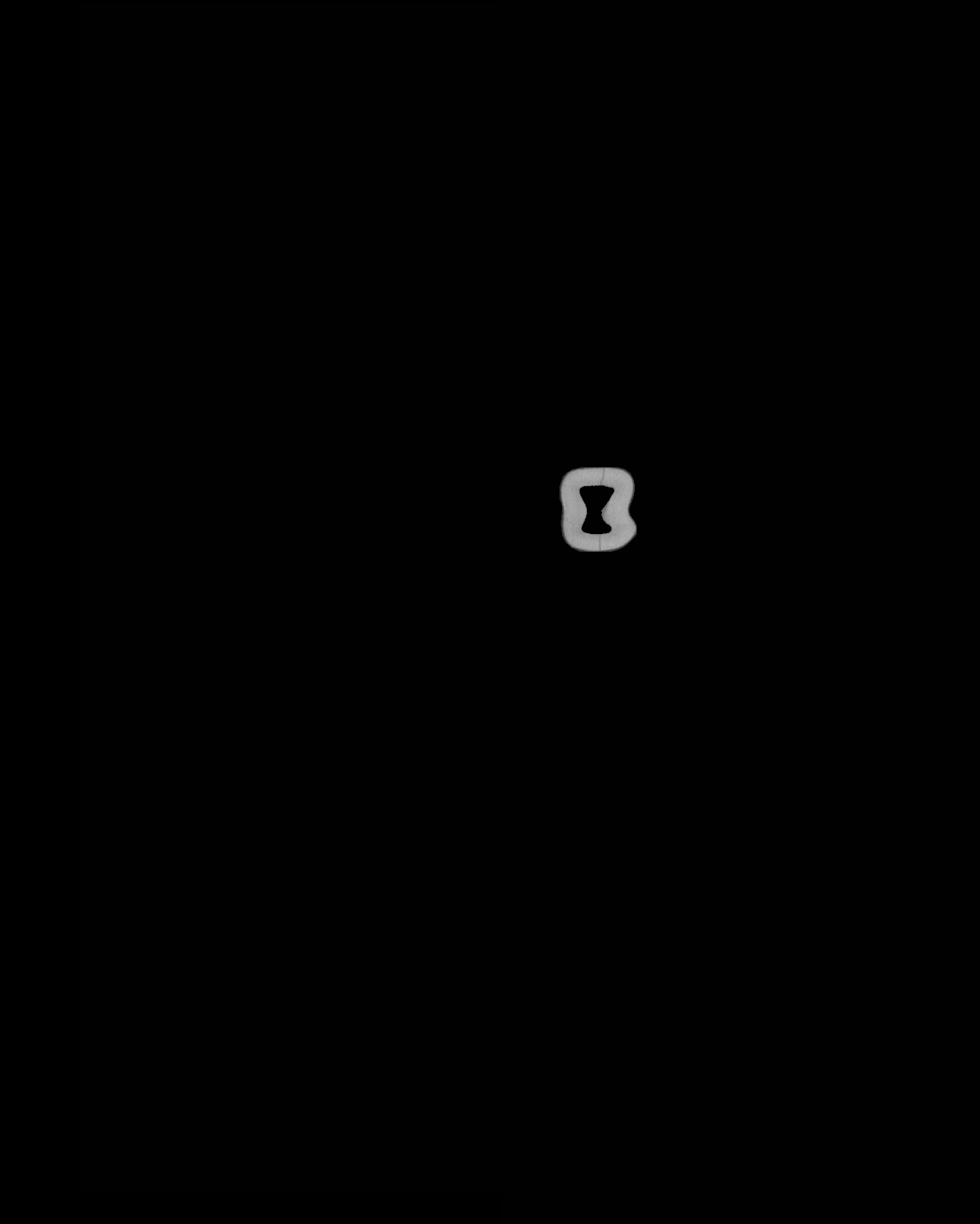

Supplement: Supplementary file 2 — Data S2: Supporting Information. [file AJPA-188-e70164-s001.zip › Cross-Section Tiff Files/mcz_17342_Rm2.tif]

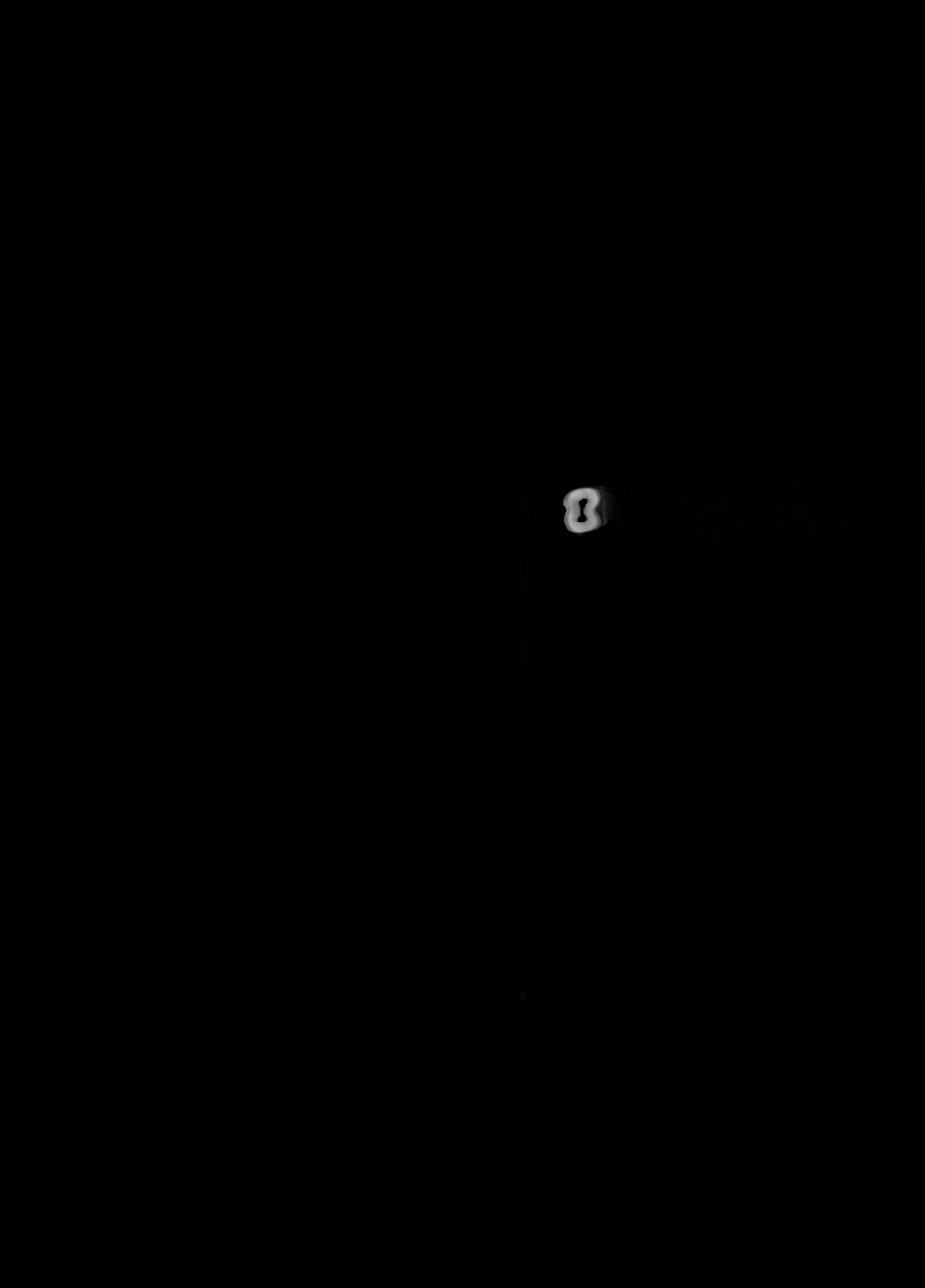

Supplement: Supplementary file 2 — Data S2: Supporting Information. [file AJPA-188-e70164-s001.zip › Cross-Section Tiff Files/mcz_41463_Rm2.tif]

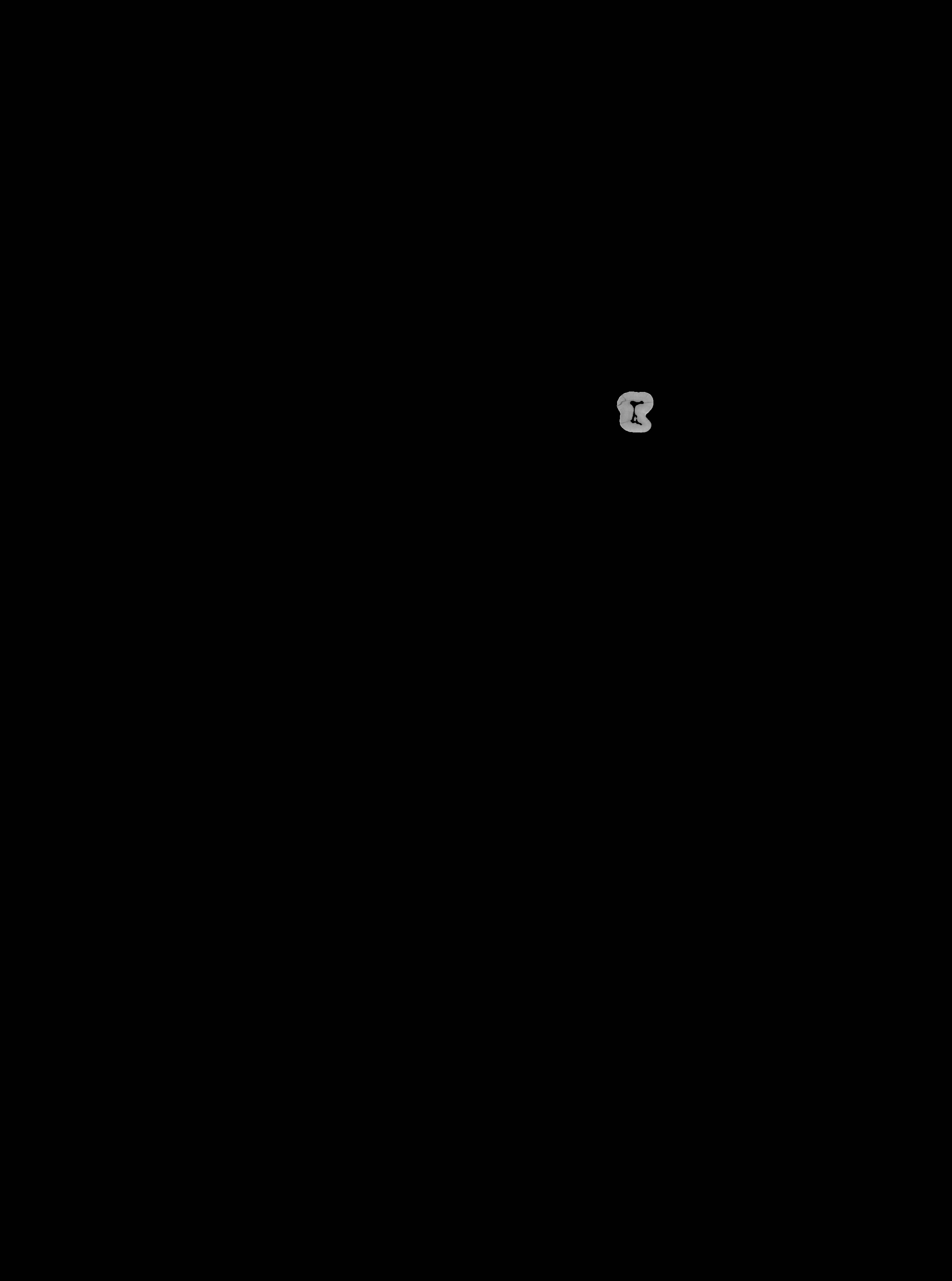

Supplement: Supplementary file 2 — Data S2: Supporting Information. [file AJPA-188-e70164-s001.zip › Cross-Section Tiff Files/amnh_34714_Rm2.tif]

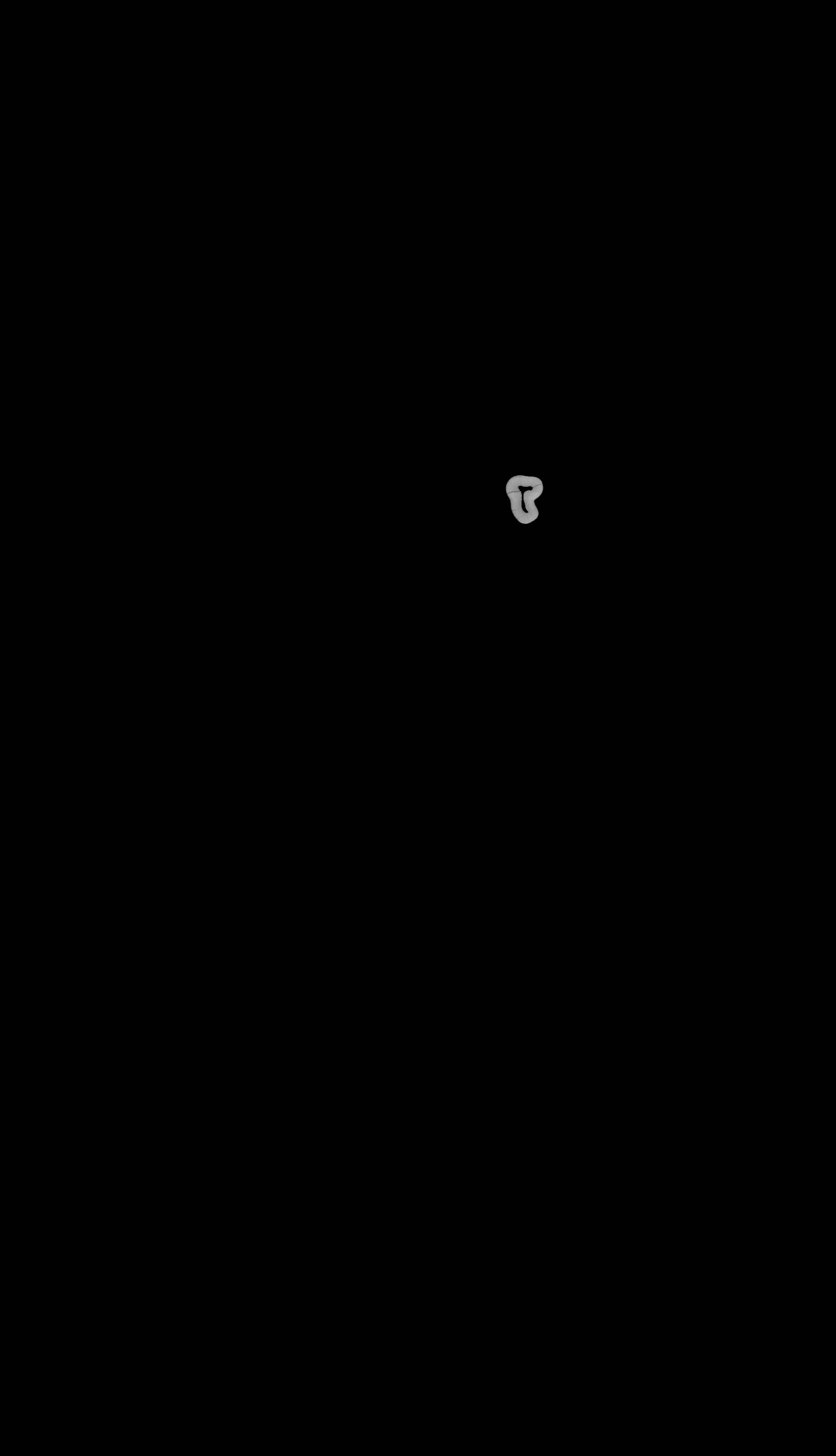

Supplement: Supplementary file 2 — Data S2: Supporting Information. [file AJPA-188-e70164-s001.zip › Cross-Section Tiff Files/amnh_34714_Rm3.tif]

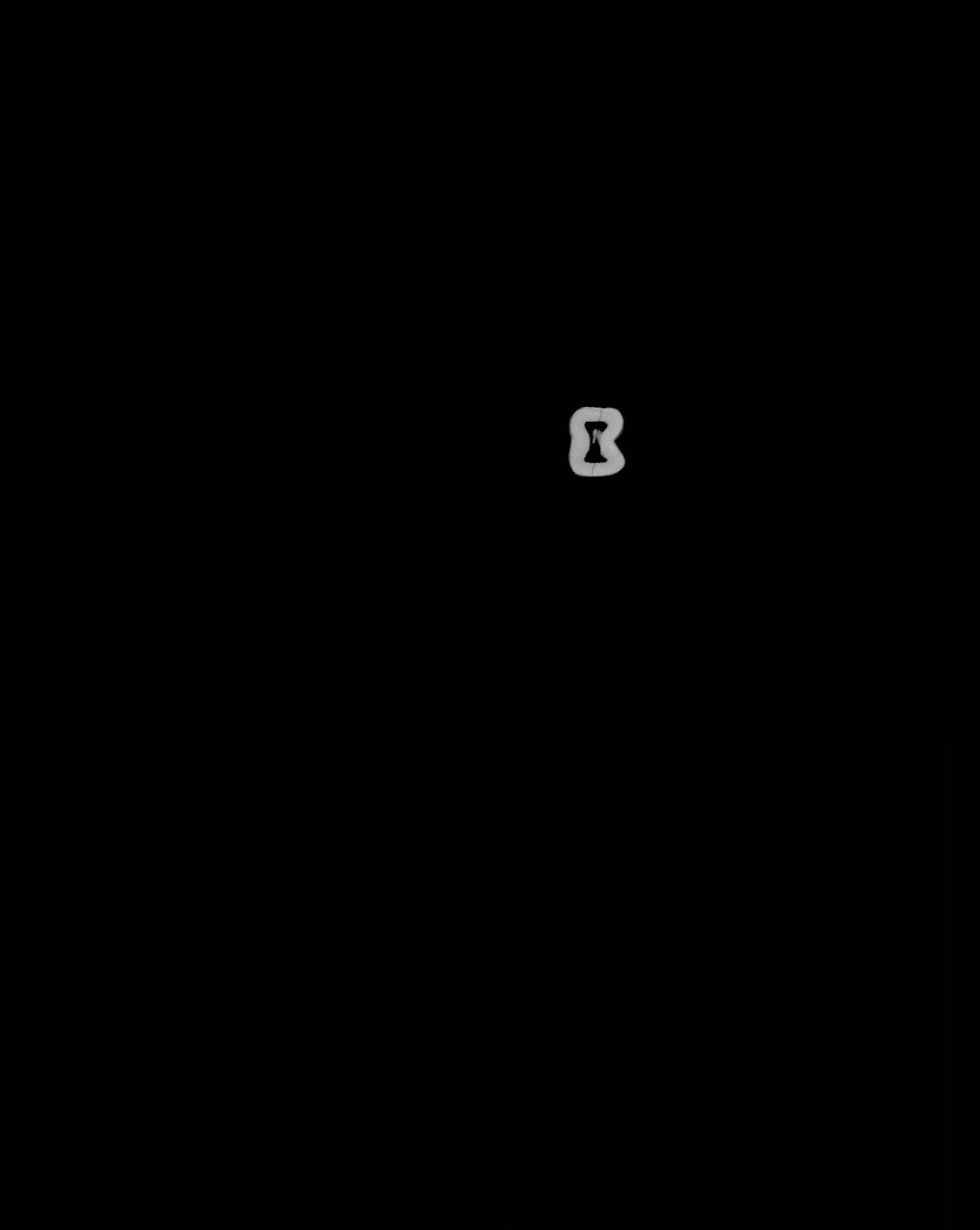

Supplement: Supplementary file 2 — Data S2: Supporting Information. [file AJPA-188-e70164-s001.zip › Cross-Section Tiff Files/mcz_17342_Rm1.tif]

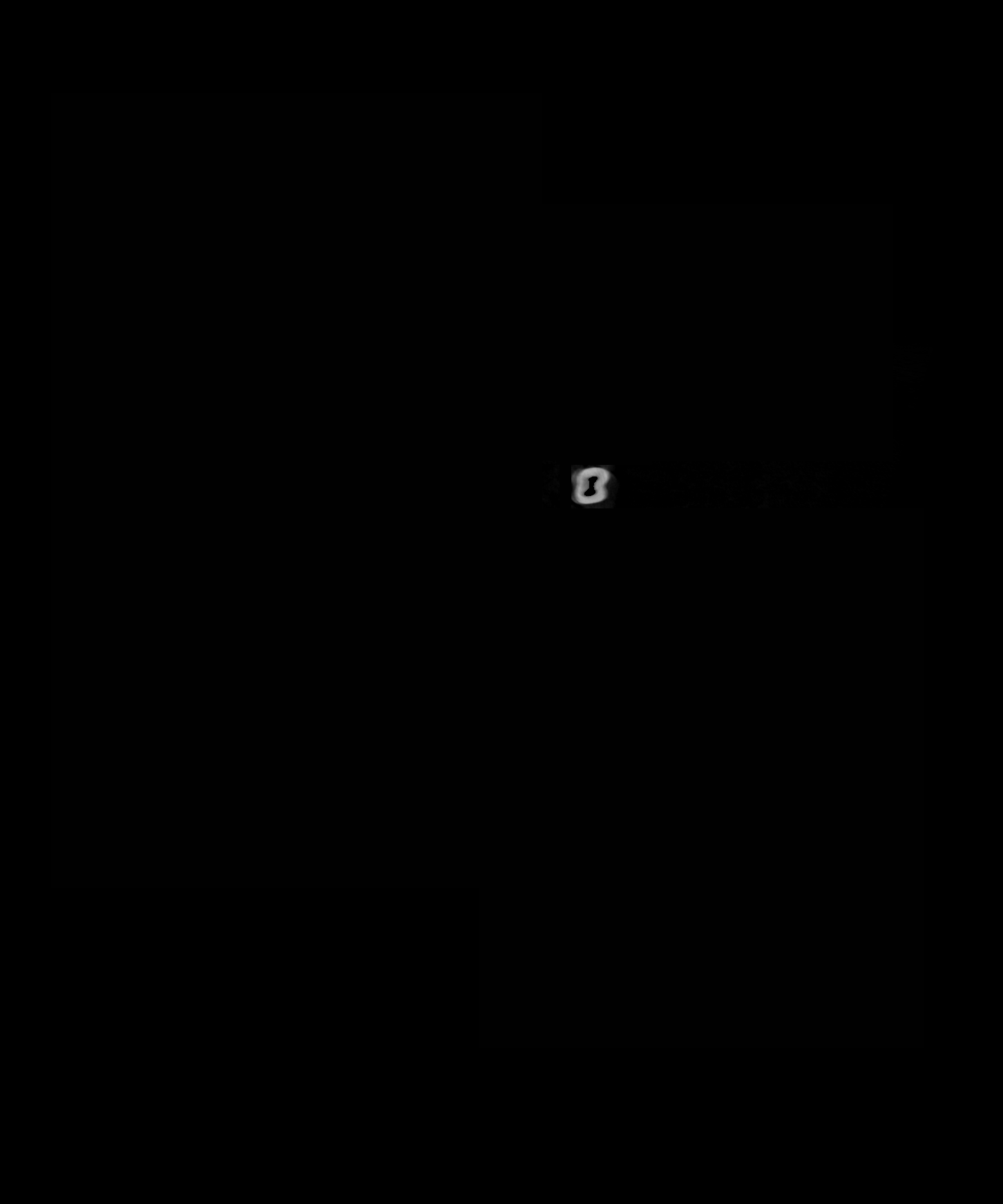

Supplement: Supplementary file 2 — Data S2: Supporting Information. [file AJPA-188-e70164-s001.zip › Cross-Section Tiff Files/mcz_41463_Rm1.tif]

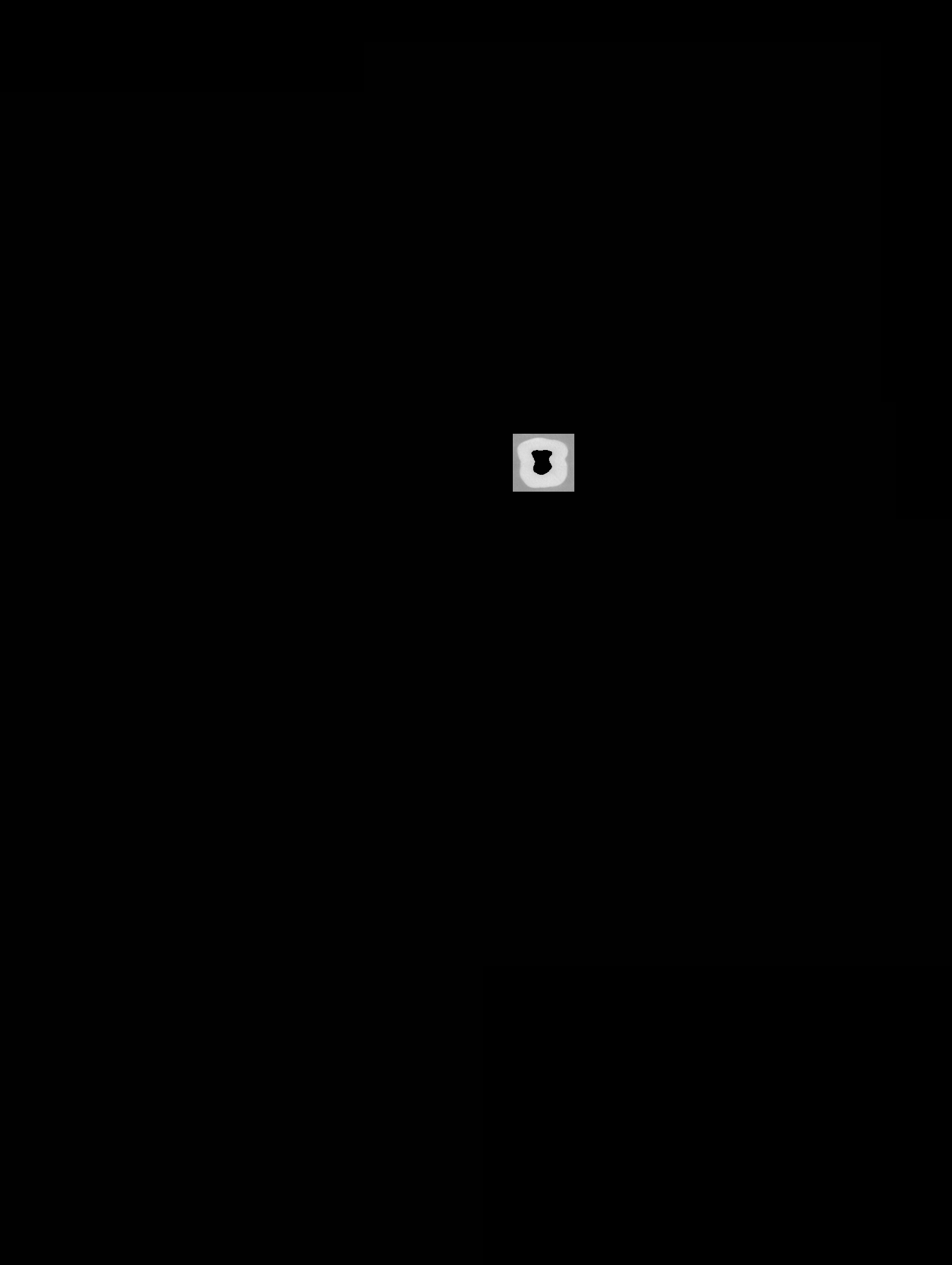

Supplement: Supplementary file 2 — Data S2: Supporting Information. [file AJPA-188-e70164-s001.zip › Cross-Section Tiff Files/mcz_23167_Rm2.tif]

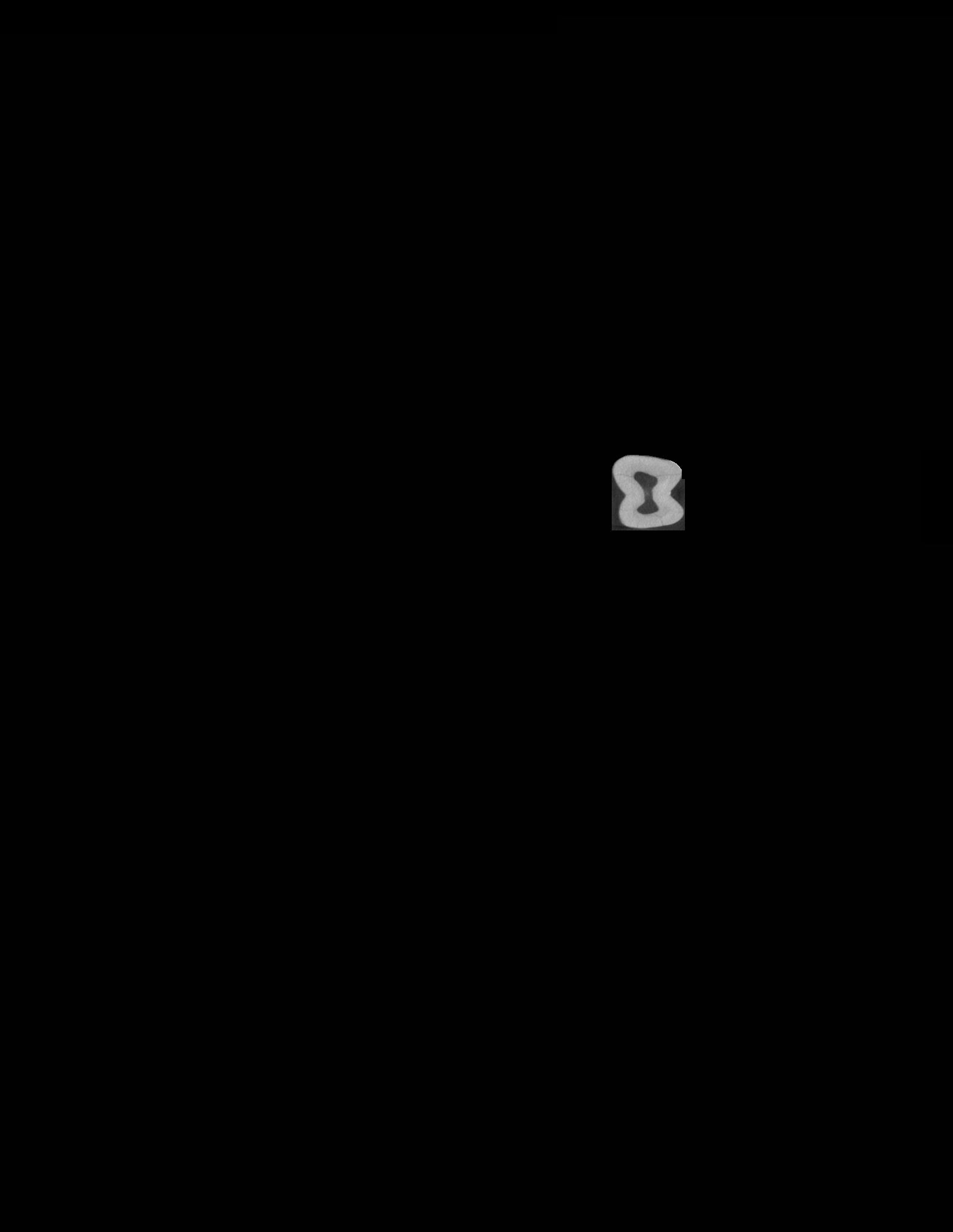

Supplement: Supplementary file 2 — Data S2: Supporting Information. [file AJPA-188-e70164-s001.zip › Cross-Section Tiff Files/mcz_23986_Rm2.tif]

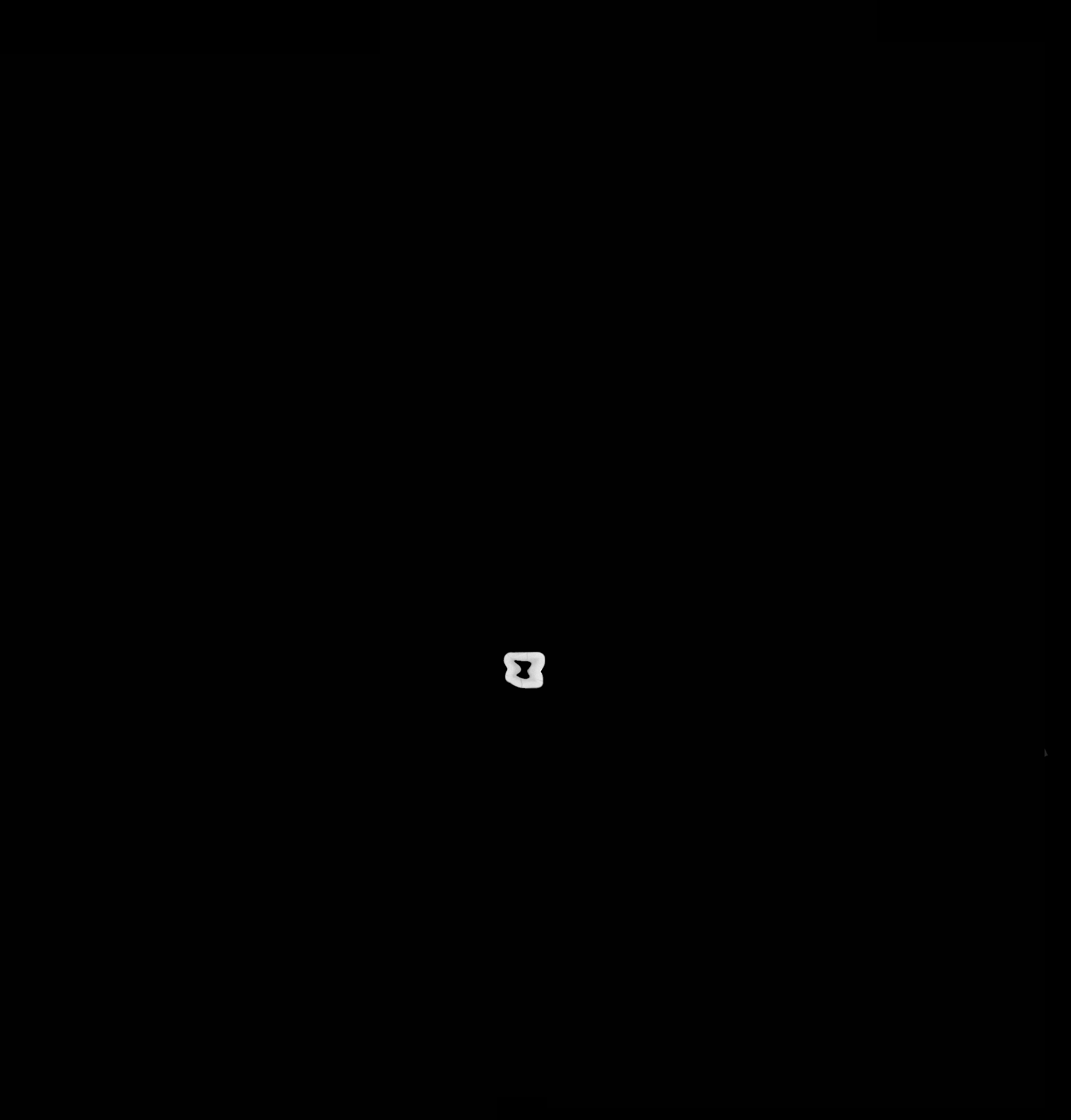

Supplement: Supplementary file 2 — Data S2: Supporting Information. [file AJPA-188-e70164-s001.zip › Cross-Section Tiff Files/amnh_52645_Rm2.tif]

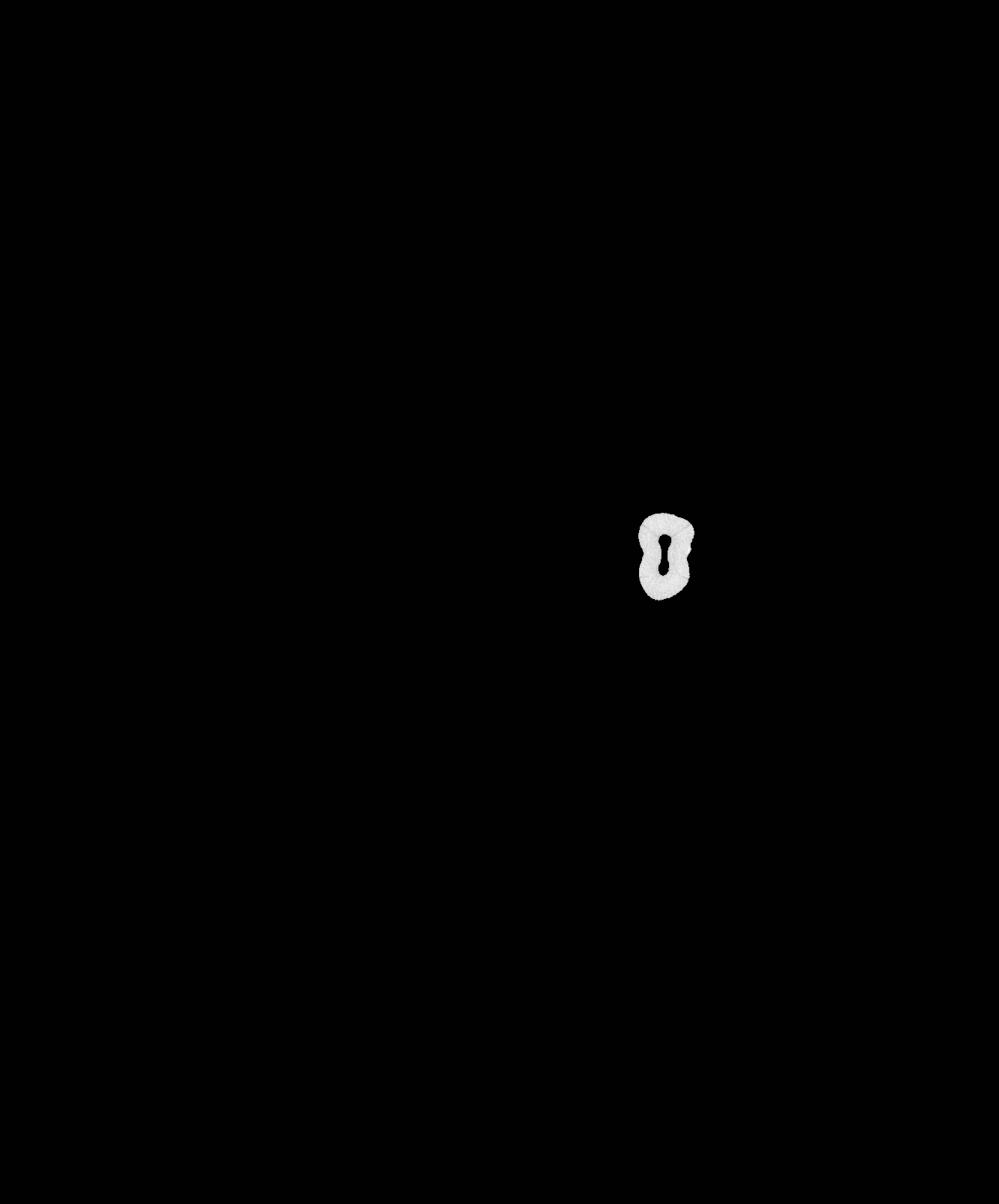

Supplement: Supplementary file 2 — Data S2: Supporting Information. [file AJPA-188-e70164-s001.zip › Cross-Section Tiff Files/mcz_37385_Rm2.tif]

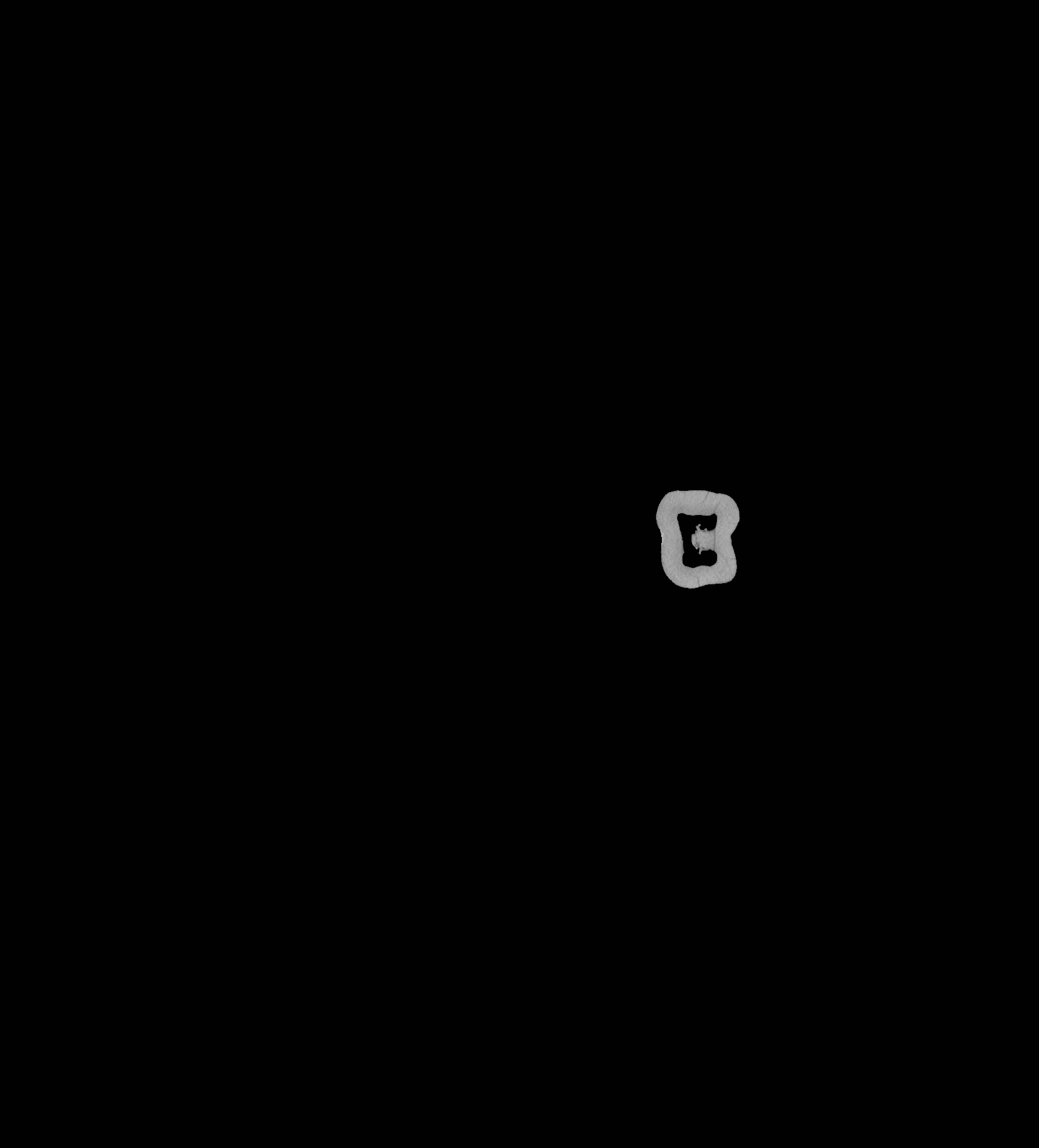

Supplement: Supplementary file 2 — Data S2: Supporting Information. [file AJPA-188-e70164-s001.zip › Cross-Section Tiff Files/mcz_14750_Rm2.tif]

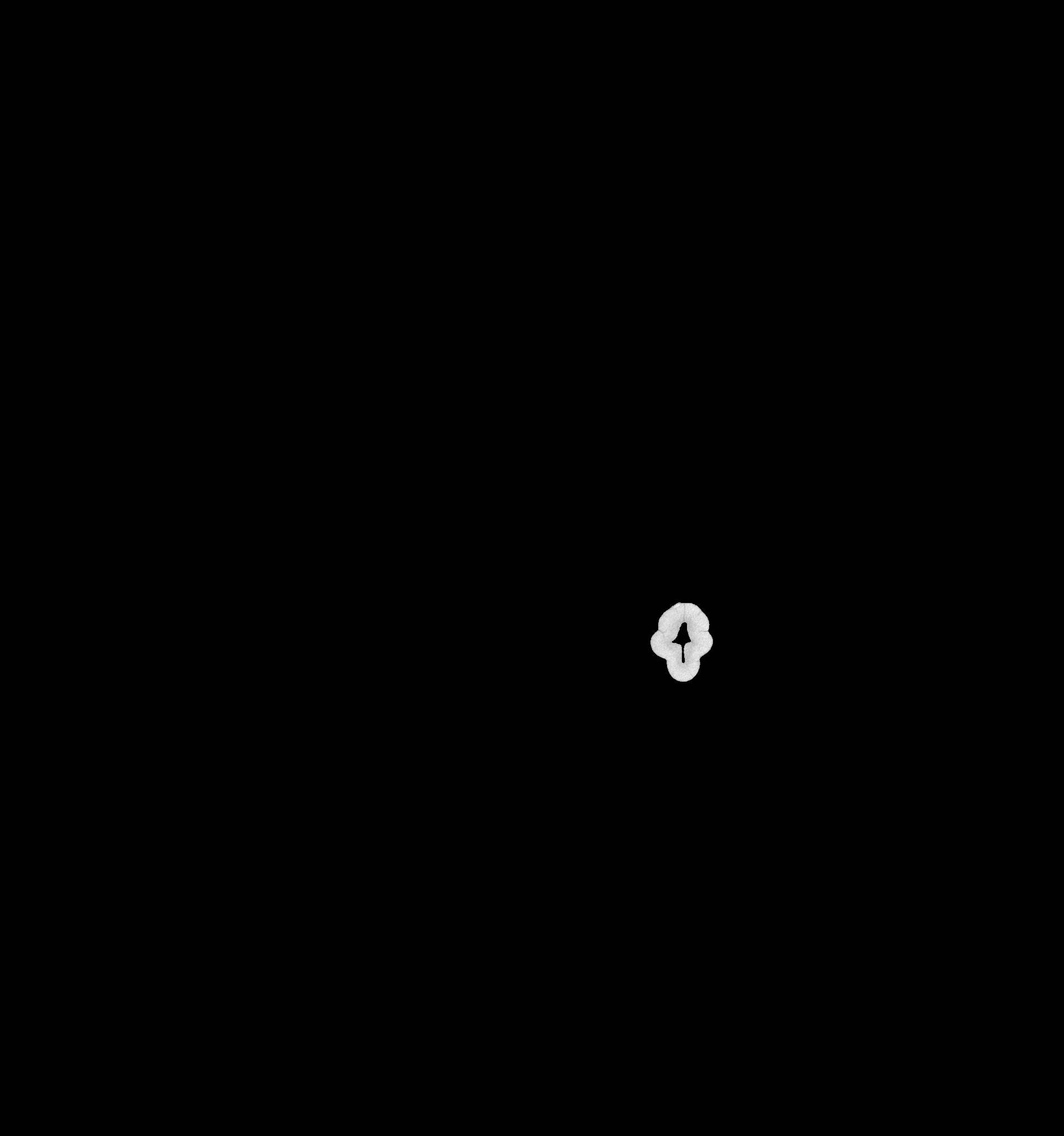

Supplement: Supplementary file 2 — Data S2: Supporting Information. [file AJPA-188-e70164-s001.zip › Cross-Section Tiff Files/mcz_14750_Rm3.tif]

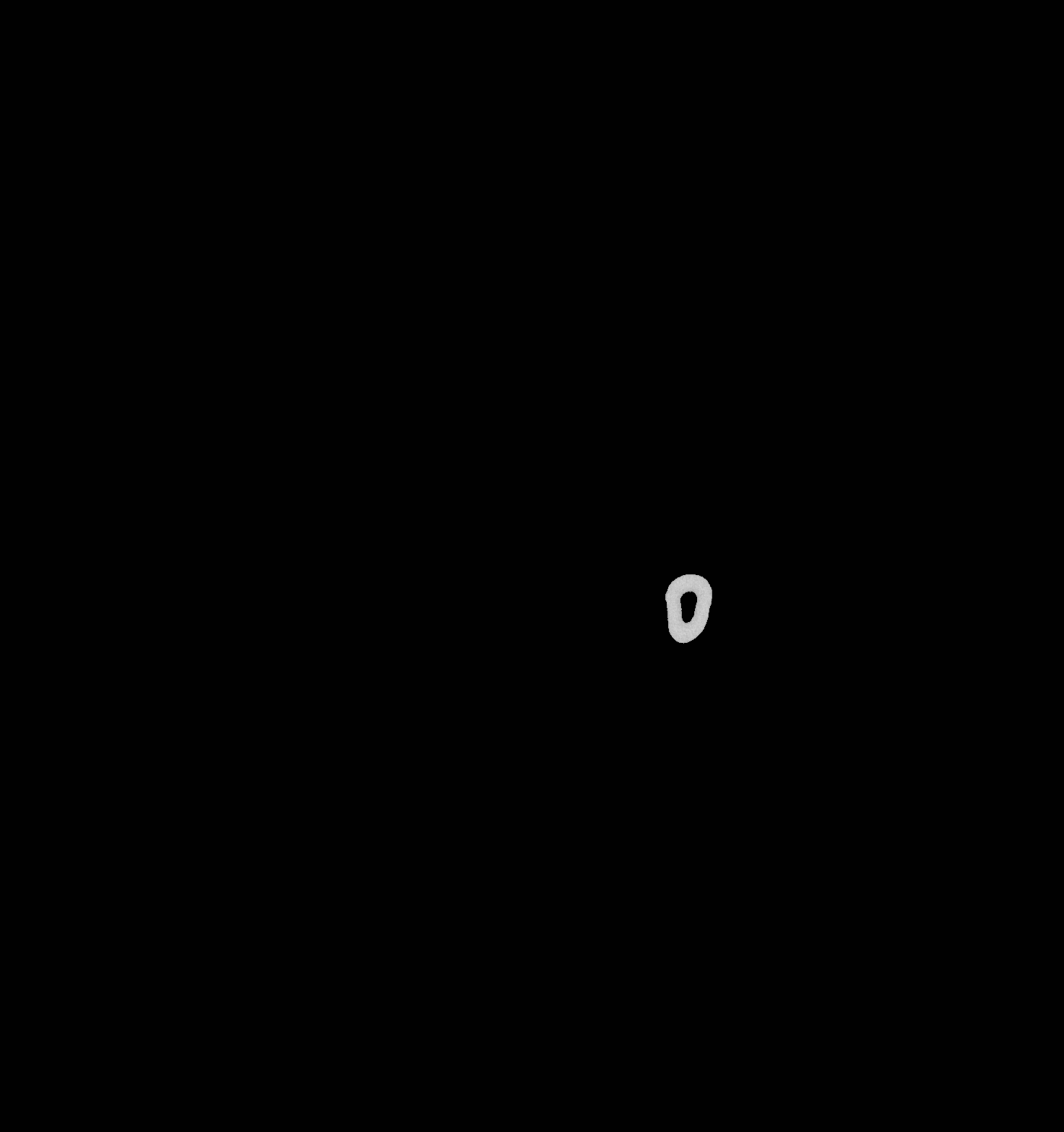

Supplement: Supplementary file 2 — Data S2: Supporting Information. [file AJPA-188-e70164-s001.zip › Cross-Section Tiff Files/mcz_37385_Rm3.tif]

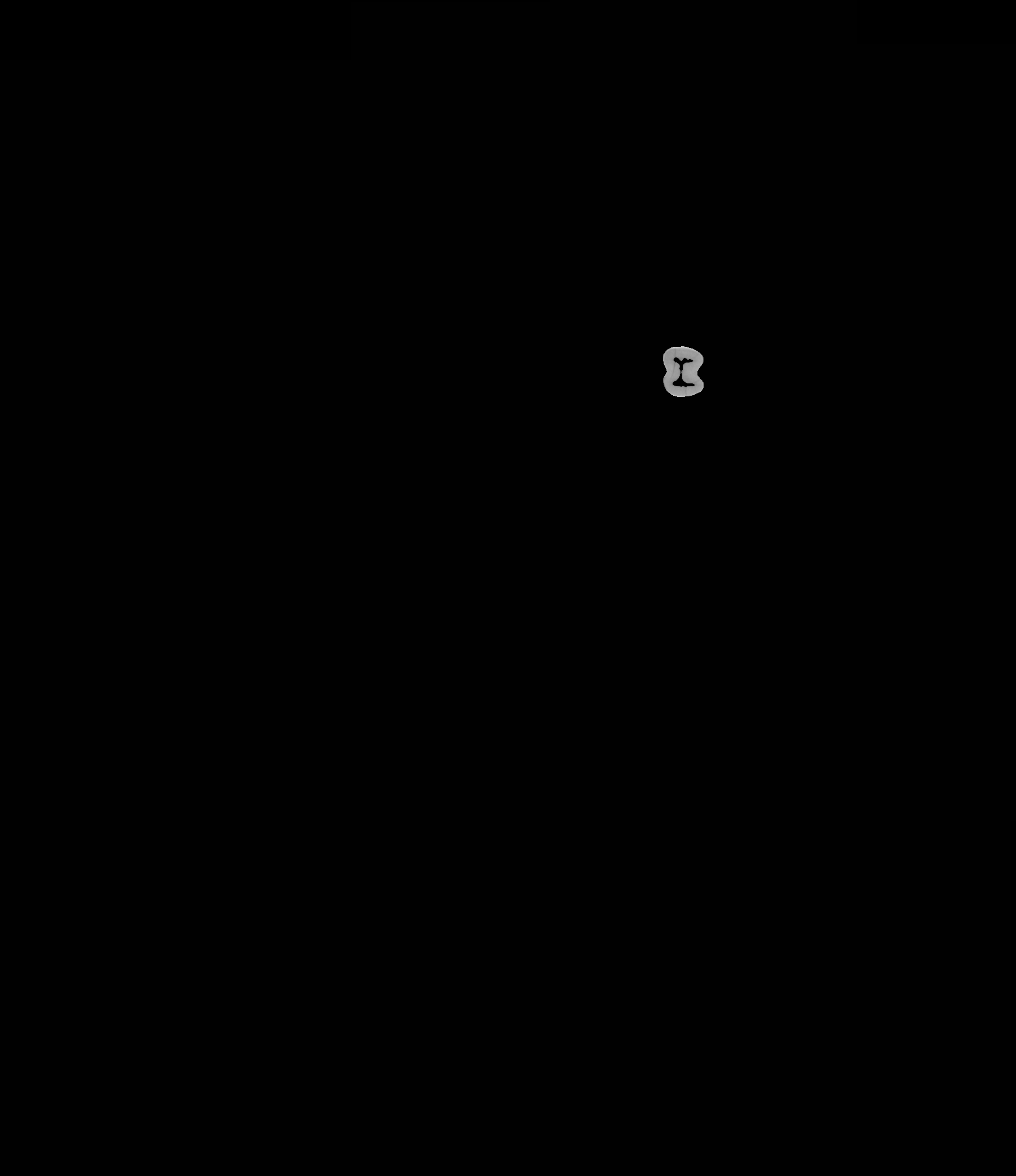

Supplement: Supplementary file 2 — Data S2: Supporting Information. [file AJPA-188-e70164-s001.zip › Cross-Section Tiff Files/amnh_52238_Rm1.tif]

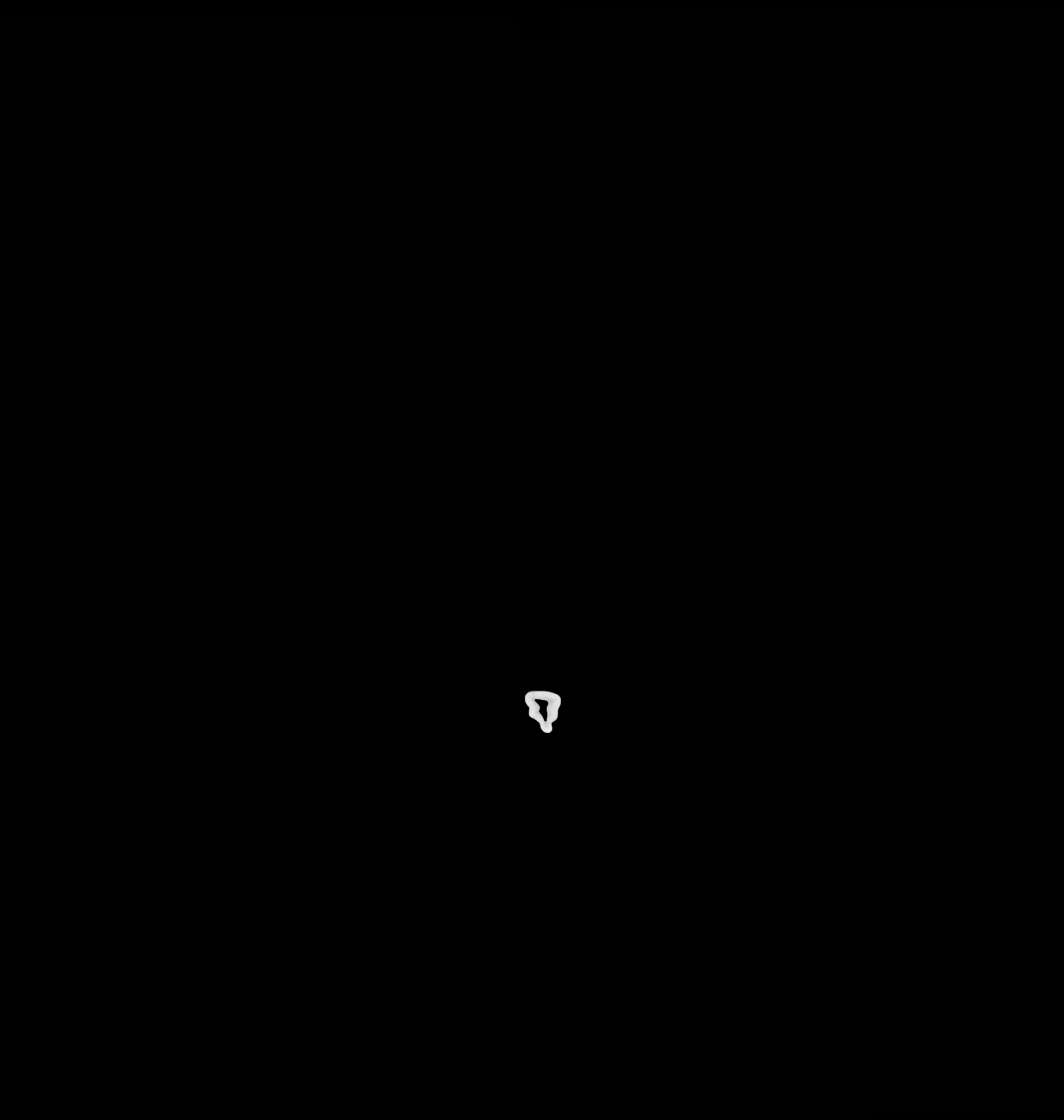

Supplement: Supplementary file 2 — Data S2: Supporting Information. [file AJPA-188-e70164-s001.zip › Cross-Section Tiff Files/amnh_52645_Rm3.tif]

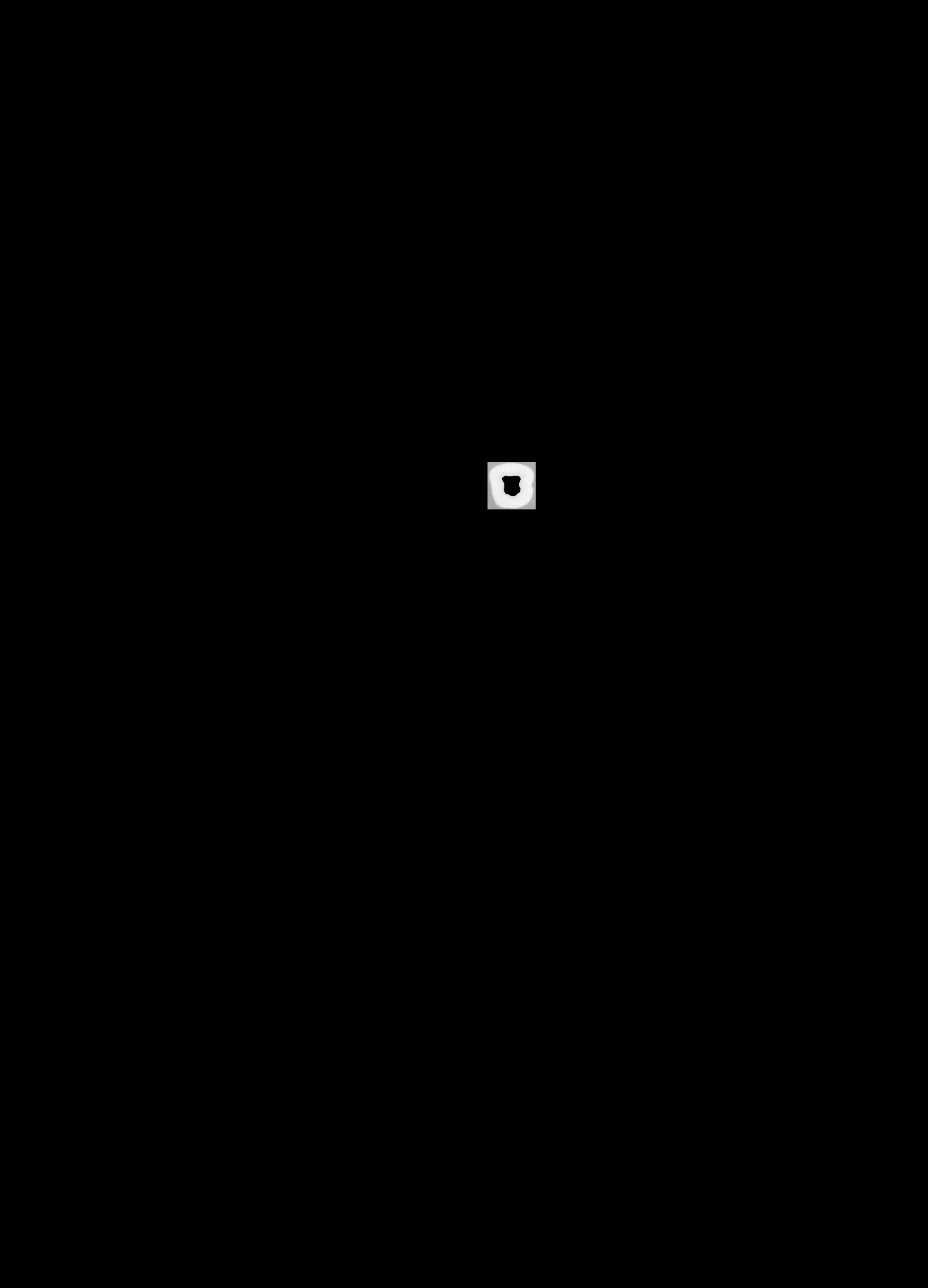

Supplement: Supplementary file 2 — Data S2: Supporting Information. [file AJPA-188-e70164-s001.zip › Cross-Section Tiff Files/mcz_23167_Rm3.tif]

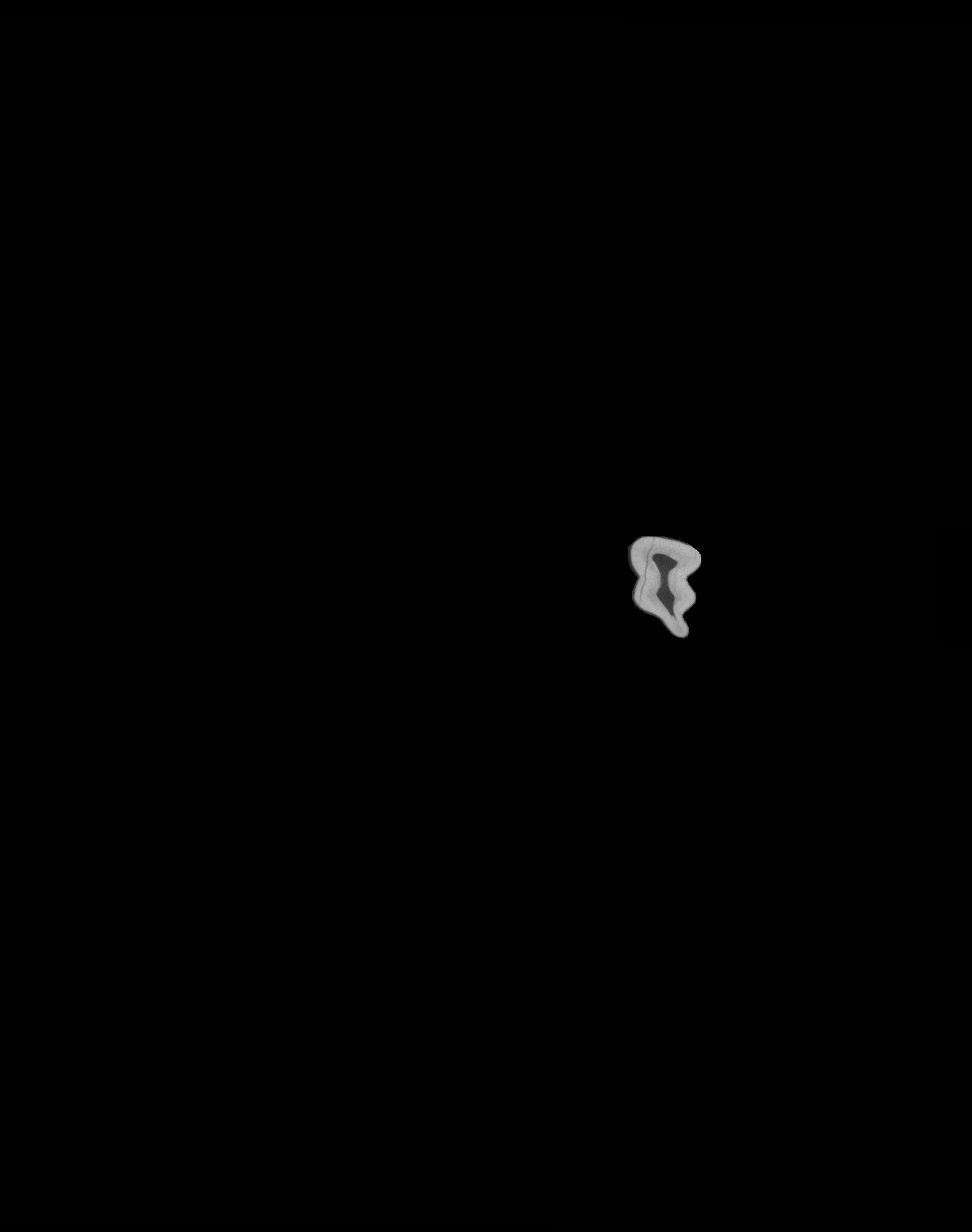

Supplement: Supplementary file 2 — Data S2: Supporting Information. [file AJPA-188-e70164-s001.zip › Cross-Section Tiff Files/mcz_23986_Rm3.tif]

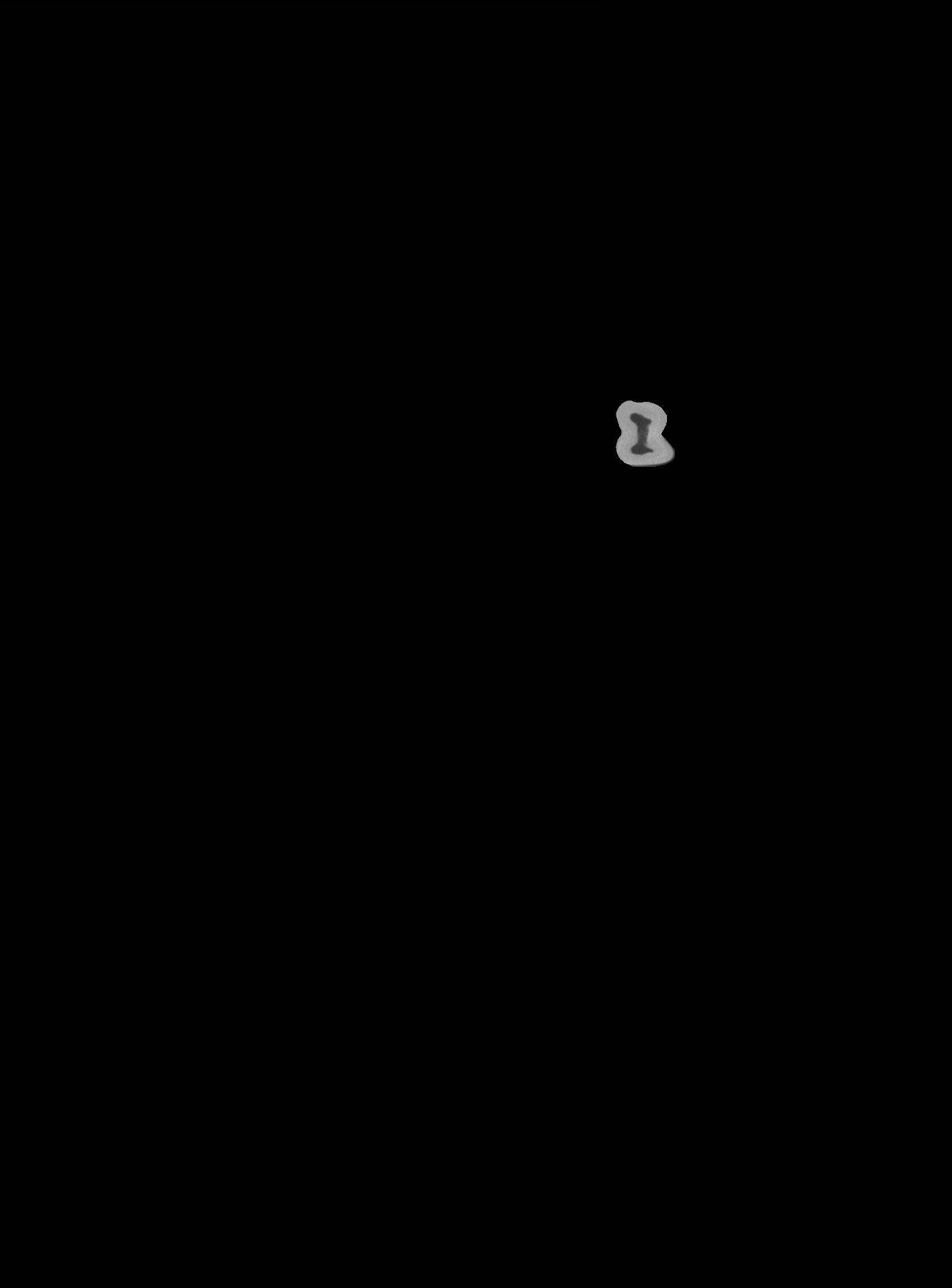

Supplement: Supplementary file 2 — Data S2: Supporting Information. [file AJPA-188-e70164-s001.zip › Cross-Section Tiff Files/mcz_23986_Rm1.tif]

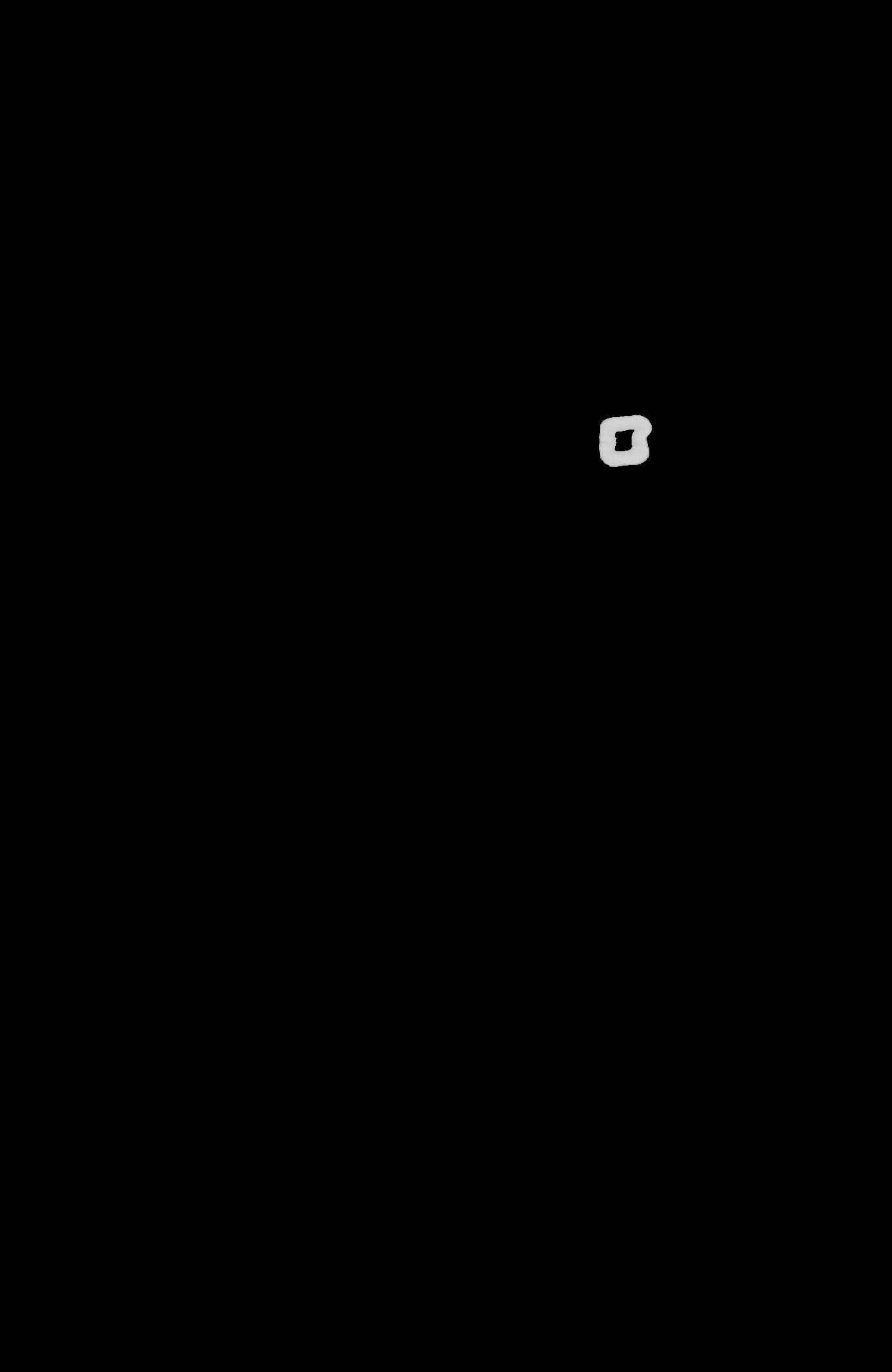

Supplement: Supplementary file 2 — Data S2: Supporting Information. [file AJPA-188-e70164-s001.zip › Cross-Section Tiff Files/mcz_23167_Rm1.tif]

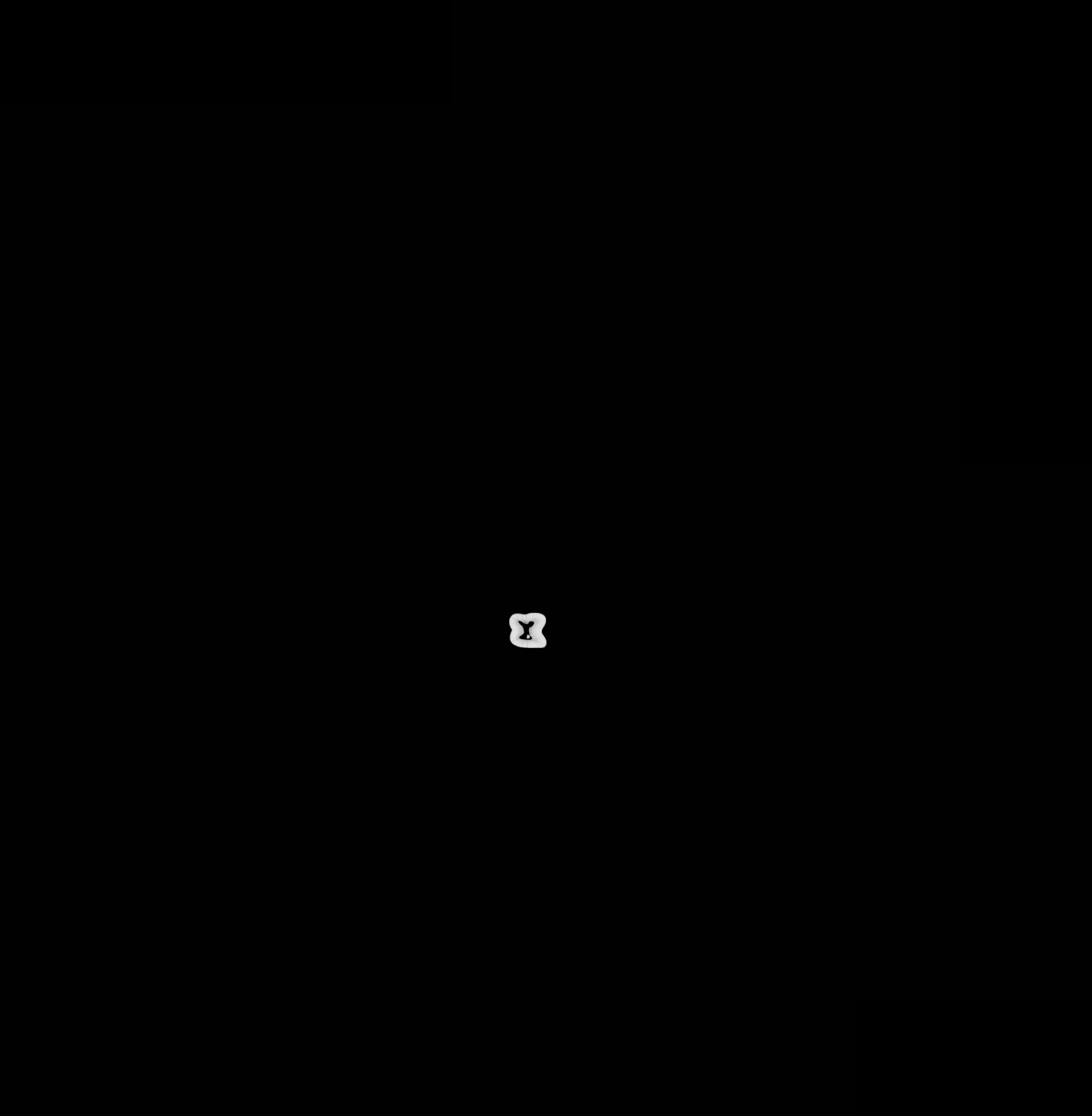

Supplement: Supplementary file 2 — Data S2: Supporting Information. [file AJPA-188-e70164-s001.zip › Cross-Section Tiff Files/amnh_52645_Rm1.tif]

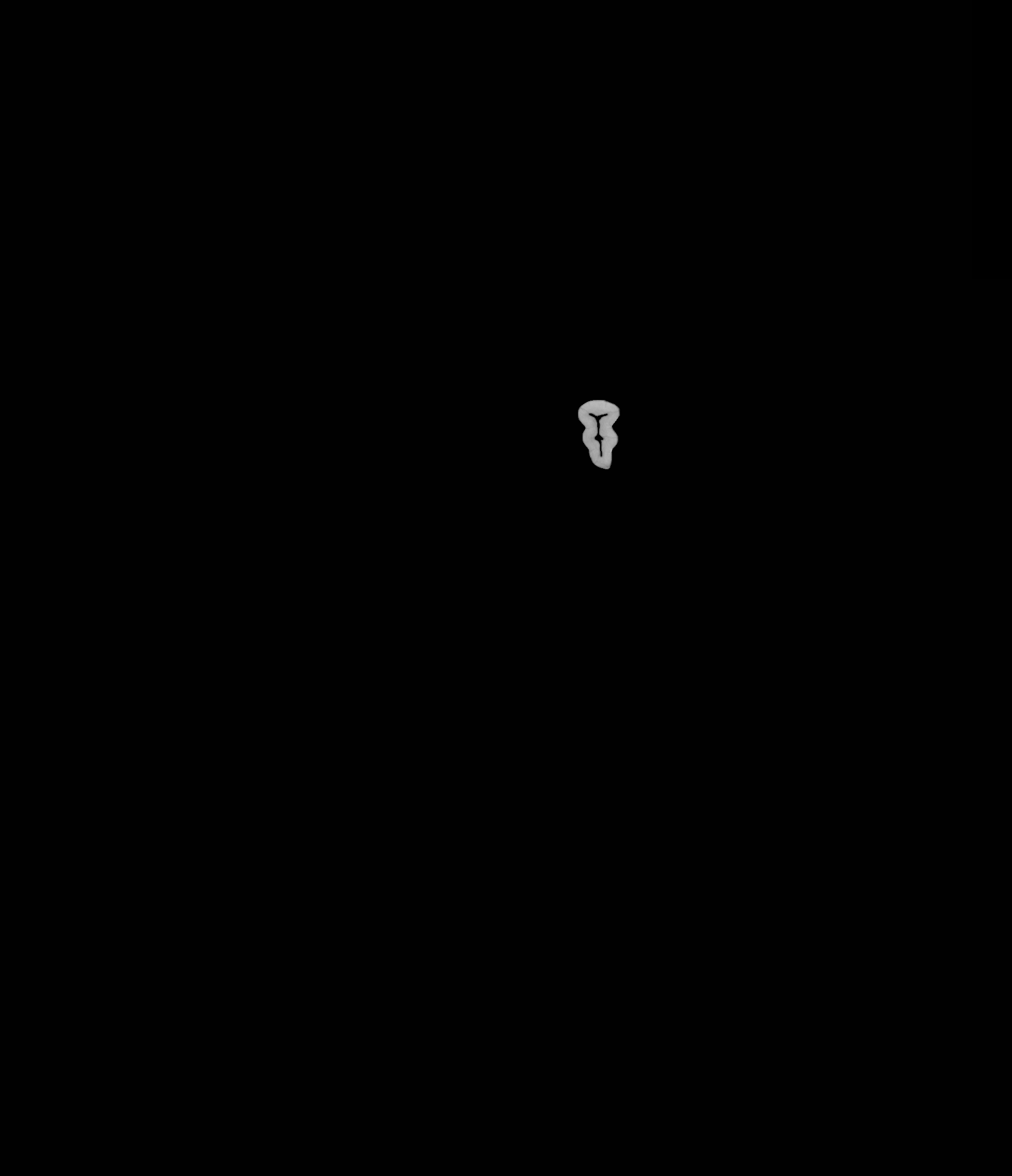

Supplement: Supplementary file 2 — Data S2: Supporting Information. [file AJPA-188-e70164-s001.zip › Cross-Section Tiff Files/amnh_52238_Rm3.tif]

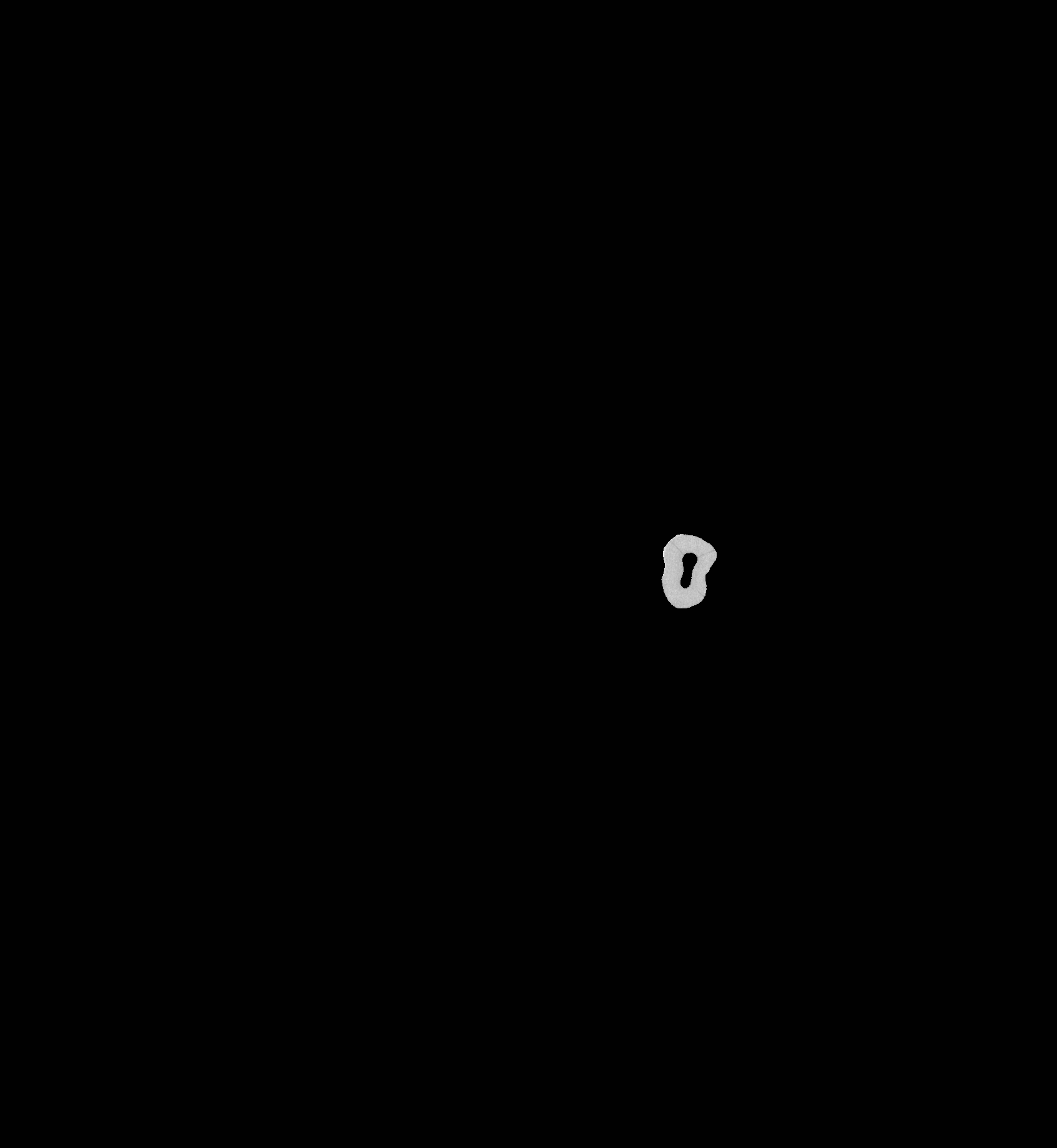

Supplement: Supplementary file 2 — Data S2: Supporting Information. [file AJPA-188-e70164-s001.zip › Cross-Section Tiff Files/mcz_37385_Rm1.tif]

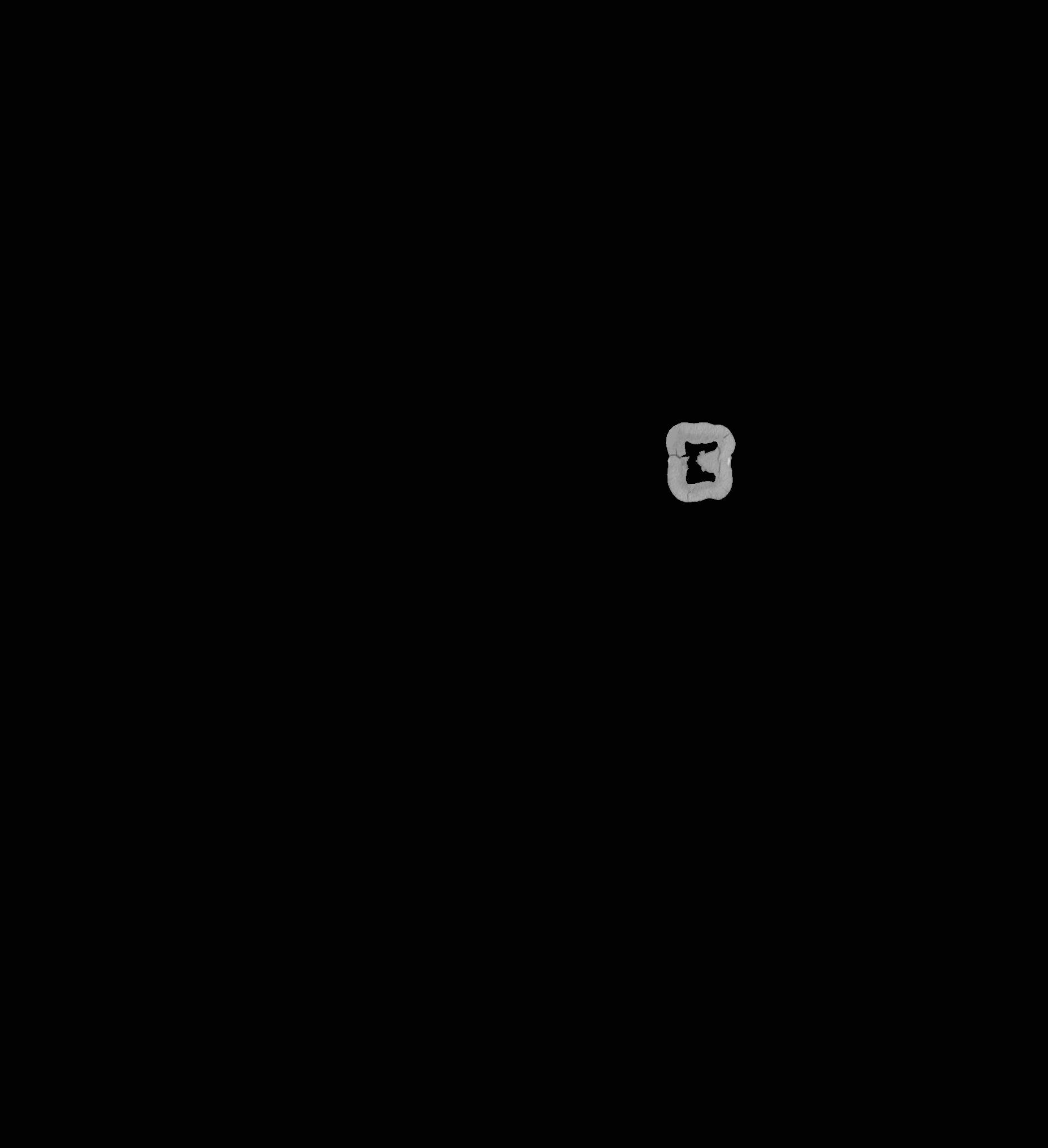

Supplement: Supplementary file 2 — Data S2: Supporting Information. [file AJPA-188-e70164-s001.zip › Cross-Section Tiff Files/mcz_14750_Rm1.tif]

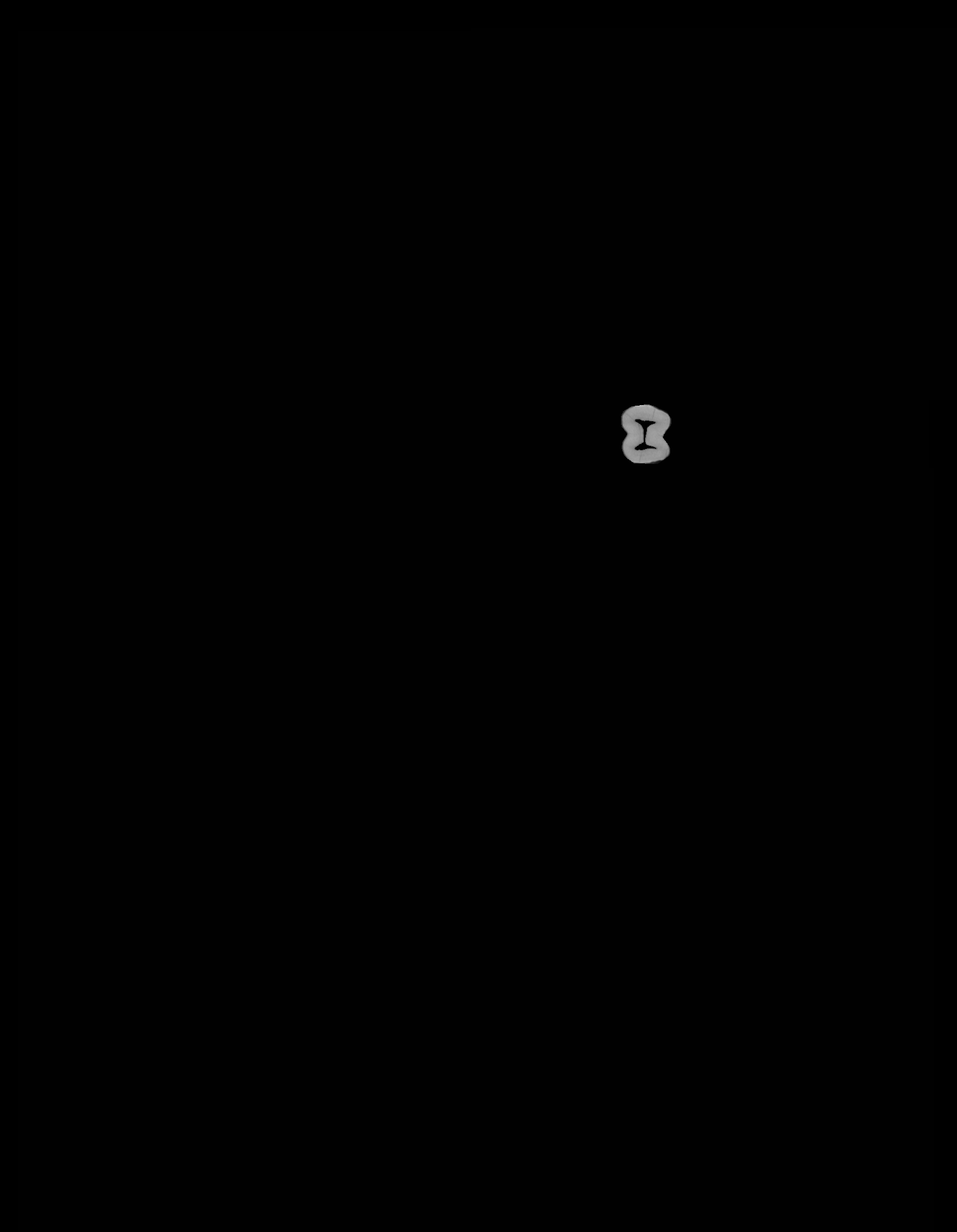

Supplement: Supplementary file 2 — Data S2: Supporting Information. [file AJPA-188-e70164-s001.zip › Cross-Section Tiff Files/amnh_52238_Rm2.tif]

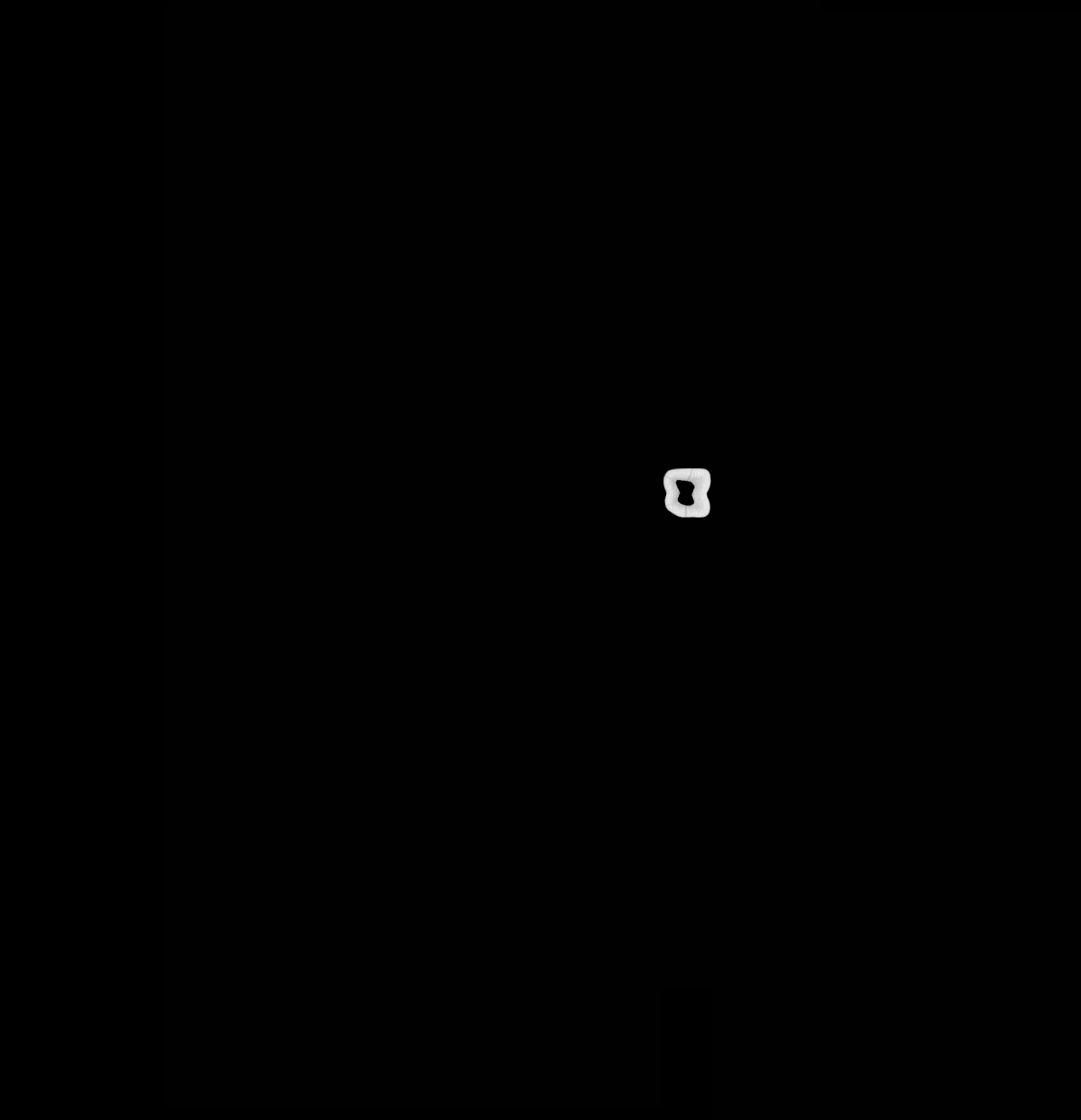

Supplement: Supplementary file 2 — Data S2: Supporting Information. [file AJPA-188-e70164-s001.zip › Cross-Section Tiff Files/amnh_52635_Rm2.tif]

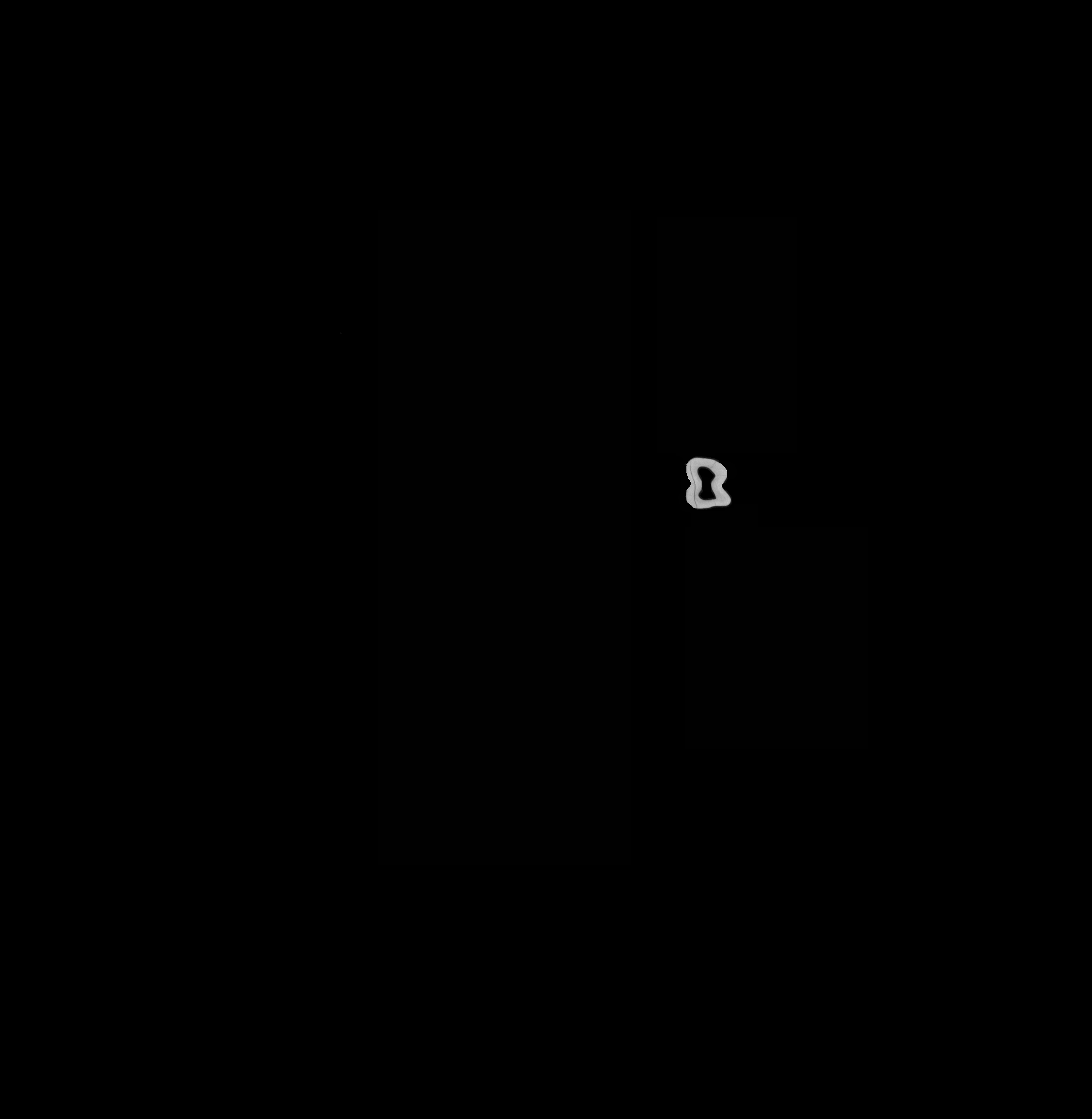

Supplement: Supplementary file 2 — Data S2: Supporting Information. [file AJPA-188-e70164-s001.zip › Cross-Section Tiff Files/amnh_19549_Rm2.tif]

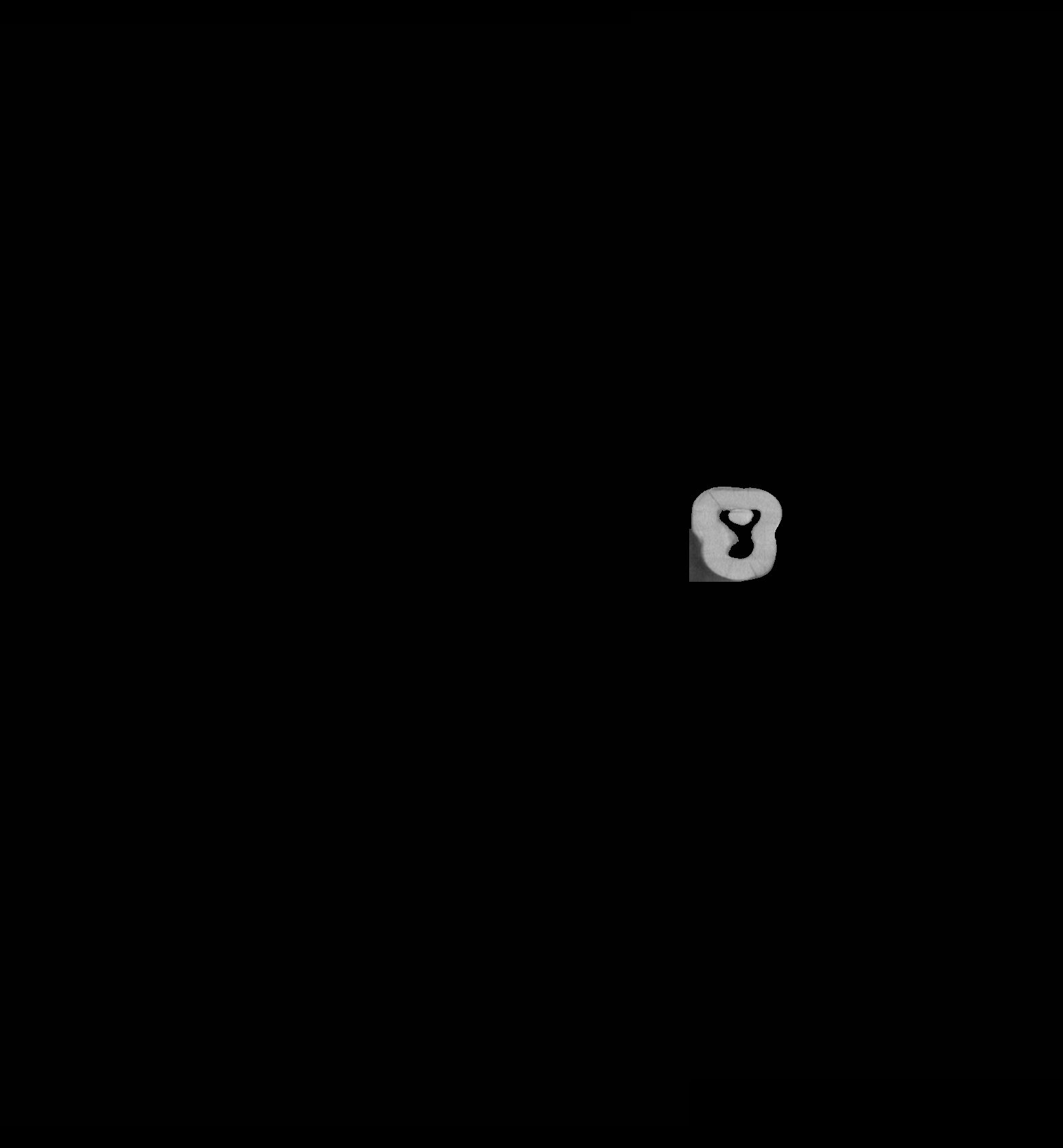

Supplement: Supplementary file 2 — Data S2: Supporting Information. [file AJPA-188-e70164-s001.zip › Cross-Section Tiff Files/mcz_37519_Rm2.tif]

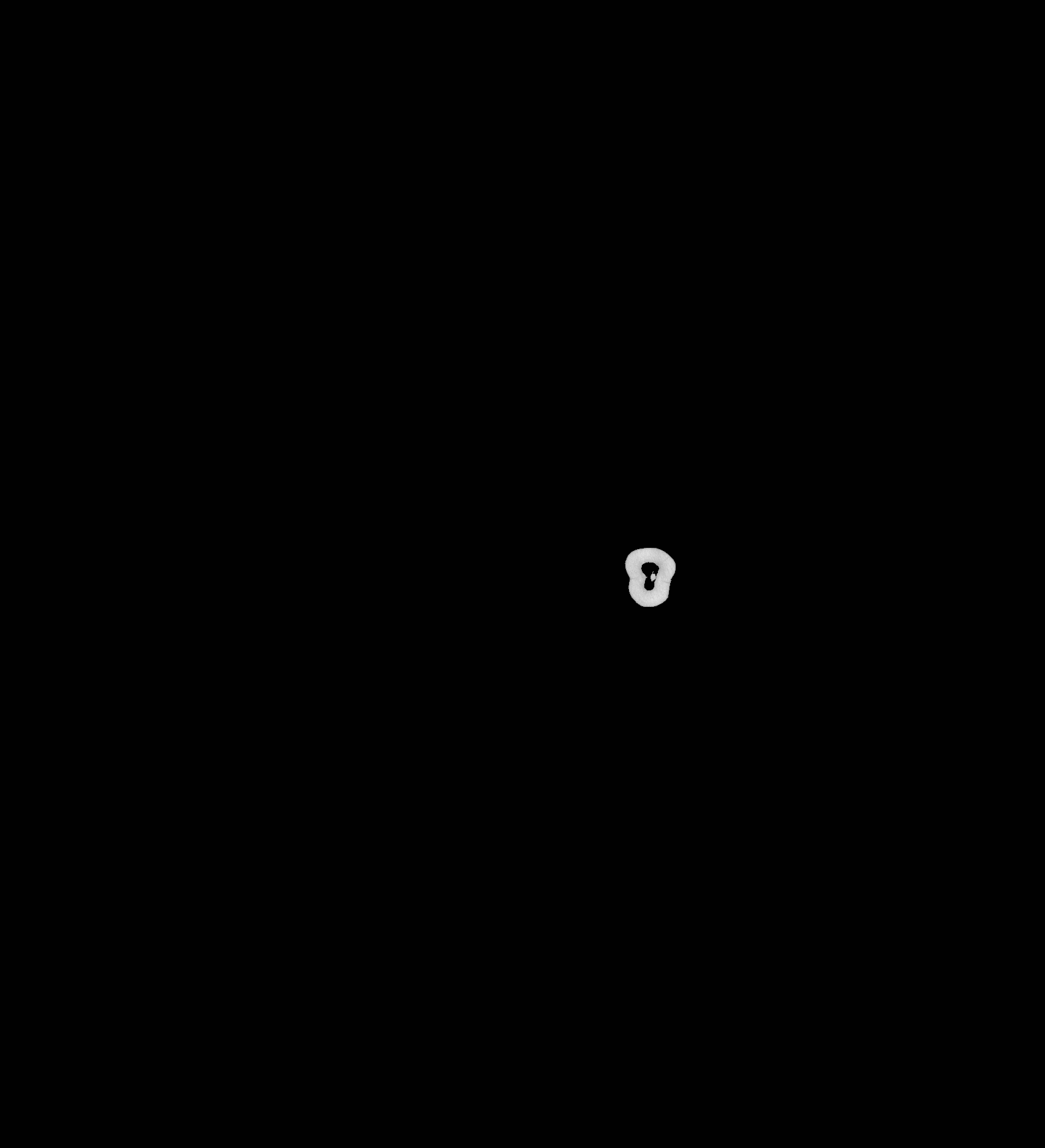

Supplement: Supplementary file 2 — Data S2: Supporting Information. [file AJPA-188-e70164-s001.zip › Cross-Section Tiff Files/mcz_37363_Rm3.tif]

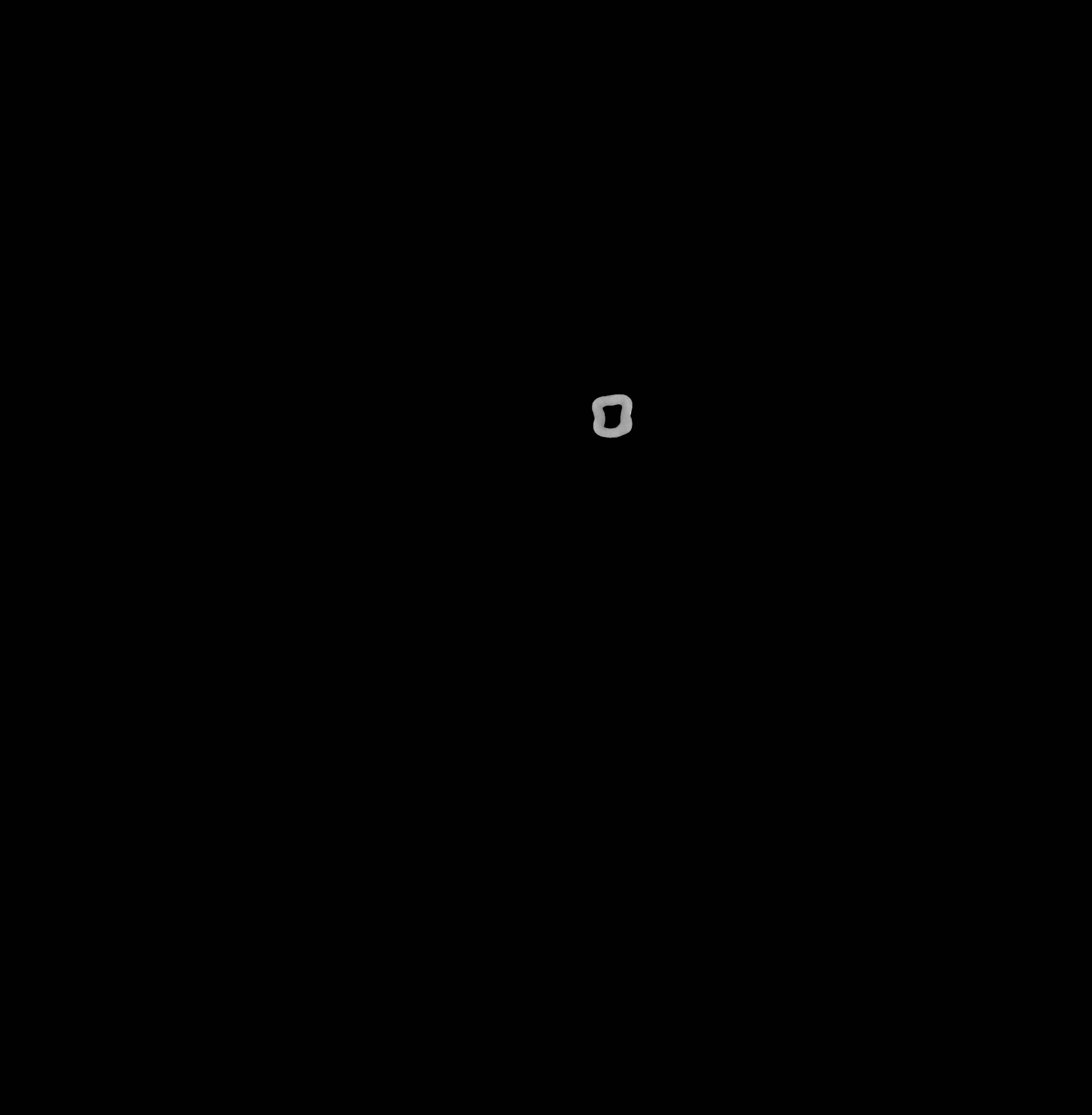

Supplement: Supplementary file 2 — Data S2: Supporting Information. [file AJPA-188-e70164-s001.zip › Cross-Section Tiff Files/mcz_50960_Rm1.tif]

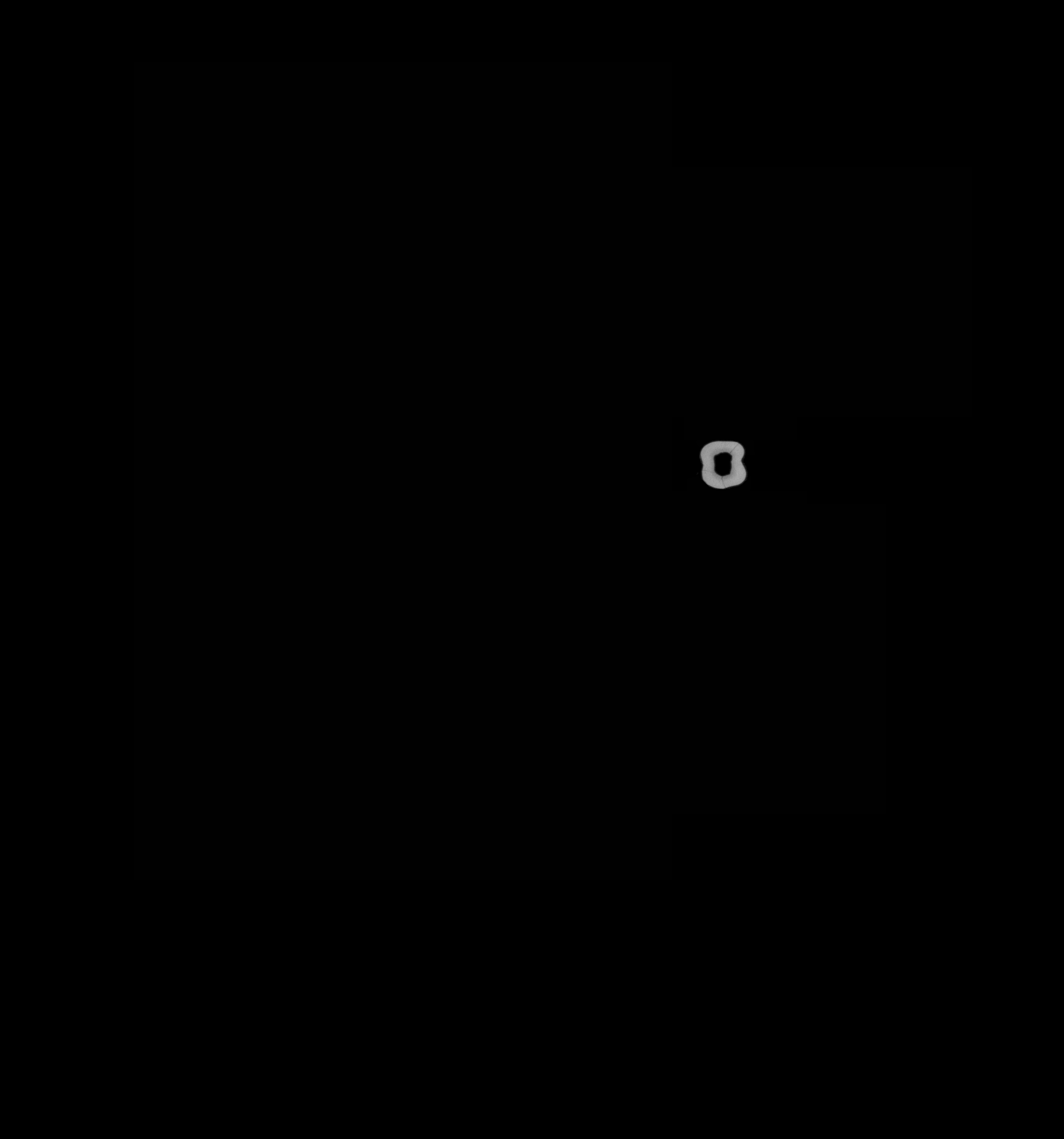

Supplement: Supplementary file 2 — Data S2: Supporting Information. [file AJPA-188-e70164-s001.zip › Cross-Section Tiff Files/amnh_102724_Rm2.tif]

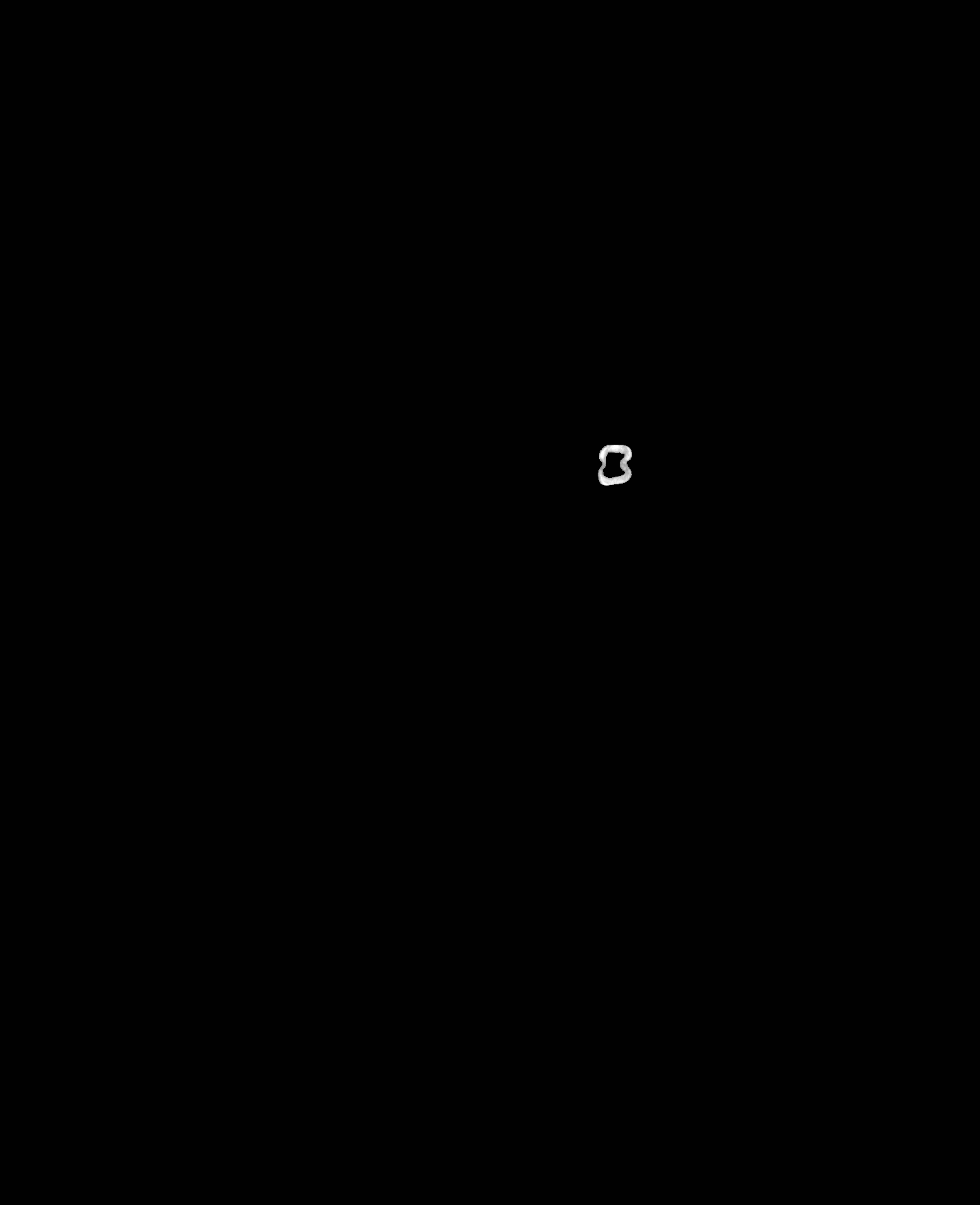

Supplement: Supplementary file 2 — Data S2: Supporting Information. [file AJPA-188-e70164-s001.zip › Cross-Section Tiff Files/mcz_41460_Rm1.tif]

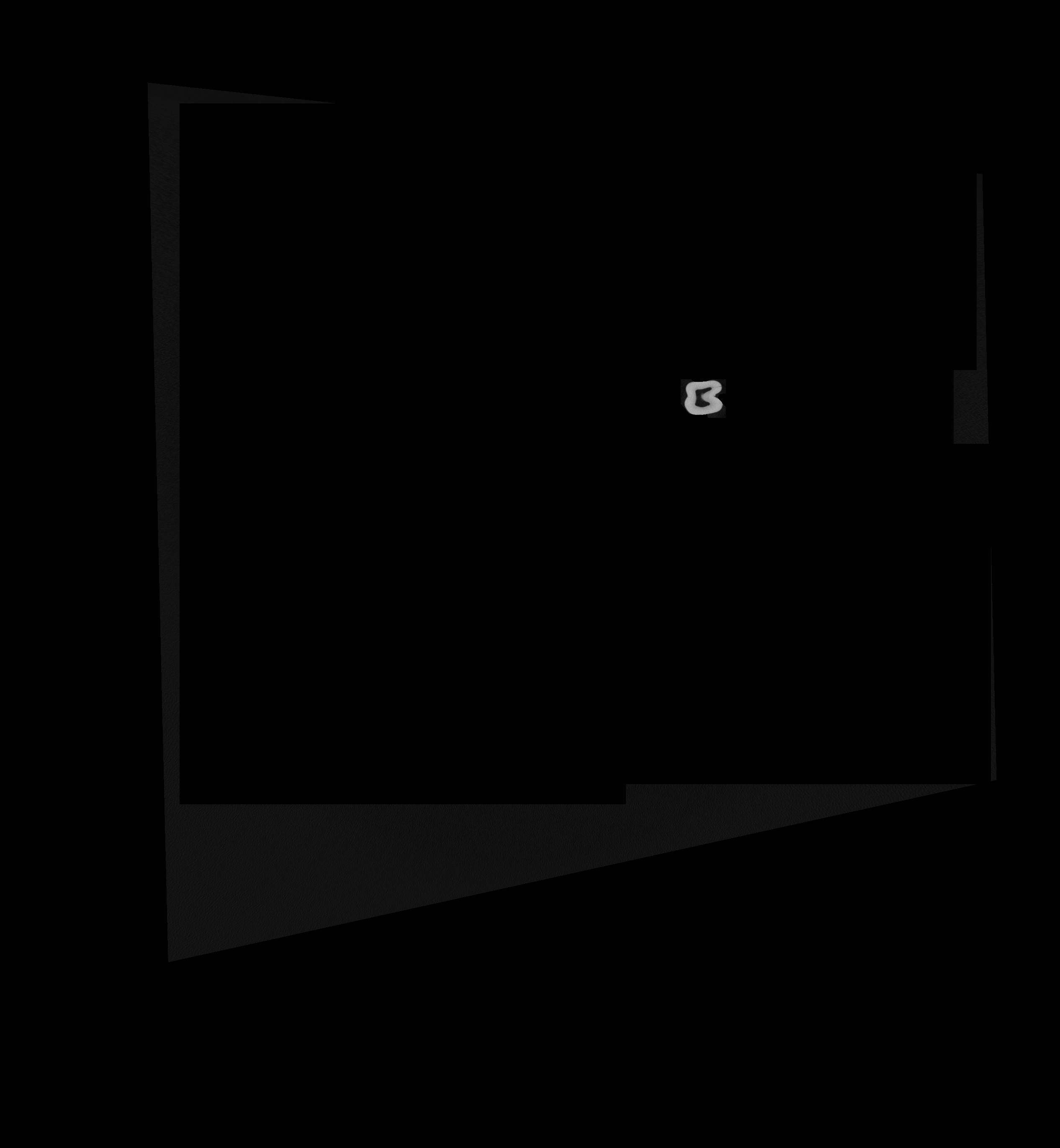

Supplement: Supplementary file 2 — Data S2: Supporting Information. [file AJPA-188-e70164-s001.zip › Cross-Section Tiff Files/amnh_167342_Rm1.tif]

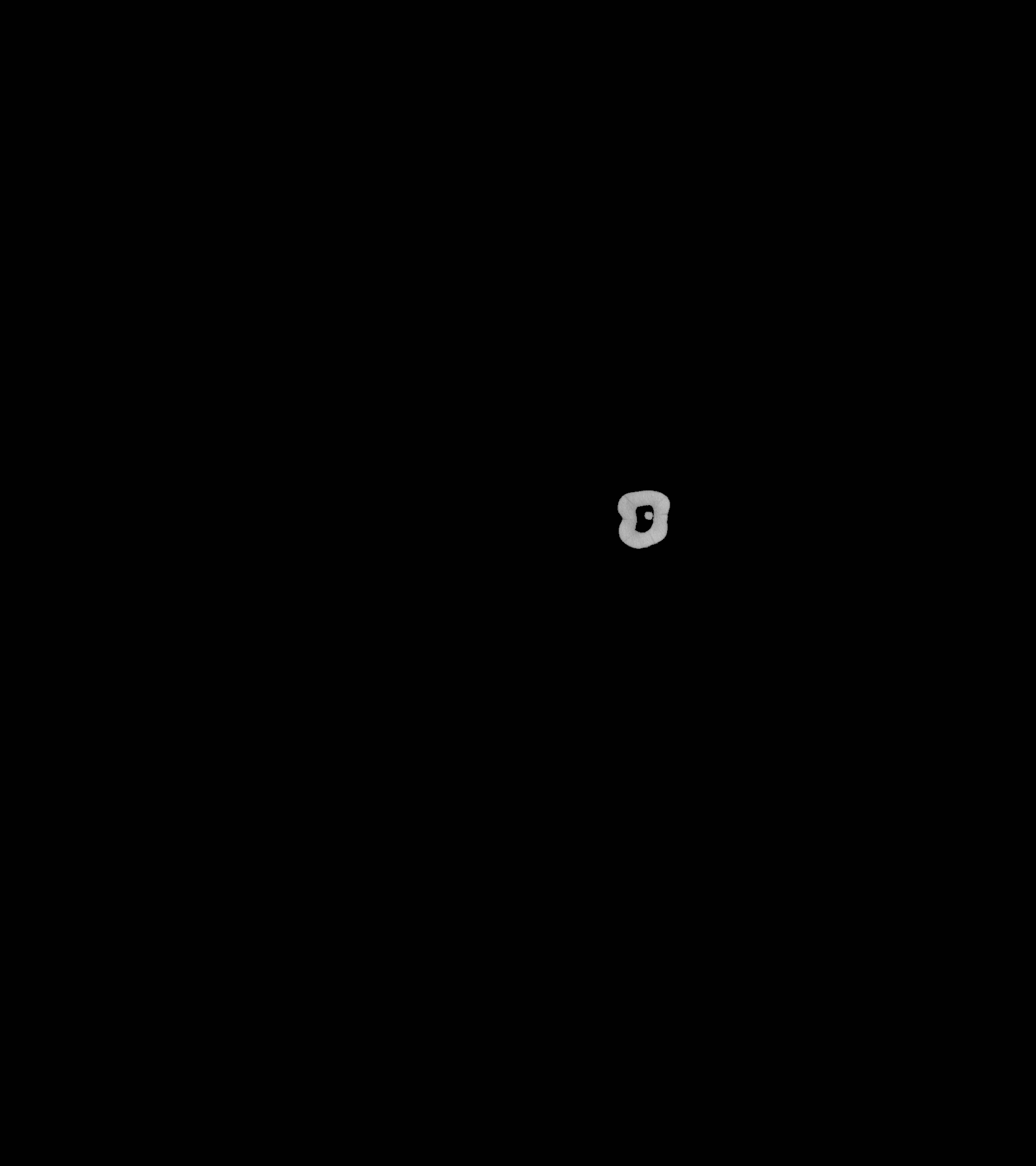

Supplement: Supplementary file 2 — Data S2: Supporting Information. [file AJPA-188-e70164-s001.zip › Cross-Section Tiff Files/mcz_37363_Rm2.tif]

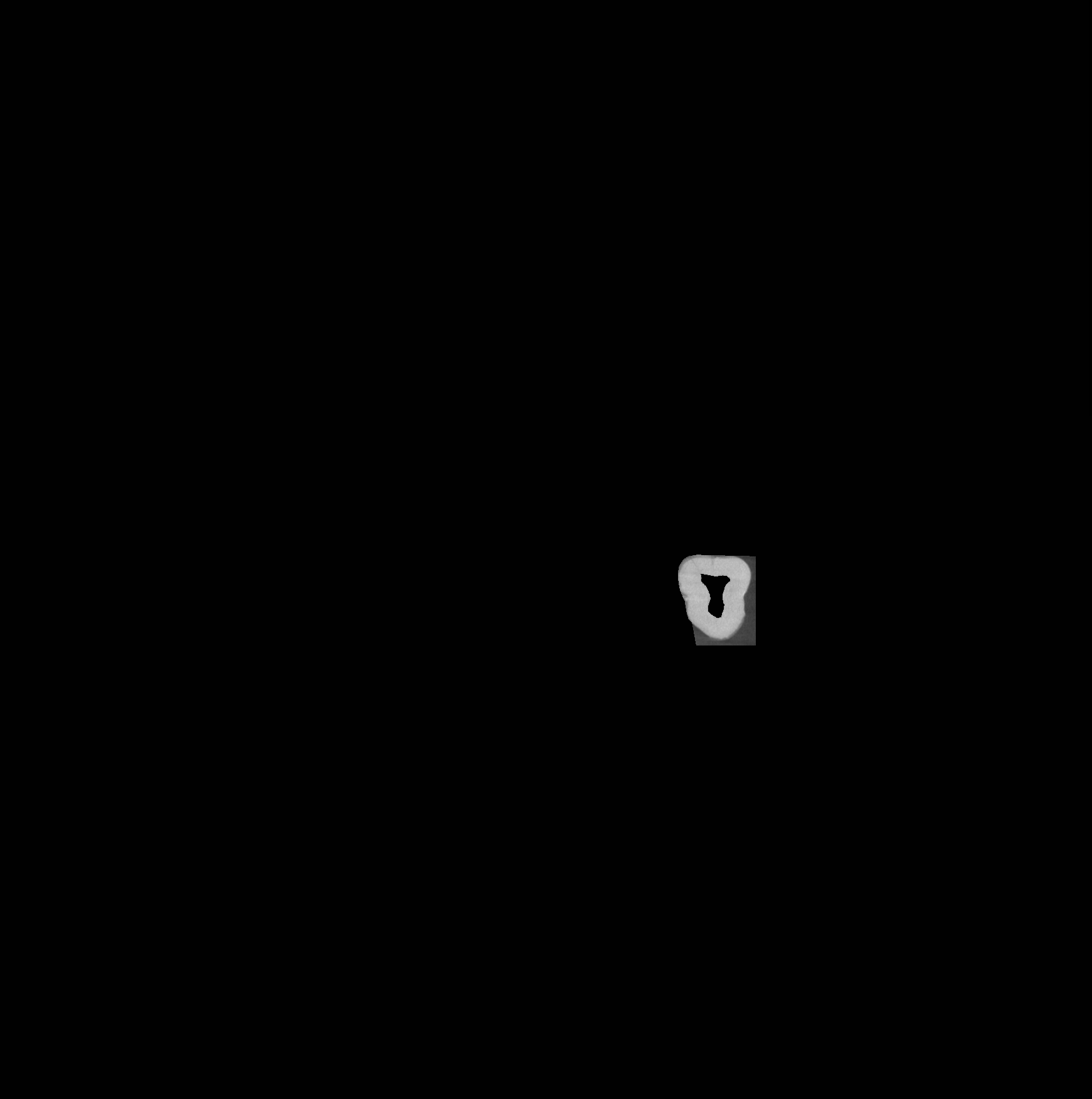

Supplement: Supplementary file 2 — Data S2: Supporting Information. [file AJPA-188-e70164-s001.zip › Cross-Section Tiff Files/mcz_37519_Rm3.tif]

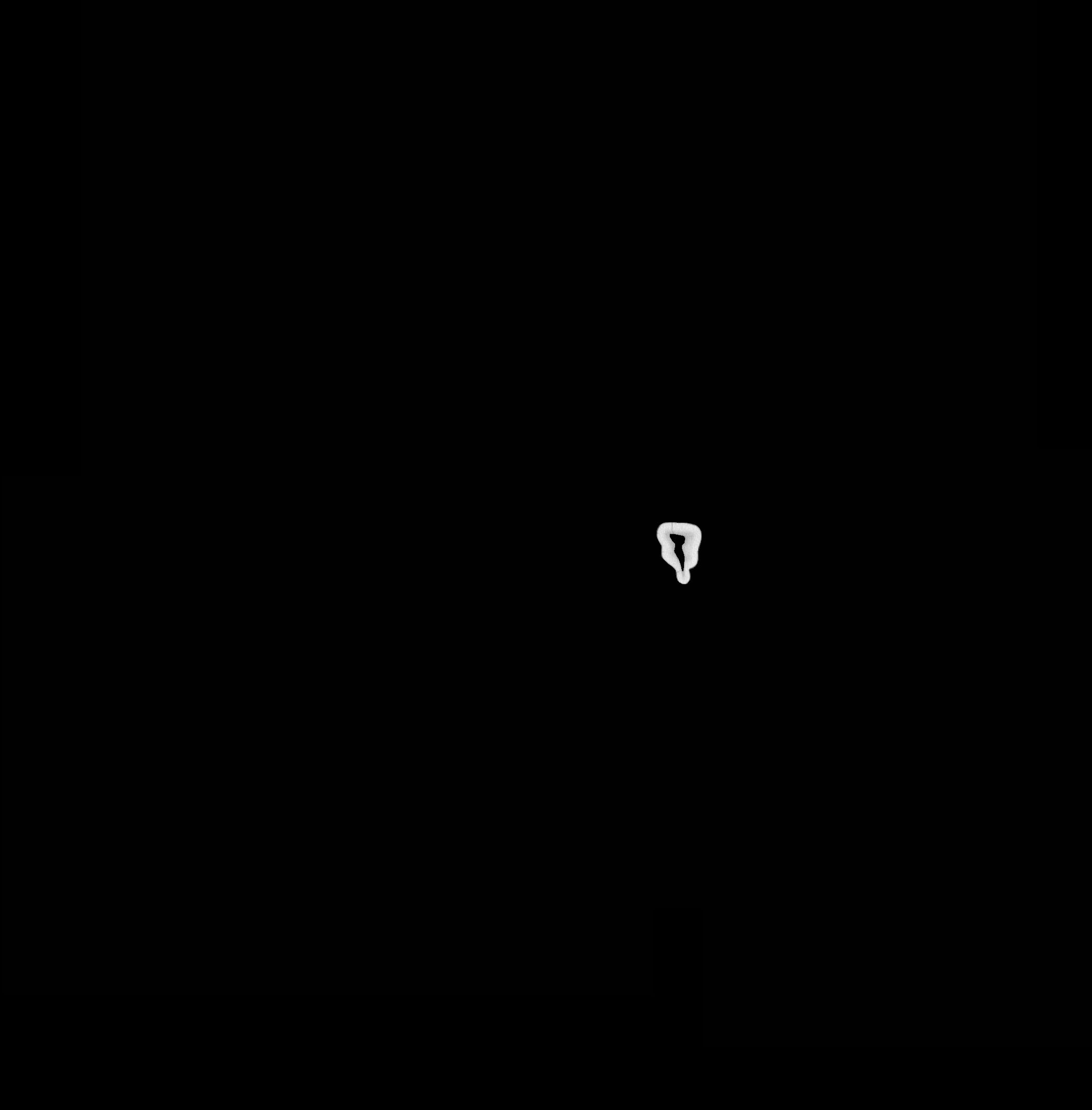

Supplement: Supplementary file 2 — Data S2: Supporting Information. [file AJPA-188-e70164-s001.zip › Cross-Section Tiff Files/amnh_52635_Rm3.tif]

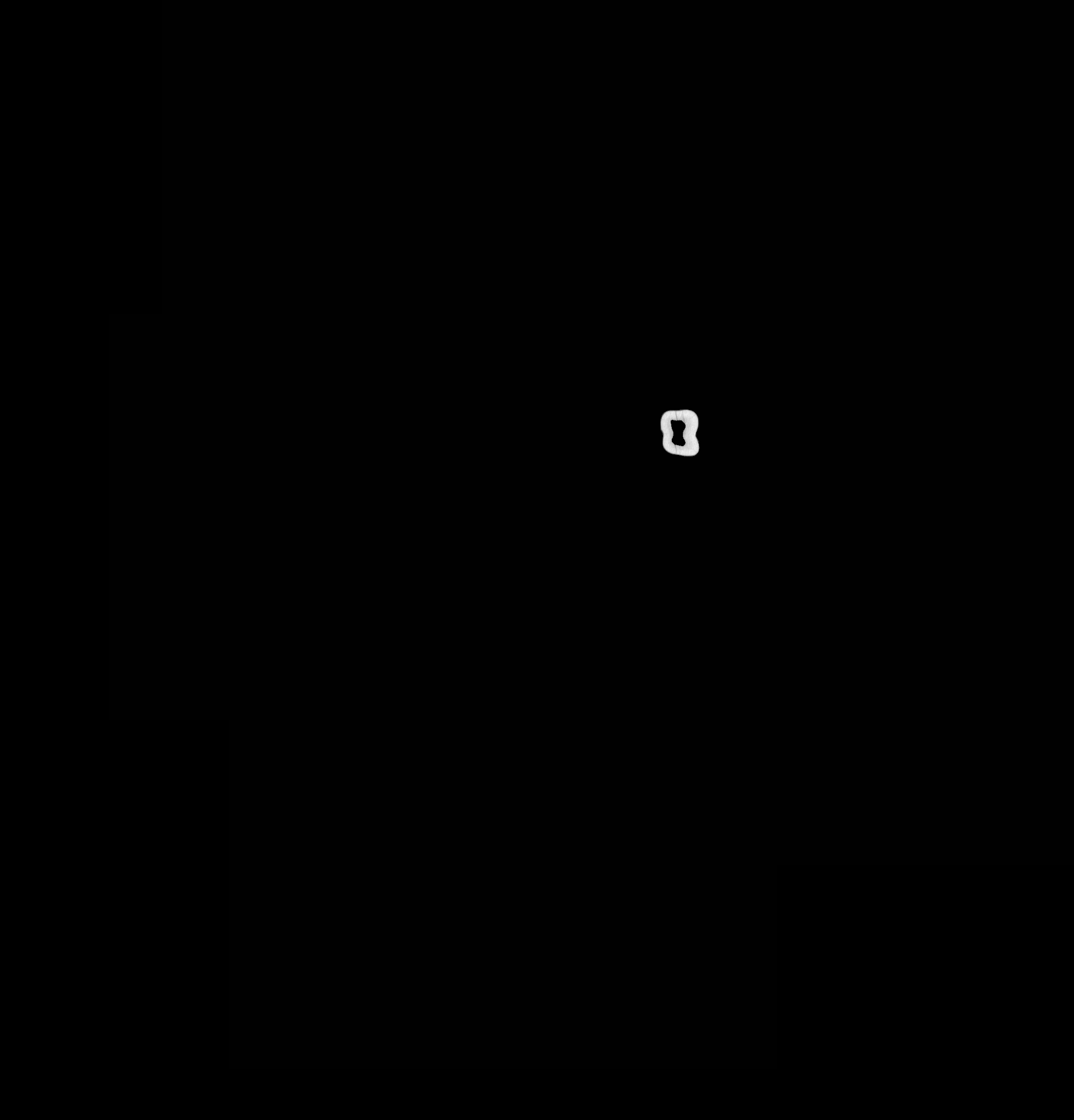

Supplement: Supplementary file 2 — Data S2: Supporting Information. [file AJPA-188-e70164-s001.zip › Cross-Section Tiff Files/amnh_52635_Rm1.tif]

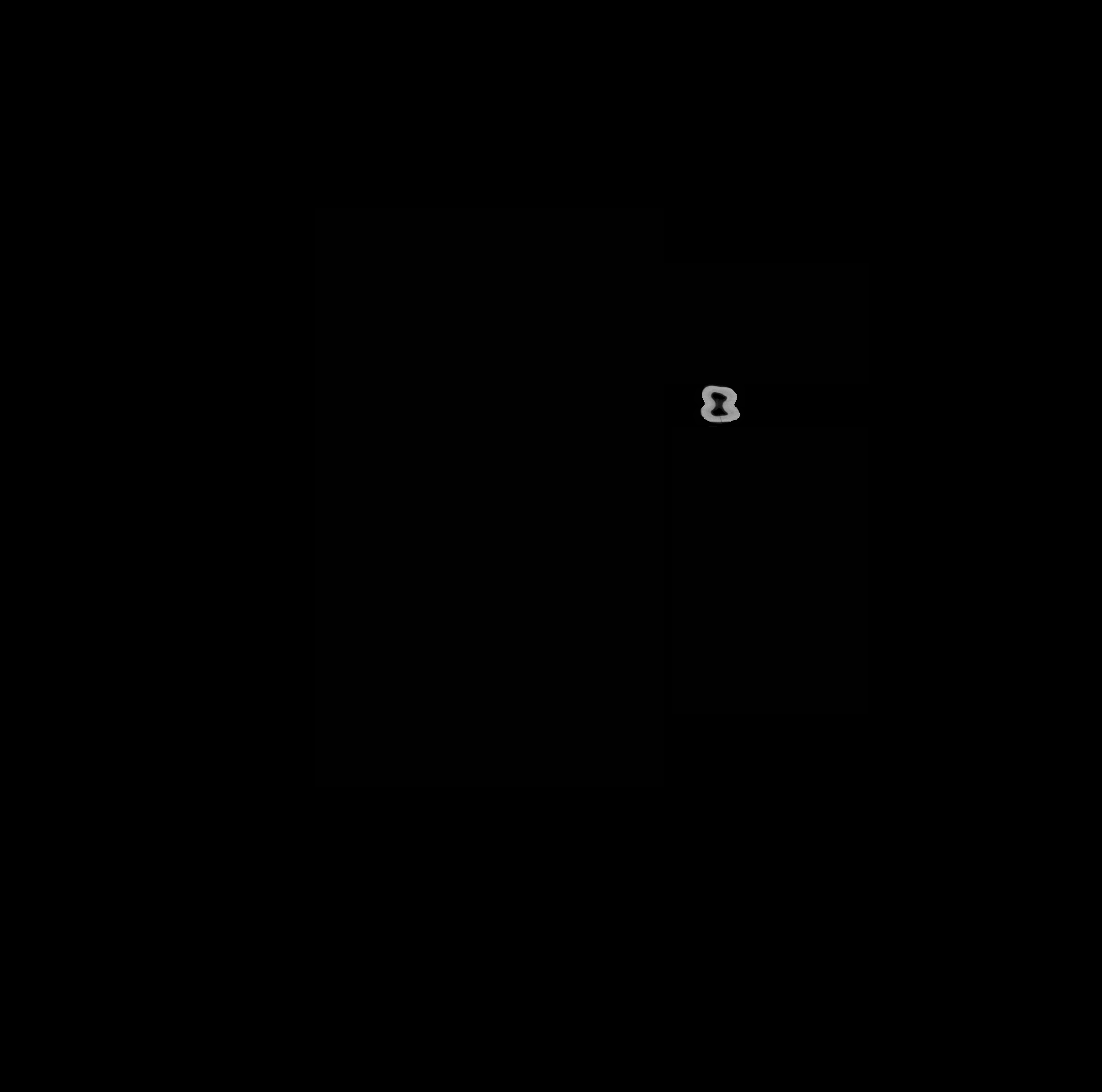

Supplement: Supplementary file 2 — Data S2: Supporting Information. [file AJPA-188-e70164-s001.zip › Cross-Section Tiff Files/amnh_19549_Rm1.tif]

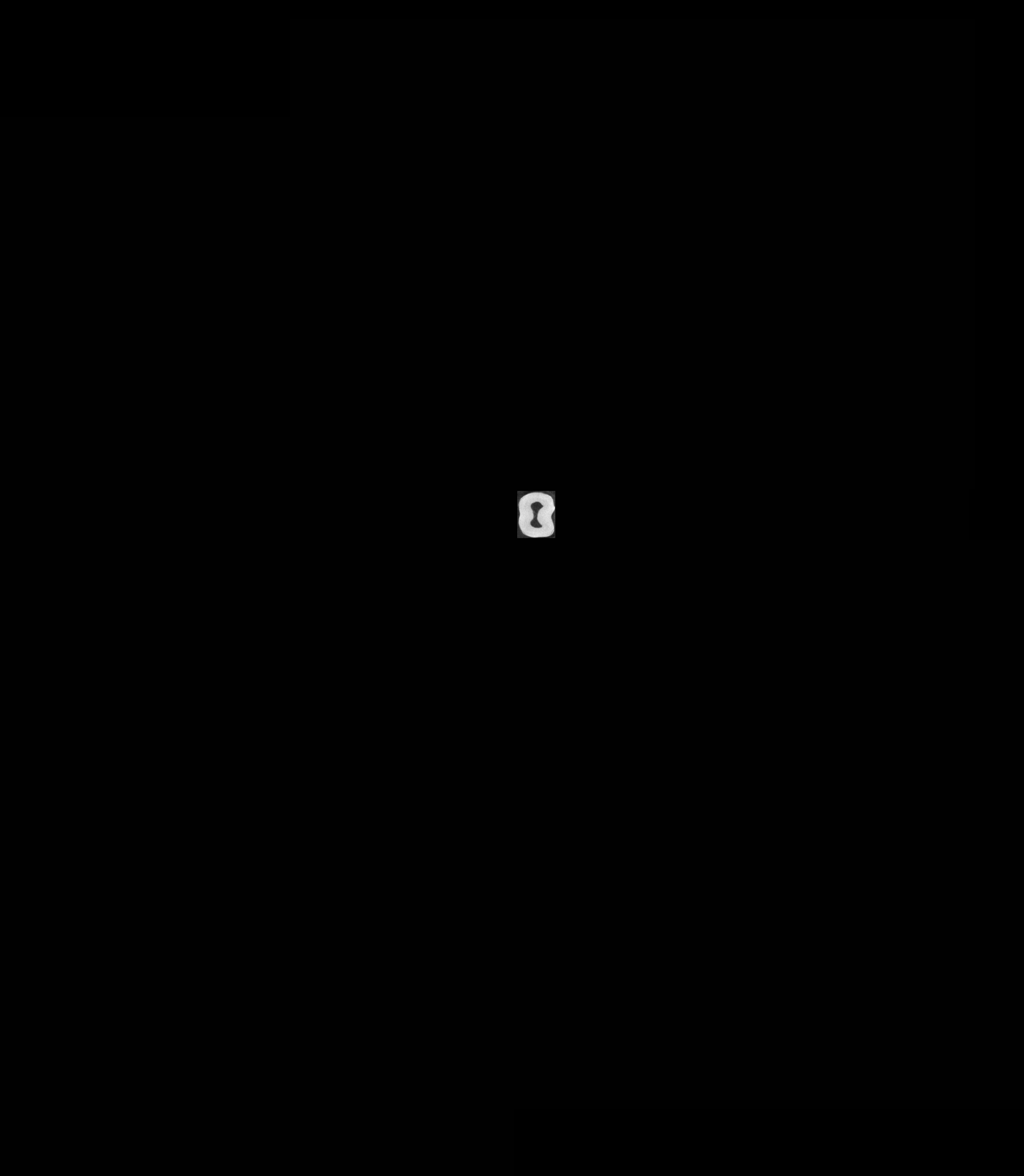

Supplement: Supplementary file 2 — Data S2: Supporting Information. [file AJPA-188-e70164-s001.zip › Cross-Section Tiff Files/amnh_52215_Rm2.tif]

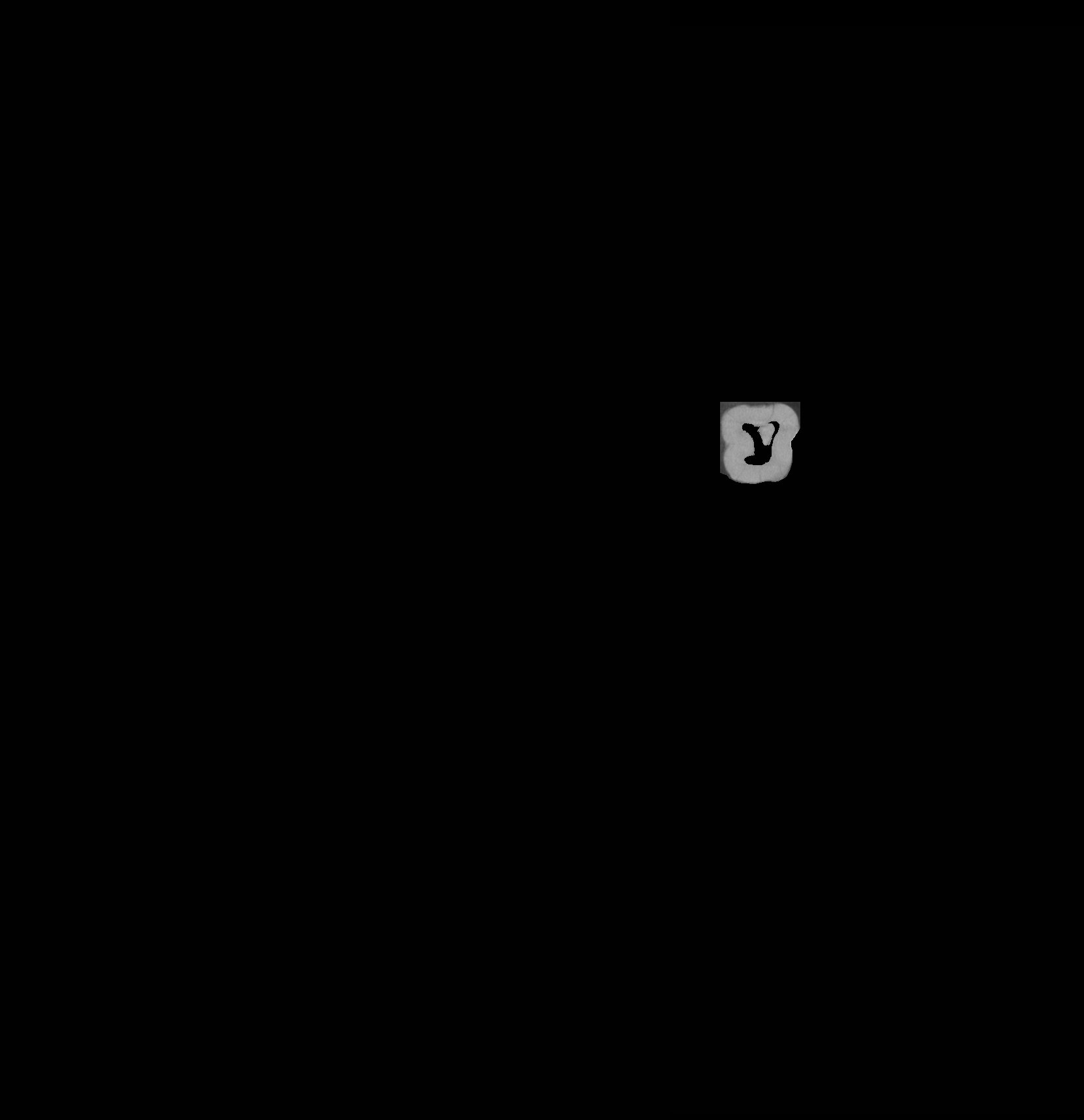

Supplement: Supplementary file 2 — Data S2: Supporting Information. [file AJPA-188-e70164-s001.zip › Cross-Section Tiff Files/mcz_37519_Rm1.tif]

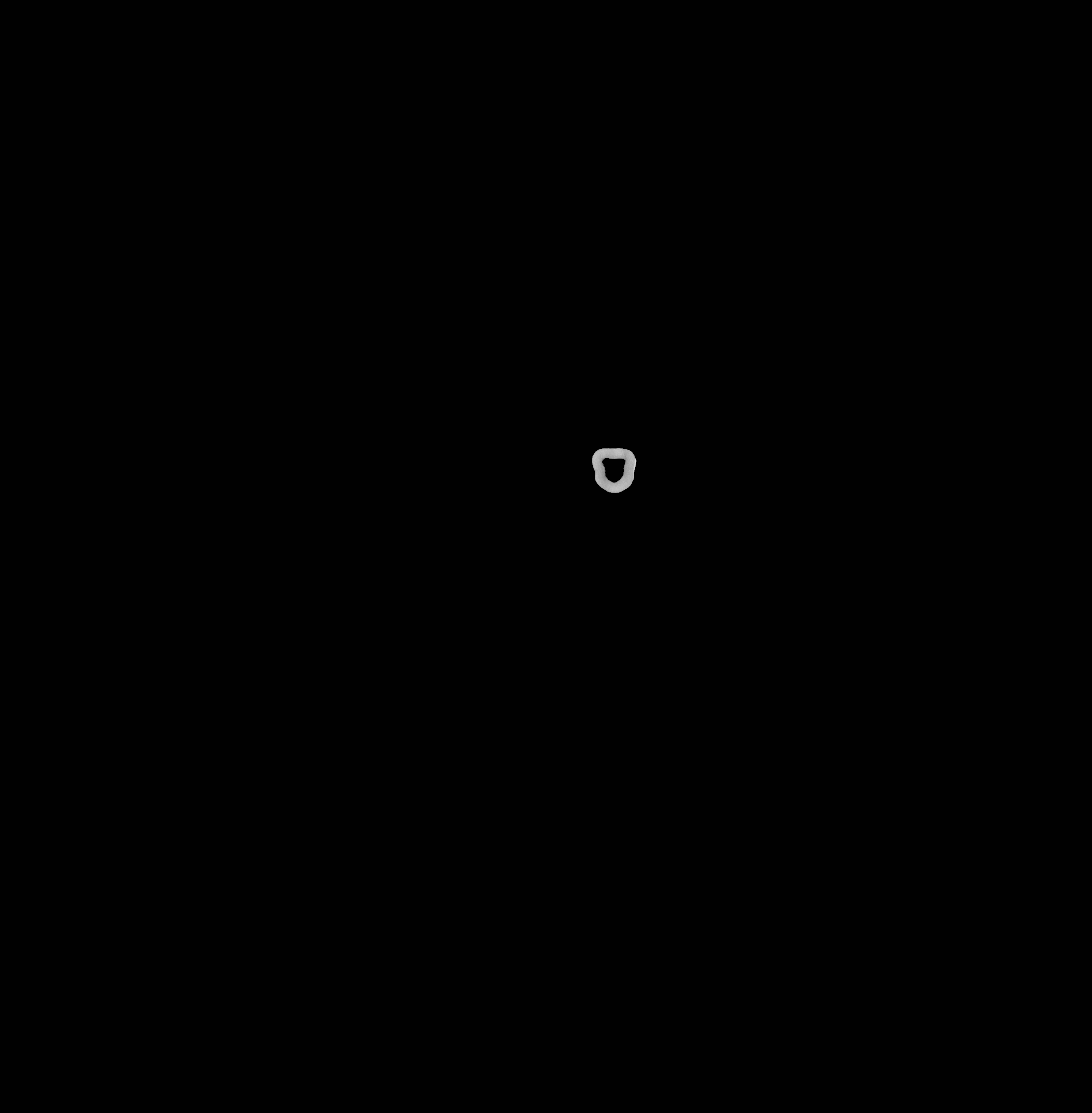

Supplement: Supplementary file 2 — Data S2: Supporting Information. [file AJPA-188-e70164-s001.zip › Cross-Section Tiff Files/mcz_50960_Rm2.tif]

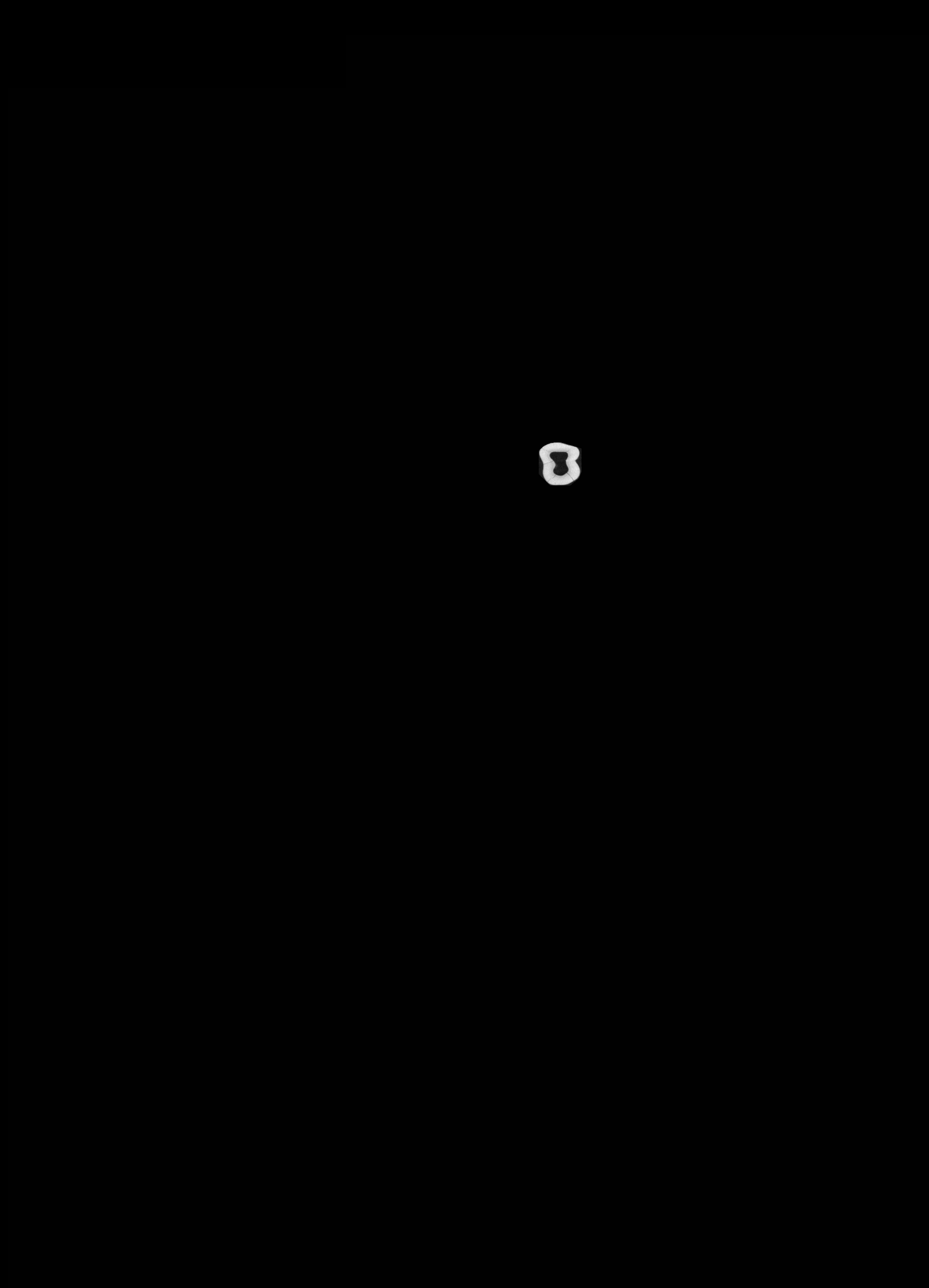

Supplement: Supplementary file 2 — Data S2: Supporting Information. [file AJPA-188-e70164-s001.zip › Cross-Section Tiff Files/amnh_167342_Rm3.tif]

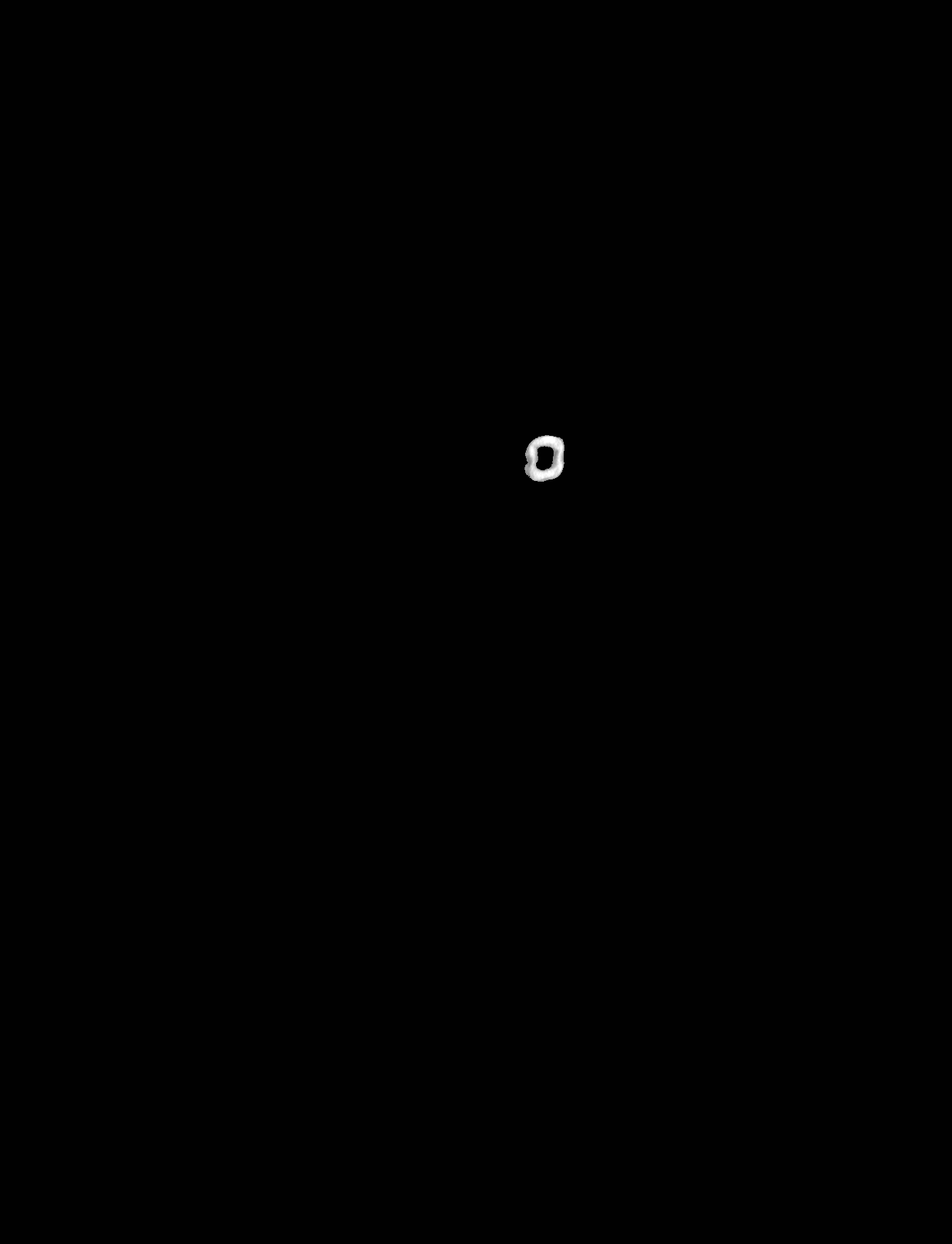

Supplement: Supplementary file 2 — Data S2: Supporting Information. [file AJPA-188-e70164-s001.zip › Cross-Section Tiff Files/mcz_41460_Rm3.tif]

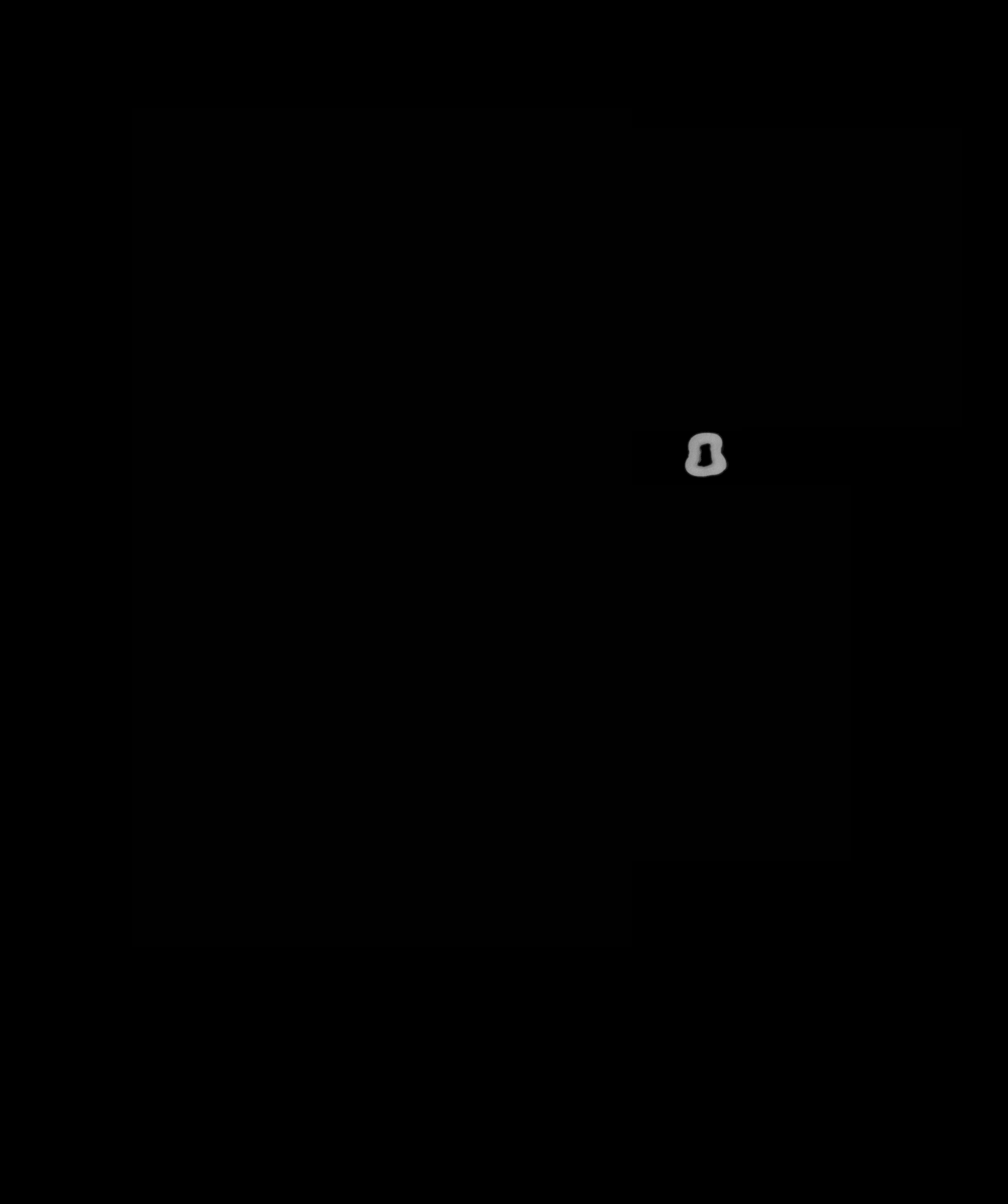

Supplement: Supplementary file 2 — Data S2: Supporting Information. [file AJPA-188-e70164-s001.zip › Cross-Section Tiff Files/amnh_102724_Rm1.tif]

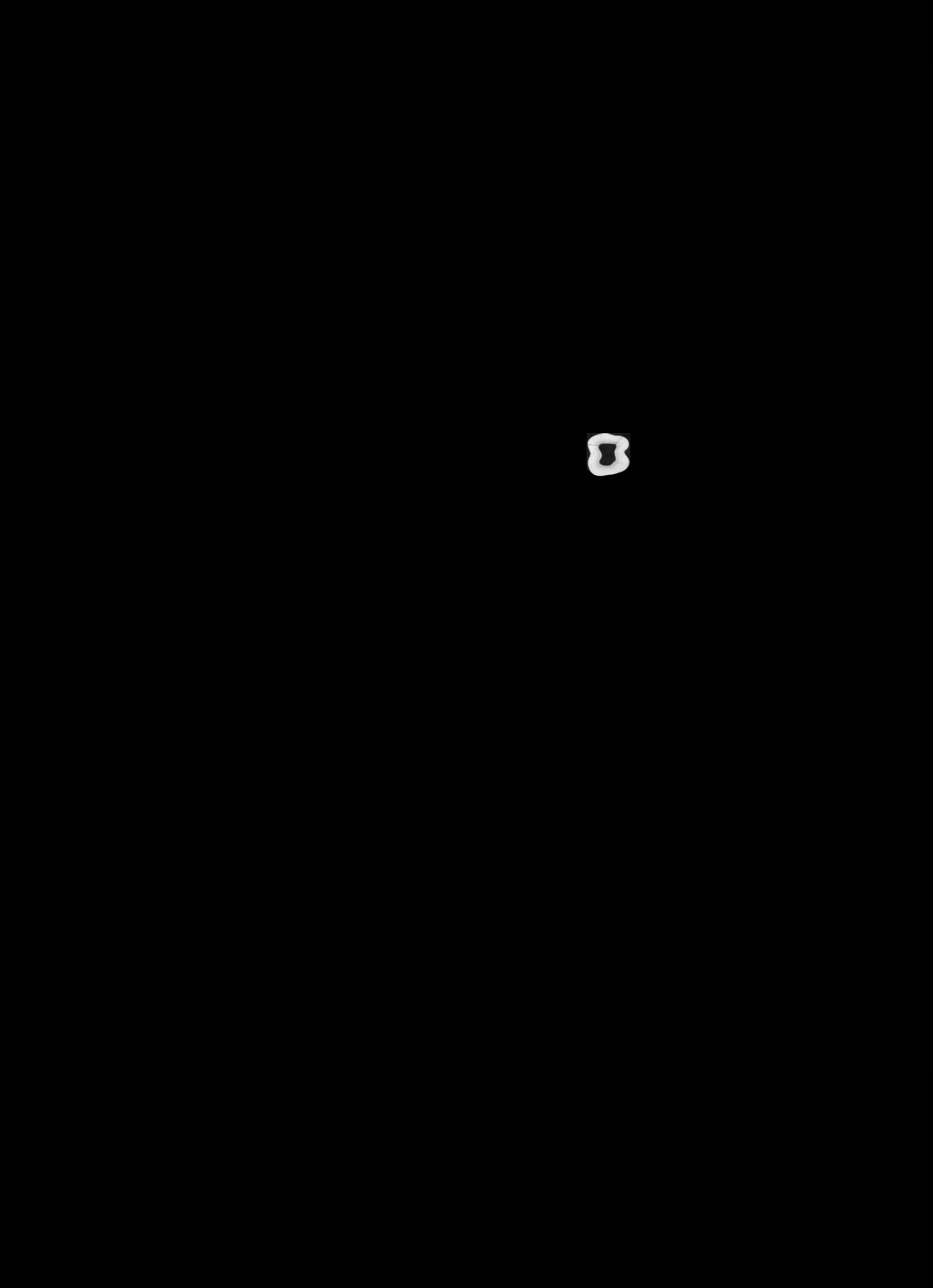

Supplement: Supplementary file 2 — Data S2: Supporting Information. [file AJPA-188-e70164-s001.zip › Cross-Section Tiff Files/amnh_167342_Rm2.tif]

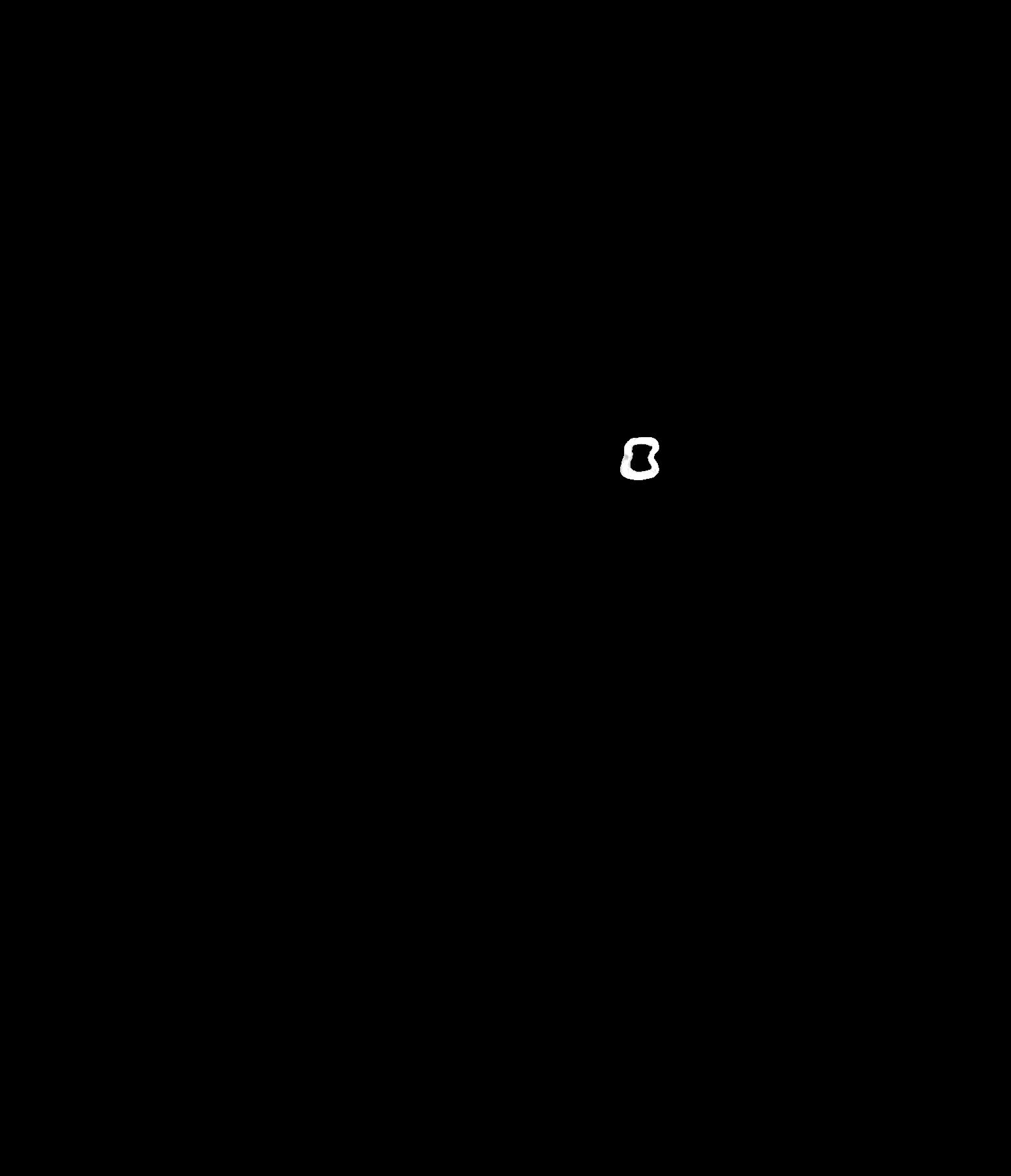

Supplement: Supplementary file 2 — Data S2: Supporting Information. [file AJPA-188-e70164-s001.zip › Cross-Section Tiff Files/mcz_41460_Rm2.tif]

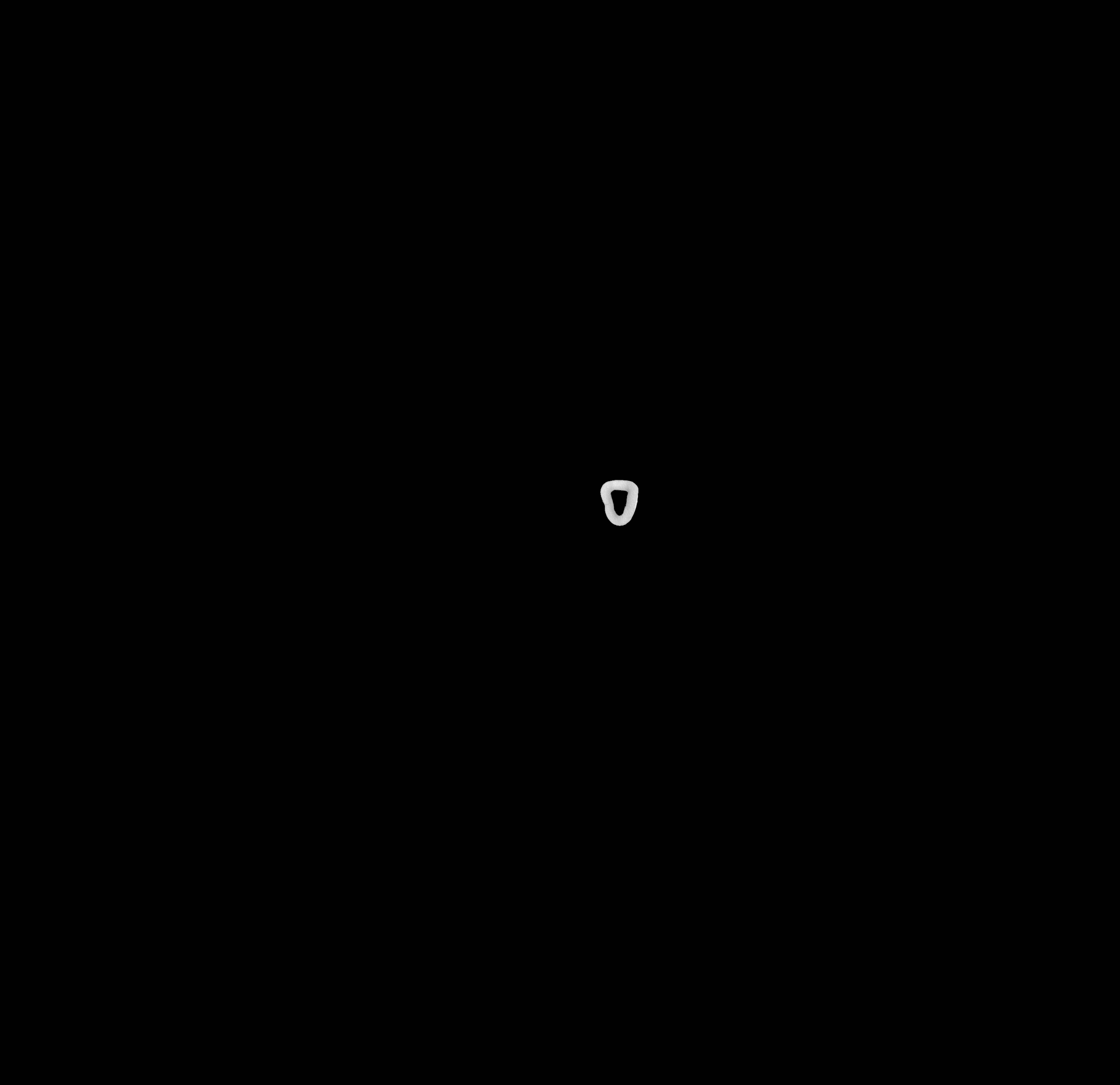

Supplement: Supplementary file 2 — Data S2: Supporting Information. [file AJPA-188-e70164-s001.zip › Cross-Section Tiff Files/mcz_50960_Rm3.tif]

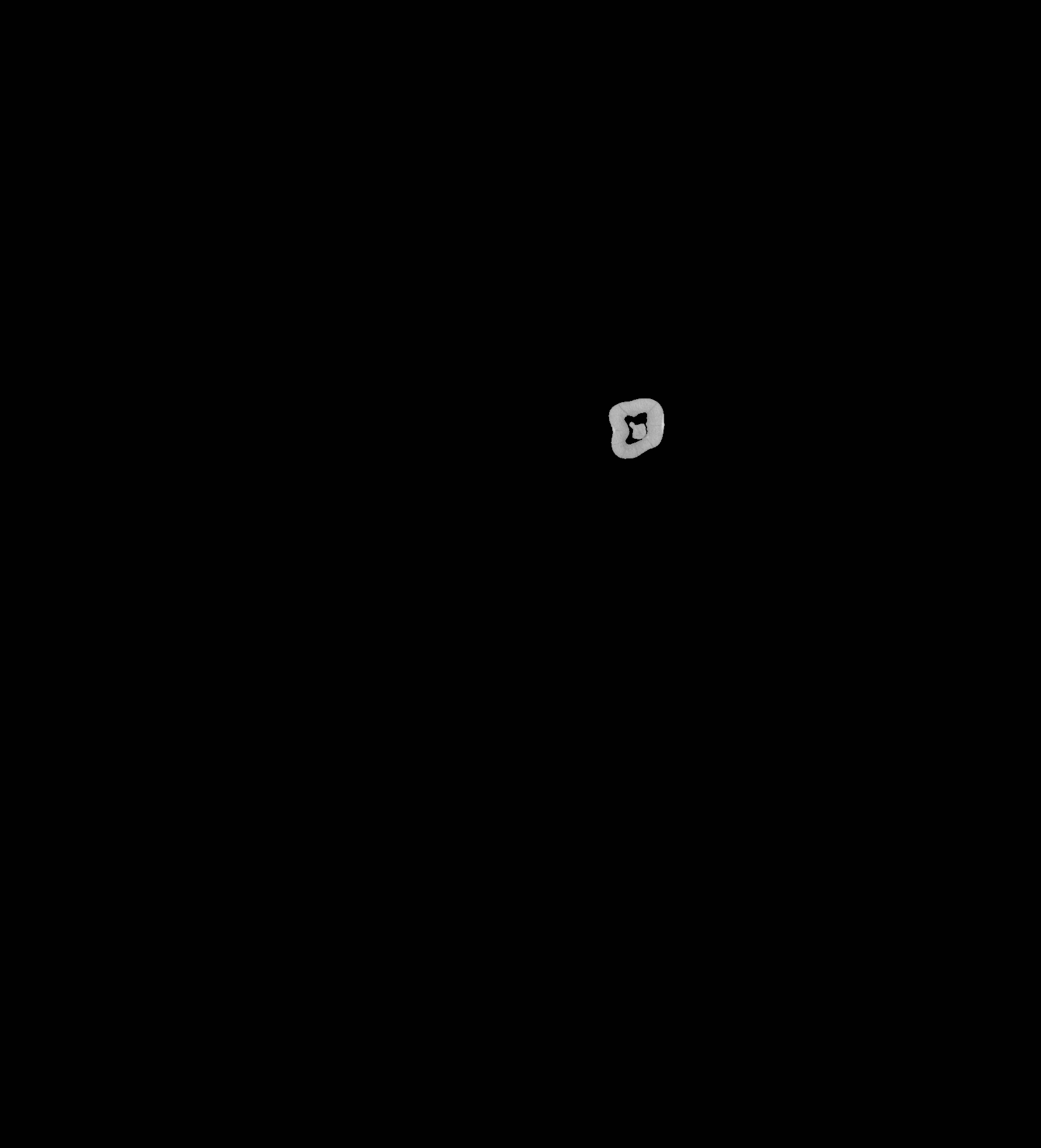

Supplement: Supplementary file 2 — Data S2: Supporting Information. [file AJPA-188-e70164-s001.zip › Cross-Section Tiff Files/mcz_37363_Rm1.tif]

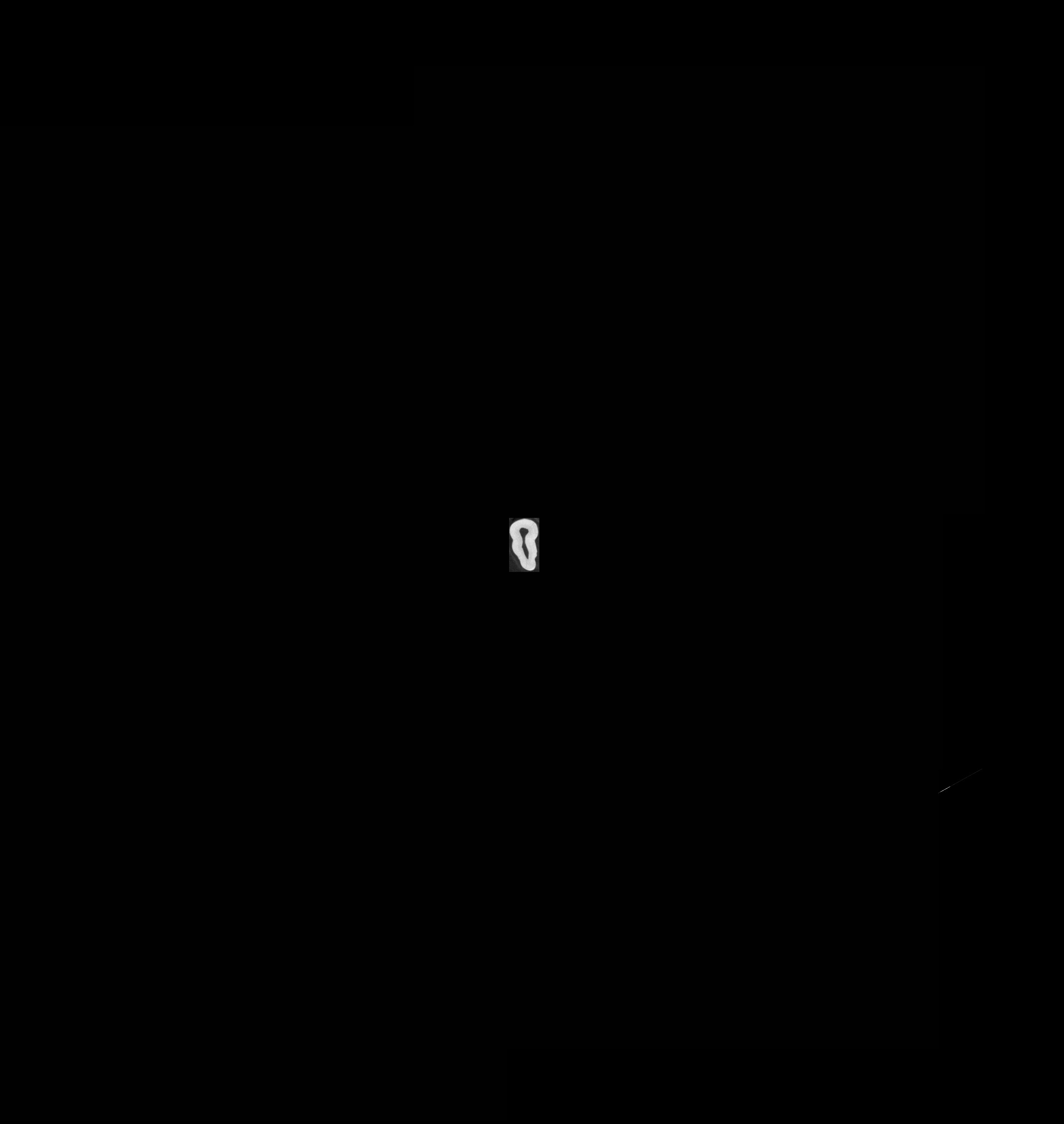

Supplement: Supplementary file 2 — Data S2: Supporting Information. [file AJPA-188-e70164-s001.zip › Cross-Section Tiff Files/amnh_52215_Rm3.tif]

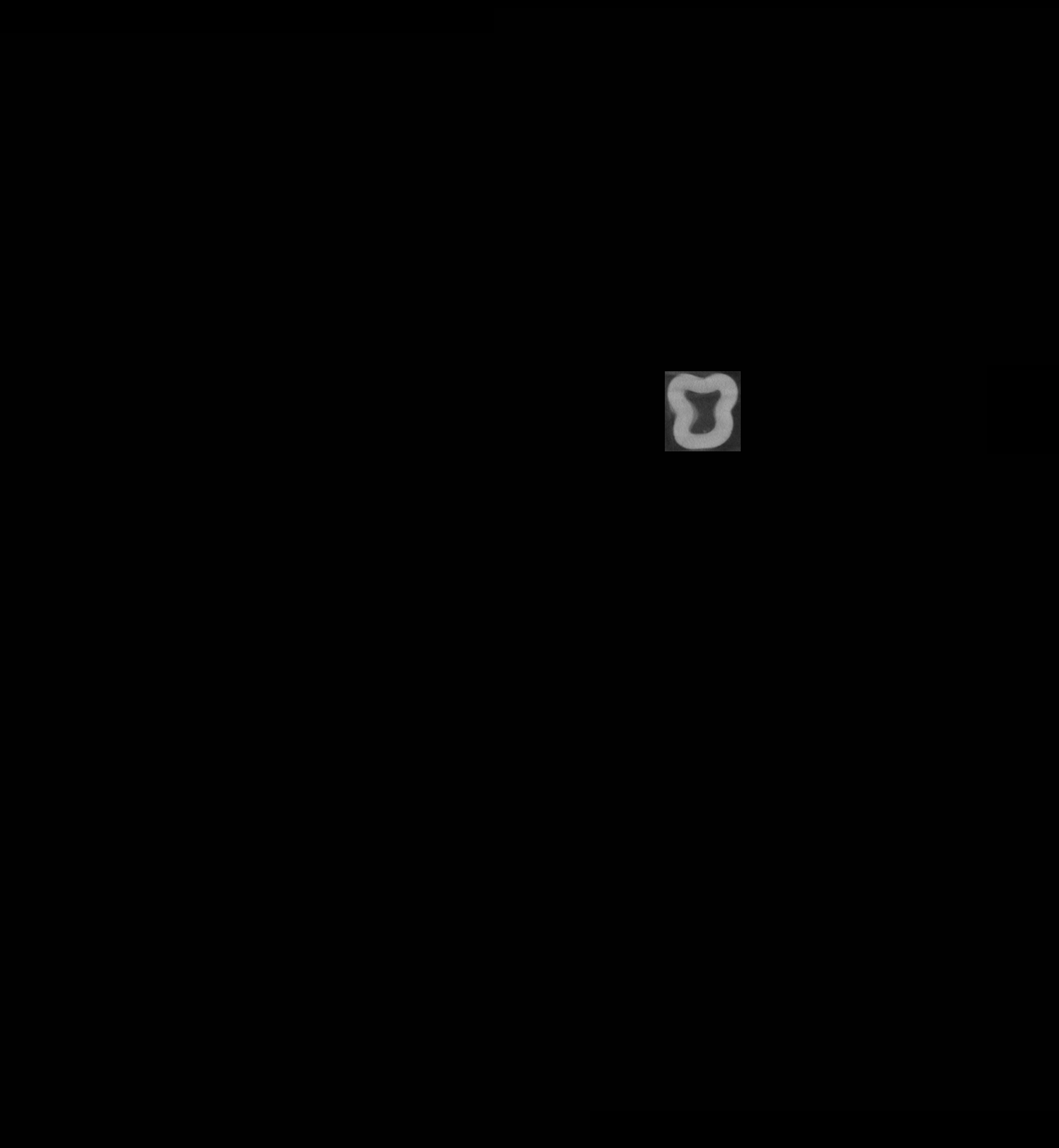

Supplement: Supplementary file 2 — Data S2: Supporting Information. [file AJPA-188-e70164-s001.zip › Cross-Section Tiff Files/mcz_37518_Rm1.tif]

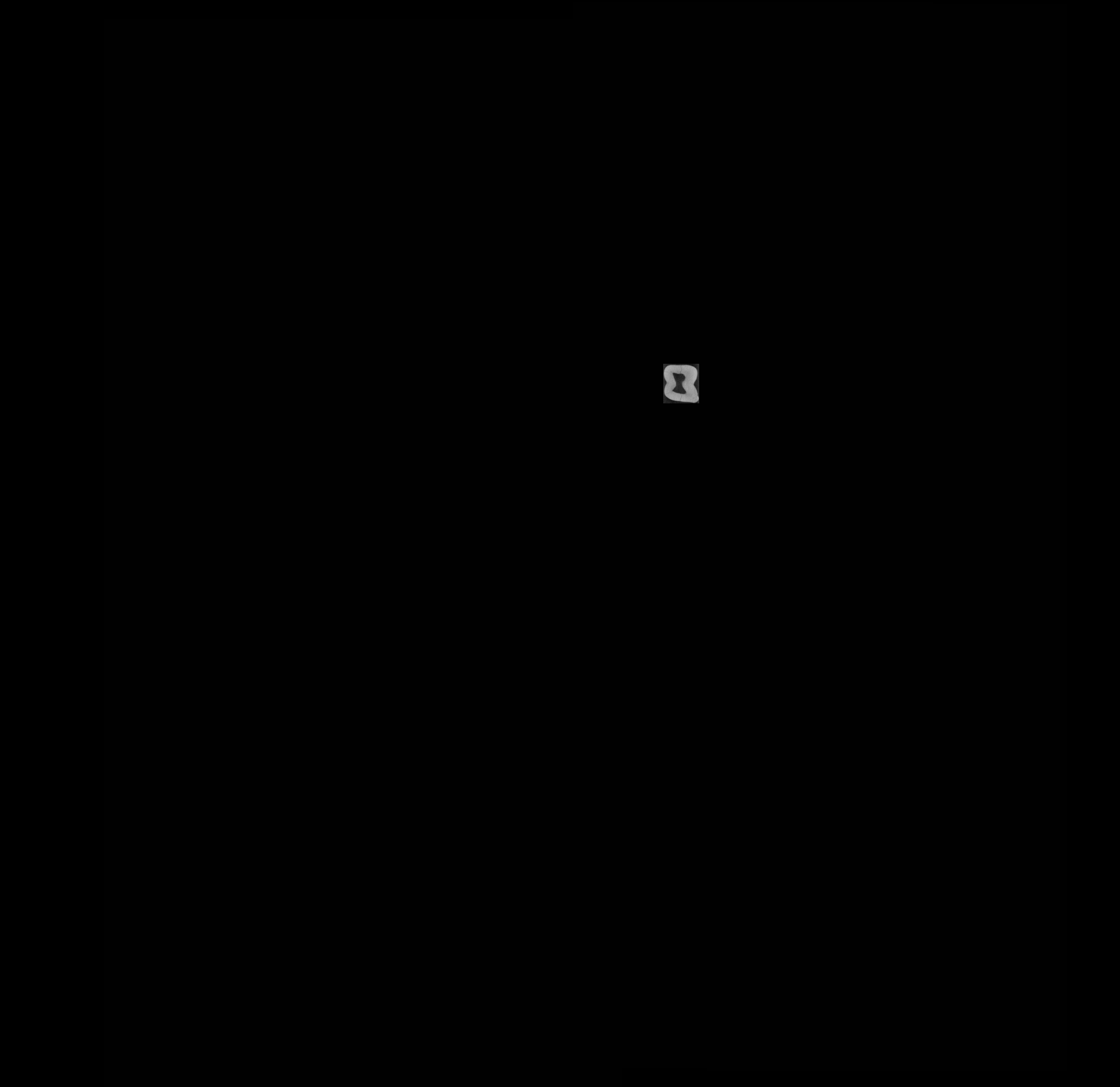

Supplement: Supplementary file 2 — Data S2: Supporting Information. [file AJPA-188-e70164-s001.zip › Cross-Section Tiff Files/amnh_52634_Rm1.tif]

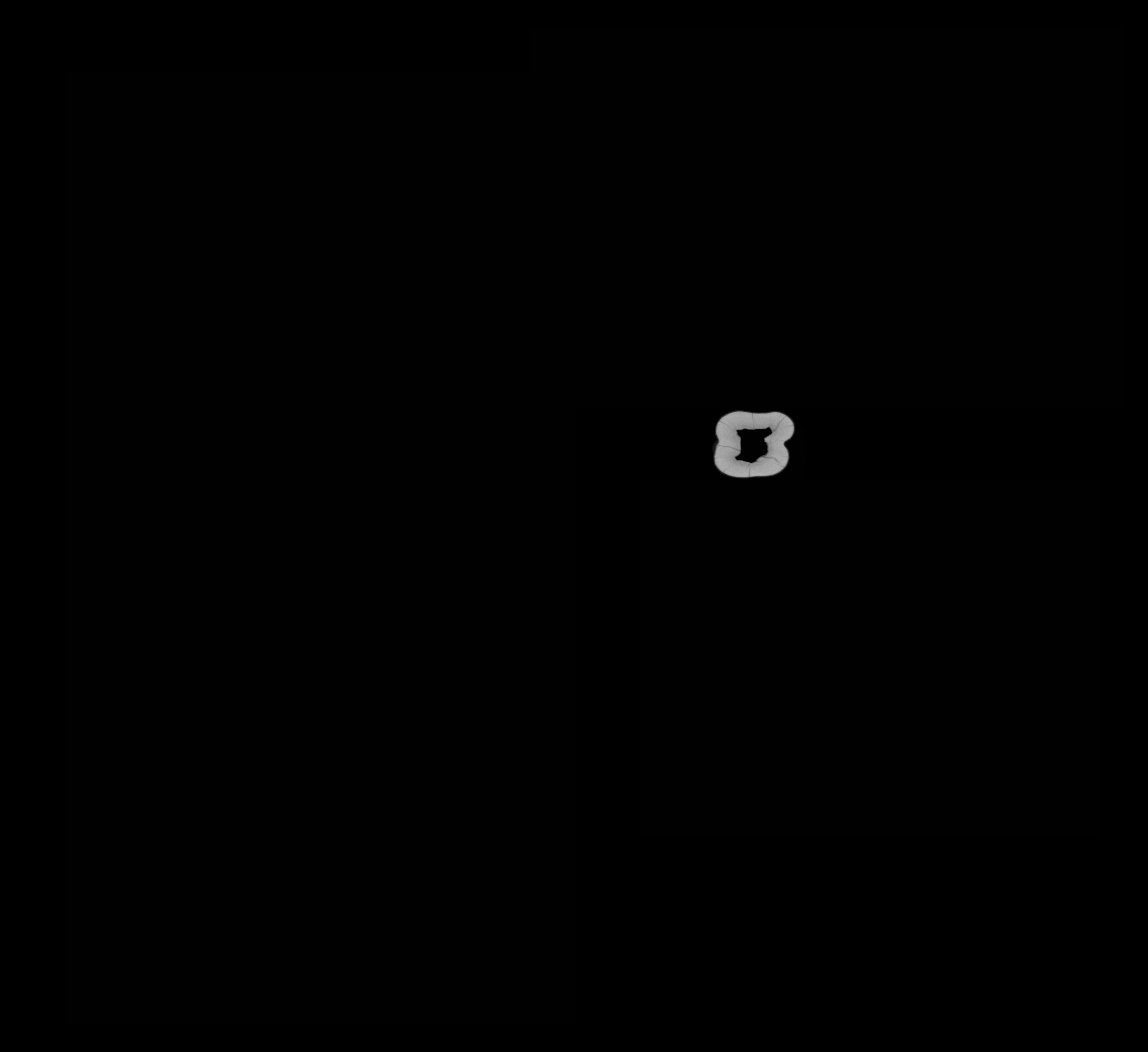

Supplement: Supplementary file 2 — Data S2: Supporting Information. [file AJPA-188-e70164-s001.zip › Cross-Section Tiff Files/amnh_AL223_Rm2.tif]

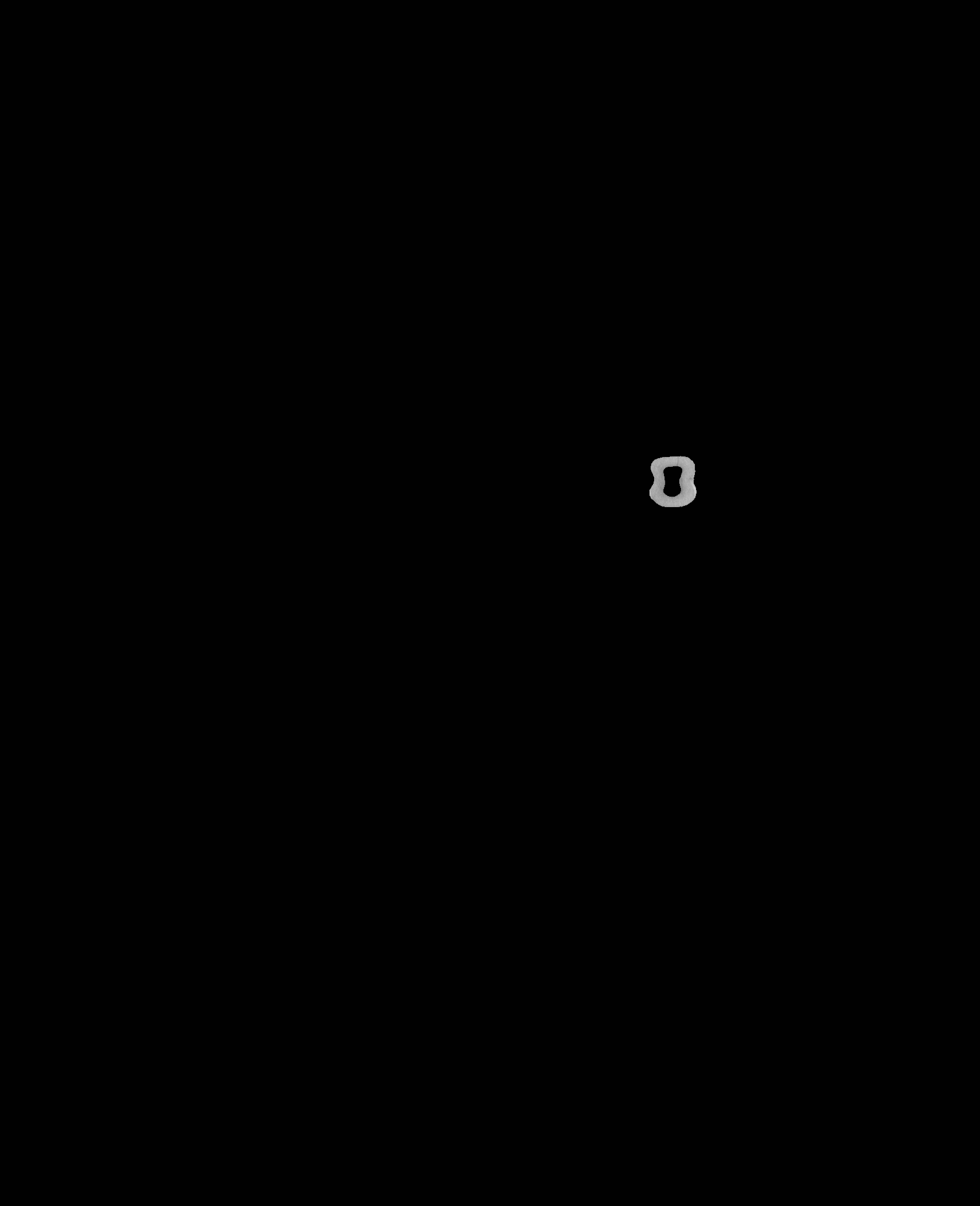

Supplement: Supplementary file 2 — Data S2: Supporting Information. [file AJPA-188-e70164-s001.zip › Cross-Section Tiff Files/mcz_23164_Rm1.tif]

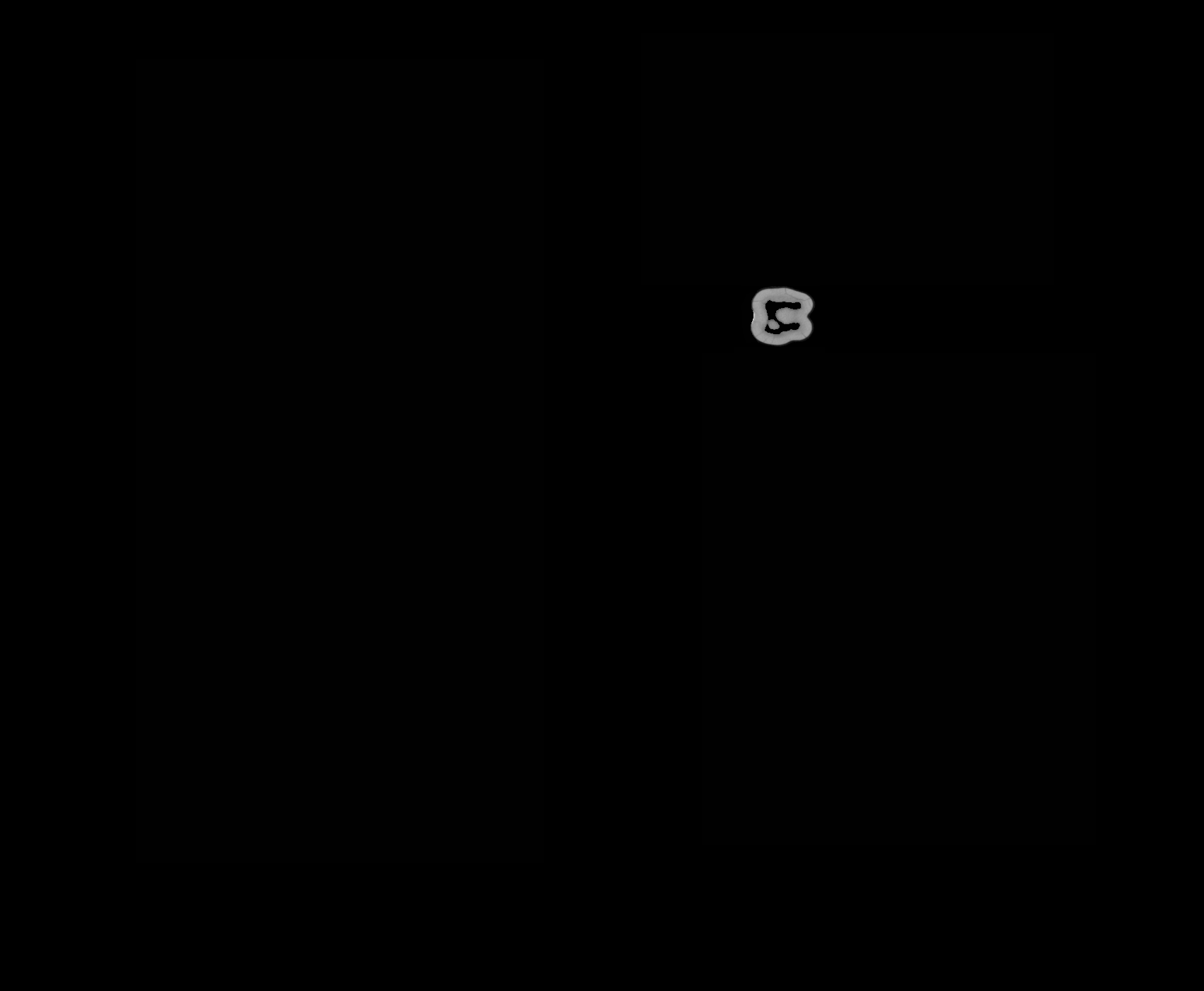

Supplement: Supplementary file 2 — Data S2: Supporting Information. [file AJPA-188-e70164-s001.zip › Cross-Section Tiff Files/amnh_167338_Rm1.tif]

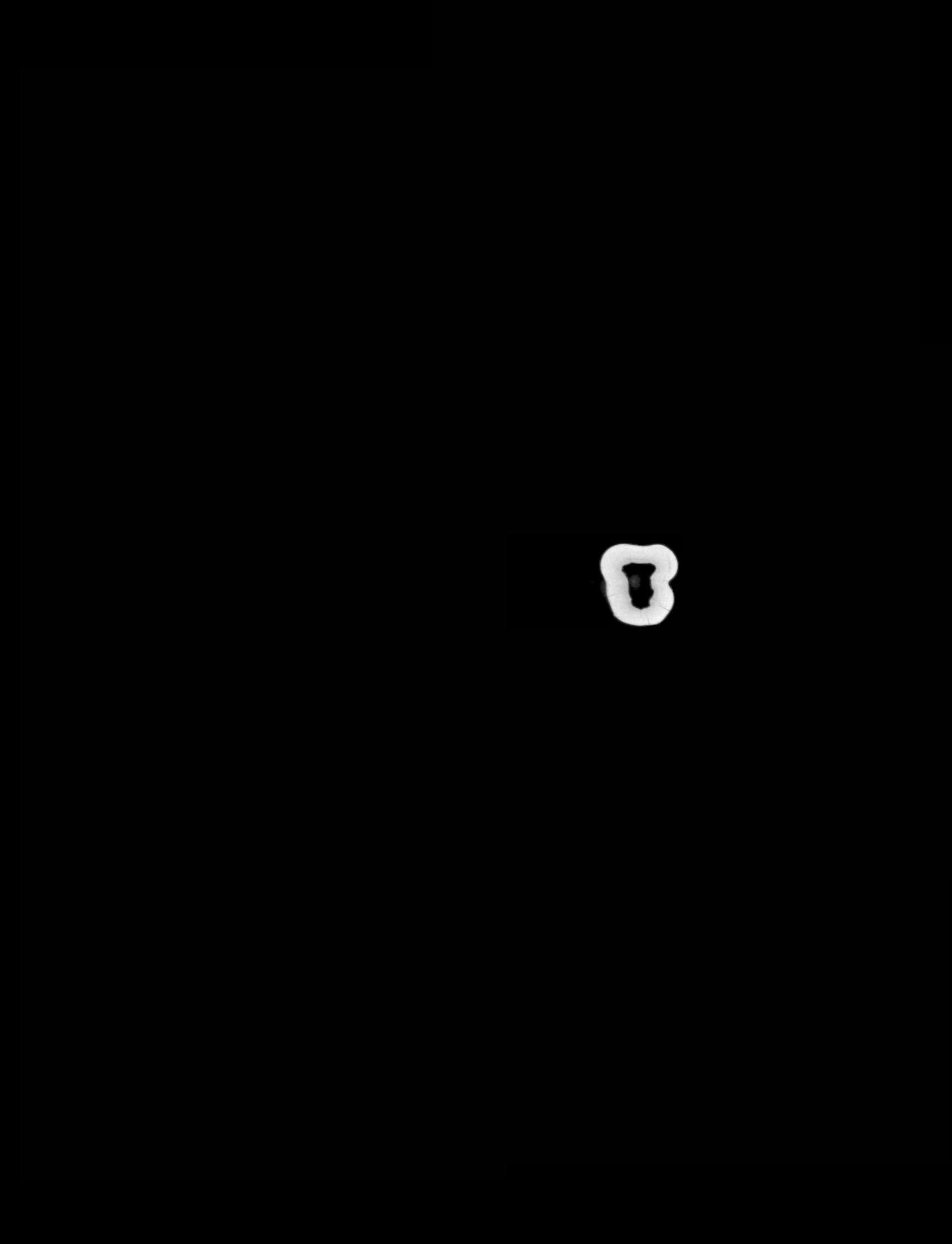

Supplement: Supplementary file 2 — Data S2: Supporting Information. [file AJPA-188-e70164-s001.zip › Cross-Section Tiff Files/amnh_AL223_Rm3.tif]

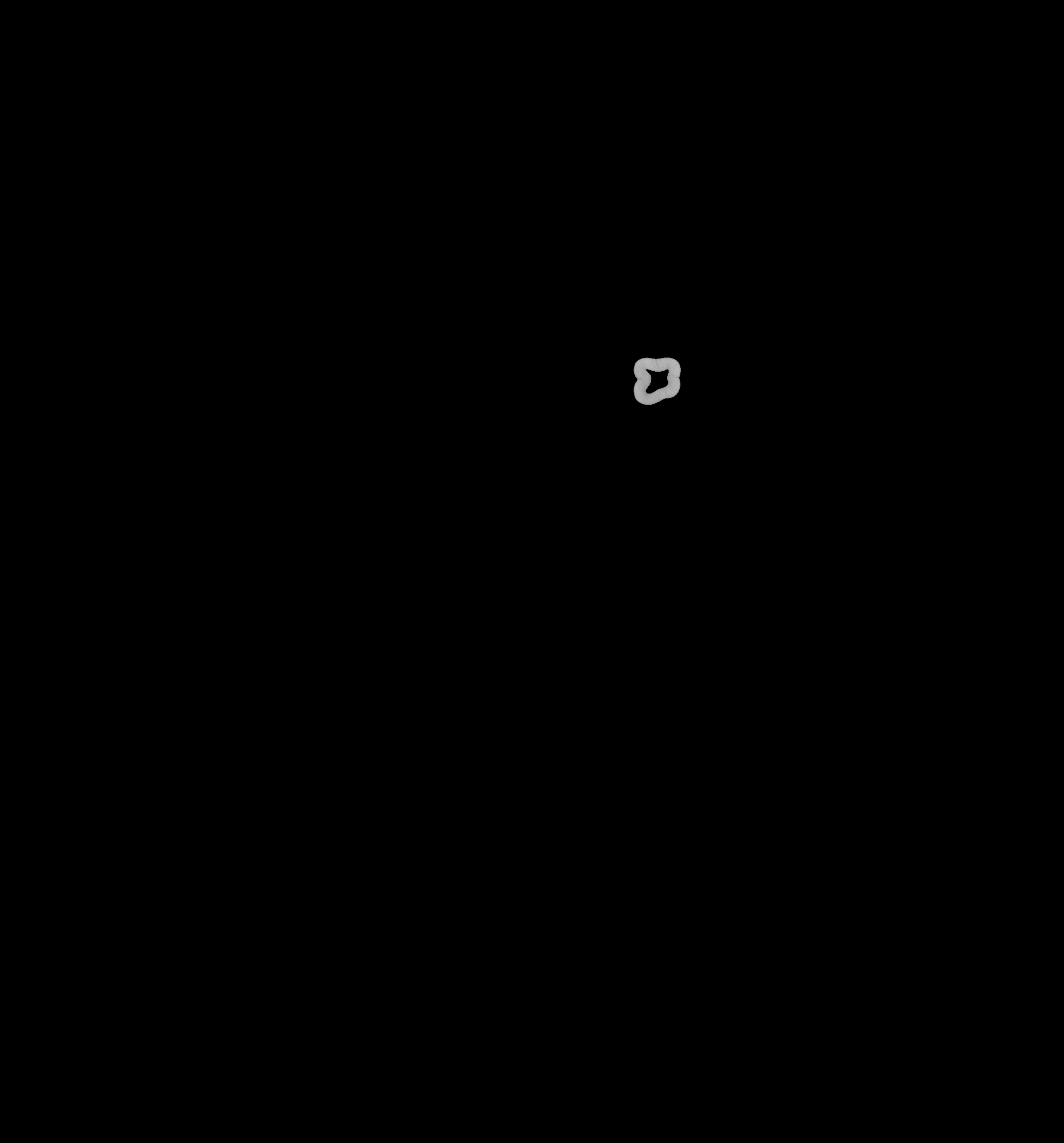

Supplement: Supplementary file 2 — Data S2: Supporting Information. [file AJPA-188-e70164-s001.zip › Cross-Section Tiff Files/mcz_37362_Rm1.tif]

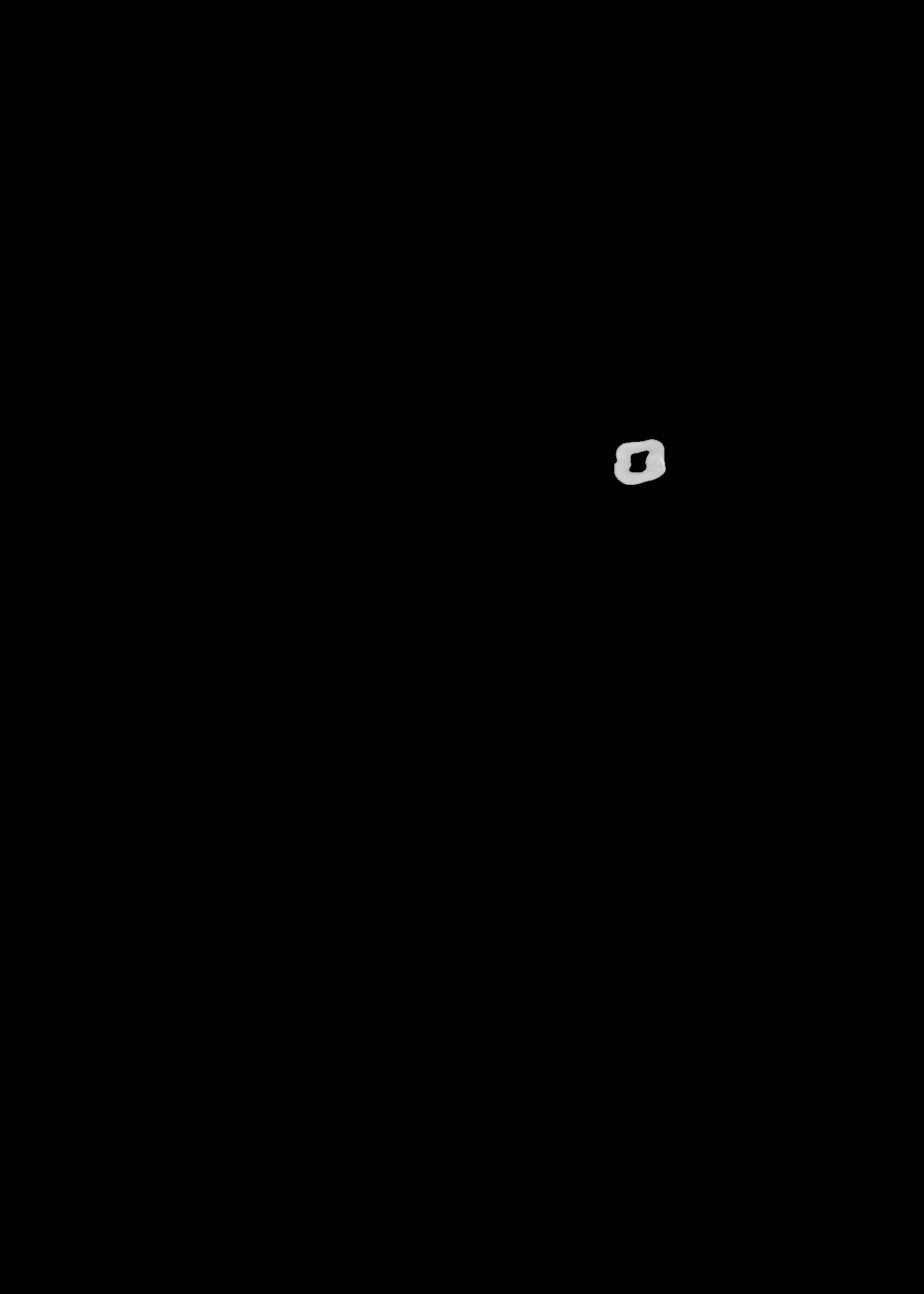

Supplement: Supplementary file 2 — Data S2: Supporting Information. [file AJPA-188-e70164-s001.zip › Cross-Section Tiff Files/mcz_BOM9493_Rm1.tif]

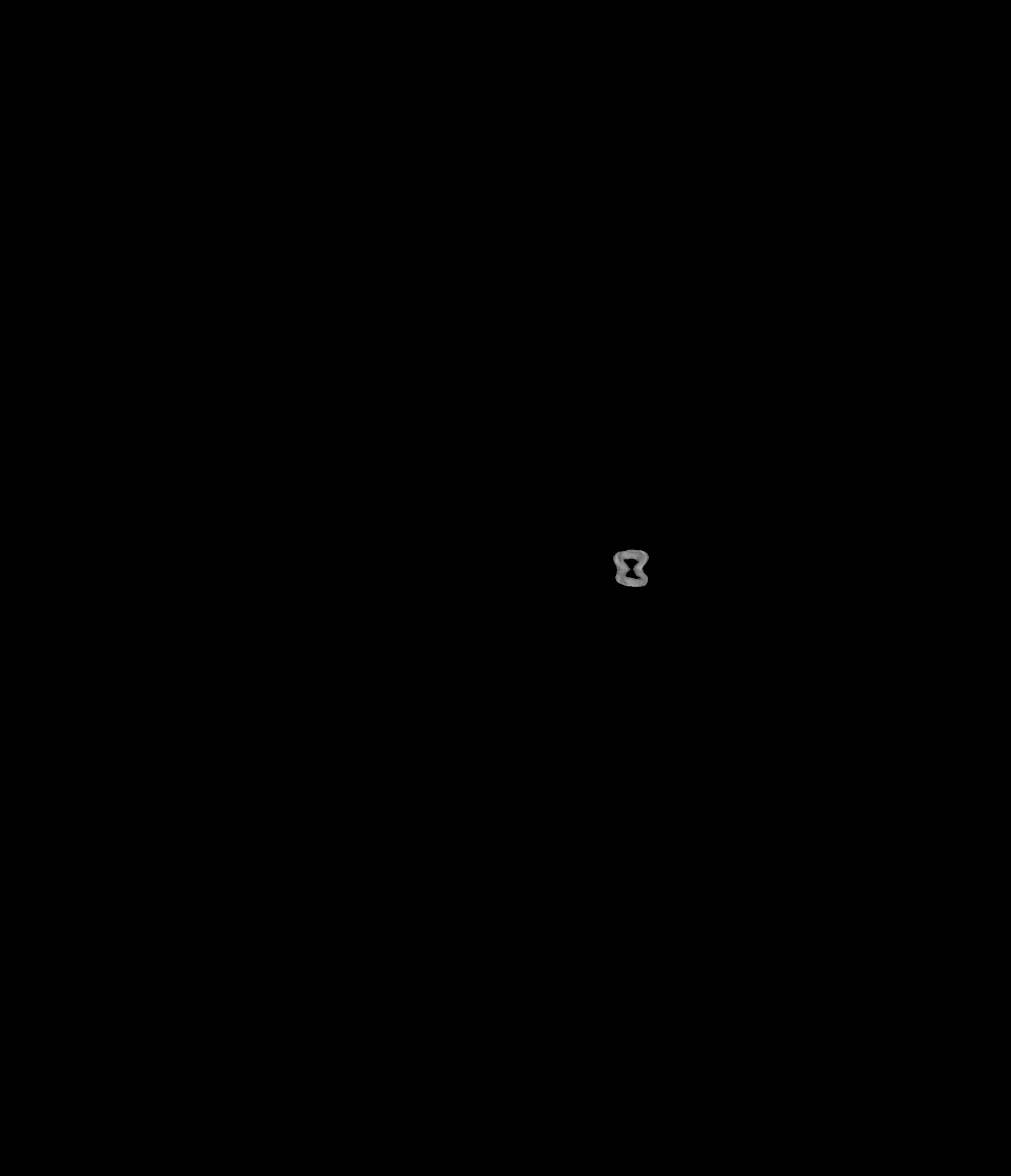

Supplement: Supplementary file 2 — Data S2: Supporting Information. [file AJPA-188-e70164-s001.zip › Cross-Section Tiff Files/mcz_37278_Rm1.tif]

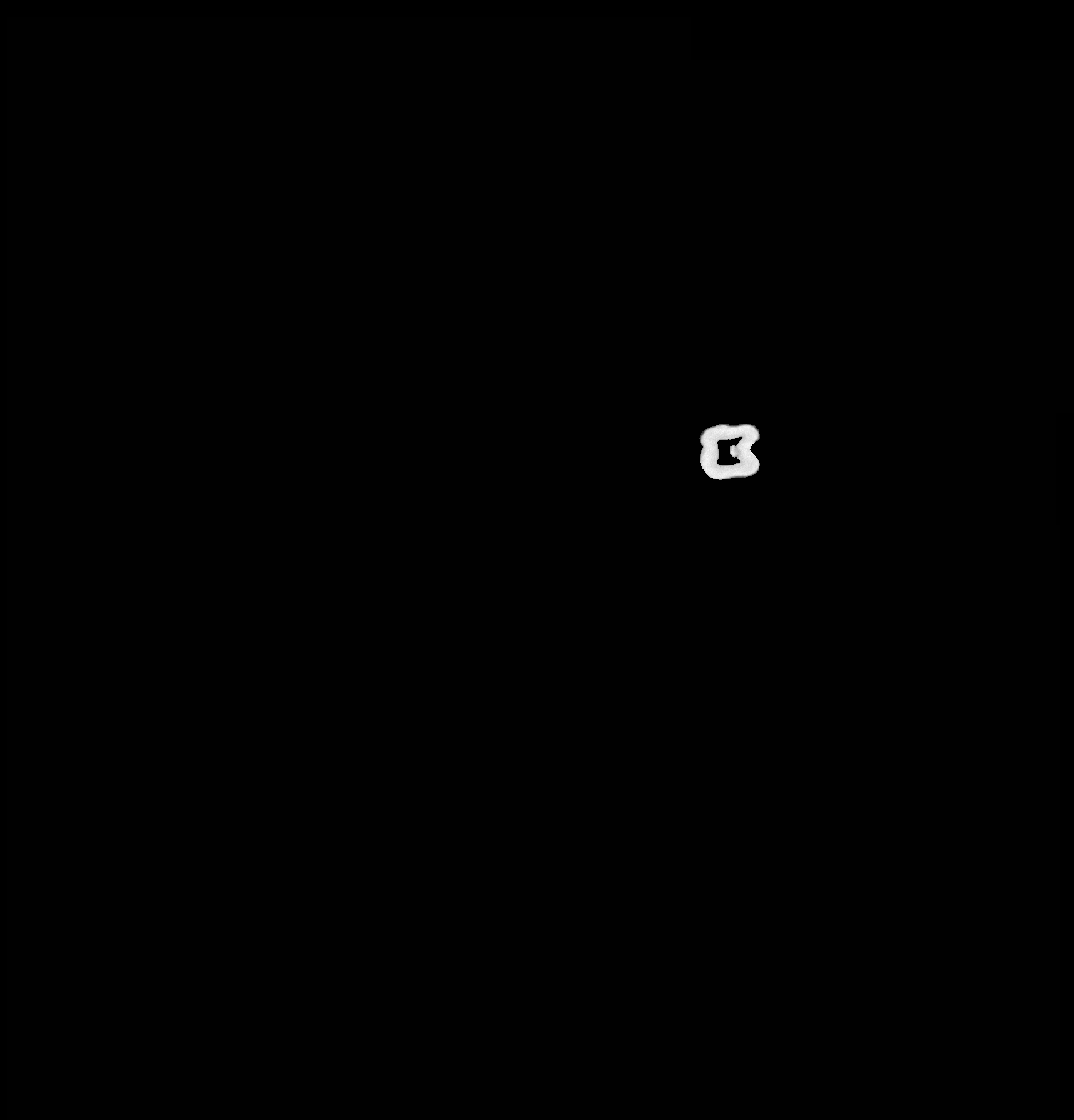

Supplement: Supplementary file 2 — Data S2: Supporting Information. [file AJPA-188-e70164-s001.zip › Cross-Section Tiff Files/mcz_17702_Rm1.tif]

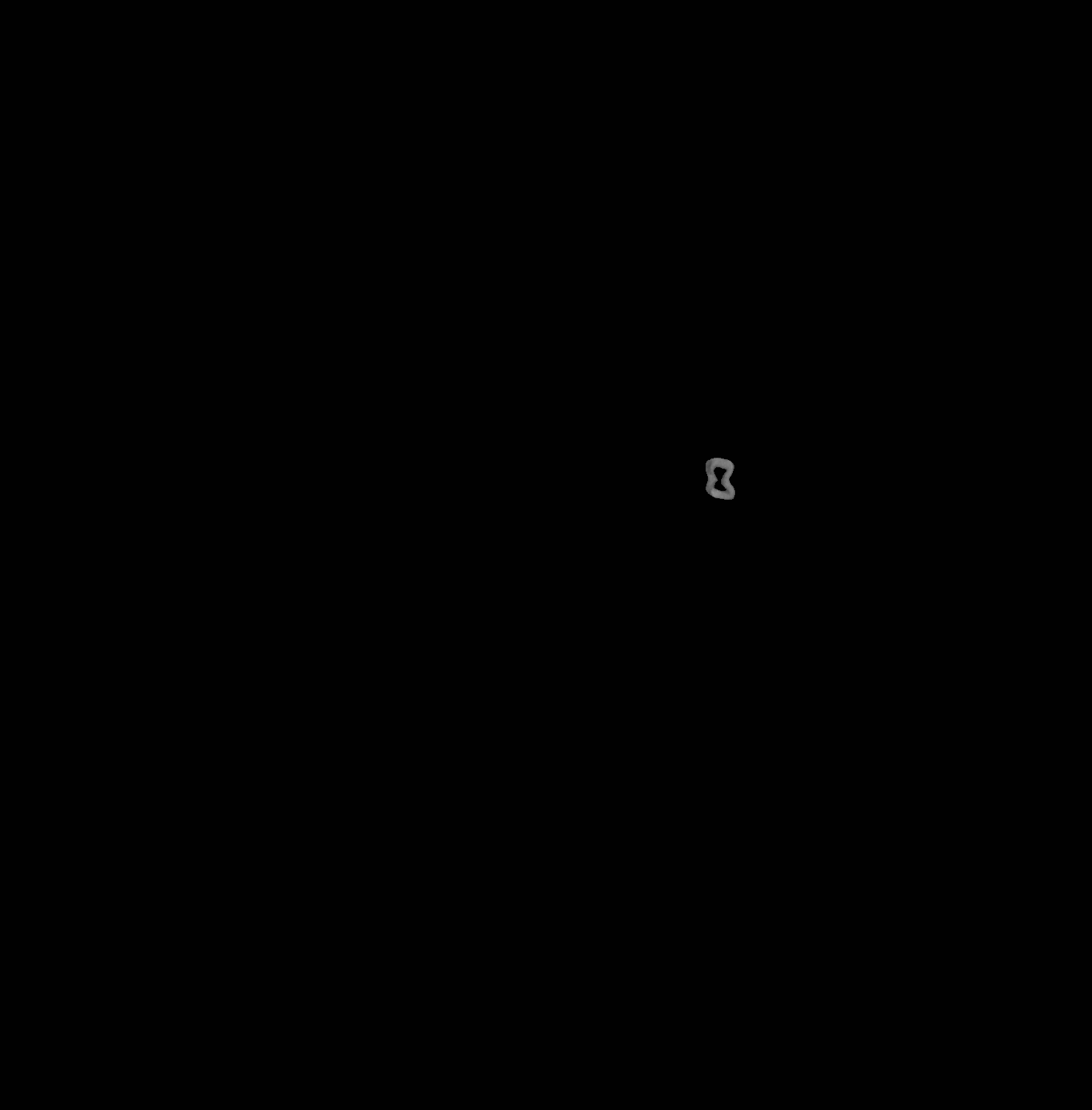

Supplement: Supplementary file 2 — Data S2: Supporting Information. [file AJPA-188-e70164-s001.zip › Cross-Section Tiff Files/mcz_34264_Rm1.tif]

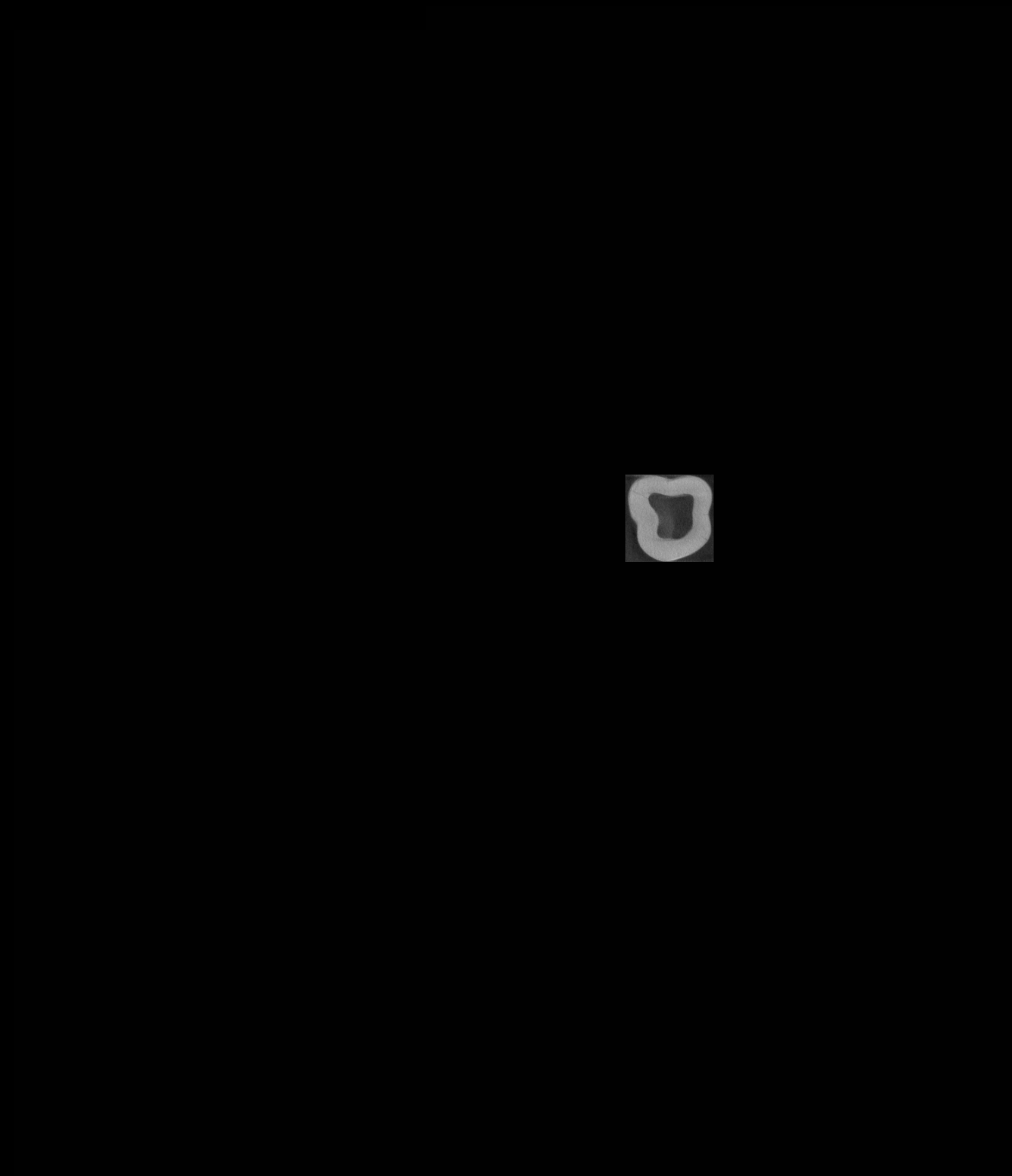

Supplement: Supplementary file 2 — Data S2: Supporting Information. [file AJPA-188-e70164-s001.zip › Cross-Section Tiff Files/mcz_37518_Rm2.tif]

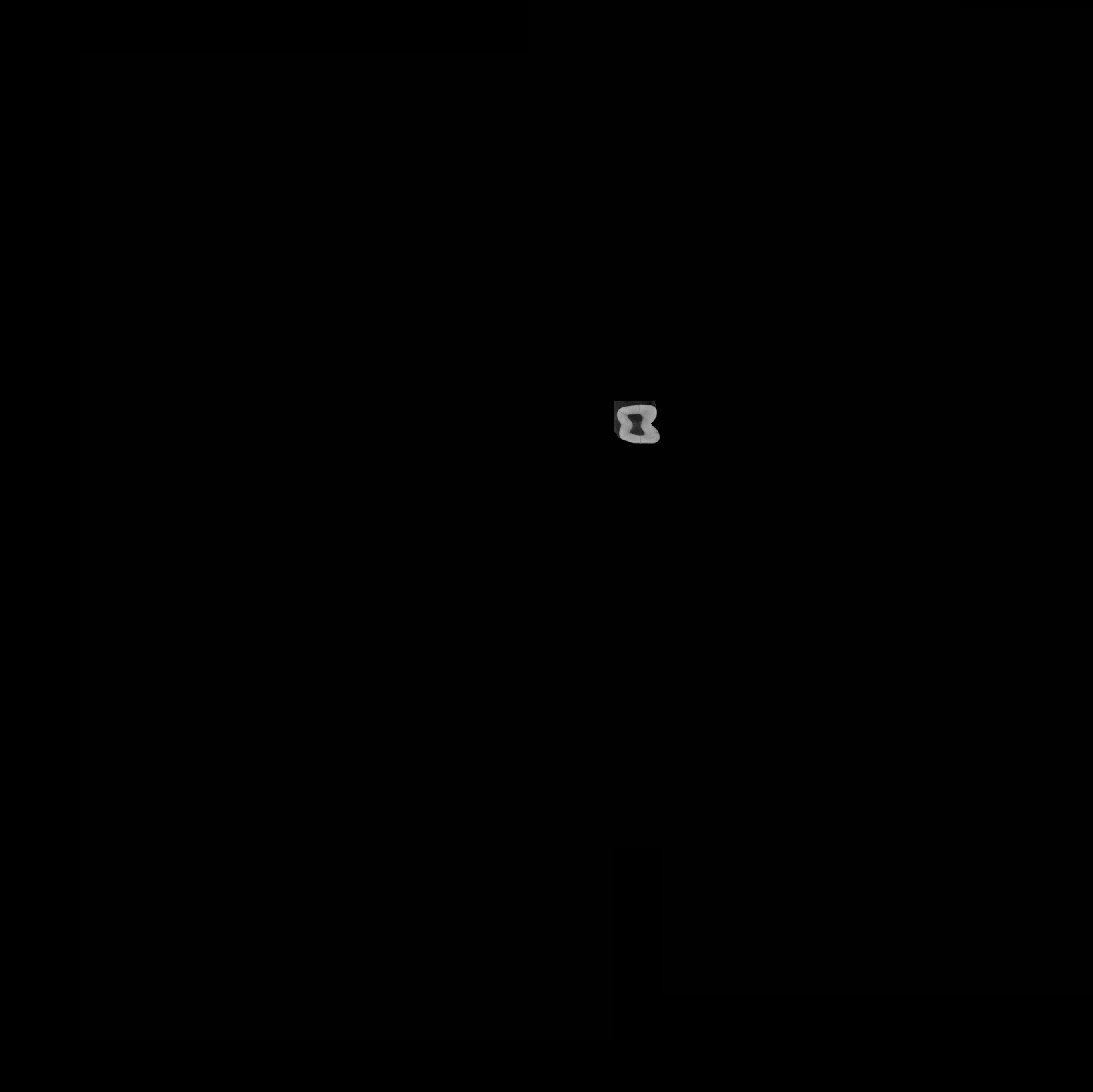

Supplement: Supplementary file 2 — Data S2: Supporting Information. [file AJPA-188-e70164-s001.zip › Cross-Section Tiff Files/amnh_52634_Rm2.tif]

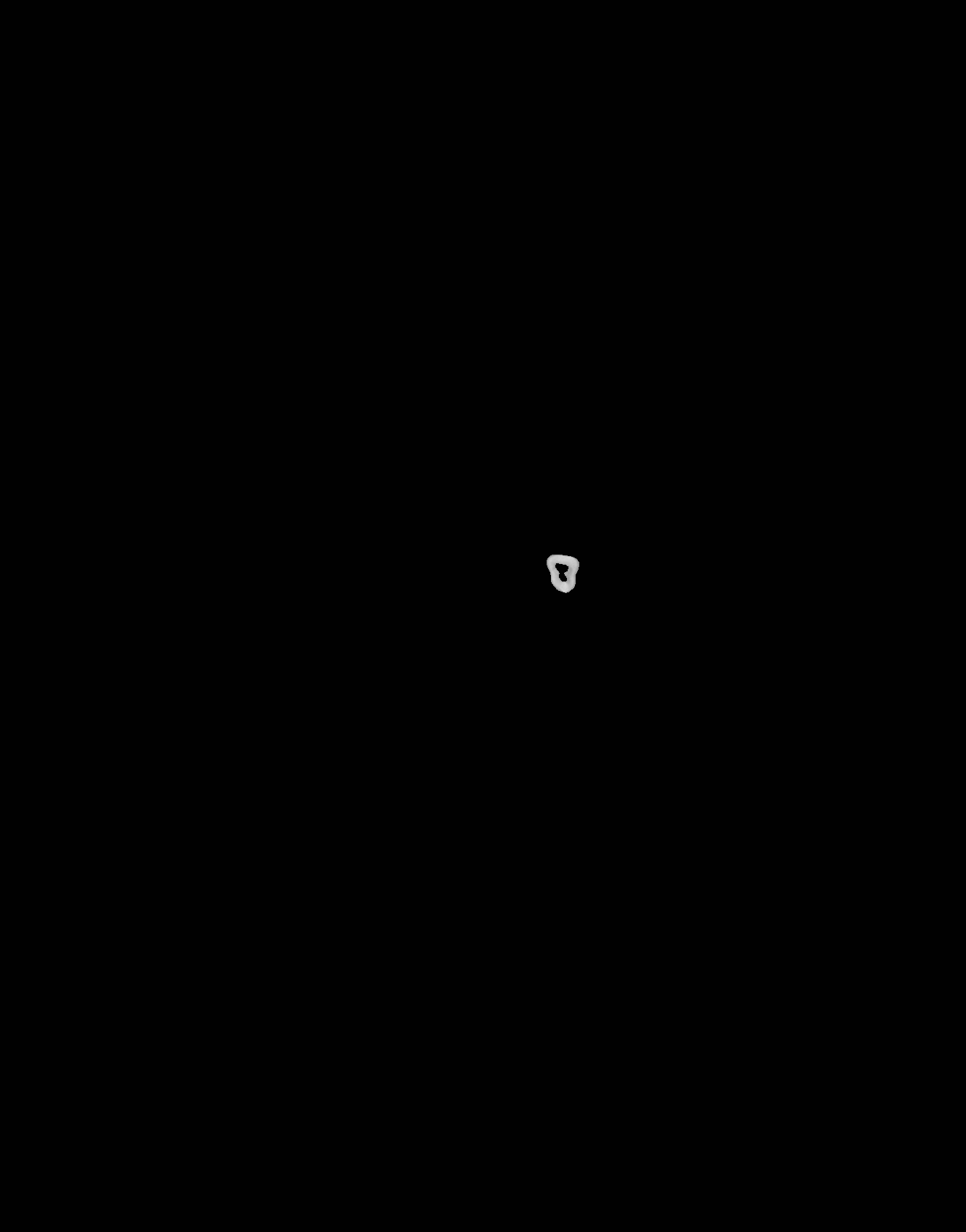

Supplement: Supplementary file 2 — Data S2: Supporting Information. [file AJPA-188-e70164-s001.zip › Cross-Section Tiff Files/mcz_34264_Rm3.tif]

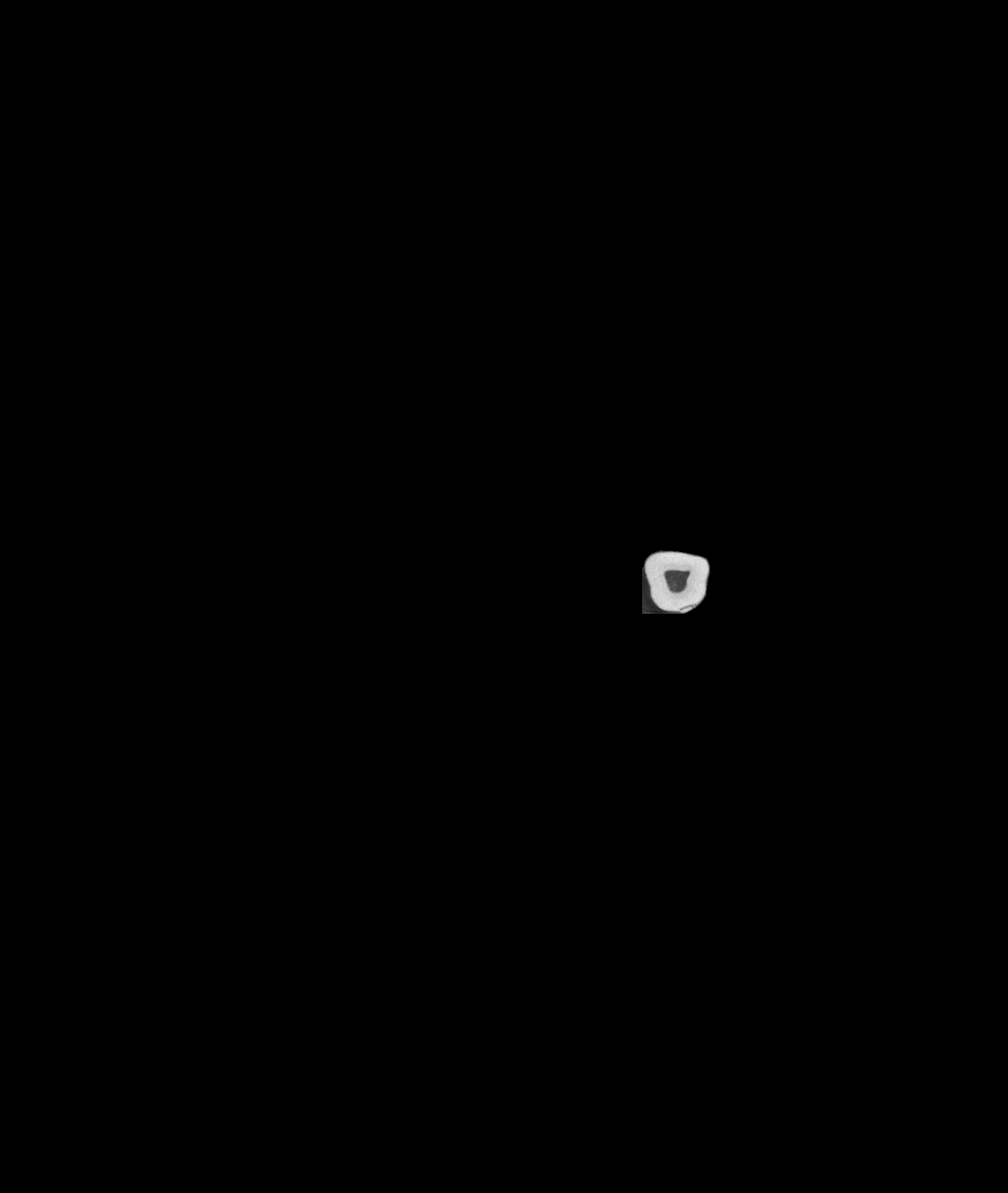

Supplement: Supplementary file 2 — Data S2: Supporting Information. [file AJPA-188-e70164-s001.zip › Cross-Section Tiff Files/mcz_17702_Rm3.tif]

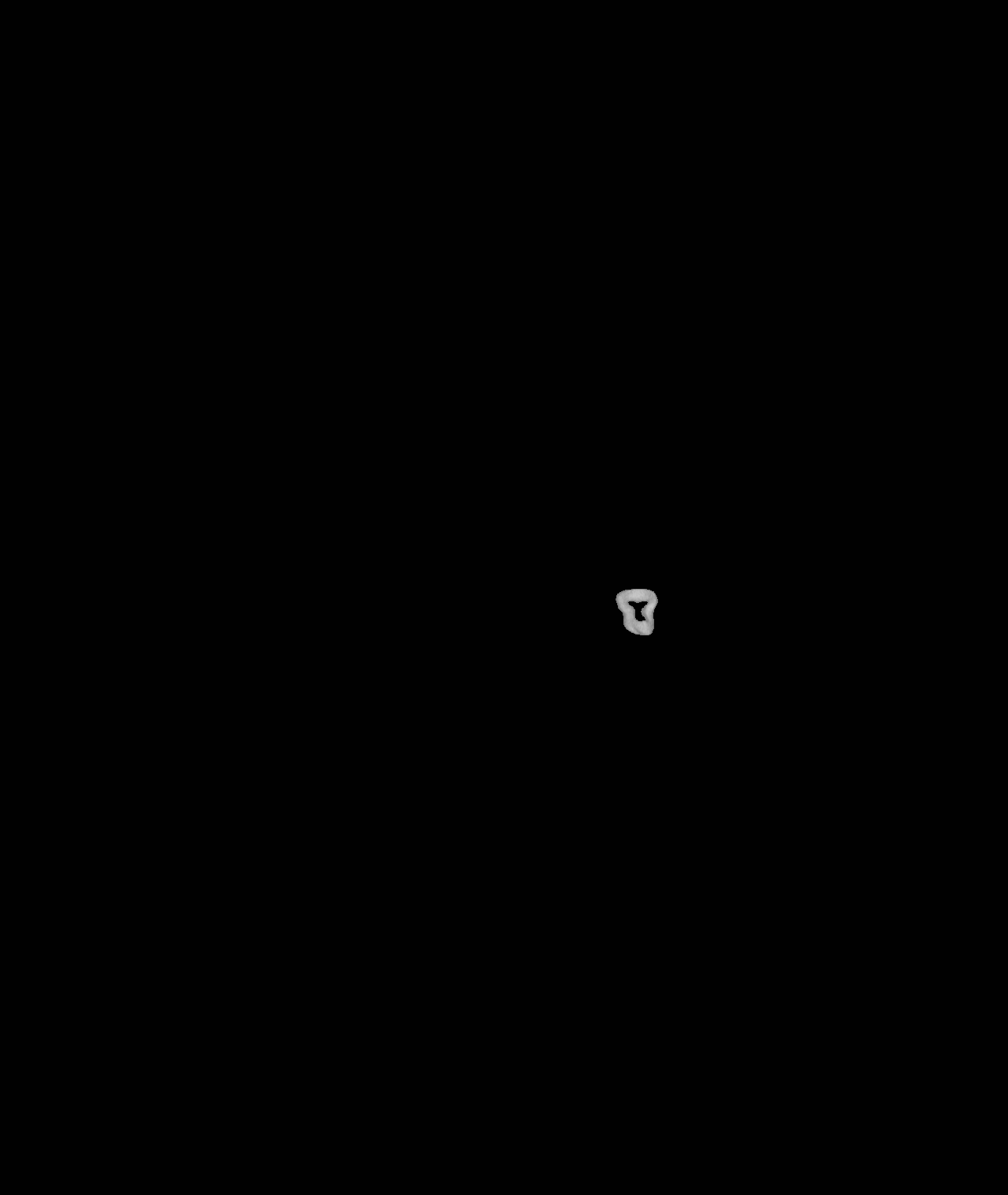

Supplement: Supplementary file 2 — Data S2: Supporting Information. [file AJPA-188-e70164-s001.zip › Cross-Section Tiff Files/mcz_37278_Rm3.tif]

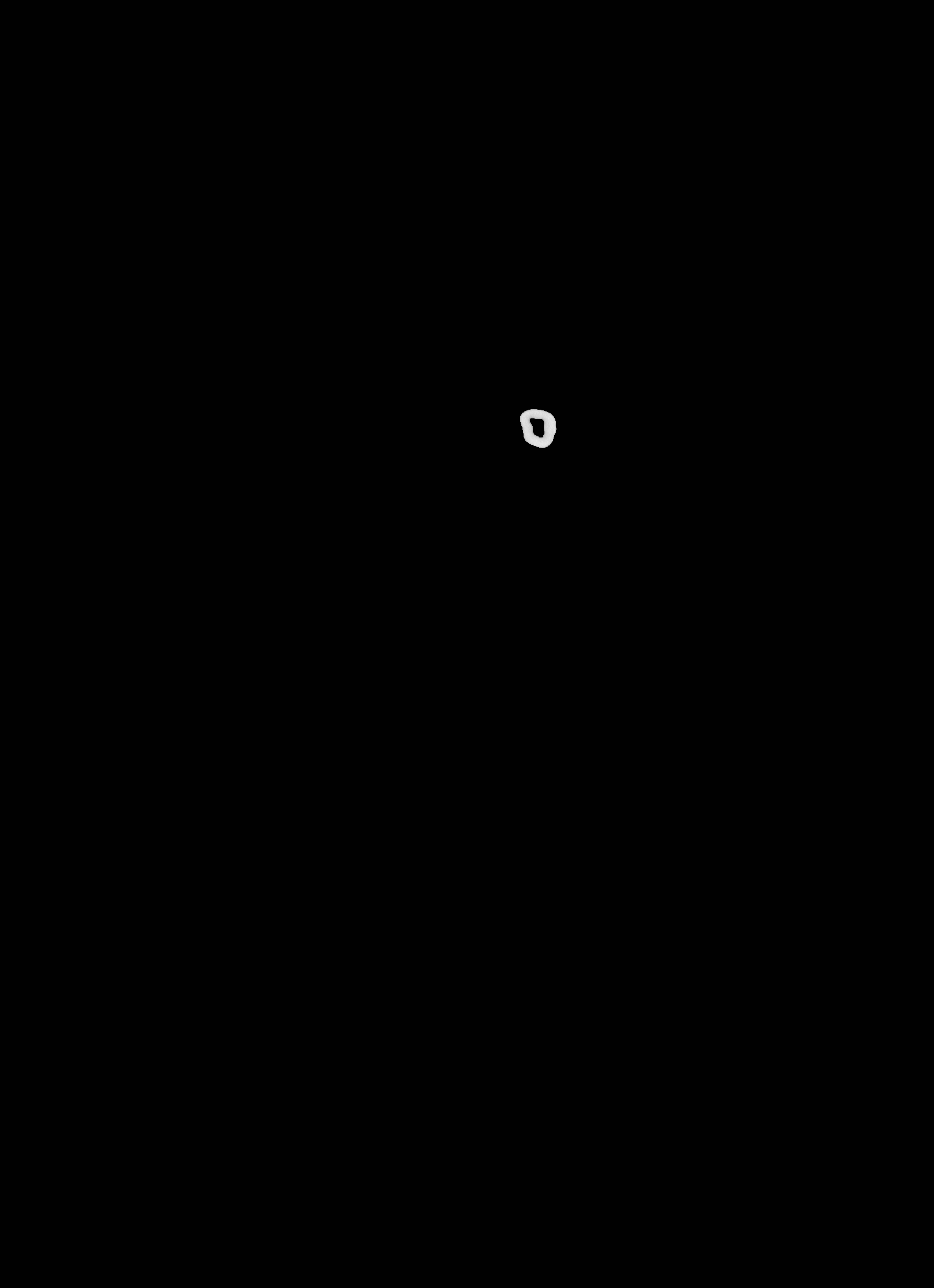

Supplement: Supplementary file 2 — Data S2: Supporting Information. [file AJPA-188-e70164-s001.zip › Cross-Section Tiff Files/mcz_BOM9493_Rm3.tif]

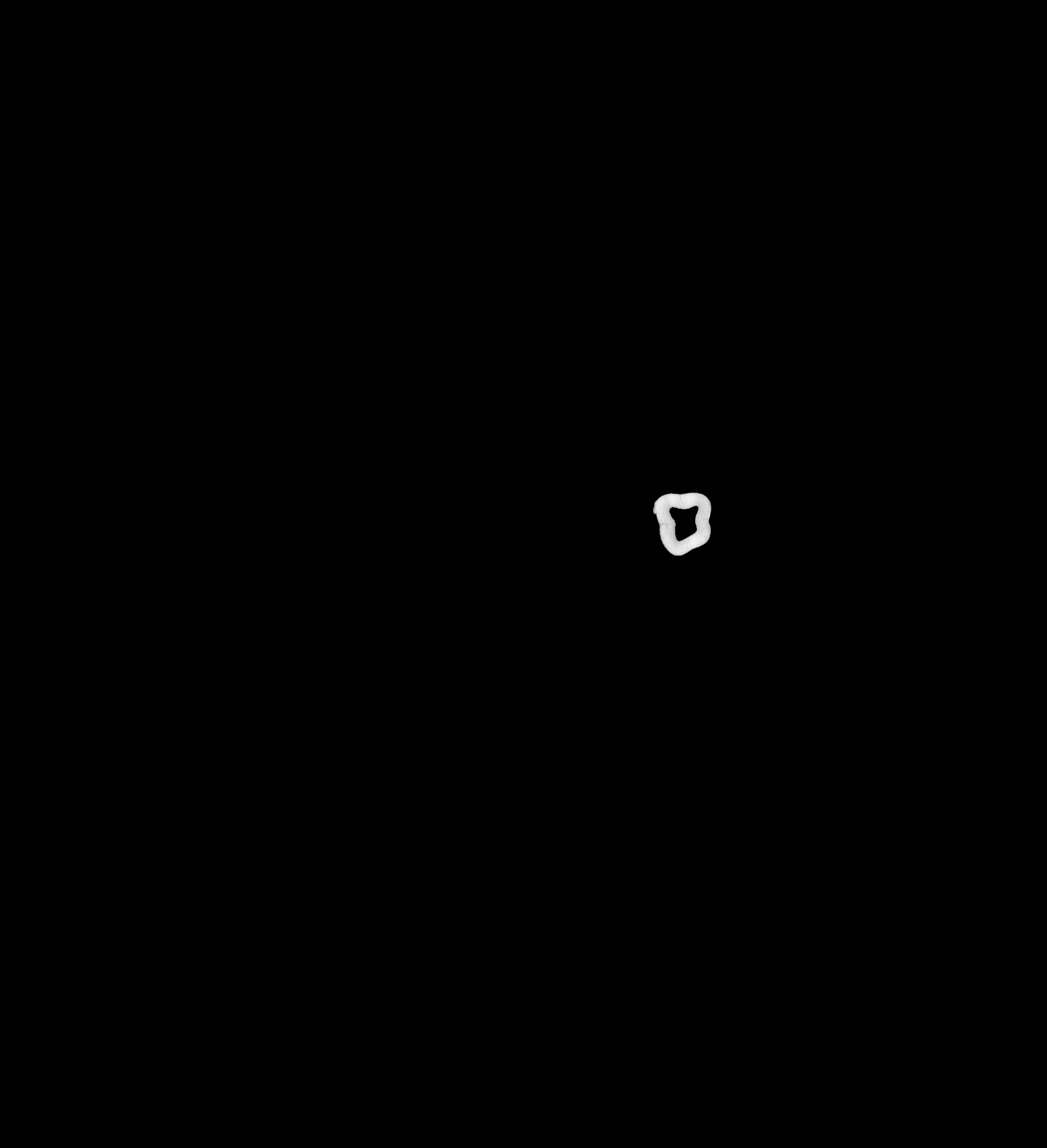

Supplement: Supplementary file 2 — Data S2: Supporting Information. [file AJPA-188-e70164-s001.zip › Cross-Section Tiff Files/mcz_37362_Rm3.tif]

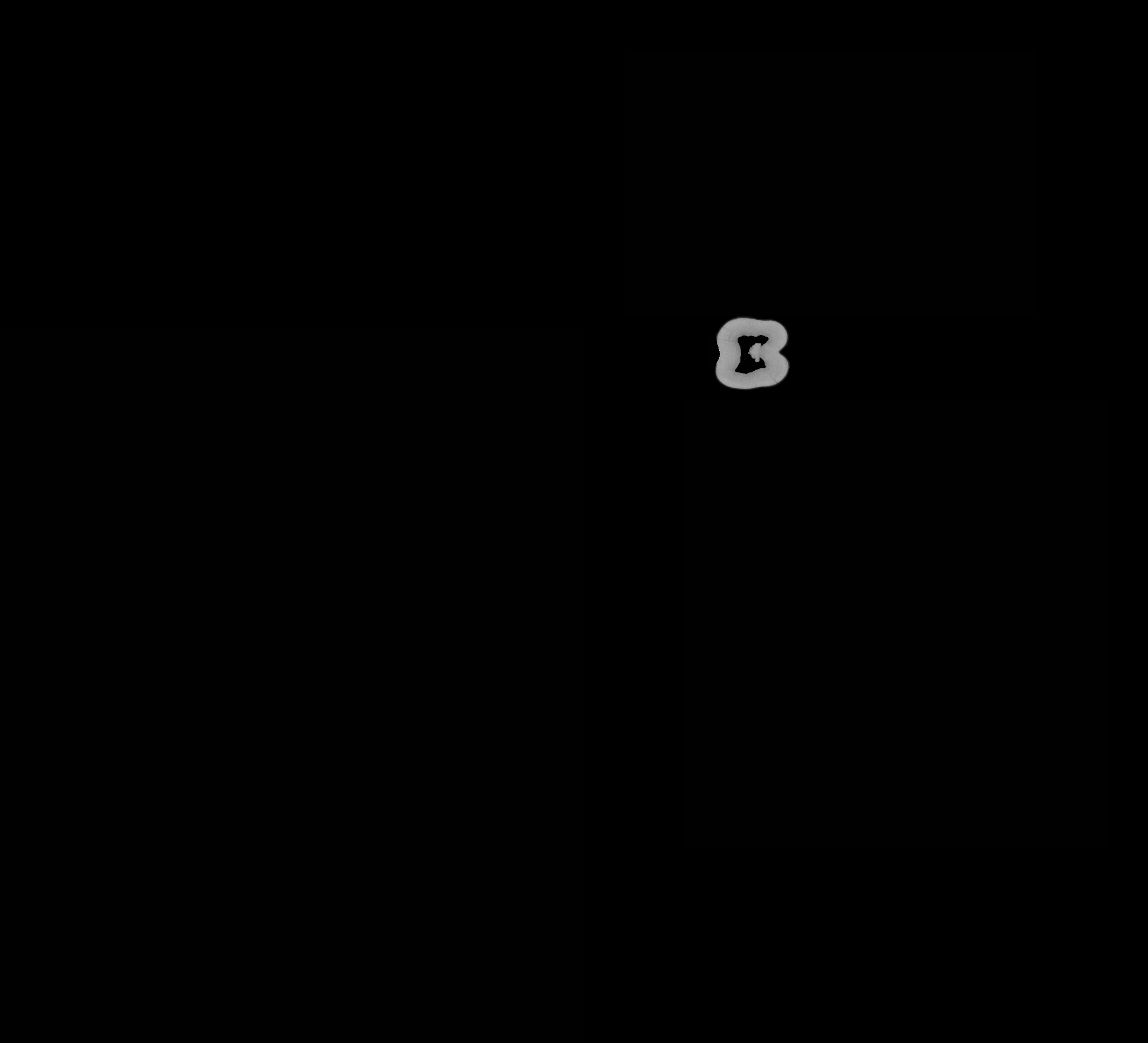

Supplement: Supplementary file 2 — Data S2: Supporting Information. [file AJPA-188-e70164-s001.zip › Cross-Section Tiff Files/amnh_AL223_Rm1.tif]

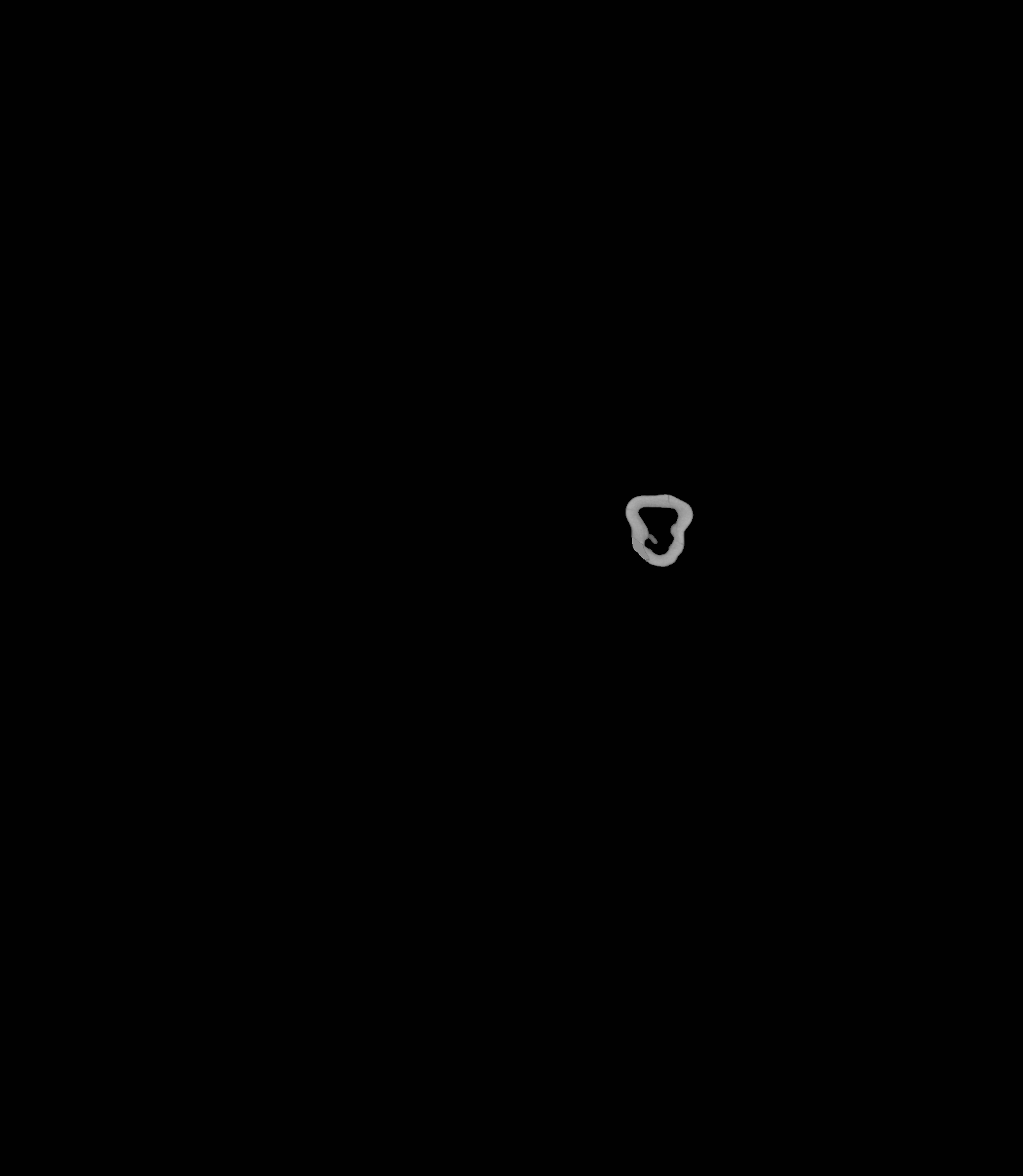

Supplement: Supplementary file 2 — Data S2: Supporting Information. [file AJPA-188-e70164-s001.zip › Cross-Section Tiff Files/amnh_167338_Rm3.tif]

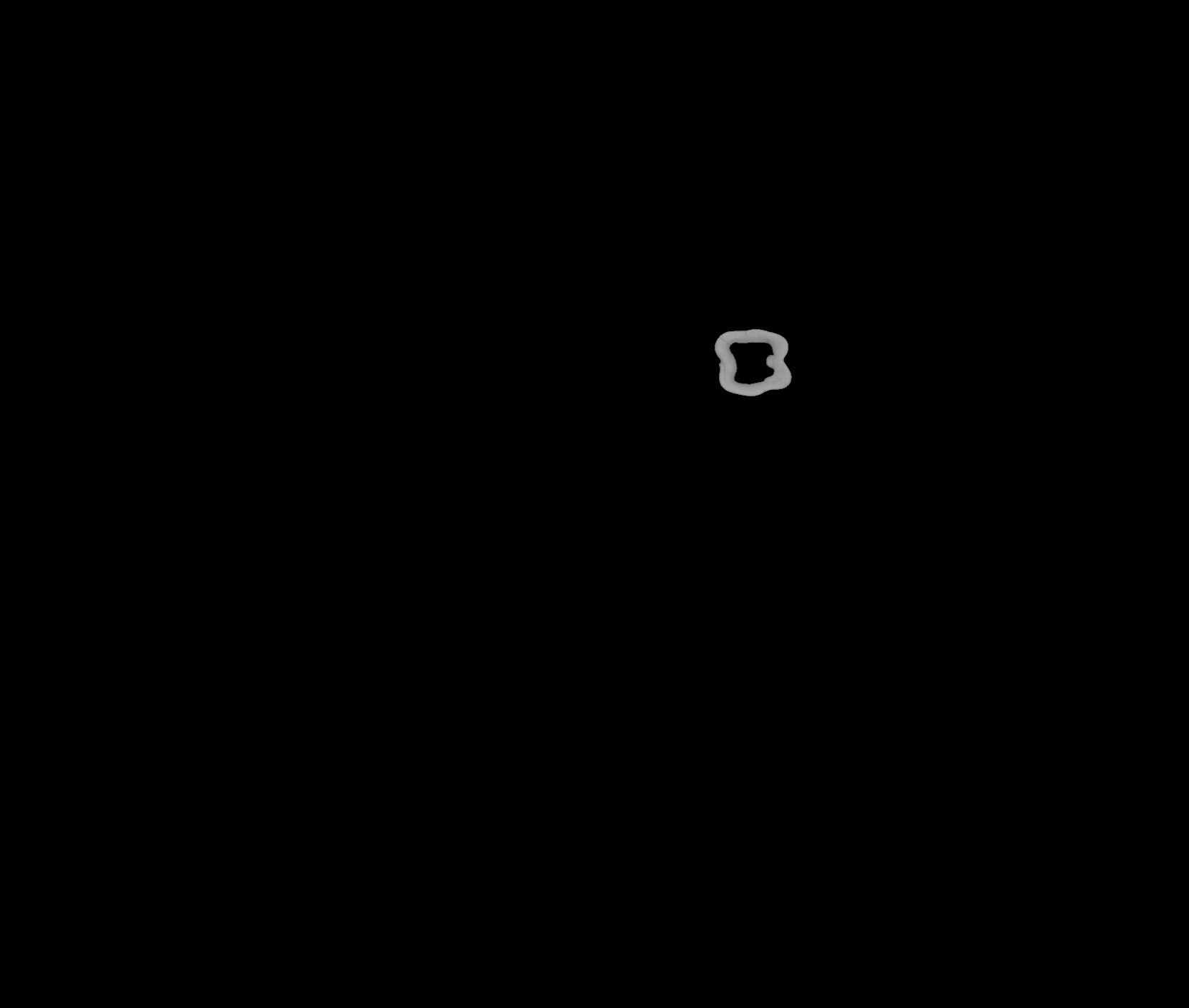

Supplement: Supplementary file 2 — Data S2: Supporting Information. [file AJPA-188-e70164-s001.zip › Cross-Section Tiff Files/amnh_167338_Rm2.tif]

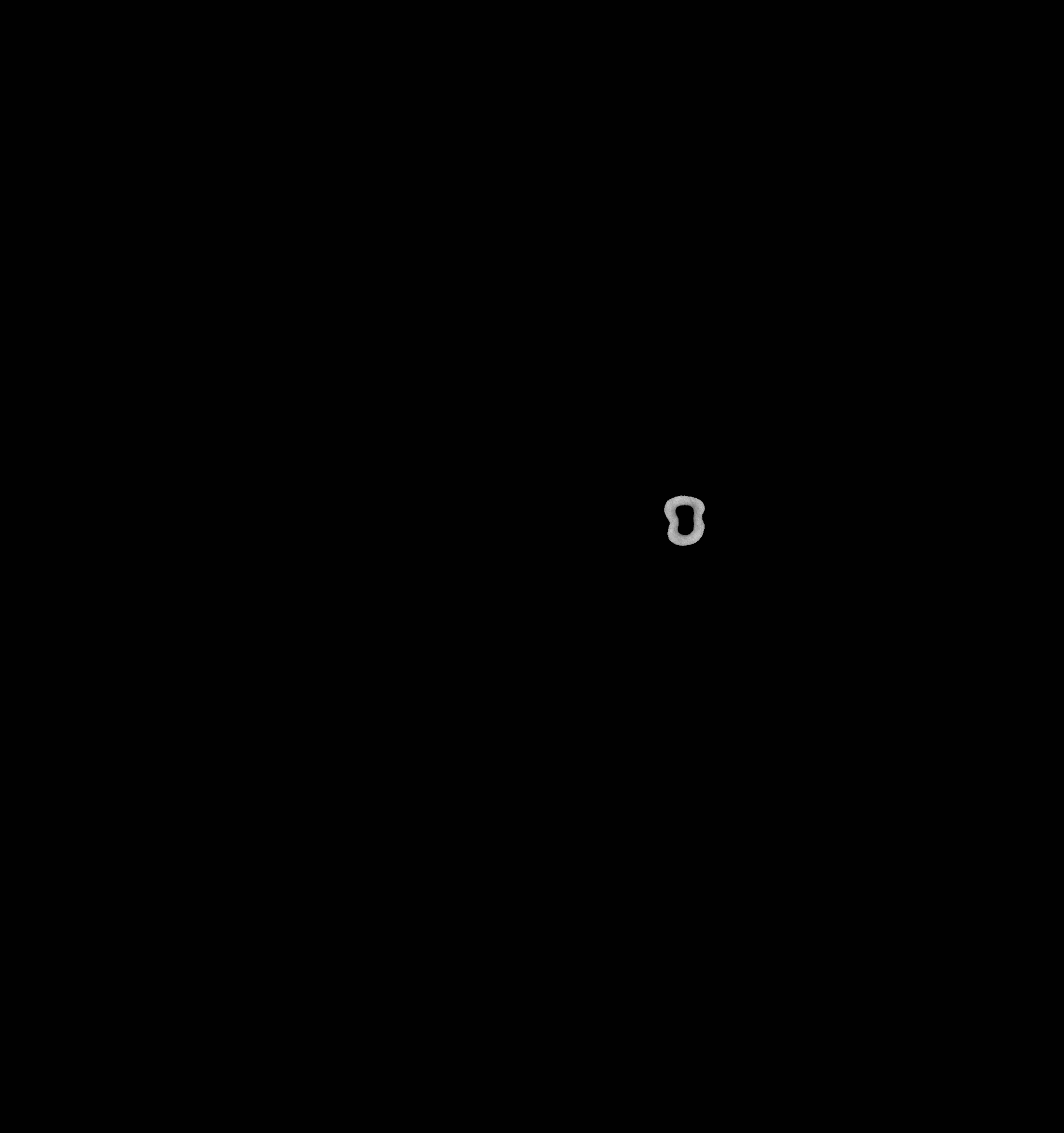

Supplement: Supplementary file 2 — Data S2: Supporting Information. [file AJPA-188-e70164-s001.zip › Cross-Section Tiff Files/mcz_23164_Rm3.tif]

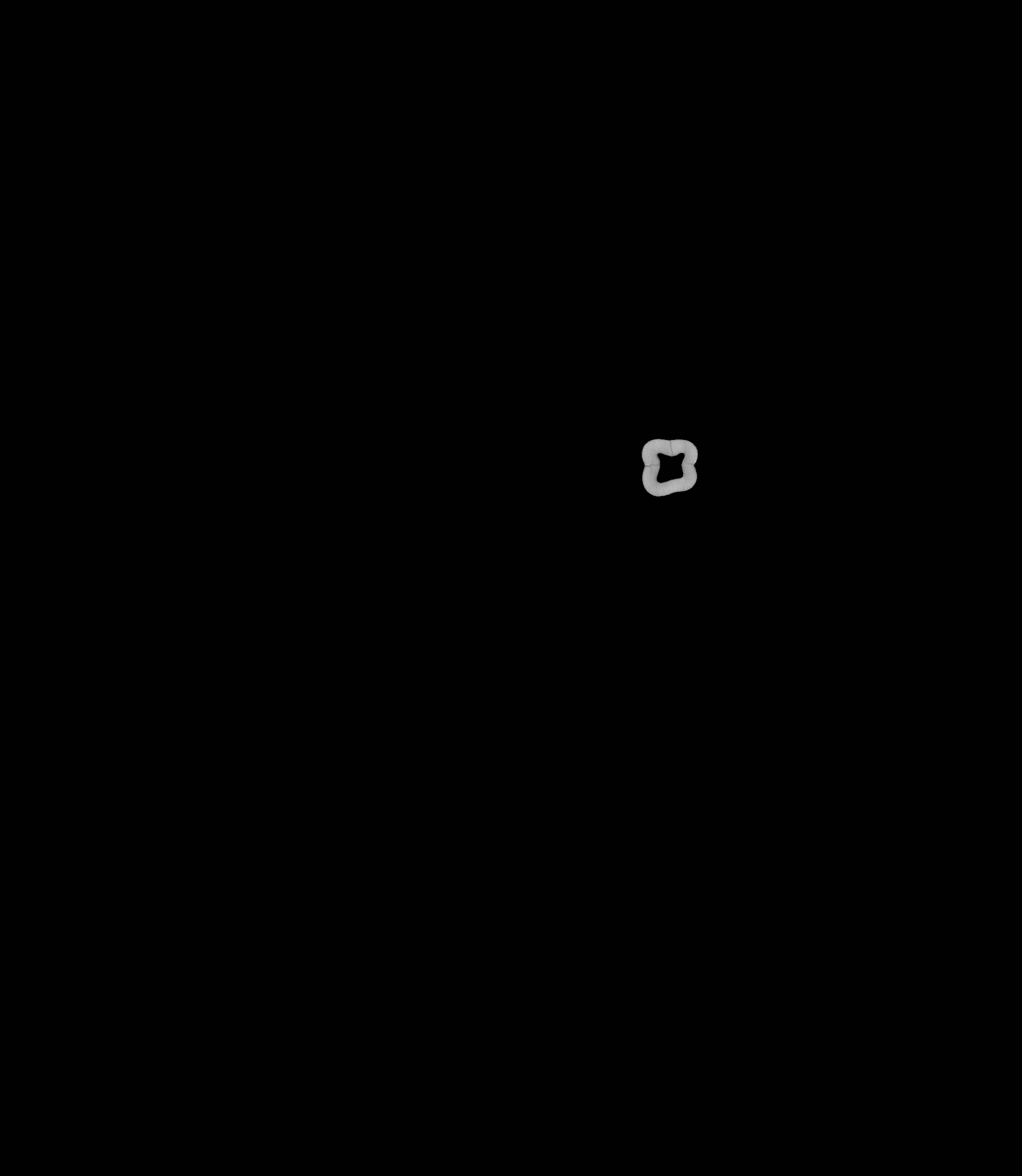

Supplement: Supplementary file 2 — Data S2: Supporting Information. [file AJPA-188-e70164-s001.zip › Cross-Section Tiff Files/mcz_37362_Rm2.tif]

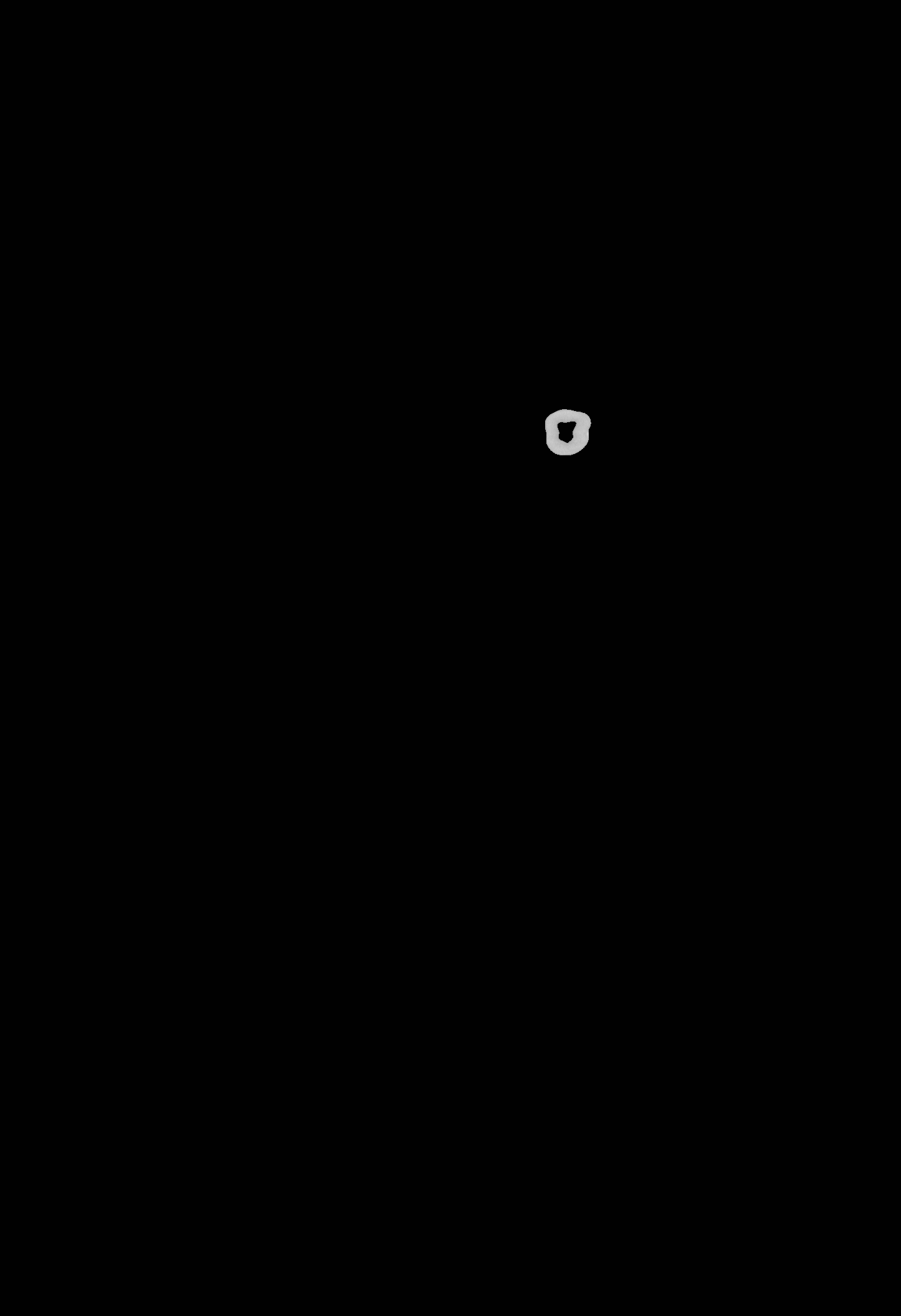

Supplement: Supplementary file 2 — Data S2: Supporting Information. [file AJPA-188-e70164-s001.zip › Cross-Section Tiff Files/mcz_BOM9493_Rm2.tif]

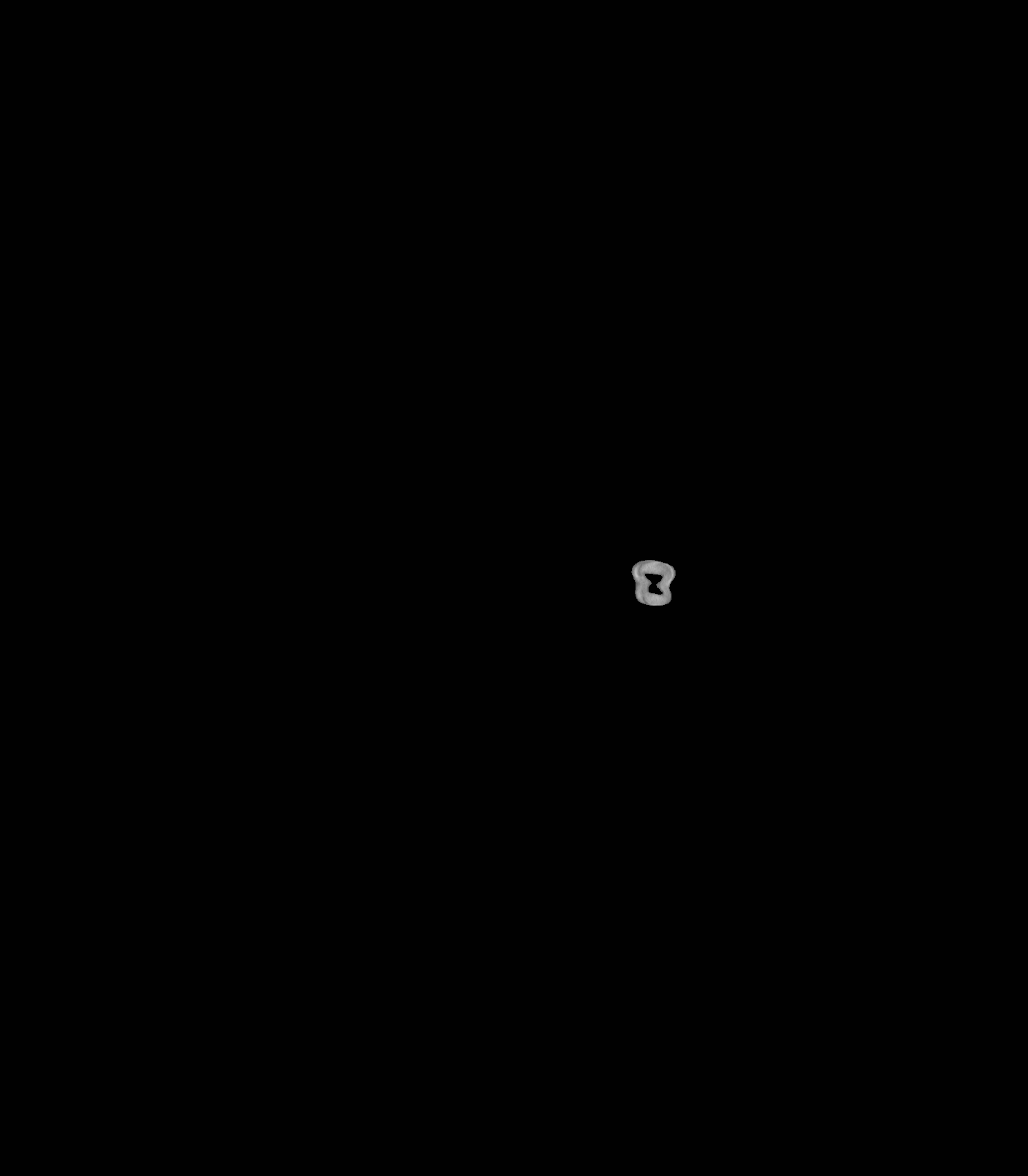

Supplement: Supplementary file 2 — Data S2: Supporting Information. [file AJPA-188-e70164-s001.zip › Cross-Section Tiff Files/mcz_37278_Rm2.tif]

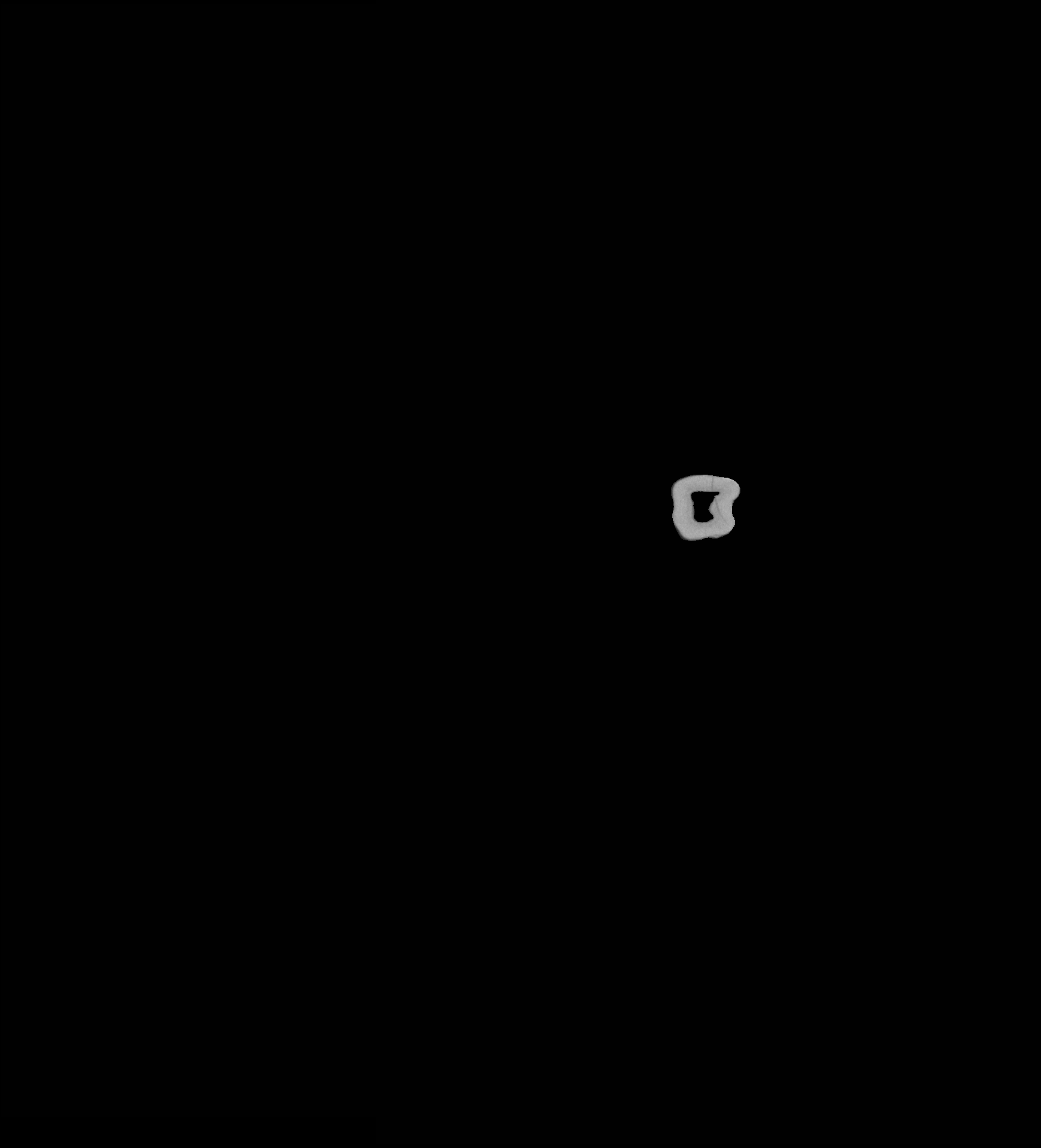

Supplement: Supplementary file 2 — Data S2: Supporting Information. [file AJPA-188-e70164-s001.zip › Cross-Section Tiff Files/mcz_17702_Rm2.tif]

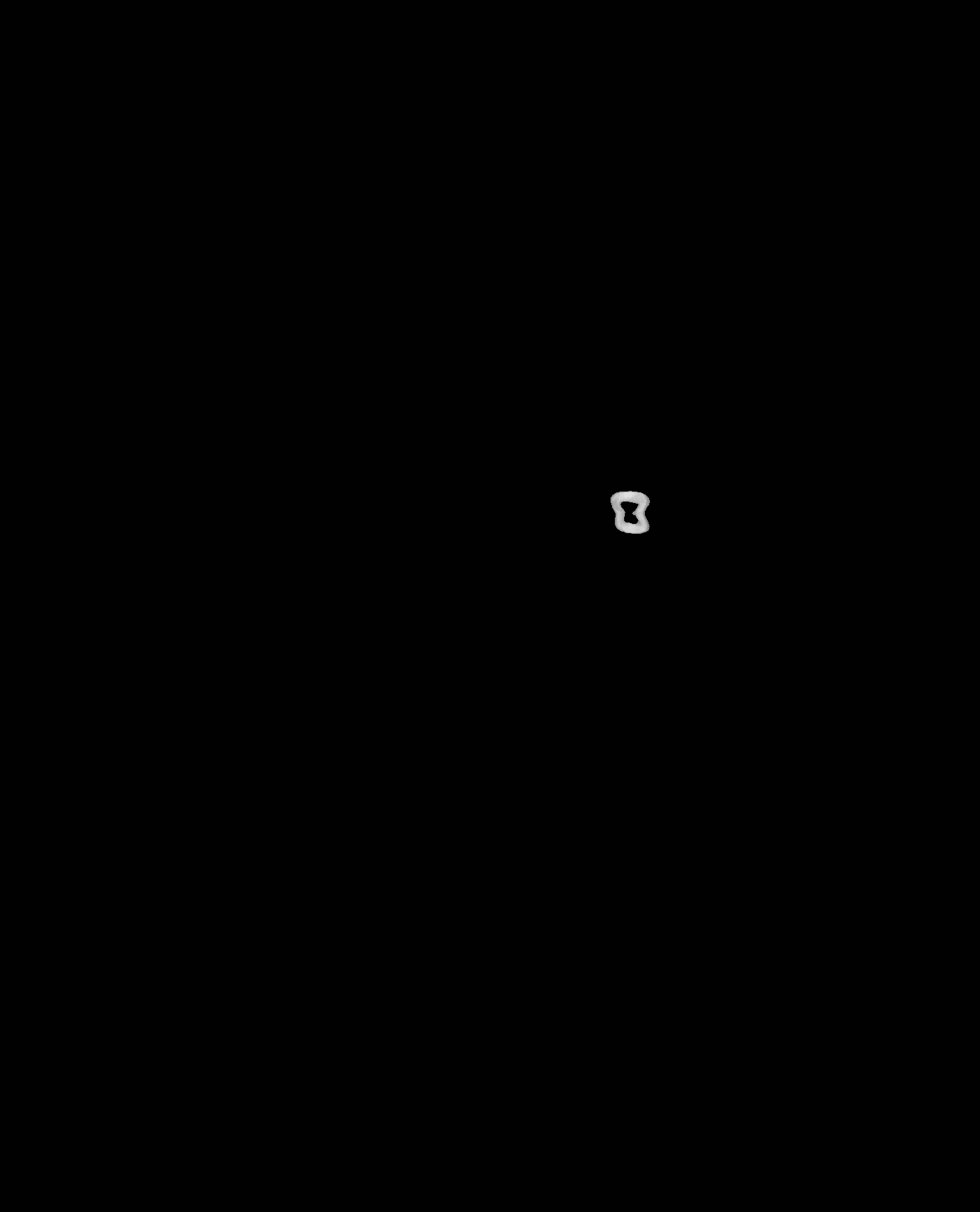

Supplement: Supplementary file 2 — Data S2: Supporting Information. [file AJPA-188-e70164-s001.zip › Cross-Section Tiff Files/mcz_34264_Rm2.tif]

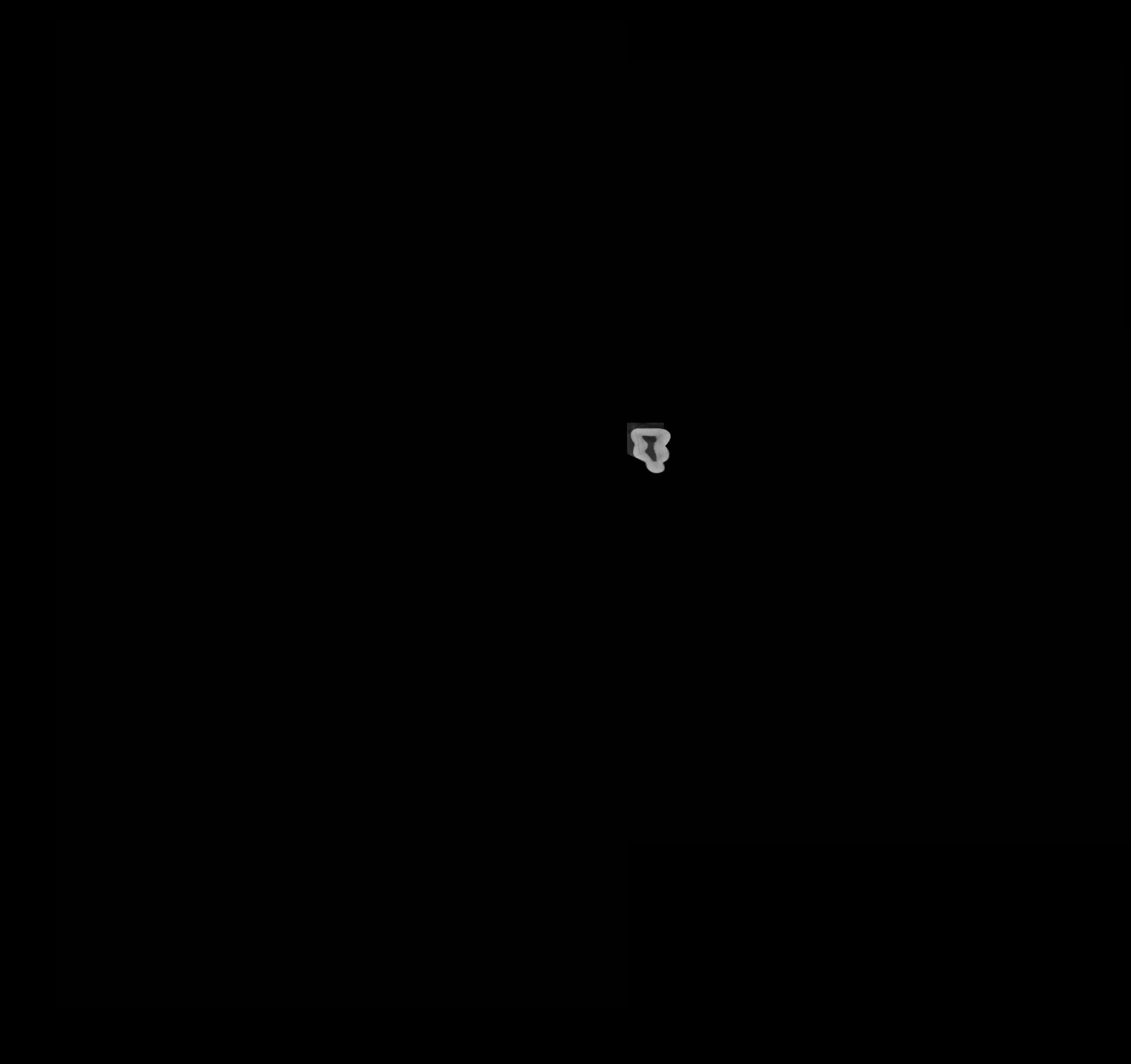

Supplement: Supplementary file 2 — Data S2: Supporting Information. [file AJPA-188-e70164-s001.zip › Cross-Section Tiff Files/amnh_52634_Rm3.tif]

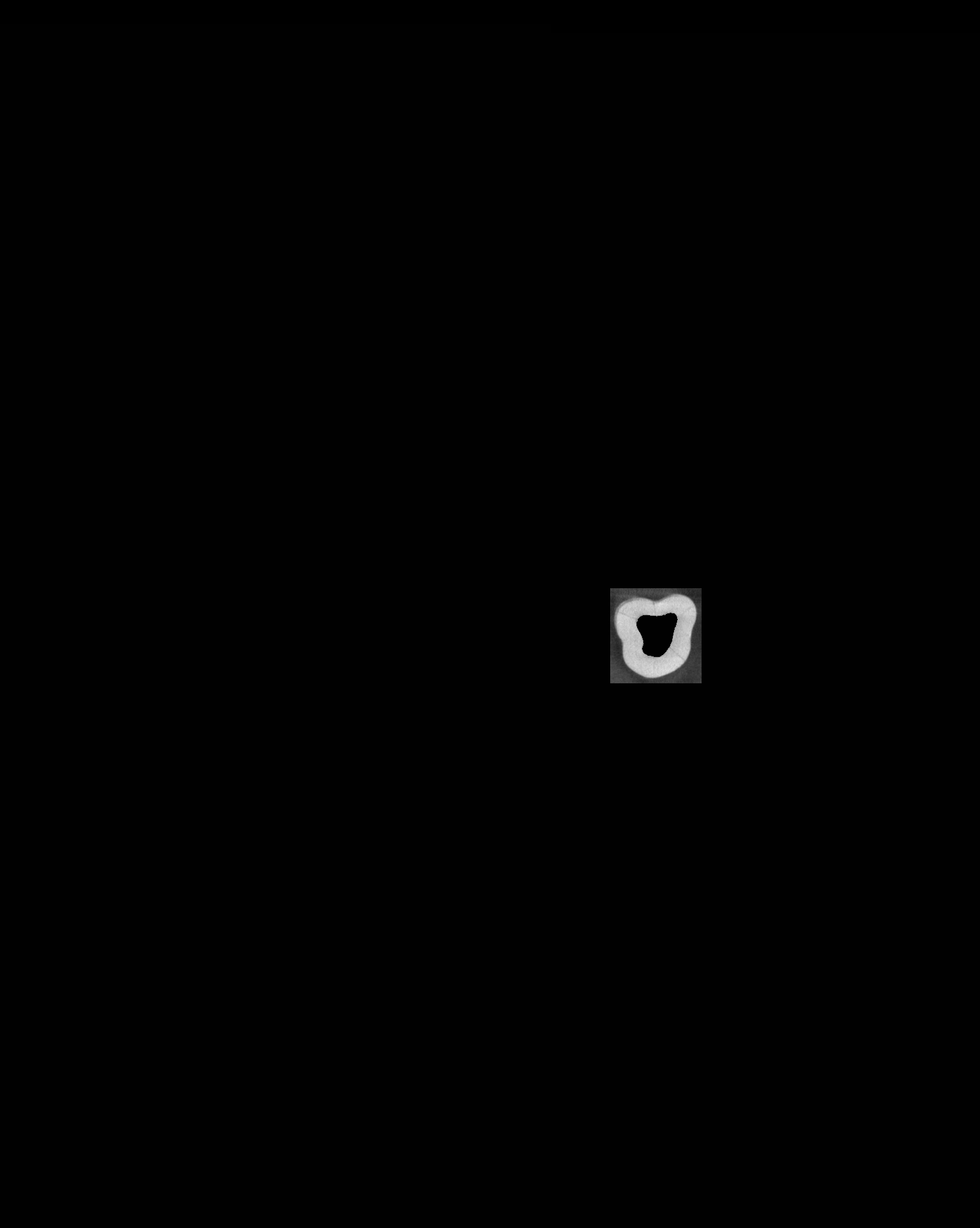

Supplement: Supplementary file 2 — Data S2: Supporting Information. [file AJPA-188-e70164-s001.zip › Cross-Section Tiff Files/mcz_37518_Rm3.tif]
