# Supplementary figures and images for: Molar Cervical Root Cross‐Sectional Morphology and Diet in Extant Catarrhines (part 1 of 2)
Source: Am J Biol Anthropol. 2025 Nov 18;188(3):e70164. doi: 10.1002/ajpa.70164 (PMC12625805; doi:10.1002/ajpa.70164)

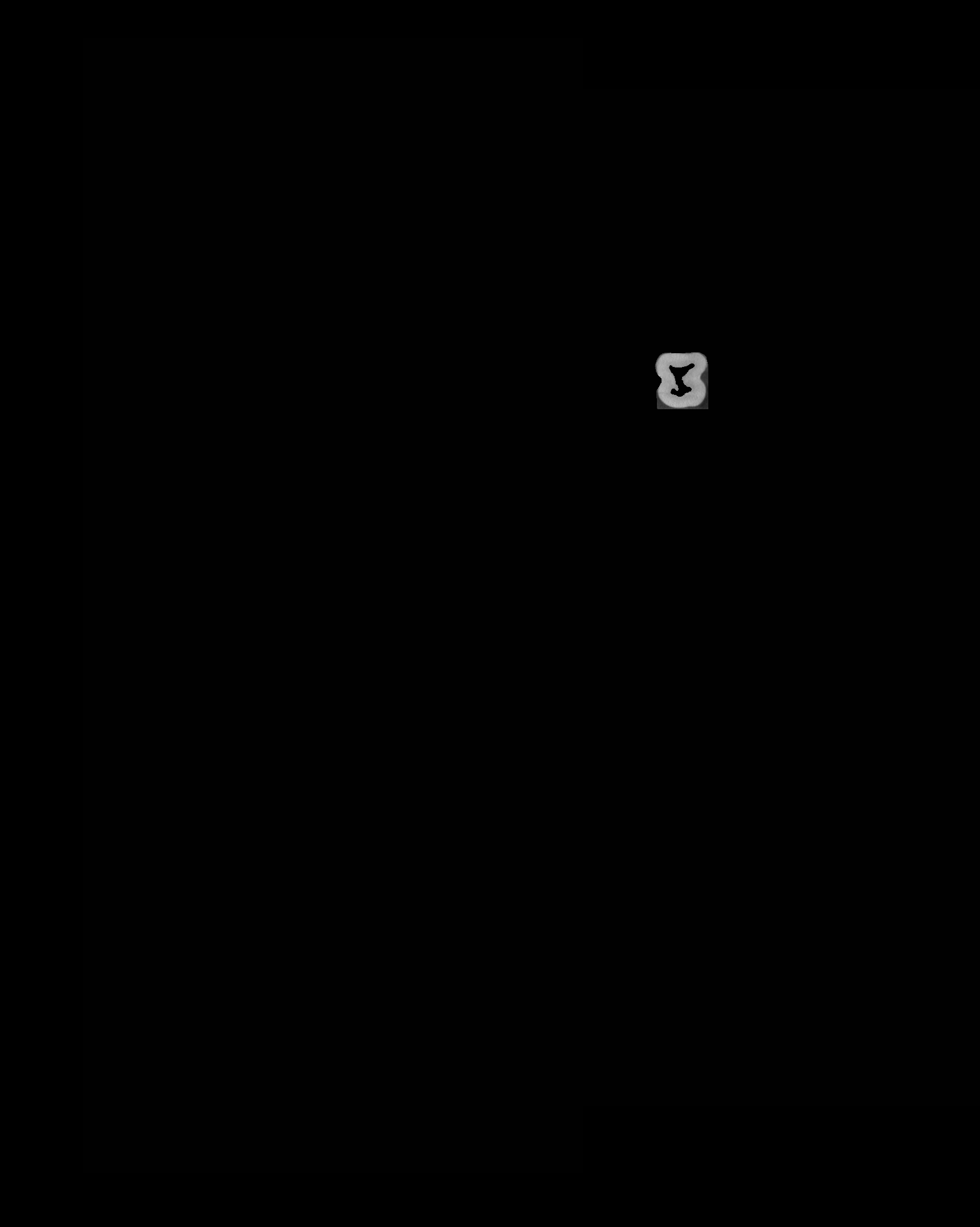

Supplement: Supplementary file 2 — Data S2: Supporting Information. [file AJPA-188-e70164-s001.zip › Cross-Section Tiff Files/mcz_37264_Rm1.tif]

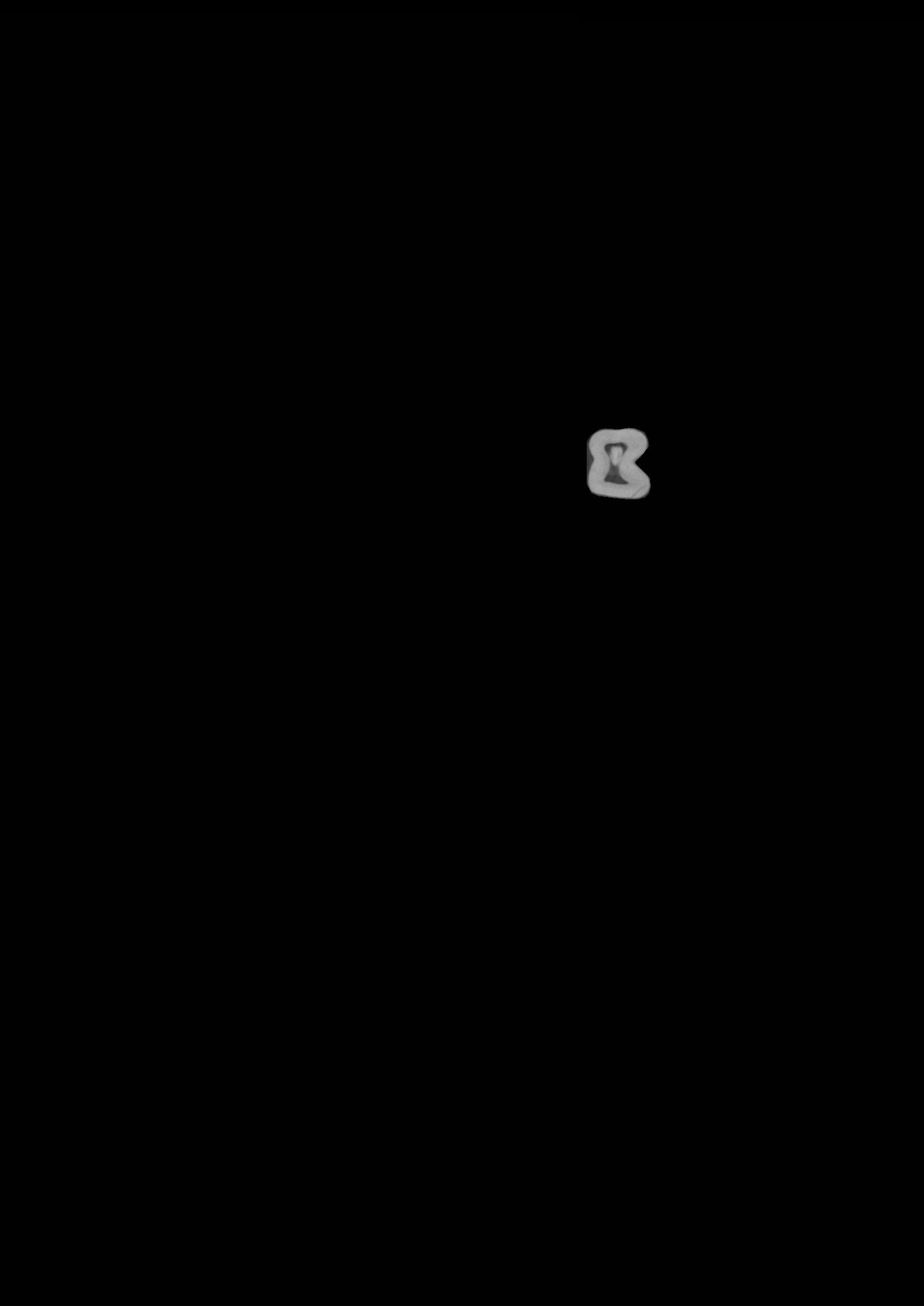

Supplement: Supplementary file 2 — Data S2: Supporting Information. [file AJPA-188-e70164-s001.zip › Cross-Section Tiff Files/mcz_29786_Rm1.tif]

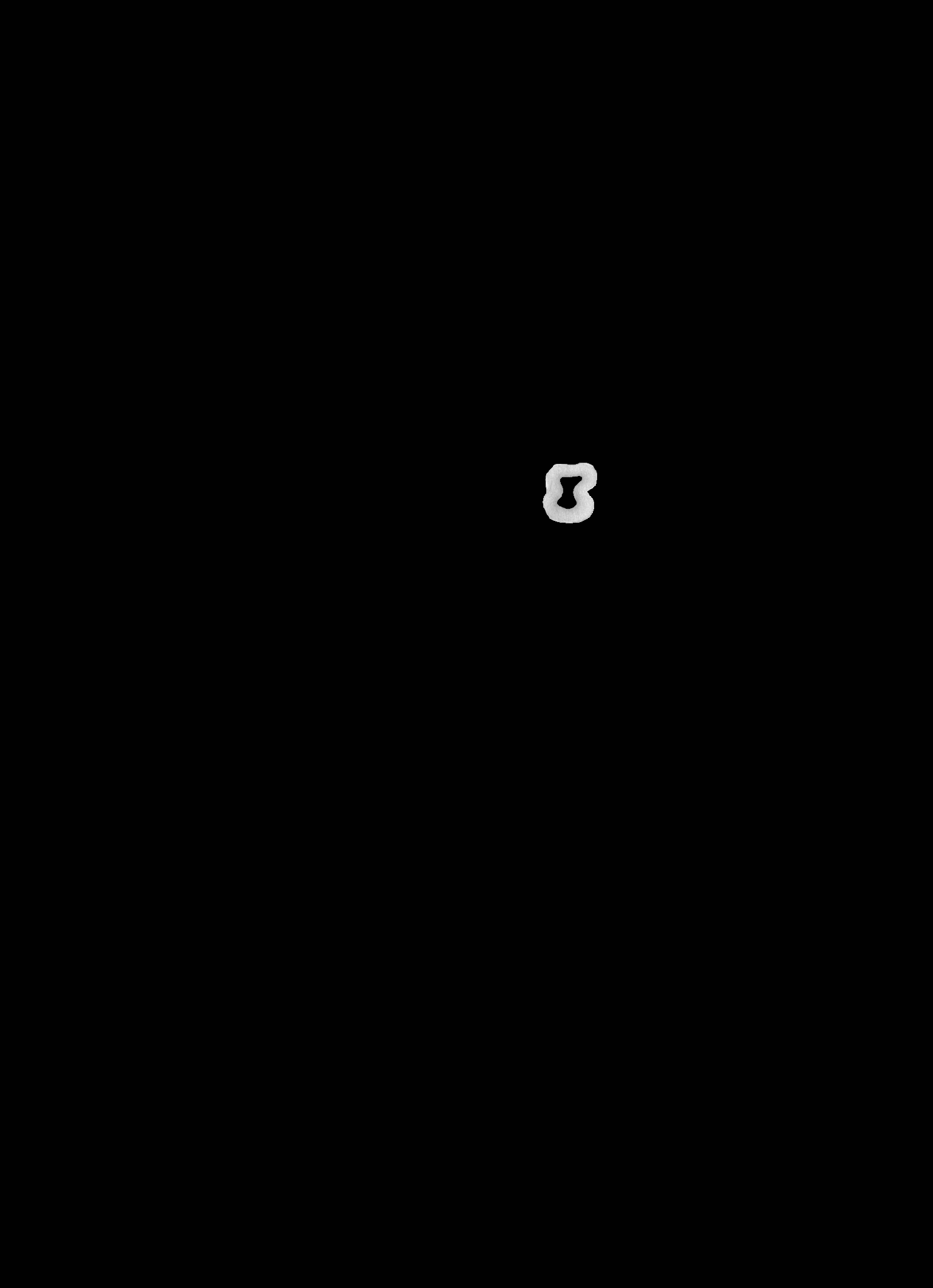

Supplement: Supplementary file 2 — Data S2: Supporting Information. [file AJPA-188-e70164-s001.zip › Cross-Section Tiff Files/mcz_20041_Rm1.tif]

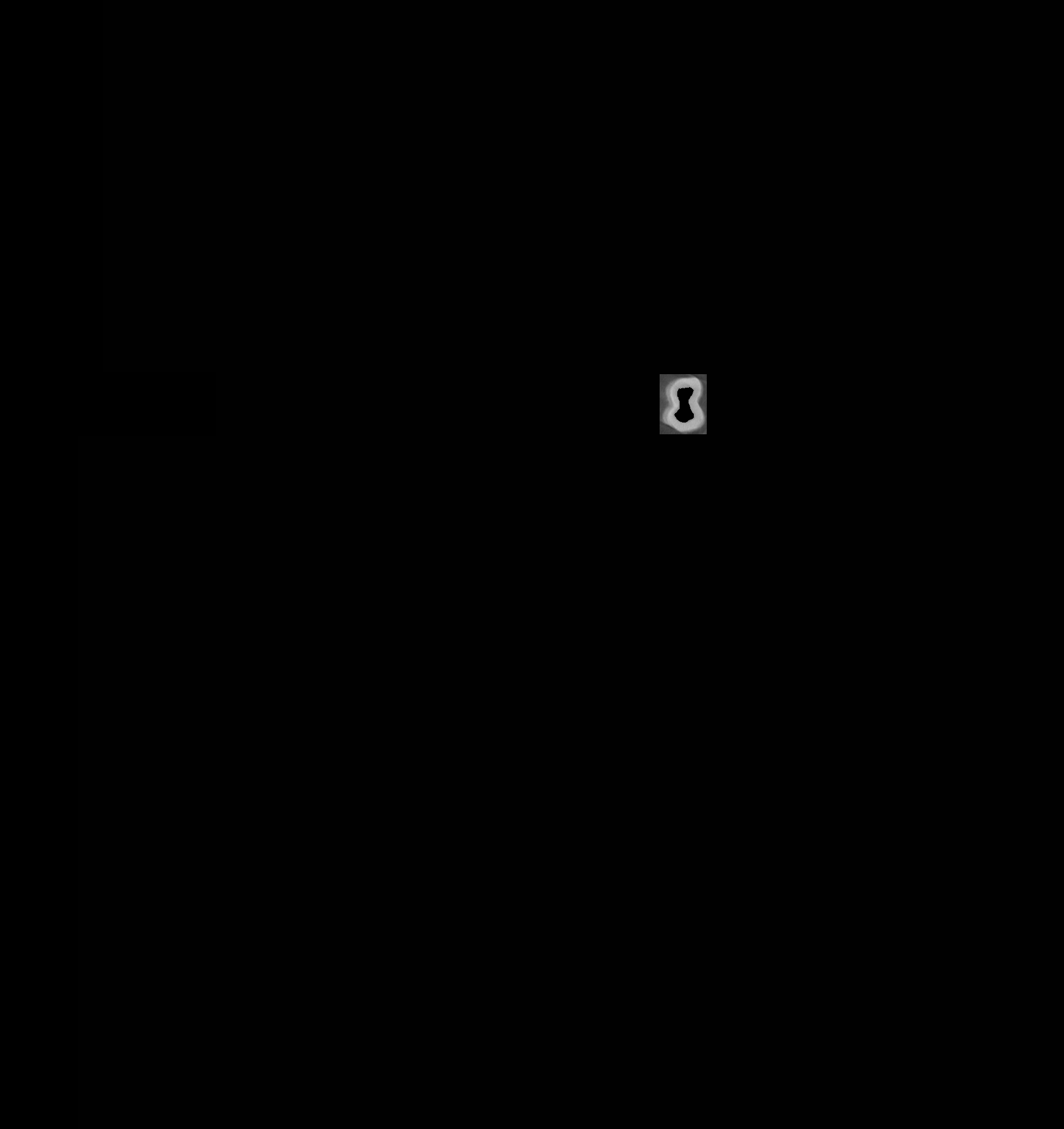

Supplement: Supplementary file 2 — Data S2: Supporting Information. [file AJPA-188-e70164-s001.zip › Cross-Section Tiff Files/mcz_36031_Rm1.tif]

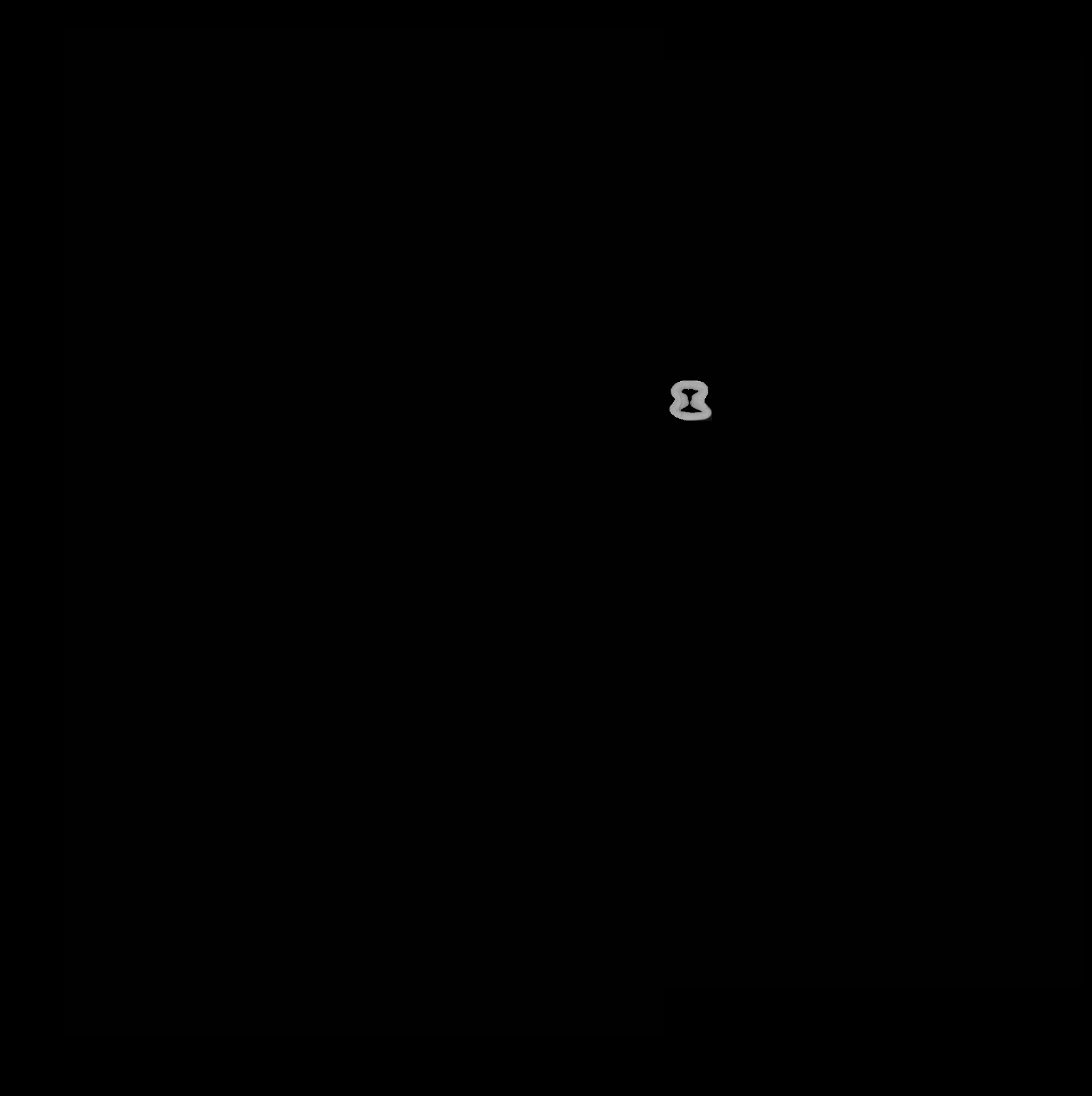

Supplement: Supplementary file 2 — Data S2: Supporting Information. [file AJPA-188-e70164-s001.zip › Cross-Section Tiff Files/amnh_52237_Rm2.tif]

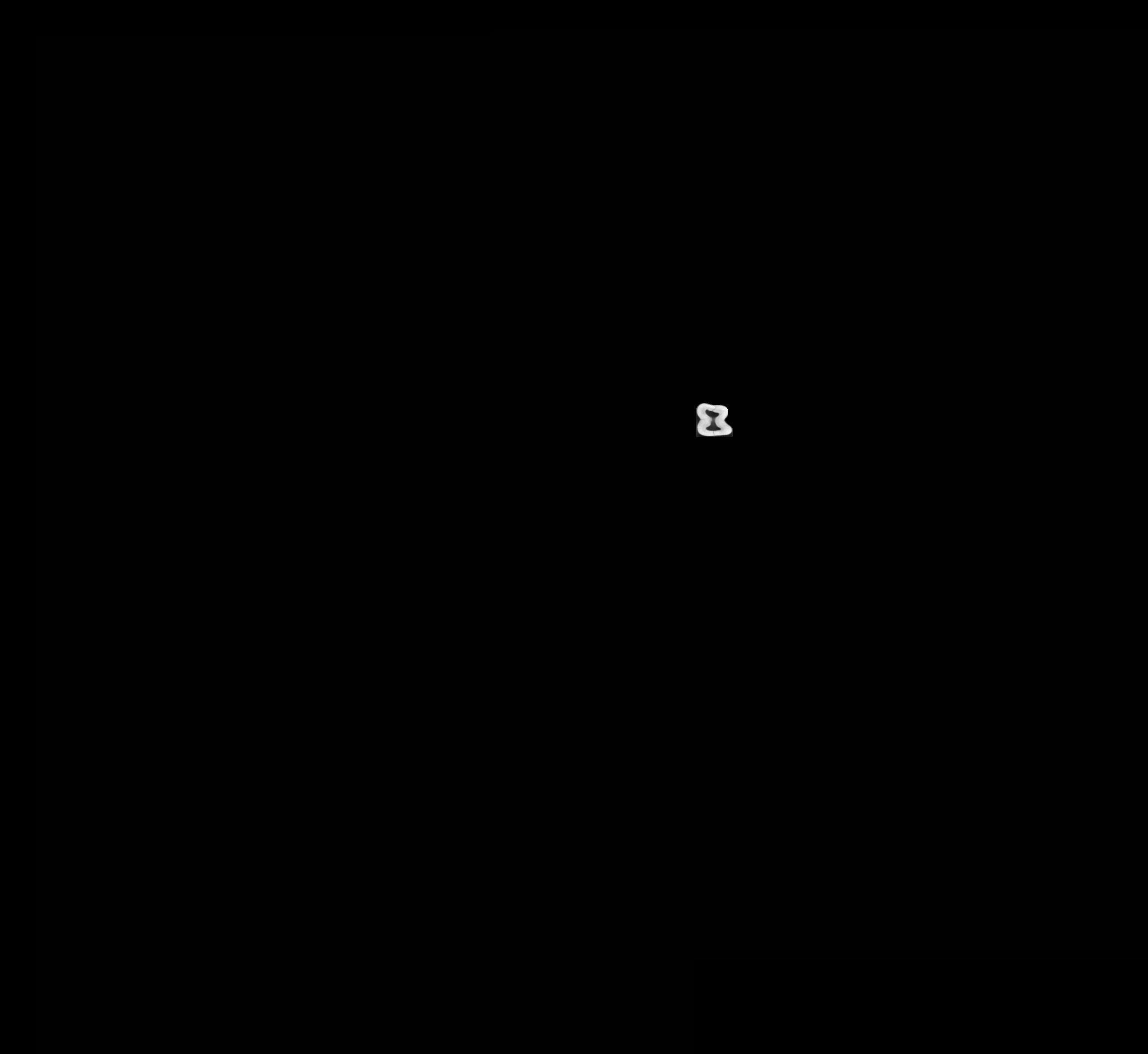

Supplement: Supplementary file 2 — Data S2: Supporting Information. [file AJPA-188-e70164-s001.zip › Cross-Section Tiff Files/amnh_90309_Rm1.tif]

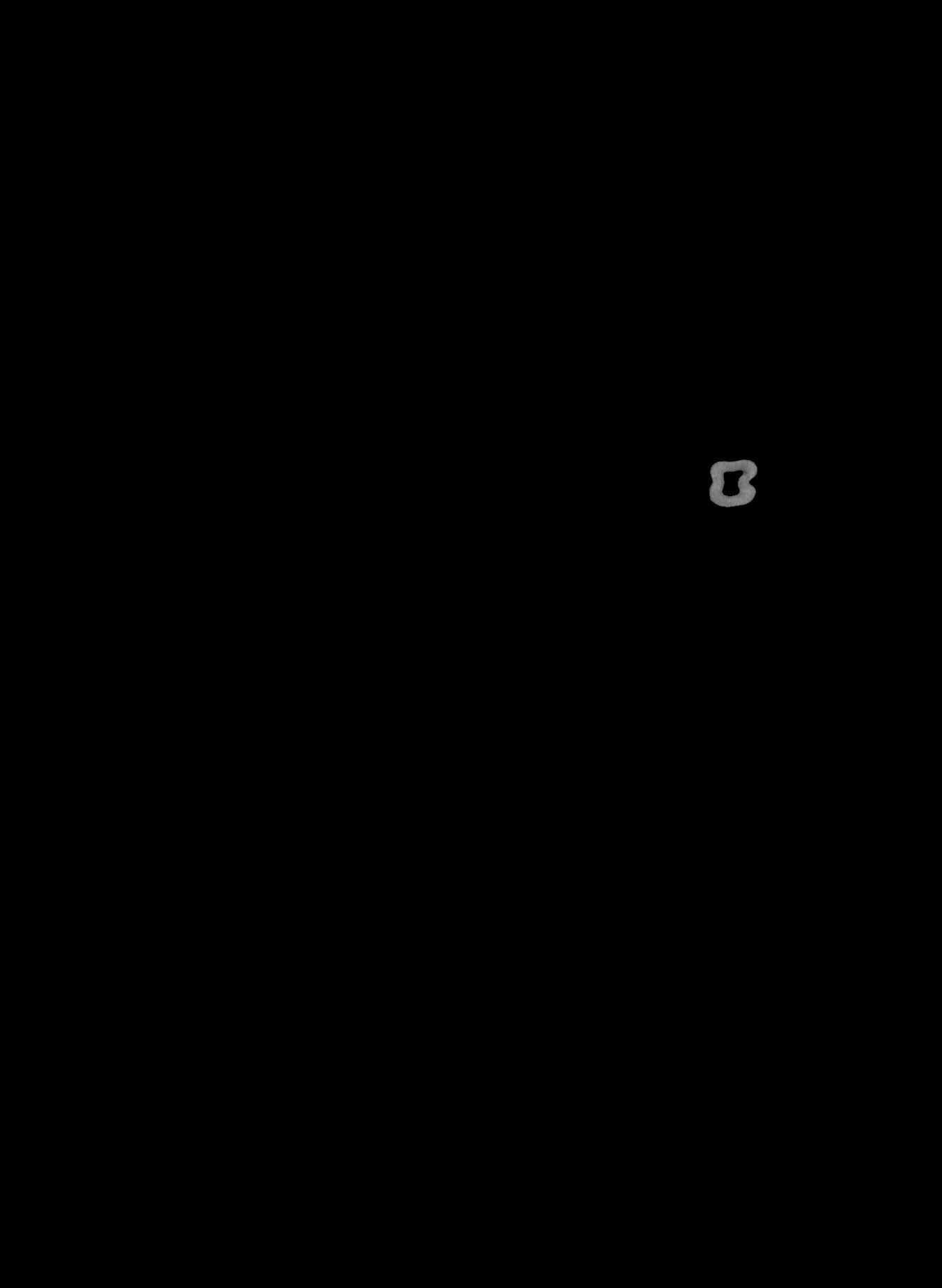

Supplement: Supplementary file 2 — Data S2: Supporting Information. [file AJPA-188-e70164-s001.zip › Cross-Section Tiff Files/mcz_15312_Rm1.tif]

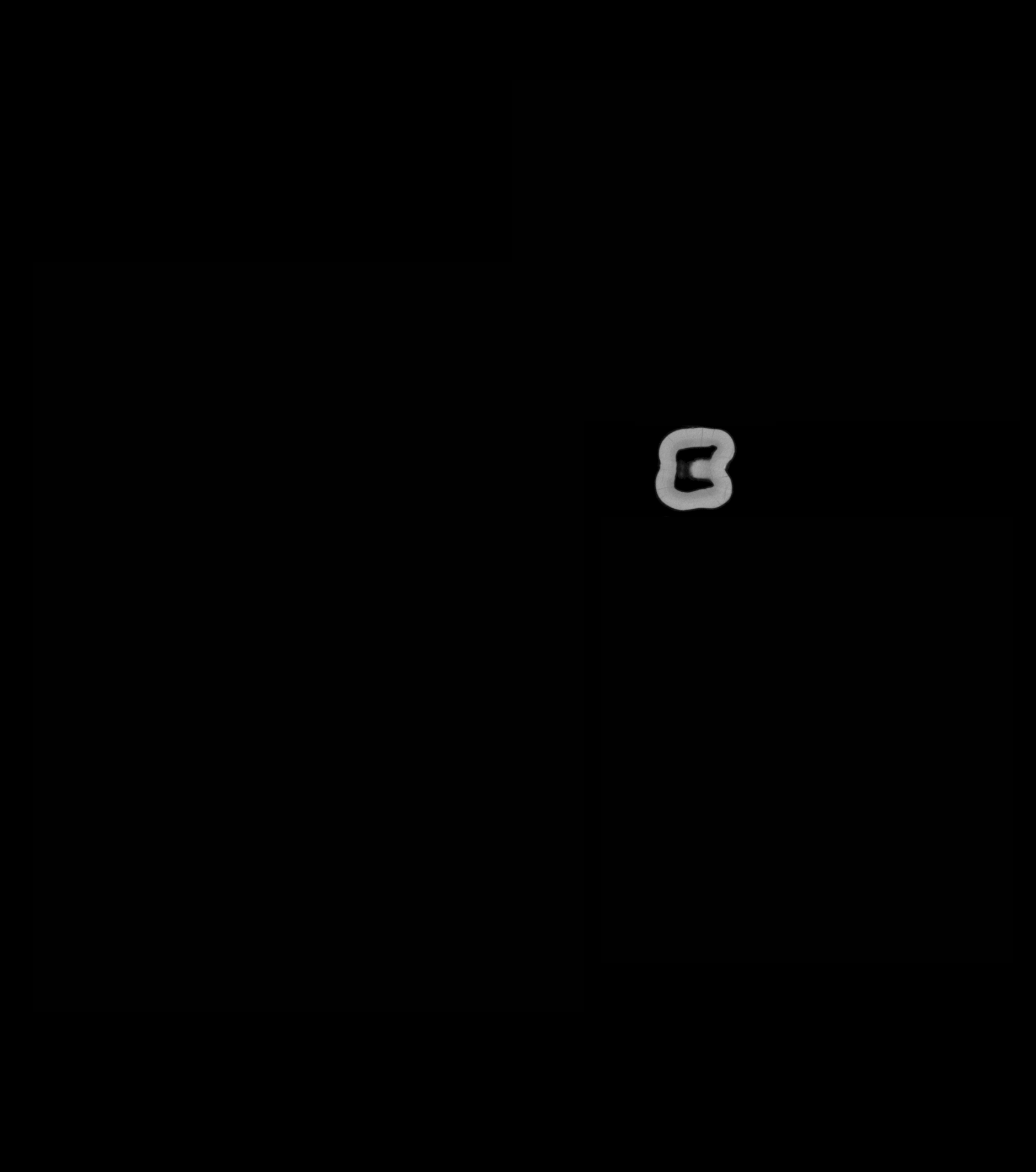

Supplement: Supplementary file 2 — Data S2: Supporting Information. [file AJPA-188-e70164-s001.zip › Cross-Section Tiff Files/amnh_A999687_Rm1.tif]

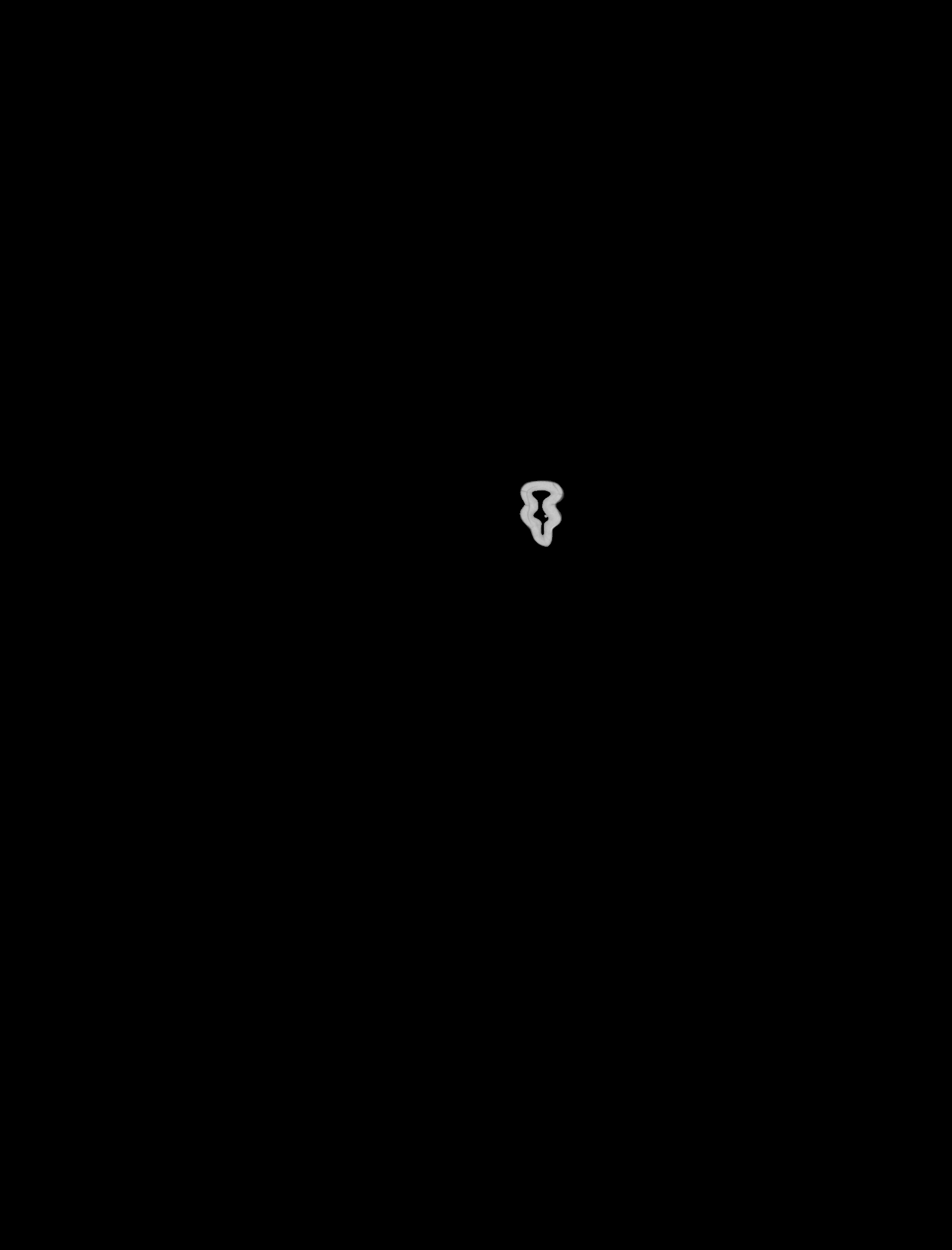

Supplement: Supplementary file 2 — Data S2: Supporting Information. [file AJPA-188-e70164-s001.zip › Cross-Section Tiff Files/amnh_52237_Rm3.tif]

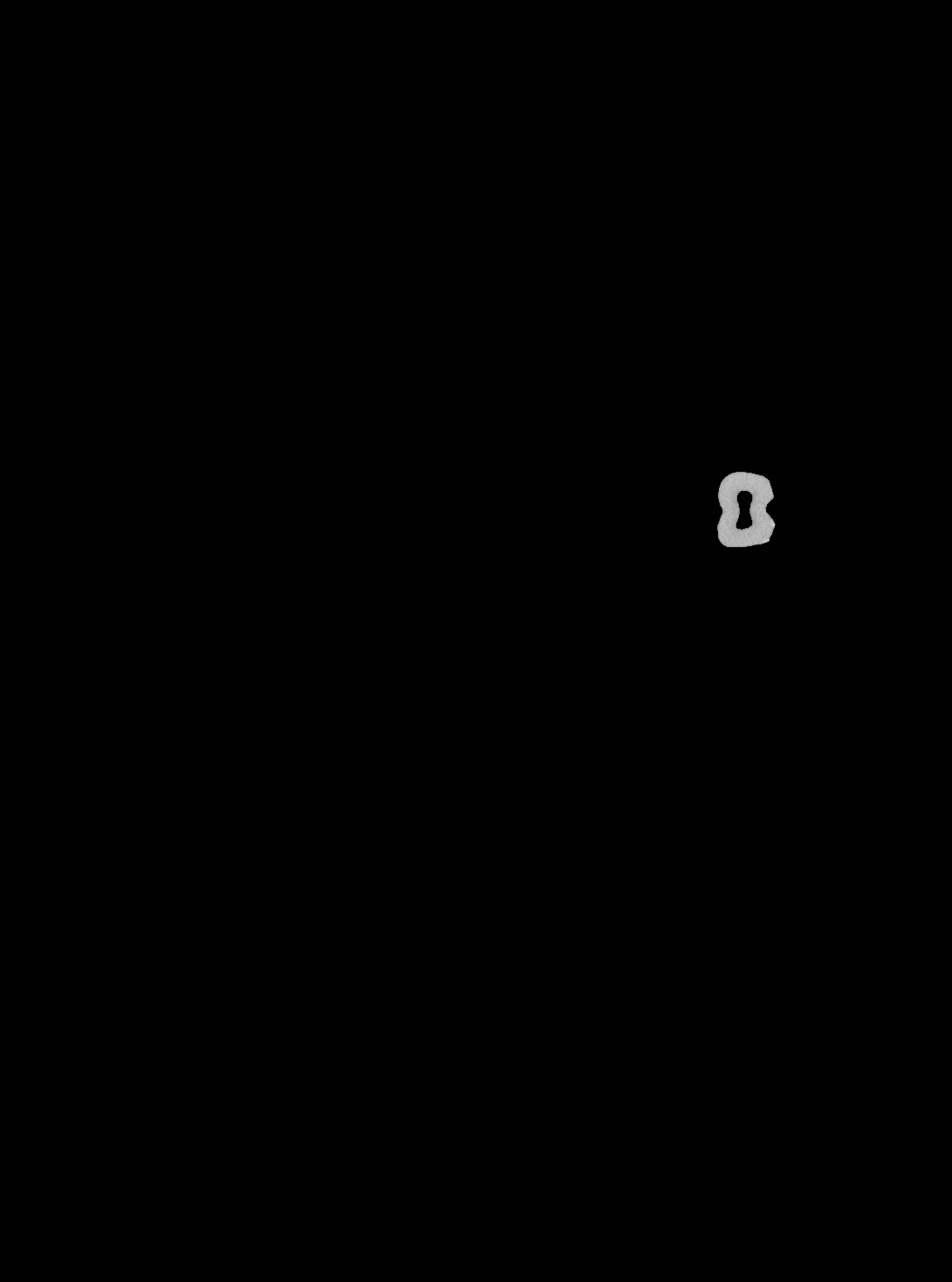

Supplement: Supplementary file 2 — Data S2: Supporting Information. [file AJPA-188-e70164-s001.zip › Cross-Section Tiff Files/mcz_12742_Rm1.tif]

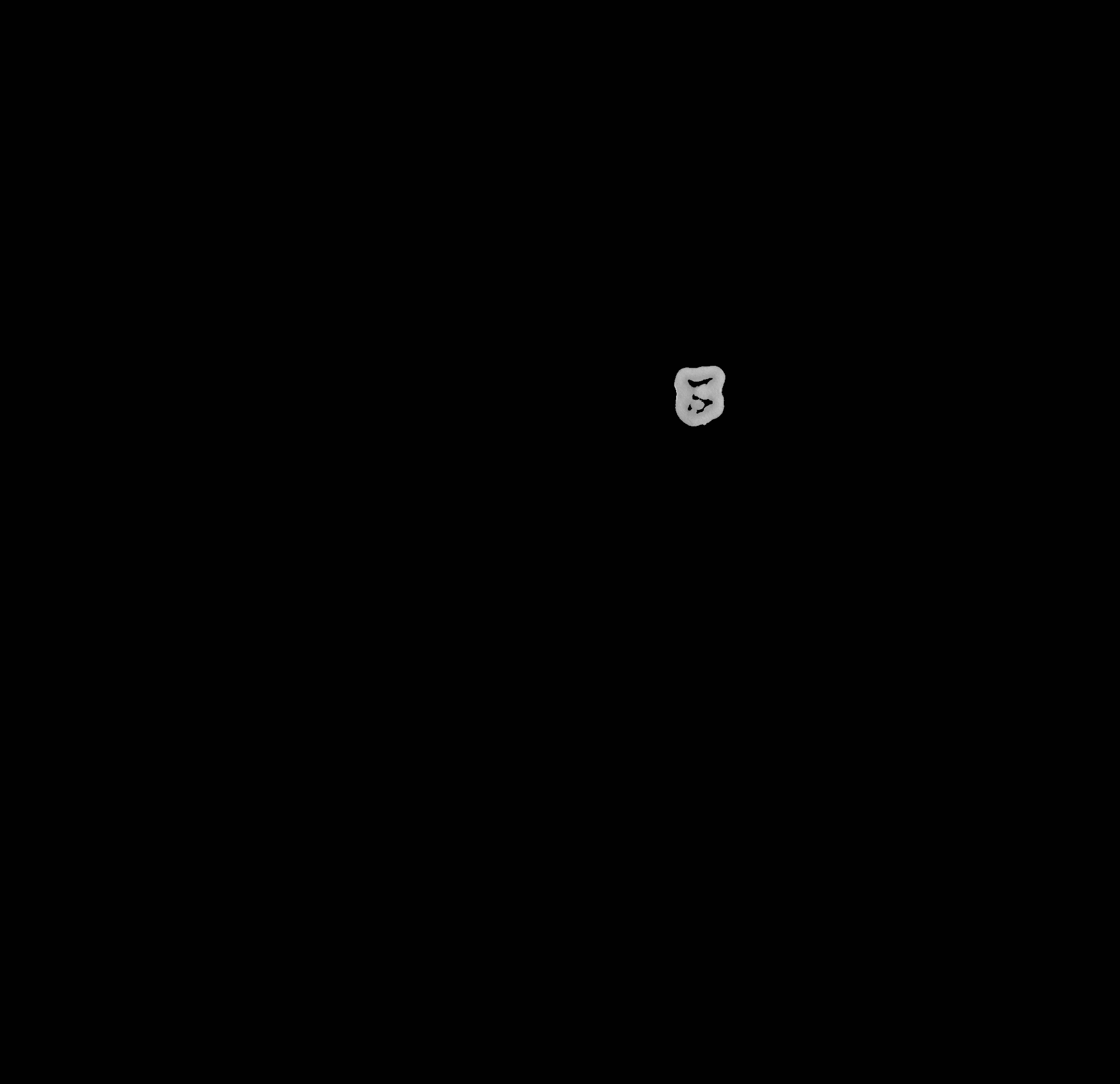

Supplement: Supplementary file 2 — Data S2: Supporting Information. [file AJPA-188-e70164-s001.zip › Cross-Section Tiff Files/mcz_57482_Rm1.tif]

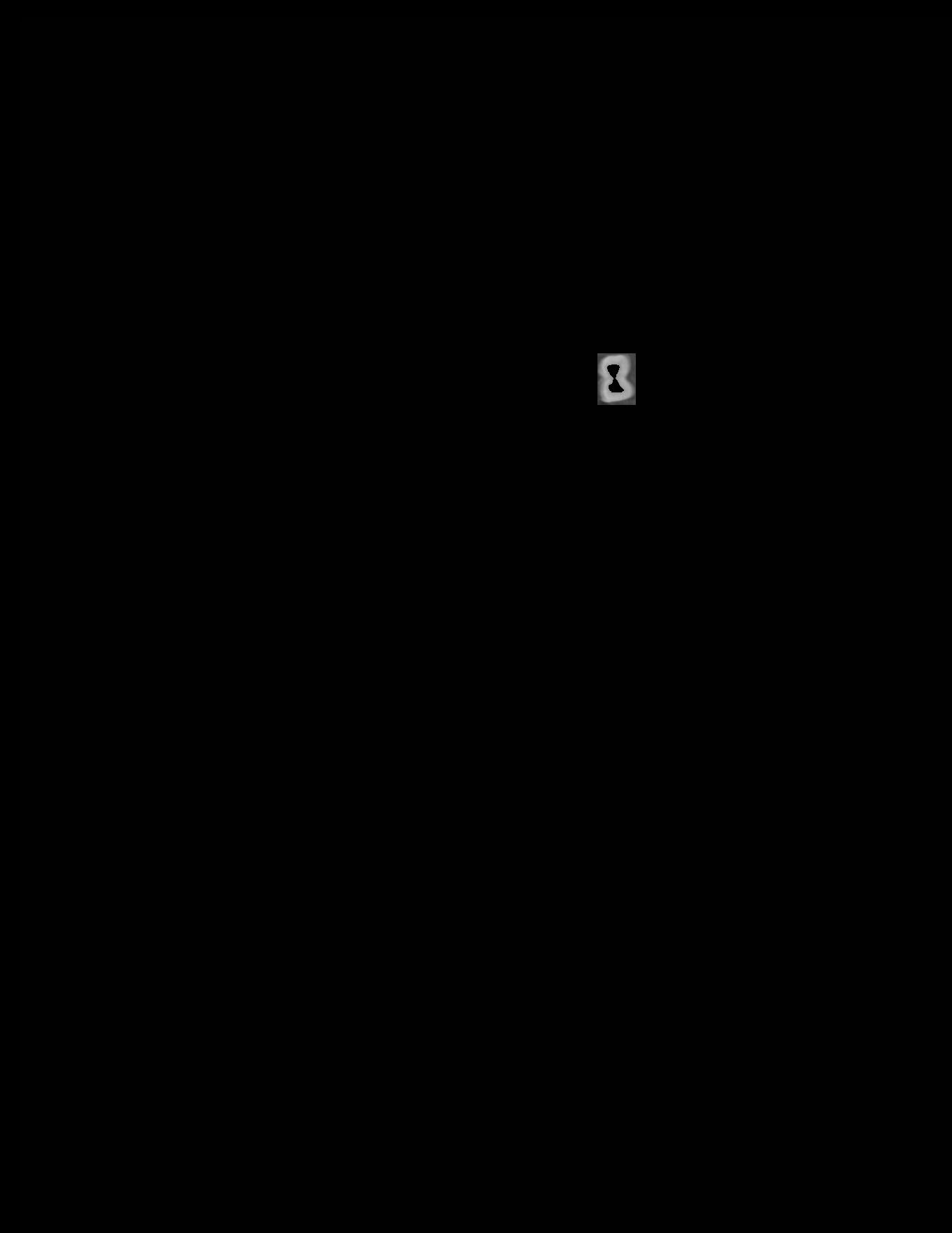

Supplement: Supplementary file 2 — Data S2: Supporting Information. [file AJPA-188-e70164-s001.zip › Cross-Section Tiff Files/mcz_37280_Rm1.tif]

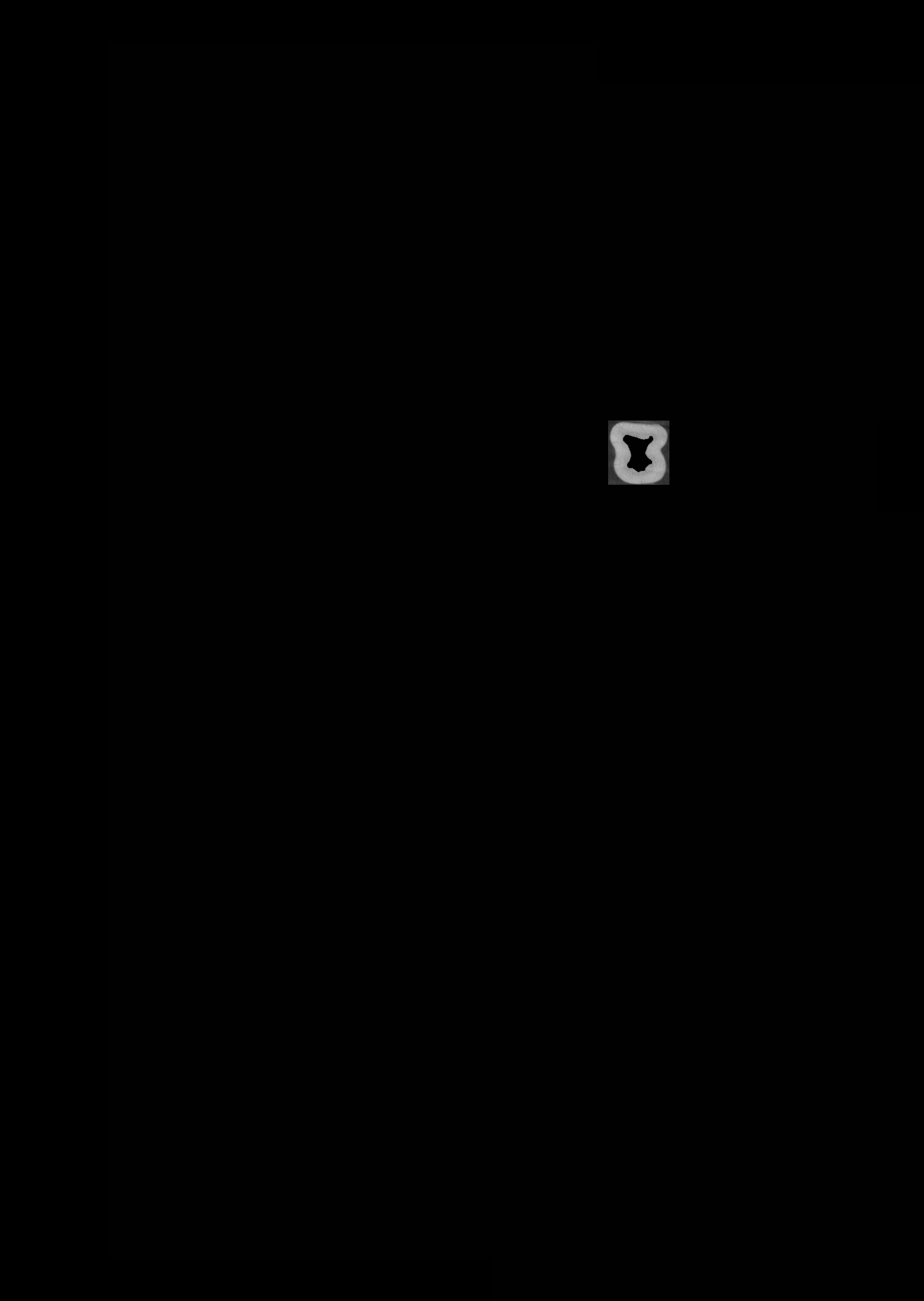

Supplement: Supplementary file 2 — Data S2: Supporting Information. [file AJPA-188-e70164-s001.zip › Cross-Section Tiff Files/mcz_37264_Rm2.tif]

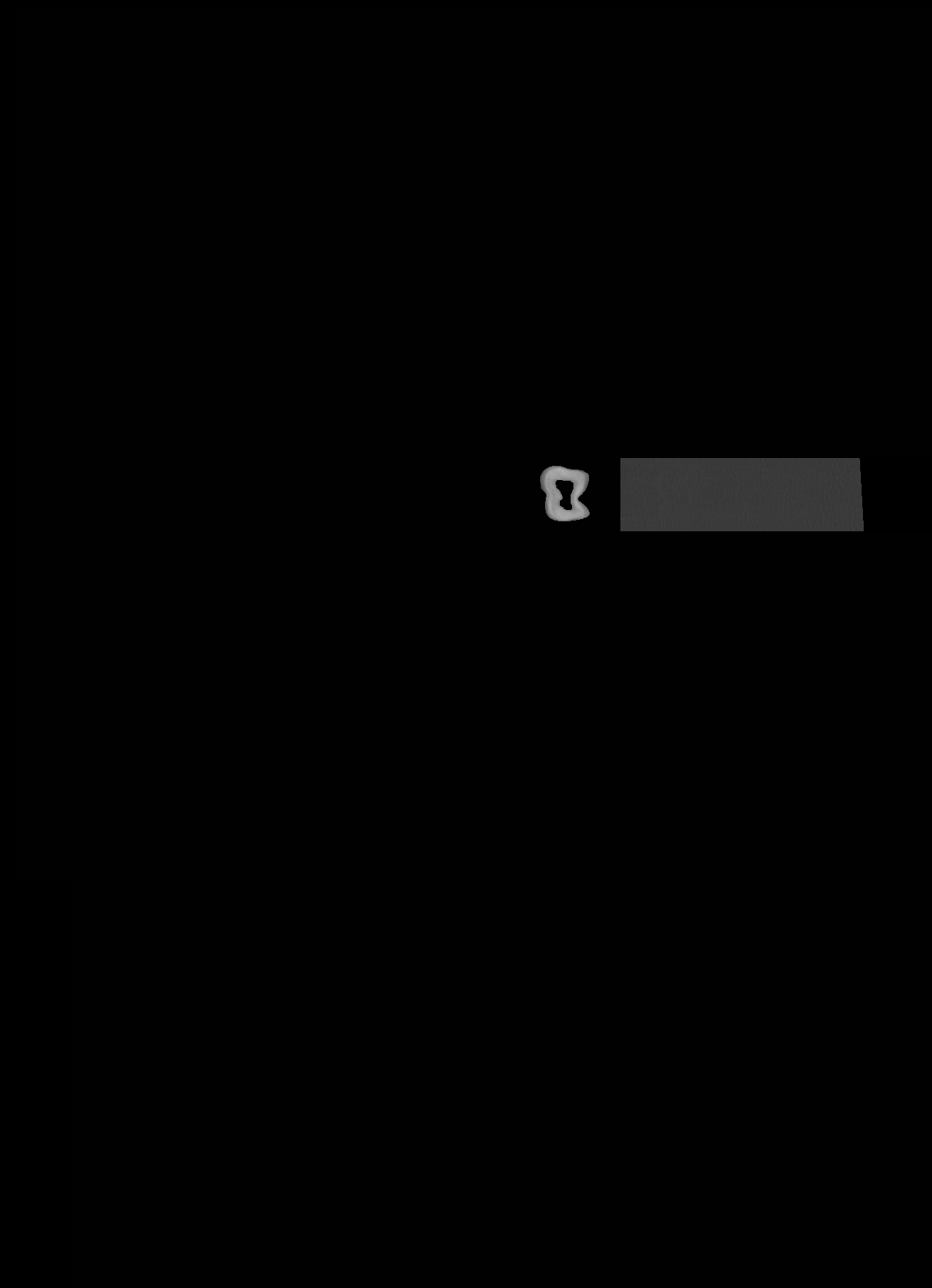

Supplement: Supplementary file 2 — Data S2: Supporting Information. [file AJPA-188-e70164-s001.zip › Cross-Section Tiff Files/mcz_37280_Rm3.tif]

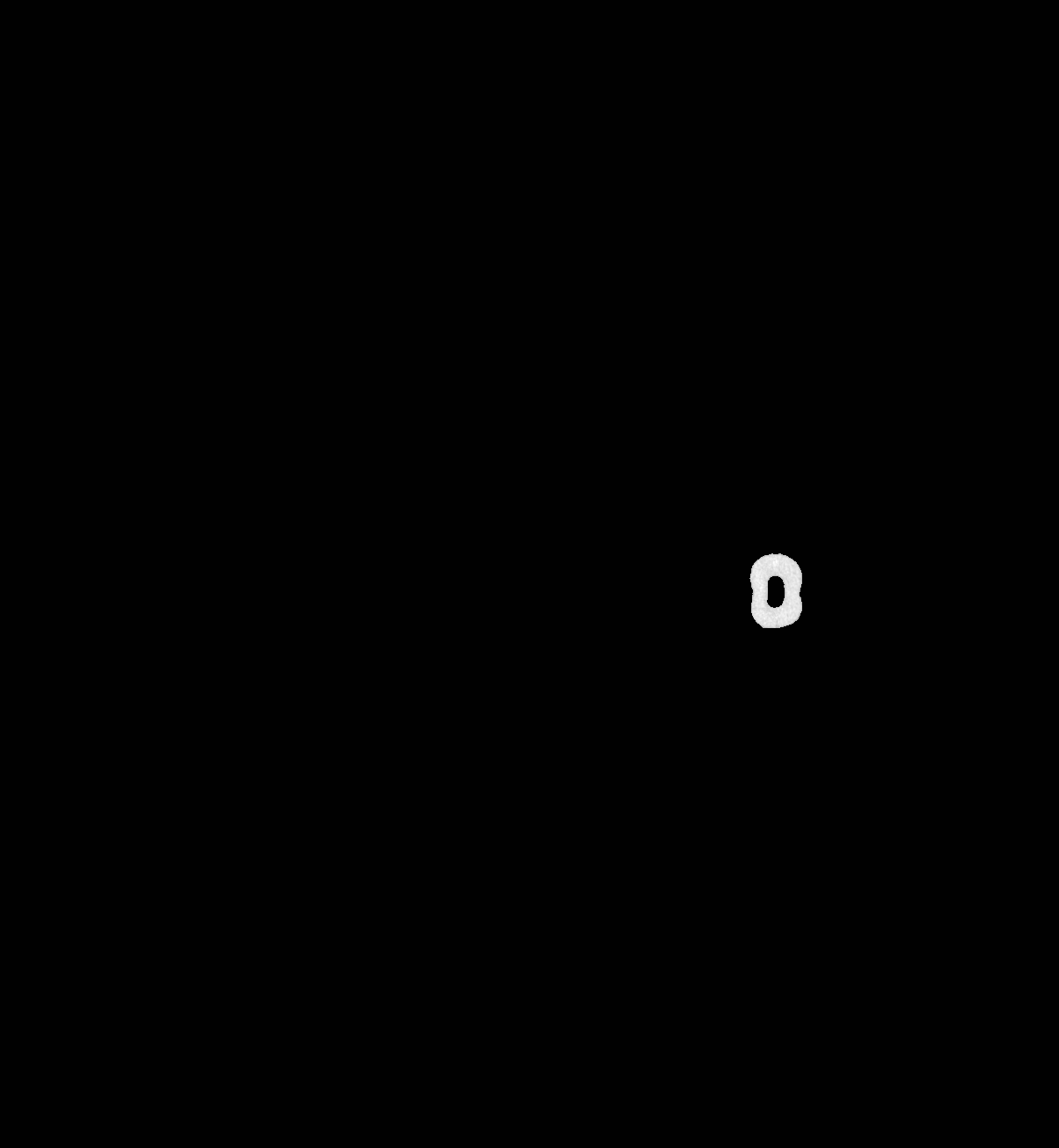

Supplement: Supplementary file 2 — Data S2: Supporting Information. [file AJPA-188-e70164-s001.zip › Cross-Section Tiff Files/mcz_12742_Rm3.tif]

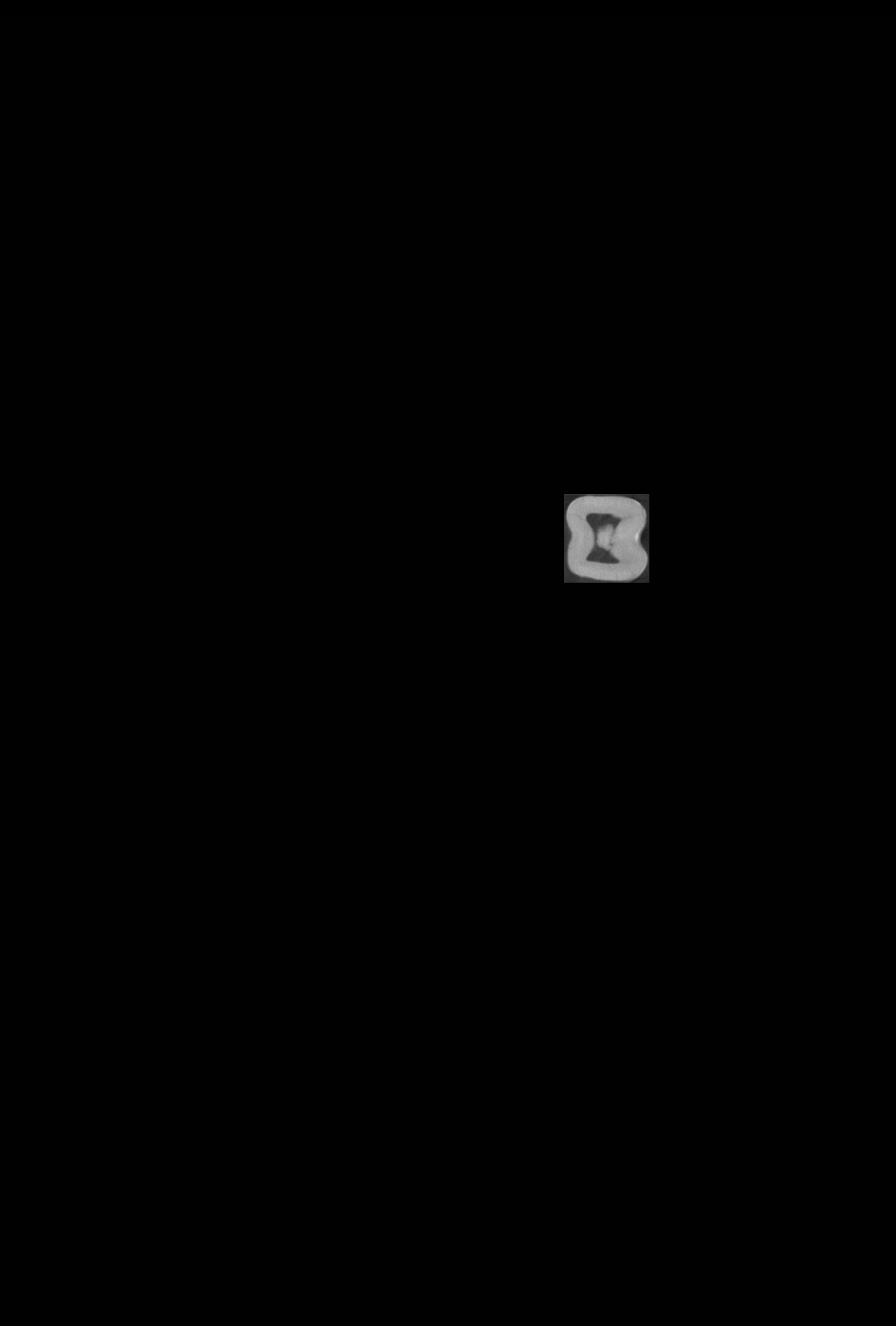

Supplement: Supplementary file 2 — Data S2: Supporting Information. [file AJPA-188-e70164-s001.zip › Cross-Section Tiff Files/mcz_29786_Rm2.tif]

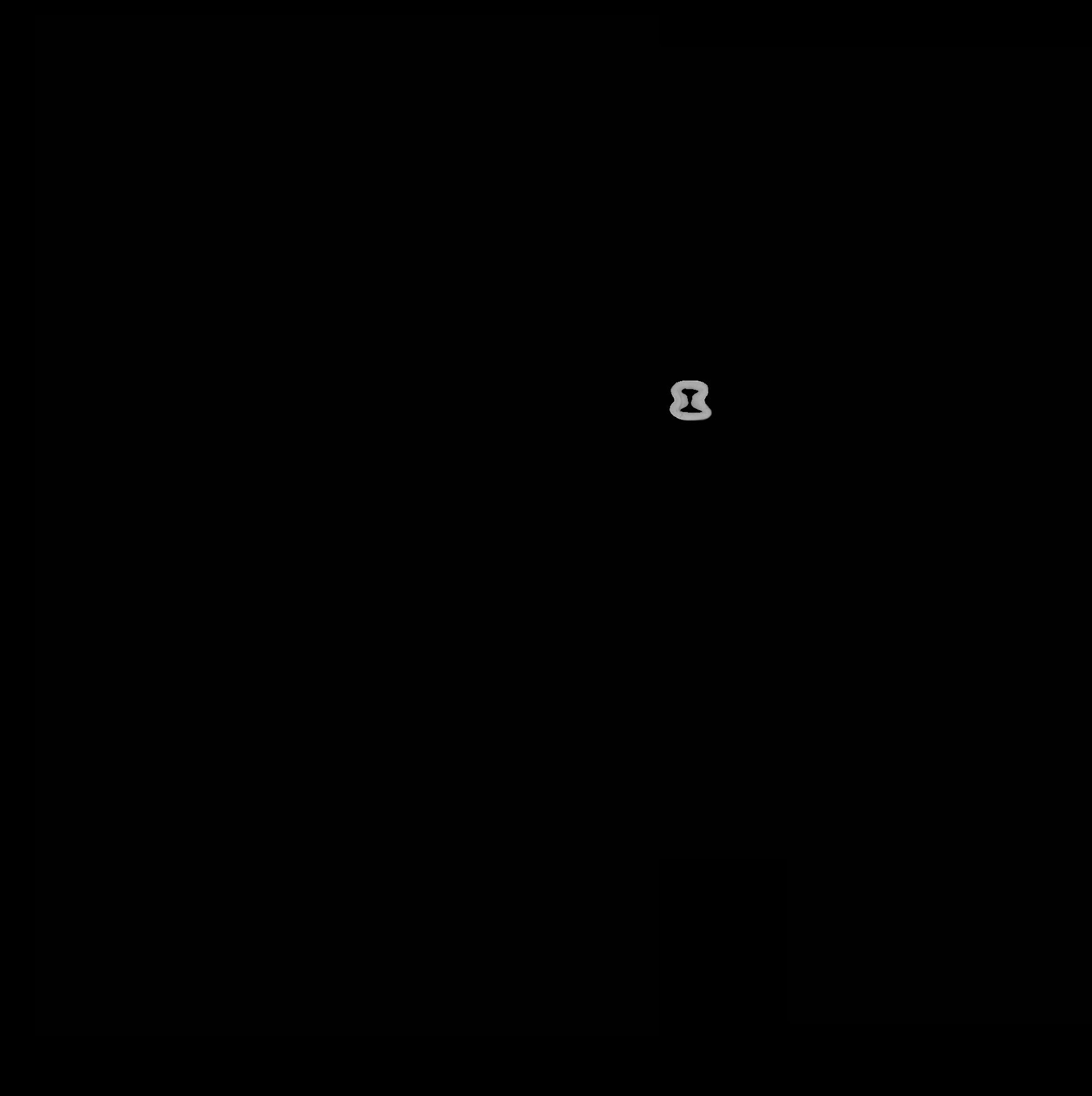

Supplement: Supplementary file 2 — Data S2: Supporting Information. [file AJPA-188-e70164-s001.zip › Cross-Section Tiff Files/amnh_52237_Rm1.tif]

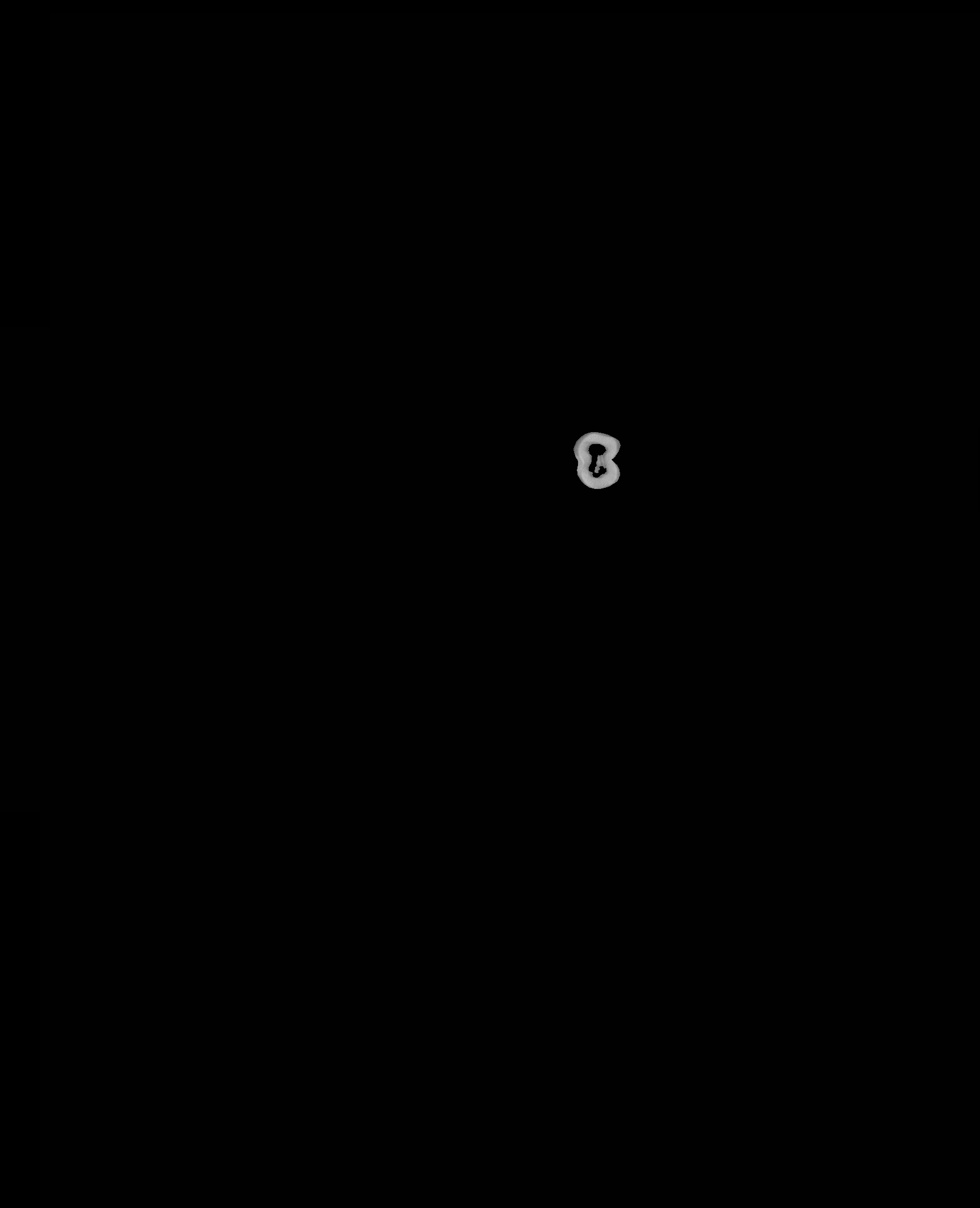

Supplement: Supplementary file 2 — Data S2: Supporting Information. [file AJPA-188-e70164-s001.zip › Cross-Section Tiff Files/mcz_36031_Rm2.tif]

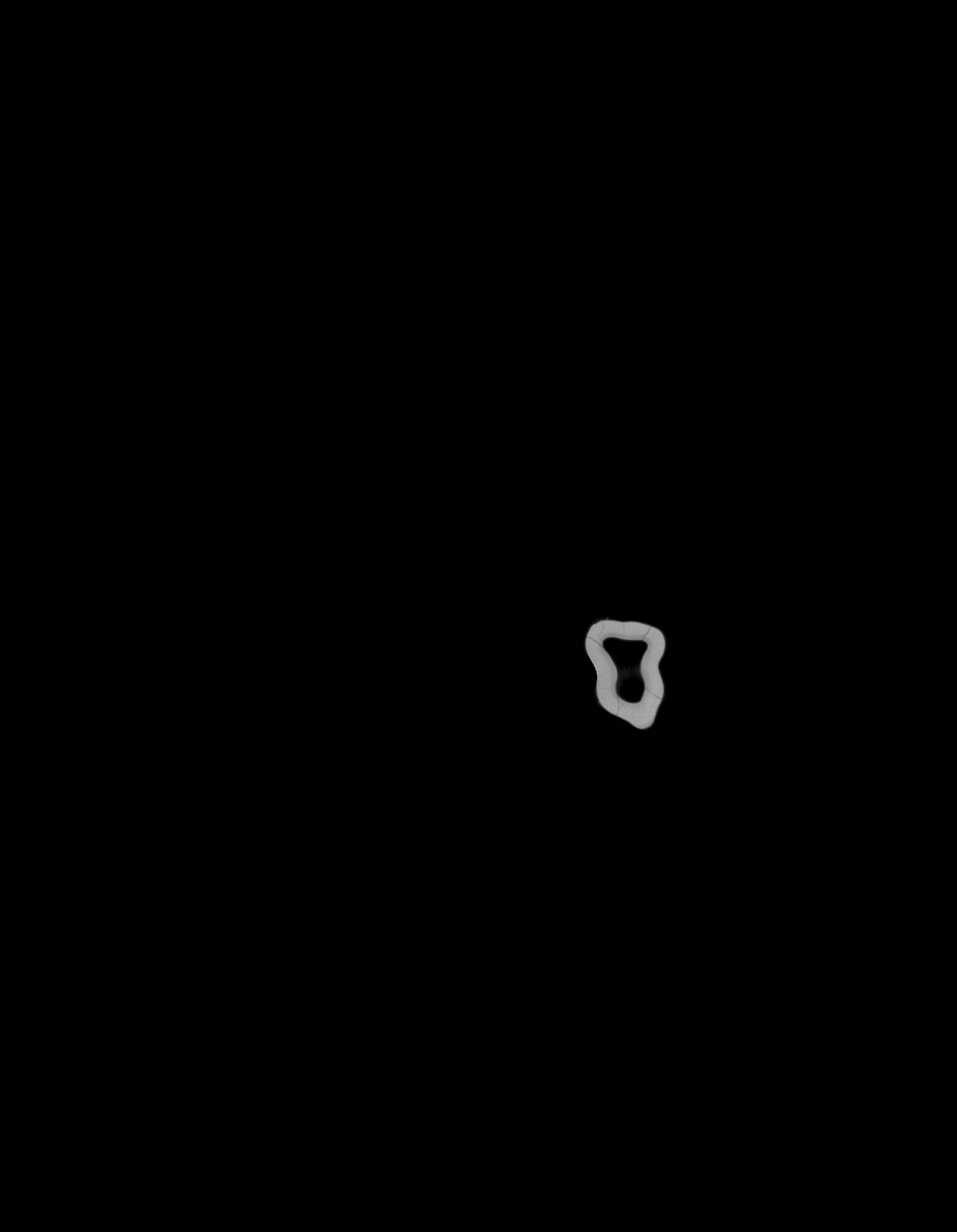

Supplement: Supplementary file 2 — Data S2: Supporting Information. [file AJPA-188-e70164-s001.zip › Cross-Section Tiff Files/amnh_A999687_Rm3.tif]

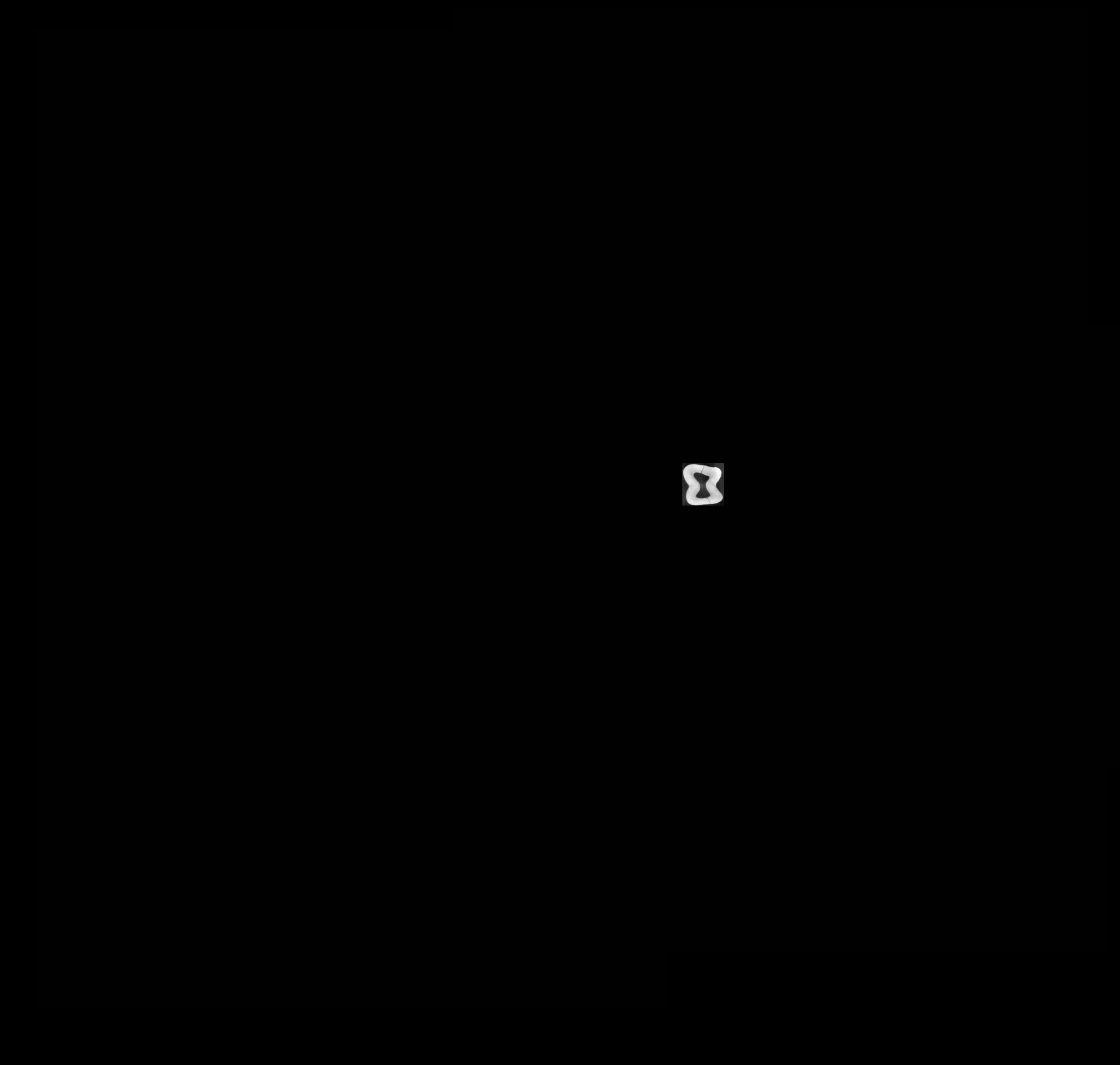

Supplement: Supplementary file 2 — Data S2: Supporting Information. [file AJPA-188-e70164-s001.zip › Cross-Section Tiff Files/amnh_90309_Rm2.tif]

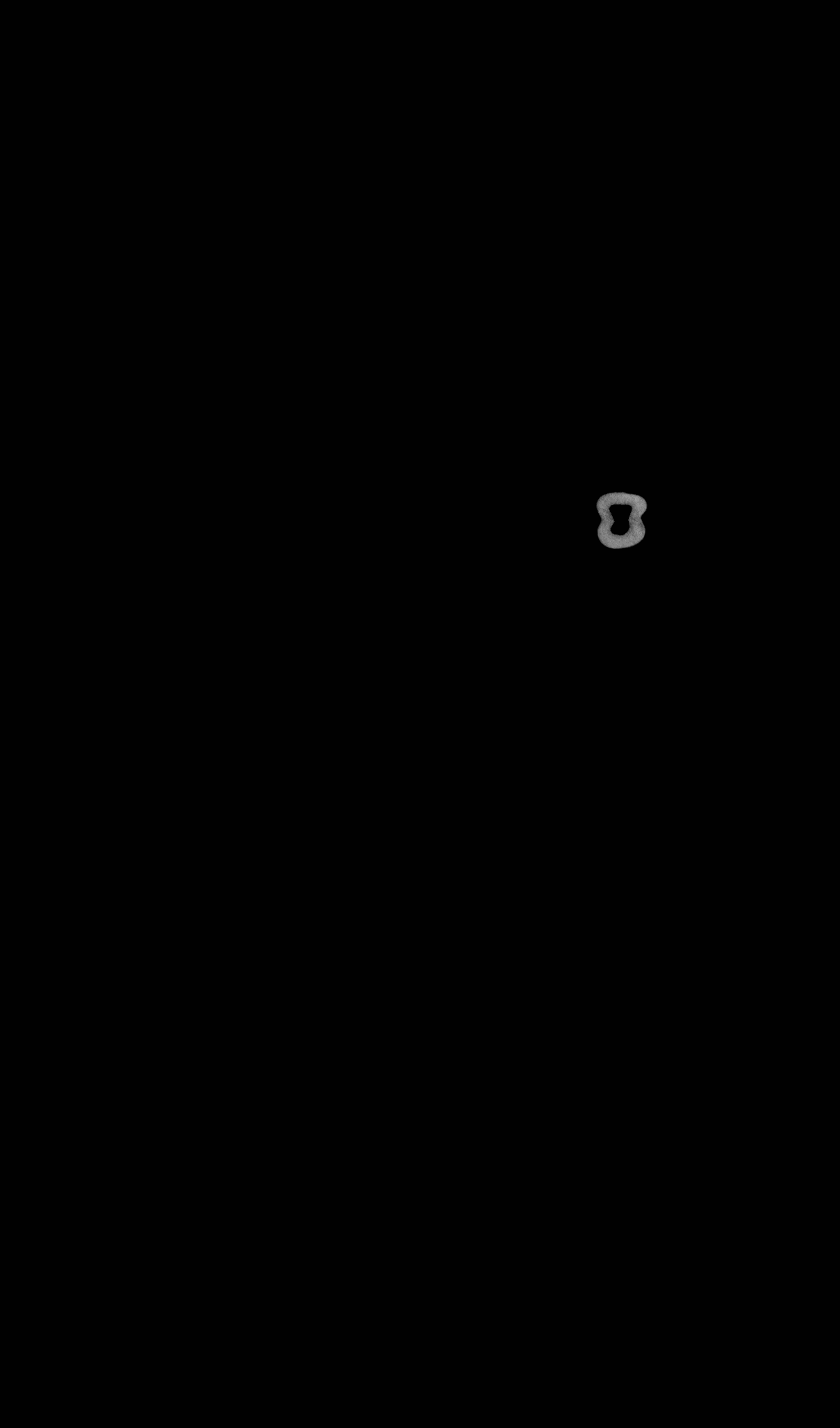

Supplement: Supplementary file 2 — Data S2: Supporting Information. [file AJPA-188-e70164-s001.zip › Cross-Section Tiff Files/mcz_15312_Rm2.tif]

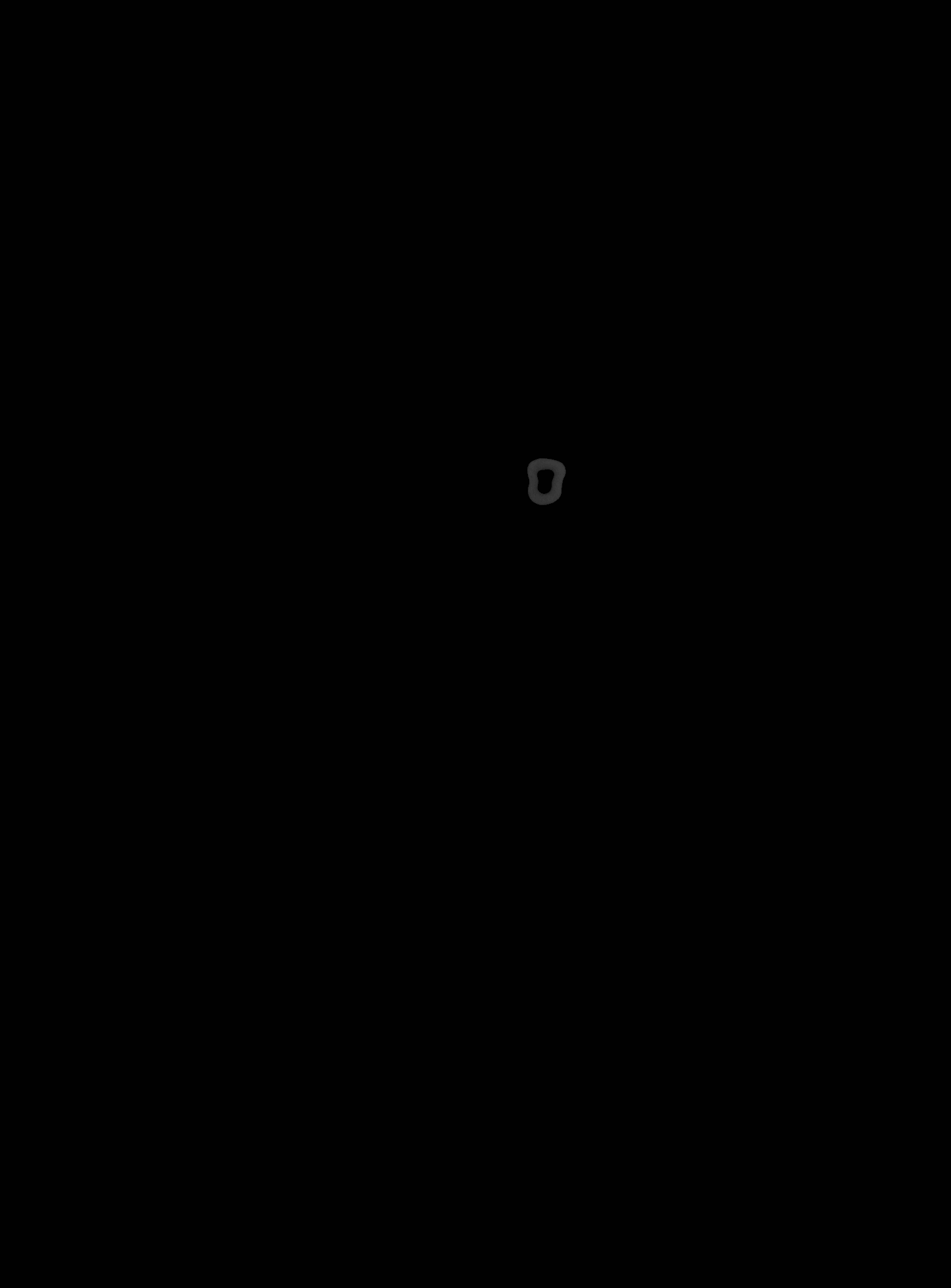

Supplement: Supplementary file 2 — Data S2: Supporting Information. [file AJPA-188-e70164-s001.zip › Cross-Section Tiff Files/mcz_15312_Rm3.tif]

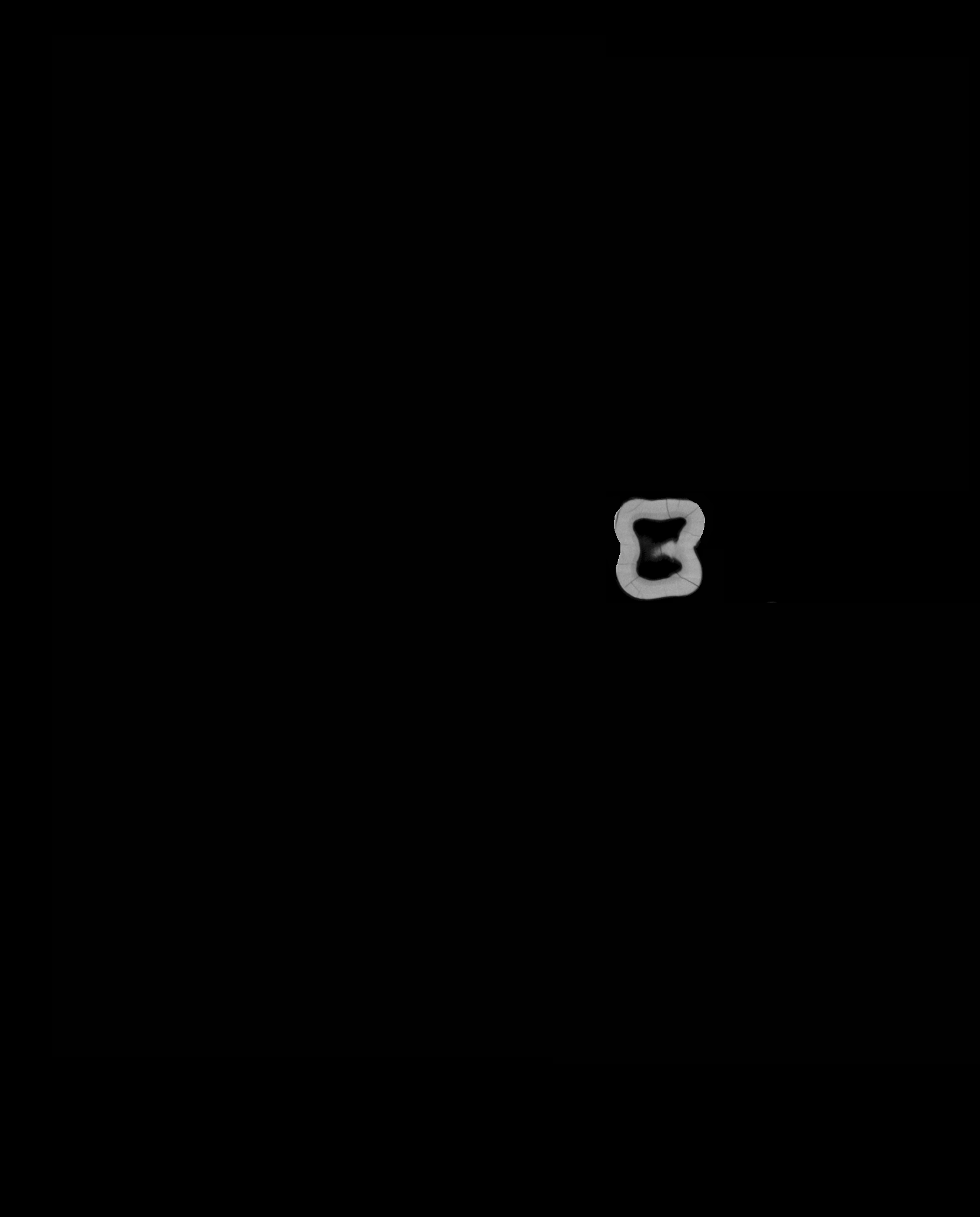

Supplement: Supplementary file 2 — Data S2: Supporting Information. [file AJPA-188-e70164-s001.zip › Cross-Section Tiff Files/amnh_A999687_Rm2.tif]

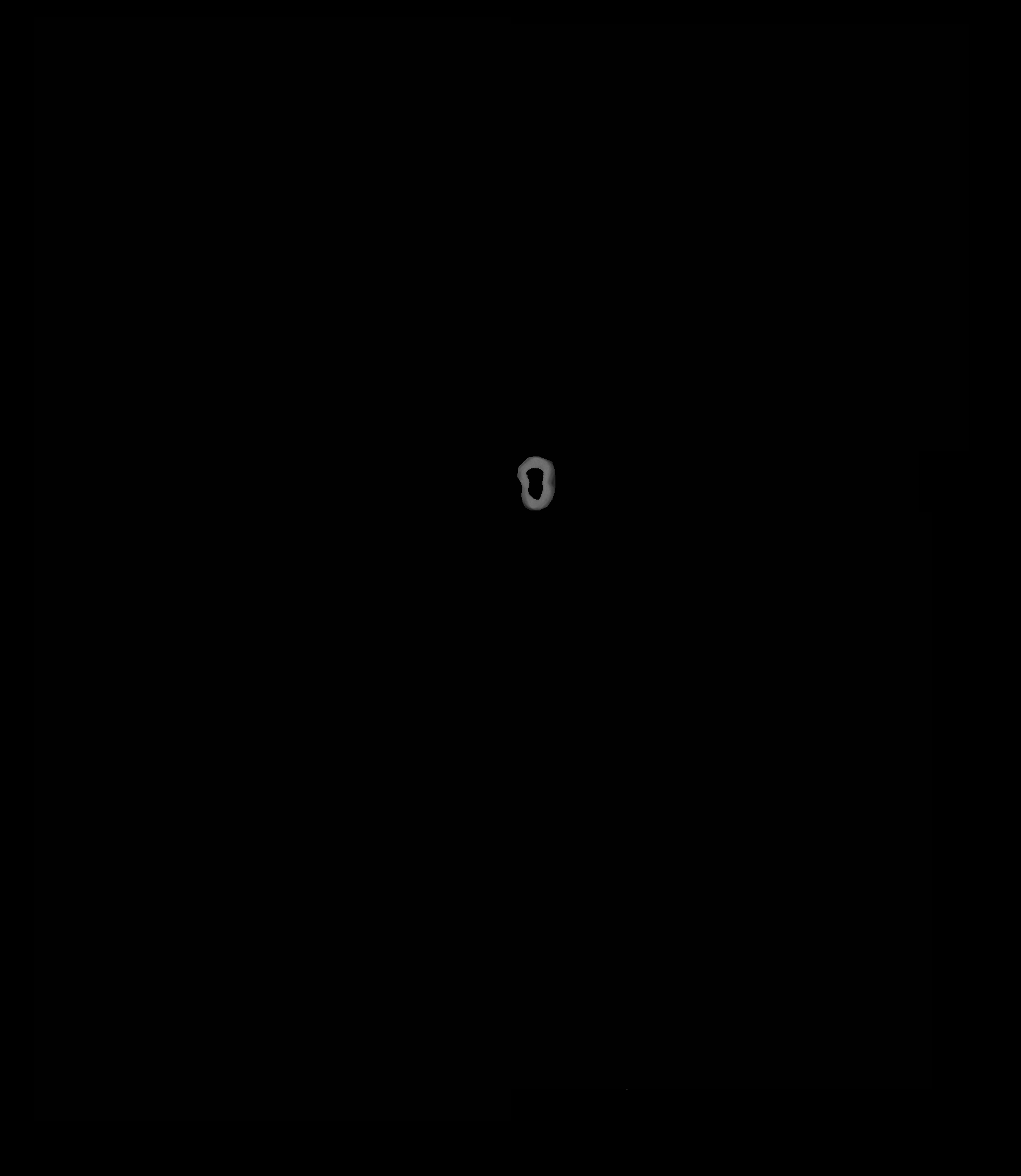

Supplement: Supplementary file 2 — Data S2: Supporting Information. [file AJPA-188-e70164-s001.zip › Cross-Section Tiff Files/mcz_36031_Rm3.tif]

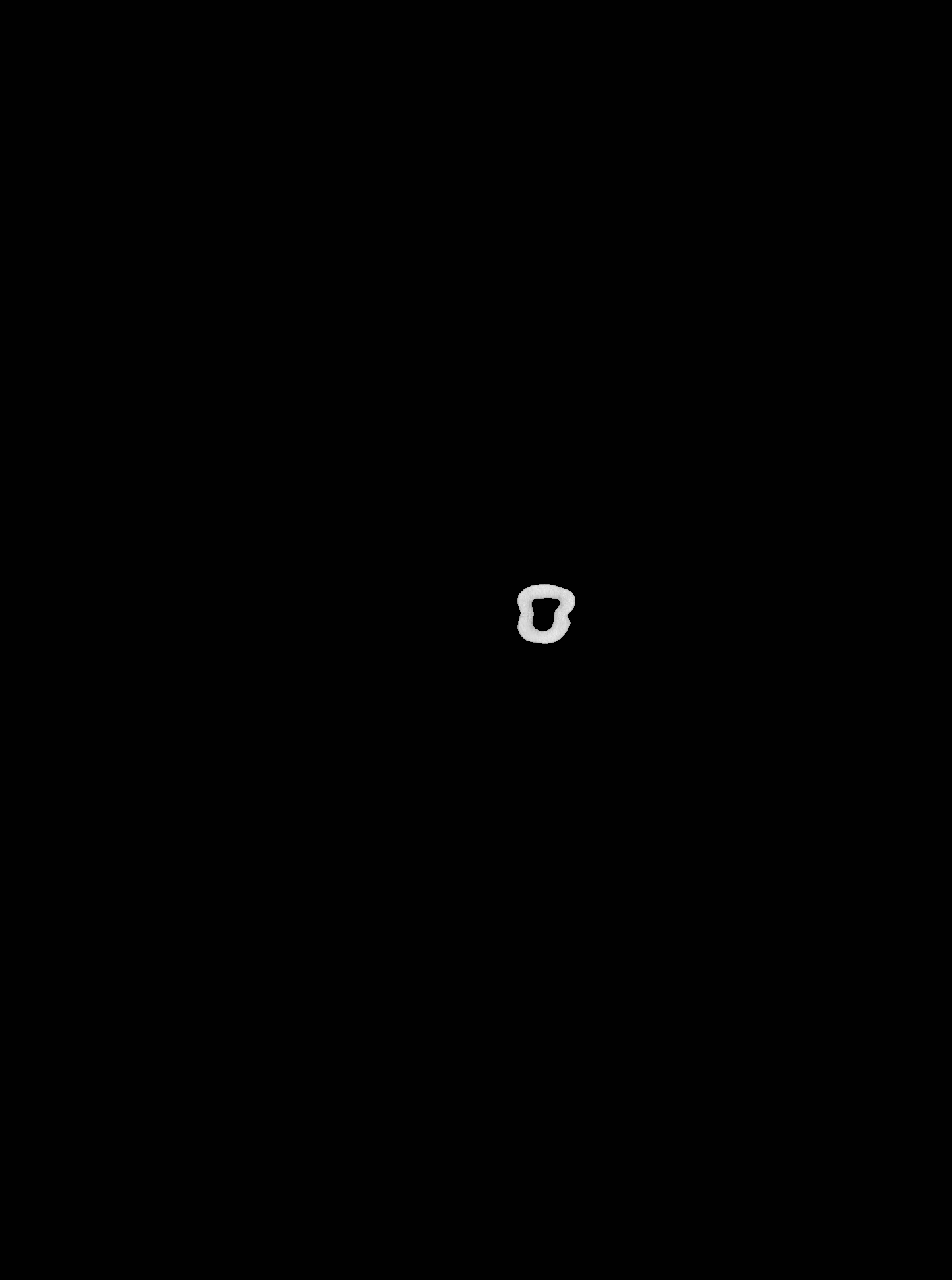

Supplement: Supplementary file 2 — Data S2: Supporting Information. [file AJPA-188-e70164-s001.zip › Cross-Section Tiff Files/mcz_20041_Rm3.tif]

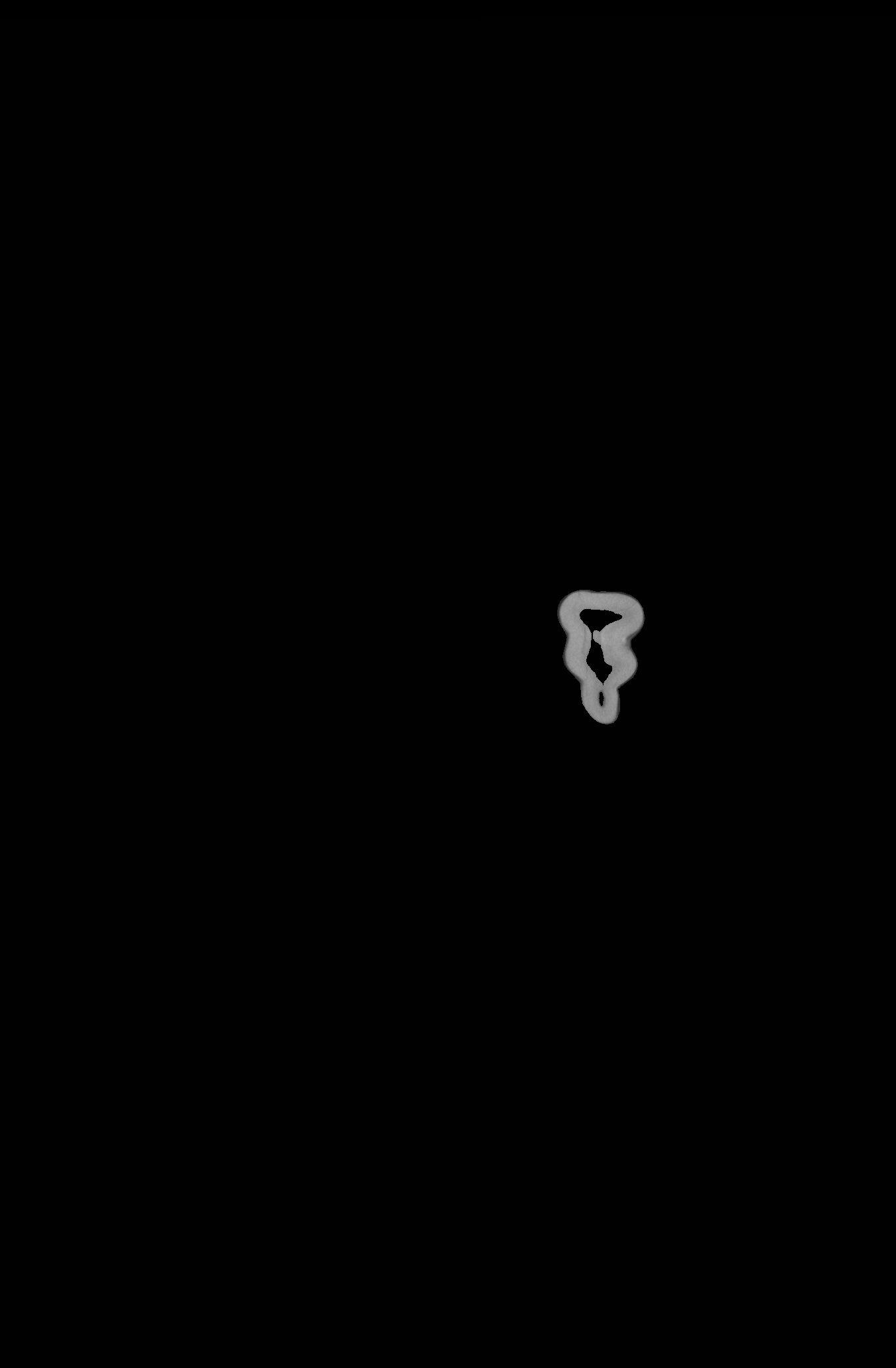

Supplement: Supplementary file 2 — Data S2: Supporting Information. [file AJPA-188-e70164-s001.zip › Cross-Section Tiff Files/mcz_29786_Rm3.tif]

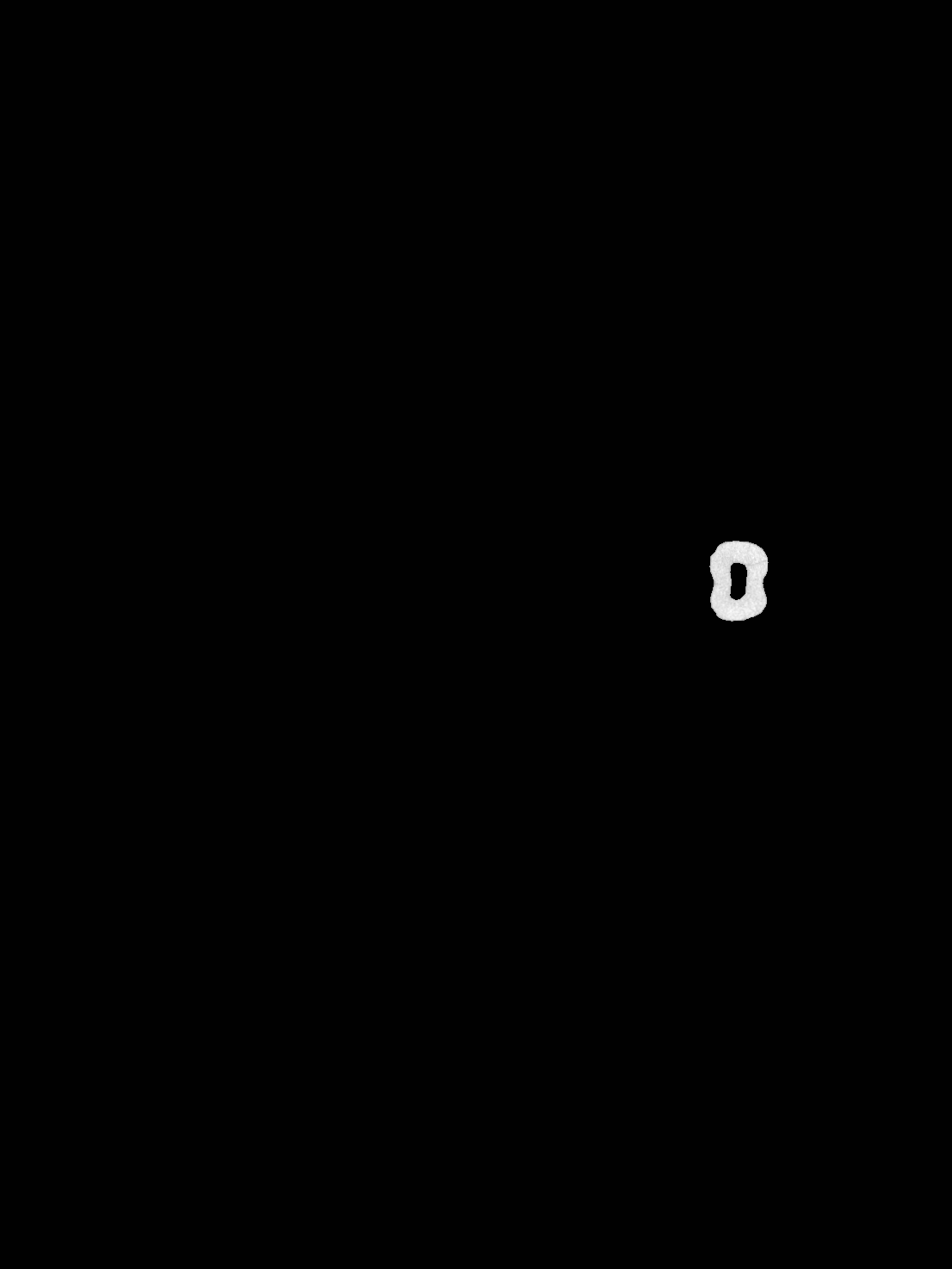

Supplement: Supplementary file 2 — Data S2: Supporting Information. [file AJPA-188-e70164-s001.zip › Cross-Section Tiff Files/mcz_12742_Rm2.tif]

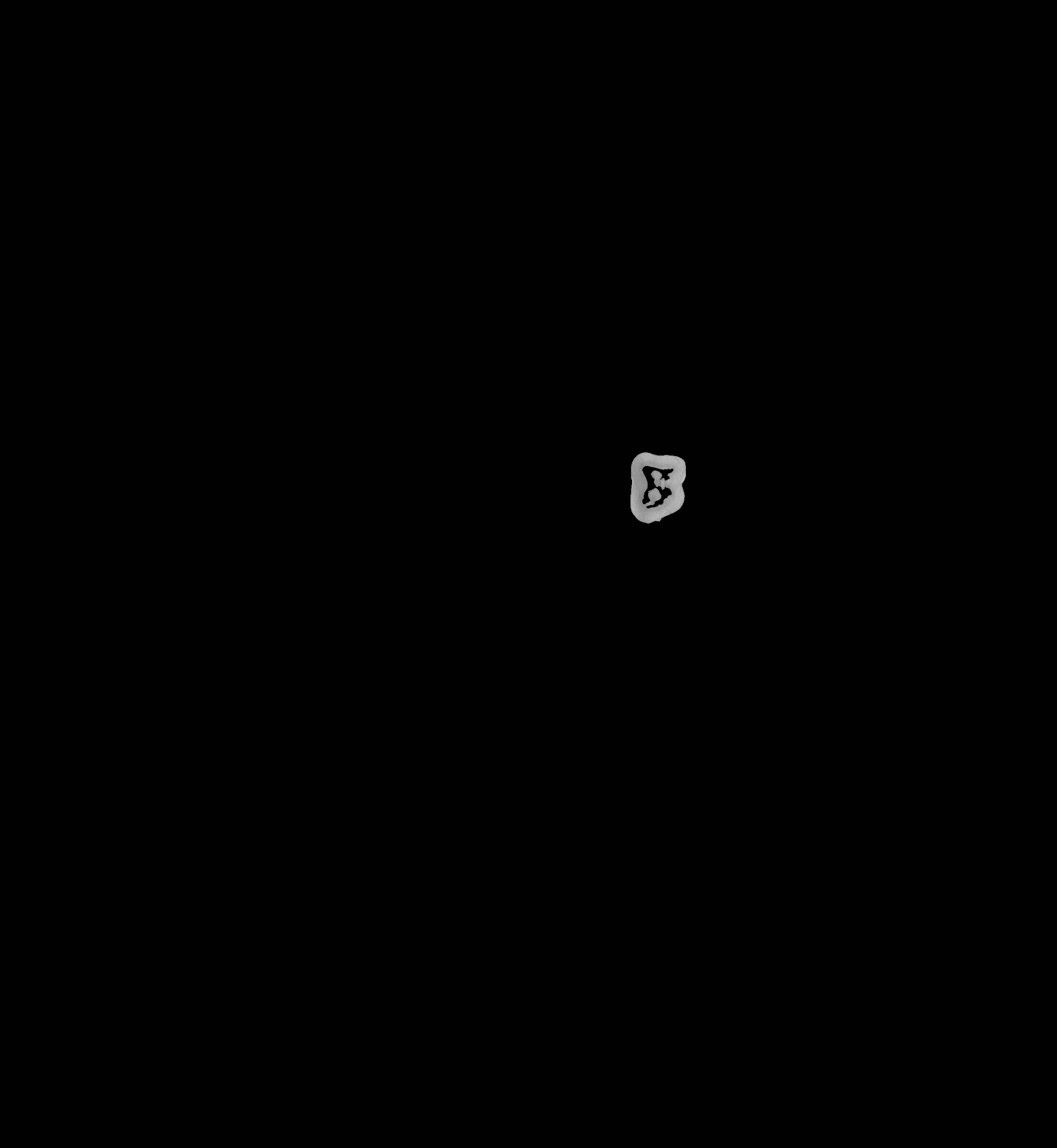

Supplement: Supplementary file 2 — Data S2: Supporting Information. [file AJPA-188-e70164-s001.zip › Cross-Section Tiff Files/mcz_57482_Rm2.tif]

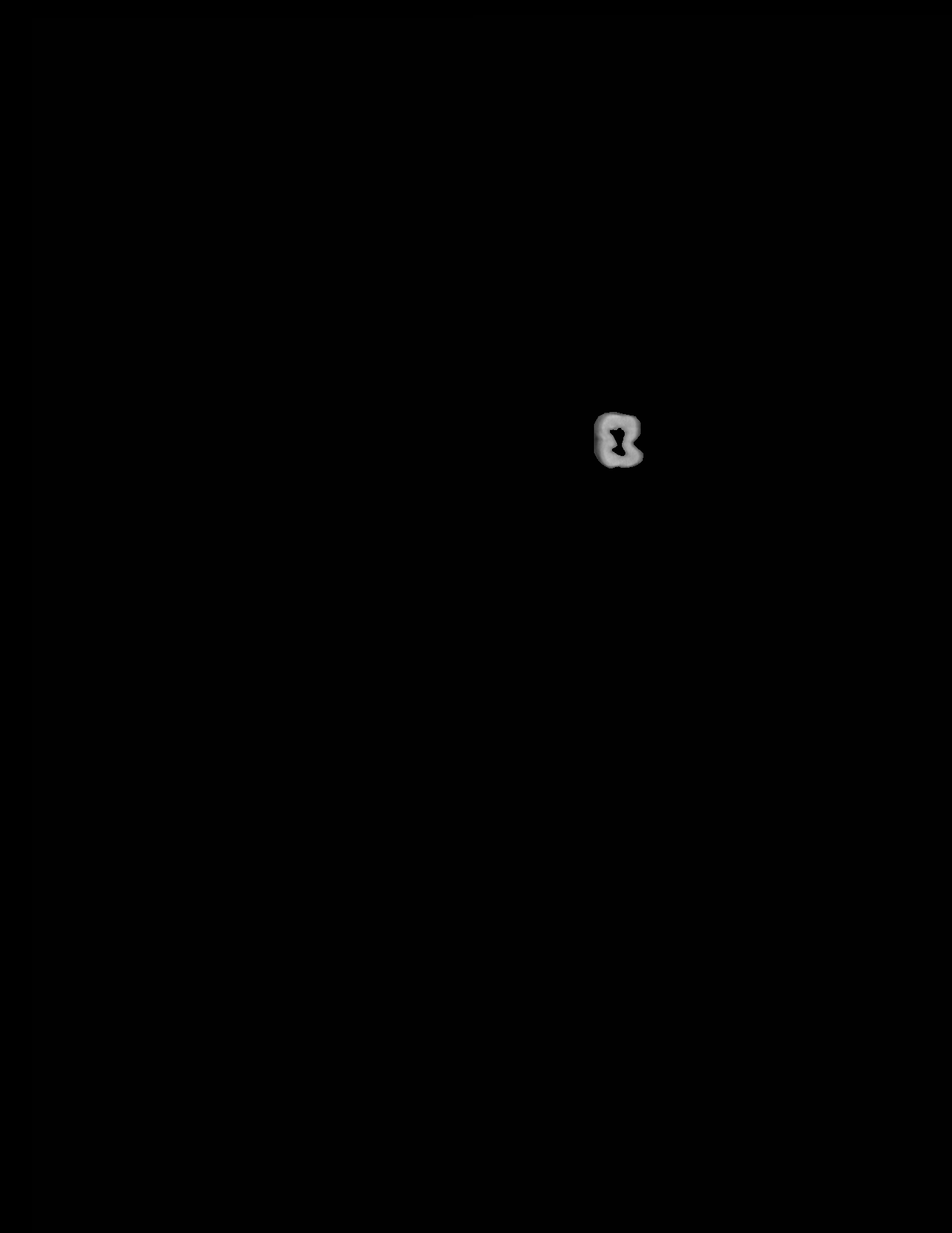

Supplement: Supplementary file 2 — Data S2: Supporting Information. [file AJPA-188-e70164-s001.zip › Cross-Section Tiff Files/mcz_37280_Rm2.tif]

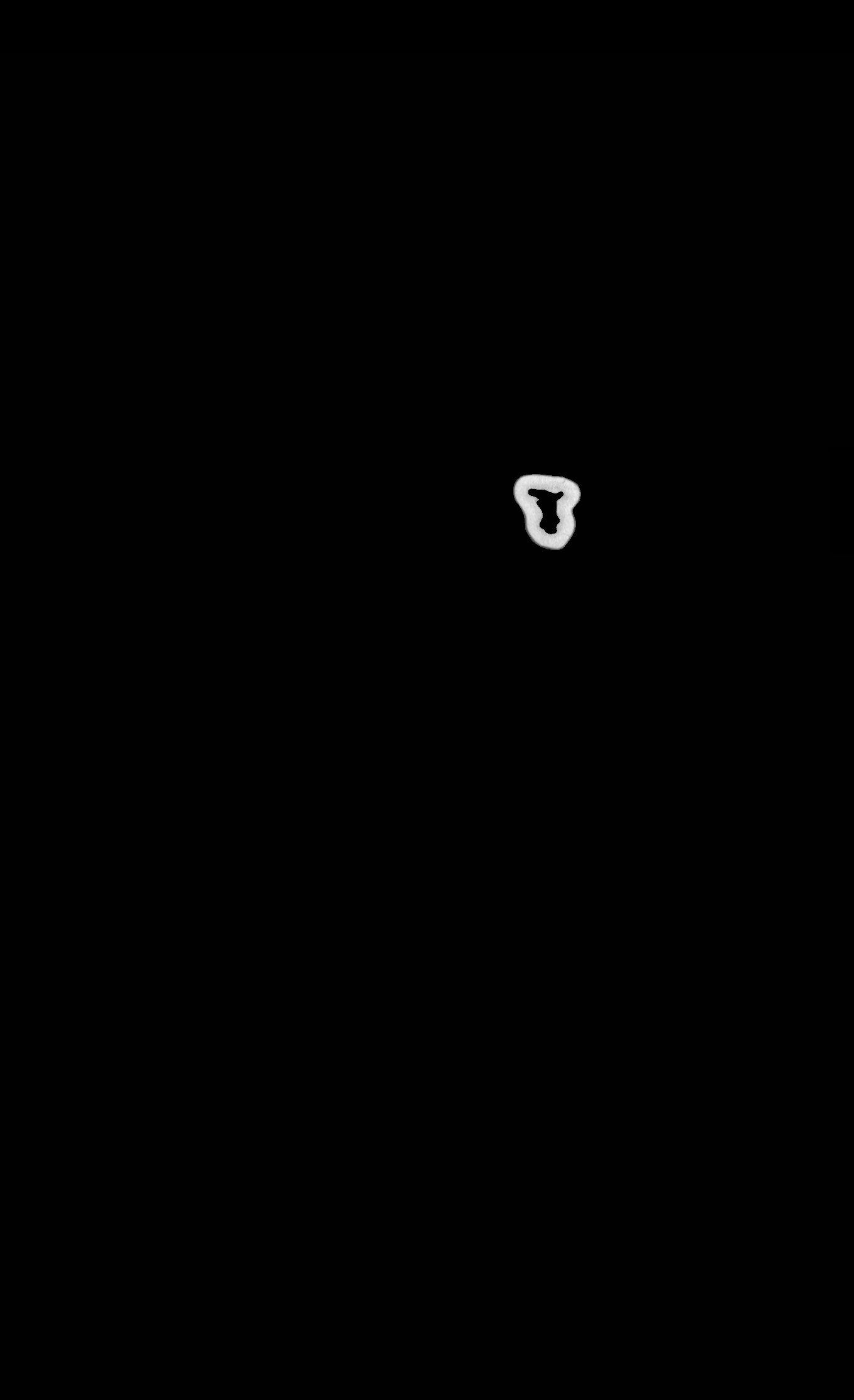

Supplement: Supplementary file 2 — Data S2: Supporting Information. [file AJPA-188-e70164-s001.zip › Cross-Section Tiff Files/mcz_37264_Rm3.tif]

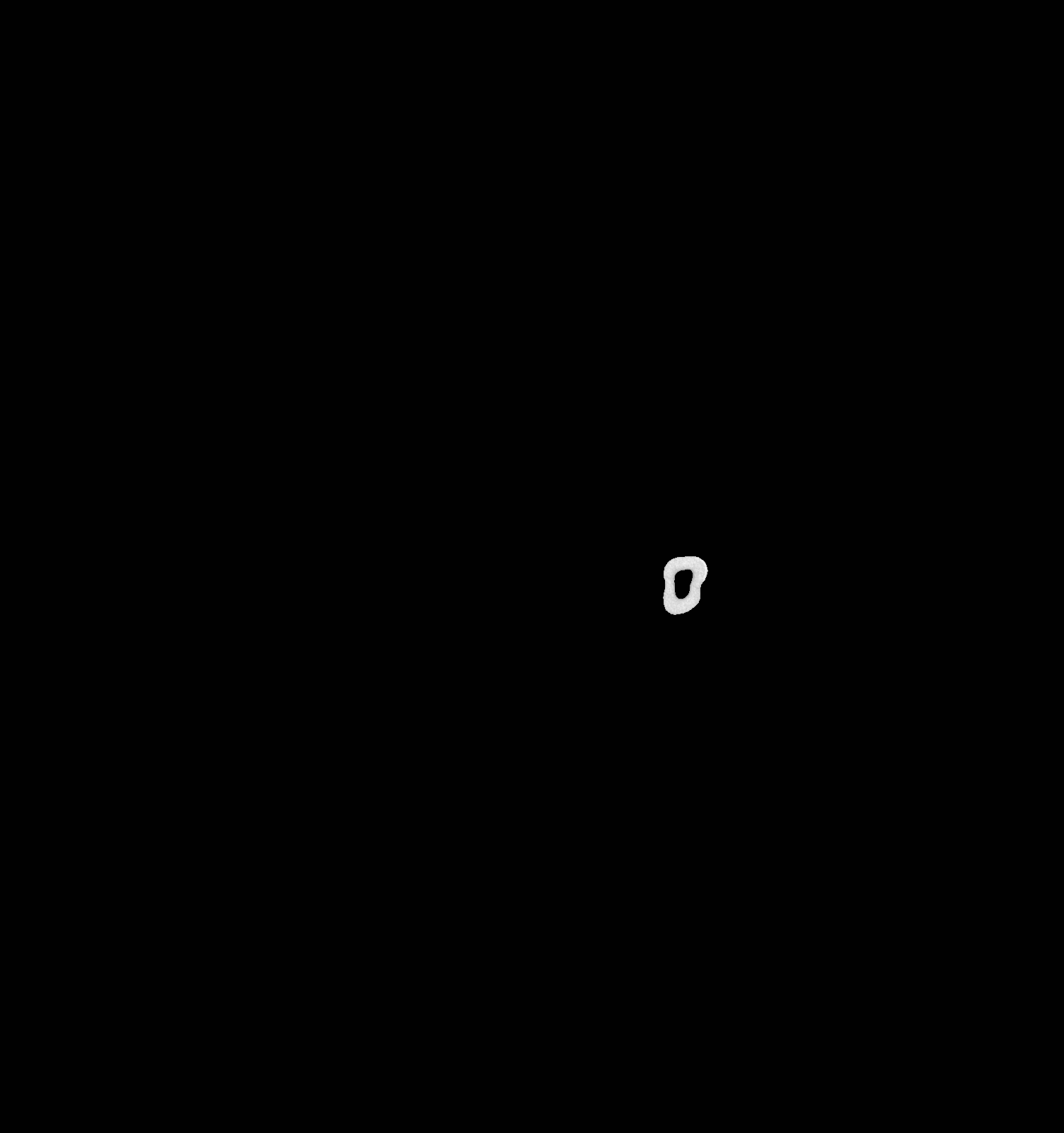

Supplement: Supplementary file 2 — Data S2: Supporting Information. [file AJPA-188-e70164-s001.zip › Cross-Section Tiff Files/mcz_41428_Rm3.tif]

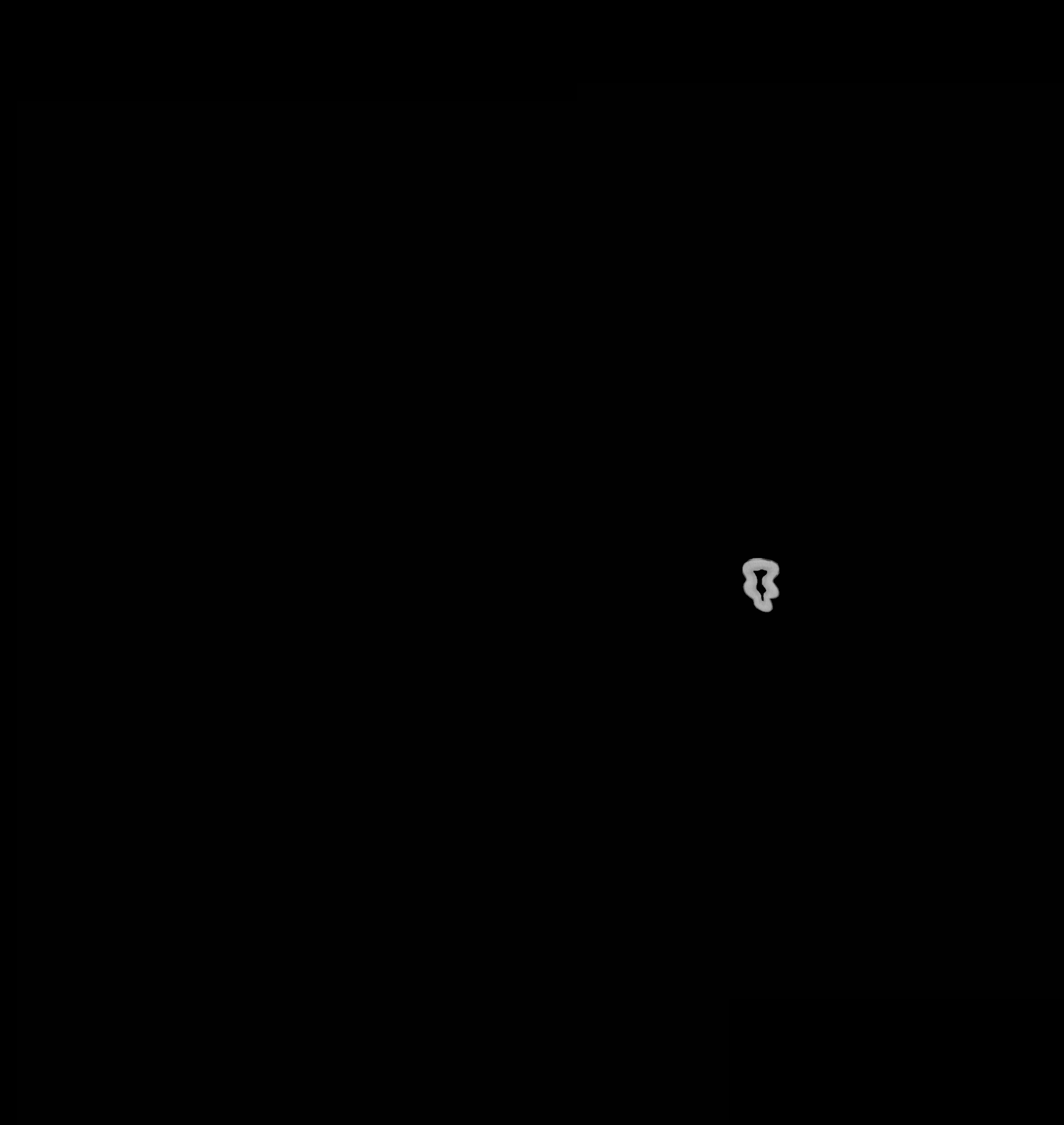

Supplement: Supplementary file 2 — Data S2: Supporting Information. [file AJPA-188-e70164-s001.zip › Cross-Section Tiff Files/amnh_52210_Rm3.tif]

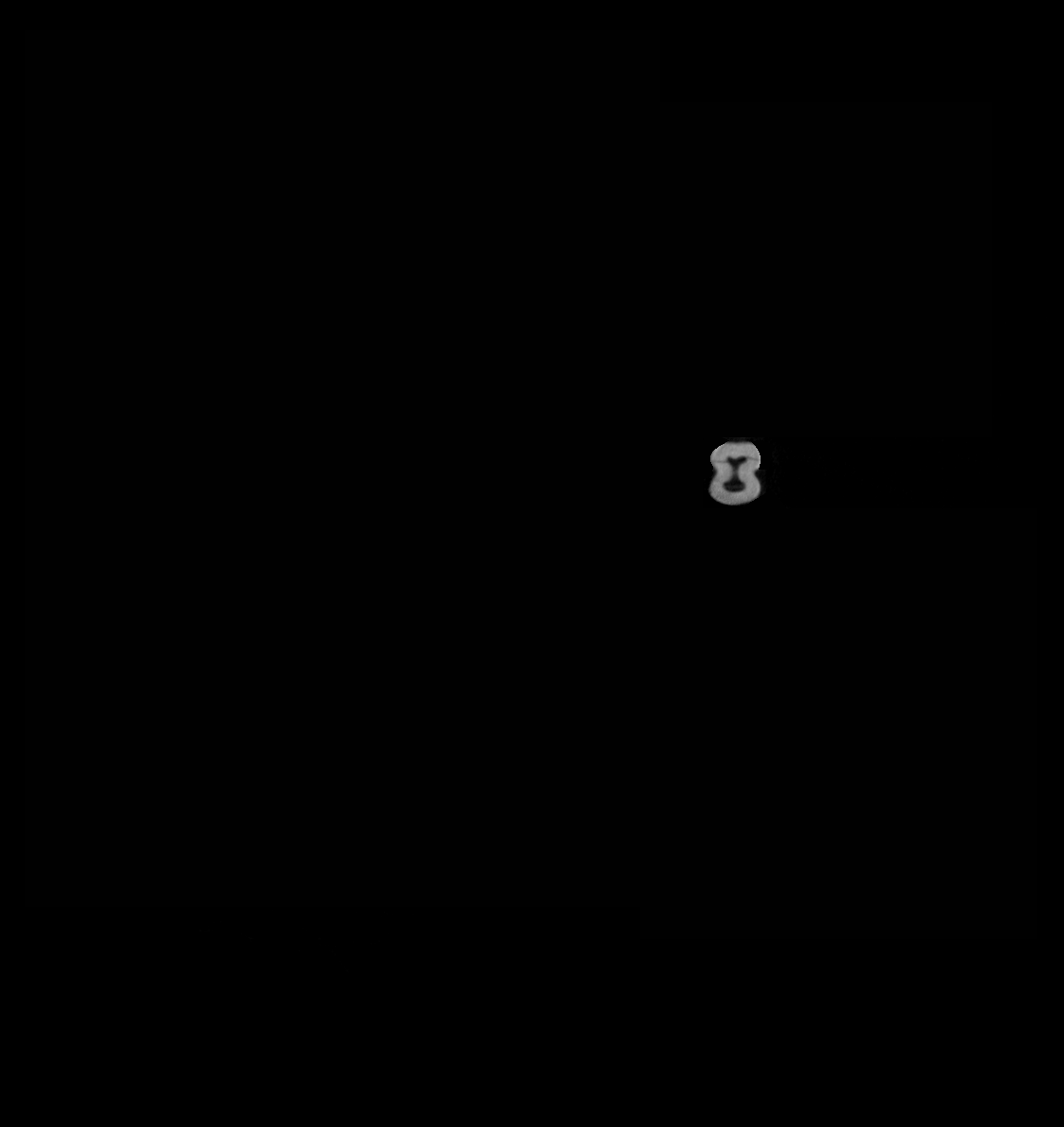

Supplement: Supplementary file 2 — Data S2: Supporting Information. [file AJPA-188-e70164-s001.zip › Cross-Section Tiff Files/mcz_41421_Rm1.tif]

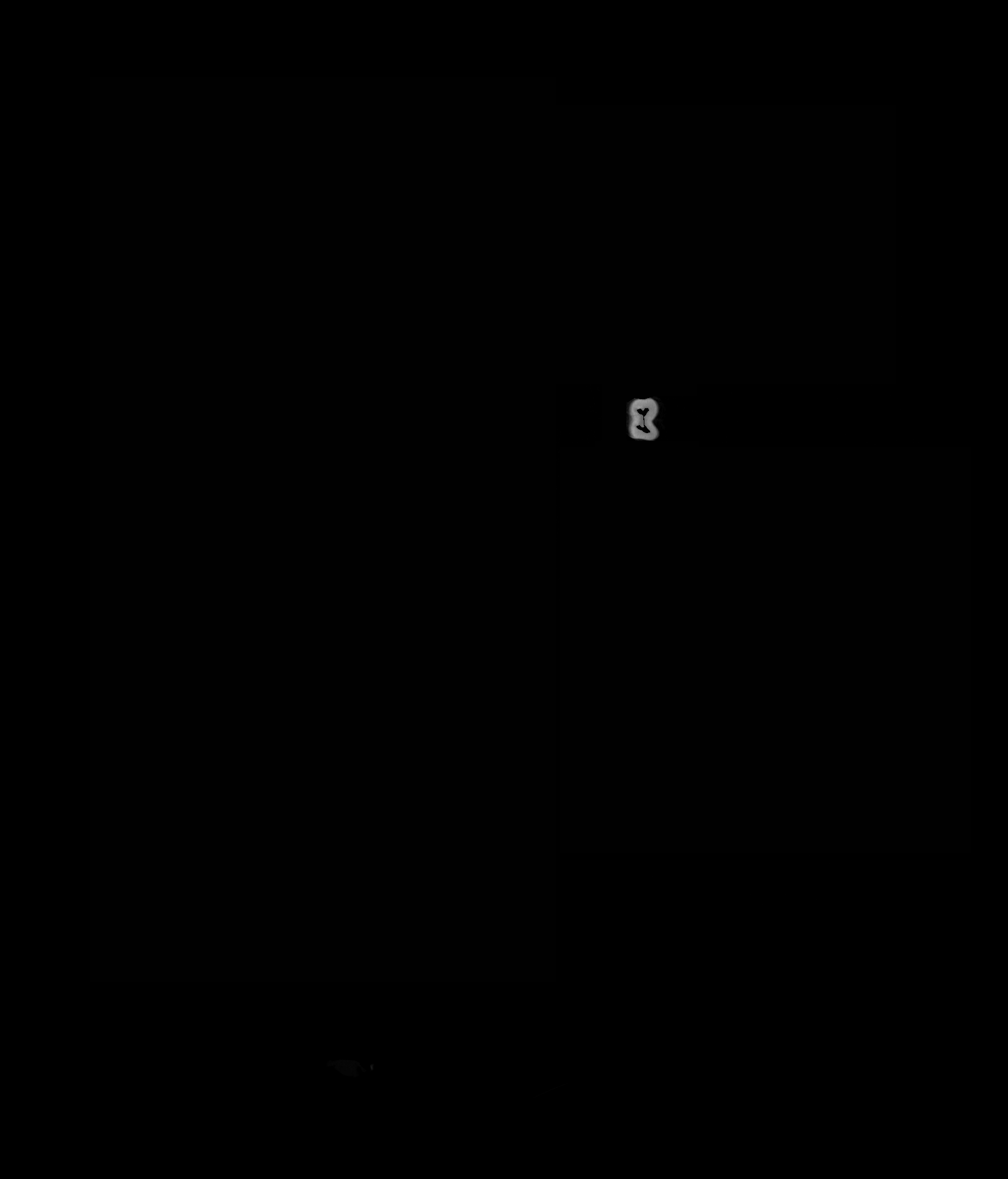

Supplement: Supplementary file 2 — Data S2: Supporting Information. [file AJPA-188-e70164-s001.zip › Cross-Section Tiff Files/mcz_47015_Rm1.tif]

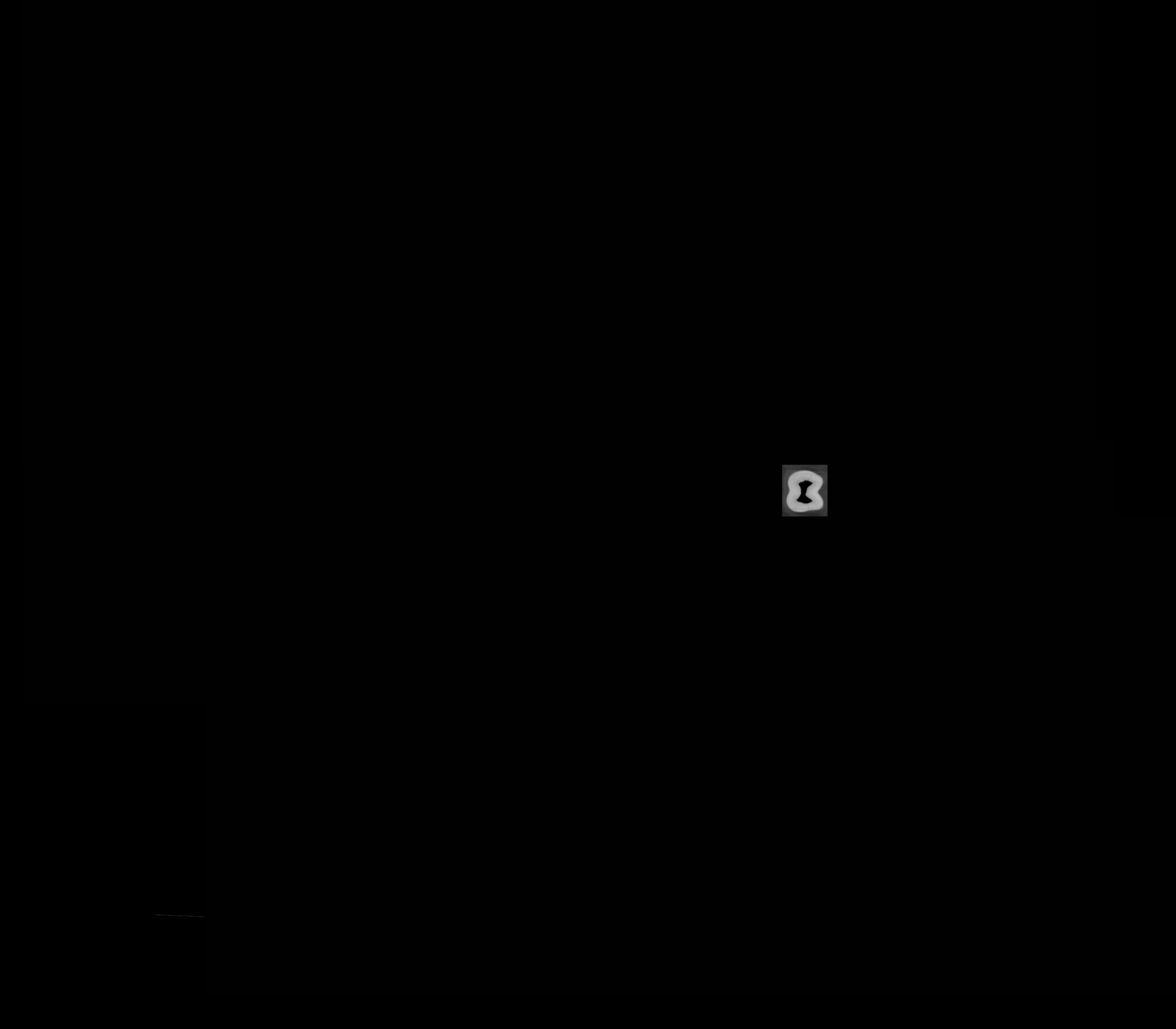

Supplement: Supplementary file 2 — Data S2: Supporting Information. [file AJPA-188-e70164-s001.zip › Cross-Section Tiff Files/amnh_52210_Rm2.tif]

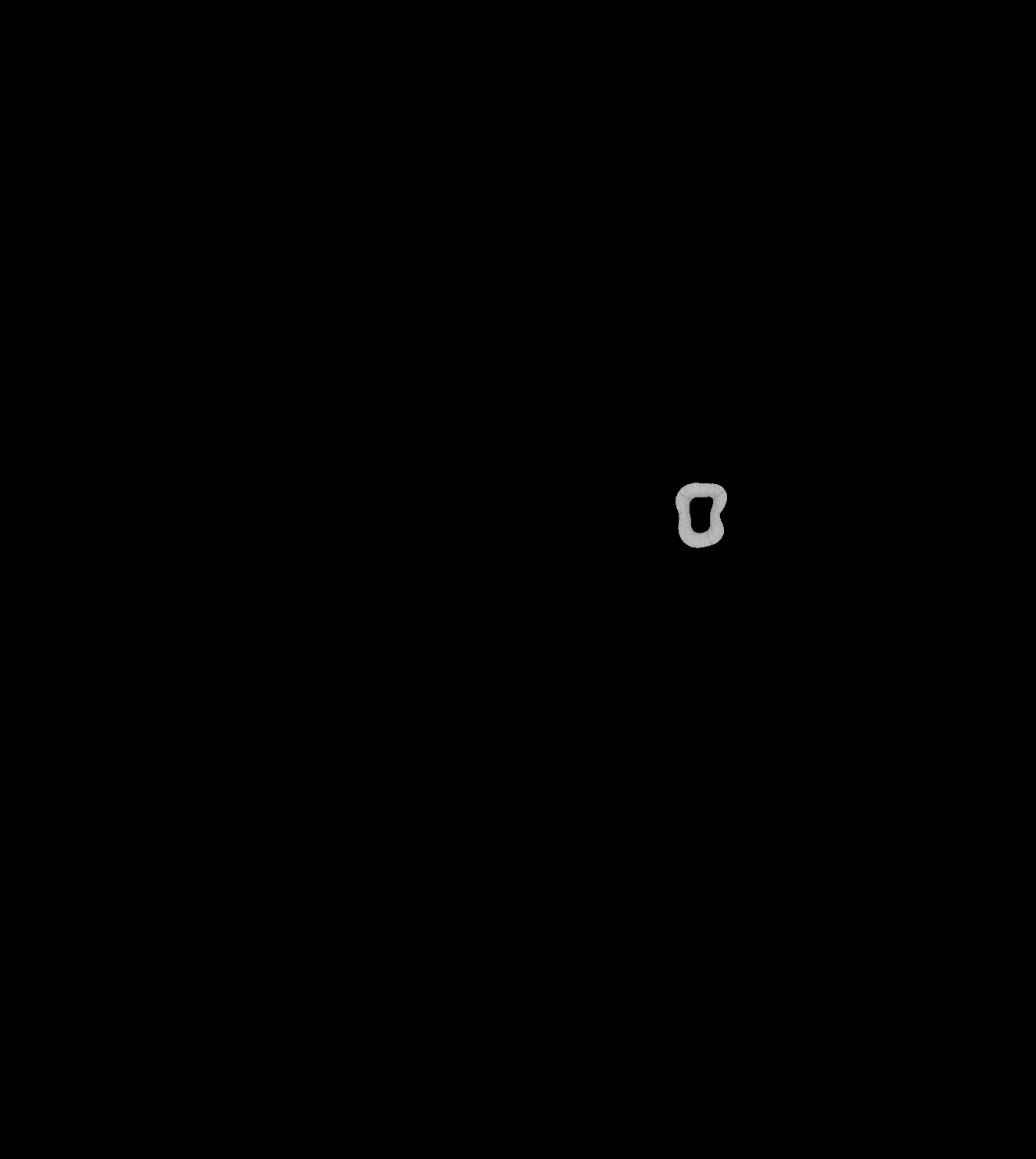

Supplement: Supplementary file 2 — Data S2: Supporting Information. [file AJPA-188-e70164-s001.zip › Cross-Section Tiff Files/mcz_41428_Rm2.tif]

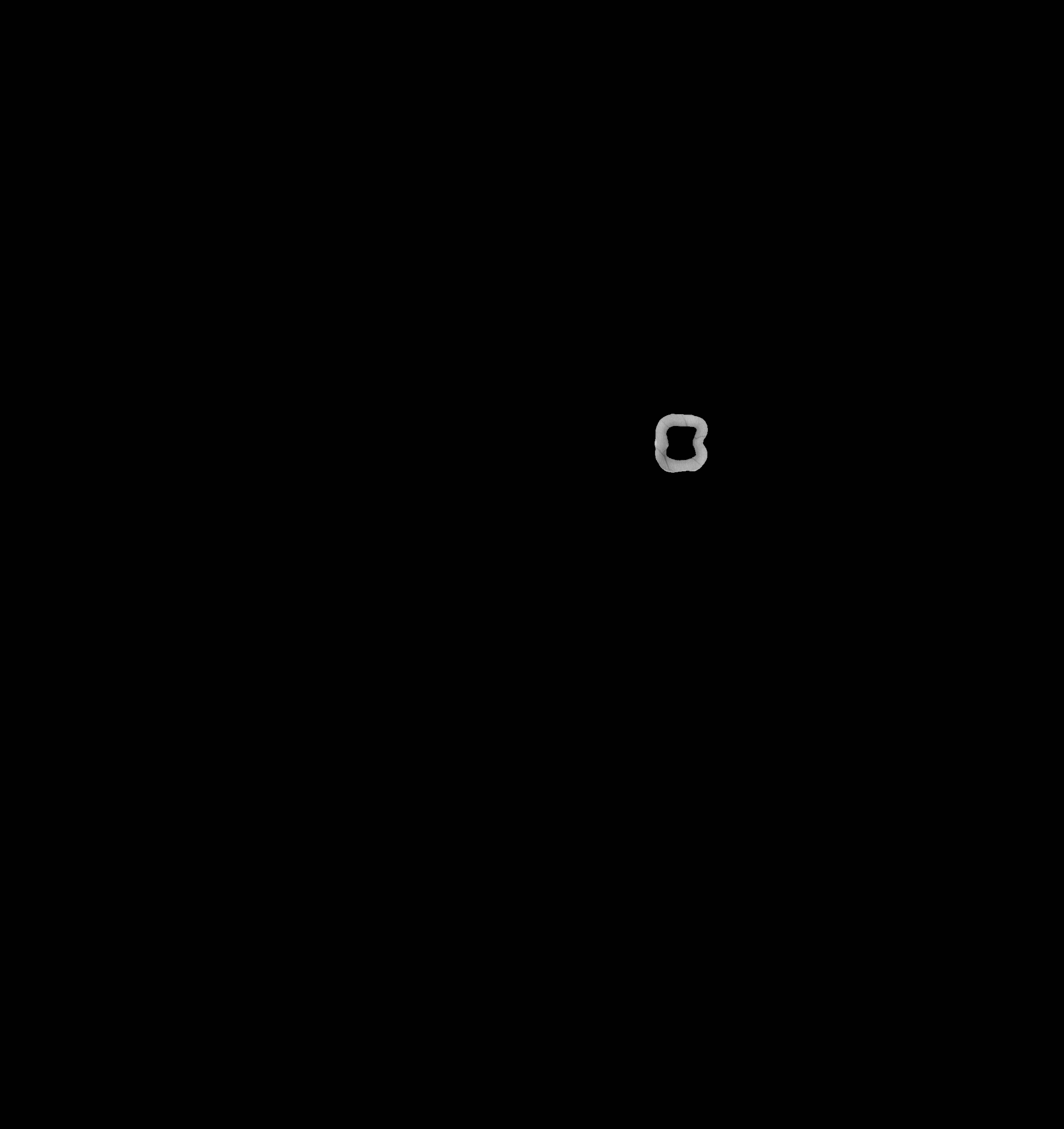

Supplement: Supplementary file 2 — Data S2: Supporting Information. [file AJPA-188-e70164-s001.zip › Cross-Section Tiff Files/mcz_23160_Rm1.tif]

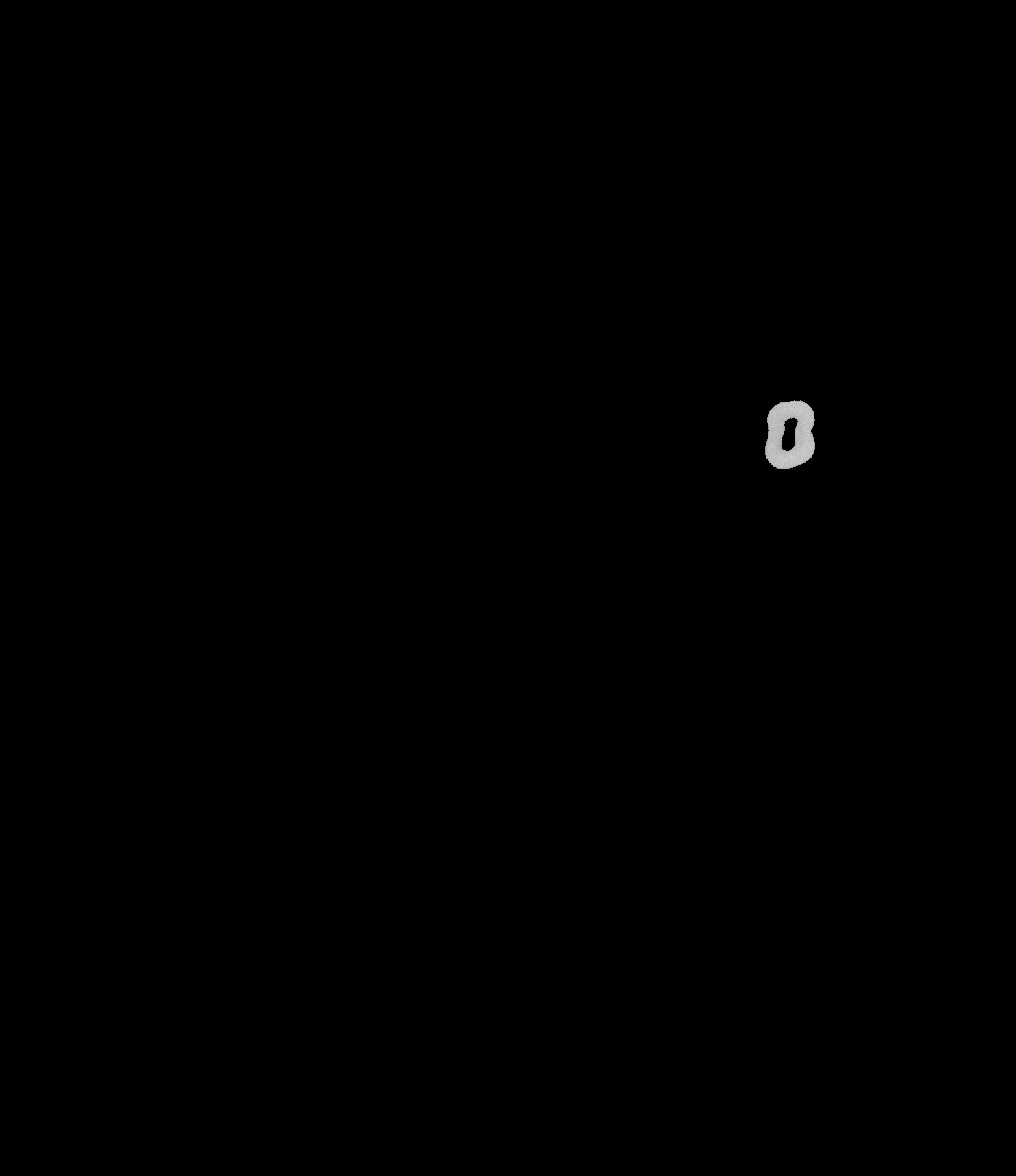

Supplement: Supplementary file 2 — Data S2: Supporting Information. [file AJPA-188-e70164-s001.zip › Cross-Section Tiff Files/mcz_37382_Rm1.tif]

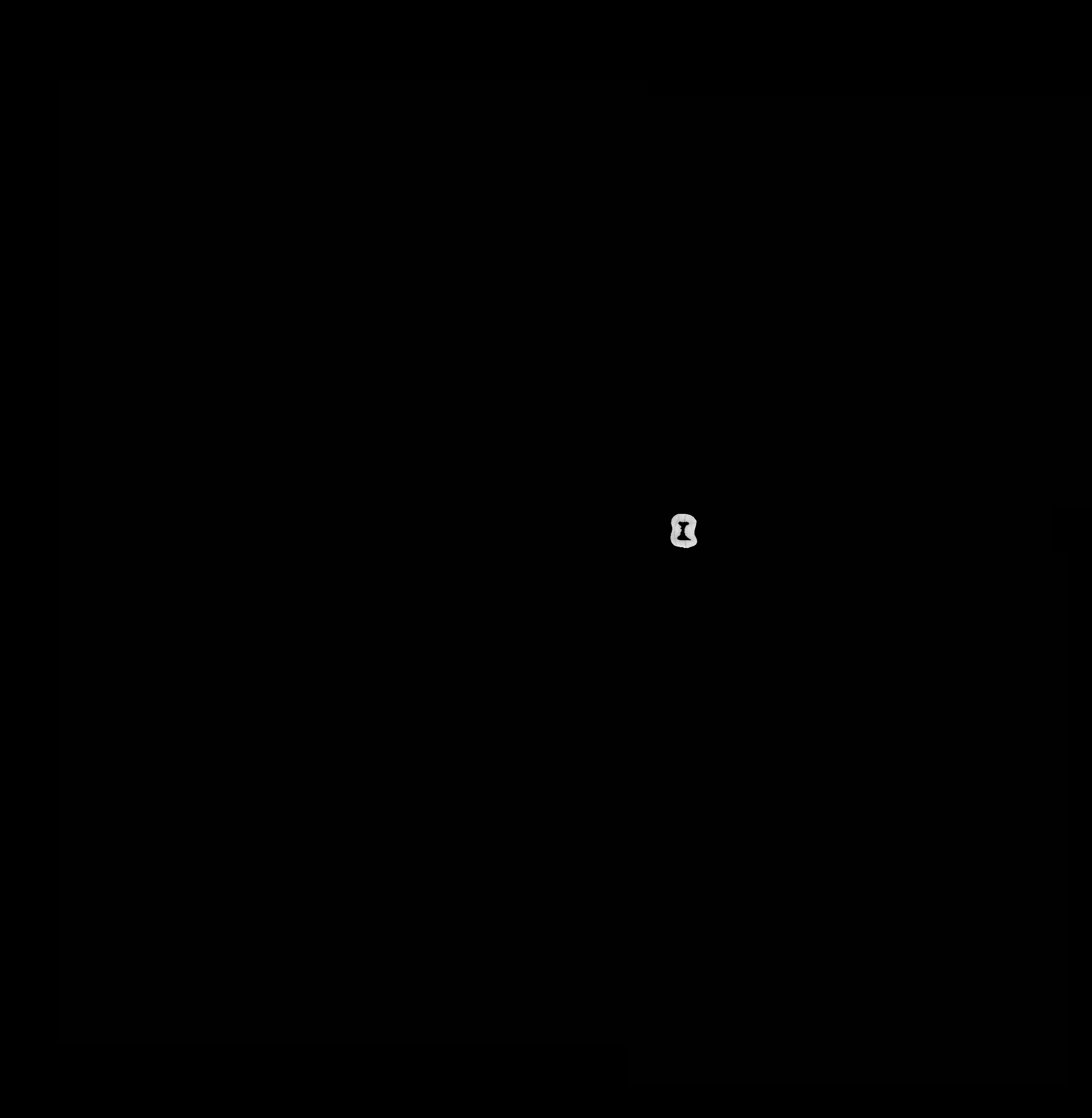

Supplement: Supplementary file 2 — Data S2: Supporting Information. [file AJPA-188-e70164-s001.zip › Cross-Section Tiff Files/amnh_52209_Rm1.tif]

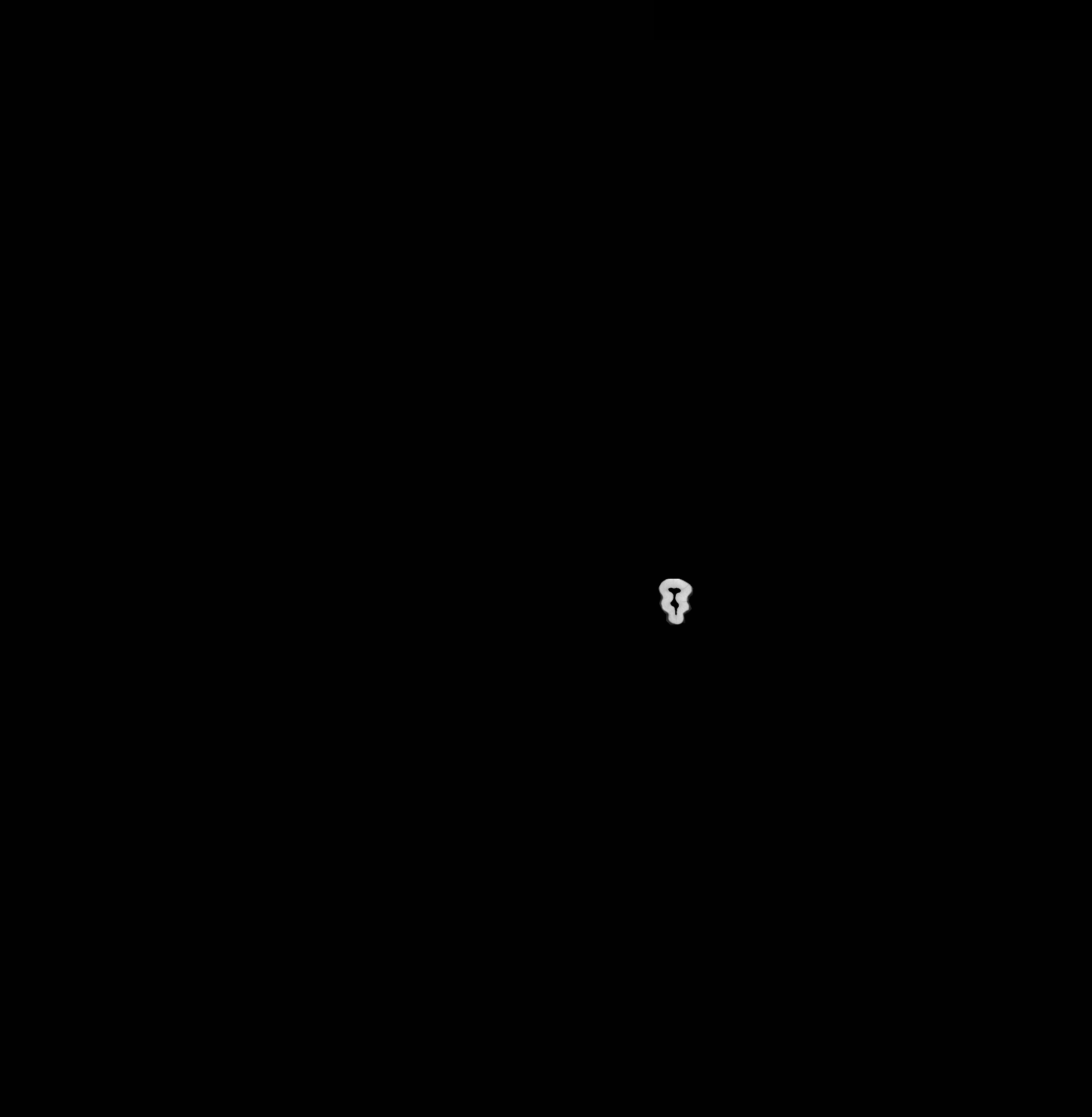

Supplement: Supplementary file 2 — Data S2: Supporting Information. [file AJPA-188-e70164-s001.zip › Cross-Section Tiff Files/amnh_52209_Rm3.tif]

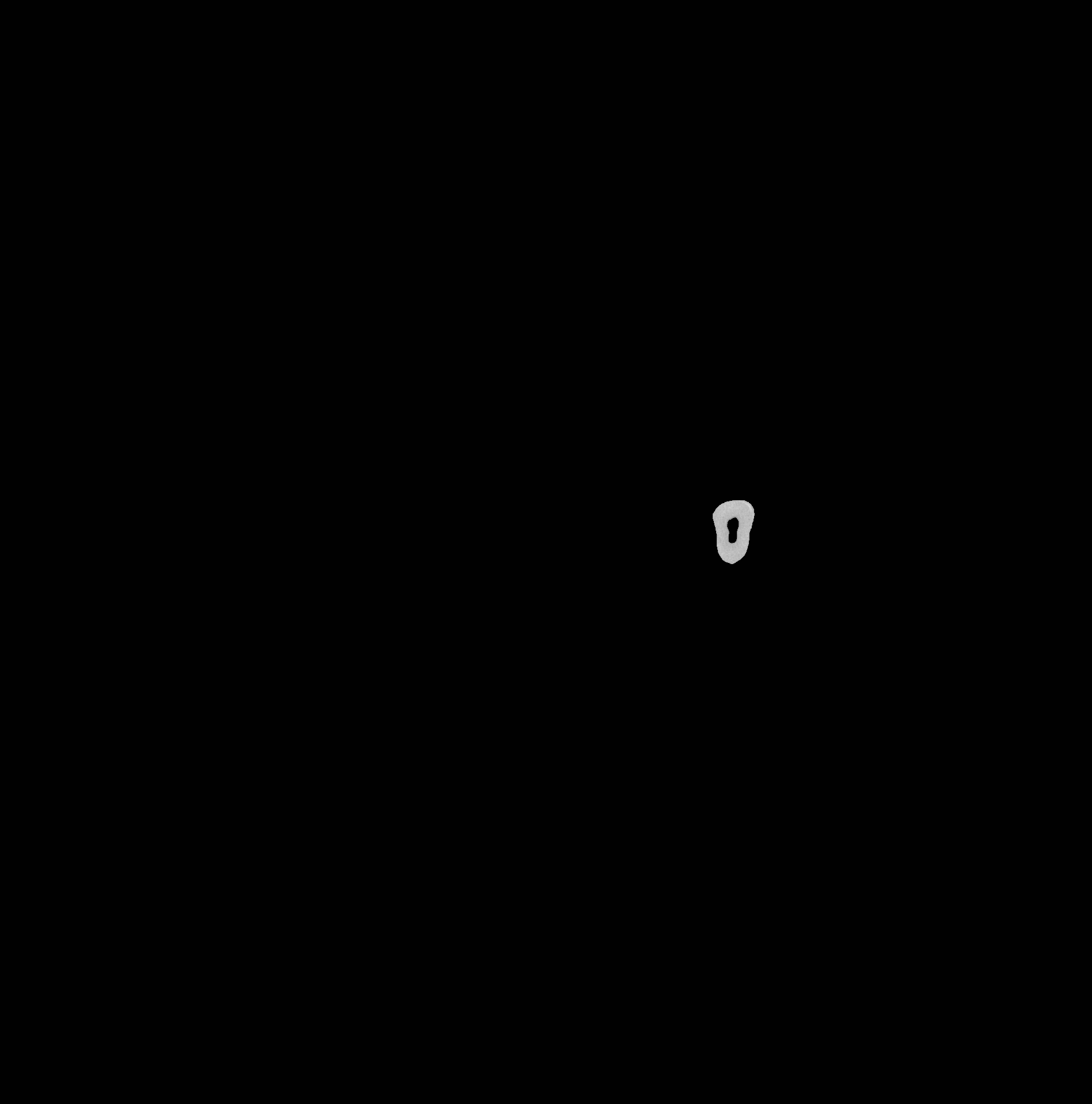

Supplement: Supplementary file 2 — Data S2: Supporting Information. [file AJPA-188-e70164-s001.zip › Cross-Section Tiff Files/mcz_37382_Rm3.tif]

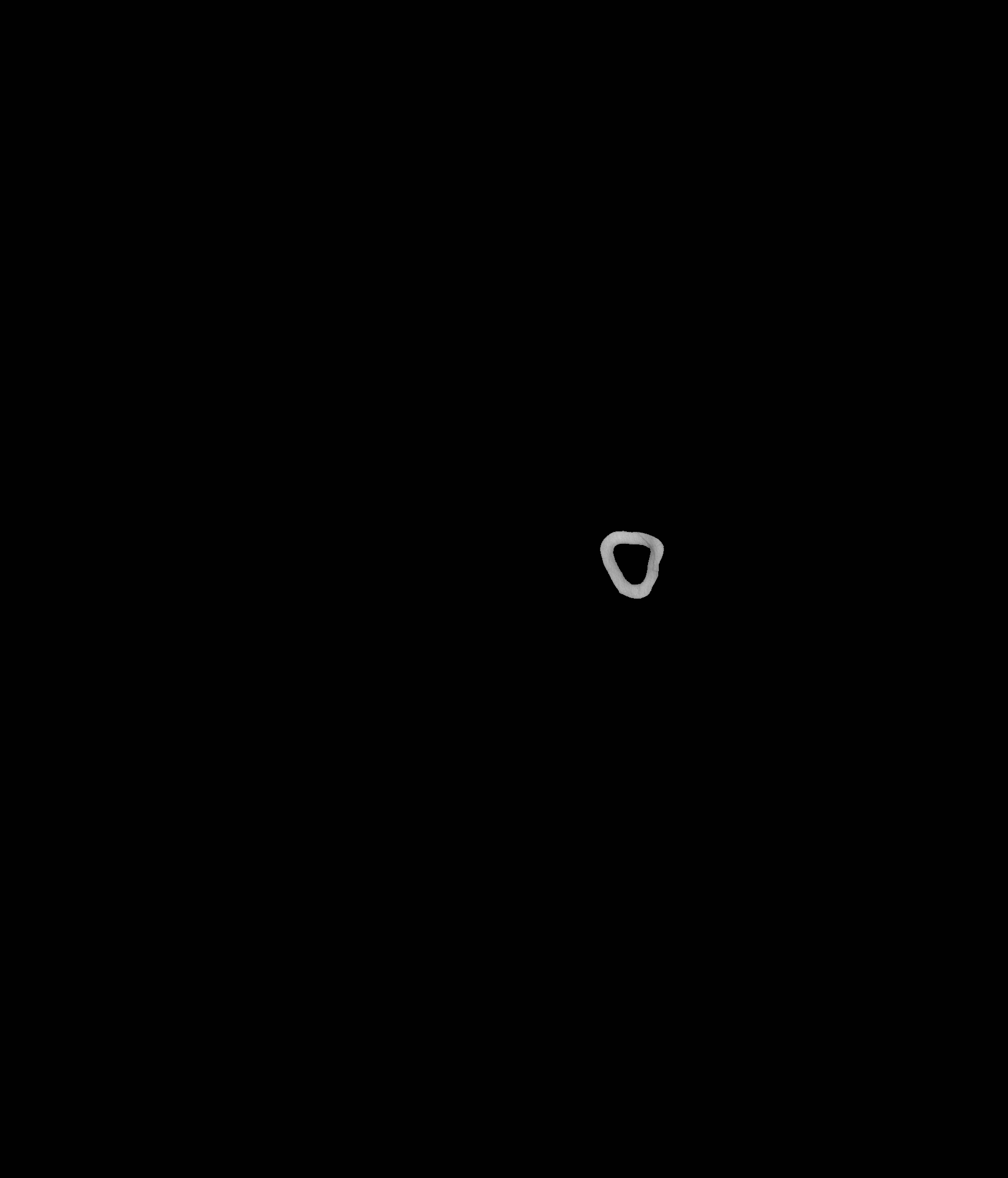

Supplement: Supplementary file 2 — Data S2: Supporting Information. [file AJPA-188-e70164-s001.zip › Cross-Section Tiff Files/mcz_23160_Rm3.tif]

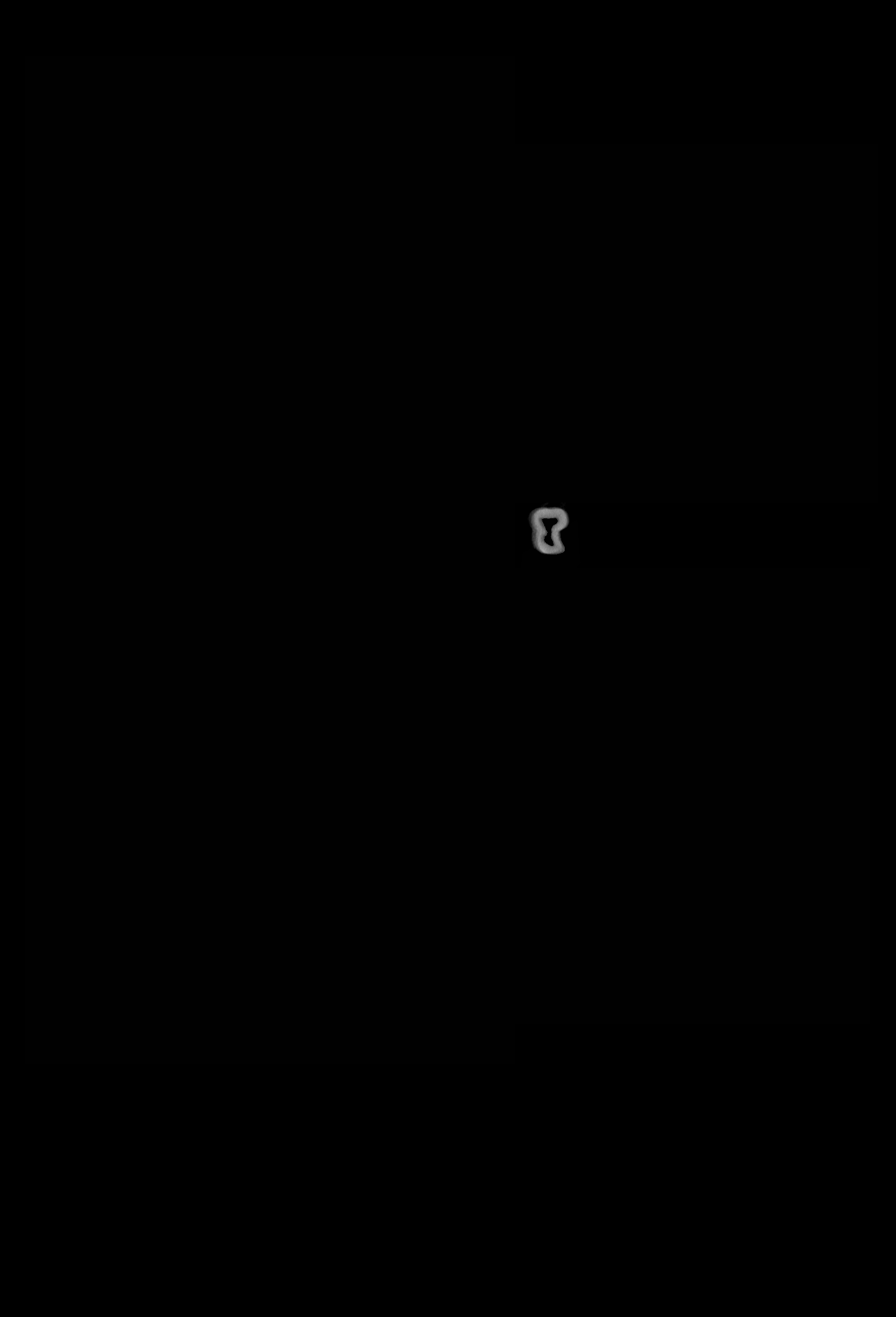

Supplement: Supplementary file 2 — Data S2: Supporting Information. [file AJPA-188-e70164-s001.zip › Cross-Section Tiff Files/mcz_47015_Rm3.tif]

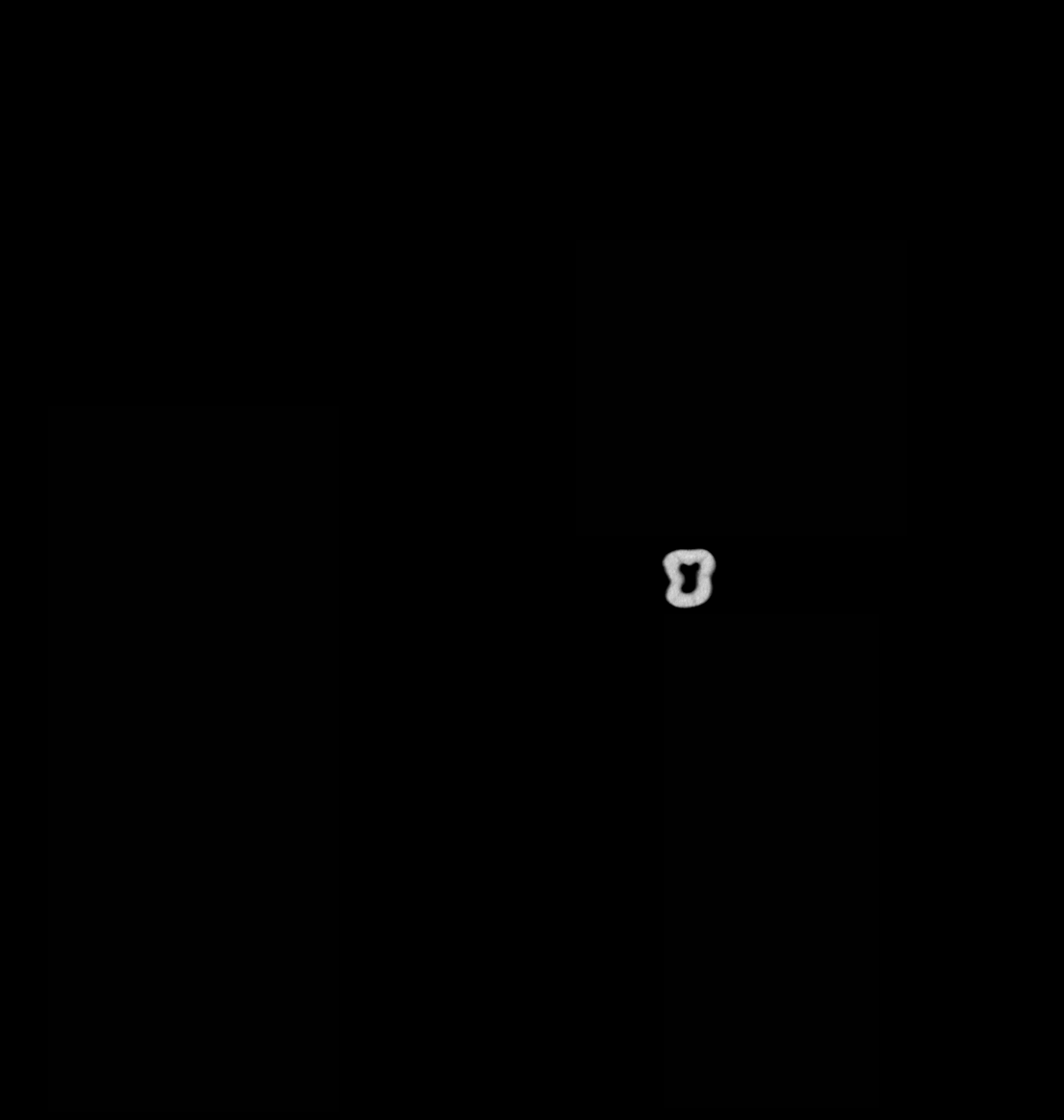

Supplement: Supplementary file 2 — Data S2: Supporting Information. [file AJPA-188-e70164-s001.zip › Cross-Section Tiff Files/mcz_41421_Rm3.tif]

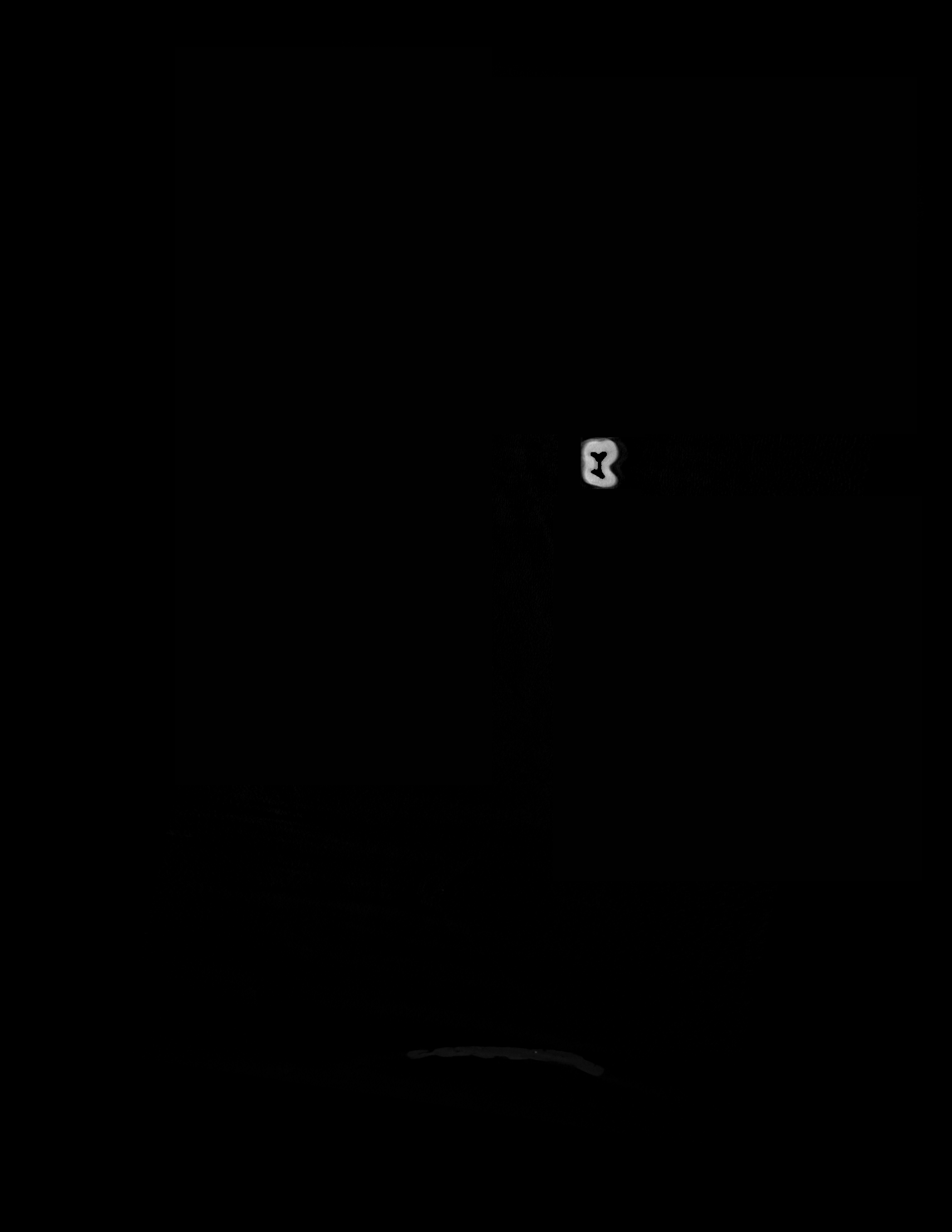

Supplement: Supplementary file 2 — Data S2: Supporting Information. [file AJPA-188-e70164-s001.zip › Cross-Section Tiff Files/mcz_47015_Rm2.tif]

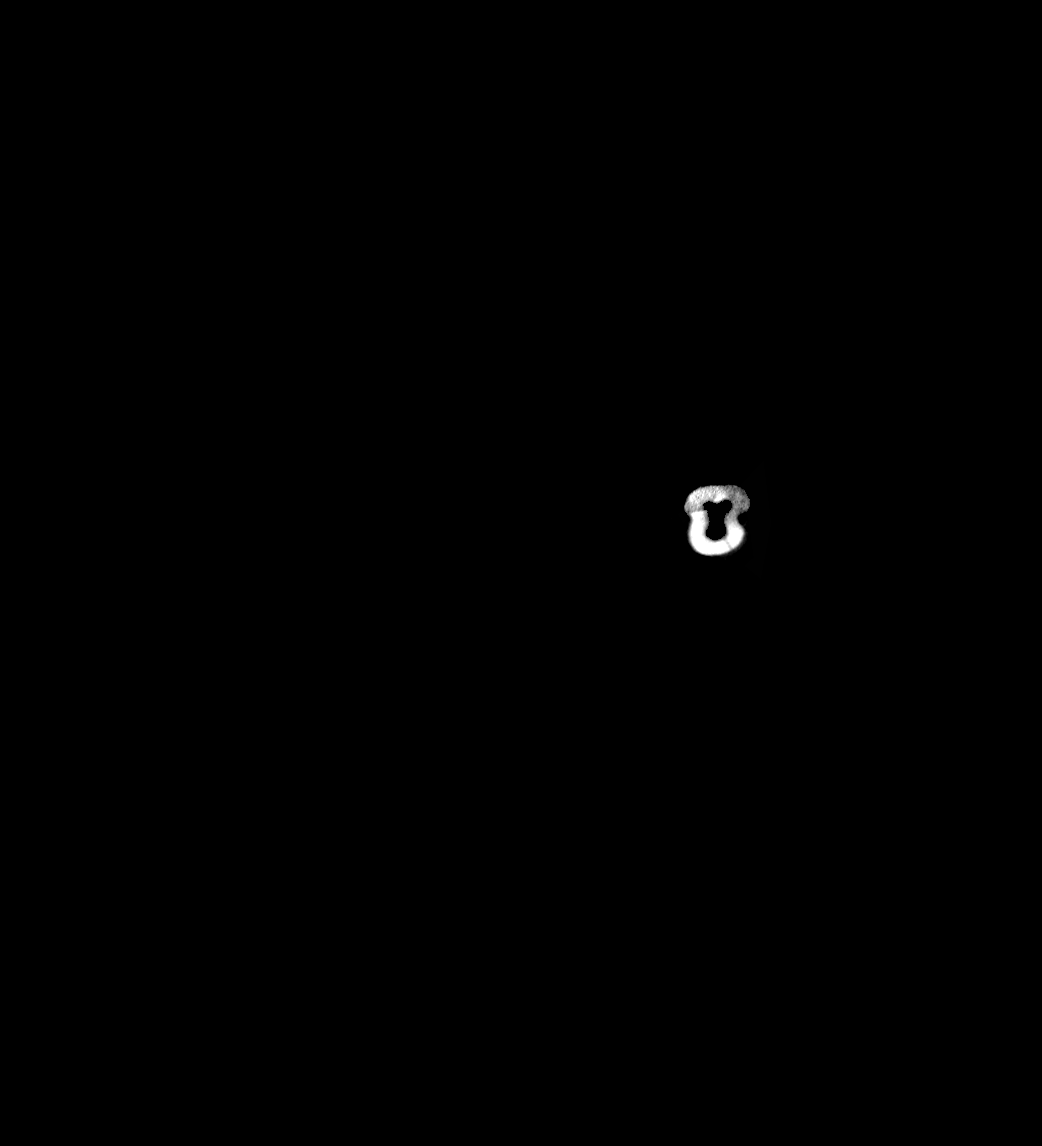

Supplement: Supplementary file 2 — Data S2: Supporting Information. [file AJPA-188-e70164-s001.zip › Cross-Section Tiff Files/mcz_41421_Rm2.tif]

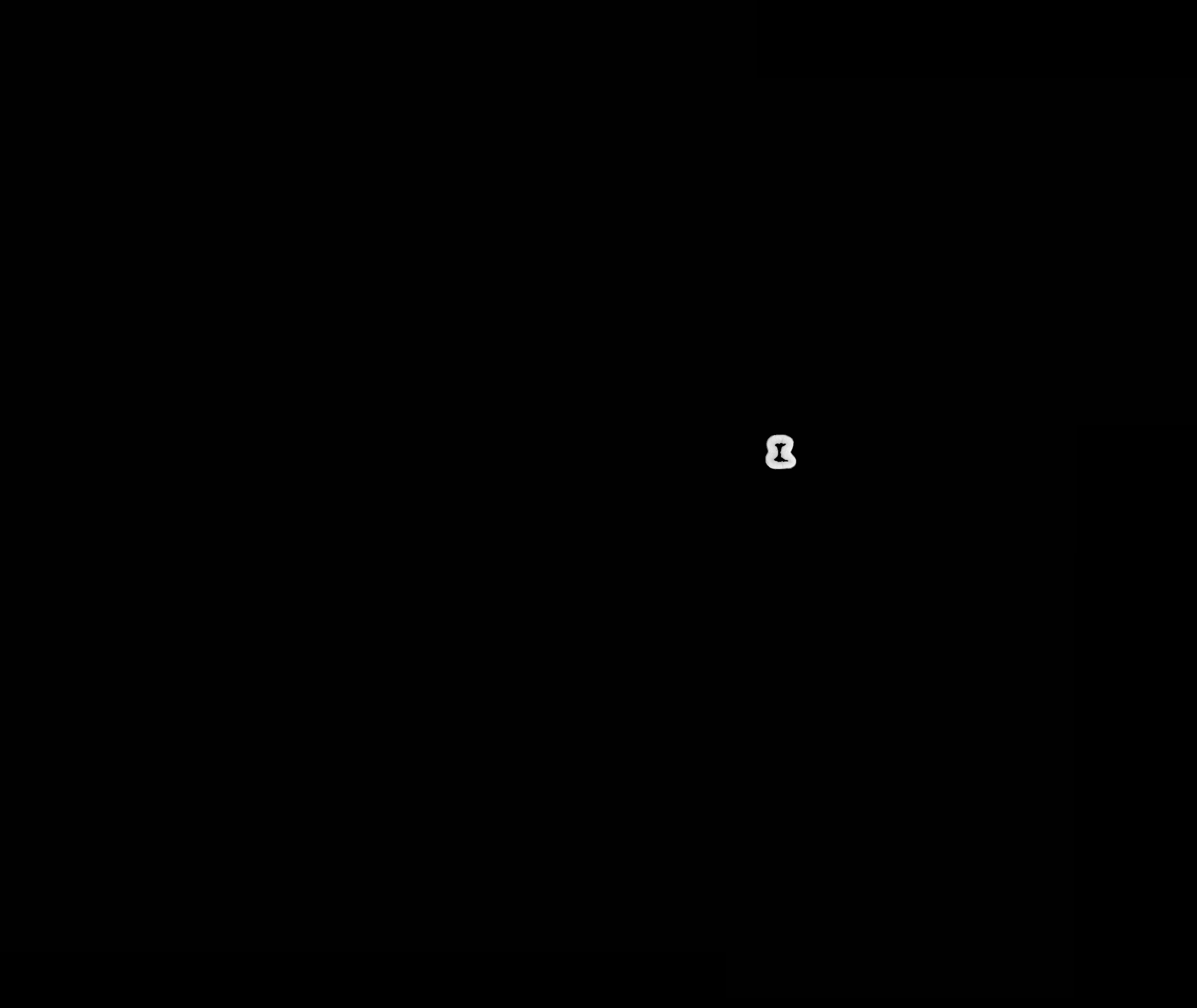

Supplement: Supplementary file 2 — Data S2: Supporting Information. [file AJPA-188-e70164-s001.zip › Cross-Section Tiff Files/amnh_52210_Rm1.tif]

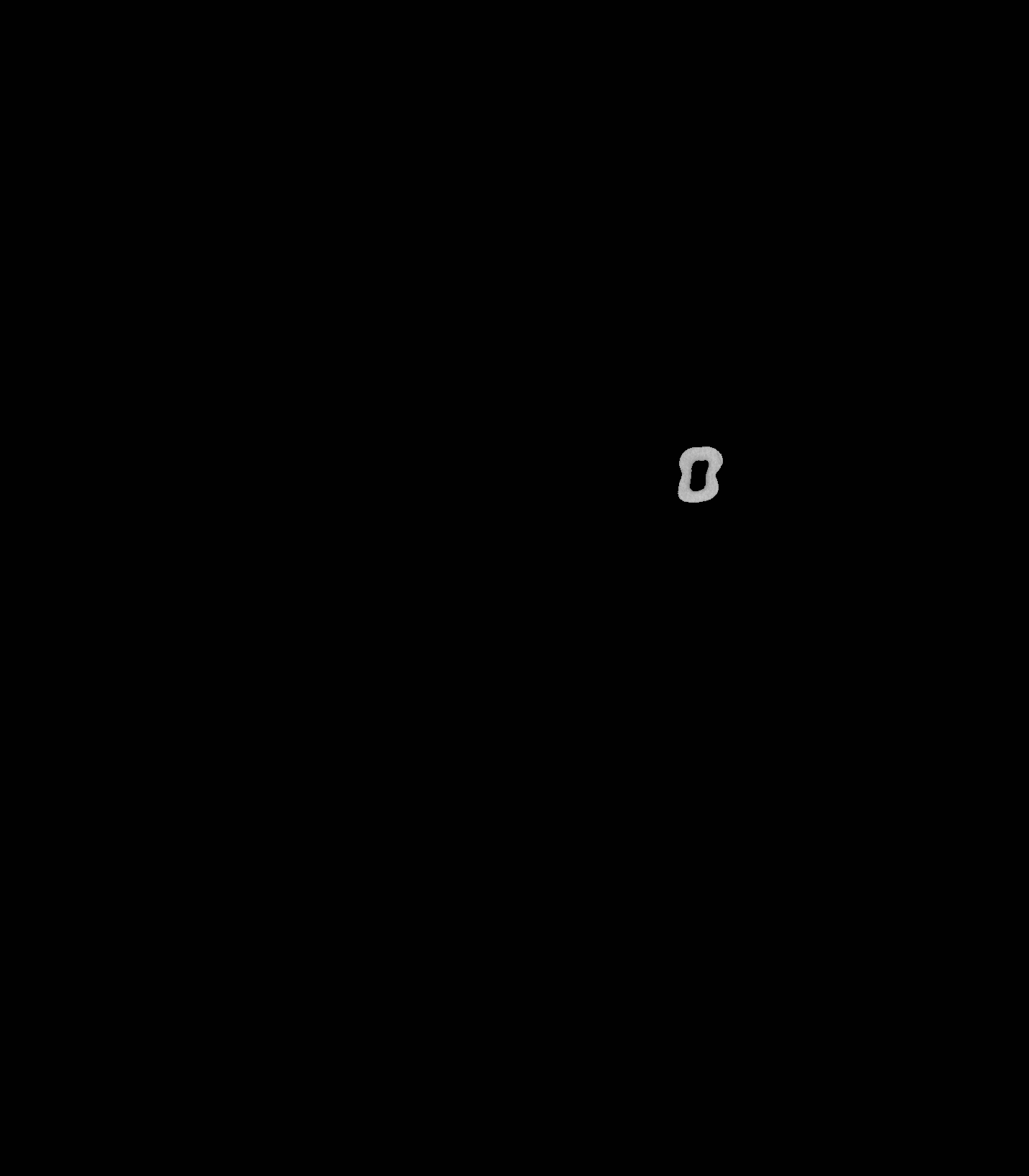

Supplement: Supplementary file 2 — Data S2: Supporting Information. [file AJPA-188-e70164-s001.zip › Cross-Section Tiff Files/mcz_41428_Rm1.tif]

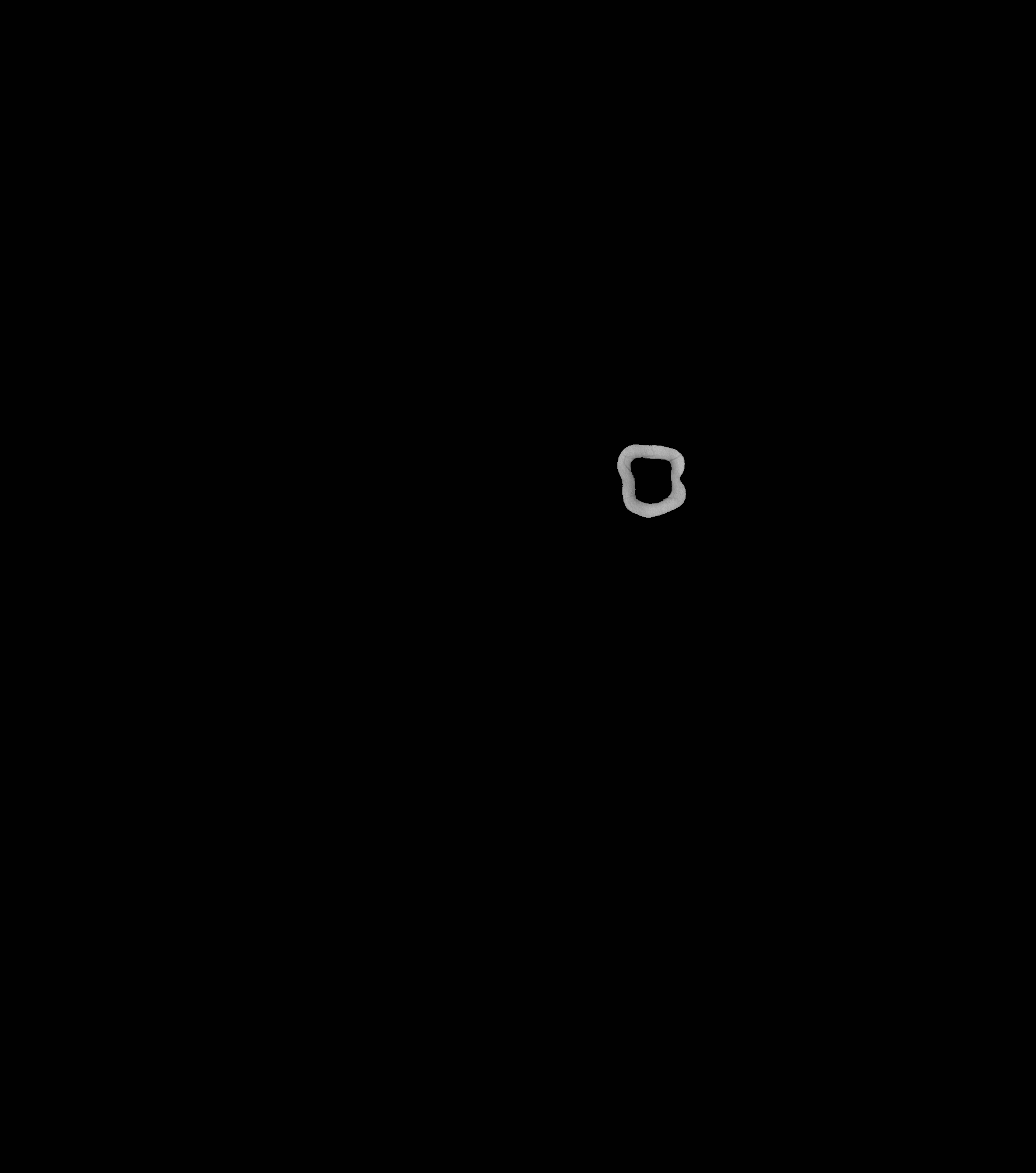

Supplement: Supplementary file 2 — Data S2: Supporting Information. [file AJPA-188-e70164-s001.zip › Cross-Section Tiff Files/mcz_23160_Rm2.tif]

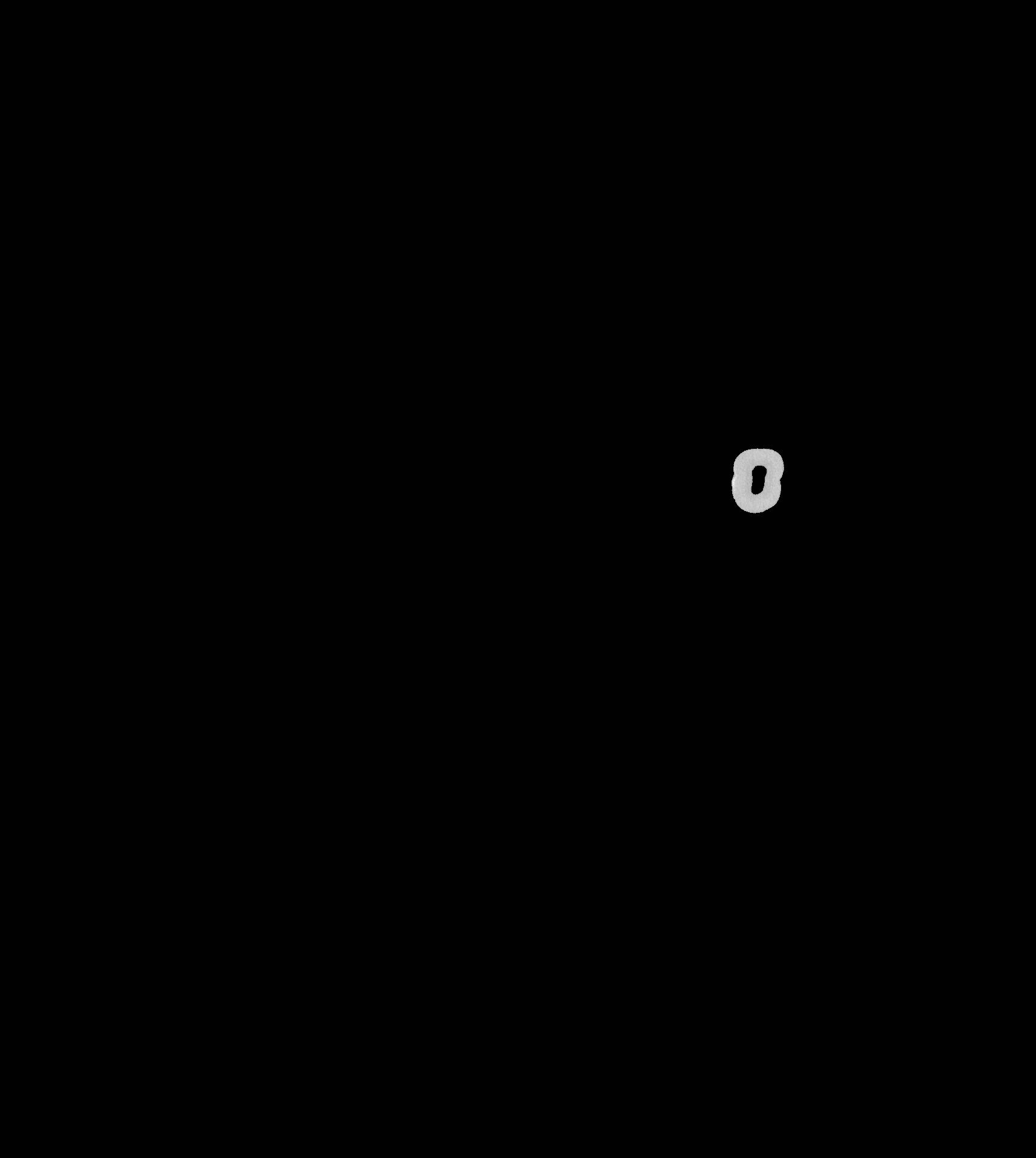

Supplement: Supplementary file 2 — Data S2: Supporting Information. [file AJPA-188-e70164-s001.zip › Cross-Section Tiff Files/mcz_37382_Rm2.tif]

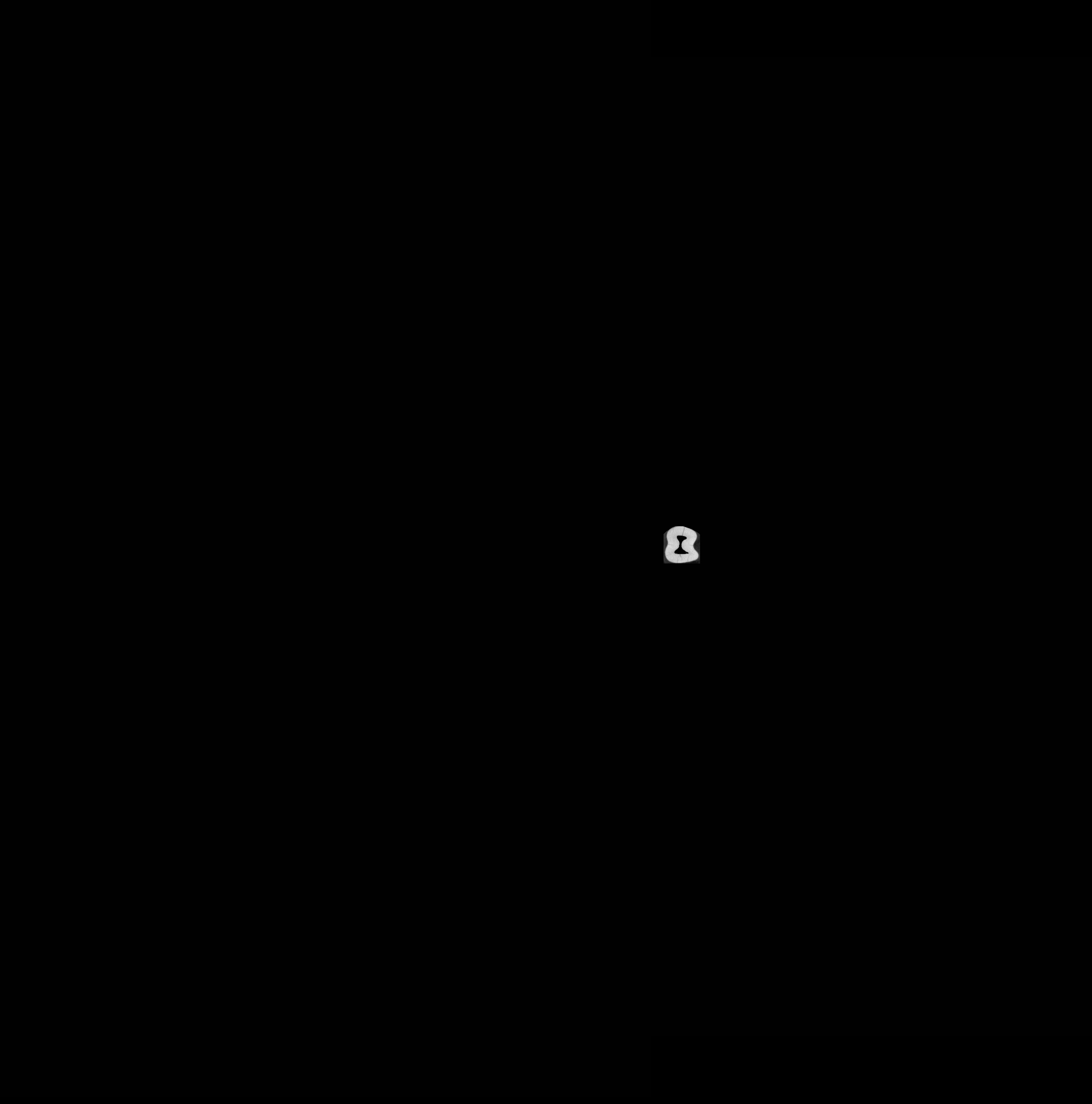

Supplement: Supplementary file 2 — Data S2: Supporting Information. [file AJPA-188-e70164-s001.zip › Cross-Section Tiff Files/amnh_52209_Rm2.tif]

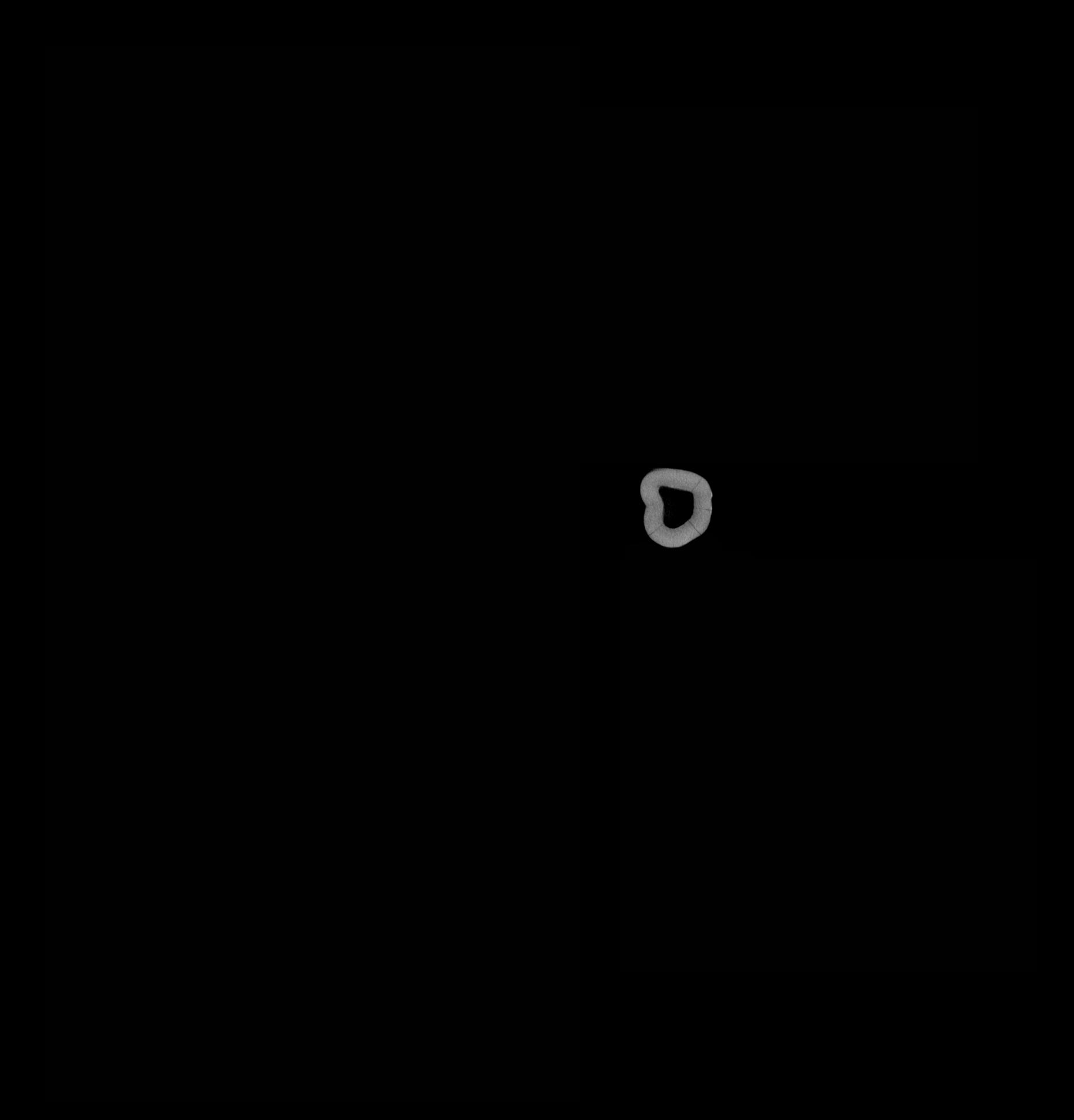

Supplement: Supplementary file 2 — Data S2: Supporting Information. [file AJPA-188-e70164-s001.zip › Cross-Section Tiff Files/mcz_50958_Rm2.tif]

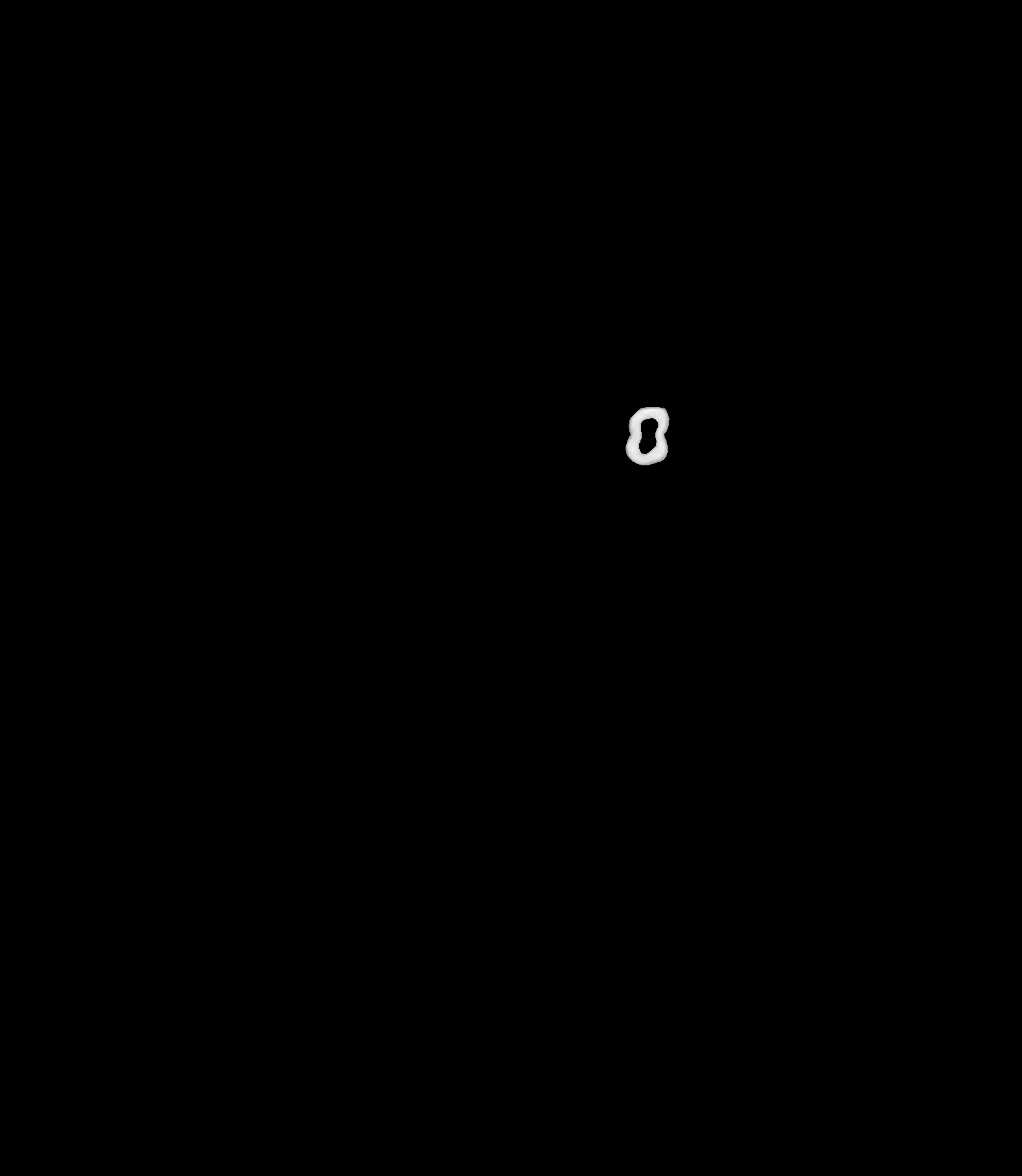

Supplement: Supplementary file 2 — Data S2: Supporting Information. [file AJPA-188-e70164-s001.zip › Cross-Section Tiff Files/mcz_36032_Rm2.tif]

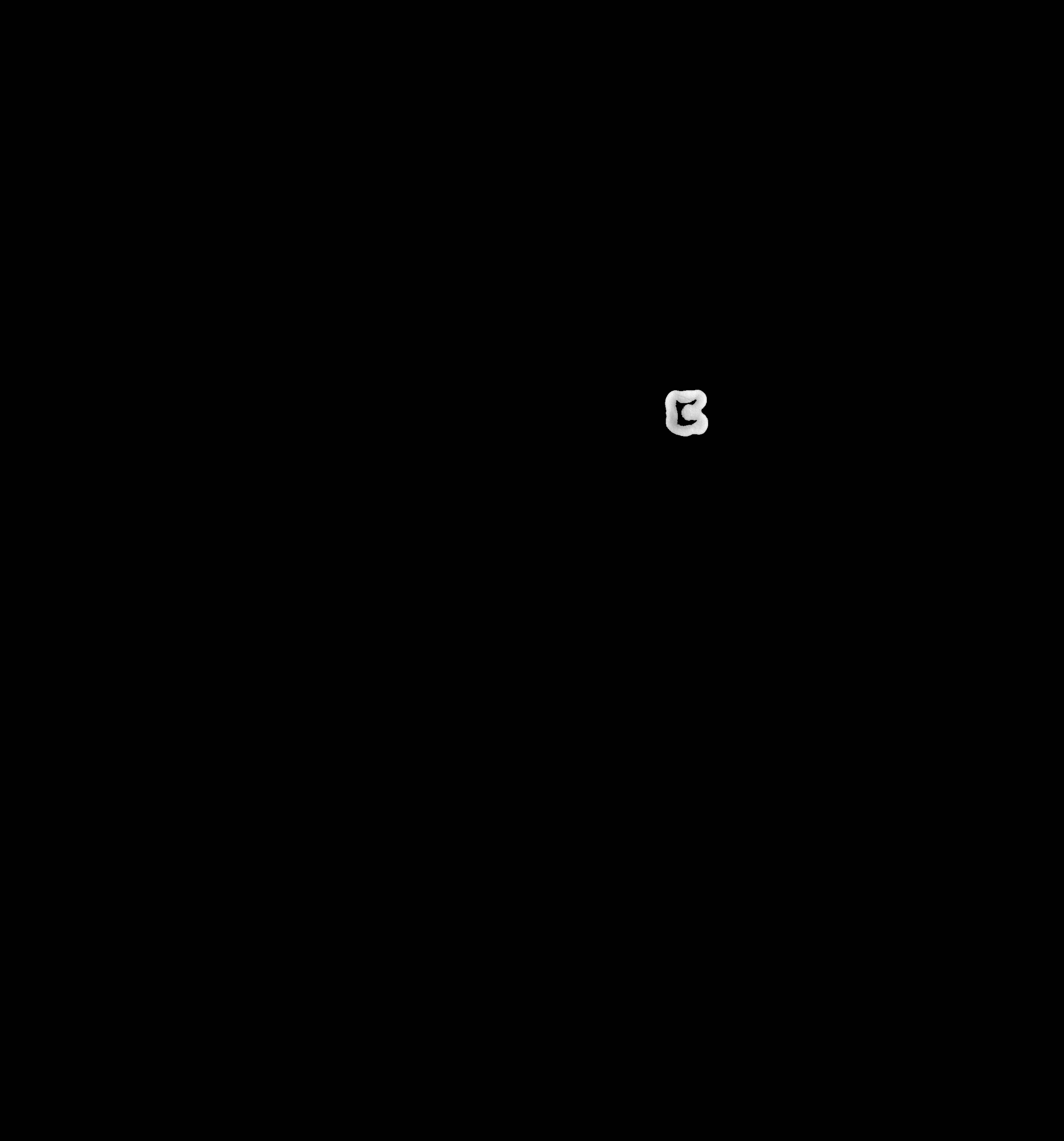

Supplement: Supplementary file 2 — Data S2: Supporting Information. [file AJPA-188-e70164-s001.zip › Cross-Section Tiff Files/mcz_20039_Rm1.tif]

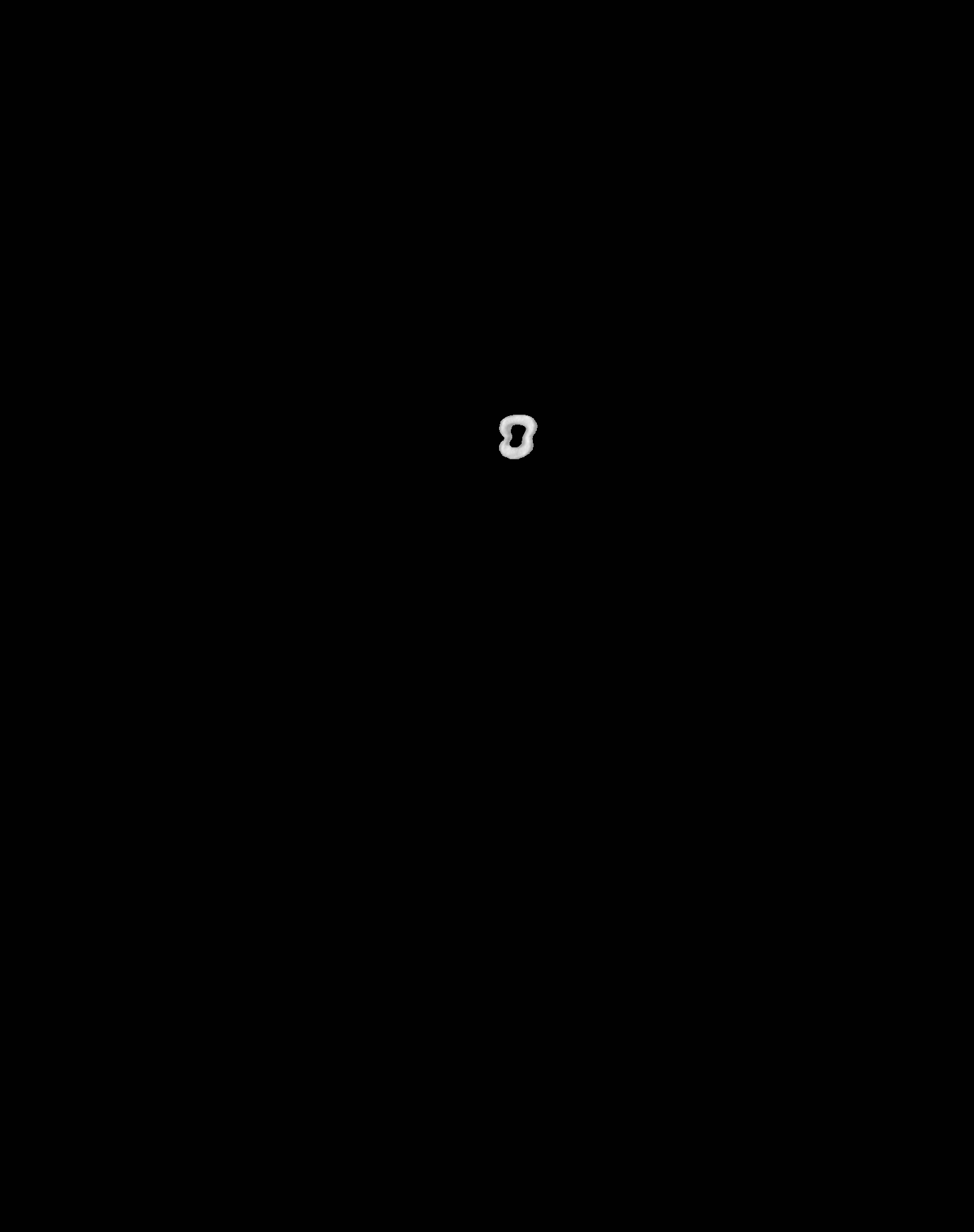

Supplement: Supplementary file 2 — Data S2: Supporting Information. [file AJPA-188-e70164-s001.zip › Cross-Section Tiff Files/mcz_41493_Rm3.tif]

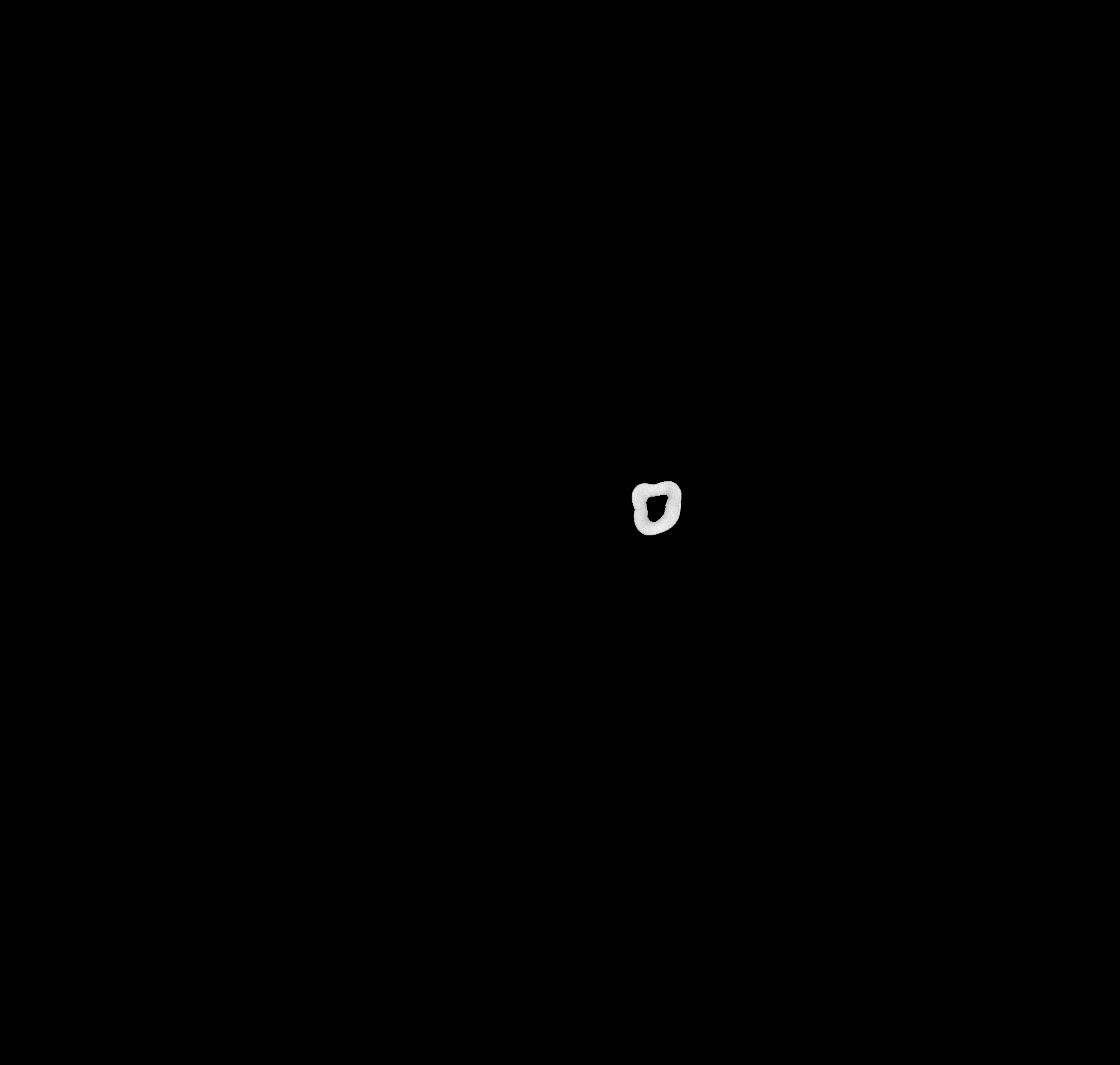

Supplement: Supplementary file 2 — Data S2: Supporting Information. [file AJPA-188-e70164-s001.zip › Cross-Section Tiff Files/mcz_37517_Rm3.tif]

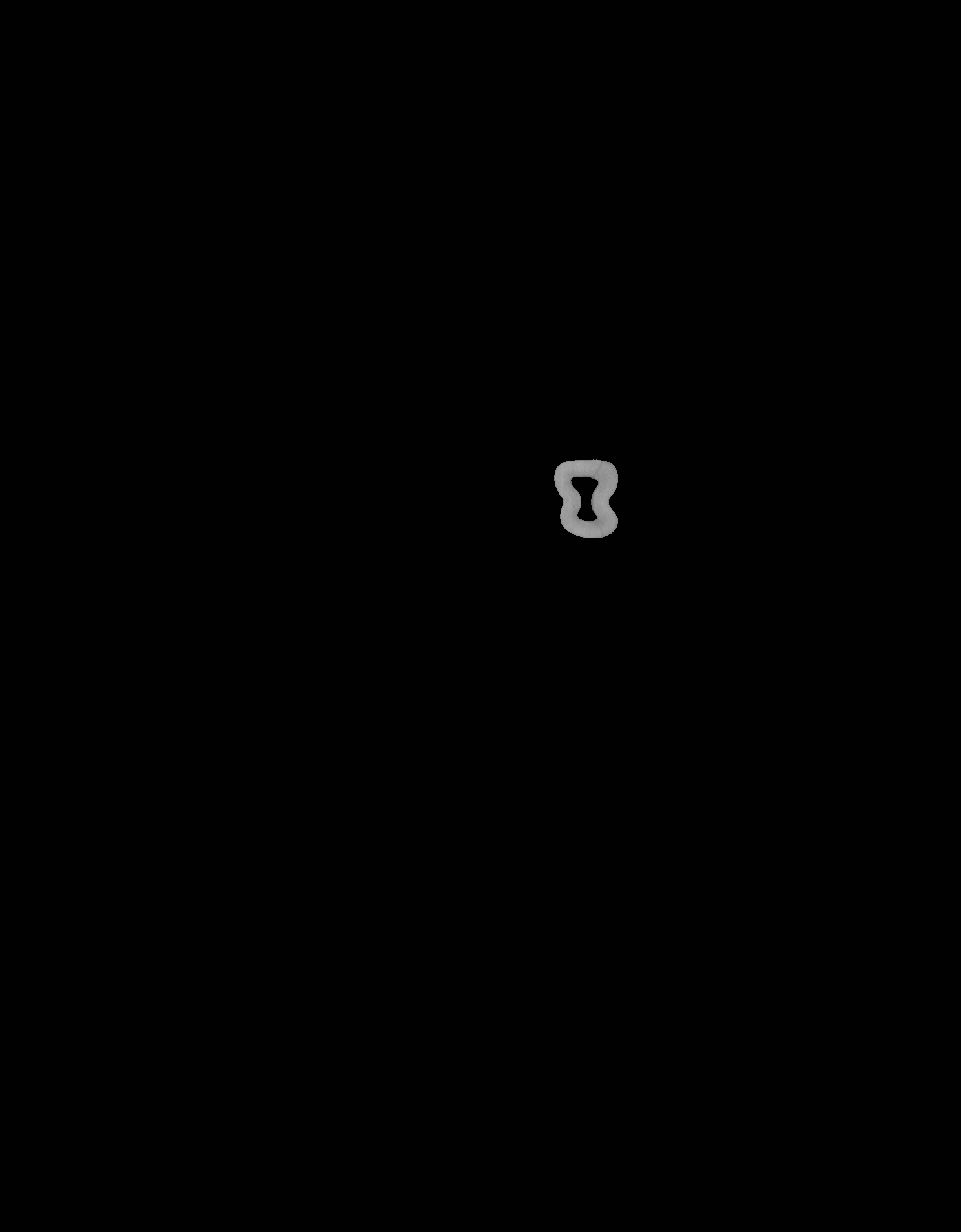

Supplement: Supplementary file 2 — Data S2: Supporting Information. [file AJPA-188-e70164-s001.zip › Cross-Section Tiff Files/mcz_21161_Rm2.tif]

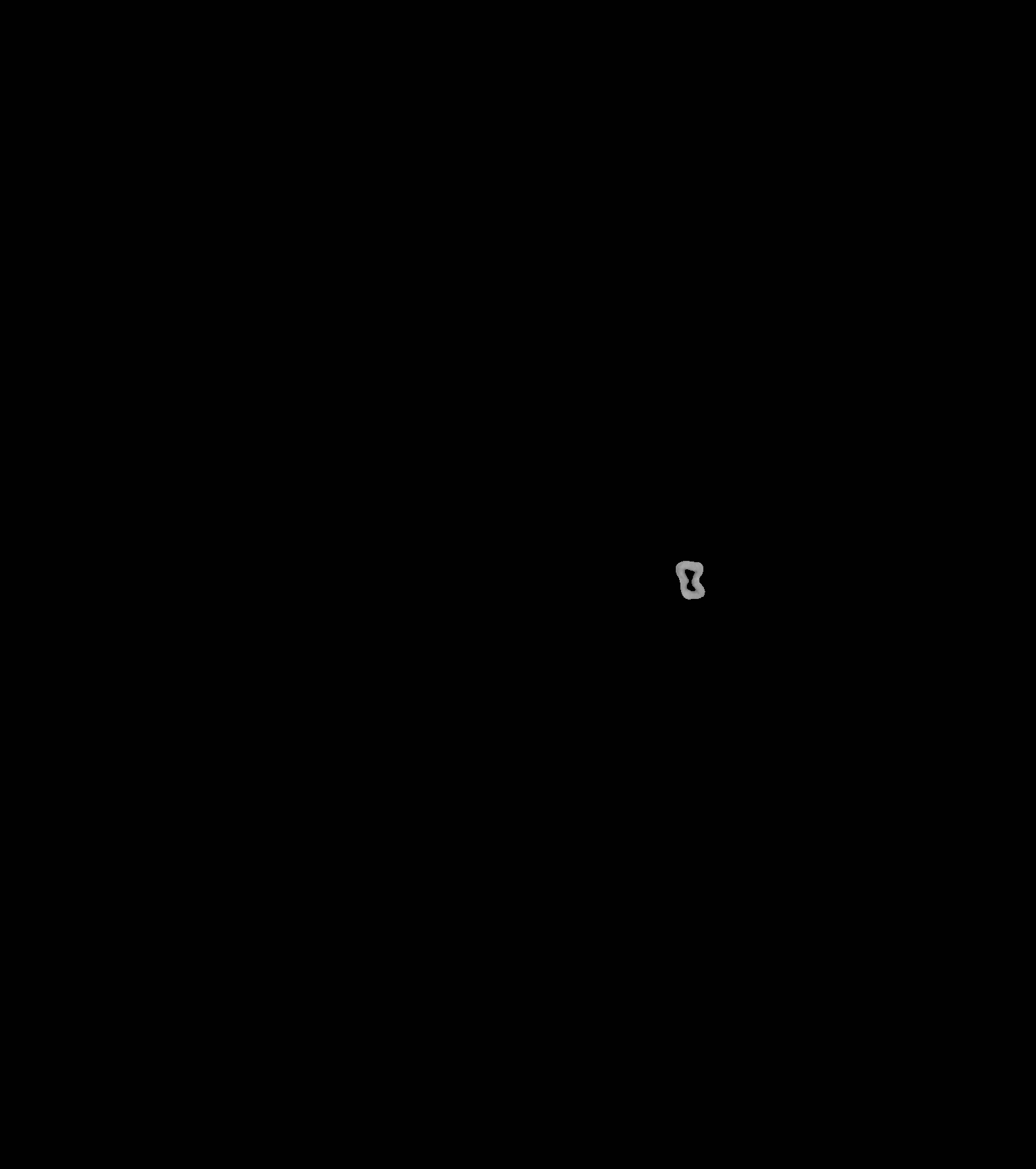

Supplement: Supplementary file 2 — Data S2: Supporting Information. [file AJPA-188-e70164-s001.zip › Cross-Section Tiff Files/mcz_23196_Rm1.tif]

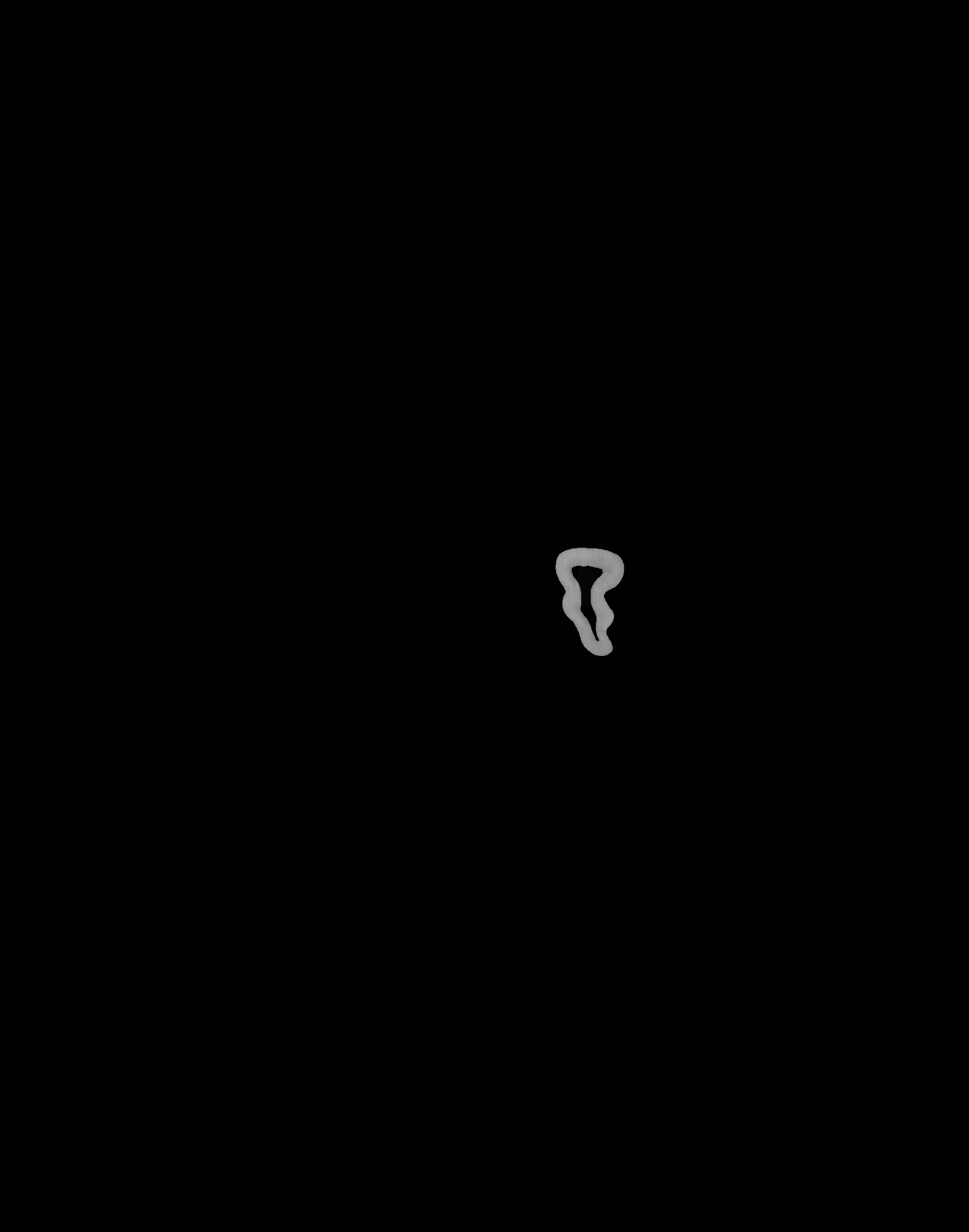

Supplement: Supplementary file 2 — Data S2: Supporting Information. [file AJPA-188-e70164-s001.zip › Cross-Section Tiff Files/mcz_21161_Rm3.tif]

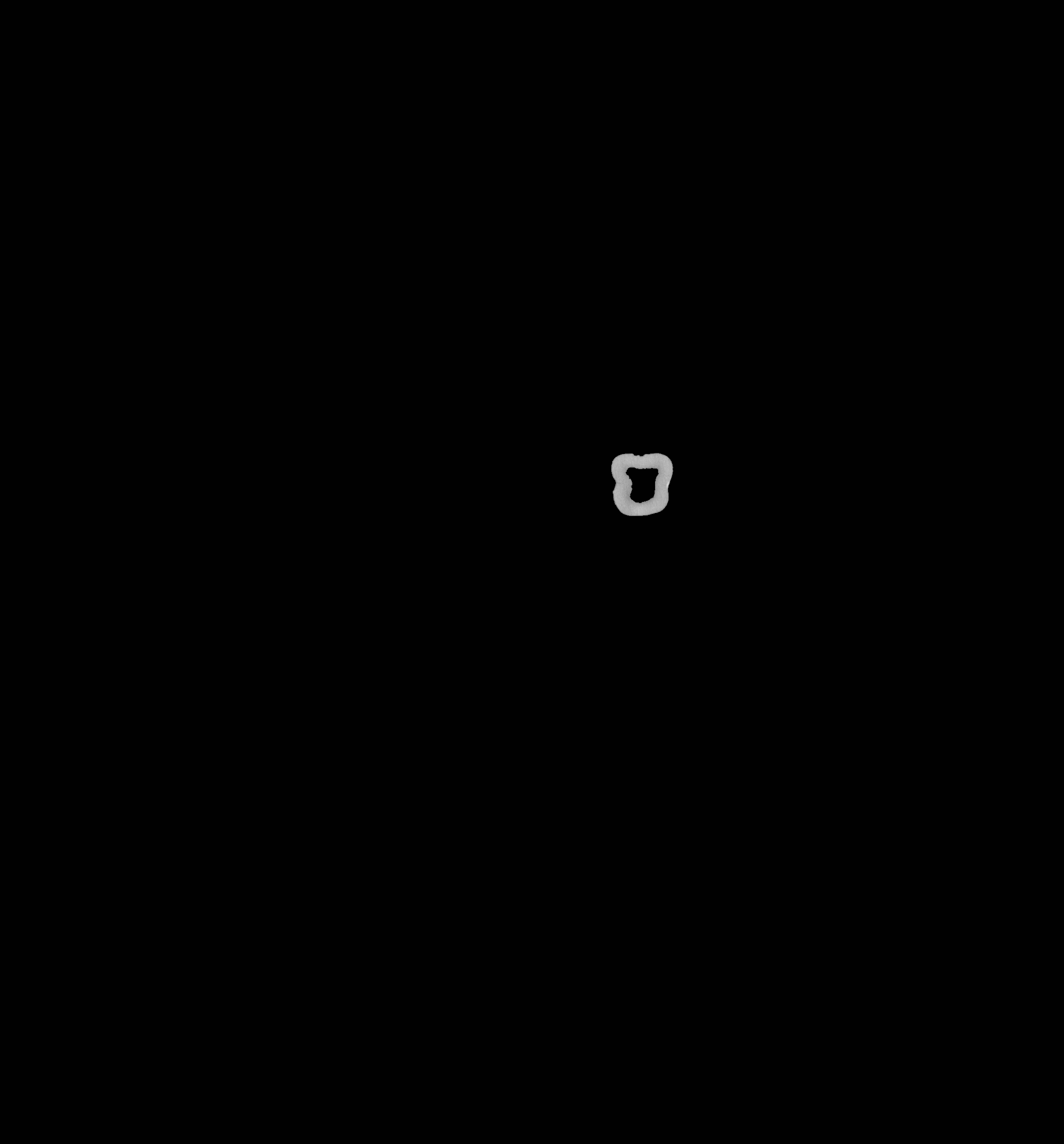

Supplement: Supplementary file 2 — Data S2: Supporting Information. [file AJPA-188-e70164-s001.zip › Cross-Section Tiff Files/mcz_37517_Rm2.tif]

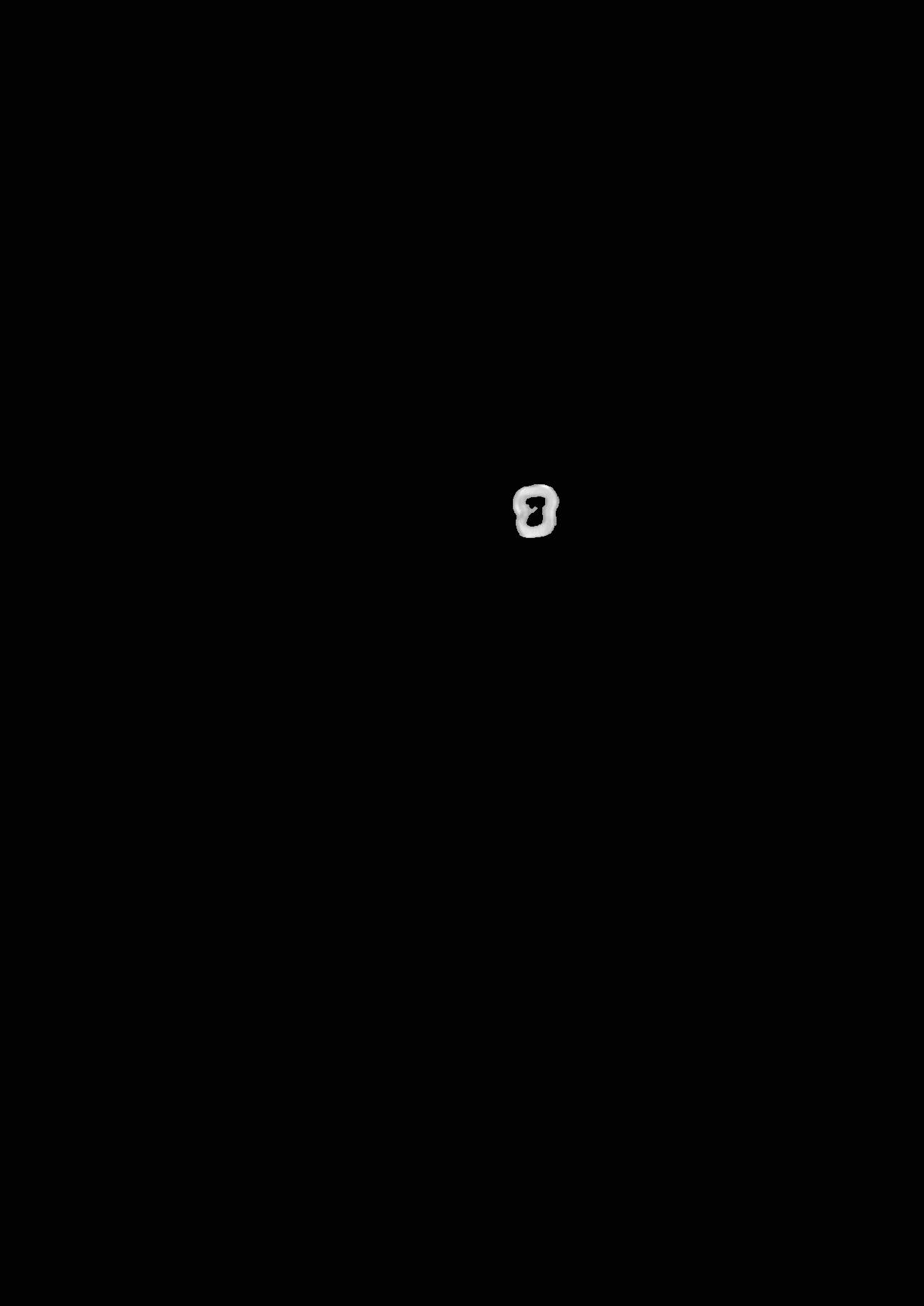

Supplement: Supplementary file 2 — Data S2: Supporting Information. [file AJPA-188-e70164-s001.zip › Cross-Section Tiff Files/mcz_41493_Rm2.tif]

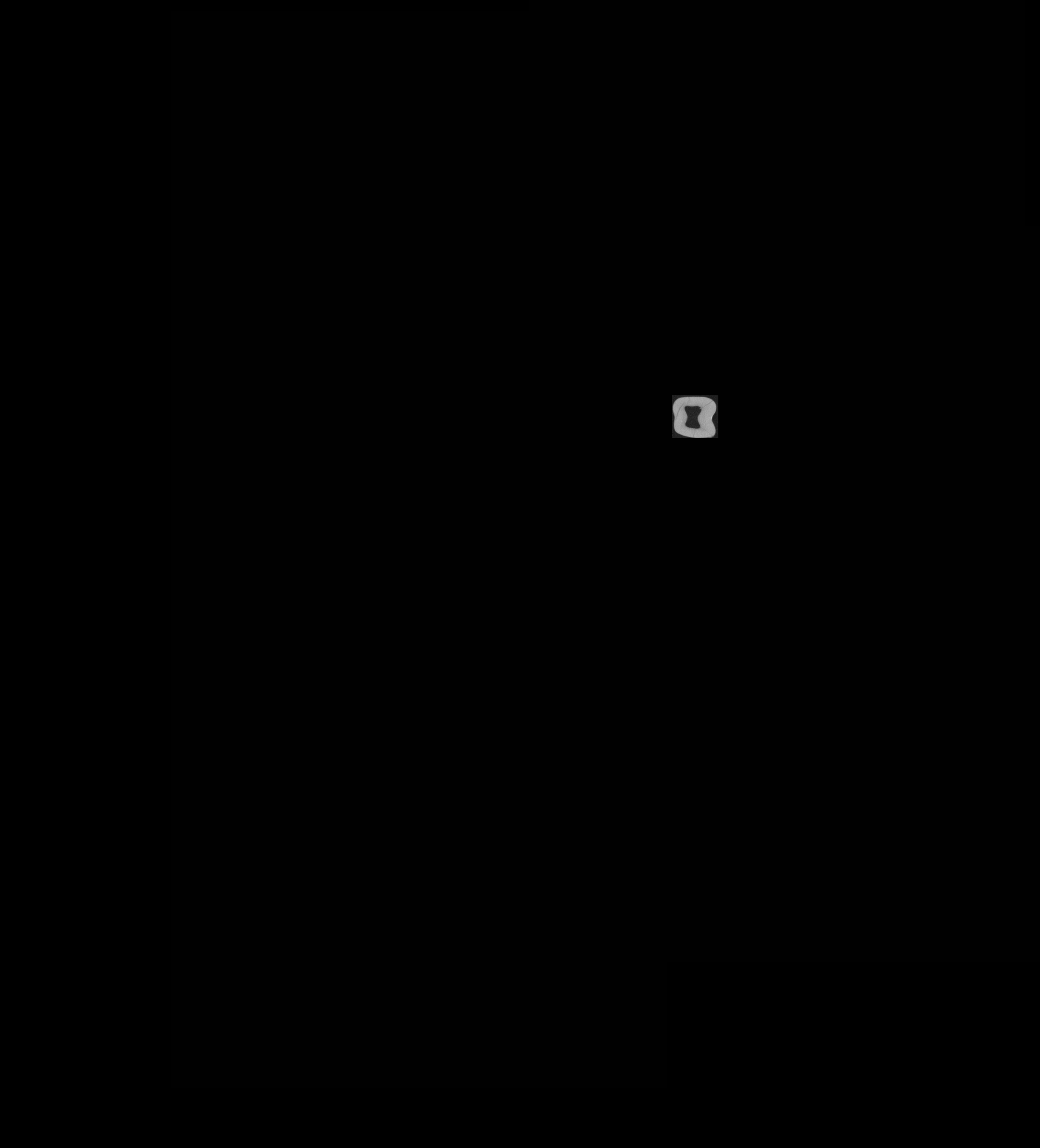

Supplement: Supplementary file 2 — Data S2: Supporting Information. [file AJPA-188-e70164-s001.zip › Cross-Section Tiff Files/amnh_52640_Rm1.tif]

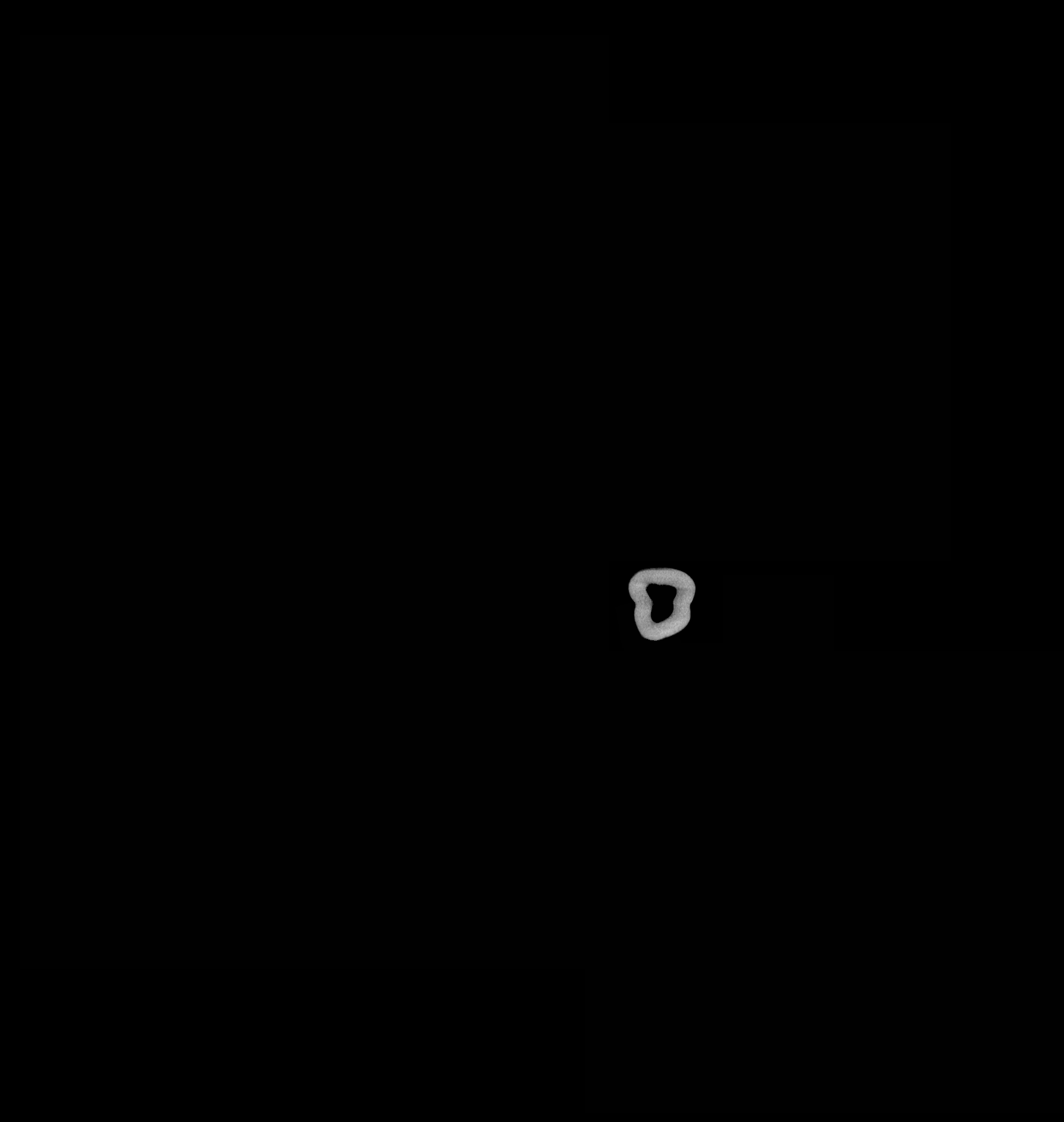

Supplement: Supplementary file 2 — Data S2: Supporting Information. [file AJPA-188-e70164-s001.zip › Cross-Section Tiff Files/mcz_50958_Rm3.tif]

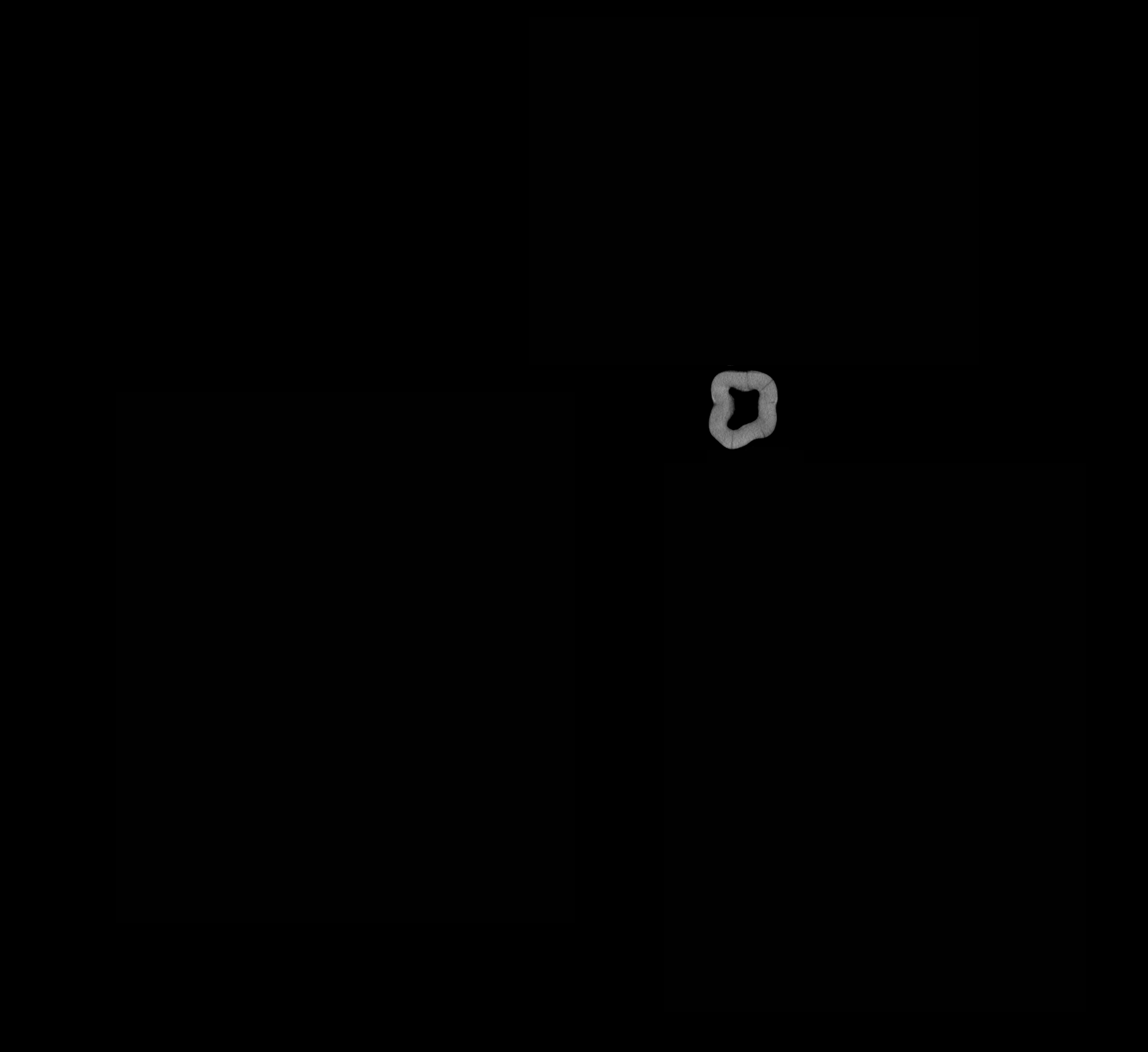

Supplement: Supplementary file 2 — Data S2: Supporting Information. [file AJPA-188-e70164-s001.zip › Cross-Section Tiff Files/mcz_50958_Rm1.tif]

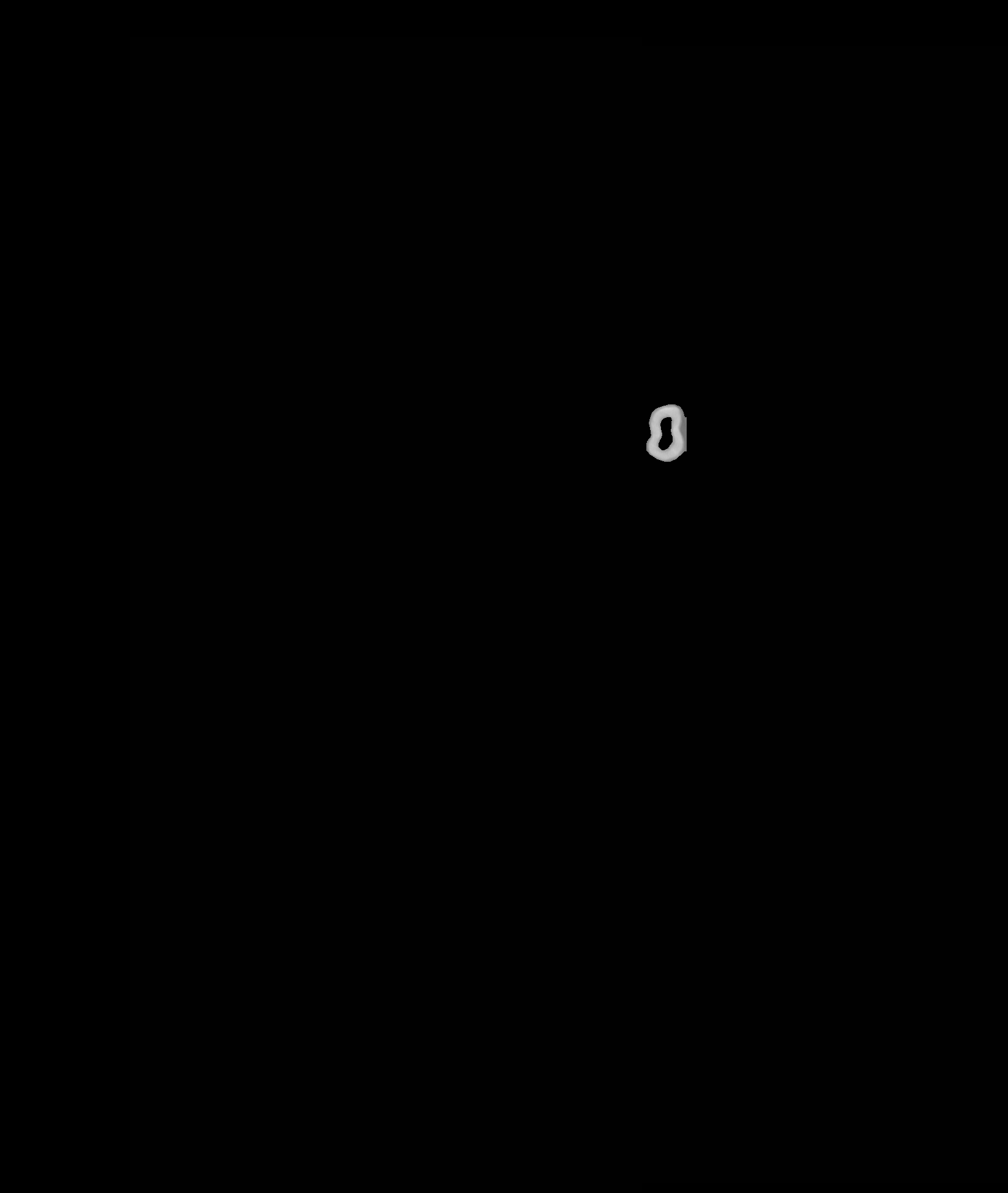

Supplement: Supplementary file 2 — Data S2: Supporting Information. [file AJPA-188-e70164-s001.zip › Cross-Section Tiff Files/mcz_36032_Rm1.tif]

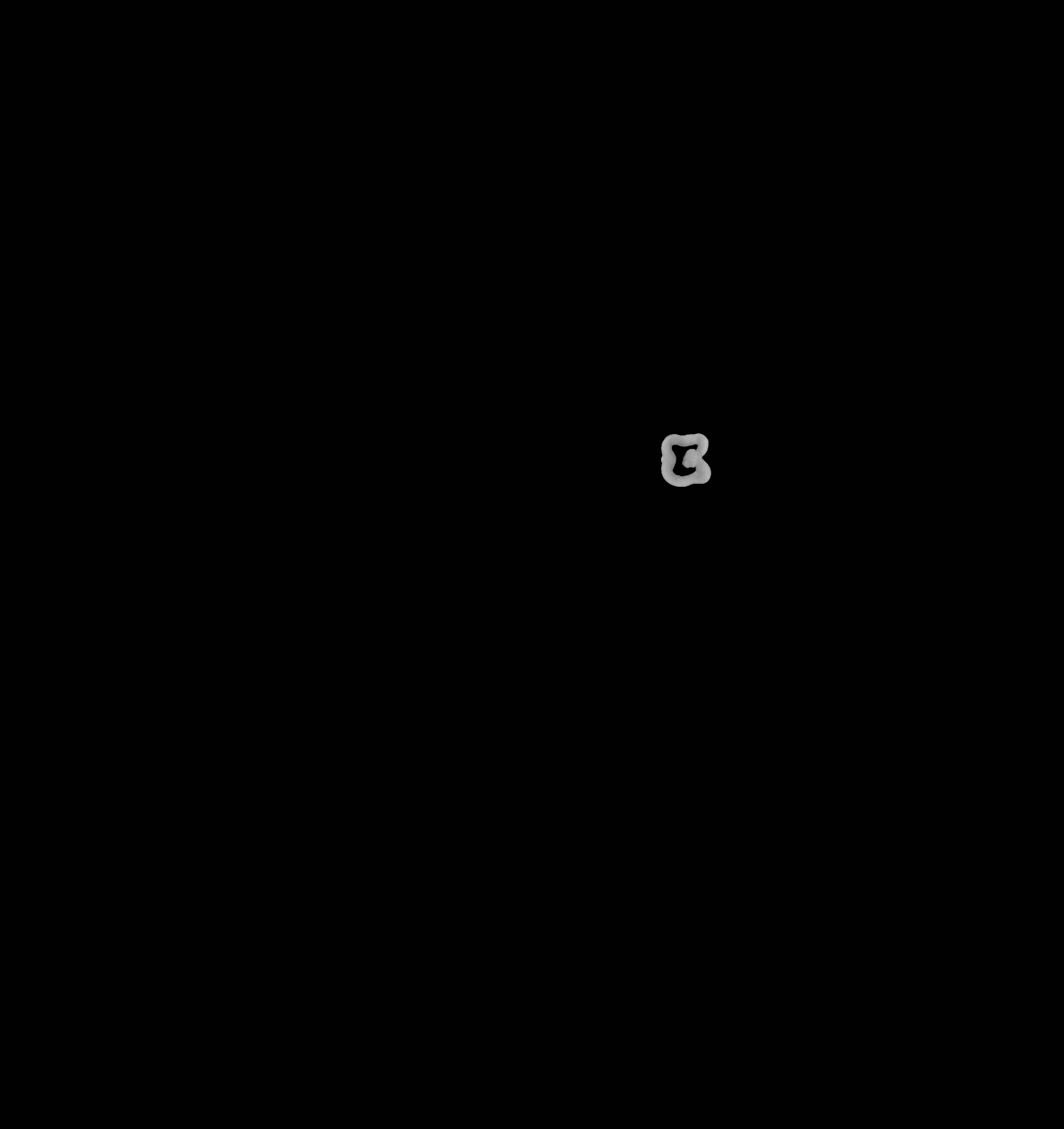

Supplement: Supplementary file 2 — Data S2: Supporting Information. [file AJPA-188-e70164-s001.zip › Cross-Section Tiff Files/mcz_20039_Rm2.tif]

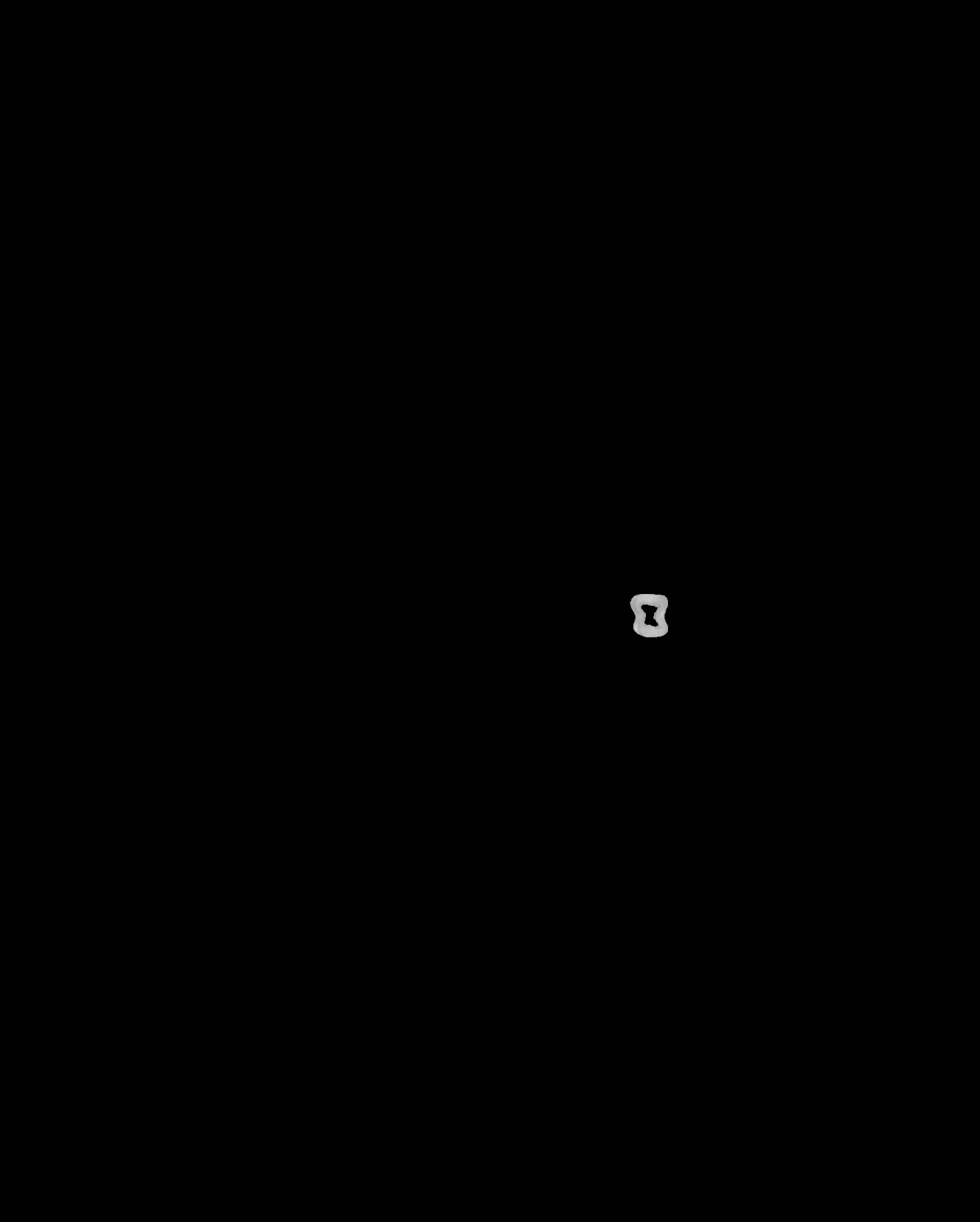

Supplement: Supplementary file 2 — Data S2: Supporting Information. [file AJPA-188-e70164-s001.zip › Cross-Section Tiff Files/mcz_23196_Rm2.tif]

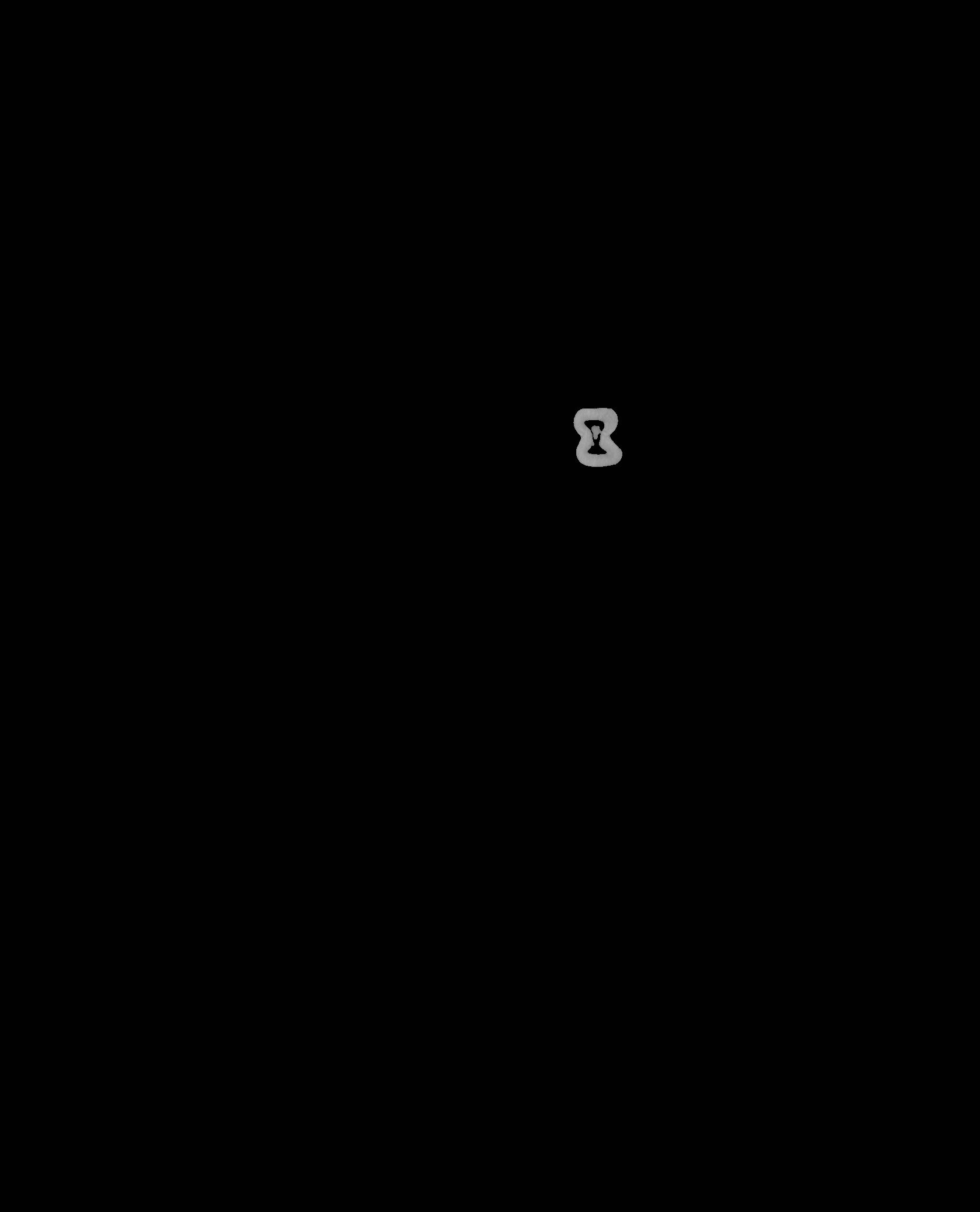

Supplement: Supplementary file 2 — Data S2: Supporting Information. [file AJPA-188-e70164-s001.zip › Cross-Section Tiff Files/mcz_21161_Rm1.tif]

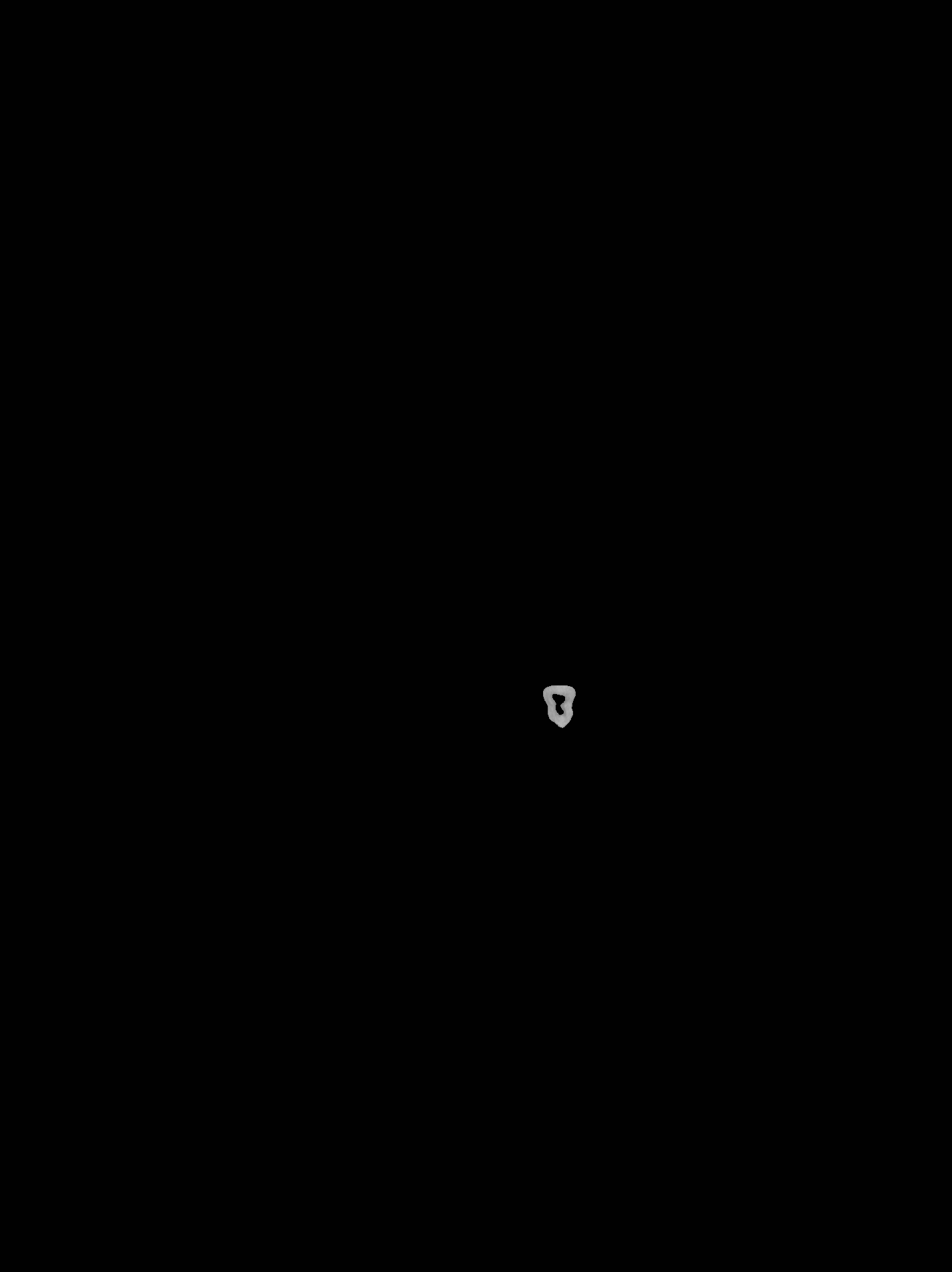

Supplement: Supplementary file 2 — Data S2: Supporting Information. [file AJPA-188-e70164-s001.zip › Cross-Section Tiff Files/mcz_23196_Rm3.tif]

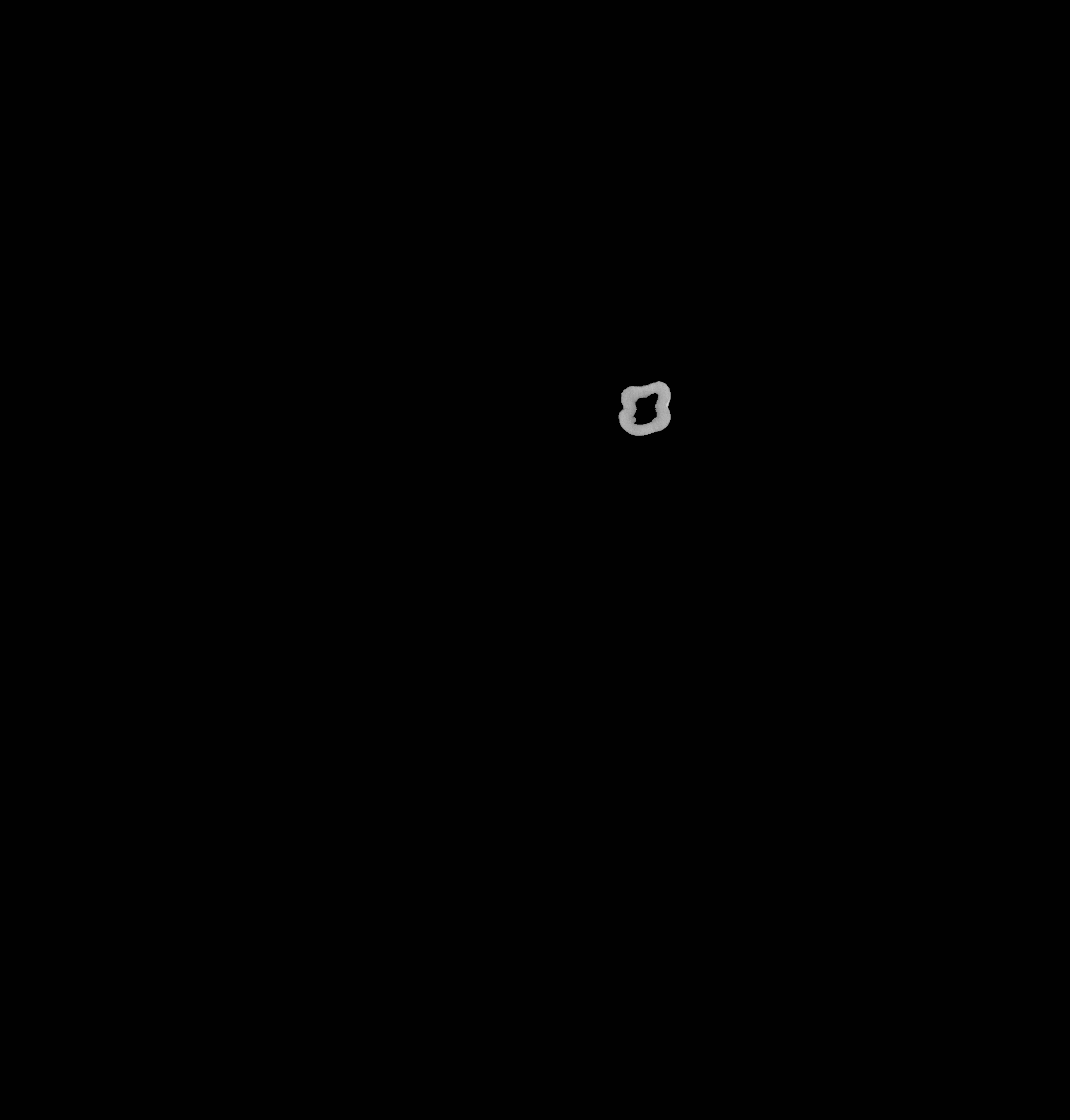

Supplement: Supplementary file 2 — Data S2: Supporting Information. [file AJPA-188-e70164-s001.zip › Cross-Section Tiff Files/mcz_37517_Rm1.tif]

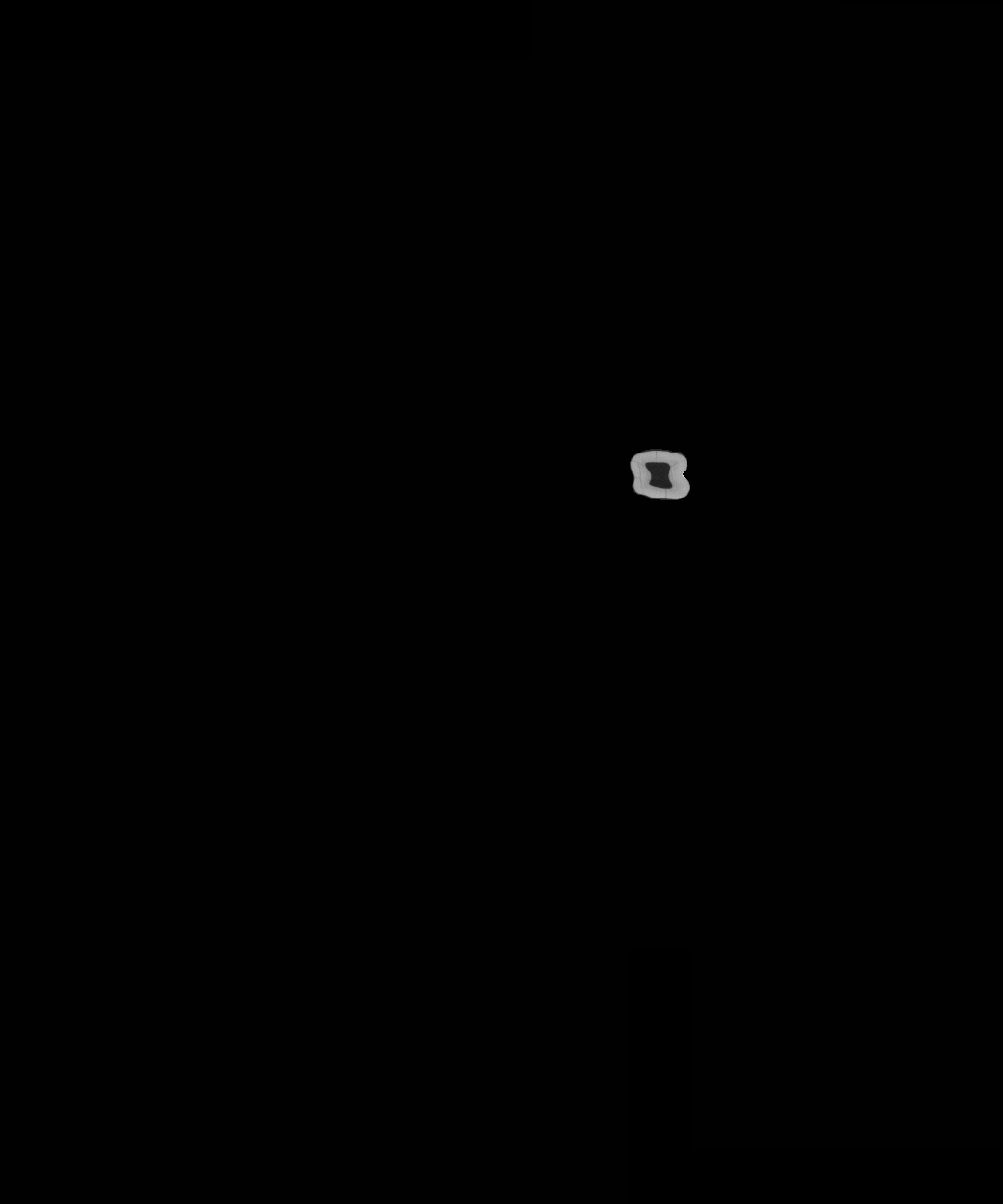

Supplement: Supplementary file 2 — Data S2: Supporting Information. [file AJPA-188-e70164-s001.zip › Cross-Section Tiff Files/amnh_52640_Rm2.tif]

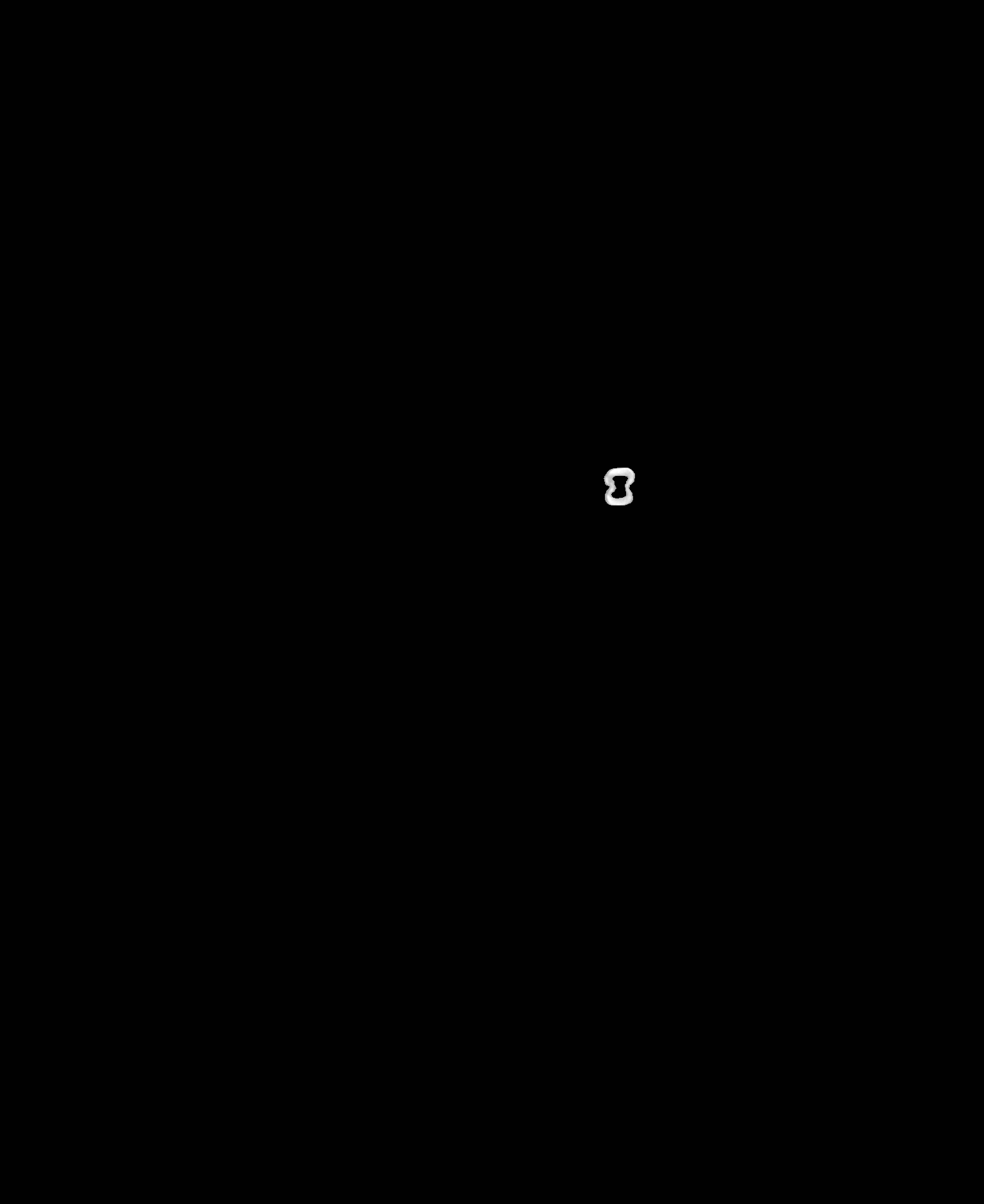

Supplement: Supplementary file 2 — Data S2: Supporting Information. [file AJPA-188-e70164-s001.zip › Cross-Section Tiff Files/mcz_41493_Rm1.tif]

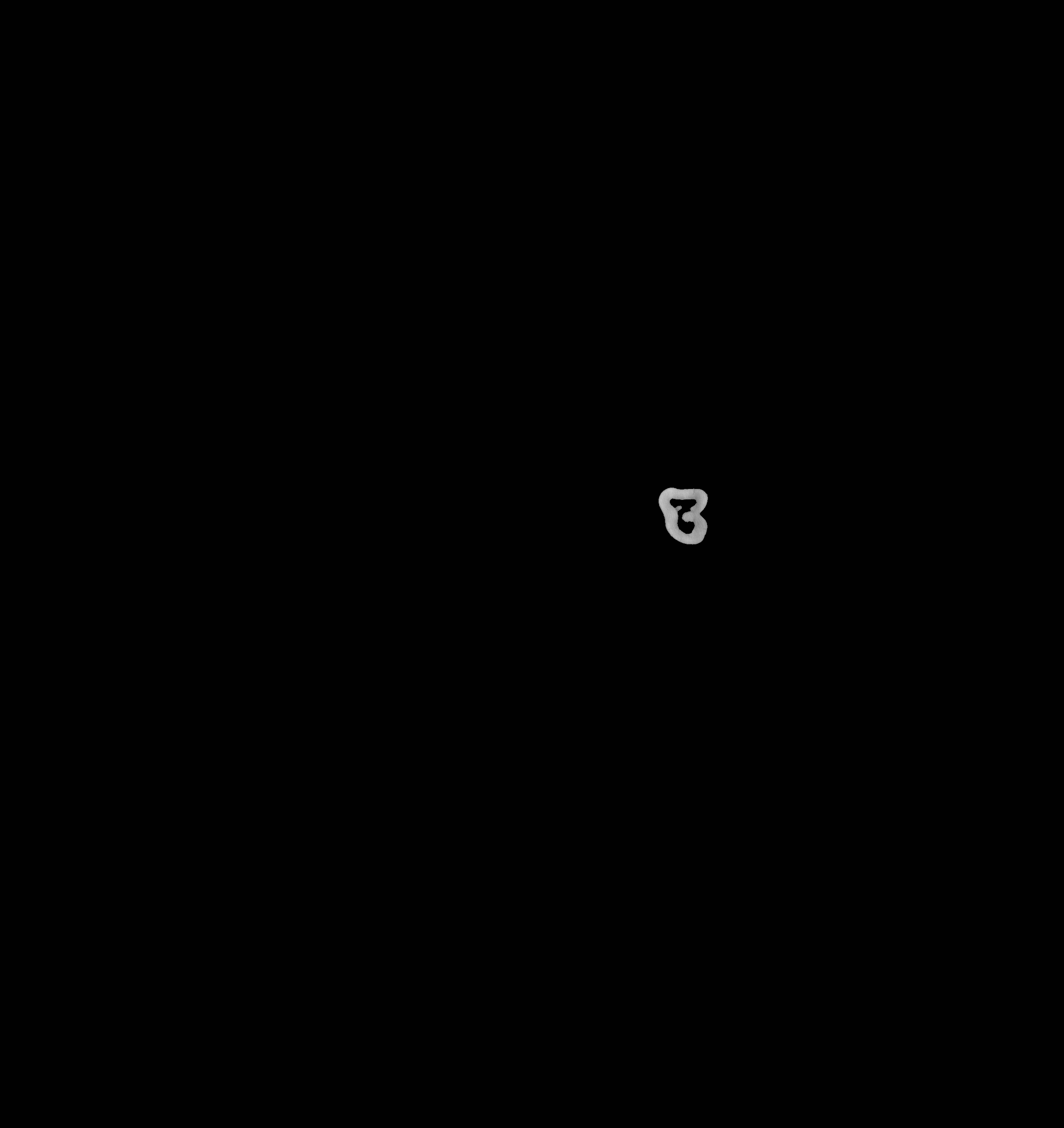

Supplement: Supplementary file 2 — Data S2: Supporting Information. [file AJPA-188-e70164-s001.zip › Cross-Section Tiff Files/mcz_20039_Rm3.tif]

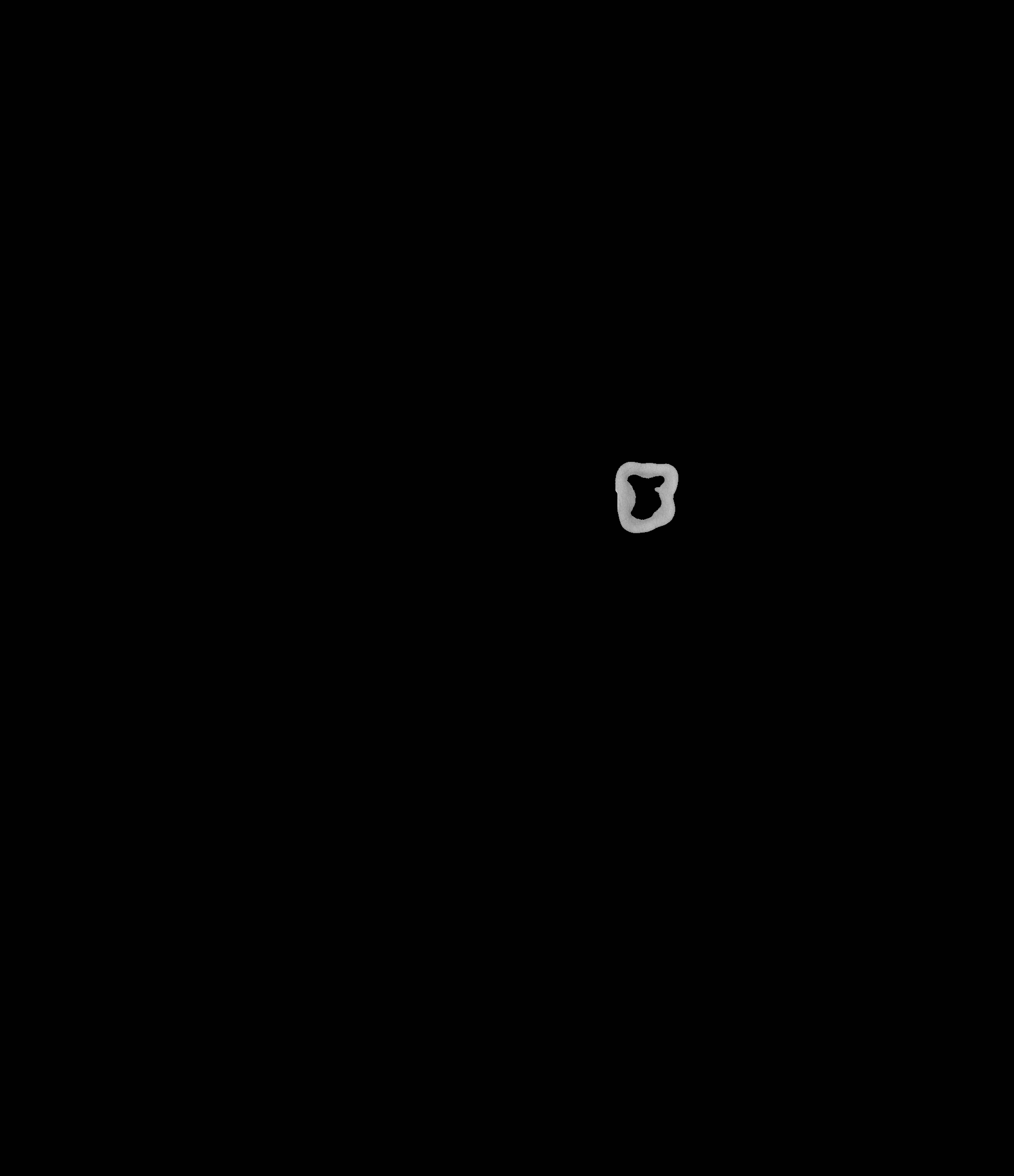

Supplement: Supplementary file 2 — Data S2: Supporting Information. [file AJPA-188-e70164-s001.zip › Cross-Section Tiff Files/mcz_20038_Rm2.tif]

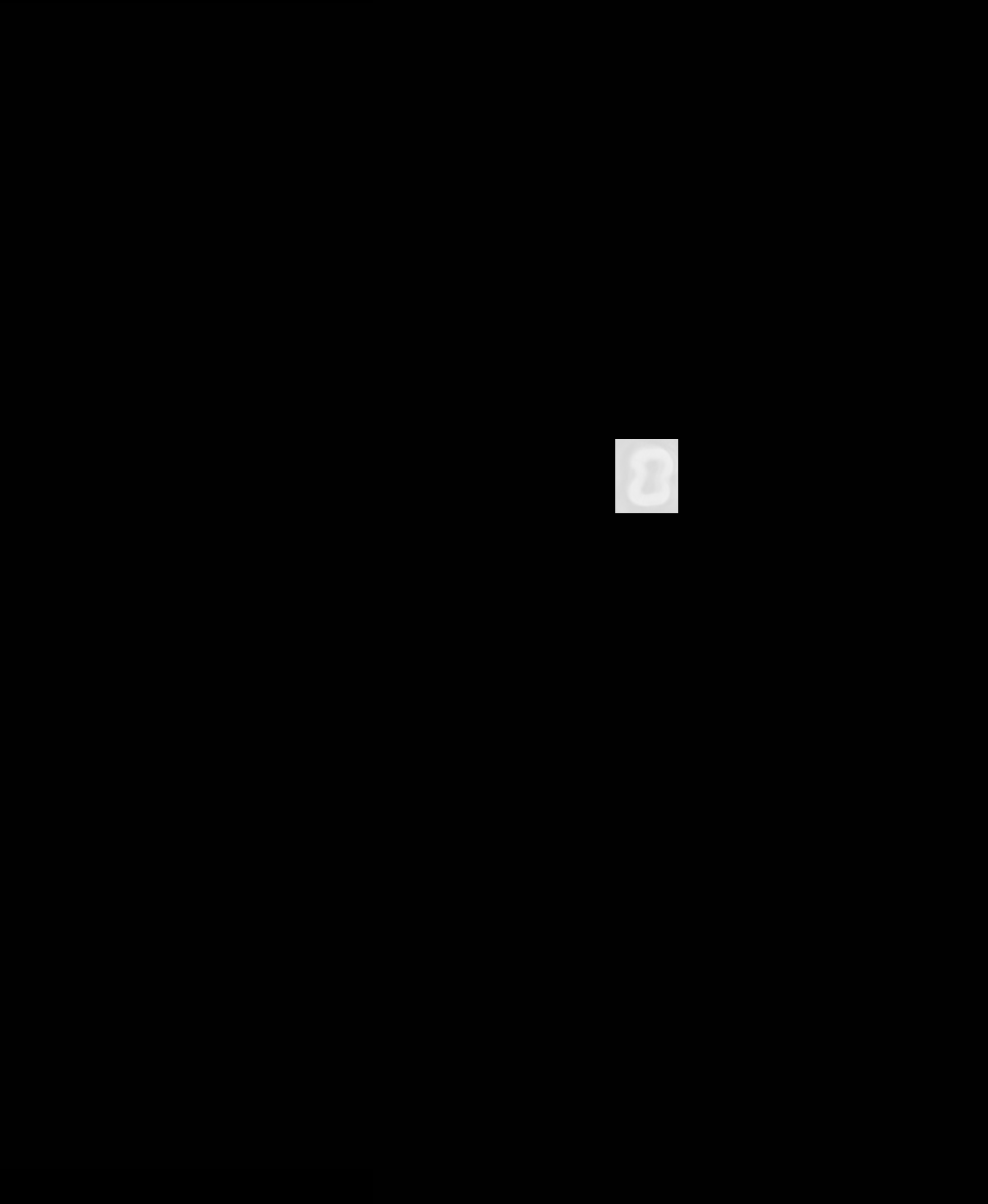

Supplement: Supplementary file 2 — Data S2: Supporting Information. [file AJPA-188-e70164-s001.zip › Cross-Section Tiff Files/mcz_41414_Rm1.tif]

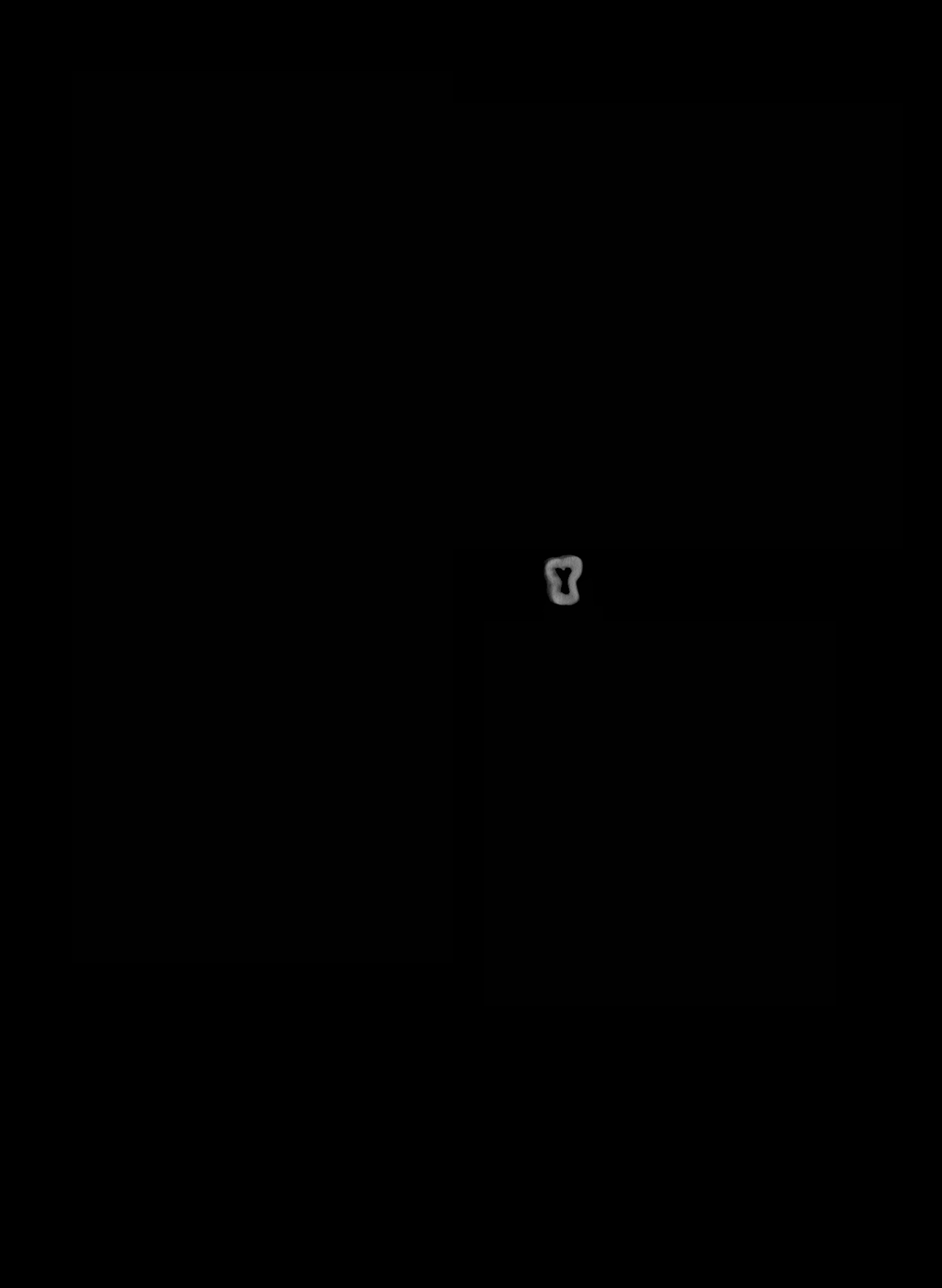

Supplement: Supplementary file 2 — Data S2: Supporting Information. [file AJPA-188-e70164-s001.zip › Cross-Section Tiff Files/mcz_47016_Rm3.tif]

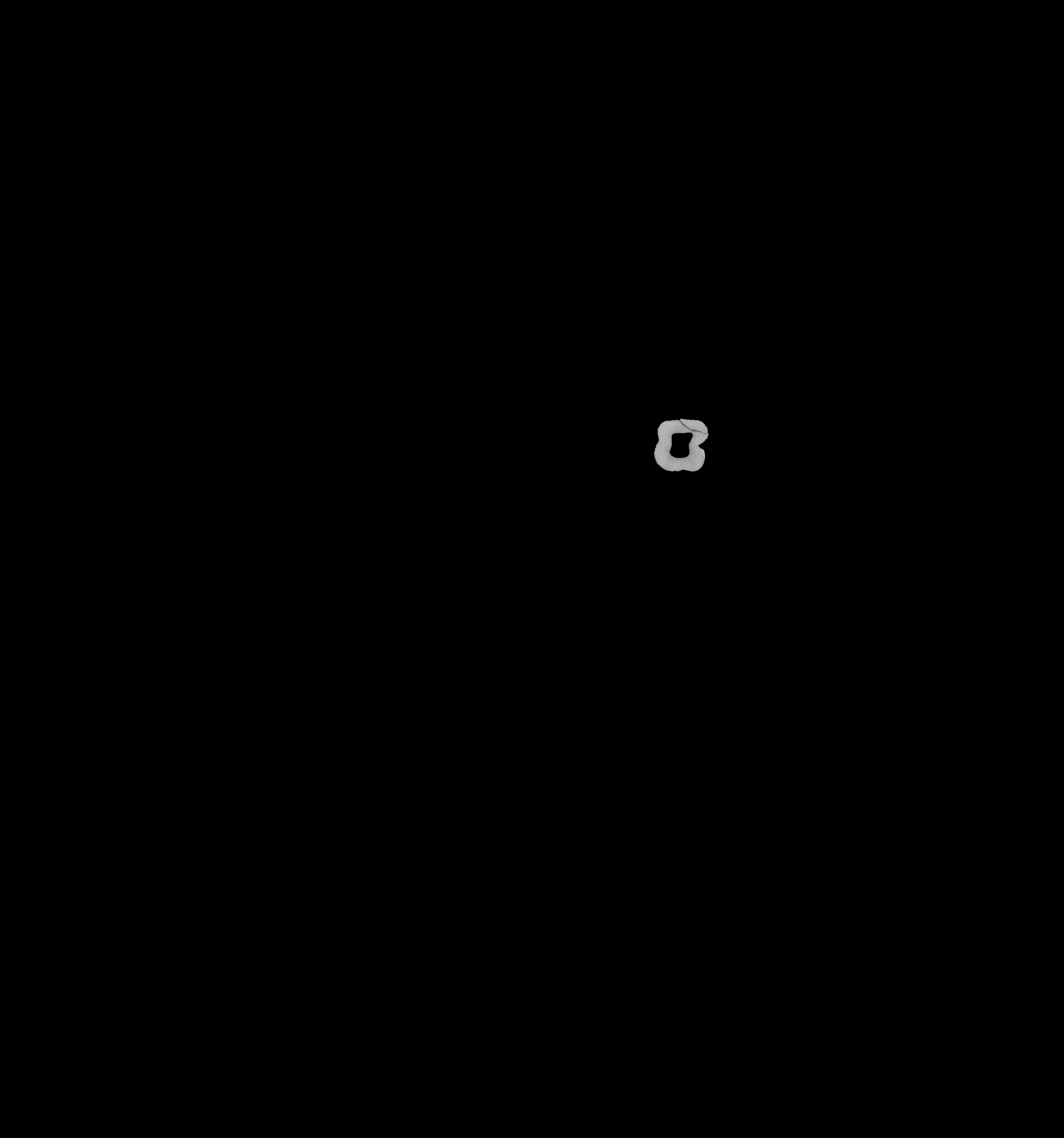

Supplement: Supplementary file 2 — Data S2: Supporting Information. [file AJPA-188-e70164-s001.zip › Cross-Section Tiff Files/mcz_19187_Rm1.tif]

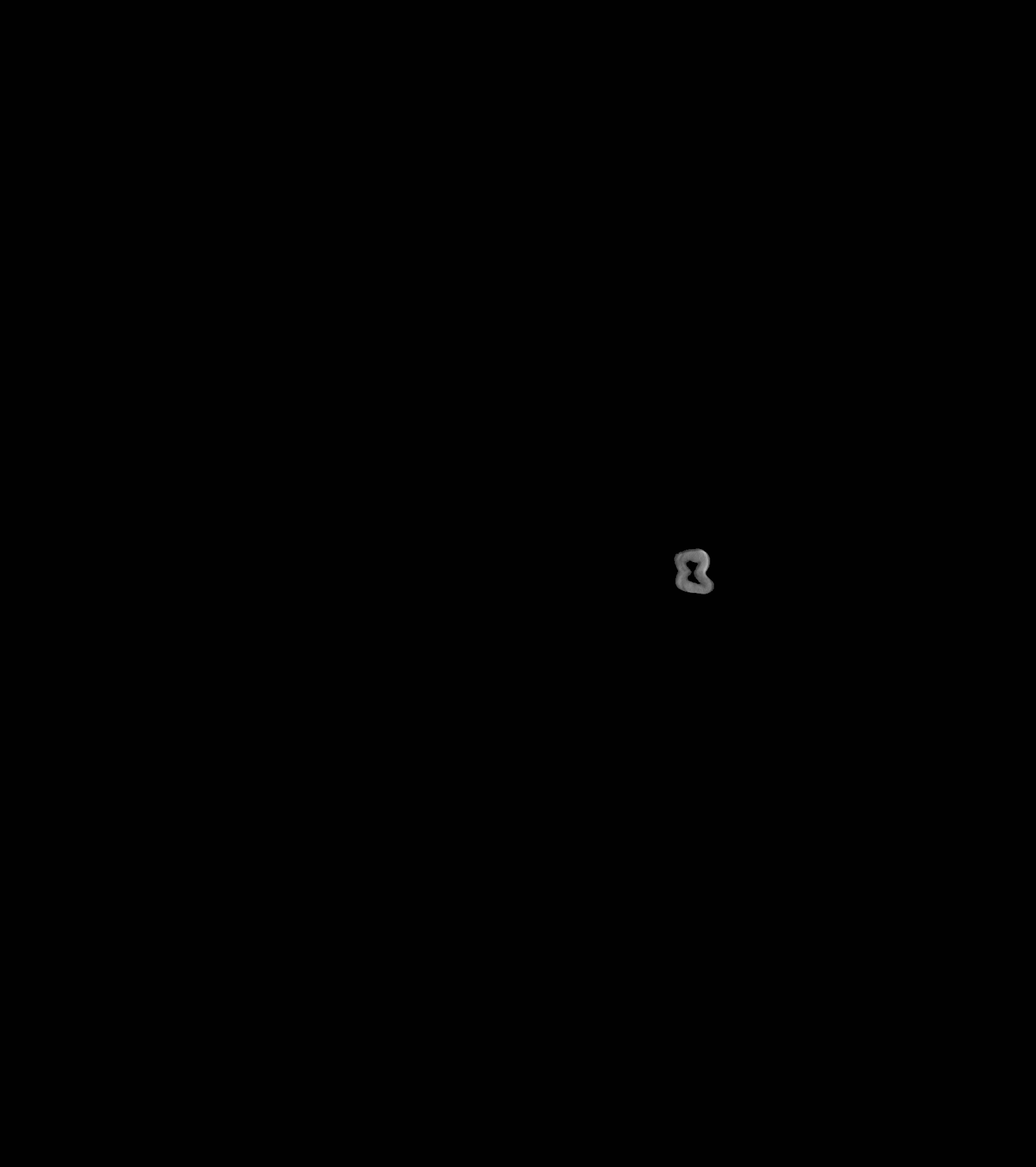

Supplement: Supplementary file 2 — Data S2: Supporting Information. [file AJPA-188-e70164-s001.zip › Cross-Section Tiff Files/mcz_19976_Rm1.tif]

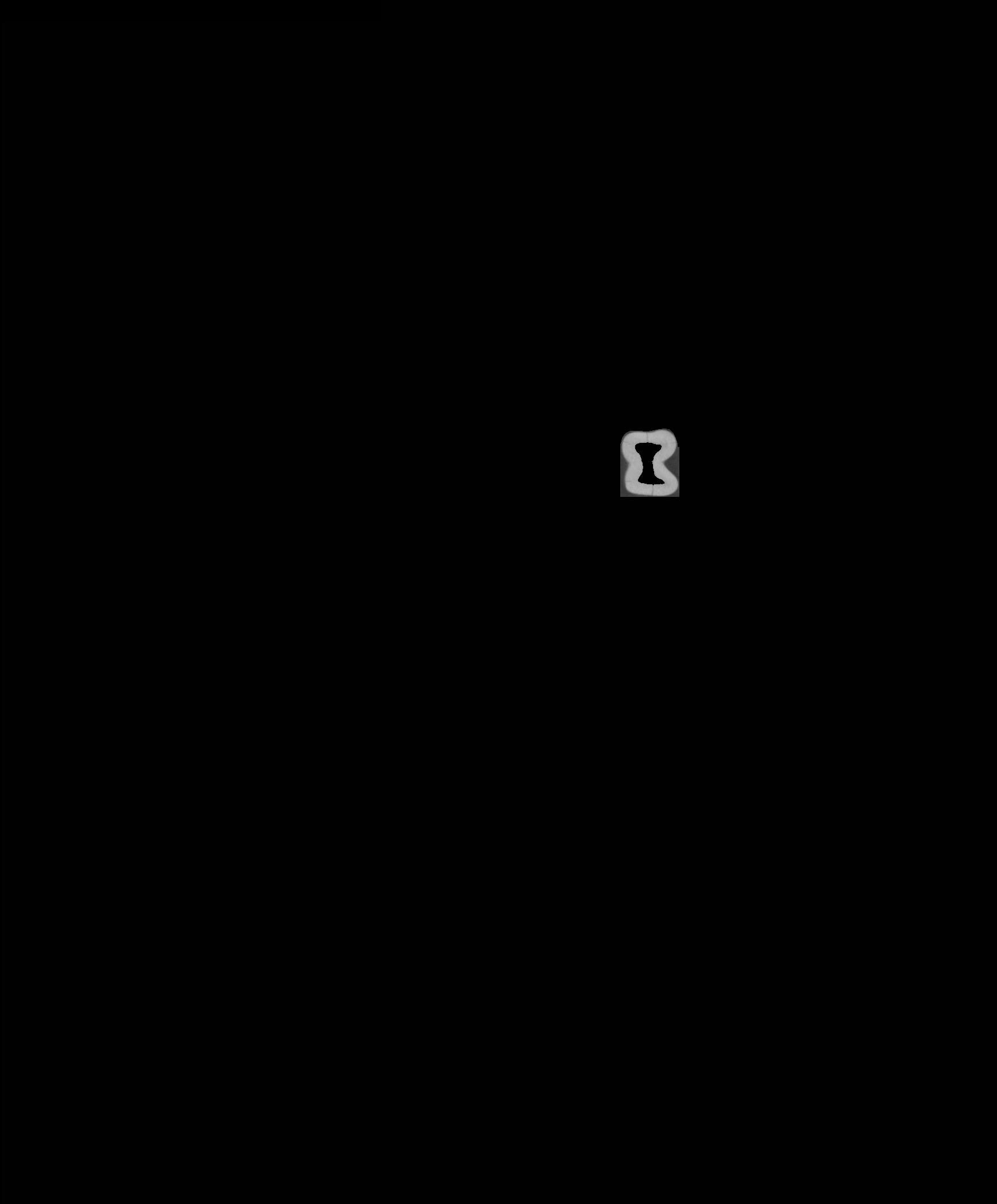

Supplement: Supplementary file 2 — Data S2: Supporting Information. [file AJPA-188-e70164-s001.zip › Cross-Section Tiff Files/mcz_21160_Rm1.tif]

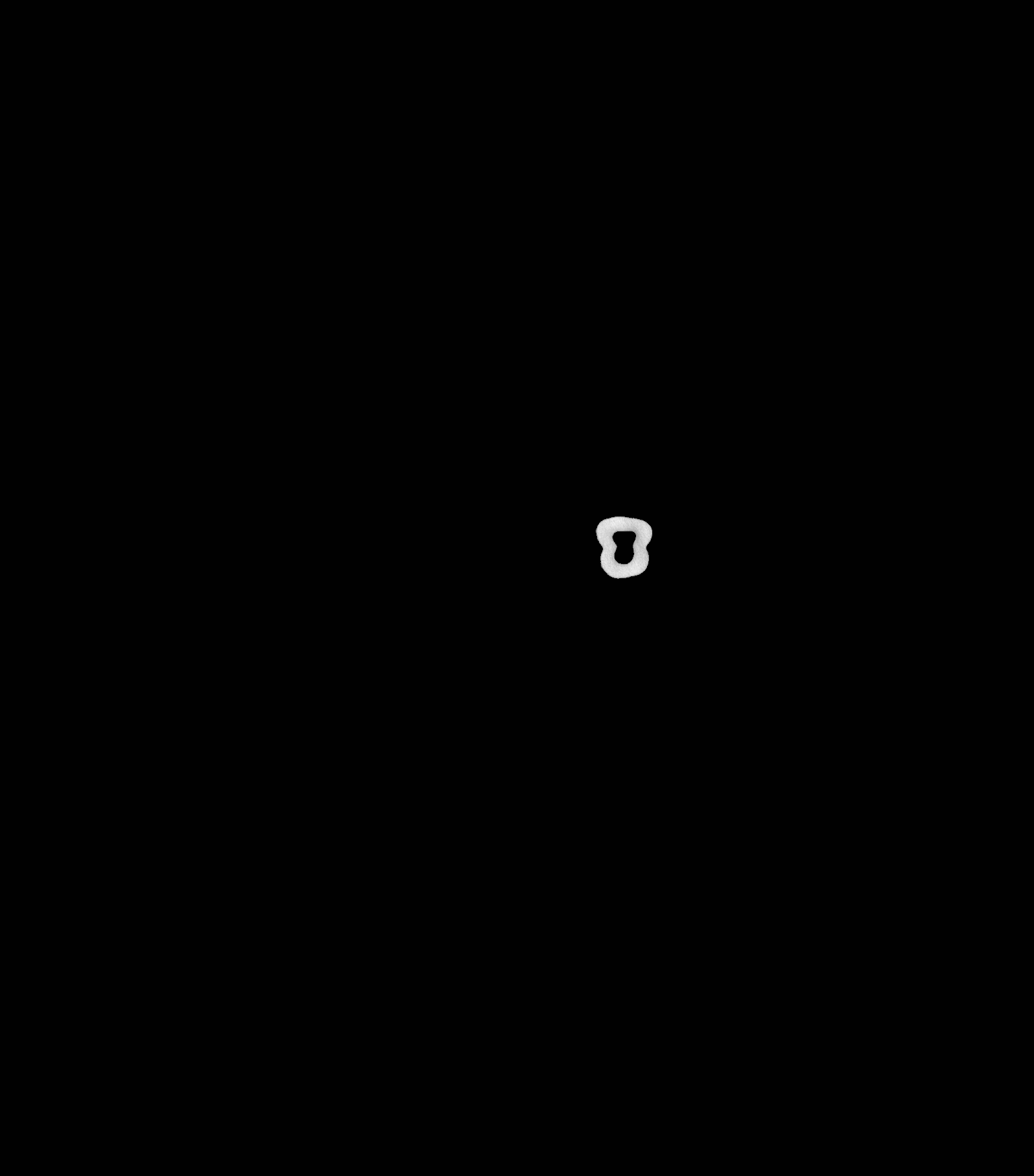

Supplement: Supplementary file 2 — Data S2: Supporting Information. [file AJPA-188-e70164-s001.zip › Cross-Section Tiff Files/mcz_23163_Rm3.tif]

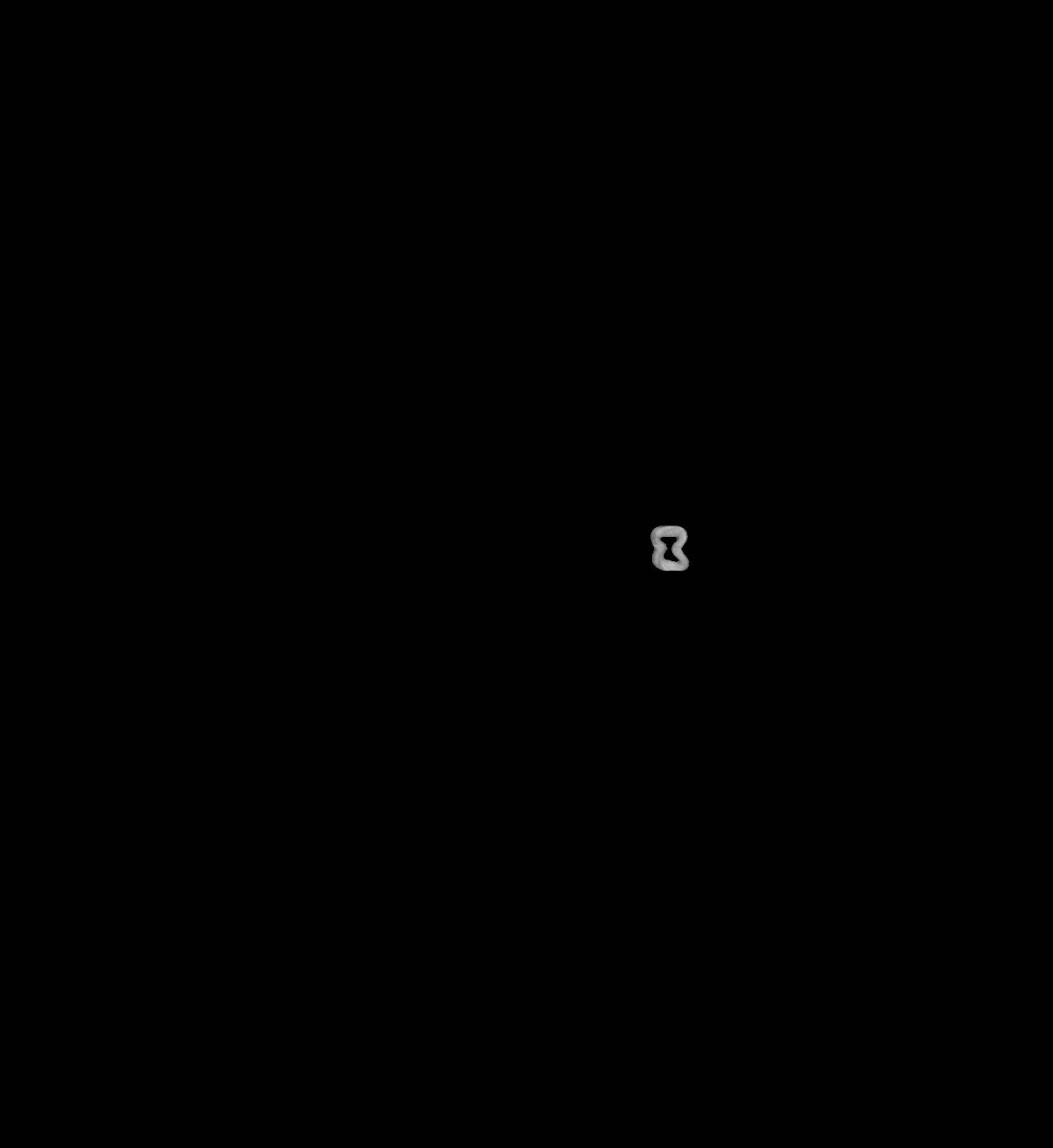

Supplement: Supplementary file 2 — Data S2: Supporting Information. [file AJPA-188-e70164-s001.zip › Cross-Section Tiff Files/mcz_23197_Rm2.tif]

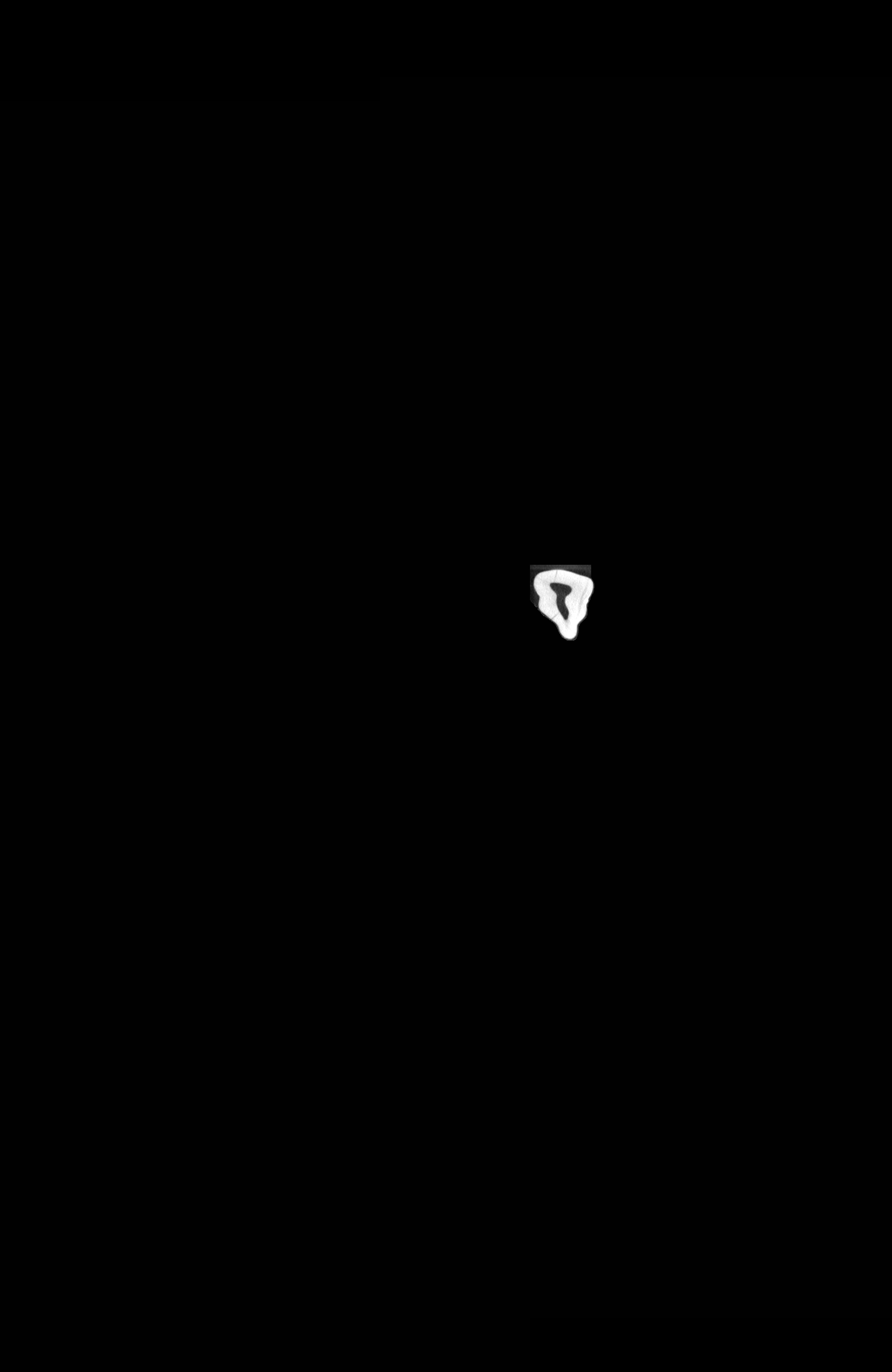

Supplement: Supplementary file 2 — Data S2: Supporting Information. [file AJPA-188-e70164-s001.zip › Cross-Section Tiff Files/amnh_52641_Rm3.tif]

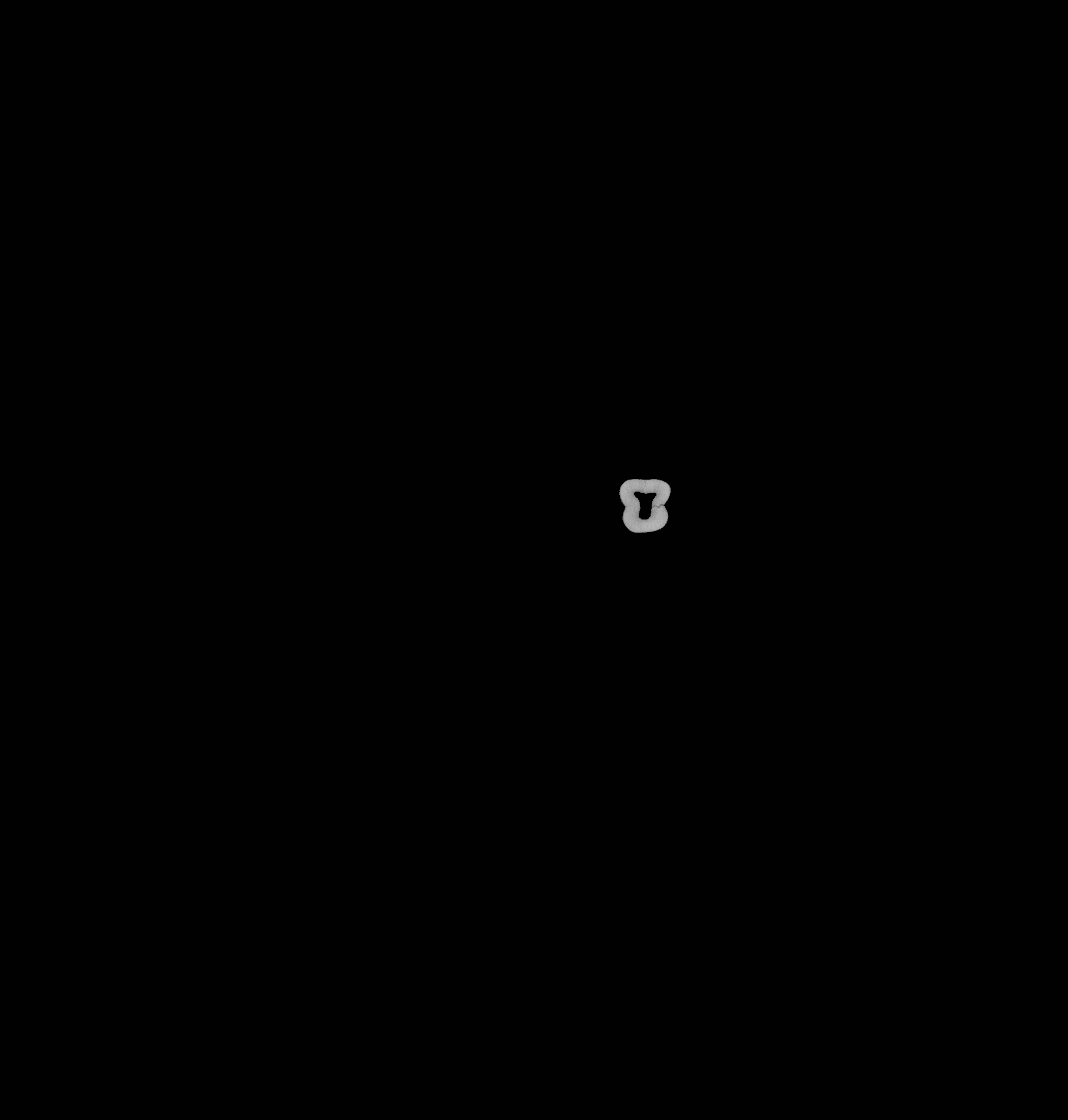

Supplement: Supplementary file 2 — Data S2: Supporting Information. [file AJPA-188-e70164-s001.zip › Cross-Section Tiff Files/mcz_37365_Rm2.tif]

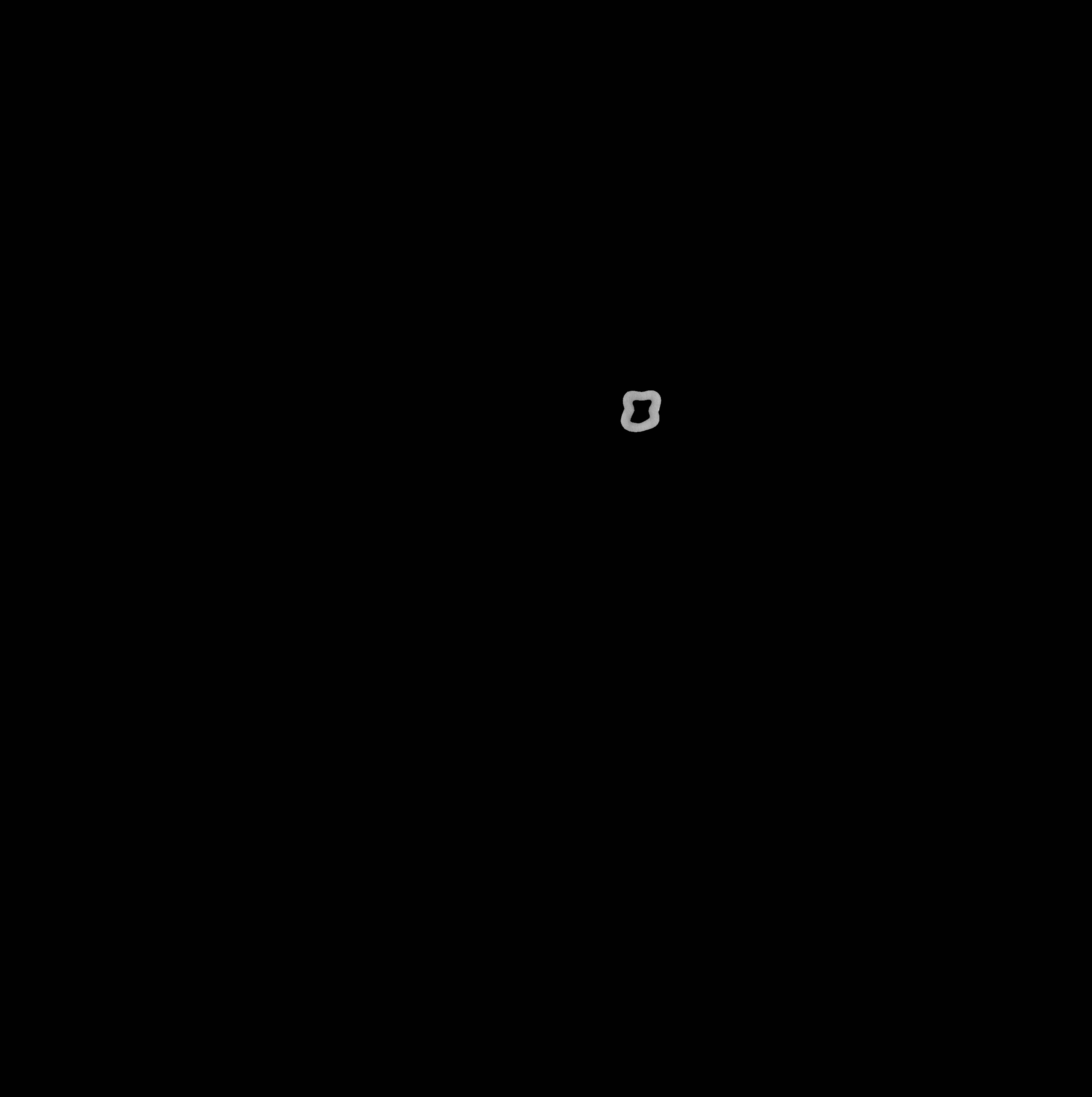

Supplement: Supplementary file 2 — Data S2: Supporting Information. [file AJPA-188-e70164-s001.zip › Cross-Section Tiff Files/mcz_37516_Rm1.tif]

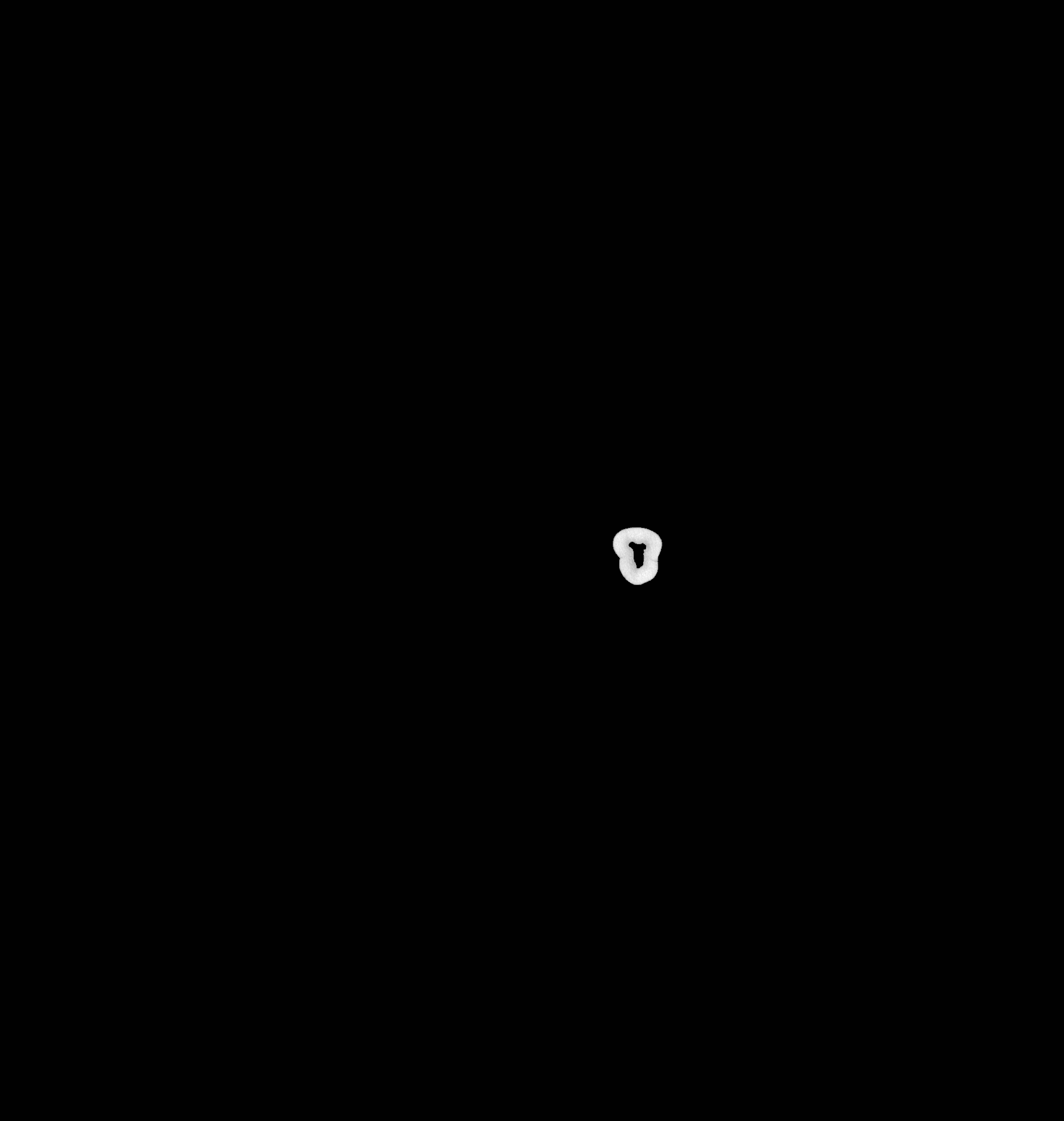

Supplement: Supplementary file 2 — Data S2: Supporting Information. [file AJPA-188-e70164-s001.zip › Cross-Section Tiff Files/mcz_37365_Rm3.tif]

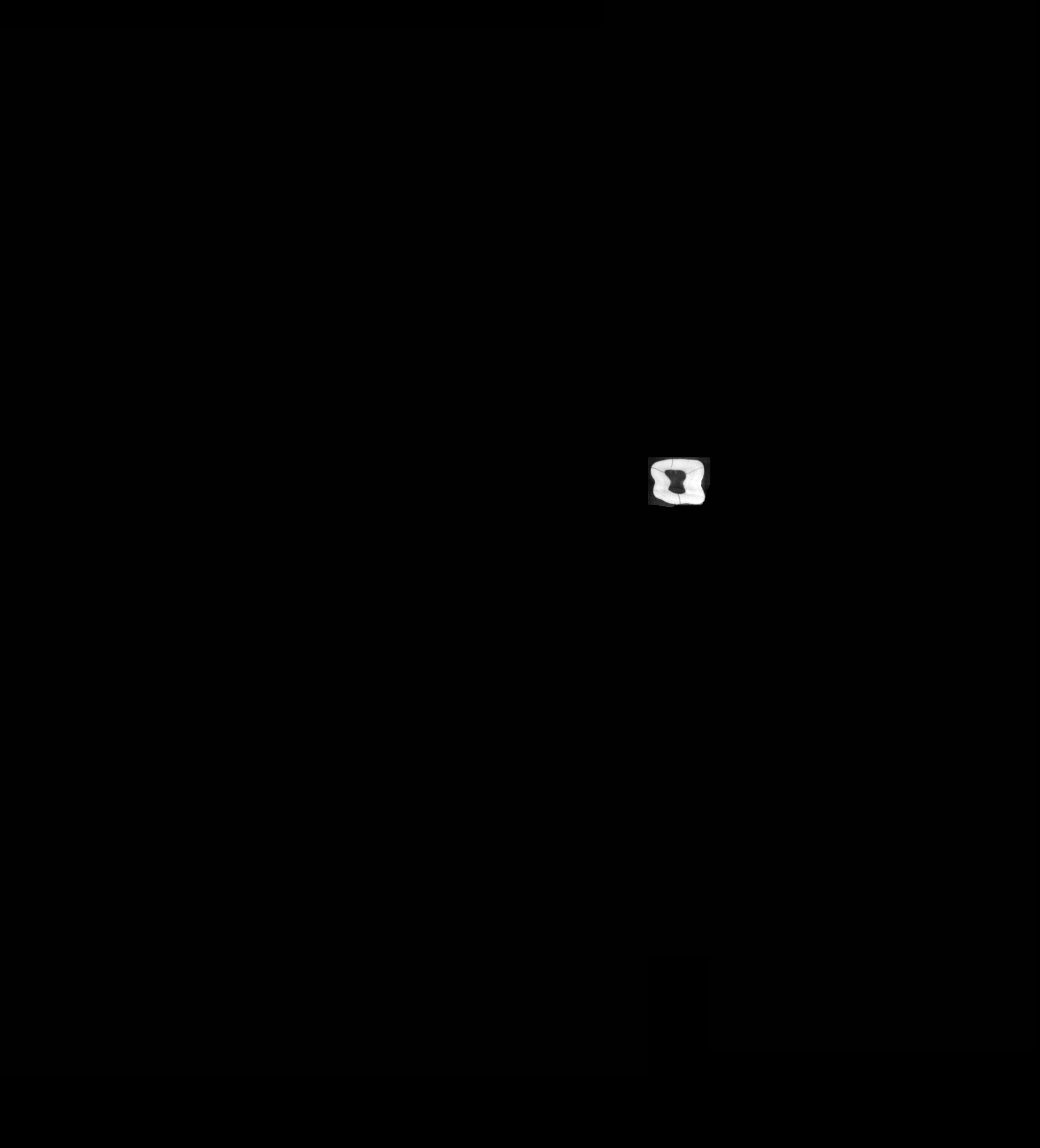

Supplement: Supplementary file 2 — Data S2: Supporting Information. [file AJPA-188-e70164-s001.zip › Cross-Section Tiff Files/amnh_52641_Rm2.tif]

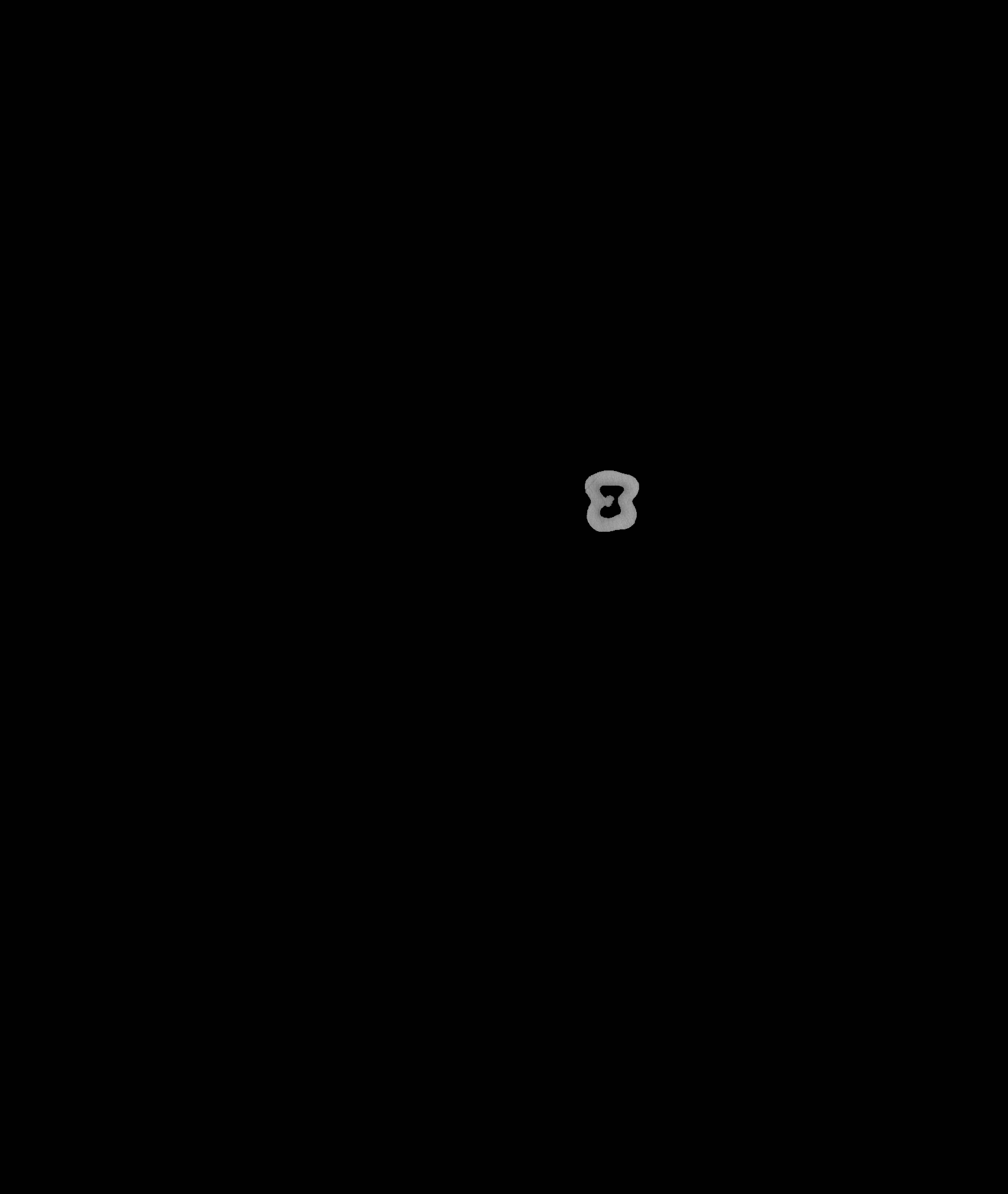

Supplement: Supplementary file 2 — Data S2: Supporting Information. [file AJPA-188-e70164-s001.zip › Cross-Section Tiff Files/mcz_23163_Rm2.tif]

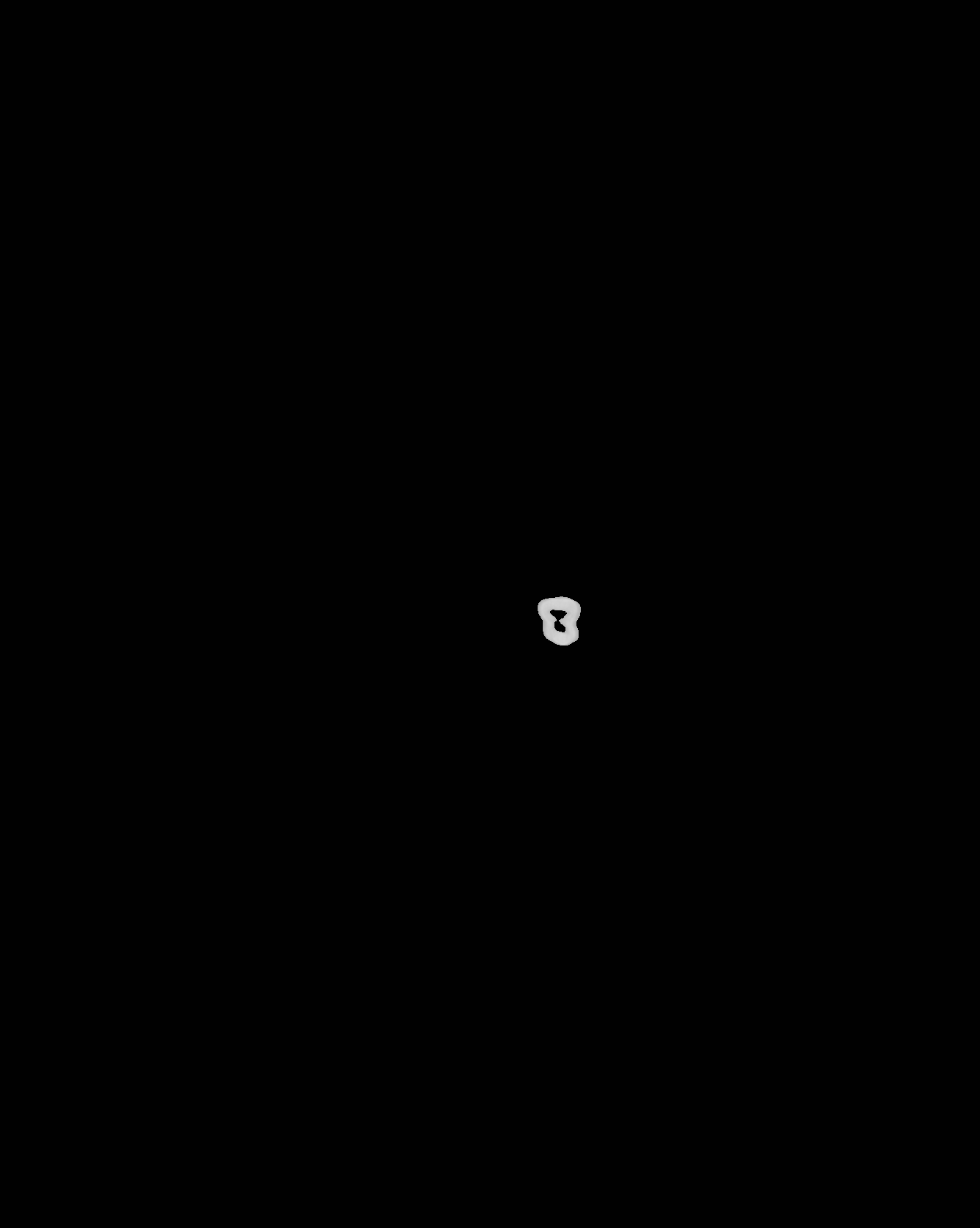

Supplement: Supplementary file 2 — Data S2: Supporting Information. [file AJPA-188-e70164-s001.zip › Cross-Section Tiff Files/mcz_23197_Rm3.tif]

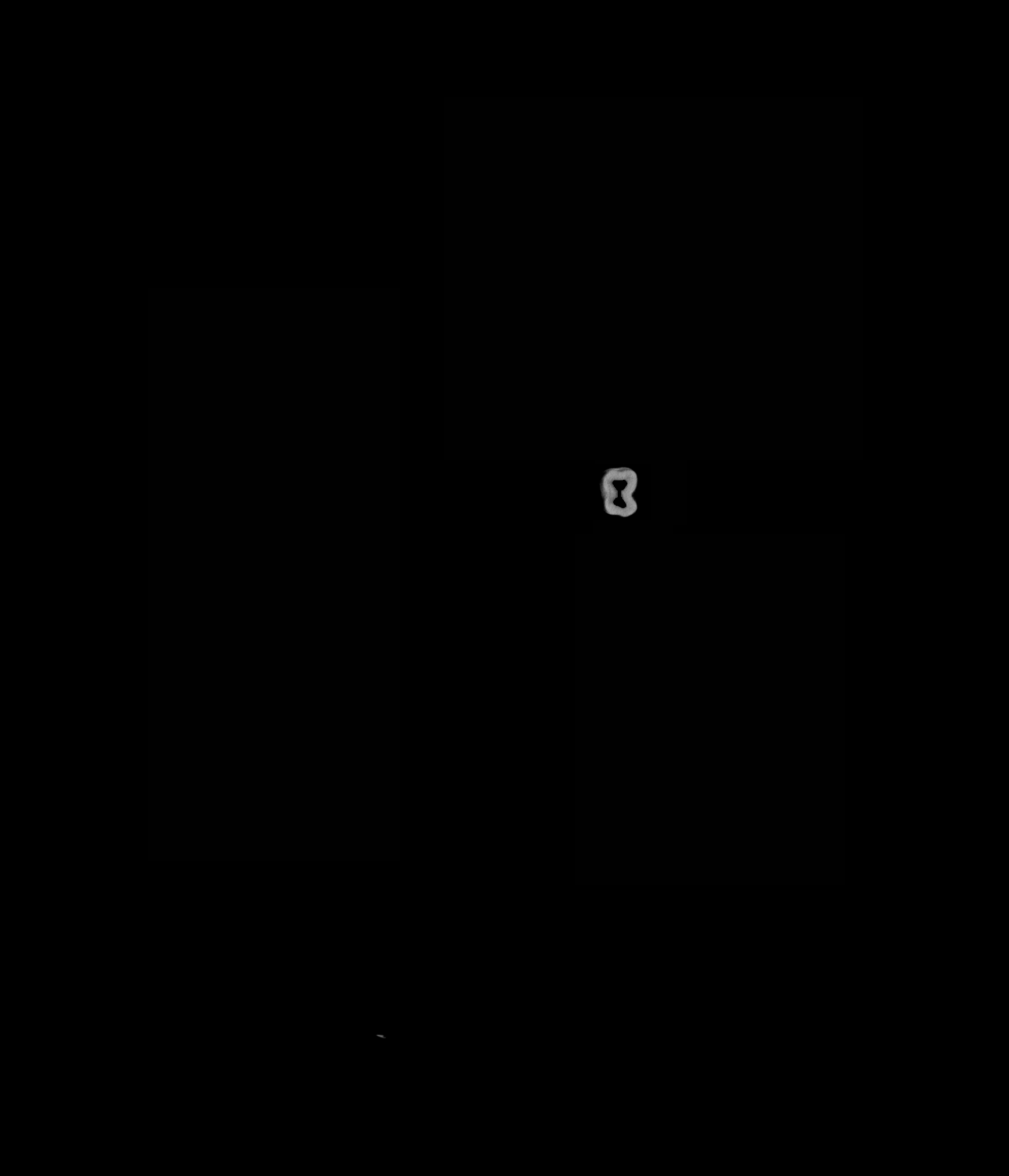

Supplement: Supplementary file 2 — Data S2: Supporting Information. [file AJPA-188-e70164-s001.zip › Cross-Section Tiff Files/mcz_47016_Rm2.tif]

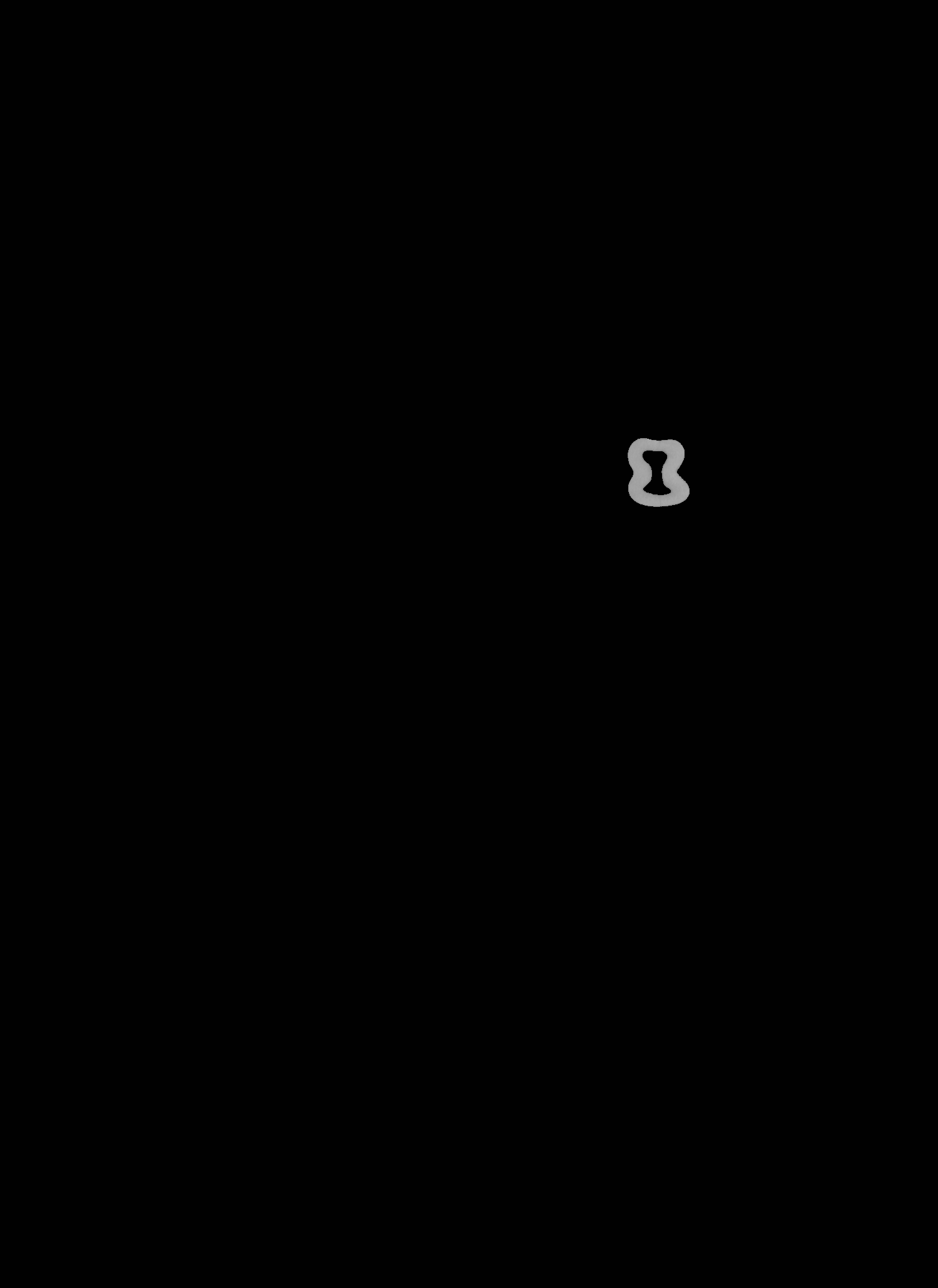

Supplement: Supplementary file 2 — Data S2: Supporting Information. [file AJPA-188-e70164-s001.zip › Cross-Section Tiff Files/mcz_8304_Rm1.tif]

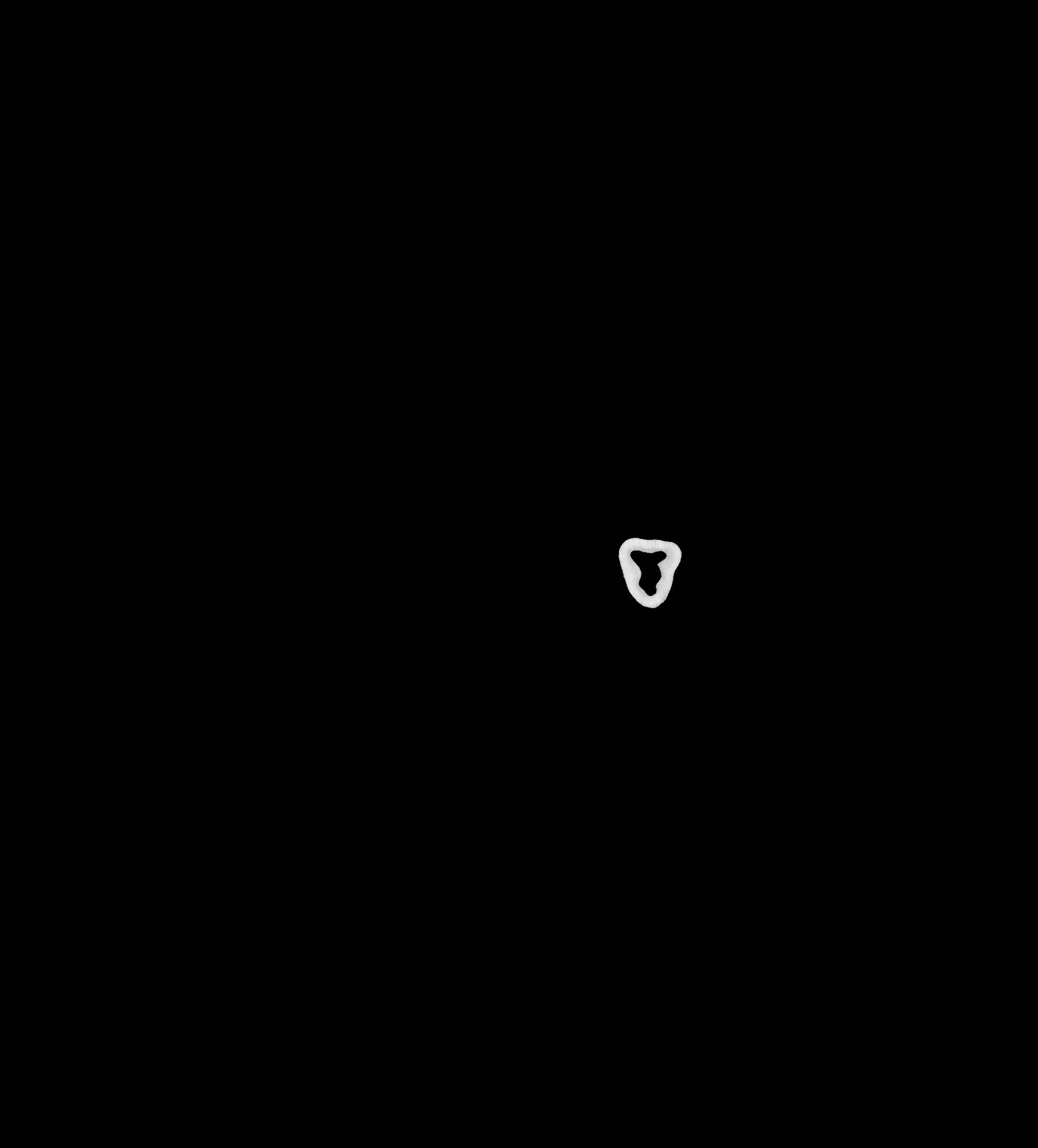

Supplement: Supplementary file 2 — Data S2: Supporting Information. [file AJPA-188-e70164-s001.zip › Cross-Section Tiff Files/mcz_20038_Rm3.tif]

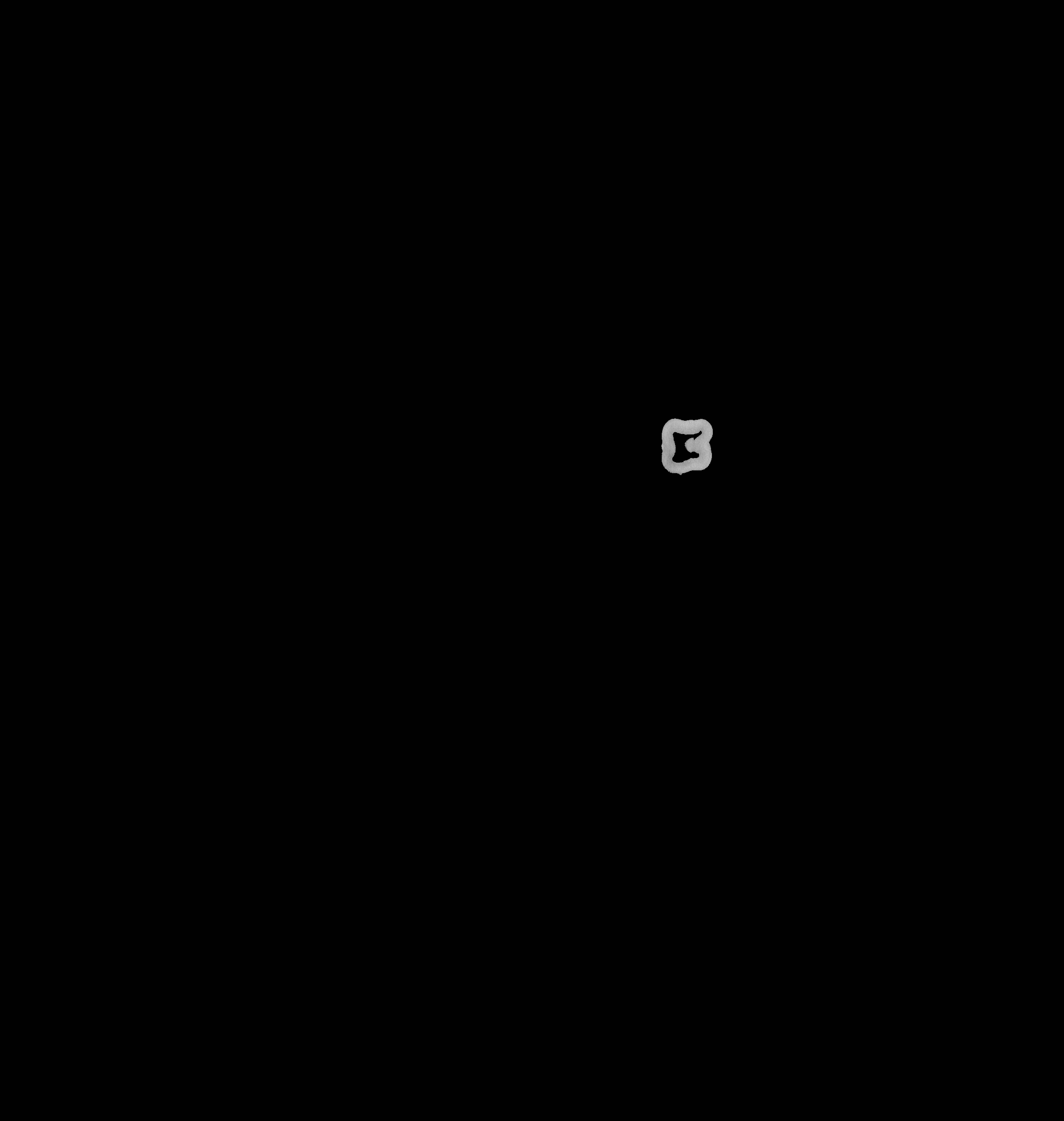

Supplement: Supplementary file 2 — Data S2: Supporting Information. [file AJPA-188-e70164-s001.zip › Cross-Section Tiff Files/mcz_20038_Rm1.tif]

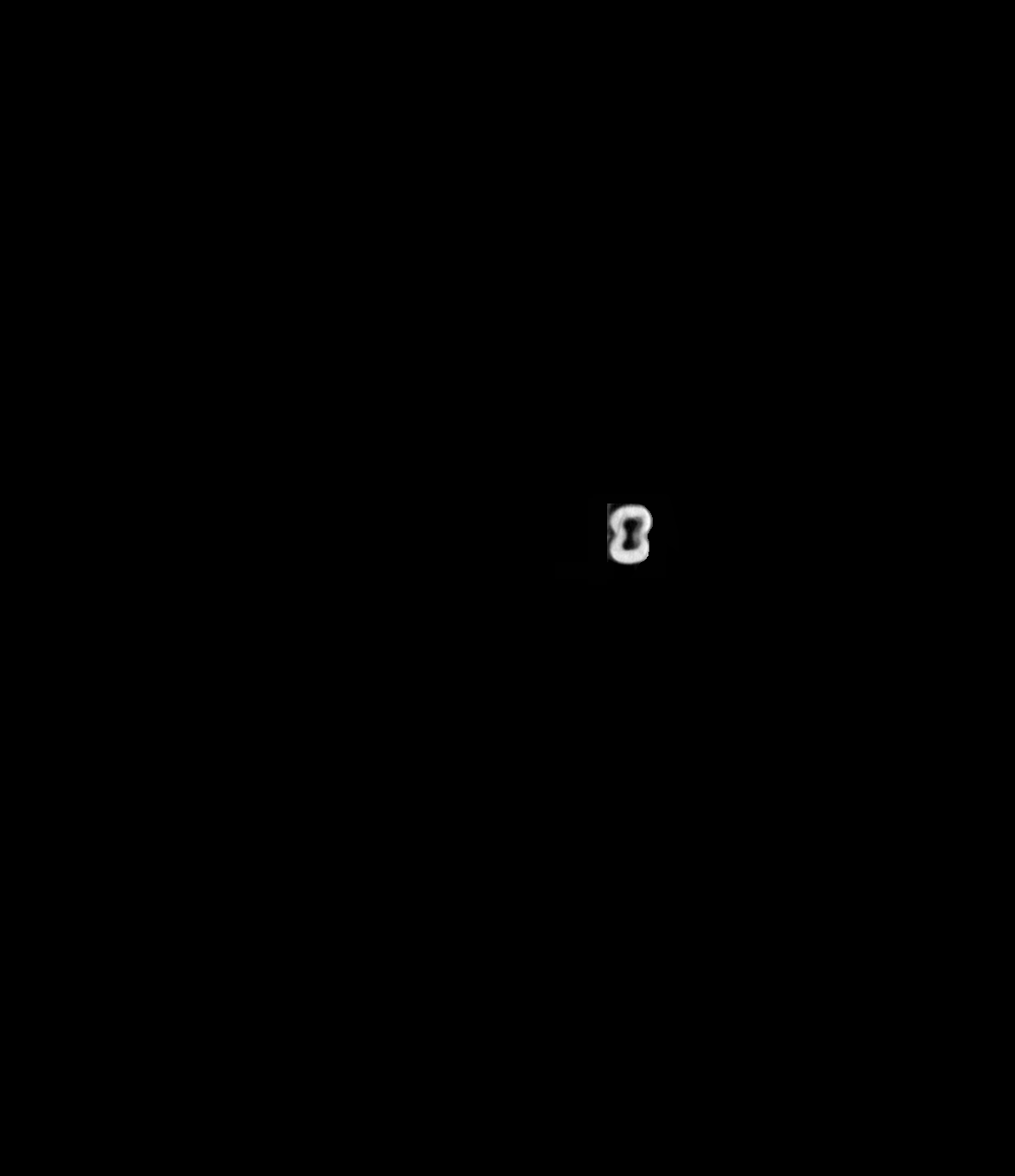

Supplement: Supplementary file 2 — Data S2: Supporting Information. [file AJPA-188-e70164-s001.zip › Cross-Section Tiff Files/mcz_41414_Rm2.tif]

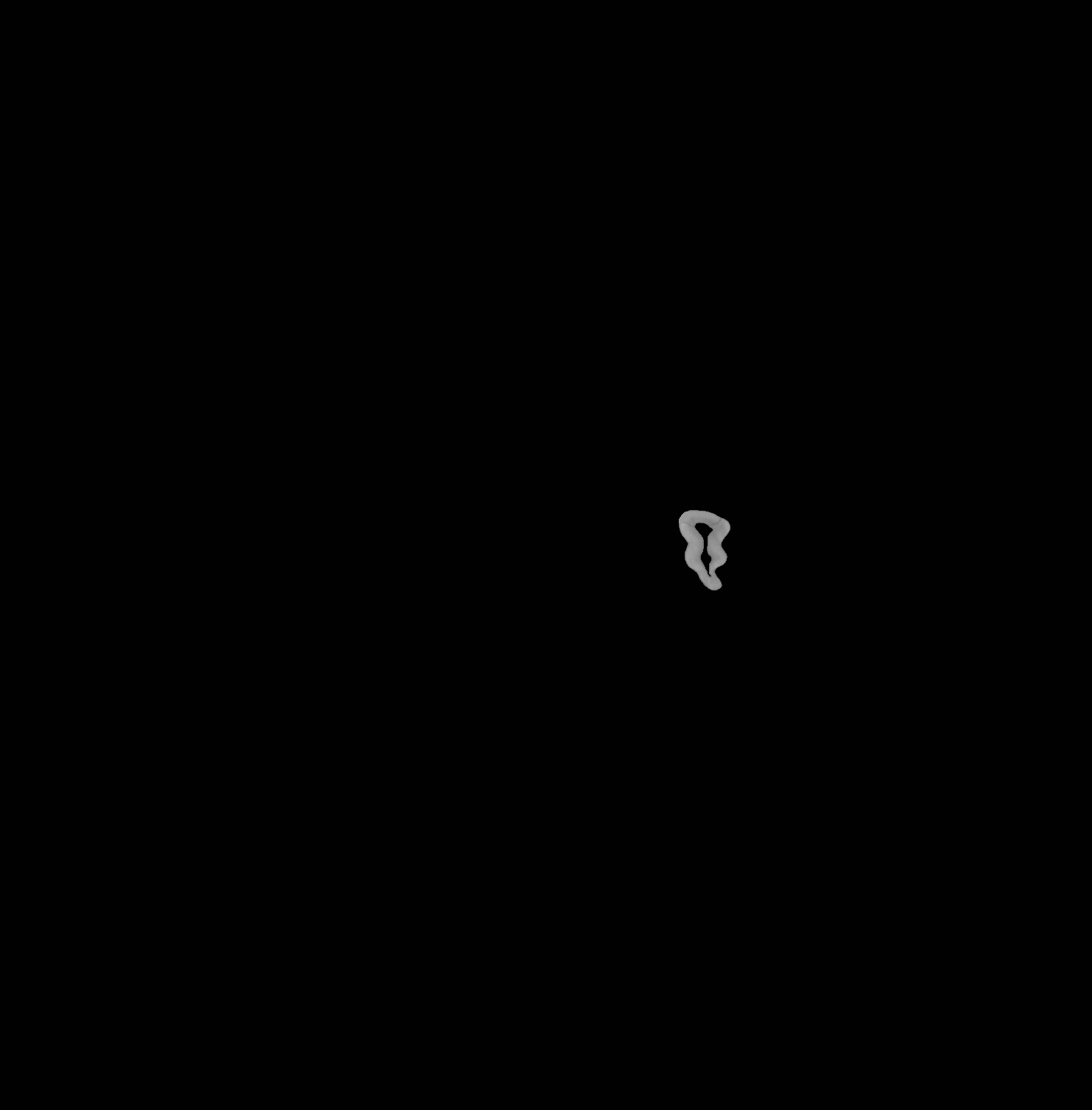

Supplement: Supplementary file 2 — Data S2: Supporting Information. [file AJPA-188-e70164-s001.zip › Cross-Section Tiff Files/amnh_238034_Rm3.tif]

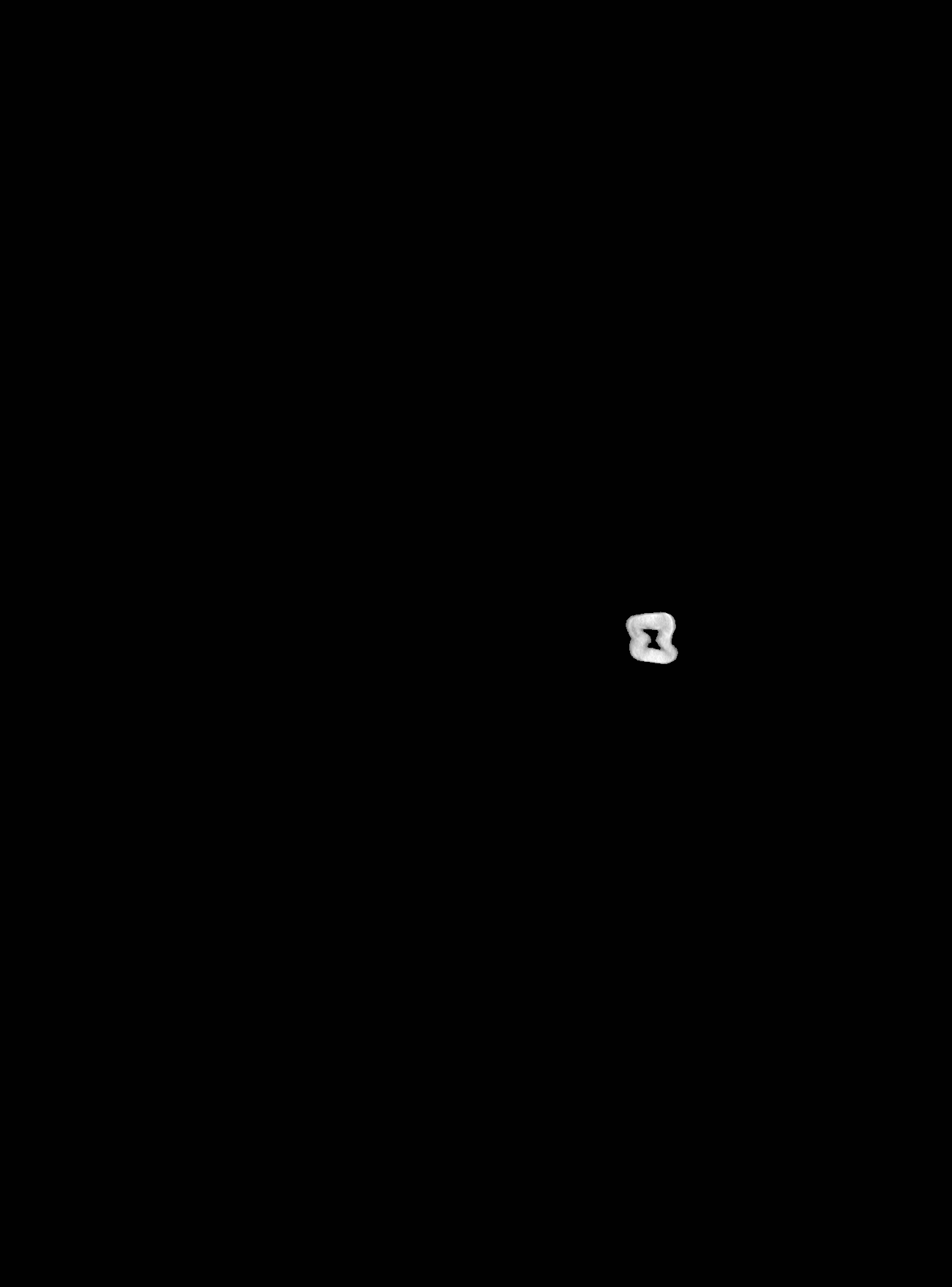

Supplement: Supplementary file 2 — Data S2: Supporting Information. [file AJPA-188-e70164-s001.zip › Cross-Section Tiff Files/mcz_19976_Rm2.tif]

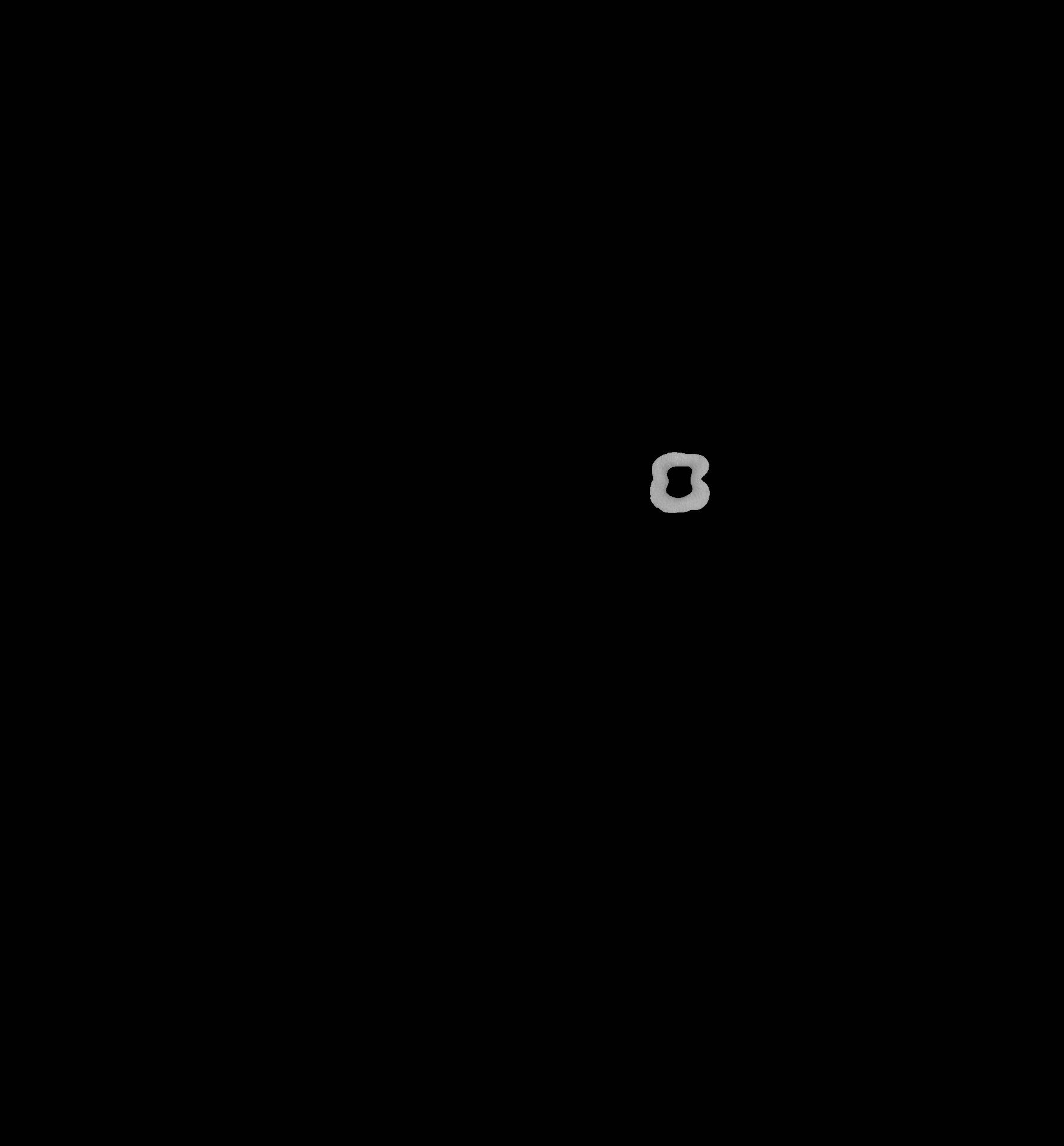

Supplement: Supplementary file 2 — Data S2: Supporting Information. [file AJPA-188-e70164-s001.zip › Cross-Section Tiff Files/mcz_19187_Rm2.tif]

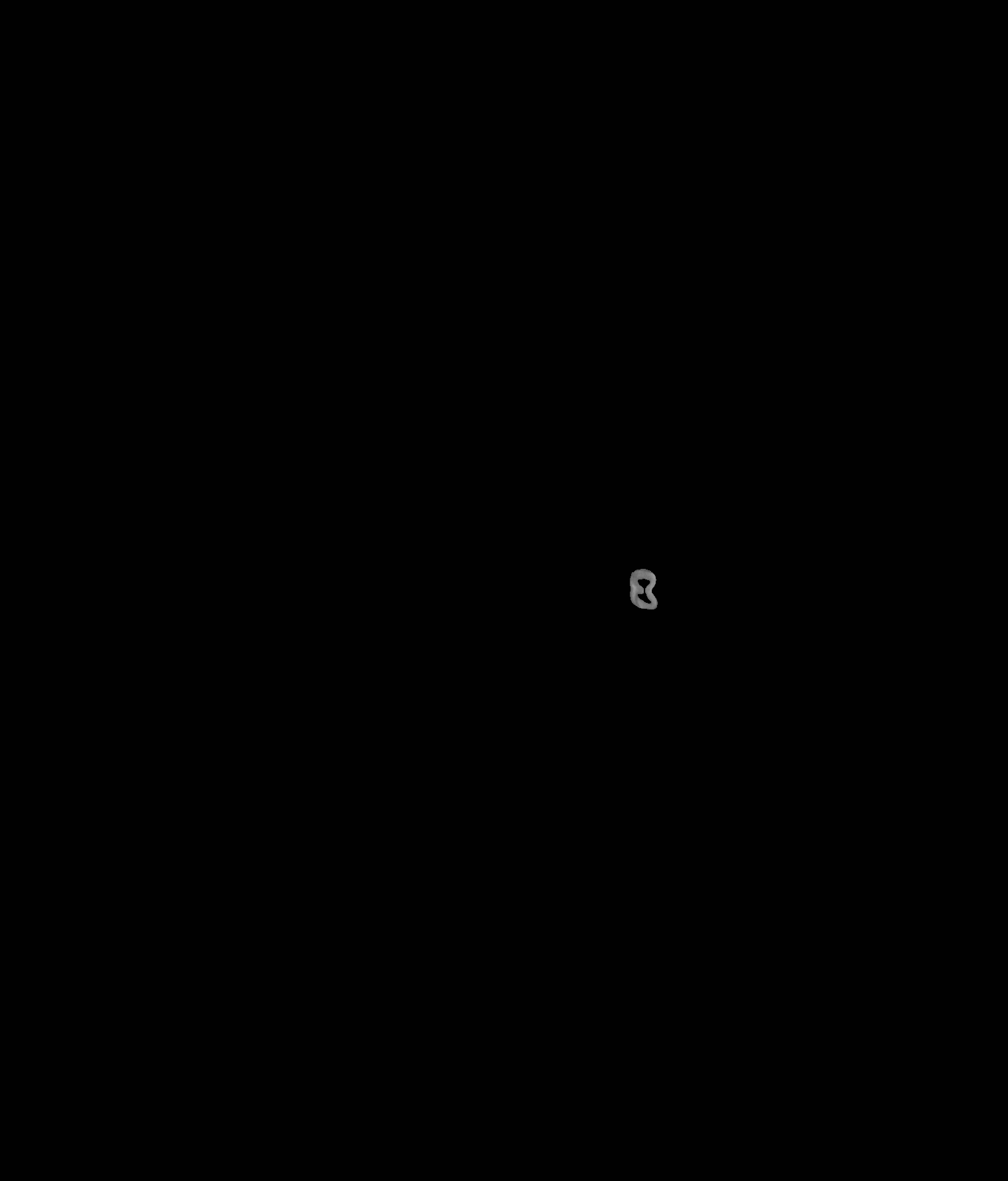

Supplement: Supplementary file 2 — Data S2: Supporting Information. [file AJPA-188-e70164-s001.zip › Cross-Section Tiff Files/mcz_23197_Rm1.tif]

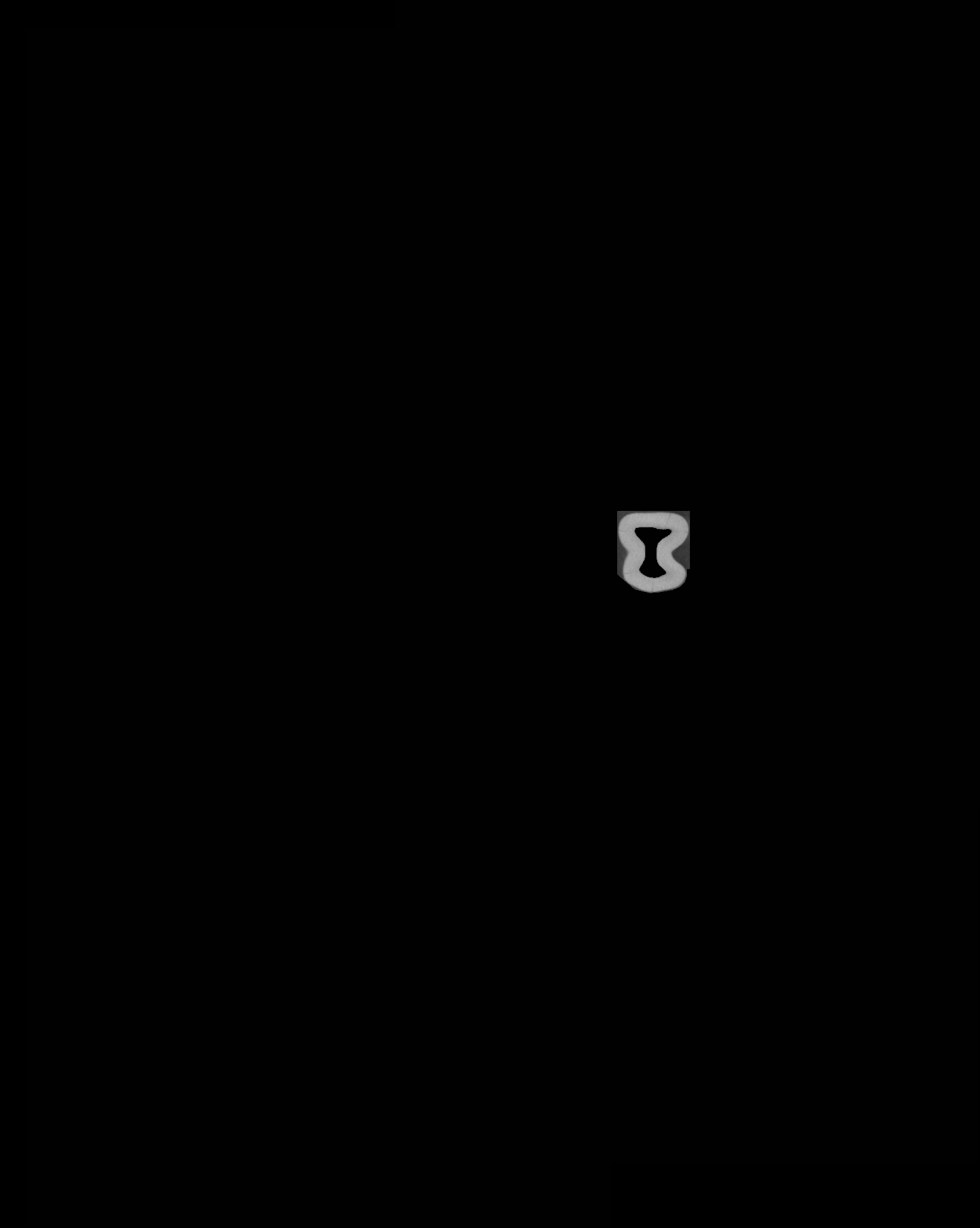

Supplement: Supplementary file 2 — Data S2: Supporting Information. [file AJPA-188-e70164-s001.zip › Cross-Section Tiff Files/mcz_21160_Rm2.tif]

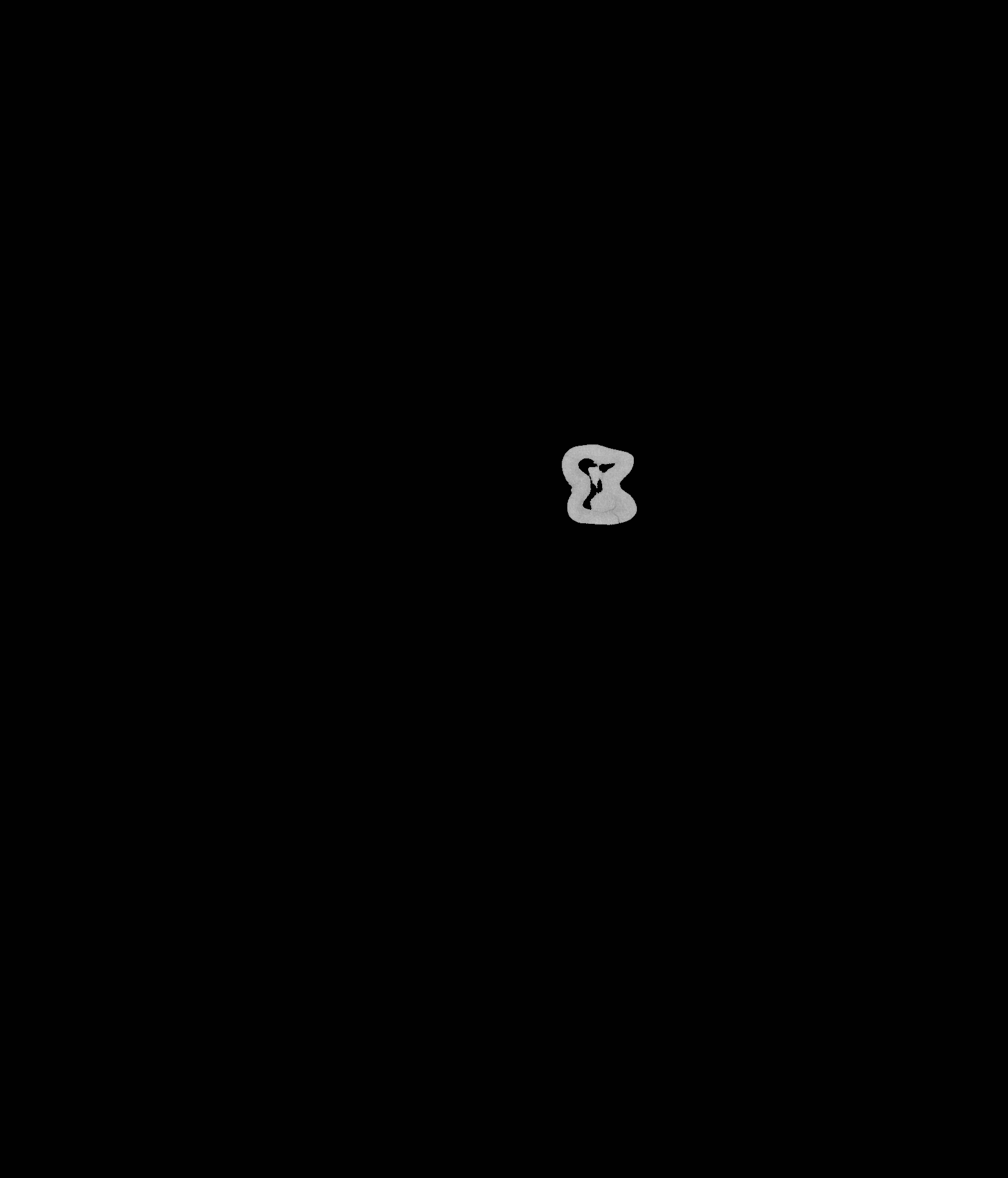

Supplement: Supplementary file 2 — Data S2: Supporting Information. [file AJPA-188-e70164-s001.zip › Cross-Section Tiff Files/mcz_23084_Rm2.tif]

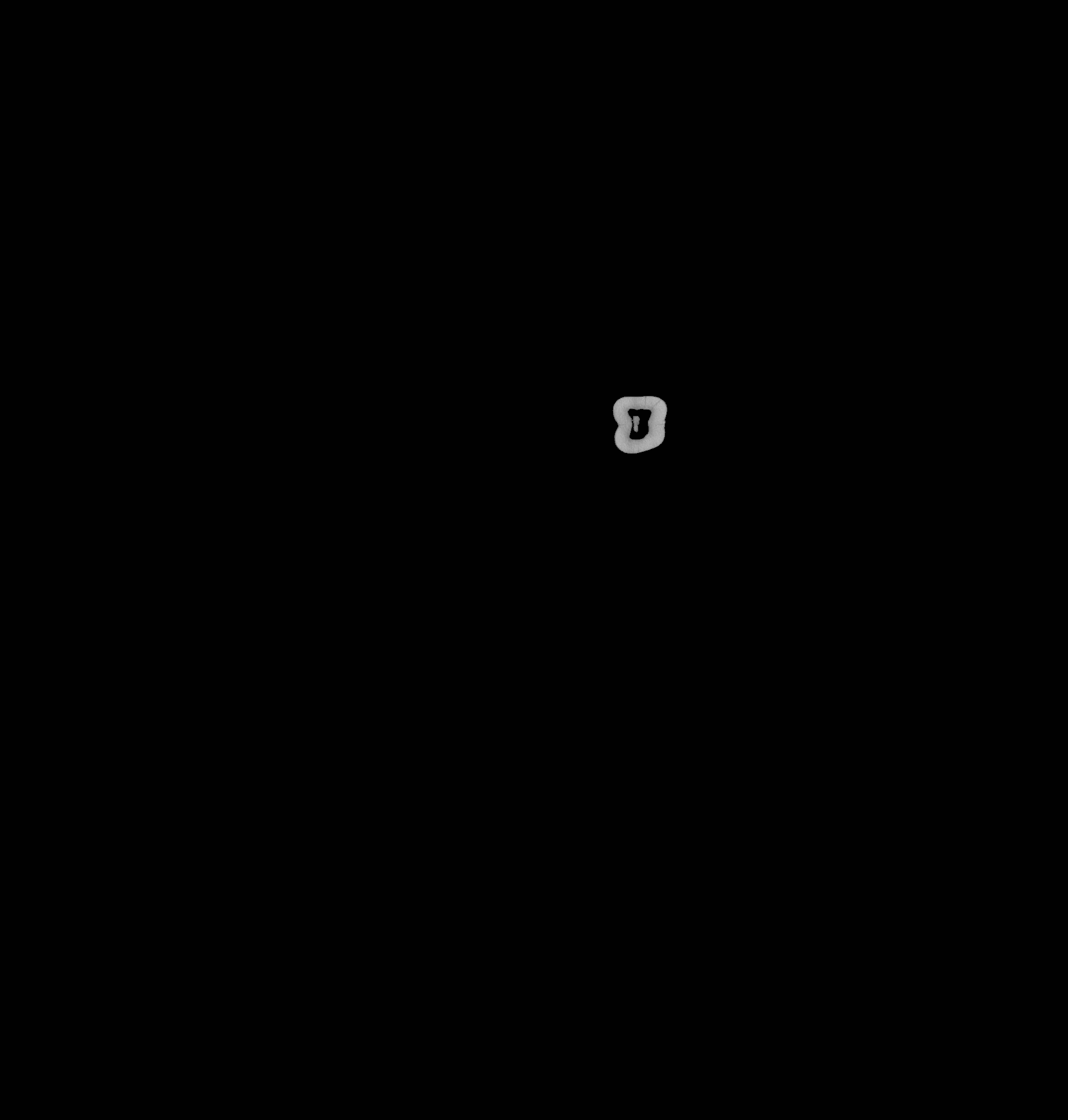

Supplement: Supplementary file 2 — Data S2: Supporting Information. [file AJPA-188-e70164-s001.zip › Cross-Section Tiff Files/mcz_37365_Rm1.tif]
